# Dirhodium Carboxylate Catalysts from 2‐Fenchyloxy or 2‐Menthyloxy Arylacetic Acids: Enantioselective C−H Insertion, Aromatic Addition and Oxonium Ylide Formation/Rearrangement

**DOI:** 10.1002/cctc.202100924

**Published:** 2021-08-23

**Authors:** Aoife M. Buckley, Daniel C. Crowley, Thomas A. Brouder, Alan Ford, U. B. Rao Khandavilli, Simon E. Lawrence, Anita R. Maguire

**Affiliations:** ^1^ School of Chemistry and School of Pharmacy Analytical and Biological Chemistry Research Facility Synthesis and Solid State Pharmaceutical Centre University College Cork Cork Ireland; ^2^ School of Chemistry Analytical and Biological Chemistry Research Facility University College Cork Cork Ireland

**Keywords:** Rhodium Catalysis, Diazo Compounds, Carbenoids, C−H Insertion, Aromatic Addition

## Abstract

A new class of dirhodium carboxylate catalysts have been designed and synthesized from 2‐fenchyloxy or 2‐menthyloxy arylacetic acids which display excellent enantioselectivity across a range of transformations of α‐diazocarbonyl compounds. The catalysts were successfully applied to enantioselective C−H insertion reactions of aryldiazoacetates and α‐diazo‐β‐oxosulfones affording the respective products in up to 93 % ee with excellent trans diastereoselectivity in most cases. Furthermore, efficient desymmetrization in an intramolecular C−H insertion was achieved. In addition, these catalysts prove highly enantioselective for intramolecular aromatic addition with up to 88 % ee, and oxonium ylide formation and rearrangement with up to 74 % ee.

## Graphical abstract

A new class of rhodium carboxylate complexes, which contain phenylacetate ligands substituted with bulky chiral alkoxy substituents, has been prepared, and the application of these complexes in some representative carbene transfer processes of α‐diazocarbonyl compounds is described. Good enantioselectivity is obtained across a range of transformations.

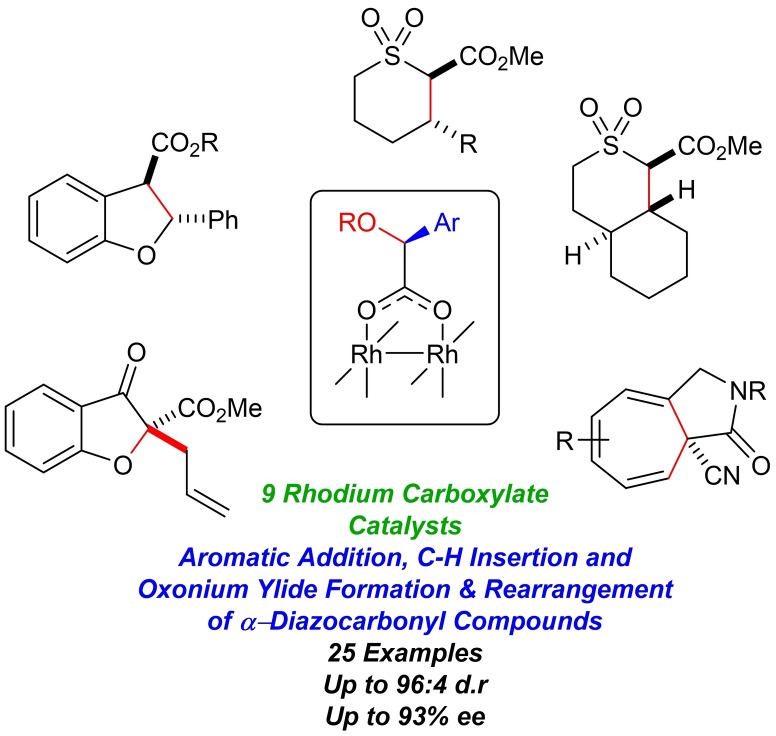

Metal carbenes are versatile intermediates that enable highly selective carbon‐carbon bond forming transformations including cyclopropanation,^[1]^ C−H insertion,^[2]^ aromatic addition,^[3]^ and ylide formation.^[4]^ The synthetic utility of α‐diazocarbonyl compounds as carbene precursors was revolutionized in the early 1980’s by the introduction of rhodium(II) carboxylates as catalysts. The first enantioselective catalysts were reported in 1990, which sparked tremendous progress in the design and development of enantioselective rhodium(II) carboxylates and carboxamidates over the past 30 years, principally for cyclopropanation and C−H insertion.^[5]^

Among the first enantiopure rhodium carboxylates (described by Cotton in 1986) was rhodium mandelate Rh_2_(*S*‐Mand)_4_ **1**,^[6]^ but while early studies proved it to be an efficient catalyst, it led to modest enantioinduction.^[7]^ Later, Moody demonstrated that *O*‐alkyl mandelate rhodium complexes performed better than the parent rhodium mandelate in Si−H insertion reactions, though were still not highly enantioselective.^[8]^ Over the last three decades, Davies, and Ikegami and Hashimoto have developed highly enantioselective rhodium carboxylate catalysts, prolinate‐based Rh_2_(*S*‐DOSP)_4_ **2**,^[9]^ phthaloyl amino acid‐based Rh_2_(*S*‐PTTL)_4_ **3** ^[10]^ and subsequently, analogues such as Rh_2_(*S*‐TCPTTL)_4_ **4**,^[11]^ Rh_2_(*S*‐TPPTTL)_4_ ^[12]^ and the cyclopropanecarboxylate Rh_2_(*R*‐TPCP)_4_ **5** ^[13]^ (Figure 1) for α‐diazocarbonyl transformations. While many highly enantio‐ and diastereoselective dirhodium carboxylate catalysts have been synthesized and evaluated to date, access to a *generally applicable* catalyst with high stereoselectivity across a range of transformations and substrates remains a priority.

**Figure 1:**
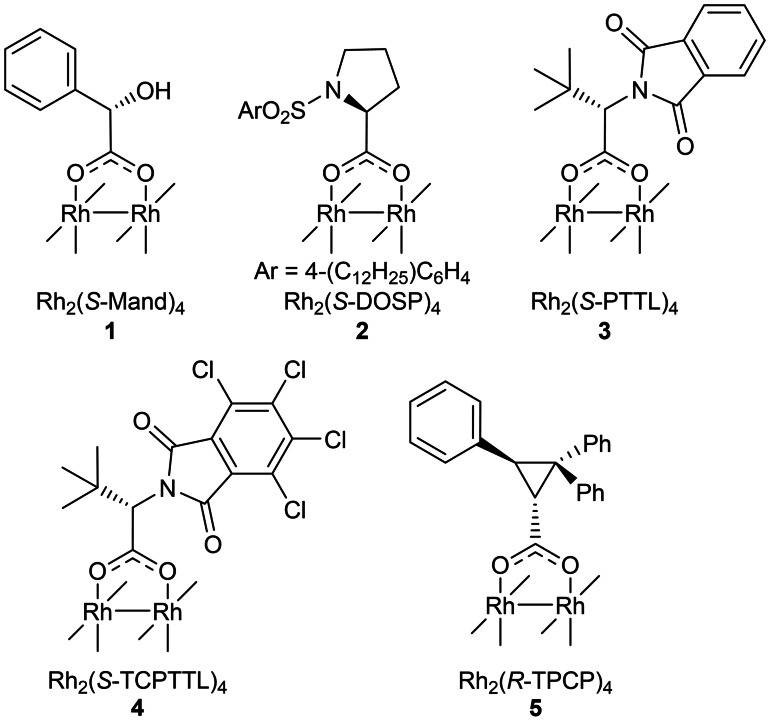
Selected enantiopure dirhodium carboxylate catalysts

Within this work we focused on catalysts structurally related to rhodium mandelate, with the objective of developing enantioselective rhodium carboxylates with broad reaction and substrate scope. Although only limited enantiocontrol was achieved to date with rhodium mandelate,[^7b^, ^7c^, ^8^] variation of the mandelate scaffold is readily achieved through either alteration of the aromatic ring or incorporation of a sterically demanding 2‐alkoxy substituent (derived from enantiopure menthol or fenchol) facilitating access to a series of structurally related rhodium carboxylates. Herein, we report the synthesis of eight novel rhodium carboxylate complexes (Scheme 1, **9 a**–**h**) with 2‐fenchyloxy or 2‐menthyloxy arylacetate ligands, and their application in a range of enantioselective carbene mediated transformations.

**Scheme 1:**
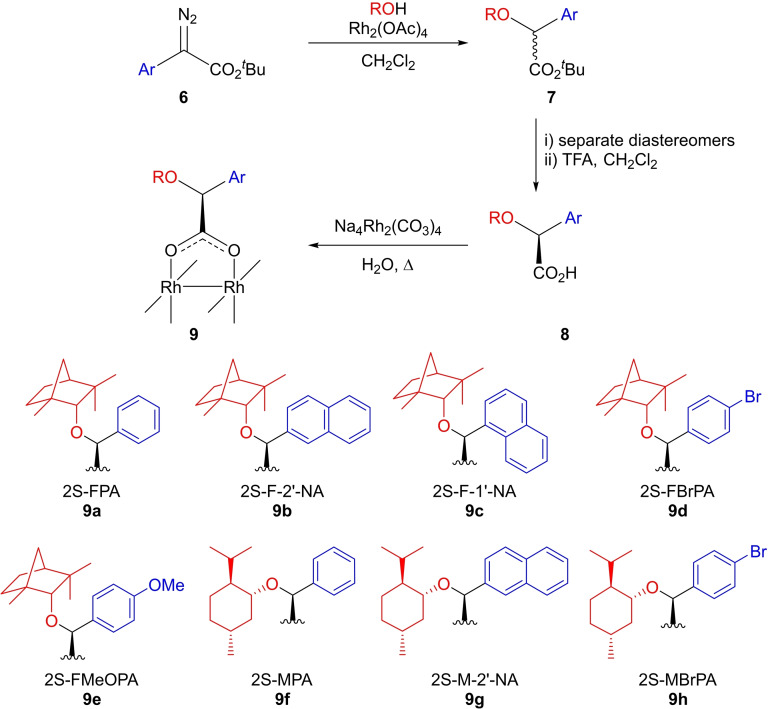
Synthesis of novel enantiopure dirhodium carboxylate complexes

The enantiopure 2*S*‐carboxylic acids (**8**) were prepared from arylacetic acids by esterification, diazo transfer, rhodium acetate mediated O−H insertion into (−)‐menthol or (+)‐fenchol followed by diastereomer separation, and hydrolysis (Scheme 1). In general, the O−H insertion favored the formation of the 2*S*‐diastereomer of the esters (**7**) (typically ∼4 : 1 2*S*/2*R*); following separation of the diastereomers by chromatography and/or recrystallisation, the 2*S* configuration (for **7 b**–**d** and **7 f**–**h**) was determined by X‐ray crystallography. The diastereomerically pure esters (2*S*‐**7**) were then hydrolyzed, and the resulting acids (**8**) were used in ligand exchange with sodium rhodium carbonate^[14]^ to afford the desired rhodium carboxylates (**9 a**–**h**) in 29–79 % yield following chromatographic purification. The green complexes were readily characterized spectroscopically, but efforts to obtain crystals suitable for X‐ray crystallography have been unsuccessful to date.

We investigated the application of these novel catalysts for the construction of three scaffolds found in biologically active compounds, dihydrobenzofurans, tetrahydrothiopyrans and fused heteroaromatics, to exemplify their scope and enantioselectivity (Figure 2).

**Figure 2:**
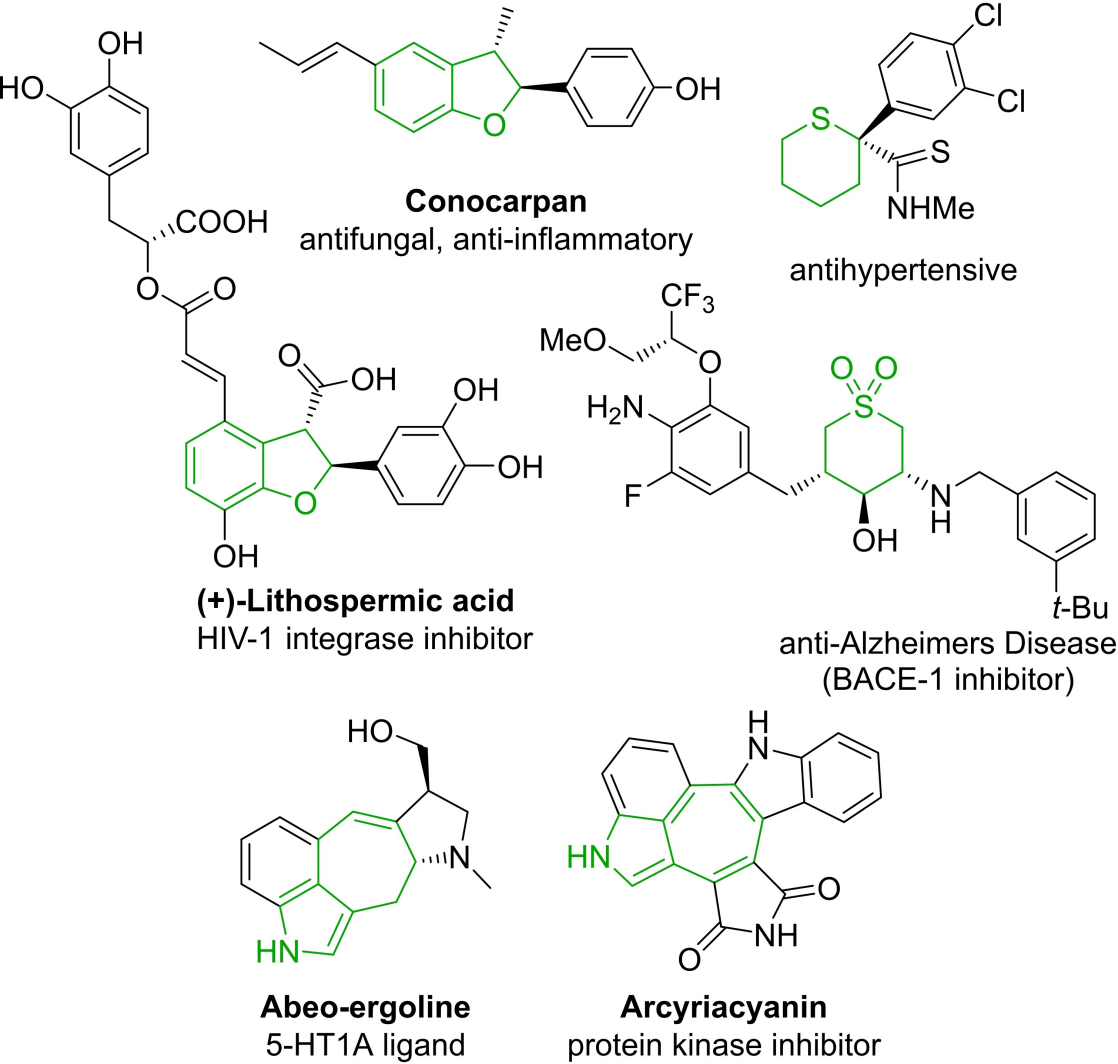
Selected biologically active compounds including dihydrobenzofurans, tetrahydrothiopyrans and fused heteroaromatics.

The dihydrobenzofuran scaffold is a key subunit of many bioactive compounds displaying antioxidant, antibacterial, antiproliferative and anti‐inflammatory effects.^[15]^ While the construction of this moiety has been explored through dehydrative cyclizations, radical and electrocyclizations, biomimetic couplings and cycloadditions, recently, the asymmetric synthesis of this scaffold via metal catalyzed C−H insertion has become more prominent.^[16]^

The rhodium carboxylate catalyzed C−H insertion of aryldiazoacetates to afford the 2,3‐dihydrobenzofuran moiety was first selected for investigation. Davies^[17]^ and Hashimoto^[18]^ have used rhodium complexes to good effect in the synthesis of 2,3‐dihydrobenzofurans, with preferential formation of the cis‐isomer.

Hashimoto has described the intramolecular C−H insertion of **10**, catalyzed by Rh_2_(*S*‐PTTL)_4_ **3** affording the cis dihydrobenzofuran **11 b** with high enantio‐ and diastereoselectivity.^[18]^ When **10** was treated with our novel dirhodium catalysts, the trans‐dihydrobenzofuran **11 a** was preferentially formed with excellent diastereoselectivity (up to 95 : 5 trans:cis ratio) and with high asymmetric induction (up to 93 % ee, Table 1). Optimization studies indicated that our catalysts were efficient at temperatures as low as −45 °C, although raising the reaction temperature to 0–3 °C did not greatly impact on the enantioselectivity. Notably, the isolated trans‐dihydrobenzofuran subunit is more frequently associated with biological activity than the cis isomer.

**Table 1:** Enantioselective rhodium(II) catalyzed C−H insertions of aryldiazoacetate **10**.

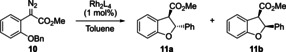							
Entry	Rh^II^ Catalyst	*T* [°C]	d.r. **11 a**:**11 b** Trans : cis	Yield^[a]^		Enantiopurity^[b]^	
				trans **11 a** [%]	cis **11 b** [%]	trans **11 a** (2*R*,3*R*) [% ee]	cis **11 b** (2*S*,3*R*) [% ee]
1	**3**	−60	<1 : 99	–	34	–	95^[c]^
2	**9 a**	−45	85 : 15	65	13	90	69
3	**9 a**	0–3	67 : 33	43	21	87	74
4^[d]^	**9 b**	−45	84 : 16	21	4	93	75
5	**9 c**	−45	6 : 94	–	69	–	79
6	**9 d**	−45	88 : 12	42	6	80	54
7	**9 e**	−45	86 : 14	32	5	87	–
8	**9 f**	−60 to −45	90 : 10	32	4	84	10
9	**9 g**	−45	94 : 6	50	4	86	18
10	**9 h**	−45	95 : 5	47	2	87	18

[a] Isolated yields after chromatography. [b] The enantiomeric excess was determined by chiral phase HPLC analysis (for full detail see the Supporting Information). [c] Stereochemistry determined to be 2*R*,3*S*. [d] Reaction performed with 0.35 mol% catalyst.

Encouraged by these preliminary results, the substrate scope was extended to include two further aryldiazoacetate analogues, benzyl ester **12** and isopropyl ester **13** (Table 2). Moderate yields of the C−H insertion products recorded throughout this study may be attributed to competing reaction pathways (see Supporting Information for details) although typically, only the desired 2,3‐dihydrobenzofuran products were isolated following purification of the reaction mixture. To improve selectivity for C−H insertion, the reactions were conducted at 0–3 °C, and for each of the catalysts **9 a**–**h**, other than **9 c**, formation of the trans isomer was favored. Furthermore, we were gratified to find that high levels of asymmetric induction (up to 91 % ee) were achieved for each of the trans 2,3‐dihydrobenzofurans (**11 a**, **14 a** and **15 a**) with all of the catalysts **9 a**–**h**, with remarkable consistency across both the substrate and catalyst range. For the cis isomers (**11 b**, **14 b** and **15 b**), in general, the enantioselectivity was lower than that seen for the trans isomers, and decreased slightly with increasing steric demand of the ester group.

**Table 2:** Enantioselective rhodium catalyzed C−H insertions of aryldiazoacetates **10**, **12** and **13**.

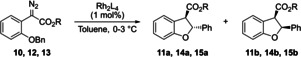							
Entry	R	Rh^II^ Catalyst	d.r. trans cis	Yield^[a]^		Enantiopurity^[b]^	
				trans [%]	cis [%]	trans 2*R*,3*R* [% ee]	cis 2*S*,3*R* [% ee]
1	Me **10**, **11**	**9 a**	67 : 33	43	21	87	74
2		**9 b**	65 : 35	36	20	82	77
3		**9 c**	7 : 93	2	43	83	65
4		**9 d**	85 : 15	48	10	77	59
5		**9 e**	67 : 33	31	15	83	68
6		**9 f**	84 : 16	36	6	80	33
7		**9 g**	87 : 13	38	6	83	42
8		**9 h**	91 : 9	58	4	84	31
9	Bn **12**, **14**	**9 a**	62 : 38	41	24	78	63
10		**9 b**	64 : 36	44	26	81	65
11		**9 c**	22 : 78	8	29	89	55
12		**9 d**	86 : 14	34	6	69	40
13		**9 e**	63 : 37	34	8	–^[c]^	59
14		**9 f**	75 : 25	54	16	79	22
15		**9 g**	85 : 15	50	8	79	42
16		**9 h**	89 : 11	48	2	77	21
17	^*i*^Pr **13**, **15**	**9 a**	58 : 42	39	28	87	79
18		**9 b**	60 : 40	24	14	86	79
19		**9 c**	9 : 91	3	31	91	39
20		**9 d**	82 : 18	66	12	75	50
21		**9 e**	66 : 34	29	15	87	72
22		**9 f**	69 : 31	35	17	78	27
23		**9 g**	78 : 22	30	8	83	22
24		**9 h**	88 : 12	57	7	77	16

[a] Isolated yields after chromatography. [b] The enantiomeric excess was determined by chiral phase HPLC analysis (for full detail see the Supporting Information). [c] A sample of sufficient purity to allow accurate determination of enantiopurity was not isolated.

Variation of the aryl substituent on the ligand (catalysts **9 a**–**c**) had little impact on the enantioselectivity of the C−H insertion to form the trans 2,3‐dihydrobenzofurans (**11 a**, **14 a** and **15 a**) however, using catalyst **9 c** with the sterically demanding 1‐naphthyl substituent, there was a dramatic change in diastereoselectivity leading preferentially to the cis isomer (**11 b**, **14 b** and **15 b**), and in parallel leading to the highest enantioselectivity in the formation of the trans isomers **14 a** and **15 a**, potentially indicating a different conformation in the catalyst **9 c** relative to those of the other catalysts.[^2a^, ^19^] Interestingly, the diastereoselectivity of **9 c** is similar to that seen with Rh_2_(PTTL)_4_ **3** (Table 1, entries 1 and 5) which might suggest common structural features in these catalysts in contrast to catalysts **9 a**,**b**,**d–h**; the conformational properties of Rh_2_(PTTL)_4_ **3** have been explored.[^19f^, ^19g^]

Introduction of an electron donating methoxy substituent on the aromatic ring of the ligand (**9 e**) had little impact on enantioselectivity, while a bromo substituent (**9 d**) led to reduced enantioselectivity for both cis and trans isomers across all three substrates (**11**, **14** and **15**) relative to **9 a**.

Comparing the fenchol‐ and menthol‐derived catalyst pairs (**9 a/f**, **9 b/g**, **9 d/h**) use of **9 h** lead to a slight increase in enantioselectivity for each dihydrobenzofuran analogue relative to **9 d**, while no discernable trends in the formation of the trans diastereoisomer were observed across the catalyst pairs. In contrast, for the cis dihydrobenzofurans, higher levels of asymmetric induction were achieved in all instances where a fenchol derived catalyst was used relative to its menthyl counterpart.

To further investigate the scope of catalysts **9 a**–**h**, the C−H insertion of α‐diazo‐β‐oxosulfones to form tetrahydrothiopyran dioxides was next examined. The tetrahydrothiopyran scaffold has been previously synthesized via enantioselective Michael‐Michael cascade reactions,^[20]^ the addition of hydrogen sulfide to divinyl ketones,^[21]^ S_N_2 cyclisation using sodium sulfide,^[22]^ intramolecular Michael addition,^[23]^ and intramolecular rhodium catalyzed C−H insertion.^[24]^ While intramolecular C−H insertion of α‐diazocarbonyl compounds generally leads to the formation of 5‐membered heterocycles and carbocycles,^[25]^ Du Bois noted that when a sulfone group is incorporated into the cyclized product, six‐membered rings are formed due to the conformational impact of the sulfonyl moiety in the transition state.^[26]^ Rhodium and iron catalysts have been utilized in the synthesis of sulfur containing 6‐membered heterocycles from α‐diazocarbonyl compounds,[^24^, ^27^] however, high levels of enantioselectivity were not achieved.^[28]^ In 2010, we described the use of copper‐bis(oxazoline) catalysts which led to the formation of cis‐tetrahydrothiopyran dioxides in up to 98 % ee.^[29]^ In contrast, to date, rhodium catalysts have afforded the trans‐tetrahydrothiopyran dioxide diastereoisomer from α‐diazo‐α‐sulfonyl esters, with poor enantioselectivity.^[28]^ Our aim was to induce high levels of stereocontrol in the synthesis of trans‐tetrahydrothiopyran dioxides for the first time using α‐diazo‐β‐oxosulfones.

An initial investigation of the intramolecular C−H insertion of α‐diazo‐β‐oxosulfone **16** with Rh_2_(*S*‐DOSP)_4_ **2** and Rh_2_(*S*‐PTTL)_4_ **3** at room temperature in dichloromethane, resulted in modest enantioselectivity, while Rh_2_(TCPTTL)_4_ **5** led to **17 a** in 91 % ee; interestingly Rh_2_(*S*‐PTTL)_4_ **3** forms preferentially cis‐**17 b** while all other catalysts explored led selectively to trans‐**17 a** (Table 3). Relative to the widely‐used Hashimoto catalysts (**3**, **5**), improved trans diastereoselectivity was seen for the novel rhodium carboxylate catalysts **9 b**, **9 d**–**h** (up to 89 : 11 d.r.), with up to 86 % ee observed for **9 b** or **9 g** in dichloromethane at room temperature (Table 3, entries 4 and 9). Notably, the rhodium carboxylates bearing the 2‐naphthyl substituent (**9 b** and **9 g**) led to the highest enantioselectivities. Once again, the diastereoselectivity seen with **9 c** differed substantially from that seen with all of the other catalysts (Table 3, entry 5). Excellent enantioselectivity was also observed in toluene, albeit with decreased efficiency (Table 3, entry 11).

**Table 3:** Enantioselective rhodium catalyzed C−H insertions of α‐diazo‐β‐oxosulfone **16**.

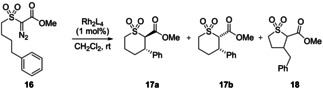						
Entry	Rh^II^ Catalyst	d.r. **17 a : 17 b**	Yield^[a]^ **17 a** [%]	Yield^[a]^ **17 b** [%]	Yield^[a]^ **18** [%]	% ee^[b]^ **17 a** (2*R*,3*S*)
1	**2**	86 : 14	40	5	14	31
2	**3**	34 : 66	17	30	26	33
3	**5**	75 : 25	42	15	29	91
4	**9 b**	85 : 15	40	7	19	86
5	**9 c**	59 : 41	12	13	8	78
6	**9 d**	86 : 14	40	8	14	72
7	**9 e**	84 : 16	31	6	10	74
8	**9 f**	87 : 13	17	5	8	75
9	**9 g**	89 : 11	24	8	16	86
10	**9 h**	86 : 14	36	6	7	74
11^[c]^	**9 b**	85 : 15	20	–	–	89
12 ^[d]^	**9 b**	90 : 10	41	5	21	92
13^[d,e]^	**9 b**	92 : 8	64	4	23	92

[a] Isolated yields after chromatography. [b] The enantiomeric excess was determined by chiral phase HPLC analysis (for full detail see the Supporting Information). [c] Reaction performed in toluene. [d] Reaction performed at −20 °C and with 4 Å molecular sieves. [e] Reaction conducted with 1 g of **16** (3.4 mmol).

The modest yields observed in these reactions can be attributed to the poor solubility of trans‐**17 a**, and competing side reactions including C−H insertion leading to the 5‐membered tetrahydrothiophene dioxide **18**. Enhanced diastereoselectivity (up to 90 : 10 d.r.) and enantioselectivity (up to 92 % ee) in the Rh_2_(2*S*‐F‐2′‐NA)_4_ (**9 b**) catalyzed formation of trans‐**17 a**, were achieved through rigorous exclusion of oxygen, and addition of 4 Å molecular sieves in dichloromethane at −20 °C (Table 3, entry 12). Notably, when the reaction was carried out on a 1 g scale (3.4 mmol), an improved yield (64 %) was obtained, while retaining the high level of diastereoselectivity (92 : 8 d.r.) and enantioselectivity (92 % ee) (Table 3, entry 13). This is the highest level of enantioselectivity recorded to date in the synthesis of a trans‐tetrahydrothiopyran dioxide by C−H insertion of an α‐diazocarbonyl compound.

With the optimized conditions in hand, rhodium(II) catalyzed C−H insertion of a series of α‐diazo‐β‐oxosulfones was examined (Table 4). The diastereoselectivity and enantioselectivity of intramolecular C−H insertion catalyzed by Rh_2_(2*S*‐F‐2′‐NA)_4_ **9 b** with a 4‐methyl or 4‐fluoro substituent on the aryl ring of the substrate were comparable to those seen in the unsubstituted derivative (89–92 % ee, **25 a**, **27 a**, **17 a**), while decreased enantioselectivity was observed in the presence of a 4‐methoxy substituent (51 % ee, **26 a**). The extent of competing hydride transfer from the benzylic position increased with the electron donating 4‐methyl and 4‐methoxy substrates relative to the unsubstituted derivative (see Supporting Information for details). The ester functionality of these substrates appears to be essential; the ketone derivative **28 a** required more forcing conditions leading to an isolated yield of only 1 %, although the ee was 69 %. The absolute stereochemistry of **27 a** was determined to be 2*R*,3*S* by X‐ray crystallography.

**Table 4:**
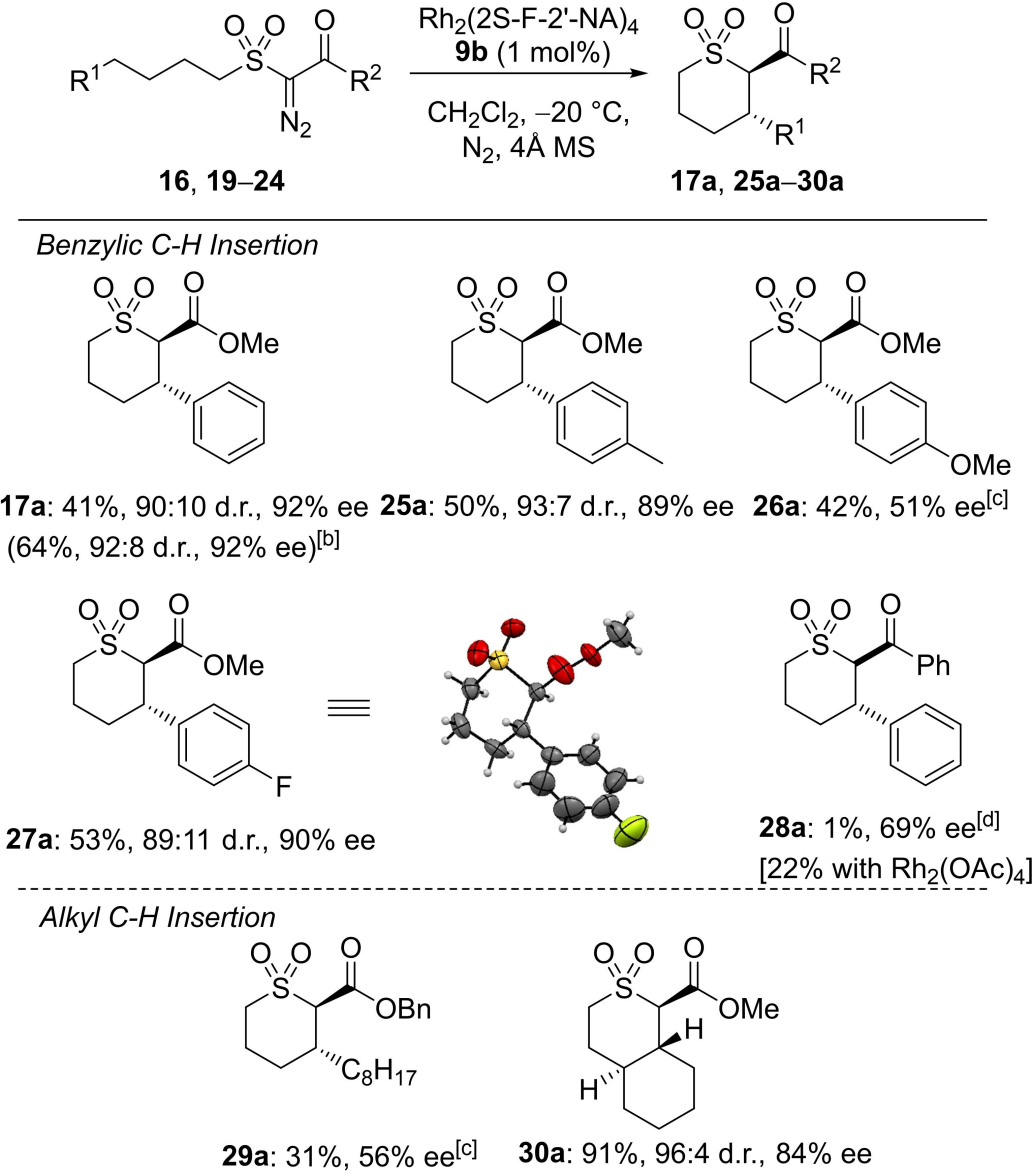
Enantioselective rhodium catalyzed C−H insertion of α‐diazo‐β‐oxosulfones **16**, **19**–**24**.^[a]^

[a] Isolated yields after chromatography. [b] Reaction conducted with 1 g of **16** (3.4 mmol). [c] The d.r. of **26 a** and **29 a** could not be determined due to signal overlap in the ^1^H NMR spectrum of the crude product mixture. [d] Reaction conducted under reflux.

In contrast to insertion at a benzylic position, the selectivity of insertion into an unactivated C−H bond was decreased, leading to the trans‐tetrahydrothiopyran dioxide **29 a** in only 31 % yield, with the corresponding cis‐tetrahydrothiopyran dioxide and 5‐membered tetrahydrothiophene dioxide formed through competing reaction pathways (see Supporting Information for details). Notably, use of Rh_2_(2*S*‐F‐2′‐NA)_4_ **9 b** led to efficient desymmetrization, with **30 a** isolated in 91 % yield, 96 : 4 d.r. (trans:cis‐tetrahydrothiopyran dioxide) and 84 % ee; the highest enantioselectivity achieved to date for a trans substituted thiopyran dioxide formed by insertion into an alkyl C−H bond, while poor enantioselectivity was achieved in this transformation with the widely used catalysts Rh_2_(*S*‐DOSP)_4_ **2**, Rh_2_(*S*‐PTTL)_4_ **3**, Rh_2_(*S*‐TCPTTL)_4_ **4** and Rh_2_(S‐PTPA)_4_ (see Supporting Information for details). As we recently reported, use of a chiral copper catalyst led to highly selective desymmetrization to form the complementary (1*S*,4a*S*,8a*R*) diastereoisomer of the cis‐tetrahydrothiopyran dioxide **30 b**;^[29c]^ accordingly access to either diastereoisomer with excellent diastereo‐ and enantioselectivity can be achieved by appropriate selection of the rhodium or copper catalyst (Scheme 2). Similarly, with the acyclic α‐diazo‐β‐oxosulfones **16**, **19**–**23** access to either the cis or trans diastereomer of tetrahydrothiopyran dioxide in highly enantioenriched form can be achieved through appropriate choice of the rhodium or copper catalyst.^[29a]^

**Scheme 2:**
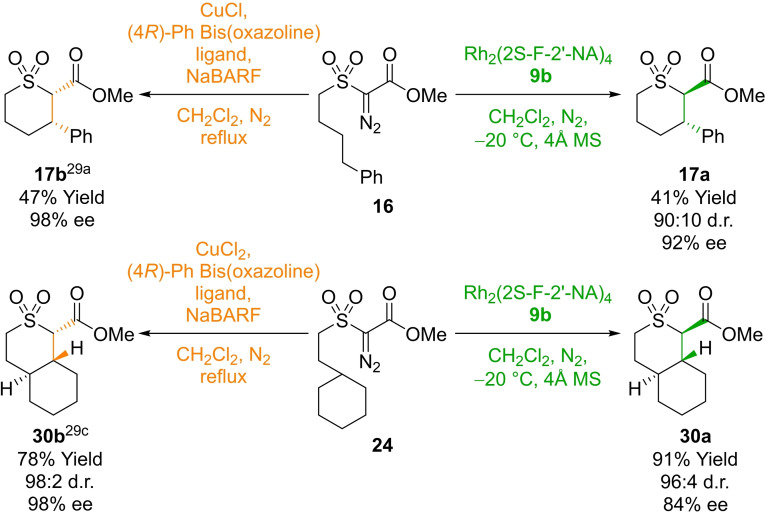
Enantioselective C−H insertion of **16** and **24** with a copper‐bis(oxazoline) catalyst and novel dirhodium carboxylate catalyst **9 b**

The novel rhodium catalysts were next applied to intramolecular aromatic addition leading to the formation of the 9‐azabicyclo[5.3.0]decane skeleton focusing on derivatives bearing a cyano substituent at the bridgehead position which open up the possibility of further functionalisation.[^3^, ^30^] Excellent yields and enantioselectivies have been achieved in the intramolecular aromatic addition using rhodium and other transition metal catalysts, but with simple alkyl bridgehead substituents.^[31]^ Notably, while this work was underway, the first report of transition metal catalyzed intramolecular aromatic additions affording products bearing a nitrile moiety at the enantioenriched bridgehead position appeared.^[32]^

An optimization study was conducted with α‐cyano‐α‐diazoacetamide **31** and Rh_2_(2*S*‐F‐2′‐NA)_4_ **9 b** (85 %, 67 % ee, Scheme 3) in dichloromethane, with optimal results achieved at lower temperatures (see Supporting Information for details).

A catalyst screen using these conditions with α‐cyano‐α‐diazoacetamide **31** found that the highest enantioselectivities were achieved with catalysts **9 b** and **9 e**; Rh_2_(2*S*‐FMeOPA)_4_ **9 e** afforded the aza‐azulenone **32** with the highest enantiopurity (Scheme 3, 90 %, 73 % ee). Notably all seven novel rhodium catalysts afforded higher enantioselectivies than those obtained with four commercially available dirhodium catalysts screened (see Supporting Information for details).

**Scheme 3:**
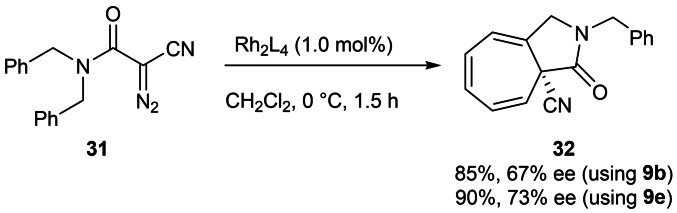
Aromatic addition of α‐cyano‐α‐diazoacetamide **31** using novel rhodium catalysts **9 b** and **9 e**.

A range of aza‐azulenones (**32**, **46**–**58**) were synthesized using the best performing catalysts, Rh_2_(2*S*‐FMeOPA)_4_ **9 e** and Rh_2_(2*S*‐F‐2′‐NA)_4_ **9 b**, which were afforded in excellent yields with enantioselectivities up to 88 % ee (Table 5), comparable to those recently reported.^[32]^ Halogenated products (**46**–**49**) were afforded with the best enantioselectivities (83–87 % ee) followed by alkyl‐substituted derivatives (**50**–**52**). While across the series, transformations were routinely carried out on a 0.3 mmol scale with 1 mol% of catalyst, fluorinated aza‐azulenone **46** was synthesized using 1.0 g (3.36 mmol) of the corresponding α‐cyano‐α‐diazoacetamide **33** and catalyzed with only 0.05 mol% of Rh_2_(2*S*‐FMeOPA)_4_ **9 e** while maintaining both the yield and enantioselectivity achieved in the initial substrate screen. Across the series, the 1*S* enantiomer dominates with both catalysts, however, interestingly, for aza‐azulenones **54** and **56**, the 1*R* enantiomer is favored with both Rh_2_(2*S*‐FMeOPA)_4_ **9 e** and Rh_2_(2*S*‐F‐2′‐NA)_4_ **9 b**, albeit with modest enantiopurity.

**Table 5:**
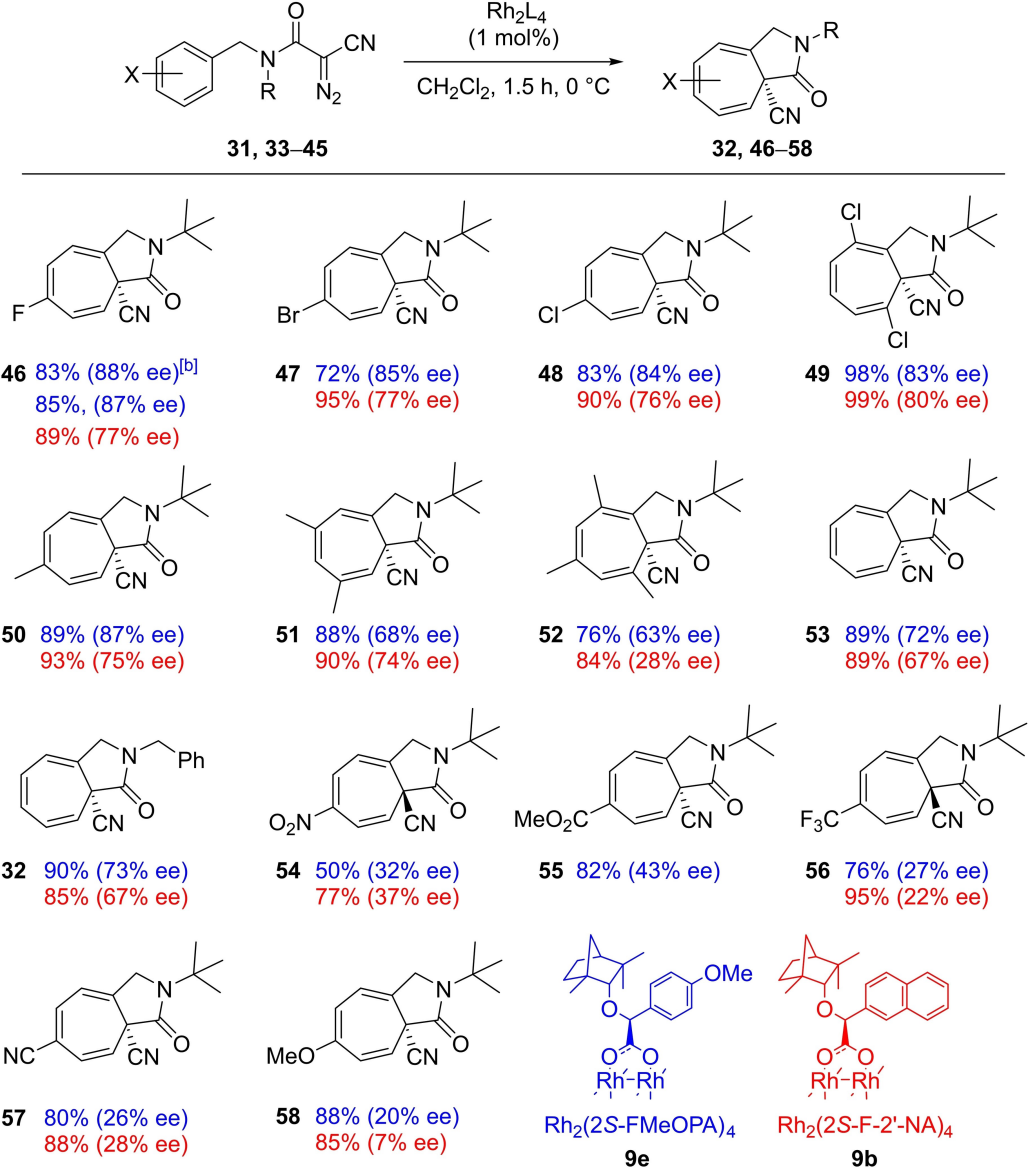
Enantioselective rhodium catalyzed aromatic addition reactions of α‐cyano‐α‐diazoacetamides **31**, **33**–**45**.^[a]^

[a] Isolated yields after chromatography. The enantiomeric excess was determined by chiral phase HPLC analysis (for full detail, including Rh_2_(OAc)_4_ catalyzed reactions, see the Supporting Information). [b] Reaction performed on 3.36 mmol scale with 0.05 mol% of catalyst **9 e**.

The aza‐azulenones generated were functionalized further by various transformations including a Diels‐Alder cycloaddition and a Suzuki cross‐coupling reaction performed with racemic samples of azulenones **32** and **47** (Scheme 4). To exemplify the synthetic versatility of the bridgehead nitrile substituent, an enantioenriched sample of aza‐azulenone **50** was selected to undergo methanolysis affording azulenone **61** in 40 % yield with 77 % ee (Scheme 4).

**Scheme 4:**
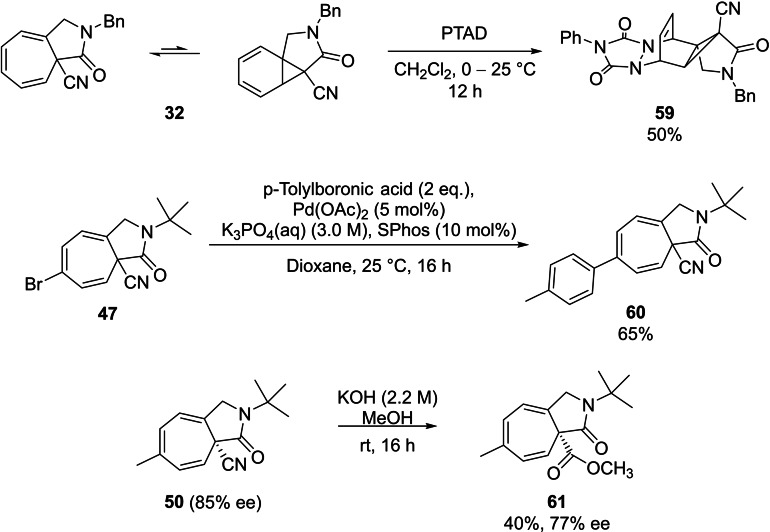
Transformations of aza‐azulenones **32**, **47** and **50**.

In addition to the C−H insertion and aromatic addition processes, as illustrated in Scheme 5, the enantioselective oxonium ylide formation and [2,3]‐sigmatropic rearrangement from **62** can be effected with up to 74 % ee using **9 b** highlighting the broad scope of the novel dirhodium carboxylates, the highest enantioselectivity in the diazo derived oxonium ylide formation and [2,3]‐rearrangement leading to **63** to date.^[33]^

**Scheme 5:**
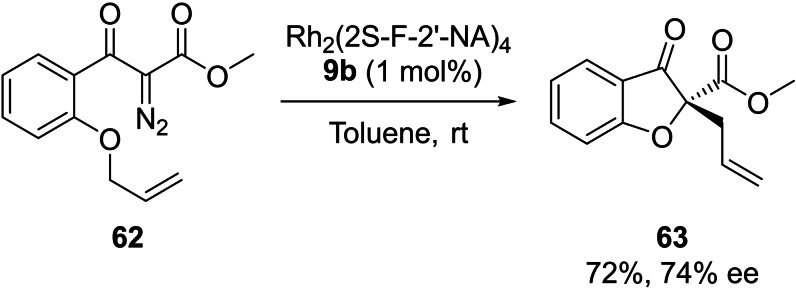
Enantioselective rhodium catalysed oxonium ylide [2,3]‐sigmatropic rearrangement reaction of **62**.

In conclusion, the novel rhodium carboxylate catalysts (**9 a**–**h**) provide high levels of enantioselectivity across a range of transformations of α‐diazocarbonyl compounds, including C−H insertions to form dihydrobenzofurans (up to 93 % ee) and tetrahydrothiopyran dioxides (up to 92 % ee), aromatic addition to form aza‐azulenones (up to 88 % ee), and oxonium ylide [2,3]‐sigmatropic rearrangement to form a dihydrobenzofuranone (up to 74 % ee), highlighting the merit of combining the mandelate framework with an additional enantiopure moiety linked through the oxygen atom, in the catalyst design. Clearly these results represent substantial progress in the search to identify a *generally applicable* stereoselective catalyst which is effective across a range of transformations. Furthermore, in some instances, the novel catalysts offer access to complementary enantioenriched diastereoisomers compared to the commercially available rhodium catalysts. Work is underway to obtain structural data to facilitate rationalisation of the observed patterns of stereoselectivity.

## Conflict of interest

The authors declare no conflict of interest.

## Supporting information

As a service to our authors and readers, this journal provides supporting information supplied by the authors. Such materials are peer reviewed and may be re‐organized for online delivery, but are not copy‐edited or typeset. Technical support issues arising from supporting information (other than missing files) should be addressed to the authors.

Supporting Information
