# Supplementary material for: Dirhodium Carboxylate Catalysts from 2‐Fenchyloxy or 2‐Menthyloxy Arylacetic Acids: Enantioselective C−H Insertion, Aromatic Addition and Oxonium Ylide Formation/Rearrangement
Source: ChemCatChem. 2021 Aug 23;13(20):4318–24. doi: 10.1002/cctc.202100924 (PMC8597163; doi:10.1002/cctc.202100924)
Supplement: Supplementary file 1 — Supporting Information [file CCTC-13-4318-s001.pdf]

ChemCatChem

Supporting Information

Dirhodium Carboxylate Catalysts from 2-Fenchyloxy or 2-Menthyloxy Arylacetic Acids: Enantioselective C–H Insertion, Aromatic Addition and Oxonium Ylide Formation/Rearrangement

Aoife M. Buckley, Daniel C. Crowley, Thomas A. Brouder, Alan Ford, U. B. Rao Khandavilli, Simon E. Lawrence, and Anita R. Maguire*

Table of Contents

1. Experimental Procedures 3
2. Synthesis of Dirhodium Carboxylate Catalysts 5
    2.1 Phenylacetic acid synthesis 5
    2.2 *tert*-Butyl ester synthesis 6
    2.3 α-Diazo ester synthesis 7
    2.4 Fenchyloxy acetate synthesis 9
    2.5 Menthyloxy acetate synthesis 11
    2.6 Carboxylic acid synthesis 14
    2.7 Dirhodium carboxylate synthesis 16
3. Synthesis of 2,3-dihydrobenzofurans 21
    3.1 Ester Synthesis 21
    3.2 α-Diazo ester synthesis 22
    3.3 Rhodium Catalysed C–H Insertion Reactions - 2,3-Dihydrobenzofuran Synthesis 23
4. Synthesis of Thiopyran Dioxides 29
    4.1 Preparation of α-Diazo-β-oxosulfone Compounds 29
    4.2 Rhodium Catalysed C–H Insertion Reactions – Thiopyran Dioxide synthesis 32
        4.2.1. Cyclisation of α-diazo-β-oxosulfone 16 34
        4.2.2. Cyclisation of α-diazo-β-oxosulfone 19 36
        4.2.3. Cyclisation of α-diazo-β-oxosulfone 20 38
        4.2.4. Cyclisation of α-diazo-β-oxosulfone 21 40
        4.2.5. Cyclisation of α-diazo-β-oxosulfone 22 42
        4.2.6. Cyclisation of α-diazo-β-oxosulfone 23 43
        4.2.7. Cyclisation of α-diazo-β-oxosulfone 24 45
5. Synthesis of α-diazoacetamides 47
6. Aromatic Addition 52
7. Synthesis of 2-allyl-2-methoxycarbonyl-2,3-dihydrobenzofuran-3-one 61
    7.1 Synthesis of α-diazo-β-keto ester 61
    7.2 Dihydrobenzofuranone synthesis 61
8. NMR Spectra 62
9. HPLC Chromatograms 174
10. Crystal Structures and Data 208
10. Author Contributions 218
11. References 218

1. Experimental Procedures

All solvents utilised in this work were distilled prior to use by the following methods: tetrahydrofuran (THF) was distilled from sodium benzophenone ketyl; dichloromethane (DCM) was distilled from phosphorus pentoxide and, when used for rhodium catalysed C–H insertion reactions, the calcium hydride distilled DCM was deoxygenated using the freeze/thaw/pump method or was stored over 4 Å molecular sieves and deoxygenated by bubbling a stream of nitrogen through it; ethyl acetate was distilled from potassium carbonate; and hexane was distilled prior to use. All commercial reagents were used without further purification unless otherwise stated.

^{1}H (300 MHz) and ^{13}C (75.5 MHz) NMR spectra were recorded on a 300 MHz NMR spectrometer. ^{1}H (400 MHz) and ^{13}C (100.6 MHz) NMR spectra were recorded on a 400 MHz NMR spectrometer. ^{1}H (500 MHz) and ^{13}C (125.8 MHz) NMR spectra were recorded on a 500 MHz NMR spectrometer. ^{1}H (600 MHz) and ^{13}C (150.9 MHz) NMR spectra were recorded on a 600 MHz NMR spectrometer. All spectra were recorded at 300 K in deuterated chloroform ($CDCl_3$) unless otherwise stated, using tetramethylsilane (TMS) as an internal standard. Chemical shifts (δ_H and δ_C) are reported in parts per million (ppm) relative to TMS, and coupling constants (*J*) are expressed in Hertz (Hz). Splitting patterns in ^{1}H NMR spectra are designated as s (singlet), br (broad), bs (broad singlet), d (doublet), t (triplet), q (quartet), qu (quintet), dd (doublet of doublets), dt (doublet of triplets), dq (doublet of quartets), ddd (doublet of doublet of doublets), dddd (doublet of doublet of doublet of doublets), dt (doublet of triplets), ddt (doublet of doublet of triplets), td (triplet of doublets), tt (triplet of triplets), qd (quartet of doublets), and m (multiplet). ^{13}C NMR spectra were calibrated using the solvent signal, *i.e.* $CDCl_3$: δ_C 77.0 ppm. ^{19}F NMR spectra chemical shifts (δ_F) are reported relative to hexafluorobenzene (C_6F_6), which shows a single resonance at –163 ppm. For previously synthesised compounds, spectroscopic details were in agreement with reported values unless otherwise stated.

Infrared spectra were measured using a FTIR UATR2 spectrometer for characterisation of pure compounds. IR monitoring of reactions was conducted using a FTIR UATR2 spectrometer or by evaporation of a solution on sodium chloride plates and recording on a PerkinElmer Paragon 1000 FT-IR spectrometer.

All Celite® and activated charcoal filtrations were carried out in a sintered glass funnel using a tea spoon of both materials. Flash chromatography was carried out either manually or using automated chromatography. Automated chromatography was carried out using a Varian (971-FP) which is equipped with automated fraction collector and UV detector. In all cases, Kieselgel silica gel 60, 0.035–0.075 mm (Merck) was used. Thin layer chromatography (TLC) was carried out on pre-coated silica gel plates (Merck 60 PF254). Visualization was achieved by UV (254 nm) light absorption, and potassium permanganate staining.

The enantiopurity of chiral compounds was measured using chiral stationary phase high performance liquid chromatography (HPLC), carried out on a Lux® 3μm Amylose-1 purchased from Phenomenex, or a Chiralcel® OJ–H, Chiralcel® purchased from Daicel Chemical Industries Limited. Details of the column conditions and mobile phase employed are included in **Table SI.6**. HPLC analysis was performed on a Waters Alliance 2695 separations

module with a Waters Alliance 2996 Photodiode Array detector. Optical rotations were measured on an Autopol V Plus Automatic Polarimeter at 589 nm in a 10 cm cell; concentrations (*c*) are expressed in g/100 mL. $[\alpha]_D^T$ is the specific rotation of a compound and is expressed in units of 10^{-1} deg cm^2 g^{-1}.

The Microanalysis Laboratory, National University of Ireland, Cork, performed elemental analysis using an Exeter Analytical CE440 elemental analyser. Low resolution mass spectra (LRMS) was recorded on a Waters Quattro Micro triple quadrupole instrument in electrospray ionization (ESI) mode using 50% acetonitrile–water containing 0.1% formic acid as eluent. High resolution (precise) mass spectra (HRMS) was recorded on a Waters LCT Premier Tof LC-MS instrument in electrospray ionization (ESI) mode using 50% acetonitrile–water containing 0.1% formic acid as eluent. High resolution (precise) mass spectra (HRMS) were recorded on an Agilent 6530B Accurate Mass Q-TOF LC/MS instrument in electrospray ionization (ESI) mode using 50% acetonitrile–water containing 0.1% formic acid as eluent. High resolution (precise) mass spectra (HRMS) was also recorded on a Waters Vion IMS instrument (SAA055K) with Waters Acquity I-class UPLC in electrospray ionization (ESI) mode using 50% acetonitrile–water containing 0.1% formic acid as eluent and Leucine Enkephalin as reference solution. Samples prepared for either LRMS or HRMS by employing acetonitrile as solvent.

Melting points were obtained using a unimelt Thomas–Hoover capillary melting point apparatus and are uncorrected.

Single crystal X-ray analysis was conducted on either a Bruker APEX II DUO diffractometer (for **7b-d**, **7f**, **7h**, **26a** and **47**) or a Bruke B8 Quest diffractometer (for **30a**) using either monochromatic Mo Kα (λ = 0.7107 Å) or Cu Kα (λ = 1.5418 Å) radiation. All calculation and refinement were made using APEX software.[1] Analysis was undertaken with SHELX suite of programs and diagrams prepared with Mercury 3.0.18.[2]

2. Synthesis of Dirhodium Carboxylate Catalysts

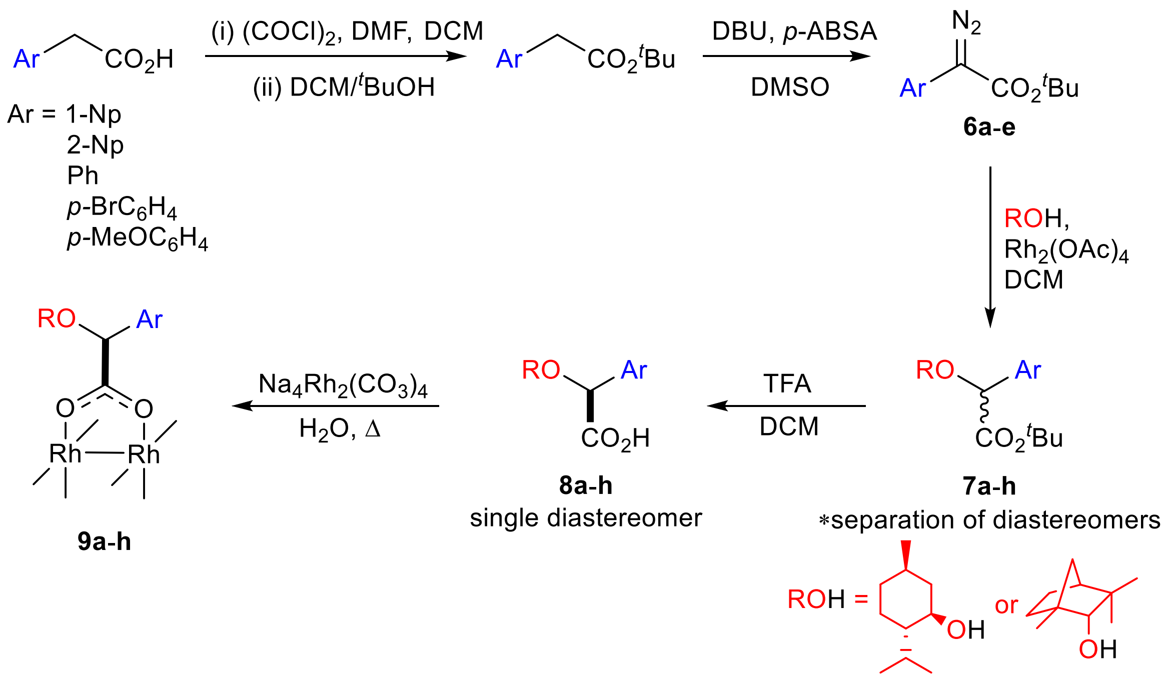

Scheme SI.1: An overview of the dirhodium carboxylate catalyst synthesis

2.1 Phenylacetic acid synthesis

2-Phenylacetic acid[3] (S1)

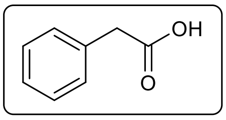

A solution of methyl 2-phenylacetate (10.00 g, 64 mmol) in methanol (60 mL) was treated with a solution of sodium hydroxide (5.1 g, 127 mmol) in water (40 mL) and heated to 70°C for 3 h. The resulting mixture was cooled, then concentrated under reduced pressure to remove the methanol. The residue was diluted with water (40 mL) and washed with diethyl ether (40 mL). The separated aqueous layer was acidified to pH 2 with 2M HCl and extracted with DCM (3 × 80 mL). The combined organic extracts were washed with brine (100 mL), dried over $MgSO_4$ and concentrated to give 2-phenylacetic acid **S1** (8.50 g, 94%) as a white solid which was used without further purification. Spectroscopic characteristics were consistent with previously reported data.[3] **m.p.** 75–77 °C (Lit. 75–77 °C);[3] **^{1}H NMR (300 MHz $CDCl_3$):** δ= 3.64 (2H, s), 7.25–7.37 (5H, m), 10.13 (1H, bs); **IR (neat):** 3200–2500 (COOH), 1690 (CO), 1407, 1228, 1186, 698.

2.2 *tert*-Butyl ester synthesis

tert-Butyl 2-phenylacetate[4] (S2)

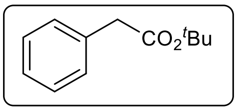

A solution of oxalyl chloride (6.0 mL, 71 mmol) and 2-phenylacetic acid **S1** (8.43 g, 62 mmol) in DCM (50 mL) was treated with catalytic DMF (1 mL) and stirred for 2 h at room temperature. The reaction mixture was concentrated under reduced pressure, the residue was diluted with DCM (30 mL) and *tert*-butanol (*t*-BuOH) (30 mL) and stirred at room temperature overnight. The reaction mixture was concentrated under reduced pressure and following purification by column chromatography on silica gel employing hexane/ethyl acetate (95:5) as the eluent, *tert*-butyl 2-phenylacetate **S2** (8.85g, 74%) was isolated as a clear oil. Spectroscopic characteristics were consistent with previously reported data.[4] **^{1}H NMR (300 MHz, $CDCl_3$):** δ= 1.43 (9H, s), 3.52 (2H, s), 7.20–7.35 (5H, m); **IR (neat):** 2979, 1729 (CO), 1367, 1134, 695.

tert-Butyl 2-(naphthalen-2-yl)acetate[5] (S3)

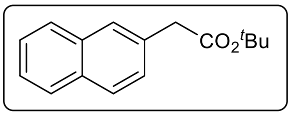

Oxalyl chloride (4.1 mL, 49 mmol), 2-naphthaleneacetic acid (8.00 g, 43 mmol), DCM (50 mL), DMF (1 mL) and *t*-BuOH/DCM (30mL/30mL) were used following the procedure described for **S2** to give, following column chromatography on silica gel employing hexane/ethyl acetate (93:7) as the eluent, *tert*-butyl 2-(naphthalen-2-yl)acetate **S3** (8.81, 85%) as a yellow solid. Spectroscopic characteristics were consistent with previously reported data.[5] **m.p.** 44–45 °C (Lit., 45–46 °C);[6] **^{1}H NMR (400 MHz, $CDCl_3$):** δ= 1.44 (9H, s), 3.68 (2H, s), 7.39–7.49 (3H, m), 7.71 (1H, s), 7.76–7.84 (3H, m); **IR (neat):** 2977, 1713 (CO), 1268, 1131, 800, 746.

tert-Butyl 2-(naphthalen-1-yl)acetate[7] (S4)

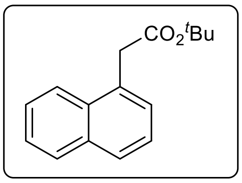

Oxalyl chloride (5.2 mL, 62 mmol), 1-naphthaleneacetic acid (10.00 g, 54 mmol), DCM (75 mL), DMF (1 mL) and *t*-BuOH/DCM (35mL/35mL) were used following the procedure described for **S2** to give, following column chromatography on silica gel employing hexane/ethyl acetate (93:7) as the eluent, *tert*-butyl 1-naphthaleneacetate **S4** (10.02, 78%) as pale-yellow oil. Spectroscopic characteristics were consistent with previously reported data.[7] **^{1}H NMR (400 MHz, $CDCl_3$):** δ= 1.41 (9H, s), 3.97 (2H, s), 7.35–7.44 (2H, m), 7.44–7.56 (2H, m), 7.76 (1H, d, *J* 7.7), 7.84 (1H, d, *J* 7.5), 7.99 (1H, d, *J* 8.3); **IR (neat):** 2977, 1726 (CO), 1367, 1134, 780.

tert-Butyl 2-(4-bromophenyl)acetate[8] (S5)

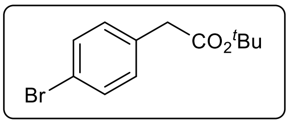

Oxalyl chloride (5.4 mL, 64 mmol), 4-bromophenylacetic acid (12.0 g, 27 mmol), DCM (100 mL), DMF (2 mL) and *t*-BuOH/DCM (40mL/40mL) were used following the procedure described for **S2** to give, following column chromatography on silica gel employing hexane/ethyl acetate (95:5) as the eluent, *tert*-butyl 2-(4-bromophenyl)acetate **S5** (12.57, 83%) as pale yellow oil. Spectroscopic characteristics were consistent with previously reported data.[8] **^{1}H NMR (400 MHz, $CDCl_3$):** δ= 1.43 (9H, s), 3.47 (2H, s), 7.14 (2H, d, *J* 8.3), 7.44 (2H, d, *J* 8.4); **IR (neat):** 2978, 1730 (CO), 1488, 1139, 1012, 803.

tert-Butyl 2-(4-methoxyphenyl)acetate[9] (S6)

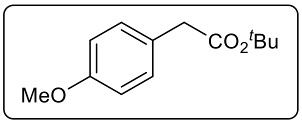

Oxalyl chloride (8.8 mL, 104 mmol), 4-methoxyphenylacetic acid (15 g, 90 mmol), DCM (125 mL), DMF (2 mL) and *t*-BuOH/DCM (50mL/50mL) were used following the procedure described for **S2** to give, following column chromatography on silica gel employing hexane/ethyl acetate (95:5) as the eluent, *tert*-butyl 2-(4-methoxyphenyl)acetate **S6** (15.0 g, 75%) as pale yellow oil. Spectroscopic characteristics were consistent with previously reported data.[9] **^{1}H NMR (300 MHz, $CDCl_3$):** δ= 1.43 (9H, s), 3.45 (2H, s), 3.78 (3H, s), 6.84 (2H, d, *J* 8.4), 7.18 (2H, d, *J* 8.6); **IR (neat):** 2978, 1726 (CO), 1512, 1277, 1135, 820.

2.3 α-Diazo ester synthesis

tert-Butyl 2-diazo-2-phenylacetate[10] (6a)

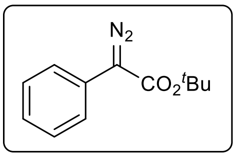

1,8-Diazabicycloundec-7-ene (DBU) (19.9 mL, 113 mmol) was added dropwise over 5 minutes to a solution of 4-acetamidobenzenesulfonyl azide (*p*-ABSA) (13.01 g, 54 mmol) and *tert*-butyl 2-phenylacetate **S2** (8.68 g, 45 mmol) in DMSO (60 mL) at room temperature. After addition was complete, the solution was stirred at room temperature overnight. The reaction mixture was diluted with diethyl ether (60 mL) and water (60 mL) and the layers separated. The aqueous layer was extracted with diethyl ether (3 × 50 mL) and combined organic extracts were washed with water (50 mL), brine (50 mL), dried over $MgSO_4$ and concentrated under reduced pressure. The crude α-diazo ester was purified by column chromatography on silica gel employing hexane/ethyl acetate (98:2) as the eluent, to give α-diazo ester **6a** (8.61 g, 88%) as a dark orange oil. Spectroscopic characteristics were consistent with previously reported data.[10] **^{1}H NMR (300 MHz, $CDCl_3$):** δ= 1.55 (9H, s), 7.12–7.19 (1H, m), 7.32–7.41 (2H, m), 7.43–7.49 (2H, m); **IR (neat):** 2978, 2078 (CN_2), 1695 (CO), 1139, 754.

tert-Butyl 2-diazo-2-(naphthalen-2-yl)acetate[11] (6b)

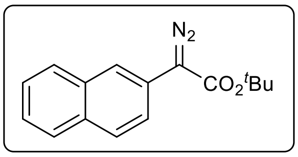

p-ABSA (6.69g, 27 mmol), *tert*-butyl 2-naphthaleneacetate **S3** (4.5 g, 17 mmol), (DBU) (6.9 mL, 46 mmol) and DMSO (40 mL) were used following the procedure described for **6a** to give, following column chromatography on silica gel employing hexane/ethyl acetate (97:3) as the eluent, α-diazo ester **6b** (4.37 g, 88%) as an orange solid. Spectroscopic characteristics were consistent with previously reported data.[11] **m.p.** 84–85 °C; **^{1}H NMR (400 MHz, $CDCl_3$):** δ= 1.58 (9H, s), 7.38–7.55 (3H, m), 7.74–7.87 (3H, m), 8.02 (1H, s); **IR (neat):** 2977, 2080 (CN_2), 1689 (CO), 1144, 1125, 733.

tert-Butyl 2-diazo-2-(naphthalen-1-yl)acetate (6c)

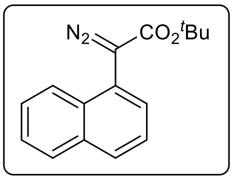

p-ABSA (6.20g, 26 mmol), *tert*-butyl 2-naphthaleneacetate **S4** (5.00 g, 21 mmol), DBU (7.7 mL, 52 mmol) and DMSO (50 mL) were used following the procedure described for **6a** to give, following column chromatography on silica gel employing hexane/ethyl acetate (97:3) as the eluent, α-diazo ester **6c** (1.35 g, 64%) as an orange oil. **^{1}H NMR (400 MHz, $CDCl_3$):** δ= 1.53 (9H, s), 7.46–7.65 (4H, m), 7.81–7.90 (3H, m); **^{13}C NMR (75.5 MHz, $CDCl_3$):** δ= 28.4, 82.0, 122.6, 124.5, 125.6, 126.1, 126.7, 128.8, 129.37, 129.41, 131.5, 134.1, 165.6; **IR (neat):** 2977, 2078 (CN_2), 1694 (CO), 1148, 1100, 771; **HRMS (ESI-TOF):** *m/z* $[M+H]^+$ for $C_{16}H_{17}N_2O_2$, 269.1286, found 269.1290.

tert-Butyl 2-(4-bromophenyl)-2-diazoacetate[11] (6d)

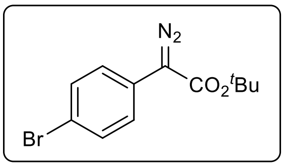

p-ABSA (13.17 g, 55 mmol), *tert*-butyl 2-(4-bromophenyl)acetate **S5** (12.39 g, 46 mmol), DBU (16.9 mL, 114 mmol) and DMSO (100 mL) were used following the procedure described for **6a** to give, following column chromatography on silica gel employing hexane/ethyl acetate (97:3) as the eluent, α-diazo ester **6d** (12.46 g, 92%) as an orange solid. Spectroscopic characteristics were consistent with previously reported data.[11] **m.p.** 71–73 °C; **^{1}H NMR (400 MHz, $CDCl_3$):** δ= 1.54 (9H, s), 7.35 (2H, d, *J* 8.7), 7.47 (2H, d, *J* 8.7); **IR (neat):** 2976, 2082 (CN_2), 1689 (CO), 1489, 1147, 1001, 809.

tert-Butyl 2-(4-methoxyphenyl)-2-diazoacetate[12] (6e)

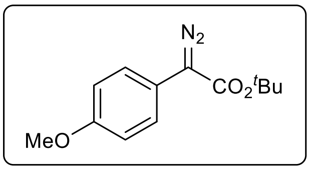

p-ABSA (19.45 g, 81 mmol), *tert*-butyl 2-(4-methoxyphenyl)acetate **S6** (15.00 g, 68 mmol), DBU (25.2 mL, 169 mmol) and DMSO (120 mL) were used following the procedure described for **6a**. Upon work up, ~1/3 of the original ester starting material was still present in the reaction product. The reaction product was re-dissolved in 120 mL DMSO with a further 0.66 equiv. of *p*-ABSA and 1 equv. DBU and stirred at room temperature for a further 24h. Upon work-up, ~10% starting material remained and an additional 0.1 equv ABSA and 0.15 DBU were added and the reaction mixture stirred for 24h at room temperature. Column chromatography on silica gel employing hexane/ethyl acetate (95:5) as the eluent, gave α-diazo ester **6e** (9.57 g, 57%) as an orange oil. **^{1}H NMR (400 MHz, $CDCl_3$):** δ= 1.54 (9H, s), 3.80 (3H, s), 6.93 (2H, d, *J* 8.9), 7.37 (2H, d, *J* 8.9); **^{13}C NMR (75.5 MHz, $CDCl_3$)**: δ= 28.4, 55.4, 81.9, 114.5, 117.6, 125.9, 157.8, 165.1; **IR (neat):** 2934, 2073 (CN_2), 1693 (CO), 1511, 1243, 1139, 1000, 825; **HRMS (ESI-TOF):** *m/z* $[M+H]^+$ for $C_{13}H_{17}N_2O_3$, 249.1239, found 249.1235.

2.4 Fenchyloxy acetate synthesis

tert-Butyl (2*S*)-2-(1″*R*,2″*R*,4″*S*)-fenchyloxy-2-phenylacetate (7a)

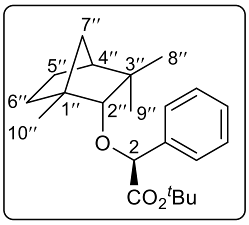

Rhodium(II) acetate (7.4 mg, 0.017 mmol) was added in one portion to a stirring solution of (1*R*)-*endo*-(+)-fenchyl alcohol (2.58 g, 17 mmol) and α-diazo ester **6a** (4.00 g, 18 mmol) in DCM (70 mL). After the evolution of gas subsided, the solution was stirred for 2 h at room temperature and the reaction mixture was concentrated under reduced pressure to give compound **7a** as an 83:17 [(2*S*)/(2*R*)] mixture of diastereomers. Purification of the crude product by column chromatography on silica gel employing hexane/ethyl acetate (98:2) as the eluent allowed partial fractionation of diastereomers but not complete separation (combined yield, 3.37 g, 70%). Sequential recrystallisations from acetonitrile gave (2*S*)-**7a** (0.56 g, 12%), a white crystalline solid, as a single isomer. The other isomer was not isolated in pure form. (2*S*)-**7a**, less polar isomer; **m.p.** 57–59 °C; **Spec. Rot.:** $[\alpha]_D^{20}$+54.9 (*c* 1.0, $CHCl_3$); **^{1}H NMR (300 MHz, $CDCl_3$):** δ= 0.89–1.10 (11H, m, contains 2H, m and 3 × 3H, s at 1.00, 1.04, 1.06), 1.29–1.49 (11H, m, contains 9H, s at 1.39 and 2H, m), 1.61–1.66 (1H, m), 1.67–1.79, (1H, m), 1.80–1.93 (1H, m), 2.99 (1H, d, *J* 1.7), 4.76 (1H, s), 7.27–7.37 (3H, m), 7.41–7.49 (2H, m); **^{13}C NMR (75.5 MHz, $CDCl_3$):** δ= 20.2, 20.9, 26.0, 26.2, 28.0, 31.2, 39.9, 41.4, 48.6, 49.4, 81.3, 81.8, 91.2, 127.2, 128.0, 128.1, 137.9, 170.7; **IR (neat):** 2937, 1742 (CO), 1106, 700; **HRMS (ESI-TOF):** *m*/*z* $[M+H]^+$ calcd for $C_{22}H_{33}O_3$, 345.2430, found 345.2436; **Elemental Analysis:** calcd (%) for $C_{22}H_{32}O_3$: C, 76.70; H, 9.36. Found: C, 76.76; H, 9.23.

A characteristic signal of the (2*R*)-**S7** isomer is the 1H d of C(2″)*H* observed at 3.09 ppm, *J* 1.7, in the ^{1}H NMR spectrum of the crude product. The ratio of diastereomers was calculated based on this signal which occurs at 2.99 ppm for the (2*S*)-**7a** isomer and 3.09 ppm for the (2*R*)-**S7** isomer.

tert-Butyl (2*S*)-2-(1″*R*,2″*R*,4″*S*)-fenchyloxy-2-(naphthalen-2′-yl)acetate (7b)

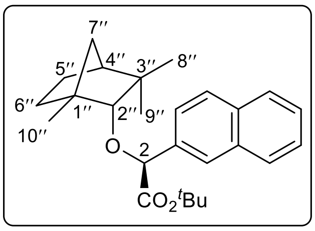

Rhodium(II) acetate (5.8 mg, 0.013 mmol), (1*R*)-*endo*-(+)-fenchyl alcohol (2.04 g, 13 mmol), α-diazo ester **6b** (3.90 g, 15 mmol) and DCM (70 mL) were used following the procedure described for **7a** to give compound **7b** as an 83:17 [(2*S*)/(2*R*)] mixture of diastereomers. Purification of the crude product by column chromatography on silica gel employing hexane/ethyl acetate (98:2) as the eluent allowed partial fractionation of diastereomers but not complete separation (combined yield, 4.27 g, 82%). Sequential recrystallisations from acetonitrile gave (2*S*)-**7b** (1.62 g, 31%), a white crystalline solid, as a single isomer. The other isomer was not isolated in pure form. (2*S*)-**7b**, less polar isomer; **m.p.** 113–114 °C; **Spec. Rot.:** $[\alpha]_D^{20}$ = +80.2 (*c* 1.0, $CHCl_3$); **^{1}H NMR (400 MHz, $CDCl_3$):** δ= 0.92–1.15 (11H, m, contains 2H, m and 3 × 3H, s at 1.00, 1.07, 1.10), 1.33–1.45 (11H, m, contains 9H, s at 1.38 and 2H, m), 1.61–1.68 (1H, m), 1.70–1.80 (1H, m), 1.85–1.95 (1H, m), 3.04 (1H, d, *J* 1.7), 4.90 (1H, s), 7.42–7.51 (2H, m), 7.56–7.65 (1H, m), 7.78–7.87 (3H, m,), 7.89 (1H, s); **^{13}C NMR (100.6 MHz, $CDCl_3$):** δ= 20.2, 20.9, 26.0, 26.2, 28.0, 31.3, 39.8, 41.4, 48.7, 49.3, 81.4, 81.8, 91.1, 125.1, 126.0, 126.7, 127.7, 127.9, 128.1, 133.1, 133.3, 135.3, 170.6; **IR (neat):** 2928, 1714 (CO), 1290, 1128, 477; **HRMS (ESI-TOF):** *m*/*z* $[M+H]^+$ calcd for $C_{26}H_{35}O_3$, 395.2567, found 395.2586; **Elemental Analysis:** calcd (%) for $C_{26}H_{34}O_3$: C, 79.15; H, 8.69. Found: C, 79.03; H, 8.57.

A characteristic signal of the (2*R*)-**S8** isomer is the 1H d of C(2″)*H* observed at 3.14 ppm, *J* 1.7, in the ^{1}H NMR spectrum of the crude product. The ratio of diastereomers was calculated based on this signal which occurs at 3.04 ppm for the (2*S*)-**7b** isomer and 3.14 ppm for the (2*R*)-**S8** isomer.

tert-Butyl (2*S*)-2-(1″*R*,2″*R*,4″*S*)-fenchyloxy-2-(naphthalen-1′-yl)acetate (7c)

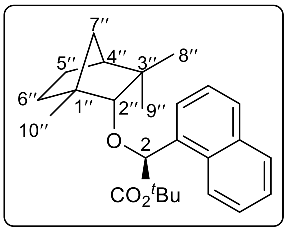

Rhodium(II) acetate (4.9 mg, 0.010 mmol), (1*R*)-*endo*-(+)-fenchyl alcohol (1.57 g, 10 mmol), α-diazo ester **6c** (3.00 g, 11 mmol) and DCM (50 mL) were used following the procedure described for **7a** to give the compound **7c** as a 47:53 [(2*S*)/(2*R*)] mixture of diastereomers. Purification of the crude product by column chromatography on silica gel employing hexane/ethyl acetate (98:2) as the eluent allowed partial fractionation of diastereomers but not complete separation (combined yield, 2.84 g, 72%). Sequential recrystallisations from acetonitrile gave (2*S*)-**7c** (1.13 g, 29%), a white crystalline solid, as a single isomer. The other isomer was not isolated in pure form. (2*S*)-**7c**, less polar isomer; **m.p.** 69–71 °C; **Spec. Rot.:** $[\alpha]_D^{20}$ = −2.0 (*c* 1.0, $CHCl_3$); **^{1}H NMR (400 MHz, $CDCl_3$):** δ= 0.80–0.96 (4H, m, contains 1H, m and 3H, s at 0.84), 1.00 (1H, d, *J* 10.1), 1.09, 1.14 (2 × 3H, s), 1.26–1.43 (11H, m, contains 9H, s at 1.30 and 2H, m), 1.63 (1H, apparent d, *J* 3.0), 1.69–1.79 (1H, m), 1.81–1.92 (1H, m), 3.07 (1H, d, *J* 1.4), 5.34 (1H, s), 7.42–7.55 (3H, m), 7.67 (1H, d, *J* 7.0), 7.78–7.88 (2H, m), 8.30 (1H, d, *J* 8.2) **^{13}C NMR (100.6 MHz, $CDCl_3$):** δ= 20.1, 21.1, 26.0, 26.2, 27.9, 31.4, 39.9, 41.4, 48.7, 49.3, 80.2, 81.5, 91.6, 124.8, 125.2, 125.5, 125.9, 126.8, 128.5, 128.7, 131.1, 133.8, 134.0, 170.6 ; **IR (neat):** 2952, 1743 (CO), 1368, 1151, 789; **Elemental Analysis:** calcd (%) for $C_{26}H_{34}O_3$: C, 79.15; H, 8.69. Found: C, 79.34; H, 8.63.

A characteristic signal of the (2*R*)-**S9** isomer is the 1H d of C(2″)*H* observed at 3.26 ppm, *J* 1.4, in the ^{1}H NMR spectrum of the crude product. The ratio of diastereomers was calculated based on this signal which occurs at 3.07 ppm for the (2*S*)-**7c** isomer and 3.26 ppm for the (2*R*)-**S9** isomer.

tert-Butyl (2*S*)-2-(4′-bromophenyl)-2-(1″*R*,2″*R*,4″*S*)-fenchyloxyacetate (7d)

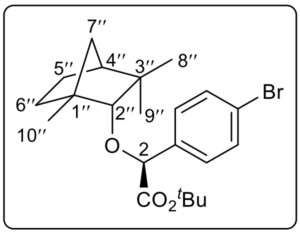

Rhodium(II) acetate (8.5 mg, 0.019 mmol), (1*R*)-*endo*-(+)-fenchyl alcohol (2.95 g, 19 mmol), α-diazo ester **6d** (6.00 g, 21 mmol) and DCM (70 mL) were used following the procedure described for **7a** to give compound **7d** as a 79:21 [(2*S*)/(2*R*)] mixture of diastereomers. Purification of the crude product by column chromatography on silica gel employing hexane/ethyl acetate (98:2) as the eluent allowed partial fractionation of diastereomers but not complete separation (combined yield, 6.35 g, 79%). Sequential recrystallisations from acetonitrile gave (2*S*)-**7d** (3.26 g, 37%), a white crystalline solid, as a single isomer. The other isomer was not isolated in pure form. (2*S*)-**7d**, less polar isomer; **m.p.** 100–102 °C; **Spec. Rot.:** $[\alpha]_D^{20}$ = +48.3 (*c* 1.0, $CHCl_3$); **^{1}H NMR (400 MHz, $CDCl_3$):** δ= 0.90–1.10 (11H, m, contains 2H, m and 3 × 3H, 2 × s, one at 1.00 and two at 1.04), 1.33–1.46 (11H, m, contains 9H, s at 1.38 and 2H, m), 1.60–1.66 (1H, m), 1.67-1.76 (1H, m), 1.77–1.87 (1H, m), 2.97 (1H, bs, *J* 1.5), 4.71 (1H, s), 7.33 (2H, d, *J* 8.4), 7.46 (2H, d, *J* 8.4); **^{13}C NMR (100.6 MHz,**

$CDCl_3$): δ= 20.3, 20.8, 26.0, 26.2, 27.9, 31.2, 39.8, 41.4, 48.6, 49.3, 81.2, 81.6, 91.5, 122.1, 128.9, 131.3, 136.9, 170.2; **IR (neat):** 2943, 1744 (CO), 1116, 1097, 785; **Elemental Analysis:** calcd (%) for $C_{22}H_{31}O_3Br$: C, 62.41; H, 7.38. Found: 62.49; H, 7.35.

A characteristic signal of the (2*R*)-**S10** isomer is the 1H bs of C(2″)*H* observed at 3.07 ppm in the ^{1}H NMR spectrum of the crude product. The ratio of diastereoisomers was calculated based on the signal which occurs at 2.97 ppm for the (2*S*)-**7d** isomer and 3.07 ppm for the (2*R*)-**S10** isomer.

***tert*-Butyl (2*S*)-2-(1″*R*,2″*R*,4″*S*)-fenchyloxy-2-(4′-methoxyphenyl)acetate (7e)**

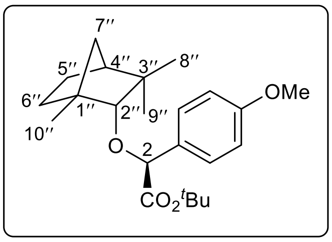

Rhodium(II) acetate (7.6 mg, 0.017 mmol), (1*R*)-*endo*-(+)-fenchyl alcohol (2.66 g, 17 mmol), α-diazo ester **6e** (4.50 g, 18 mmol) and DCM (50 mL) were used following the procedure described for **7a** to give compound **7e** as a 83:17 [(2*S*)/(2*R*)] mixture of diastereomers. Purification of the crude product by column chromatography on silica gel employing hexane/ethyl acetate (95:5) as the eluent allowed partial fractionation of diastereomers but not complete separation (combined yield, 5.06 g, 80%). Sequential recrystallisations from acetonitrile gave (2*S*)-**7e** (2.32 g, 36%), a white crystalline solid, as a single isomer. The other isomer was not isolated in pure form. (2*S*)-**7e**, less polar isomer; **m.p.** 66–68 °C; **Spec. Rot.:** $[\alpha]_D^{20}$ = +60.4 (*c* 1.0, $CHCl_3$); **^{1}H NMR (400 MHz, $CDCl_3$):** δ= 0.87–1.07 (11H, m, contains 2H, m and 3 × 3H, s at 0.99, 1.03 and 1.04), 1.31–1.46 (11H, m, contains 9H, s at 1.39 and 2H, m), 1.59–1.64 (1H, m), 1.66-1.77 (1H, m), 1.79–1.91 (1H, m), 2.96 (1H, d, *J* 1.4), 3.80 (3H, s), 4.70 (1H, s), 7.86 (2H, d, *J* 8.7), 7.36 (2H, d, *J* 8.7); **^{13}C NMR (100.6 MHz, $CDCl_3$):** δ= 20.2, 20.8, 26.0, 26.2, 28.0, 31.2, 39.8, 41.4, 48.6, 49.3, 55.2, 81.1, 81.2, 90.8, 113.5, 128.6, 130.0, 159.4, 170.9; **IR (neat):** 2946, 1714 (CO), 1239, 1095, 847; **Elemental Analysis:** calcd (%) for $C_{23}H_{34}O_4$: C, 73.76; H, 9.15. Found: C, 73.84; H, 9.00.

A characteristic signal of the (2*R*)-**S11** isomer is the 1H d of C(2″)*H* observed at 3.05 ppm, *J* 1.4, in the ^{1}H NMR spectrum of the crude product. The ratio of diastereomers was calculated based on this signal which occurs at 2.96 ppm for the (2*S*)-**7e** isomer and 3.05 ppm for the (2*R*)-**S11** isomer.

2.5 Menthyloxy acetate synthesis

***tert*-Butyl (2*S*)-2-(1″*R*,2″*S*,5″*R*)-menthyloxy-2-phenylacetate (7f)**

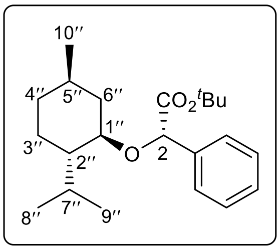

Rhodium(II) acetate (10.6 mg, 0.026 mmol), (−)-menthol (3.76 g, 24 mmol) and α-diazo ester **6a** (5.75 g, 26 mmol) and DCM (80 mL) were used following the procedure for **7a** to give compound **7f** as a 77:23 [(2*S*)/(2*R*)] mixture of diastereomers. Purification of the mixture by column chromatography on silica gel employing hexane/ethyl acetate (98:2) as the eluent allowed partial separation of isomers but not complete separation (combined yield, 6.93 g, 83%). Sequential recrystallisations from acetonitrile gave (2*S*)-**7f** (3.35 g, 40%), a white crystalline solid, as a single isomer. (2*S*)-**7f**, less polar isomer; **m.p.** 75–77 °C; **Spec. Rot.:** $[\alpha]_D^{20}$ = −61.6 (*c* 1.0, $CHCl_3$); **^{1}H NMR (300 MHz, $CDCl_3$):** δ= 0.79–1.08 (12H, m, which contains, 3H, m, 3H,

d, *J* 6.9 at 0.85, 3H, d, *J* 6.8 at 0.89 and 3H, d, *J* 7.1 at 0.94), 1.23–1.46 (11H, m, contains 2H, m, and 9H, s at 1.39), 1.58–1.71 (2H, m), 2.01–2.13 (1H, m), 2.44–2.61 (1H, m), 3.31 (1H, td, *J* 10.5, 4.2), 4.94 (1H, s), 7.23–7.37 (3H, m), 7.42–7.50 (2H, m); **^{13}C NMR (75.5 MHz, $CDCl_3$):** δ= 16.4, 21.2, 22.3, 23.2, 25.2, 27.9, 31.5, 34.5, 40.4, 48.4, 78.5, 78.6, 81.4, 126.9, 128.0, 128.3, 138.3, 170.8; **IR (neat):** 2958, 1734 (CO), 1146, 1097, 701; **Elemental Analysis:** calcd (%) for $C_{22}H_{34}O_3$: C, 76.26; H, 9.89. Found: 76.45; H, 9.80.

tert-Butyl (2*R*)-2-(1″*R*,2″*S*,5″*R*)-menthyloxy-2-phenylacetate (S12)

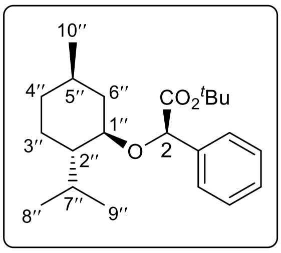

(2*R*)-**S12**, more polar isomer (0.65 g, 8%), **m.p.** 80–81 °C; **Spec. Rot.:** $[\alpha]_D^{20}$ = −137.3 (*c* 1.0, $CHCl_3$); **^{1}H NMR (400 MHz, $CDCl_3$):** δ= 0.49 (3H, d, *J* 6.9), 0.78–0.96 (8H, m, contains 2H, m and 3H, d, *J* 7.0 at 0.85 and 3H, d, *J* 7.0 at 0.92), 1.02 (1H, q, *J* 11.1), 1.23–1.45 (11H, m), 1.55–1.70 (2H, m), 2.07–2.17 (1H, m), 2.22–2.35 (1H, m), 3.09 (1H, td, *J* 10.5, 4.1), 4.83 (1H, s), 7.28–7.37 (3H, m), 7.39–7.47 (2H, m); **^{13}C NMR (100.6 MHz, $CDCl_3$):** δ= 15.6, 21.1, 22.4, 22.9, 25.1, 27.9, 31.6, 34.4, 40.5, 48.1, 78.2, 79.2, 81.3, 127.4, 128.1, 128.3, 137.7, 171.0; **IR (neat):** 2959, 2930, 1735 (CO), 1151, 1049, 697; **Elemental Analysis:** calcd (%) for $C_{22}H_{34}O_3$: C, 76.26; H, 9.89. Found C, 75.95; H, 9.75.

tert-Butyl (2*S*)-(1″*R*,2″*S*,5″*R*)-menthyloxy-2-(naphthalen-2-yl)acetate[13] (7g)

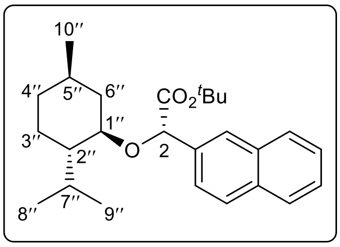

Rhodium(II) acetate (10.5 mg, 0.024 mmol), (−)-menthol (3.71 g, 24 mmol) and α-diazo ester **6b** (7.00 g, 26 mmol) and DCM (60 mL) were used following the procedure for **7a** to give compound **7g** as a 76:24 [(2*S*)/(2*R*)] mixture of diastereomers. Purification of the mixture by column chromatography on silica gel employing hexane/ethyl acetate (98:2) as the eluent allowed partial separation of isomers (combined yield, 7.16 g, 75%). Sequential recrystallisations from acetonitrile gave (2*S*)-**7g** (2.35, 29%), a white crystalline solid, as a single isomer. (2*S*)-**7g**, less polar isomer; **m.p.** 77–79 °C; **Spec. Rot.:** $[\alpha]_D^{20}$ = −33.1 (*c* 1.0, $CHCl_3$); **^{1}H NMR (400 MHz, $CDCl_3$):** δ= 0.78–1.08 (12H, m, contains, 3H, m, 2 × overlapping 3H, d, *J* ~7.0, at 0.87 and 0.88 and 3H, d, *J* 7.1 at 0.96), 1.26–1.46 (11H, m, contains 2H, m and 9H, s at 1.36), 1.60–1.72 (2H, m), 2.08 (1H, bd, *J* 11.6), 2.51–2.65 (1H, m), 3.36 (1H, td, *J* 10.5, 4.1), 5.10 (1H, s), 7.42–7.51 (2H, m), 7.60 (1H, dd, *J* 8.5, 1.4), 7.77–7.89 (3H, m), 7.92 (1H, s); **^{13}C NMR (100.6 MHz, $CDCl_3$):** δ= 16.4, 21.3, 22.4, 23.2, 25.3, 28.0, 31.6, 34.5, 40.5, 48.4, 78.9, 79.0, 81.6, 124.8, 125.99, 126.06, 126.1, 127.7, 128.0, 128.2, 133.21, 133.23, 135.8, 170.8; **IR (neat):** 2953, 1737 (CO), 1147, 1095, 760; **Elemental Analysis:** calcd (%) for $C_{26}H_{36}O_3$: C, 78.75; H, 9.15. Found: 78.75; H, 9.08.

tert-Butyl (2*R*)-(1″*R*,2″*S*,5″*R*)-menthyloxy-2-(naphthalen-2-yl)acetate[13] (S13)

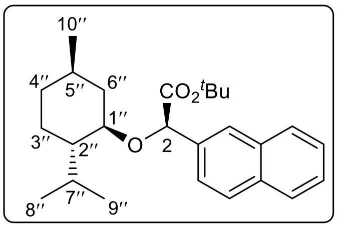

(2*R*)-**S13**, more polar, minor isomer, white solid (0.52, 5%), **m.p.** 92–93 °C; **Spec. Rot.:** $[\alpha]_D^{20}$ = −156.1 (*c* 1.0, $CHCl_3$); **^{1}H NMR (400 MHz, $CDCl_3$):** δ= 0.46 (3H, d, *J* 6.9), 0.75–0.99 [8H, m, contains, 2H, m, 3H, d, *J* 7.1 at 0.86 and 3H, d, *J* 6.5 at 0.93], 1.05 (1H, q, *J* 11.1), 1.21–1.49 (11H, m, contains 2H, m and 9H, s at 1.37), 1.54–1.67 (2H, m), 2.18 (1H, bd, *J* 11.7), 2.29–2.43 (1H, m), 3.13 (1H, td, *J* 10.5, 4.1), 5.02 (1H, s), 7.43–7.53 (2H, m), 7.57 (1H, dd, *J* 8.5, 1.4), 7.78–7.93 (3H, m), 7.89 (1H, s); **^{13}C NMR (100.6 MHz, $CDCl_3$):** δ= 15.7, 21.1, 22.4, 22.9, 25.1, 27.9, 31.6, 34.4, 40.4, 48.1, 78.0, 79.2, 81.5, 125.1, 126.07, 126.10, 126.8, 127.8, 128.13, 128.15, 133.1, 133.3, 135.1, 170.9; **IR (neat):** 2920, 1732 (CO), 1148, 1084, 817; **Elemental Analysis:** calcd (%) for $C_{26}H_{36}O_3$: C, 78.75; H, 9.15. Found: 78.64; H, 9.06.

tert-Butyl (2*S*)-2-(4′-bromophenyl)-2-(1″*R*,2″*S*,5″*R*)-menthyloxyacetate (7h)

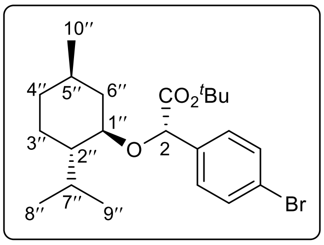

Rhodium(II) acetate (8.8 mg, 0.020 mmol), (−)-menthol (3.19 g, 20 mmol) and α-diazo ester **6d** (6.10 g, 21 mmol) and DCM (60 mL) were used following the procedure for **7a** to give compound **7h** as a 87:13 [(2*S*)/(2*R*)] mixture of diastereomers. Purification of the mixture by column chromatography on silica gel employing hexane/ethyl acetate (98:2) as the eluent allowed partial separation of isomers (combined yield, 6.89 g, 81%). Sequential recrystallisations from acetonitrile gave (2*S*)-**7h** (3.93 g, 44%), a white crystalline solid, as a single isomer. The other isomer was not isolated in pure form. (2*S*)-**7h**, less polar isomer; **m.p.** 100–102 °C; **Spec. Rot.:** $[\alpha]_D^{20}$ = −45.7 (*c* 1.0, $CHCl_3$); **^{1}H NMR (400 MHz, $CDCl_3$):** δ= 0.76–1.07 (12H, m, contains, 3H, m, 3H, d, *J* 6.9 at 0.84, 3H, d, *J* 6.5 at 0.89 and 3H, d, *J* 7.1 at 0.94), 1.26–1.44 (11H, m, contains 2H, m and 9H, s at 1.39), 1.60–1.71 (2H, m), 2.03 (1H, bd, *J* 12.0), 2.42–2.57 (1H, m), 3.30 (1H, td, *J* 10.5, 4.1), 4.89 (1H, s), 7.31–7.37 (2H, m), 7.42–7.49 (2H, m); **^{13}C NMR (100.6 MHz, $CDCl_3$):** δ= 16.3, 21.2, 22.3, 23.1, 25.3, 27.9, 31.5, 34.4, 40.3, 48.4, 78.0, 78.8, 81.7, 122.1, 128.6, 131.4, 137.4, 170.3; **IR (neat):** 2929, 1737 (CO), 1144, 1096, 780; **HRMS (ESI-TOF):** *m/z* $[M+H]^+$ calcd for $C_{22}H_{34}O_3{}^{79}Br$, 425.169, found 425.1684; **Elemental Analysis:** calcd (%) for $C_{22}H_{33}O_3Br$: C, 62.12; H, 7.82. Found: C, 62.19; H, 7.75.

A characteristic signal of the (2*R*)-**S14** isomer is the 1H triplet of doublets of C(1″)*H*, *J* 10.5, 4.1, observed at 3.08 ppm in the ^{1}H NMR spectrum of the crude product. The ratio of diastereomers was calculated based on this signal which occurs at 3.30 ppm for the (2*S*)-**7h** isomer and 3.08 ppm for the (2*R*)-**S14** isomer.

2.6 Carboxylic acid synthesis

(2*S*)-2-(1″*R*,2″*R*,4″*S*)-Fenchyloxy-2-phenylacetic acid (8a)

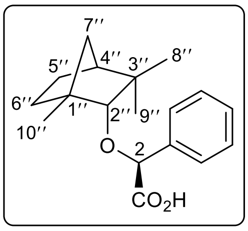

Trifluoroacetic acid (TFA) (1.5 mL) was added to a stirring solution of (2*S*)-**7a** (0.50 g, 1.7 mmol) in DCM (5mL). The solution was stirred for 2 h at room temperature and was then concentrated under reduced pressure to give the crude product. Following purification by column chromatography on silica gel employing hexane/ethyl acetate (99:1) as eluent, the pure acid **8a** (0.44 g, 90%) was isolated as a white solid. **m.p.** 113–115 °C; **Spec. Rot.:** $[\alpha]_D^{20}$ = +137.5 (*c* 0.75, $CHCl_3$); **^{1}H NMR (400 MHz, $CDCl_3$):** δ= 0.91 (3H, s), 0.98–1.11 (8H, m, contains 2H, m and 2 × overlapping 3H, s at 1.04), 1.34–1.51 (2H, m), 1.64–1.68 (1H, m), 1.69–1.80 (2H, m), 3.08 (1H, finely split s, *J* 1.3), 4.88 (1H, s), 7.32–7.44 (5H, m), ~9.30 (1H, bs); **^{13}C NMR (100.6 MHz, $CDCl_3$):** δ= 19.8, 20.9, 26.05, 26.07, 31.4, 39.6, 41.3, 48.7, 49.1, 80.5, 91.0, 127.7, 128.6, 129.0, 135.8, 172.9; **IR (neat):** 3400–2500 (COOH), 2951, 1721 (CO), 1455, 1114, 1099, 697; **Elemental Analysis:** calcd (%) for $C_{18}H_{24}O_3$: C, 74.97; H, 8.39. Found: 74.97; H, 8.38.

(2*S*)-2-(1″*R*,2″*R*,4″*S*)-Fenchyloxy-2-(naphthalen-2′-yl)acetic acid (8b)

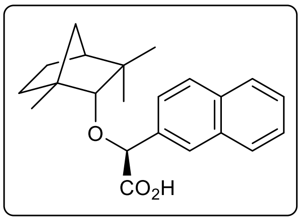

TFA (1 mL), (2*S*)-**7b** (0.15 g, 0.38 mmol) in DCM (5mL) were used following the procedure for **8a** to give, following column chromatography on silica gel employing hexane/ethyl acetate (95:5) as the eluent, the acid **8b** (95 mg, 74%) as a clear, viscous oil. **Spec. Rot.:** $[\alpha]_D^{20}$ = +130.9 (*c* 1.2, $CHCl_3$); **^{1}H NMR (400 MHz, $CDCl_3$):** δ= 0.81–1.13 (11H, m, contains 2H, m, 3 × 3H, s at 0.92, 1.05 and 1.08), 1.31–1.48 (2H, m), 1.61–1.68 (1H, m), 1.69–1.88 (2H, m), 3.10 (1H, bs), 5.04 (1H, s), 7.42–7.58 (3H, m), 7.77–7.90 (4H, m), ~9.21 (1H, bs); **^{13}C NMR (100.6 MHz, $CDCl_3$):** δ= 19.9, 20.9, 26.09, 26.11, 31.5, 39.7, 41.3, 48.7, 49.1, 80.7, 91.0, 124.7, 126.4, 126.6, 127.6, 127.8, 128.1, 128.6, 133.0, 133.4, 133.6, 174.3; **IR (neat):** 3300–2400 (COOH), 2950, 1717 (CO), 1112, 1101, 774, 732; **HRMS (ESI-TOF):** *m*/*z* $[M+H]^+$ calcd for $C_{22}H_{27}O_3$, 339.1960, found 339.1960.

(2*S*)-2-(1″*R*,2″*R*,4″*S*)-Fenchyloxy-2-(naphthalen-1′-yl)acetic acid (8c)

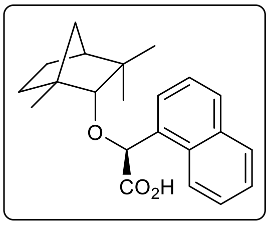

TFA (1 mL), (2*S*)-**7c** (0.15 g, 0.38 mmol) and DCM (2.5mL) were used following the procedure for **8a** to give, following column chromatography on silica gel employing hexane/ethyl acetate (95:5) as the eluent, the acid **8c** (0.12 g, 93%) as a clear, viscous oil. **Spec. Rot.:** $[\alpha]_D^{20}$= 170.2 (*c* 1.5, $CHCl_3$); **^{1}H NMR (400 MHz, $CDCl_3$):** δ= 0.71 (3H, s), 0.77–1.17 (8H, m, contains 2H, m and 2 × 3H, s at 1.06, 1.12), 1.19–1.47 (2H, m), 1.61–1.67 (1H, m), 1.68–1.83 (2H, m), 3.10 (1H, finely split s, *J* 1.2), 5.44 (1H, s), 7.41–7.53 (3H, m), 7.59 (1H, d, *J* 6.7), 7.81–7.88 (2H, m), 8.15 (1H, d, *J* 7.9), ~9.88 (1H, bs); **^{13}C NMR (100.6 MHz, $CDCl_3$):** δ= 19.7, 21.1, 26.0, 26.1, 31.5, 39.8, 41.3, 48.7, 49.1, 79.3, 91.6, 124.2, 125.0, 126.0, 126.6, 127.7, 128.8, 129.7, 131.1, 132.0, 134.0, 174.4; **IR (neat):** 3500–2600 (COOH), 2950, 1717 (CO), 1111, 1101, 774; **Elemental Analysis:** calcd (%) for $C_{22}H_{26}O_3$: C, 78.07; H, 7.56. Found: 77.93; H, 7.56.

(2*S*)- 2-(4′-Bromophenyl)-2-(1″*R*,2″*R*,4″*S*)-fenchyloxyacetic acid (8d)

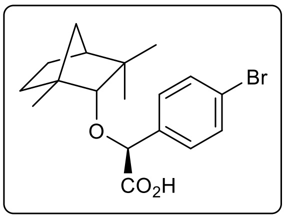

TFA (2 mL), (2*S*)-**7d** (0.25 g, 0.59 mmol) and DCM (5 mL) were used following the procedure for **8a** to give, following column chromatography on silica gel employing hexane/ethyl acetate (95:5) as the eluent, the acid **8d** (0.16 g, 74%) as a white solid. **m.p.** 65–68 °C; **Spec. Rot.:** $[\alpha]_D^{25}$ = +98.1 (*c* 1.0, $CHCl_3$); **^{1}H NMR (400 MHz, $CDCl_3$):** δ= 0.95 (3H, s), 0.98–1.09 (8H, m, contains 2H, m and 2 × overlapping 3H, s at 1.02), 1.35–1.48 (2H, m), 1.62–1.68 (1H, m), 1.68–1.82 (2H, m), 3.02 (1H, finely split s, *J* 1.1), 4.84 (1H, s), 7.31 (2H, d, *J* 8.4), 7.49 (2H, d, *J* 8.4), ~9.20 (1H); **^{13}C NMR (100.6 MHz, $CDCl_3$):** δ= 20.0, 20.9, 26.0, 26.1, 31.3, 39.7, 41.3, 48.6, 49.2, 80.0, 91.5, 123.1, 129.2, 131.8, 135.2, 174.8; **IR (neat):** 3300-2400 (COOH), 2950, 1722 (CO), 1094, 816; **Elemental Analysis:** calcd (%) for $C_{18}H_{23}O_3Br$: C, 58.86; H, 6.31. Found: 58.92; H, 6.27.

(2*S*)-2-(1″*R*,2″*R*,4″*S*)-Fenchyloxy-2-(4′-methoxy)phenylacetic acid (8e)

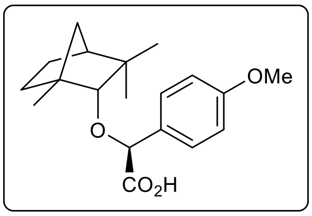

A solution of (2*S*)-**7e** (0.60 g, 1.6 mmol) in methanol (15 mL) was treated with sodium hydroxide (0.64 g, 16 mmol) and heated under reflux for 4 h. The resulting mixture was cooled, then concentrated under reduced pressure to remove the methanol. The residue was dissolved in DCM (30 mL) and washed with 2M HCl (30 mL). The combined organic extracts were washed with brine (50 mL), dried over $MgSO_4$ and concentrated to give compound **8e** (0.47 g, 92%), a white solid, as a 95:5 [(2*S*):(2*R*)] mixture of diastereomers which was used without further purification. **m.p.** 90–93 °C; **Spec. Rot.:** $[\alpha]_D^{25}$ = +133.6 (*c* 1.0, $CHCl_3$); **^{1}H NMR (400 MHz, $CDCl_3$):** δ= 0.91 (3H, s), 0.99–1.12 (8H, m, contains 2H, m and 2 × 3H, s, at 1.028, 1.033), 1.33–1.50 (2H, m), 1.62–1.68 (1H, m), 1.68–1.80 (2H, m), 3.06 (1H, finely split s, *J* 1.2), 3.81 (3H, s), 4.82 (1H, s), 6.89 (2H, d, *J* 8.6), 7.32 (2H, d, *J* 8.6), COO*H* not observed; **^{13}C NMR (100.6 MHz, $CDCl_3$):** δ= 19.8, 20.9, 26.05, 26.08, 31.4, 39.5, 41.3, 48.7, 49.0, 55.3, 80.0, 90.5, 114.0, 127.9, 129.1, 160.1, 173.5; **IR (neat):** 3500–2300 (COOH), 2951, 1721 (CO), 1512, 1033, 829; **Elemental Analysis:** calcd (%) for $C_{19}H_{26}O_4$: C, 71.67; H, 8.23. Found C, 71.40; H, 8.09.

A characteristic signal of the (2*R*)-**S15** isomer is the 1H, finely split singlet of C(2)*H*, *J* 1.2 observed at 3.12 ppm in the ^{1}H NMR spectrum of the crude product. The ratio of diastereomers was calculated based on this signal which occurs at 3.06 ppm for the (2*S*)-**8e** isomer and 3.12 ppm for the (2*R*)-**S15** isomer.

(2*S*)-2-(1″*R*,2″*S*,5″*R*)-Menthyloxy-2-phenylacetic acid (8f)

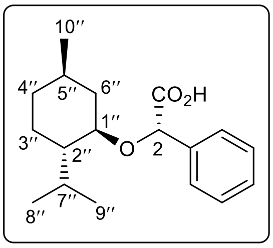

TFA (5 mL), (2*S*)-**7f** (2.00 g, 5.8 mmol) and DCM (5mL) were used following the procedure for **8a** to give compound **8f** (1.61 g, quantitative) as a white solid which was used without further purification. **m.p.** 96–98 °C; **Spec. Rot.:** $[\alpha]_D^{20}$ = −14.05 (*c* 1.0, $CHCl_3$); **^{1}H NMR (300 MHz, $CDCl_3$):** δ= 0.73–1.06 (12H, m, contains, 3H, m, 3H, d, *J* 6.6 at 0.81, 3H, d, *J* 7.0 at 0.85 and 3H, d, *J* 7.0 at 0.94), 1.18–1.45 (2H, m), 1.58–1.73 (2H, m), 1.90 (1H, bd, *J* 12.1), 2.28–2.47 (1H, m), 3.36 (1H, td, *J* 10.5, 4.2), 5.04 (1H, s), 7.29–7.41 (3H, m), 7.42–7.50 (2H, m), 9.01 (1H, bs); **^{13}C NMR (75.5 MHz, $CDCl_3$):** δ= 16.0, 21.2, 22.2, 23.0, 25.5, 31.5,

34.3, 40.7, 48.6, 78.3, 79.5, 127.1, 128.6, 128.7, 137.0, 175.6; **IR (neat):** 3400–3000 (COOH), 2932, 1721 (CO), 1694, 1105, 1095, 696; **Elemental Analysis:** calcd (%) for $C_{18}H_{26}O_3$: C, 74.45; H, 9.02, found: C, 74.55; H, 8.99.

(2*S*)-2-(1″*R*,2″*S*,5″*R*)-Menthyloxy-2-(naphthalen-2′-yl)acetic acid (8g)

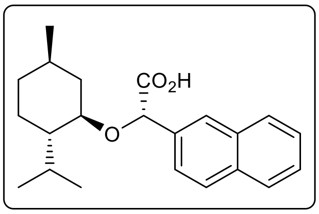

TFA (5 mL), (2*S*)-**7g** (0.90 g, 2.5 mmol) and DCM (5mL) were used following the procedure for **8a** to give compound **8g** (0.71 g, quantitative) as an off white foamy solid which was used without further purification. **m.p.** 112–115 °C; **Spec. Rot.:** $[\alpha]_D^{20}$ = 19.25 (*c* 1.0, $CHCl_3$); **^{1}H NMR (400 MHz, $CDCl_3$):** δ= 0.73–1.05 (12H, m, contains, 3H, m, 3H, d, *J* 6.5 at 0.81, 3H, d, *J* 6.9 at 0.85, and 3H, d, *J* 7.0 at 0.96), 1.18–1.34 (1H, m), 1.34–1.48 (1H, m), 1.57–1.71 (2H, m), 1.91 (1H, bd, *J* 11.9), 2.34–2.48 (1H, m), 3.40 (1H, td, *J* 10.5, 4.1), 5.19 (1H, s), 7.44–7.52 (2H, m), 7.57 (1H, dd, *J* 8.5, 1.4), 7.77–7.87 (3H, m), 7.91 (1H, s), 8.57 (1H, bs); **^{13}C NMR (100.6 MHz, $CDCl_3$):** δ= 16.0, 21.3, 22.2, 22.9, 25.5, 31.5, 34.3, 40.9, 48.6, 78.7, 79.8, 124.5, 126.3, 126.4, 126.6, 127.7, 128.2, 128.5, 133.1, 133.5, 134.4, 175.3; **IR (neat):** 3500–2200 (COOH), 2955, 1707 (CO), 1113, 1085, 747; **Elemental Analysis:** calcd (%) for $C_{22}H_{28}O_3$: C, 77.61; H, 8.29. Found: C, 77.62; H, 8.19.

(2*S*)- 2-(4′-Bromophenyl)-2-(1″*R*,2″*S*,5″*R*)-menthyloxyacetic acid (8h)

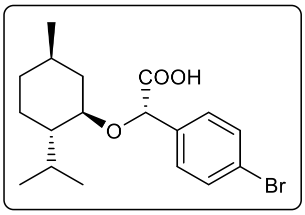

TFA (2.5 mL), (2*S*)-**7h** (0.60 g, 1.4 mmol) and DCM (5mL) were used following the procedure for **8a** to give, following column chromatography on silica gel employing hexane/ethyl acetate (95:5) as the eluent, the acid **8h** (0.36 g, 70%) as a colourless oily solid. **Spec. Rot.:** $[\alpha]_D^{20}$ = −9.1 (*c* 1.7, $CHCl_3$); **^{1}H NMR (400 MHz, $CDCl_3$):** δ= 0.71–1.04 (12H, m, contains, 3H, m, 3H, d, *J* 6.8 at 0.79, 3H, d, *J* 6.6 at 0.86 and 3H, d, *J* 7.0 at 0.93), 1.22–1.40 (2H, m), 1.58–1.71 (2H, m), 1.93 (1H, bd, *J* 11.6), 2.31–2.47 (1H, m), 3.34 (1H, td, *J* 10.5, 4.1), 5.01 (1H, s), 7.34 (2H, d, *J* 8.4), 7.47 (2H, d, *J* 8.3), 11.10 (1H, bs); **^{13}C NMR (100.6 MHz, $CDCl_3$):** δ= 16.0, 21.2, 22.3, 23.0, 25.3, 31.5, 34.3, 40.5, 48.5, 77.4, 79.4, 122.8, 128.8, 131.7, 136.2, 176.5; **IR (neat):** 3500–2200 (COOH), 2921, 1718 (CO), 1487, 1011, 813; **Elemental Analysis:** calcd (%) for $C_{18}H_{25}O_3Br$: 58.54; H, 6.82. Found: 58.21; H, 6.97.

2.7 Dirhodium carboxylate synthesis

Sodium rhodium carbonate [$Na_4Rh_2(CO_3)_4\cdot2.5H_2O$][14] (S16)

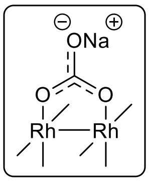

Rhodium acetate dimer (1.50 g, 3.4 mmol) in aqueous sodium carbonate (2M, 25 mL) was heated under reflux for 1 h. The resulting mixture was cooled to room temperature, filtered and washed with water (20 mL), methanol (20 mL) and diethyl ether (20 mL) to give the sodium salt of rhodium carbonate **S16** (1.92 g, 97%) as a blue purple solid.

****Note: Four ligands surround the dirhodium unit throughout with just one shown for clarity.***

Dirhodium tetrakis [(2*S*)-2-(1″*R*,2″*R*,4″*S*)-fenchyloxy-2-phenylacetate] (2*S*-FPA) (9a)

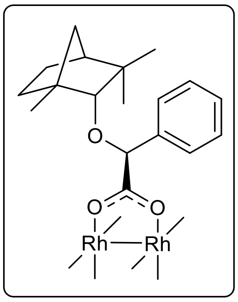

Sodium rhodium carbonate [$Na_4Rh_2(CO_3)_4{\cdot}2.5H_2O$] **S16** (98 mg, 0.17 mmol) and (2*S*)-**8a** (0.39 g, 1.3 mmol) were added to water (20 mL) and refluxed overnight. The solution was cooled, extracted with DCM (3 × 30 mL) and the combined organic extracts were washed with saturated sodium bicarbonate solution (2 × 30 mL), brine (30 mL), dried over $MgSO_4$ and concentrated. Following purification by column chromatography on silica gel employing hexane/ethyl acetate (92:8) as the eluent, the rhodium complex **9a** (70 mg, 31%) was isolated as a green oil. **^{1}H NMR (400 MHz, $CDCl_3$):** δ= 0.79–0.99 (11H, m, contains, 2H, m, 3H, s at 0.81, 3H, s at 0.85 and 3H, s at 0.94), 1.22–1.38 (2H, m), 1.55–1.61 (1H, m), 1.63–1.79 (2H, m), 2.49 (1H, bs), 4.44 (1H, s), 7.11–7.26 (5H, m); **^{13}C NMR (100.6 MHz, $CDCl_3$):** δ= 20.1, 20.9, 26.1, 26.2, 30.9, 39.8, 41.1, 48.5, 49.4, 82.0, 91.5, 126.8, 127.6, 128.0, 138.3, 191.7; **IR (neat):** 2947, 2869, 1689 (weak), 1602 (strong), 1400, 1129, 733.

*Ethyl acetate (1.4 mol/ Rh dimer) and water (0.8 mol/Rh dimer) were observed in the NMR spectra of compound **9a** as axial ligands; ethyl acetate: δ_H 1.29 (t), 2.04 (s), 4.22 (q); δ_C 14.2, 20.9, 60.9, 173.0; H_2O: δ_H 2.25.*

Dirhodium tetrakis [(2*S*)-2-(1″*R*,2″*R*,4″*S*)-fenchyloxy-2-(naphthalen-2′-yl)acetate] (2*S*-F-2′-NA) (9b)

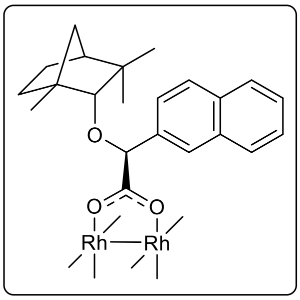

Sodium rhodium carbonate **S16** (0.24 g, 0.41 mmol), **8b** (1.10 g, 3.3 mmol) and water (50 mL) were used following the procedure for **9a** to give, following purification by column chromatography on silica gel employing hexane/ethyl acetate (92:8) as the eluent, the rhodium complex **9b** (0.50 g, 79%) as a green foamy solid. **Spec. Rot.:** $[\alpha]_D^{20}$ = +59.8 (*c* 0.2, $CHCl_3$); **^{1}H NMR (400 MHz, $CDCl_3$):** δ= 0.64 (3H, s), 0.73–0.97 (8H, m, contains 2H, m and 2 x 3H, s at 0.89, 0.91), 1.06–1.13 (1H, m), 1.24–1.36 (1H, m), 1.52–1.57 (1H, m), 1.62–1.73 (2H, m), 2.45 (1H, bs), 4.47 (1H, s), 7.16 (1H, dd, *J* 8.6, 1.2), 7.39–7.52 (3H, m), 7.57 (1H, d, *J* 8.6), 7.69–7.77 (2H, m); **^{13}C NMR (100.6 MHz, $CDCl_3$):** δ= 19.9, 20.9, 26.1, 31.0, 39.8, 41.0, 48.5, 49.2, 82.1, 91.6, 124.4, 125.89, 125.94, 126.1, 127.6, 127.7, 128.2, 132.9, 135.5, 191.7; **IR (neat):** 2927, 1685 (weak), 1605 (strong), 1402, 1014, 756; **HRMS (ESI-TOF):** *m/z* $[M+CH_3CN+H_2O+H]^+$ calcd for $C_{90}H_{106}NO_{13}Rh_2$, 1614.5769, found 1614.4830.

*Ethyl acetate (0.8 mol/ Rh dimer) and water (2 mol/Rh dimer) were observed in the NMR spectra of compound **9b** as axial ligands; ethyl acetate: δ_H 1.27 (t), 2.04 (s), 4.17 (q); δ_C 14.2, 21.0, 60.7; H_2O: δ_H 1.85.*

Dirhodium tetrakis [(2*S*)-2-(1″*R*,2″*R*,4″*S*)-fenchyloxy-2-(naphthalen-1′-yl)acetate] (2*S*-F-1′-NA) (9c)

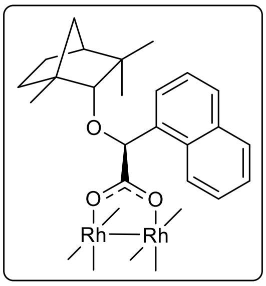

Sodium rhodium carbonate **S16** (37 mg, 0.06 mmol), **8c** (0.17 g, 0.51 mmol) in water (10 mL) were used following the procedure for **9a** to give, after purification by column chromatography on silica gel employing hexane/ethyl acetate (92:8) as the eluent, the rhodium complex **9c** (70 mg, 31%) as a green oil. **Spec. Rot.:** $[\alpha]_D^{20}$ = +92.6 (*c* 0.29, $CHCl_3$); **^{1}H NMR (400 MHz, $CDCl_3$):** δ= 0.67–0.93 (7H, m, contains, 1H, m, 3H, s at 0.73 and 3H, s at 0.86), 0.94–0.99 (1H, m), 1.07 (3H, s), 1.21–1.41 (2H, m), 1.50–1.70 (3H, m), 2.71 (1H, bs), 4.74 (1H, s), 7.15 (1H, d, *J* 6.9), 7.33 (2H, t, *J* 7.6), 7.42 (1H, t, *J* 7.6), 7.65 (1H, d, *J* 8.5), 7.75 (1H, d, *J* 8.1), 7.81 (1H, d, *J* 8.0); **^{13}C NMR (100.6 MHz, $CDCl_3$):** δ= 20.0, 21.0, 26.0, 26.1, 31.2, 40.0, 41.2, 48.6, 49.3, 79.2, 93.0, 124.7, 125.1, 125.3, 125.8, 125.9, 128.28, 128.31, 131.0, 133.6, 134.9, 191.1; **IR (neat):** 2947, 2869, 1684 (weak), 1605 (strong), 1388, 1118, 773; **HRMS (ESI-TOF):** *m/z* $[M+CH_3CN+H_2O]^+$ calcd for $C_{90}H_{105}NO_{13}Rh_2$, 1613.5691, found 1613.5752.

*Ethyl acetate (1.4 mol/ Rh dimer) and water (0.9 mol/Rh dimer) were observed in the NMR spectra of compound **9c** as axial ligands; ethyl acetate: δ_H 1.24 (t), 1.88 (s), 4.13 (q); δ_C 14.2, 20.8, 60.7, 172.4; H_2O: δ_H 1.85.*

Dirhodium tetrakis [(2*S*)-2-(4′-bromophenyl)-2-(1″*R*,2″*R*,4″*S*)-fenchyloxyacetate] (2*S*-FBrPA) (9d)

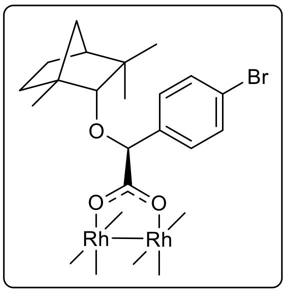

Sodium rhodium carbonate **S16** (0.26 g, 0.45 mmol), **8d** (1.33 g, 3.6 mmol) in water (50 mL) were used following the procedure for **9a** to give, after purification by column chromatography on silica gel employing hexane/ethyl acetate (92:8) as the eluent, the rhodium complex **9d** (0.47 g, 62%) as a green solid. **Spec. Rot.:** $[\alpha]_D^{20}$ = +17.6 (*c* 0.21, $CHCl_3$); **^{1}H NMR (400 MHz, $CDCl_3$):** δ= 0.80–1.03 (11H, m, contains 2H, m, 3 × 3H, s, one at 0.84 and two at 0.91), 1.22–1.41 (2H, m), 1.56–1.77 (3H, m), 2.46 (1H, d, *J* 1.4), 4.40 (1H, s), 7.06 (2H, d, *J* 8.4), 7.36 (2H, d, *J* 8.5); **^{13}C NMR (100.6 MHz, $CDCl_3$):** δ= 20.1, 20.9, 26.0, 26.1, 31.0, 39.8, 41.0, 48.4, 49.3, 81.2, 92.0, 121.8, 128.4, 131.2, 137.0, 191.3; **IR (neat):** 2948, 2858, 1685 (weak), 1604 (strong), 1386, 1129, 1010, 770.

*Ethyl acetate (0.4 mol/ Rh dimer) and water (1.8 mol/Rh dimer) were observed in the NMR spectra of compound **9d** as axial ligands; ethyl acetate: δ_H 1.30 (t), 2.06 (s), 4.23 (q); δ_C 14.2, 21.0, 60.9; H_2O: δ_H 2.16.*

Dirhodium tetrakis [(2*S*)-2-(1″*R*,2″*R*,4″*S*)-fenchyloxy-2-(4′-methoxyphenyl)acetate] (2*S*-FMeOPA) (9e)

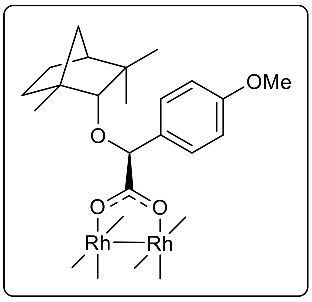

Sodium rhodium carbonate **S16** (84 mg, 0.14 mmol), **8e** (0.37 g, 1.15 mmol) in water (20 mL) were used following the procedure for **9a** to give, after purification by column chromatography on silica gel employing hexane/ethyl acetate (90:10) as the eluent, the rhodium complex **9e** (85 mg, 40%) as a green oil. **Spec. Rot.:** $[\alpha]_D^{20}$ = +45.4 (*c* 0.23, $CHCl_3$); **^{1}H NMR (400 MHz, $CDCl_3$):** δ= 0.78–1.00 (11H, m, contains 2H, m, 3 × 3H, s at 0.83, 0.91 and 0.93), 1.24–1.37 (2H, m), 1.53–1.61 (1H, m), 1.62–1.78 (2H, m), 2.48 (1H, bs), 3.75 (3H, s), 4.43 (1H, s), 6.76 (2H, d, *J* 8.6), 7.12 (2H, d, *J* 8.7); **^{13}C NMR (100.6 MHz, $CDCl_3$):** δ= 20.0, 20.9, 26.1, 26.2, 30.9, 39.8, 41.1, 48.5, 49.3, 55.1, 81.4, 91.3, 113.3, 128.0, 130.5, 158.9, 191.7; **IR (neat):** 2949, 2869, 1691 (weak), 1604 (strong), 1511, 1247, 1100; **HRMS (ESI-TOF):** *m*/*z* $[M+CH_3CN+H_2O+H]^+$ calcd for $C_{78}H_{106}NO_{17}Rh_2$, 1534.5565, found 1534.4889.

Ethyl acetate (1.4 mol/ Rh dimer) and water (0.9 mol/Rh dimer) were observed in the NMR spectra of compound ***9e*** *as axial ligands; ethyl acetate: δ_H 1.30 (t), 2.07 (s), 4.24 (q); δ_C 14.2, 21.0, 61.0, 173.1; H_2O: δ_H 2.59.*

Dirhodium tetrakis [(2*S*)-2-(1″*R*,2″*S*,5″*R*)-menthyloxy-2-phenylacetate] (2*S*-MPA) (9f)

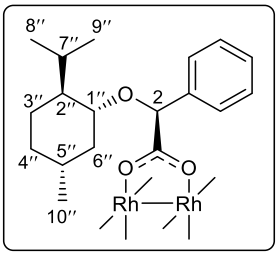

Sodium rhodium carbonate **S16** (0.12 g, 0.2 mmol), **8f** (0.47 g, 1.6 mmol) in water (25 mL) were used following the procedure for **9a** to give, after purification by column chromatography on silica gel employing hexane/ethyl acetate (90:10) as the eluent, the rhodium complex **9f** (0.11 g, 40%) as a foamy green solid. **Spec. Rot.:** $[\alpha]_D^{20}$ = −41.8 (*c* 0.2, $CHCl_3$); **^{1}H NMR (300 MHz, $CDCl_3$):** δ= 0.65 (1H, q, *J* 11.1), 0.73–0.99 (11H, m, contains, 2H, m, 3H, d, *J* 6.9 at 0.77, 3H, d, *J* 6.5 at 0.82 and 3H, d, *J* 7.0 at 0.91), 1.10–1.24 (2H, m), 1.53–1.66 (2H, m), 1.72–1.83 (1H, m), 2.35–2.49 (1H, m), 2.89 (1H, td, *J* 10.3, 3.9), 4.53 (1H, s), 7.07–7.14 (2H, m), 7.17–7.24 (3H, m); **^{13}C NMR (75.5 MHz, $CDCl_3$):** δ= 16.5, 21.3, 22.3, 23.1, 24.9, 31.3, 34.5, 40.3, 48.6, 78.2, 78.6, 126.9, 127.6, 128.1, 139.1, 191.6; **IR (neat):** 2922, 1602, 1398, 1099, 754; **HRMS (ESI-TOF):** *m*/*z* $[M+CH_3CN+H_2O+H]^+$ calcd for $C_{74}H_{106}NO_{13}Rh_2$, 1422.5769, found 1422.6068.

Ethyl acetate (0.8 mol/ Rh dimer) and water (1.2 mol/Rh dimer) were observed in the NMR spectra of compound ***9f*** *as axial ligands; ethyl acetate: δ_H 1.28 (t), 2.02 (s), 4.19 (q); δ_C 14.2, 21.0, 60.8, 172.5; H_2O: δ_H 2.81.*

Dirhodium tetrakis [(2*S*)-2-(1″*R*,2″*S*,5″*R*)-menthyloxy-2-(naphthalen-2′-yl)acetate] (2*S*-M-2′-NA) (9g)

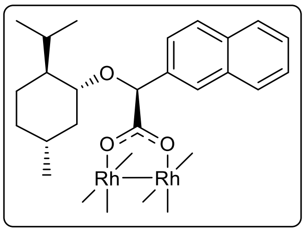

Sodium rhodium carbonate **S16** (0.11 g, 0.18 mmol), **8g** (0.50 g, 1.4 mmol) in water (20 mL) were used following the procedure for **9a** to give, after purification by column chromatography on silica gel employing hexane/ethyl acetate (92:8) as the eluent, the rhodium complex **9g** (55 mg, 29%) as a green oil. **Spec. Rot.:** $[\alpha]_D^{20}$ = −9.5 (*c* 0.2, $CHCl_3$); **^{1}H NMR (400 MHz, $CDCl_3$):** δ= 0.56 (1H, q, *J* 11.2), 0.65–0.99 (11H, m, contains, 2H, m, 3H, d, *J* 6.4 at 0.72, 3H, d, *J* 6.8 at 0.75, and 3H, d, *J* 7.0 at 0.91), 1.11–1.34 (2H, m), 1.45–1.67 (3H, m), 2.37–2.50 (1H, m), 2.79 (1H, td, *J* 10.3, 3.9), 4.52 (1H, s), 7.09 (1H, dd, *J* 8.5, 1.2), 7.39–7.48 (3H, m), 7.52 (1H, d, *J* 8.5), 7.72 (2H, d, *J* 7.8); **^{13}C NMR (100.6 MHz, $CDCl_3$):** δ= 16.5, 21.3, 22.3, 23.1, 24.9, 31.2, 34.4, 40.4, 48.5, 78.8, 79.3, 124.6, 125.8, 125.88, 125.89, 127.59, 127.64, 128.2, 132.9, 133.0, 136.5, 191.6; **IR (neat):** 2923, 1688 (weak), 1608 (strong), 1401, 1106, 758; **HRMS (ESI-TOF):** *m*/*z* $[M+CH_3CN+H_2O+H]^+$ calcd for $C_{90}H_{114}NO_{13}Rh_2$, 1622.6395, found 1622.6412.

*Ethyl acetate (0.7 mol/ Rh dimer) and water (1.2 mol/Rh dimer) were observed in the NMR spectra of compound **9g** as axial ligands; ethyl acetate: δ_H 1.28 (t), 2.02 (s), 4.19 (q); δ_C 14.2, 21.0, 60.6; H_2O: δ_H 2.25.*

Dirhodium tetrakis [(2*S*)-2-(4′-bromophenyl)-2-(1″*R*,2″*S*,5″*R*)-menthyloxyacetate] (2*S*-MBrPA) (9h)

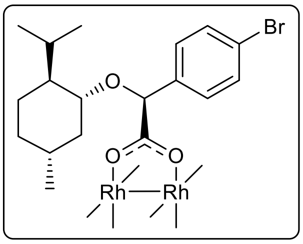

Sodium rhodium carbonate **S16** (58 mg, 0.10 mmol), **8h** (0.29 g, 0.79mmol) in water (15 mL) were used following the procedure for **9a** to give, after purification by column chromatography on silica gel employing hexane/ethyl acetate (95:5) as the eluent, the rhodium complex **9h** (73 mg, 50%) as a green solid. **Spec. Rot.:** $[\alpha]_D^{20}$ = −23.0 (*c* 0.12, $CHCl_3$); **^{1}H NMR (400 MHz, $CDCl_3$):** δ= 0.71–1.04 (12H, m, contains, 3H, m, 3H, d, *J* 6.8 at 0.74, 3H, d, *J* 6.4 at 0.83 and 3H, d, *J* 7.0 at 0.93), 1.10–1.35 (2H, m), 1.55–1.66 (2H, m), 1.68−1.97 (1H, m), 2.31–2.44 (1H, m), 2.86 (1H, td, *J* 10.3, 3.8), 4.45 (1H, s), 6.99 (2H, d, *J* 8.3), 7.35 (2H, d, *J* 8.4); **^{13}C NMR (100.6 MHz, $CDCl_3$):** δ= 16.5, 21.2, 22.3, 23.1, 25.0, 31.4, 34.4, 40.4, 48.5, 77.8, 79.4, 121.7, 128.5, 131.2, 138.0, 191.3; **IR (neat):** 2952, 2921, 1604, 1388, 1011, 772; **HRMS (ESI-TOF):** *m*/*z* $[M+CH_3CN+H_2O]^+$ calcd for $C_{74}H_{101}{}^{81}Br_4NO_{13}Rh_2$, 1737.2070, found 1737.2301

*Ethyl acetate (0.2 mol/ Rh dimer) and water (0.8 mol/Rh dimer) were observed in the NMR spectra of compound **9h** as axial ligands; ethyl acetate: δ_H 1.30 (t), 2.04 (s), 4.21 (q); δ_C 14.2, 20.9, 60.9, 173.0; H_2O: δ_H 2.49.*

3. Synthesis of 2,3-dihydrobenzofurans

3.1 Ester Synthesis

Methyl 2-(2-(benzyloxy)phenyl)acetate[15] (S17)

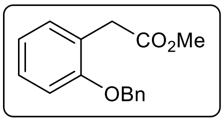

Concentrated sulfuric acid (1 mL) was added to a stirring solution of 2-(2-(benzyloxy)phenyl)acetic acid (4.70 g, 19.39 mmol) in methanol (50 mL) and the reaction mixture was heated under reflux for 3 h. The reaction mixture was cooled to room temperature followed by addition of anhydrous sodium bicarbonate (1.5 g). The mixture was filtered and concentrated under reduced pressure to afford methyl 2-(2-(benzyloxy)phenyl)acetate **S17** (4.65 g, quantitative) as a pale yellow oil which was used without further purification. Spectroscopic characteristics were consistent with previously reported data.[15] **^{1}H NMR (300 MHz, $CDCl_3$):** δ= 3.63 (3H, s), 3.69 (2H, s), 5.08 (2H, s), 6.87–6.98 (2H, m), 7.18–7.45 (7H, m); **IR (neat):** 2950, 1736 (CO), 1244, 1156, 1015, 750, 696.

Benzyl 2-(2-(benzyloxy)phenyl)acetate[16] (S18)

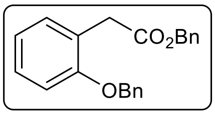

Anhydrous potassium carbonate (10.60 g, 77 mmol) was added in one portion to a stirring solution of 2-(2-hydroxyphenyl)acetic acid (5.0 g, 33.0 mmol) in *N*,*N*-dimethylformamide (75 mL) at room temperature. Neat benzyl bromide (7.8 mL, 66 mmol) was added dropwise to the reaction mixture followed by stirring at room temperature for 24 h. The reaction mixture was diluted with ether (100 mL) and water (50 mL), followed by gradual addition of aqueous hydrochloric acid (2.0 M, 100 mL). The layers were separated and the aqueous layer was extracted using ether (3 × 100 mL). The combined organic extracts were washed with aqueous hydrochloric acid (2.0 M, 2 × 75 mL), water (75 mL) and brine (75 mL), dried using magnesium sulfate and concentrated under reduced pressure to give the crude dibenzylated ester as a pale pink solid. The crude product was recrystallized from hot acetonitrile to give the purified dibenzylated ester **S18** (8.02 g, 73%) as a white solid. Spectroscopic characteristics were consistent with previously reported data.[16] **m.p.** 72–75 °C (Lit. 74.4–74.6 °C);[16] **^{1}H NMR (400 MHz, $CDCl_3$):** δ= 3.73 (2H, s), 5.04 (2H, s), 5.08 (2H, s), 6.88–6.97 (2H, m), 7.18–7.42 (12H, m); **IR (neat):** 1735 (CO), 1173, 1013, 728.

3.2 α-Diazo ester synthesis

Methyl 2-(2-(benzyloxy)phenyl)-2-diazoacetate[17] (10)

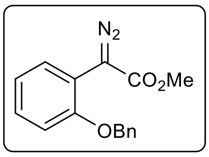

p-ABSA (7.26g, 30 mmol), methyl 2-(2-(benzyloxy)phenyl)acetate **S17** (2.56 g, 10 mmol), DBU (6.25 mL, 42 mmol) and DMSO (30 mL) were used following the procedure described for **6a** to give, following column chromatography on silica gel employing hexane/ethyl acetate (90:10) as the eluent, to give α-diazo ester **10** (2.71 g, 80%) as a yellow oil. Spectroscopic characteristics were consistent with previously reported data.[17] **^{1}H NMR (400 MHz, $CDCl_3$):** δ= 3.82 (3H, s), 5.10 (2H, s), 6.97 (1H, d, *J* 8.2), 7.03 (1H, t, *J* 7.6), 7.18–7.27 (1H, m), 7.29–7.44 (5H, m), 7.57 (1H, dd, *J* 7.8, 1.4); **^{13}C NMR (100.6 MHz, $CDCl_3$):** δ= 52.0, 59.8, 70.7, 112.2, 114.0, 121.5, 127.5, 128.1, 128.6, 130.4, 136.3, 154.7, 166.7; **IR (neat):** 2951, 2093 (CN_2), 1694 (CO), 1247, 745, 696.

Benzyl 2-(2-(benzyloxy)phenyl)-2-diazoacetate (12)

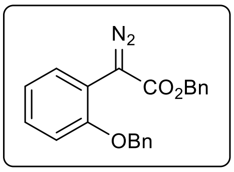

p-ABSA (2.17 g, 9 mmol), benzyl 2-(2-(benzyloxy)phenyl)acetate **S18** (1.38 g, 4.2 mmol), DBU (2.0 mL, 14 mmol) and DMSO (20 mL) were used following the procedure described for **6a** to give, following column chromatography on silica gel employing hexane/ethyl acetate (90:10) as the eluent, α-diazo ester **12** (1.25 g, 83%) as an yellow oil; **^{1}H NMR (400 MHz, $CDCl_3$):** δ= 5.10 (2H, s), 5.28 (2H, s), 6.97 (1H, d, *J* 8.3), 7.03 (1H, td, *J* 7.6, 1.0), 7.20–7.24 (1H, m), 7.29–7.44 (10H, m), 7.59 (1H, dd, *J* 7.7, 1.4); **^{13}C NMR (100.6 MHz, $CDCl_3$):** δ= 59.9, 66.4, 70.7, 112.2, 114.0, 121.5, 127.6, 128.10, 128.15, 128.18, 128.56, 128.58, 128.61, 130.4, 136.1, 136.3, 154.7, 166.1; **IR (neat):** 2093 (CN_2), 1693 (CO), 1242, 1147, 1009, 741, 694; **HRMS (ESI-TOF):** *m/z* $[M+H]^+$ calcd for $C_{22}H_{19}N_2O_3$ 359.1396, found 359.1391.

Isopropyl 2-(2-(benzyloxy)phenyl)-2-diazoacetate (13)

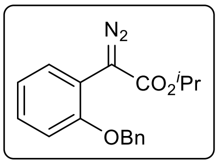

Elemental sodium (0.14 g, 6 mmol) was added to IPA (20 mL) at 0 °C. The mixture was removed from the ice bath and stirred at room temperature for 30 minutes. Benzyl 2-(2-(benzyloxy)phenyl)-2-diazoacetate **12** (0.1 g, 2.8 mmol) was dissolved in IPA (20 mL) and added to the stirring sodium isoproproxide solution. The resulting solution was stirred at room temperature for 48 h then it was evaporated onto silica gel and following column chromatography on silica gel employing hexane/ethyl acetate (95:5) as the eluent, α-diazo ester **13** (0.49 g, 57%) was isolated as a yellow oil; **^{1}H NMR (400 MHz, $CDCl_3$):** δ= 1.29, (6H, d, *J* 6.2), 5.10 (2H, s), 5.16 (1H, septet, *J* 6.3), 6.96 (1H, d, *J* 8.2), 7.03 (1H, t, *J* 7.0, with further unresolved splitting), 7.18–7.25 (1H, m), 7.30–7.47 (5H, m), 7.59 (1H, dd, *J* 7.8, 1.4); **^{13}C NMR (100.6 MHz, $CDCl_3$):** δ= 22.1, 68.5, 70.7, 112.2, 114.3, 121.4, 127, 128, 128.3, 128.6, 130.3, 136.4, 154.6, 165.9; **IR (neat):** 2093 (CN_2), 1692 (CO), 1244, 1003, 746, 696; **HRMS (ESI-TOF):** *m/z* $[M+H]^+$ calcd for $C_{18}H_{19}N_2O_3$ 311.1396, found 311.1390.

3.3 Rhodium Catalysed C–H Insertion Reactions - 2,3-Dihydrobenzofuran Synthesis

Table SI.1: Investigation of reaction conditions on the cyclisation of **10**[a]

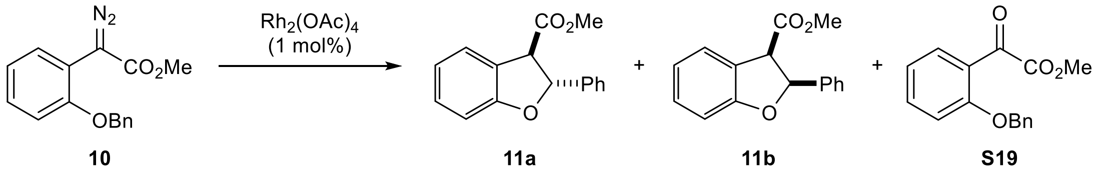

Entry	Solvent	Atmospheric Conditions	Temperature (°C)	11a:11b:S19[b]
**1**	toluene	under N_2	rt	53:32:15[c]
**2**	DCM	under N_2	rt	1.3:0.7:98[d]
**3**	toluene	under N_2	−60→rt[e]	0.8:0.8:98.4
**4**	toluene	open to air	rt	0.7:0.8:98.5
**5**	toluene	Schlenk	rt	46:28:26
**6**	toluene	degassed solvent, Schlenk	rt	60:35:5

[a]The general procedure for the $Rh_2(OAc)_4$ catalysed C–H insertion involved the dropwise addition of a solution of aryl diazoacetate (in 8 mL solvent) to a stirring suspension of rhodium acetate (in 3 mL solvent). Reactions were monitored by IR spectroscopy and were generally complete within 30 min. [b]The ratio of **11a**:**11b:S19** was determined by the relative integration of signals at δ_H 6.12 (1H, d), 5.99 (1H, d) and 7.91 (1H, dd) ppm, respectively, in the ^{1}H NMR spectra of the crude reaction mixtures.[c]Experiment was repeated and the ratio of **11a**:**11b**:**S19** was 58:35:7. [d]Experiment was repeated and the ratio of **180a**:**180b**:**S19** was 2:1.5:96.5. [e]The reaction mixture was found to still contain aryldiazoacetate (by IR spectroscopy of a sample withdrawn) after 1.5 h stirring between −50 °C and −60 °C so was allowed to warm up to room temperature over 30 min.

Procedure A:

General procedure for rhodium catalysed C–H insertion reactions to afford 2,3-dihydrobenzofurans

A solution of α-diazoacetate (0.10 g, 1 equiv.) in HPLC grade toluene or freshly distilled DCM (8 mL, further degassed using freeze, pump, thaw technique) was added dropwise via syringe pump over ~30 min to a stirring solution of rhodium(II) catalyst (1 mol%) in HPLC grade toluene or freshly distilled DCM (3 mL). The reaction was carried out at 0–3 °C or –45 °C using Schlenk techniques. The mixture was stirred at this temperature for 30 minutes after addition was complete. The mixture was then concentrated under reduced pressure to provide the crude product(s), a ^{1}H NMR spectrum obtained and following column chromatography on silica gel (25 g) employing hexane/ethyl acetate (99:1) as the eluent, both isomers of the dihydrobenzofuran were isolated. The less polar trans isomers were isolated as colourless oils while the more polar cis isomers were found to be white solids.

3-Methoxycarbonyl-2-phenyl-2,3-dihydrobenzofuran[17] (11)

The title compund was prepared according to ***Procedure A*** from methyl 2-(2-(benzyloxy)phenyl)-2-diazoacetate **10** (100 mg, 0.35 mmol) and Rh_2(2*S*-M-2′-NA)$_4$ **9g** (6 mg, 1 mol%) in toluene (11 mL) at −45 °C.

(2*R*,3*R*)-trans-3-Methoxycarbonyl-2-phenyl-2,3-dihydrobenzofuran[17] (11a)

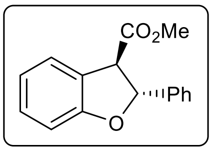

Spectroscopic characteristics were consistent with previously reported data.[17] **11a**, Colourless oil (45.2 mg, 50%); **Spec. Rot.:** $[\alpha]_D^{20}$ = −67.6 (*c* 1.39, $CHCl_3$) [lit. $[\alpha]_D^{21}$ = −58.2 (*c* 1.12, $CHCl_3$) for 80% ee of (2*R*,3*R*)-**11a**]; **HPLC:** 86% ee (determined by chiral phase HPLC, see **Table SI.6** for HPLC conditions); **^{1}H NMR (400 MHz, $CDCl_3$):** δ= 3.82 (3H, s), 4.29 (1H, d, *J* 7.4), 6.12 (1H, d, *J* 7.4), 6.86–6.98 (2H, m), 7.20–7.29 (1H, m), 7.29–7.46 (6H, m); **IR (neat):** 2922, 1736 (CO), 1478, 1234, 748. The preferred absolute configuration of **11a** was determined to be (2*R*,3*R*) by comparison of the recorded optical rotation values and HPLC data with literature reports.

(2*S*,3*R*)-cis-3-Methoxycarbonyl-2-phenyl-2,3-dihydrobenzofuran[17] (11b)

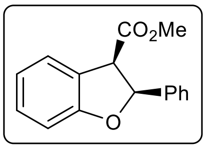

Spectroscopic characteristics were consistent with previously reported data.[17] **11b**, white solid (3.5 mg, 4%); **m.p.** 90–92 °C [lit. 90–91 °C][17]; **Spec. Rot.:** $[\alpha]_D^{20}$ = −16.3 (*c* 0.01, $CHCl_3$) [lit. $[\alpha]_D^{21}$ = +57.9 (*c* 1.00, $CHCl_3$) for 70% ee of (2*R*,3*S*)-**11b**][17]; **HPLC:** 18% ee (determined by chiral phase HPLC, see **Table SI.6** for HPLC conditions); **^{1}H NMR (400 MHz, $CDCl_3$):** δ= 3.21 (3H, s,), 4.62 (1H, d, *J* 9.8), 5.99 (1H, d, *J* 9.8), 6.91–7.00 (2H, m), 7.20–7.45 (7H, m); **IR (neat):** 2980, 1727 (CO), 1480, 1104,748. The preferred absolute configuration of **11b** was determined to be (2*S*,3*R*) by specific rotation and HPLC analysis following conversion to (2*S*,3*S*)-**11a** (*vide infra*).

Isomerization Reaction: Preparation of (2*S*,3*S*)-11a from (2*S*,3*R*)-11b

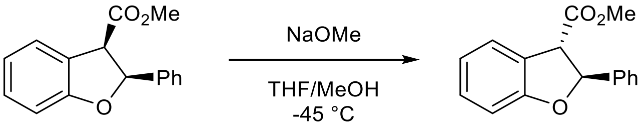

(2*S*,3*R*)-11b **(2*S*,3*S*)-11a**

To a solution of **11b** (8.9 mg, 0.035 mmol, 33% ee) in THF (0.8 mL) at –45 °C was added a solution of NaOMe in MeOH (0.1 M, 0.08 mL, 0.008 mmol, 0.44 eq). After 0.5 h of stirring at –45 °C, the reaction was quenched with saturated ammonium chloride solution (3 mL) and the whole mixture was extracted with EtOAc (3 x 6 mL). The combined organic extracts were washed with water (2 x 5 mL) and brine (2 x 5 mL), and dried over anhydrous $MgSO_4$. Filtration and evaporation furnished the crude product (8.8 mg as colorless oil), $[\alpha]_D^{20}$ = +13.90 (*c* 0.41, $CHCl_3$). The enantiomeric excess of **11a** was determined to be 33% by chiral HPLC analysis (*vide supra*).

Methyl 2-(2-(benzyloxy)phenyl)-2-oxoacetate[18] (S19)

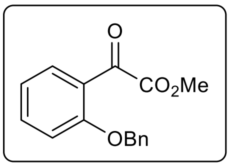

Spectroscopic characteristics were consistent with previously reported data.[18] White solid; **m.p.** 84–86 °C [Lit. 84–85 °C];[18] **^{1}H NMR (400 MHz, $CDCl_3$):** δ= 3.34 (3H, s), 5.08 (2H, s), 7.03–7.13 (2H, m), 7.32–7.47 (5H, m), 7.54–7.62 (1H, m), 7.91 (1H, dd, *J* 7.7, 1.4); **^{13}C NMR (100.6 MHz, $CDCl_3$):** δ= 51.9, 71.3, 112.8, 121.5, 122.8, 128.5, 128.6, 128.7, 131.0, 135.2, 136.3, 159.5, 165.5, 186.5; **IR (neat):** 1739 (CO), 1665, 1596 (CO), 1274, 1009, 751, 698; **HRMS (ESI-TOF):** *m/z* $[M+H]^+$ calcd for $C_{16}H_{15}O_4$, 271.0970, found 287.0973.

Table SI.2: Investigation of reaction conditions in the enantioselective cyclisation of **12**[a]

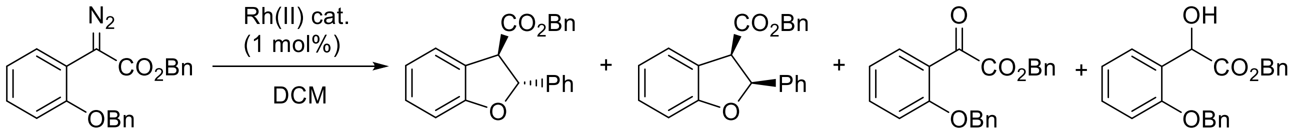

12 **14a** **14b** **S20** **S21**

Entry	Catalyst	Temp (°C)	14a:14b:S20:S21[b]	Yield[c] 14a (%)	Yield[c] 14b (%)	Enantiopurity[d,e] 14a (% ee)	Enantiopurity[d,e] 14b (% ee)
1[f]	Rh_2(*S*-PTTL)$_4$ **3**	−45	1.2:1.5:97.3:0	-	-	-	-
2[f]	Rh_2(*S*-PTTL)$_4$ **3**	−20	0.7:1.3:5:93	-	-	-	-
3[f]	Rh_2(*S*-PTTL)$_4$ **3**	−5	0.7:4.5:5.7:89.1	-	-	-	-
4	Rh_2(*S*-PTTL)$_4$ **3**	0–3	1:61.5:9:28.5[g]	-	69	-	98[h]
5	Rh_2(*S*-PTTL)$_4$ **3**	0–3	1.5:51.5:11:36[i]	-	55	-	98[h]
6	Rh_2(2*S*-MPA)$_4$ **9f**	−45	3.9:2.1:12.3:81.7[j]	3	1	-[k]	-[k]
7	Rh_2(2*S*-MPA)$_4$ **9f**	0–3	48.5:28:7.5:16[l]	18	-[k]	78	-[k]
8[f]	Rh_2(2*S*-M-2′-NA)$_4$ **9g**	−45	9:7.8:19.2:64[j]	-[k]	-[k]	-[k]	-[k]
9	Rh_2(2*S*-M-2′-NA)$_4$ **9g**	−45	54.5:19.5:9:17[m]	26	8	75[n]	60[o]
10	Rh_2(2*S*-F-1′-NA)$_4$ **9c**	0–3	9:74:4:13[i]	3	42	91[n]	62[o]
11	Rh_2(2*S*-FBrPA)$_4$ **9d**	0–3	56:28.8:5:10.2[g]	27	10	78[n]	70[o]

[a]Reactions conducted using the general **Procedure A** for rhodium-catalysed C–H insertion reactions. [b]The ratio of **14a**:**14b**:**S20:S21** was determined by the relative integration of signals at 6.13 (1H, d), 5.98 (1H, d), 7.93 (1H, dd) and 5.41 (1H, d), respectively, in the ^{1}H NMR spectra of the crude reaction mixtures. [c]Isolated yield following column chromatography. [d]Enantiopurity determined by chiral stationary phase HPLC (see **Table SI.6** for further details). [e]Stereochemical assignments made by analogy to **11a** and **11b.** [f]Crude reaction mixture not purified. [g]Crude reaction mixture contains ~10% unknown side-product. [h]Stereochemistry assigned as 2*R*,3*S* by analogy to **11b**. [i]Crude reaction mixture contained ~5% unknown side-product. [j]Crude reaction mixture contained ~40% unknown side-product. [k]A sample of sufficient purity to allow accurate determination of enantiopuritywas not isolated. [l]Crude reaction mixture contained ~20% unknown side-product. [m]Crude reaction mixture contained ~50% unknown side-product. [n]Stereochemistry assigned as 2*R*,3*R* by analogy to **11a**. [o]Stereochemistry assigned as 2*S*,3*R* by analogy to **11b**.

Figure SI.1: Ratio of known products in Rh(II) catalysed reaction of **12**.

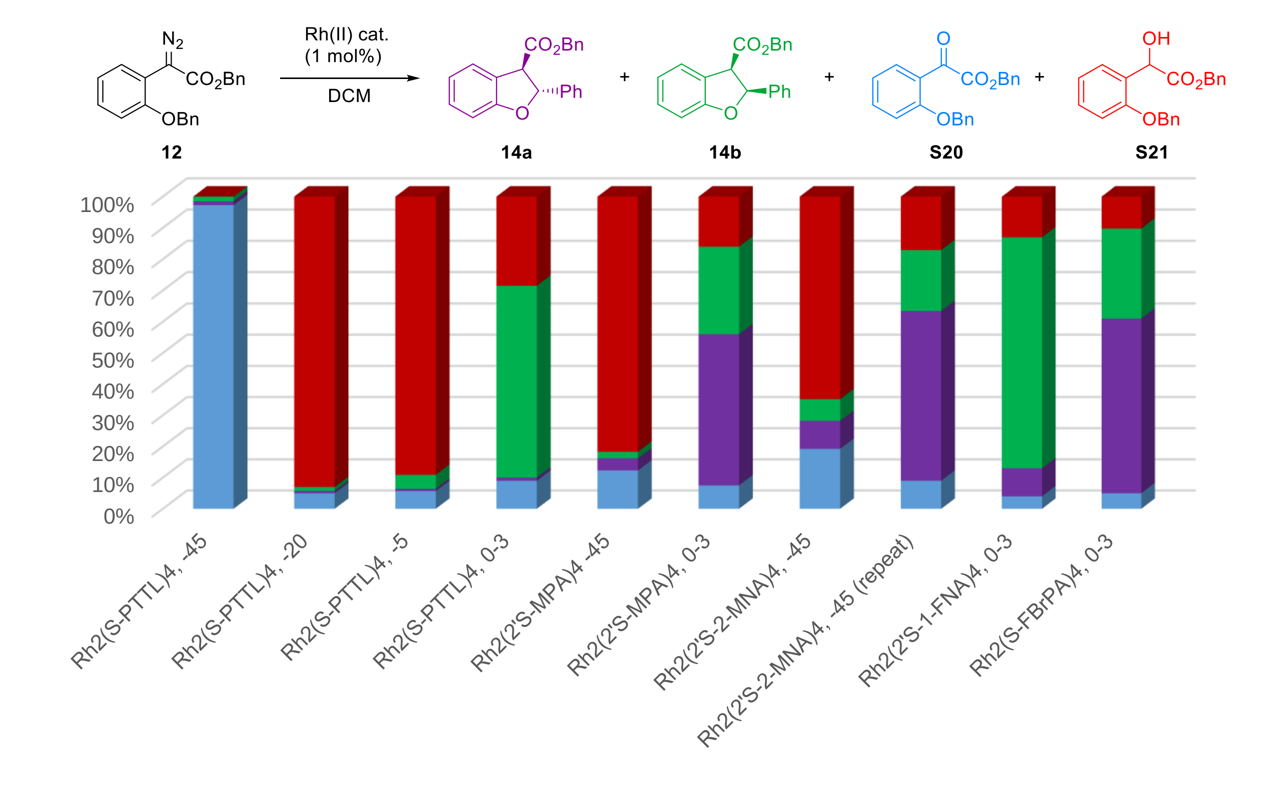

3-Benxyloxycarbonyl-2-phenyl-2,3-dihydrobenzofuran (14)

The title compund was prepared according to ***Procedure A*** from benzyl 2-(2-benzyloxyphenyl)-2-diazoacetate **12** (100 mg, 0.28 mmol) and Rh_2(2*S*-MPA)$_4$ **9f** (4 mg, 1 mol%) in toluene (11 mL).

(2*R*,3*R*)-trans-3-Benzyloxycarbonyl-2-phenyl-2,3-dihydrobenzofuran (14a)

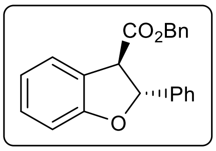

Colourless oil (50 mg, 54%); **Spec. Rot.:** $[\alpha]_D^{20}$ = −76.2 (*c* 2.11, $CHCl_3$); **HPLC:** 79% ee (determined by chiral phase HPLC, see **Table SI.6** for HPLC conditions); **^{1}H NMR (400 MHz, $CDCl_3$):** δ= 4.32 (1H, d, *J* 7.5), 5.20–5.29 (2H, fine AB q, *J* 12.5), 6.13 (1H, d, *J* 7.5), 6.86–6.95 (2H, m), 7.20–7.28 (1H, m), 7.28–7.43 (11H, m, Ar*H*); **^{13}C NMR (100.6 MHz, $CDCl_3$):** δ= 55.9, 67.5, 85.5, 110.0, 121.0, 123.7, 125.3, 125.8, 128.3, 128.4, 128.5, 128.7, 128.8, 129.7, 135.4, 140.6, 159.4, 170.7; **IR (neat):** 3034, 1733 (CO), 1478, 1232, 748, 696; **HRMS (ESI-TOF):** *m/z* $[M+H]^+$ calcd for $C_{22}H_{19}O_3$, 331.1334, found 331.1345.

(2*S*,3*R*)-cis-3-Benzyloxycarbonyl-2-phenyl-2,3-dihydrobenzofuran (14b)

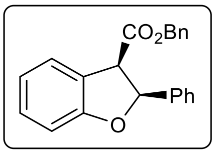

White solid (15 mg, 16%); **m.p.** 89–90 °C; **Spec. Rot.:** $[\alpha]_D^{20}$ = −10.4 (*c* 0.47, $CHCl_3$); **HPLC:** 22% ee (determined by chiral phase HPLC, see **Table SI.6** for HPLC conditions); **^{1}H NMR (400 MHz, $CDCl_3$):** δ= 4.50 (1H, d, *J* 12.2), 4.64 (1H, d, *J* 9.8), 4.71 (1H, d, *J* 12.3), 5.98 (1H, d, *J* 9.8), 6.90–7.03 (4H, m), 7.19–7.40 (10H, m); **^{13}C NMR (100.6 MHz, $CDCl_3$):** δ= 53.8, 66.7, 85.7, 110.0, 121.3, 124.8, 125.9, 126.4, 128.17, 128.25, 128.28, 128.4, 128.5, 129.7, 135.2, 137.0, 160.4, 169.8; **IR (neat):** 3033, 1733 (CO), 1478, 1150, 748, 696; **HRMS (ESI-TOF):** *m/z* $[M+H]^+$ calcd for $C_{22}H_{19}O_3$, 331.1334, found 331.1345.

Methyl 2-(2-(benzyloxy)phenyl)-2-oxoacetate (S20)

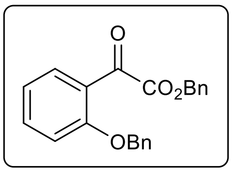

^{1}H NMR (400 MHz, $CDCl_3$): δ=4.78 (2H, s), 4.99 (2H, s,), 6.99–7.04 (1H, m), 7.05–7.12 (1H, m), 7.16–7.43 (10H, m), 7.52–7.60 (1H, m), 7.93 (1H, dd, *J* 7.8, 1.5).

Benzyl 2-(2-(benzyloxy)phenyl)-2-hydroxyacetate (S21)

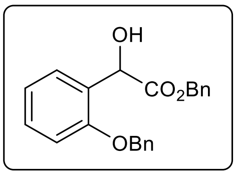

^{1}H NMR (400 MHz, $CDCl_3$): δ= 3.60 (1H, d, *J* 6.0, appears reduced in a D_2O shake), 4.94–5.07 (2H, ABq, *J* 11.9), 5.08–5.18 (2H, ABq, *J* 12.4), 5.41 (1H, d, 4.3), 6.89–7.01 (2H, m), 7.11–7.19 (2H, m), 7.22–7.39 (10H, m); **^{13}C NMR (100.6 MHz, $CDCl_3$):** δ= 67.2, 70.2, 70.3, 112.3, 121.1, 127.2, 127.9, 128.0, 128.2, 128.5, 128.6, 129.5, 129.9, 135.4, 136.5, 156.2, 173.5; **IR (neat):** 3100–3600, 1733 (CO), 1492, 1453, 1242, 1246, 752, 696.

3-Isopropyloxycarbonyl-2-phenyl-2,3-dihydrobenzofuran (15)

The title compund was prepared according to ***Procedure A*** from isopropyl 2-(2-benzyloxyphenyl)-2-diazoacetate **13** (100 mg, 0.32 mmol) and Rh_2(2*S*-MPA)$_4$ **9f** (4 mg, 1 mol%) in toluene (11 mL).

(2*R*,3*R*)-trans-3-Isopropyloxycarbonyl-2-phenyl-2,3-dihydrobenzofuran (15a)

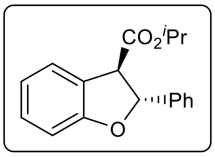

Colourless oil (31.8 mg, 35%); **Spec. Rot.:** $[\alpha]_D^{20}$ = −53.6 (*c* 1.20, $CHCl_3$); **HPLC:** 78% ee (determined by chiral phase HPLC, see **Table SI.6** for HPLC conditions); **^{1}H NMR (400 MHz, $CDCl_3$):** δ= 1.30 (3H, d, *J* 6.2), 1.31 (3H, d, *J* 6.2), 4.23 (1H, d, *J* 7.7), 5.13 (1H, septet, *J* 6.3), 6.11 (1H, d, *J* 7.7), 6.86–6.97 (2H, m), 7.18–7.28 (1H, m), 7.28–7.45 (6H, m); **^{13}C NMR (100.6 MHz, $CDCl_3$):** δ= 21.86, 21.89, 55.9, 69.3, 85.5, 109.9, 120.9, 124.2, 125.0, 125.8, 128.3, 128.8, 129.6, 140.8, 159.3, 170.3; **IR (neat):** 2981, 1729 (CO), 1478, 1234, 749, 698; **HRMS (ESI-TOF):** *m/z* $[M+H]^+$ calcd for $C_{18}H_{19}O_3$, 283.1334, found 283.1310.

(2*S*,3*R*)-cis-3-Isopropyloxycarbonyl-2-phenyl-2,3-dihydrobenzofuran (15b)

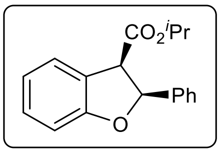

White solid (15.4 g, 17%); **m.p.** 56–58 °C; **Spec. Rot.:** $[\alpha]_D^{20}$ = -12.6 (*c* 0.12, $CHCl_3$); **HPLC:** 27% ee (determined by chiral phase HPLC, see **Table SI.6** for HPLC conditions); **^{1}H NMR (400 MHz, $CDCl_3$):** δ= 0.67 (3H, d, *J* 6.2), 0.92 (3H, d, *J* 6.3), 4.52–4.63 (2H, m, contains 1H, septet), 5.97 (1H, d, *J* 9.9), 6.91–6.99 (2H, m), 7.21–7.42 (7H, m); **^{13}C NMR (100.6 MHz, $CDCl_3$):** δ= 20.9, 21.4, 53.7, 68.5, 85.6, 109.9, 121.2, 125.1, 125.9, 126.6, 128.2, 128.3, 129.4, 137.3, 160.3, 169.4; **IR (neat):** 2984, 1727 (CO), 1480, 1101, 757, 695; **HRMS (ESI-TOF):** *m*/*z* $[M+H]^+$ calcd for $C_{18}H_{19}O_3$, 283.1334, found 283.1310.

4. Synthesis of Thiopyran Dioxides

4.1 Preparation of α-Diazo-β-oxosulfone Compounds

Methyl 2-diazo-2-((4'-phenylbutyl)sulfonyl)acetate[19] (16)

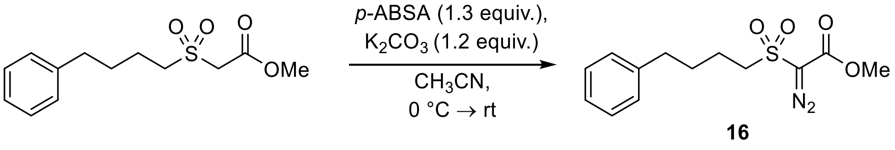

Potassium carbonate (3.07 g, 22.2 mmol) was added to a stirring solution of methyl 2-((4'-phenylbutyl)sulfonyl)acetate[19a] (5.0 g, 18.5 mmol) in acetonitrile (100 mL) at room temperature. The reaction mixture was stirred for 10 min before being cooled to 0 °C while a solution of 4-acetamidobenzenesulfonyl azide (*p*-ABSA) (5.78 g, 24.0 mmol) in acetonitrile (50 mL) was added. The reaction mixture was stirred at 0 °C for 30 min, returned to room temperature and stirred overnight before the addition of a non-polar co-solvent, hexane (60 mL) and diethyl ether (30 mL), to precipitate sulfonamide salts. The reaction mixture was stirred for a further 15 minutes, concentrated under reduced pressure and dichloromethane was added in order to decant from the bulk sulfonamide salts. Purification by column chromatography on silica gel, using ethyl acetate/hexane (20:80 to 40:60) as eluent, gave pure methyl 2-diazo-2-((4'-phenylbutyl)sulfonyl)acetate **16** as a yellow oil (5.08 g, 93%). Spectroscopic characteristics were consistent with previously reported data.[19] **^{1}H NMR (300 MHz, $CDCl_3$):** δ= 1.69–1.94 (4H, m), 2.65 (2H, t, *J* 7.2), 3.34–3.43 (2H, m), 3.85 (3H, s), 7.11–7.23 (3H, m), 7.23–7.33 (2H, m); **^{13}C NMR (75.5 MHz, $CDCl_3$):** δ= 22.1, 29.6, 35.1, 53.0, 56.4, 72.8, 126.0, 128.2, 128.4, 141.0, 160.4; **IR (neat):** 2125 (CN_2), 1713 (CO), 1332, 1293, 1145, 1083 (SO_2); **HRMS (ESI-TOF):** *m/z* $[M+H]^+$ calcd for $C_{13}H_{17}N_2O_4S$, 297.0904, found 297.0907.

Methyl 2-diazo-2-((4'-(*p*-tolyl)butyl)sulfonyl)acetate[19] (19)

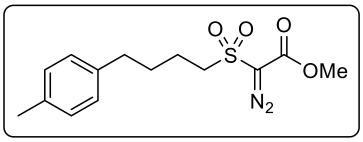

The title compound was prepared using the procedure described for methyl 2-diazo-2-((4'-phenylbutyl)sulfonyl)acetate **16**, using potassium carbonate (1.75 g, 12.7 mmol), methyl 2-((4'-(*p*-tolyl)butyl)sulfonyl)acetate[19a] (3.0 g, 10.6 mmol) in acetonitrile (100 mL) and *p*-ABSA (3.30 g, 13.7 mmol) in acetonitrile (30 mL). The mixture was stirred at 0 °C for 30 min, returned to room temperature and stirred overnight. Purification by column chromatography on silica gel, using ethyl acetate/hexane (20:80) as eluent, gave the pure product methyl 2-diazo-2-((4'-(*p*-tolyl)butyl)sulfonyl)acetate **19** as a yellow oil (2.68 g, 82%) which solidified upon storage in the freezer. Spectroscopic characteristics were consistent with previously reported data.[19] **m.p.** 35–37 °C; **^{1}H NMR (300 MHz, $CDCl_3$):** δ= 1.66–1.91 (4H, m), 2.30 (3H, s), 2.60 (2H, t, *J* 7.3), 3.32–3.42 (2H, m), 3.84 (3H, s), 7.03 (2H, apparent d, *J* 8.0), 7.08 (2H, apparent d, *J* 8.0); **^{13}C NMR (75.5 MHz, $CDCl_3$):** δ= 20.8, 22.0, 29.6, 34.5, 52.8, 56.3, 72.7, 128.0, 129.0, 135.3, 137.8, 160.3; **IR (neat):** 2125 (CN_2), 1713 (CO), 1333, 1293, 1145, 1082 (SO_2).

Methyl 2-diazo-2-((4'-(4''-methoxyphenyl)butyl)sulfonyl)acetate[19] (20)

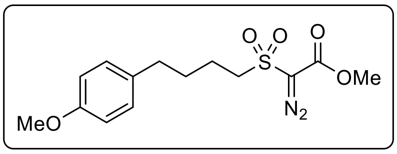

The title compound was prepared using the procedure described for methyl 2-diazo-2-((4'-phenylbutyl)sulfonyl)acetate **16**, using potassium carbonate (1.66 g, 12.0 mmol), methyl 2-((4'-(4''-methoxyphenyl)butyl)sulfonyl)acetate[19a] (3.0 g, 10.0 mmol) in acetonitrile (100 mL) and *p*-ABSA (3.12 g, 13.0 mmol) in acetonitrile (30 mL). The mixture was stirred at 0 °C for 30 min, returned to room temperature and stirred overnight. Purification by column chromatography on silica gel, using ethyl acetate/hexane (20:80) as eluent, gave the pure product methyl 2-diazo-2-((4'-(4''-methoxyphenyl)butyl)sulfonyl)acetate **20** as a yellow solid (2.74 g, 84%). Spectroscopic characteristics were consistent with previously reported data.[19] **m.p.** 50–51 °C (lit. 49–51 °C);[19b] **^{1}H NMR (300 MHz, $CDCl_3$):** δ= 1.65–1.93 (4H, m), 2.59 (2H, t, *J* 7.3), 3.30–3.43 (2H, m), 3.78 (3H, s), 3.85 (3H, s), 6.82 (2H, apparent d, *J* 8.6), 7.07 (2H, apparent d, *J* 8.6); **^{13}C NMR (75.5 MHz, $CDCl_3$):** δ= 22.0, 29.8, 34.2, 53.0, 55.2, 56.4, 72.8, 113.8, 129.1, 133.0, 157.9, 160.4; **IR (neat):** 2117 (CN_2), 1724 (CO), 1331, 1299, 1147 (SO_2).

Methyl 2-diazo-2-((4'-(4''-fluorophenyl)butyl)sulfonyl)acetate[19a] (21)

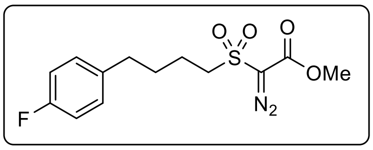

The title compound was prepared using the procedure described for methyl 2-diazo-2-((4'-phenylbutyl)sulfonyl)acetate **16**, using potassium carbonate (1.81 g, 13.1 mmol), methyl 2-((4'-(4''-fluorophenyl)butyl)sulfonyl)acetate[19a] (3.15 g, 10.9 mmol) in acetonitrile (100 mL) and *p*-ABSA (3.41 g, 14.2 mmol) in acetonitrile (30 mL). The mixture was stirred at 0 °C for 30 min, returned to room temperature and stirred overnight. Purification by column chromatography on silica gel, using ethyl acetate/hexane (20:80) as eluent, gave the pure product methyl 2-diazo-2-((4'-(4''-fluorophenyl)butyl)sulfonyl)acetate **21** as a yellow oil (3.03 g, 88%). Spectroscopic characteristics were consistent with previously reported data.[19a] **^{1}H NMR (300 MHz, $CDCl_3$):** δ= 1.64–1.94 (4H, m), 2.63 (2H, t, *J* 7.3), 3.35–3.44 (2H, m), 3.86 (3H, s), 6.91–7.02 (2H, m), 7.06–7.16 (2H, m); **^{13}C NMR (75.5 MHz, $CDCl_3$):** δ= 22.0, 29.7, 34.3, 53.0, 56.3, 72.8, 115.1 (d, $^2J_{CF}$ 21.2), 129.6 (d, $^3J_{CF}$ 7.9), 136.6 (d, $^4J_{CF}$ 3.3), 160.4, 161.3 (d, $^1J_{CF}$ 244.1); **IR (neat):** 2126 (CN_2), 1713 (CO), 1333, 1294, 1216, 1145, 1083 (SO_2).

2-Diazo-1-phenyl-2-((4'-phenylbutyl)sulfonyl)ethan-1-one[19] (22)

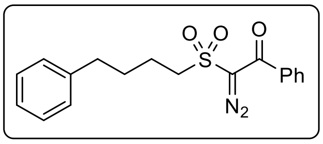

The title compound was prepared using the procedure described for methyl 2-diazo-2-((4'-phenylbutyl)sulfonyl)acetate **16**, using potassium carbonate (1.57 g, 11.38 mmol), 1-phenyl-2-((4'-phenylbutyl)sulfonyl)ethan-1-one[19a] (3.0 g, 9.48 mmol) in acetonitrile (100 mL) and *p*-ABSA (2.96 g, 12.33 mmol) in acetonitrile (30 mL). The mixture was stirred at 0 °C for 30 min, returned to room temperature and stirred overnight. Purification by column chromatography on silica gel, using ethyl acetate/hexane (10:90–30:70) as eluent, gave the pure product 2-diazo-1-phenyl-2-((4'-phenylbutyl)sulfonyl)ethan-1-one **22** as a yellow solid (2.79 g, 86%). Spectroscopic characteristics were consistent with previously reported data.[19] **m.p.** 93–95 °C (lit. 97–99 °C);[19b] **^{1}H NMR (300 MHz, $CDCl_3$):** δ= 1.73–1.95 (4H, m), 2.66 (2H, t, *J* 7.2), 3.50–3.60 (2H, m), 7.10–7.21 (3H, m), 7.22–7.31 (2H, m), 7.45–7.54 (2H, m), 7.56–7.67 (3H, m); **^{13}C NMR (75.5 MHz, $CDCl_3$):** δ= 22.1, 29.6, 35.1, 56.5, 80.1, 126.0, 127.3, 128.3, 128.4, 129.0, 133.3, 135.6, 141.0, 183.3; **IR (neat):** 2112 (CN_2), 1647 (CO), 1327, 1282, 1224, 1137 (SO_2).

Benzyl 2-diazo-2-(dodecylsulfonyl)acetate[19] (23)

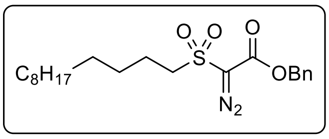

The title compound was prepared using the procedure described for methyl 2-diazo-2-((4'-phenylbutyl)sulfonyl)acetate **16**, using potassium carbonate (2.17 g, 15.7 mmol), benzyl 2-(dodecylsulfonyl)acetate[19a] (5.0 g, 13.1 mmol) in acetonitrile (100 mL) and *p*-ABSA (4.08 g, 17.0 mmol) in acetonitrile (50 mL). The mixture was stirred at 0 °C for 30 min, returned to room temperature and stirred overnight. Purification by column chromatography on silica gel, using ethyl acetate/hexane (20:80) as eluent, gave the pure product benzyl 2-diazo-2-(dodecylsulfonyl)acetate **23** as a yellow solid (4.19 g, 78%). Spectroscopic characteristics were consistent with previously reported data.[19b] **m.p.** 50–51 °C (lit. 48–50 °C);[19] **^{1}H NMR (300 MHz, $CDCl_3$):** δ= 0.83–0.94 (3H, apparent t), 1.19–1.45 (18H, m), 1.72–1.86 (2H, m), 3.30–3.39 (2H, m), 5.29 (2H, s), 7.37 (5H, s); **^{13}C NMR (75.5 MHz, $CDCl_3$):** δ= 14.1, 22.6, 27.9, 28.9, 29.2, 29.3, 29.4, 29.52, 29.53, 31.8, 56.7, 67.8, 73.0, 128.4, 128.7, 128.8, 134.6, 160.0; **IR (neat):** 2124 (CN_2), 1720 (CO), 1330, 1288, 1217, 1142 (SO_2).

Methyl 2-((2'-cyclohexylethyl)sulfonyl)-2-diazoacetate[20] (24)

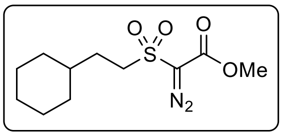

The title compound was prepared using the procedure described for methyl 2-diazo-2-((4'-phenylbutyl)sulfonyl)acetate **16**, using potassium carbonate (1.85 g, 13.4 mmol), methyl 2-((2-cyclohexylethyl)sulfonyl) acetate[20] (3.03 g, 12.2 mmol) in acetonitrile (30 mL) and *p*-ABSA (2.93 g, 12.2 mmol) in acetonitrile (30 mL). The mixture was stirred at 0 °C for 30 min, returned to room temperature and stirred overnight. Purification by column chromatography on silica gel, using ethyl acetate/hexane (20:80) as eluent, gave pure methyl 2-((2'-cyclohexylethyl)sulfonyl)-2-diazoacetate **24** as a yellow oil (2.65 g, 79%). Spectroscopic characteristics were consistent with previously reported data.[20] **^{1}H NMR (300 MHz, $CDCl_3$):** δ= 0.84–1.05 (2H, m), 1.06–1.46 (4H, m), 1.58–1.81 (7H, m), 3.34–3.46 (2H, symmetrical m), 3.88 (3H, s); **^{13}C NMR (75.5 MHz, $CDCl_3$):** δ= 25.9, 26.1, 29.5, 32.7, 36.4, 52.9, 54.6, 72.8, 160.4; **IR (neat):** 2124 (CN_2), 1713 (CO), 1331, 1294, 1144 (SO_2).

4.2 Rhodium Catalysed C–H Insertion Reactions – Thiopyran Dioxide synthesis

Table SI.3: Chiral rhodium(II) catalysis of α-diazo-β-oxosulfones **16**, **19–23**

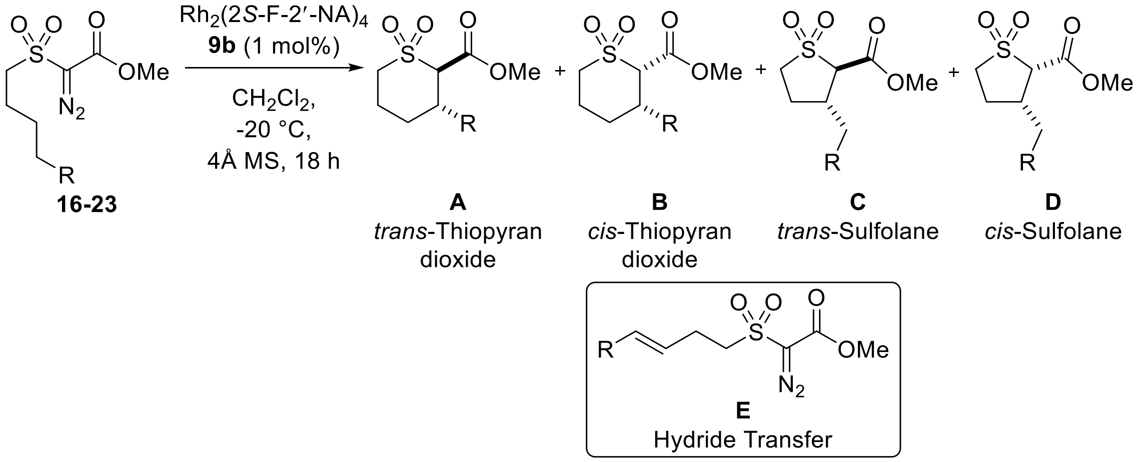

Entry[a]	Diazo	R	R[1]	d.r.[b] A:B	A % Yield[c] (% ee)[d]	B % Yield[c] (% ee)[d]	C & D % Yield[c,e,f]	E % Yield
					**17a**	**17b**	**18a&b**	**S22**
**1**	**16**	Ph	OMe	90:10	41 (92)[h]	5 (4)[i]	21	0
**2**[g]	**16**	Ph	OMe	92:8	64 (92)[h]	4 (6)[i]	23	1
					**25a**	**25b**	**S23a&b**	**S24**
**3**	**19**	*p*-MeC_6H_4	OMe	93:7	50 (89)[h]	4 (11)[j]	11	7
					**26a**	**26b**	**S25a&b**	**S26**
**4**	**20**	*p*-$OMeC_6H_4$	OMe	-[k]	42 (51)[h]	11 (9)[i]	13	8
					**27a**	**27b**	**S27a&b**	**S28**
**5**	**21**	*p*-FC_6H_4	OMe	89:11	53 (90)[h]	7 (6)[i]	12	1
					**28a**	**28b**	**S29a&b**	**-**
**6**[l]	**22**	Ph	Ph	-[k]	1 (69)[m]	2[n] (-)	25[n]	-
					**29a**	**29b**	**S30a&b**	**-**
**7**	**23**	C_8H_{17}	OBn	-[k]	31 (56)[h]	[o]	[o]	-

[a]The relative ratio of C–H insertion products could not be determined due to overlapping signals in the ^{1}H NMR spectra of the crude product mixtures. The spectra for the samples were recorded in both $CDCl_3$ and in C_6D_6 in an attempt to resolve signals. [b]The diastereomeric ratio was determined by integration of the methyl signals in the ^{1}H NMR spectra of the crude product mixtures in C_6D_6. [c]Isolated yield after column chromatography. [d]The enantiomeric excess was measured by chiral-phase HPLC analysis (for full details see **Table SI.6**).[e]The *trans*-sulfolane (**C**) and *cis*-sulfolane (**D**) were not isolated as pure compounds due to epimerisation of the *cis*-sulfolane (**D**) to the *trans*-sulfolane (**C**) during column chromatography on silica gel. The isolated yields of *trans*-sulfolane (**C**) are for samples which contain traces of the *cis*-sulfolane (**C**) (See Experimental for details). [f]Clear baseline resolution of chiral HPLC peaks was difficult to achieve for the mixed sulfolane samples and therefore enantiomeric excess was unable to be determined for the *trans*-sulfolane (**C**) and *cis*-sulfolane (**D**). [g]Reaction was conducted on a 1 gram scale (3.5 mmol). [h]The major enantiomer has a 2*R*,3*S* configuration. [i]The major enantiomer has a 2*S*,3*S* configuration. [j]The major enantiomer has a 2*R*,3*R* configuration. [k]Diastereomeric ratio was not determined due to overlapping signals in the ^{1}H NMR spectra of the crude product mixtures. [l]Reaction conducted under reflux. [m]Absolute configuaration undertermined. [n]Sample was not isolated pure. Conatianed unknown side-products. [o]**29b** and **S30a&b** were isolated in 42% yield as a combined mixture.

Table SI.4: Chiral rhodium(II) catalysis of α-diazo-β-oxosulfones **24**

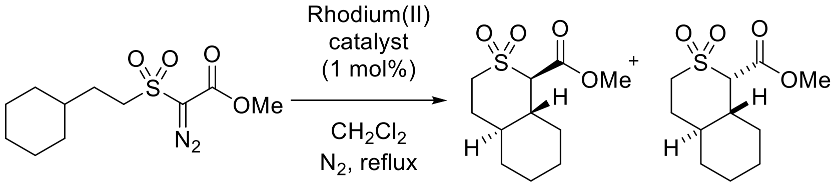

Entry	Rhodium catalyst	Solvent	Temp (°C)	Time (h)	d.r.[a] 30a:30b	30a % Yield[b] (% ee)[c]	30b % Yield[b] (% ee)[c]
			**No Molecular Sieves used**				
**1**	Rh_2(*S*-DOSP)$_4$	CH_2Cl_2	rt	0.5	96:4	64 (22)[d]	-
**2**[e]	Rh_2(5*R*-MEPY)$_4$	CH_2Cl_2	reflux	24	-	0	0
**3**	Rh_2(*S*-PTTL)$_4$	CH_2Cl_2	rt	0.5	98:2	80 (36)[d]	-
**4**	Rh_2(*S*-PTPA)$_4$	CH_2Cl_2	rt	0.5	94:6	65 (25)[d]	-
**5**	Rh_2(*S*-TCPTTL)$_4$	CH_2Cl_2	rt	0.5	97:3	81 (10)[d]	-
**6**	Rh_2(2*S*-F-2'-NA)$_4$	CH_2Cl_2	rt	2	94:6	67 (60)[d]	-
**7**	Rh_2(*S*-PTTL)$_4$	toluene	rt	0.5	97:3	63 (43)[d]	-
**8**	Rh_2(*S*-PTTL)$_4$	toluene	−20	2	97:3	62 (47)[d]	-
**9**	Rh_2(2*S*-F-2'-NA)$_4$	toluene	−20	2	91:9	52 (62)[d]	-
**10**	Rh_2(2*S*-F-2'-NA)$_4$	toluene	−70	2	91:9	56 (60)[d]	-
			**4Å Molecular Sieves used**				
**11**	Rh_2(2*S*-FBrPA)$_4$	CH_2Cl_2	−20	18	88:12	82 (53)[d]	12 (27)[f]
**12**	Rh_2(2*S*-MBrPA)$_4$	CH_2Cl_2	−20	18	92:8	88 (28)[d]	6 (13)[f]
**13**	Rh_2(2*S*-F-2'-NA)$_4$	CH_2Cl_2	−20	18	95:5	85 (84)[d]	-
**14**[g]	Rh_2(2*S*-F-2'-NA)$_4$	CH_2Cl_2	−20	18	96:4	91 (84)[d]	2 (~0)

[a]Diastereomeric ratio was calculated from ^{1}H NMR spectra of the crude product mixture. [b]Isolated yield after column chromatography. [c]The enantiomeric excess was measured by chiral-phase HPLC analysis (for full details see **Table SI.6**).[d]Second peak in the HPLC trace. [e]Only starting material (**24**) was evident in the ^{1}H NMR spectrum of the crude product mixture.[f]The major enantiomer has a (1*S*,4a*S*,8a*R*) configuration. [g]Reaction conducted on a 2 mmol scale compared to 0.35–0.85 mmol for **entry 1–13**.

4.2.1. Cyclisation of α-diazo-β-oxosulfone 16

Methyl (2*R*,3*S*)-3-phenyltetrahydro-2*H*-thiopyran-2-carboxylate 1,1-dioxide (17a)

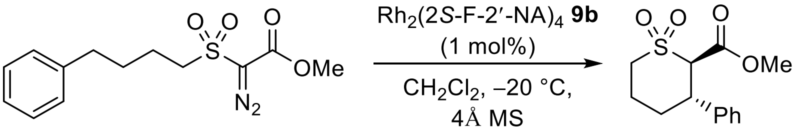

An oven dried 60 mL Schlenk tube (27 × 150 mm) containing 4Å molecular sieves (~ 600–800 mg) and a magnetic stir bar was flame dried and cooled under nitrogen. Rh_2(2*S*-F-2′-NA)$_4$ **9b** (53.1 mg, 34.2 μmol) was charged as a solid to the Schlenk tube. Methyl 2-diazo-2-(4'-phenylbutylsulfonyl)acetate **16** (1.01 g, 3.42 mmol) was charged into a dry 25 mL roundbottom flask. The Schlenk tube and roundbottom flask were both placed under vacuum and back filled with nitrogen 3 times. The Rh_2(2*S*-F-2′-NA)$_4$ **9b** was dissolved in deoxygenated dichloromethane (DCM, 5 ml), and the methyl 2-diazo-2-(4'-phenylbutylsulfonly)acetate **16** was dissolved in deoxygenated DCM (20 ml). The Schlenk tube containing the rhodium solution was placed into an ethanol cooling bath set at −20 °C while stirring. Once the ethanol bath had stabilised at −20 °C, the diazo **16** solution was added dropwise over 240 minutes via syringe to the Schlenk tube using a syringe pump. The reaction solution was stirred overnight at −20 °C before warming to room temperature. The reaction solution was filtered through a short pad of Celite® and activated charcoal, and concentrated under reduced pressure to give the crude product, which was then analysed by ^{1}H NMR spectroscopy in $CDCl_3$ and in C_6D_6. The ^{1}H NMR in $CDCl_3$ showed that compound **17a** was the major product, d at δ_H 4.16 (*J* 12.2), with minor compounds: **17b**, s at δ_H 3.53, **18a**, s at δ_H 3.74, **18b**, s at δ_H 3.83 and **S22**, d at δ_H 6.55 (*J* 15.8). The ^{1}H NMR analysed in C_6D_6 allowed the determination of the thiopyran diastereomeric ratio (92:8 dr, **17a**:**17b**, s at δ_H 3.06: s at δ_H 2.98) compared to overlapping peaks in the $CDCl_3$ ^{1}H NMR spectra. The crude product mixture, which was loaded using Celite®, was purified using column chromatography on silica gel, employing ethyl acetate/hexane (10:90 to 50:50) as eluent, and gave methyl (2*R*,3*S*)-3-phenyltetrahydro-2*H*-thiopyran-2-carboxylate 1,1-dioxide **17a** (589 mg, 64%), methyl (2*S*,3*S*)-3-phenyltetrahydro-2*H*-thiopyran-2-carboxylate 1,1-dioxide **17b** (41 mg, 4%), methyl (2*R**,3*R**)-3-benzyltetrahydrothiophene-2-carboxylate 1,1-dioxide **18a** (208 mg, 23%, of which contains ~15% **18b**, s at 3.83) and methyl 2-((4'-phenylbut-3'-en-1'-yl)sulfonyl)acetate **S22** (8 mg, 1%). **18a** or **18b** were not isolated as single pure compounds, but as a mixture. An enriched **18b** sample was obtained by taking the first few test tubes of the second least polar component, and an enriched **18a** sample was obtained by taking the last few test tubes of the third least polar component. **17a**, most polar component, white solid; **m.p.** 152–154 °C; **Spec. Rot.:** $[\alpha]_D^{20}$ −34.9 (*c* 1.0, CH_2Cl_2); **HPLC:** 92% ee (determined by chiral phase HPLC, see **Table SI.6** for HPLC conditions); **^{1}H NMR (300 MHz, $CDCl_3$):** δ= 1.64–1.81 (1H, m), 2.03–2.14 (1H, m), 2.14–2.40 (2H, m), 3.02–3.15 (1H, m), 3.26 (1H, dt, *J* 14.2, 3.6, 3.6), 3.58 (3H, s), 3.64 (1H, td, *J* 12.4, 12.4, 3.3), 4.16 (1H, d, *J* 12.2), 7.15–7.36 (5H, m); **^{1}H NMR (300 MHz, C_6D_6):** δ= 0.78–0.97 (1H, m), 1.04–1.16 (1H, m), 1.25–1.40 (1H, m), 1.64–1.82 (1H, m), 2.23 (1H, td, *J* 13.8, 13.8, 3.6), 2.43–2.53 (1H, m), 3.06 (3H, s), 3.57 (1H, td, *J* 12.4, 12.4, 3.4), 3.91 (1H, d, *J* 12.2), 6.87–7.12 (5H, m); **^{13}C NMR (75.5 MHz, $CDCl_3$):** δ= 23.2, 32.7, 45.5, 52.3, 53.1, 71.4, 127.2, 127.7, 129.0, 140.1, 163.1; **IR (neat):** 1732 (CO), 1320, 1293, 1142, 1124 (SO_2); **HRMS (ESI-TOF):** *m/z* $[M+H]^+$ calcd for $C_{13}H_{17}O_4S$, 269.0842, found 269.0845; **Elemental Analysis:** calcd (%) for $C_{13}H_{16}O_4S$: C, 58.19; H, 6.01, found: C, 58.41; H, 6.12.

Methyl (2*S*,3*S*)-3-phenyltetrahydro-2*H*-thiopyran-2-carboxylate 1,1-dioxide[19] 17b

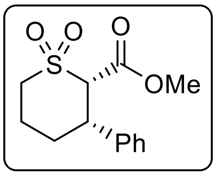

Methyl (2*S*,3*S*)-3-phenyltetrahydro-2*H*-thiopyran-2-carboxylate 1,1-dioxide **17b** (41 mg, 4%) was isolated as a white solid (least polar component). Spectroscopic characteristics were consistent with previously reported data.[19] **m.p.** 112–115 °C (lit 116–118 °C);[19b] **^{1}H NMR (300 MHz, $CDCl_3$):** δ= 1.86 (1H, dq, *J* 14.0, 3.3, 3.3, 3.3), 2.11–2.36 (2H, m,), 2.61 (1H, dq, *J* 13.3, 13.3, 13.3, 4.0), 3.04 (1H, dq, *J* 14.1, 3.3, 3.3, 3.3), 3.53 (3H, s), 3.61–3.75 (2H, m), 3.99 (1H, dd, *J* 4.5, 2.9), 7.15–7.21 (2H, m), 7.23–7.37 (3H, m); **^{1}H NMR (300 MHz, C_6D_6):** δ= 1.22 (1H, dq, *J* 13.8, 3.4, 3.4, 3.4), 1.30–1.43 (1H, m), 1.62–1.81 (1H, m), 2.23–2.47 (2H, m), 2.98 (3H, s), 3.30–3.46 (2H, m), 3.99 (1H, dd, *J* 4.5, 2.9), 6.76–6.85 (2H, m), 6.92–7.05 (3H, m); **^{13}C NMR (75.5 MHz, $CDCl_3$):** δ= 23.0, 23.7, 44.6, 47.9, 52.7, 70.5, 127.0, 127.9, 129.0, 139.6, 166.6; **IR (neat):** 1725 (CO), 1310, 1294, 1169, 1112 (SO_2); **HRMS (ESI-TOF):** *m*/*z* $[M+H]^+$ calcd for $C_{13}H_{17}O_4S$, 269.0842, found 269.0839.

Methyl (2*R,3*R**)-3-benzyltetrahydrothiophene-2-carboxylate 1,1-dioxide (18a)**

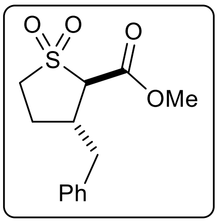

Methyl (2*R**,3*R**)-3-benzyltetrahydrothiophene-2-carboxylate 1,1-dioxide **18a** (208 mg, 23%, of which contains ~15% **18b**, s at 3.83) was isolated as a colourless oil (an enriched **18a** sample was obtained by taking the last few test tubes of the third least polar component); **^{1}H NMR (300 MHz, $CDCl_3$):** δ= 1.78–1.96 (1H, m), 2.21–2.34 (1H, m), 2.73 (1H, dd, *J* 13.5, 8.0), 2.91 (1H, dd, *J* 13.5, 6.4), 2.97–3.13 (2H, m), 3.27 (1H, qd, *J* 13.0, 7.2, 2.1), 3.66 (1H, d, *J* 9.8), 3.74 (3H, s), 7.09–7.19 (2H, m), 7.20–7.35 (3H, m); **^{1}H NMR (300 MHz, C_6D_6):** δ= 0.96–1.14 (1H, m), 1.21–1.40 (1H, m), 2.00 (1H, dd, *J* 13.5, 8.0), 2.18–2.31 (1H, m), 2.37–2.47 (2H, m), 2.64–2.80 (1H, m), 3.29 (3H, s), 3.46 (1H, d, *J* 9.9 Hz), 6.79–6.86 (2H, m), 6.97–7.10 (3H, m); **^{13}C NMR (75.5 MHz, $CDCl_3$):** δ= 26.2, 39.9, 41.6, 52.7, 53.3, 70.3, 127.0, 128.7, 129.1, 137.1, 165.5; **IR (neat):** 1739 (CO), 1315, 1268, 1168, 1119 (SO_2); **HRMS (ESI-TOF):** *m*/*z* $[M+H]^+$ calcd for $C_{13}H_{17}O_4S$, 269.0842, found 269.0841.

Methyl (2*S,3*R**)-3-benzyltetrahydrothiophene-2-carboxylate 1,1-dioxide (18b)**

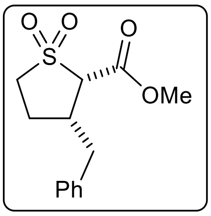

Methyl (2*S**,3*R**)-3-benzyltetrahydrothiophene-2-carboxylate 1,1-dioxide **18b** was isolated as a mixture with **18a** (an enriched **18b** sample was obtained by taking the first few test tubes of the second least polar component); characteristic peaks of **18b**: **^{1}H NMR (300 MHz, $CDCl_3$):** δ= 3.38–3.51 (1H, m), 3.80 (1H, d, *J* 6.3), 3.83 (3H, s).

Methyl 2-((4'-phenylbut-3'-en-1'-yl)sulfonyl)acetate (S22)

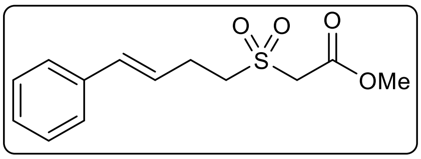

Methyl 2-((4'-phenylbut-3'-en-1'-yl)sulfonyl)acetate **S22** (8 mg, 1%) was isolated as a mixture with **18a**; colourless oil; **^{1}H NMR (600 MHz, $CDCl_3$):** δ= 2.77–2.85 (2H, m), 3.40–3.46 (2H, m), 3.83 (3H, s), 4.00 (2H, s), 6.19 (1H, dt, *J* 15.8, 7.0, 7.0), 6.55 (1H, d, *J* 15.8), 7.22–7.39 (5H, m); **^{1}H NMR (300 MHz, C_6D_6):** δ= 2.37–2.48 (2H, m), 2.89–2.98 (2H, m), 3.15 (3H, s), 3.36 (2H, s), 5.75 (1H, dt, *J* 15.8, 7.0), 6.14 (1H, d, *J* 15.8), 6.74–7.16 (5H, m); **^{13}C NMR (150.9 MHz, $CDCl_3$):** δ= 25.7, 53.0, 53.4, 57.7, 124.8, 126.2, 127.8, 128.7, 133.2, 136.5, 163.6; **IR (neat):** 1744 (CO), 1317, 1300, 1263, 1143, 1111 (SO_2); **HRMS (ESI-TOF):** *m*/*z* $[M+H]^+$ calcd for $C_{13}H_{17}O_4S$, 269.0842, found 269.0838.

4.2.2. Cyclisation of α-diazo-β-oxosulfone 19

Methyl (2*R*,3*S*)-3-(*p*-tolyl)tetrahydro-2*H*-thiopyran-2-carboxylate 1,1-dioxide (25a)

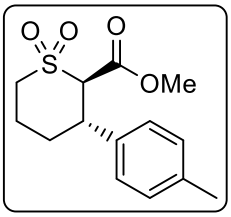

The title compound was prepared following the procedure described for methyl (2*R*,3*S*)-3-phenyltetrahydro-2*H*-thiopyran-2-carboxylate 1,1-dioxide **17a** using methyl 2-diazo-2-(4'-(4''-methylphenyl)butylsulfonyl)acetate **19** (113 mg, 0.36 mmol), Rh_2(2*S*-F-2′-NA)$_4$ **9b** (5.7 mg, 3.64 μmol) in deoxygenated DCM (15 mL). The diazo **19** solution was added dropwise over 120 minutes via syringe to the Schlenk tube using a syringe pump. The reaction solution was stirred overnight at −20 °C before warming to room temperature. The reaction solution was filtered through a short pad of Celite® and activated charcoal, and concentrated under reduced pressure to give the crude product, which was then analysed by ^{1}H NMR spectroscopy in $CDCl_3$ and in C_6D_6. The ^{1}H NMR in $CDCl_3$ showed that compound **25a** was the major product, d at δ_H 4.16 (*J* 12.2), with minor compounds: **25b**, s at δ_H 3.56, **S23a**, s at δ_H 3.76, **S23b**, s at δ_H 3.83 and **S24**, d at δ_H 6.51 (*J* 15.8). The ^{1}H NMR analysed in C_6D_6 allowed the determination of the thiopyran diastereomeric ratio (93:7 dr, **25a**:**25b**, s at δ_H 3.10: s at δ_H 3.02) compared to overlapping peaks in the $CDCl_3$ ^{1}H NMR spectra. The crude product mixture, which was loaded using Celite®, was purified using column chromatography on silica gel, employing ethyl acetate/hexane (10:90 to 30:70) as eluent, and gave methyl (2*R*,3*S*)-3-(*p*-tolyl)tetrahydro-2*H*-thiopyran-2-carboxylate 1,1-dioxide **25a** (51 mg, 50%), methyl (2*R*,3*R*) 3-(*p*-tolyl)tetrahydro-2*H*-thiopyran-2-carboxylate 1,1-dioxide **25b** (4 mg, 4%), methyl (2*R**,3*R**)-3-(4'-methylbenzyl)tetrahydrothiophene-2-carboxylate 1,1-dioxide **S23a** (11 mg, 11%, of which contains <10% **S23b**, s at 3.83), and methyl 2-((4''-methyphenyl)but-3'-en-1'-yl)sulfonyl)acetate **S24** (7 mg, 7%). **S23a** or **S23b** were not isolated as single pure compounds, but as a mixture. An enriched **S23b** sample was obtained by taking the first few test tubes of the second least polar component, and an enriched **S23a** sample was obtained by taking the last few test tubes of the third least polar component. **25a**, most polar component, white solid; **m.p.** 150–151 °C; **Spec. Rot.:** $[\alpha]_D^{20}$ −41.1 (*c* 1.0, CH_2Cl_2); **HPLC:** 89% ee (determined by chiral phase HPLC, see **Table SI.6** for HPLC conditions); **^{1}H NMR (300 MHz, $CDCl_3$):** δ= 1.62–1.80 (1H, m), 1.98–2.11 (1H, m), 2.12–2.38 (5H, m containing s at 2.31), 3.02–3.16 (1H, m), 3.24 (1H, dt, *J* 14.2), 3.53–3.66 (4H, m containing s at 3.58), 4.16 (1H, d, *J* 12.2), 7.04–7.15 (4H); **^{1}H NMR (300 MHz, C_6D_6):** δ= 0.94–1.11 (1H, m), 1.15–1.26 (1H, m), 1.36–1.46 (1H, m), 1.73–1.91 (1H, m), 2.04 (3H, s), 2.43 (1H, td, *J* 13.7, 3.6), 2.55–2.65 (1H, m), 3.10 (3H, s), 3.61 (1H, td, *J* 12.3, 3.3), 4.05 (1H, d, *J* 12.2), 6.91 (4H, s); **^{13}C NMR (75.5 MHz, $CDCl_3$):** δ= 21.0, 23.2, 32.7, 45.0, 52.2, 53.0, 71.4, 127.0, 129.5, 137.1, 137.3, 163.1; **IR (neat):** 1730 (CO), 1310, 1137, 1124 (SO_2); **HRMS (ESI-TOF):** *m*/*z* $[M+H]^+$ calcd for $C_{14}H_{19}O_4S$, 283.0999, found 283.0996; **Elemental Analysis:** calcd (%) for $C_{14}H_{18}O_4S$: C, 59.55; H, 6.43, found: C, 59.55; H, 6.36;

Methyl (2*R*,3*R*)-3-(*p*-tolyl)tetrahydro-2*H*-thiopyran-2-carboxylate 1,1-dioxide[19] (25b)

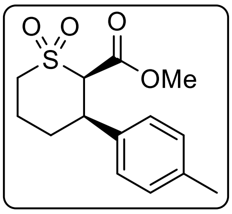

Methyl (2*R*,3*R*)-3-(*p*-tolyl)tetrahydro-2*H*-thiopyran-2-carboxylate 1,1-dioxide **25b** (4 mg, 4%) was isolated as a white solid (least polar component). Spectroscopic characteristics were consistent with previously reported data.[19] **^{1}H NMR (300 MHz, $CDCl_3$):** δ= 1.83 (1H, dq, *J* 14.0, 3.4), 2.10–2.36 (5H, m containing s at 2.32), 2.58 (1H, qd, *J* 13.4, 4.1), 3.03 (1H, dq, *J* 14.0, 3.3), 3.53–3.74 (5H, m containing s 3.56), 3.97 (1H, dd, *J* 4.5, 3.0), 7.06 (2H, d, *J* 8.2), 7.13 (2H, d, *J* 8.2); **^{1}H NMR (300 MHz, C_6D_6):** δ= 1.21–1.32 (1H, m), 1.33–1.45 (1H, m), 1.65–1.84 (1H, m), 2.01

(3H, s), 2.27–2.49 (2H, m), 3.02 (3H, s), 3.32–3.49 (2H, m), 4.02 (1H, dd, *J* 4.5, 2.9), 6.77 (2H d, *J* 8.2), 6.85 (2H, d, *J* 8.2); **^{13}C NMR (75.5 MHz, $CDCl_3$):** δ= 21.0, 23.1, 23.8, 44.3, 47.9, 52.7, 70.6, 126.9, 129.6, 136.6, 137.6, 166.7; **IR (neat):** 1736, 1310, 1173, 1120; **HRMS (ESI-TOF):** *m/z* $[M+Na]^+$ calcd for $C_{14}H_{18}O_4SNa$, 305.0818, found 305.0816.

Methyl (2*R,3*R**)-3-(4'-methylbenzyl)tetrahydrothiophene-2-carboxylate 1,1-dioxide (S23a)**

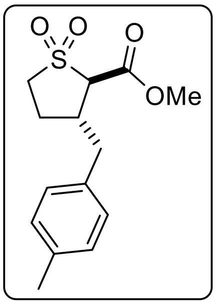

Methyl (2*R**,3*R**)-3-(4'-methylbenzyl)tetrahydrothiophene-2-carboxylate 1,1-dioxide **S23a** (11 mg, 11%, containing <10% **S23b**, s at 3.83) as a white solid (an enriched **S23a** sample was obtained by taking the last few test tubes of the third least polar component); **^{1}H NMR (300 MHz, $CDCl_3$):** δ= 1.76–1.95 (1H, m), 2.20–2.37 (4H, m containing s 2.32), 2.68 (1H, dd, *J* 13.5, 8.0), 2.88 (1H, dd, *J* 13.5, 6.3), 2.94–3.12 (2H, m), 3.26 (1H, qd, *J* 12.9, 7.2, 2.2), 3.65 (1H, d, *J* 9.8), 3.76 (3H, s, OC*H$_3$*), 7.03 (2H, d, *J* 8.1), 7.11 (2H, d, *J* 8.1); **^{1}H NMR (300 MHz, C_6D_6):** δ= 0.99–1.17 (1H, m), 1.26–1.42 (1H, m), 1.97–2.15 (4H, m containing s at 2.09), 2.20–2.33 (1H, m), 2.38–2.48 (2H, m), 2.66–2.83 (1H, m), 3.30 (3H, s), 3.48 (1H, d, *J* 9.8), 6.77 (2H, d, *J* 8.0), 6.91 (2H, d, *J* 8.0); **^{13}C NMR (75.5 MHz, $CDCl_3$):** δ= 21.0, 26.1, 39.4, 41.6, 52.7, 53.3, 70.3, 129.0, 129.4, 134.0, 136.6, 165.5; **IR (neat):** 1742, 1311, 1273, 1171, 1126, 1120; **HRMS (ESI-TOF):** *m/z* $[M+H]^+$ calcd for $C_{14}H_{19}O_4S$, 283.0999, found 283.1000.

Methyl (2*R,3*S**)-3-(4'-methylbenzyl)tetrahydrothiophene-2-carboxylate 1,1-dioxide (S23b)**

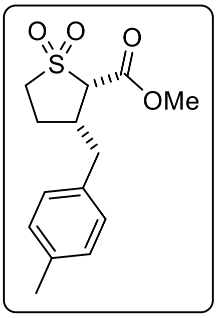

Methyl (2*R**,3*S**)-3-(4'-methylbenzyl)tetrahydrothiophene-2-carboxylate 1,1-dioxide **S23b** was isolated as a mixture with **S23a** (an enriched **S23b** sample was obtained by taking the first few test tubes of the second least polar component); characteristic peaks of **S23b**: **^{1}H NMR (300 MHz, $CDCl_3$):** δ= 3.38–3.50 (1H, m), 3.79 (1H, d, *J* 6.2), 3.83 (3H, s).

Methyl 2-((4'-(*p*-tolyl)but-3'-en-1'-yl)sulfonyl)acetate (S24)

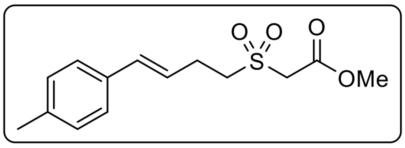

Methyl 2-((4'-(*p*-tolyl)but-3'-en-1'-yl)sulfonyl)acetate **S24** (7 mg, 7%) was isolated as a white solid; **m.p.** 91–93 °C; **^{1}H NMR (300 MHz, $CDCl_3$):** δ= 2.33 (3H, s), 2.72–2.84 (2H, m), 3.37–3.46 (2H, m), 3.82 (3H, s), 3.99 (2H, s), 6.12 (1H, dt, *J* 15.8, 7.0), 6.51 (1H, d, *J* 15.8), 7.12 (2H, d, *J* 8.1), 7.24 (2H, d, *J* 8.1); **^{1}H NMR (300 MHz, C_6D_6):** δ= 2.10 (3H, s), 2.40–2.53 (2H, m), 2.93–3.03 (2H, m), 3.18 (3H, s), 3.44 (2H, s), 5.76 (1H, dt, *J* 15.8, 7.0), 6.19 (1H, d, *J* 15.8), 6.96 (2H, d, *J* 8.0), 7.12 (2H, d, *J* 8.0); **^{13}C NMR (75.5 MHz, $CDCl_3$):** δ= 21.2, 25.7, 53.1, 53.4, 57.7, 123.7, 126.1, 129.3, 133.0, 133.8, 137.6, 163.6; **IR (neat):** 1753, 1745, 1300, 1260, 1144, 1110; **HRMS (ESI-TOF):** *m/z* $[M+H]^+$ calcd for $C_{14}H_{19}O_4S$, 283.0999, found 283.0997; **Elemental Analysis:** calcd (%) for $C_{14}H_{18}O_4S$: C, 59.55; H, 6.43, found: C, 59.30; H, 6.38.

4.2.3. Cyclisation of α-diazo-β-oxosulfone 20

Methyl (2*R*,3*S*)-3-(4'-methoxyphenyl)tetrahydro-2*H*-thiopyran-2-carboxylate 1,1-dioxide[19b] (26a)

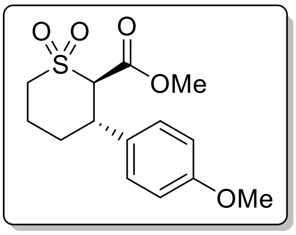

The title compound was prepared following the procedure described for methyl (2*R*,3*S*)-3-phenyltetrahydro-2*H*-thiopyran-2-carboxylate 1,1-dioxide **17a** using methyl 2-diazo-2-(4'-(4''-methoxyphenyl)butylsulfonyl)acetate **20** (147.5 mg, 0.45 mmol), Rh_2(2*S*-F-2′-NA)$_4$ **9b** (7.0 mg, 4.52 µmol) in deoxygenated DCM (15 mL). The diazo **20** solution was added dropwise over 120 minutes via syringe to the Schlenk tube using a syringe pump. The reaction solution was stirred overnight at −20 °C before warming to room temperature. The reaction solution was filtered through a short pad of Celite® and activated charcoal, and concentrated under reduced pressure to give the crude product, which was then analysed by ^{1}H NMR spectroscopy in $CDCl_3$ and in C_6D_6. The ^{1}H NMR in $CDCl_3$ showed that compound **26a** was the major product, d at δ_H 4.09 (*J* 12.1), with minor compounds: **26b**, s at δ_H 3.56, **S25b**, s at δ_H 3.83, and **S26**, s at δ_H 3.80, also evident. No evidence of **S25a**. Both the $CDCl_3$ and C_6D_6 ^{1}H NMR spectra contained overlapping peaks making it difficult to determine accurately the thiopyran diastereomeric ratio. The crude product mixture, which was loaded using Celite®, was purified using column chromatography on silica gel, employing ethyl acetate/hexane (20:80 to 40:60) as eluent, and gave methyl (2*R*,3*S*)-3-(4'-methoxyphenyl)tetrahydro-2*H*-thiopyran-2-carboxylate 1,1-dioxide **26a** (56 mg, 42%), methyl (2*S*,3*S*)-3-(4'-methoxyphenyl)tetrahydro-2*H*-thiopyran-2-carboxylate 1,1-dioxide **26b** (15 mg, 11%), methyl (2*R**,3*R**)-3-(4'-methoxybenzyl)tetrahydrothiophene-2-carboxylate 1,1-dioxide **S25a** (14 mg, 13%, of which contains ~20% **S25b**, s at 3.83), and methyl 2-(4'-(4''-methoxyphenyl)but-3'-en-1'-yl)sulfonyl)acetate **S26** (11 mg, 8%). **S25a** or **S25b** were not isolated as single pure compounds, but as a mixture. An enriched **S25b** sample was obtained by taking the first few test tubes of the second least polar component, and an enriched **S25a** sample was obtained by taking the last few test tubes of the third least polar component. **26a**, most polar component, white solid; **m.p.** 175–177 °C; **Spec. Rot.:** $[\alpha]_D^{20}$ −19.4 (*c* 1.0, CH_2Cl_2); **HPLC**: 51% ee (determined by chiral phase HPLC, see **Table SI.6** for HPLC conditions); **^{1}H NMR (300 MHz, $CDCl_3$):** δ= 1.60–1.79 (1H, m), 2.00–2.11 (1H, m), 2.12–2.38 (2H, m), 2.99–3.13 (1H, m), 3.24 (1H, dt, *J* 14.3, 3.6), 3.52–3.65 (4H, m containing s at 3.59), 3.78 (3H, s), 4.09 (1H, d, *J* 12.1), 6.80–6.88 (2H, m), 7.08–7.15 (2H, m); **^{13}C NMR (75.5 MHz, $CDCl_3$):** δ= 23.2, 32.7, 44.7, 52.2, 53.1, 55.2, 71.7, 114.3, 128.2, 132.1, 159.0, 163.1; **IR (neat):** 1726, 1325, 1257, 1140; **HRMS (ESI-TOF):** *m*/*z* $[M+H]^+$ calcd for $C_{14}H_{19}O_5S$, 299.0948, found 299.0946. **Elemental Analysis:** calcd (%) for $C_{14}H_{18}O_5S$: C, 56.36; H, 6.08. Found: C, 56.11; H, 5.96.

Methyl (2*S*,3*S*)-3-(4'-methoxyphenyl)tetrahydro-2*H*-thiopyran-2-carboxylate 1,1-dioxide[19] (26b)

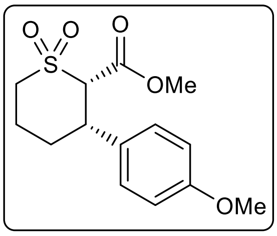

Methyl (2*S*,3*S*)-3-(4'-methoxyphenyl)tetrahydro-2*H*-thiopyran-2-carboxylate 1,1-dioxide **26b** (14.8 mg, 11%) was isolated as a white solid (least polar component). Spectroscopic characteristics were consistent with previously reported data.[19] **m.p.** 110–113 °C (lit. 90–92 °C of a 91% ee **26b** sample);[19b] **^{1}H NMR (300 MHz, $CDCl_3$):** δ= 1.82 (1H, dq, *J* 14.0, 3.4), 2.09–2.34 (2H, m), 2.57 (1H, qd, *J* 13.3, 4.0), 3.03 (1H, dq, *J* 14.0, 3.3), 3.52–3.73 (5H, m containing s at 3.56), 3.79 (3H, s), 3.96 (1H, dd, *J* 4.5, 2.9), 6.81–6.90 (2H, m), 7.06–7.14 (2H, m); **^{13}C NMR (75.5 MHz, $CDCl_3$):** δ= 23.0, 23.9, 43.8, 47.8, 52.7, 55.2, 70.6, 114.2, 128.0, 131.6,

159.0, 166.6; **IR (neat):** 1723, 1324, 1249, 1172, 1117; **HRMS (ESI-TOF):** *m/z* $[M+H]^+$ calcd for $C_{14}H_{19}O_5S$, 299.0948, found 299.0952.

Methyl (2*R,3*R**)-3-(4'-methoxybenzyl)tetrahydrothiophene-2-carboxylate 1,1-dioxide (S25a)**

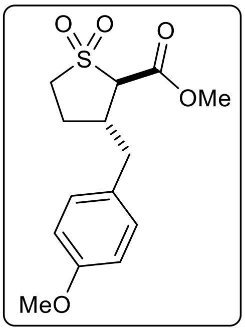

Methyl (2*R**,3*R**)-3-(4'-methoxybenzyl)tetrahydrothiophene-2-carboxylate 1,1-dioxide **S25a** (14 mg, 13%, of which contains ~20% **S25b,** s at 3.83) as a white solid (an enriched **S25a** sample was obtained by taking the last few test tubes of the third least polar component); **m.p.** 127–129 °C; **^{1}H NMR (300 MHz, CDCl$_3$):** δ= 1.77–1.94 (1H, m), 2.20–2.33 (1H, m), 2.68 (1H, dd, *J* 13.6, 7.9), 2.85 (1H, dd, *J* 13.6, 6.4), 2.93–3.13 (2H, m), 3.26 (1H, qd, *J* 13.0, 7.2, 2.2), 3.65 (1H, d, *J* 9.8), 3.76 (3H, s), 3.79 (3H, s), 6.81–6.87 (2H, m), 7.02–7.12 (2H, m); **^{13}C NMR (75.5 MHz, CDCl$_3$):** δ= 26.1, 39.0, 41.8, 52.7, 53.3, 55.3, 70.2, 114.1, 129.1, 130.1, 158.6, 165.5; **IR (neat):** 1742, 1311, 1300, 1245, 1171, 1121; **HRMS (ESI-TOF):** *m/z* $[M+H]^+$ calcd for $C_{14}H_{19}O_5S$, 299.0948, found 299.0944.

Methyl (2*R,3*S**)-3-(4'-methoxybenzyl)tetrahydrothiophene-2-carboxylate 1,1-dioxide (S25b)**

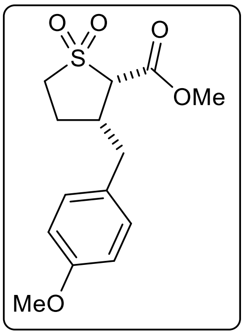

Methyl (2*R**,3*S**)-3-(4'-methoxybenzyl)tetrahydrothiophene-2-carboxylate 1,1-dioxide **S25b** was isolated as a mixture with **S25a** (an enriched **S25b** sample was obtained by taking the first few test tubes of the second least polar component); characteristic peaks of **S25b**: **^{1}H NMR (300 MHz, CDCl$_3$):** δ= 3.38–3.50 (1H, m), 3.83 (3H, s).

Methyl 2-((4'-(4''-methoxyphenyl)but-3'-en-1'-yl)sulfonyl)acetate (S26)

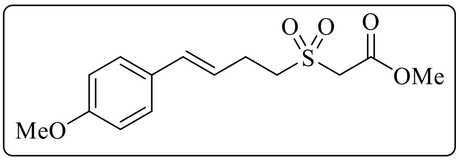

Methyl 2-((4'-(4''-methoxyphenyl)but-3'-en-1'-yl)sulfonyl)acetate **S26** (11 mg, 8%) was isolated as a colourless oil; **^{1}H NMR (300 MHz, CDCl$_3$):** δ= 2.72–2.83 (2H, m), 3.36–3.46 (2H, m), 3.80 (3H, s), 3.82 (3H, s), 3.99 (2H, s), 6.03 (1H, dt, *J* 15.8, 7.0), 6.48 (1H, d, *J* 15.8), 6.81–6.88 (2H, m), 7.24–7.32 (2H, m); **^{13}C NMR (75.5 MHz, CDCl$_3$):** δ= 25.7, 53.1, 53.3, 55.3, 57.7, 114.1, 122.5, 127.4, 129.4, 132.5, 159.3, 163.6; **IR (neat):** 1728, 1306, 1243, 1101; **HRMS (ESI-TOF):** *m/z* $[M+H]^+$ calcd for $C_{14}H_{19}O_5S$, 299.0948, found 299.0945.

4.2.4. Cyclisation of α-diazo-β-oxosulfone 21

Methyl (2*R*,3*S*)-3-(4'-fluorophenyl)tetrahydro-2*H*-thiopyran-2-carboxylate 1,1-dioxide (27a)

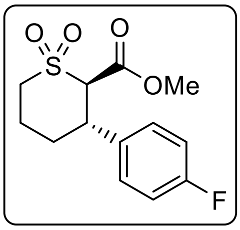

The title compound was prepared following the procedure described for methyl (2*R*,3*S*)-3-phenyltetrahydro-2*H*-thiopyran-2-carboxylate 1,1-dioxide **17a** using methyl 2-diazo-2-(4'-(4''-fluorophenyl)butylsulfonyl)acetate **21** (127.5 mg, 0.41 mmol), Rh_2(2*S*-F-2′-NA)$_4$ **9b** (6.3 mg, 4.06 μmol) in deoxygenated DCM (15 mL). The diazo **21** solution was added dropwise over 120 minutes via syringe to the Schlenk tube using a syringe pump. The reaction solution was stirred overnight at −20 °C before warming to room temperature. The reaction solution was filtered through a short pad of Celite® and activated charcoal, and concentrated under reduced pressure to give the crude product, which was then analysed by ^{1}H NMR spectroscopy in $CDCl_3$ and in C_6D_6. The ^{1}H NMR in $CDCl_3$ showed that compound **27a** was the major product, d at δ_H 4.15 (*J* 12.2), with minor compounds: **27b**, s at δ_H 3.56, **S27a**, s at δ_H 3.76, **S27b**, s at δ_H 3.83, and **S28**, dt at δ_H 6.10 (*J* 15.8, 7.0) also evident. The ^{1}H NMR analysed in C_6D_6 allowed the determination of the thiopyran diastereomeric ratio (89:11 dr, **27a**:**27b**) compared to overlapping peaks in the $CDCl_3$ ^{1}H NMR spectra. The crude product mixture, which was loaded using Celite®, was purified using column chromatography on silica gel, employing ethyl acetate/hexane (15:85 to 50:50) as eluent, and gave methyl (2*R*,3*S*)-3-(4'-fluorophenyl)tetrahydro-2*H*-thiopyran-2-carboxylate 1,1-dioxide **27a** (61 mg, 53%), methyl (2*S*,3*S*)-3-(4'-fluorophenyl)tetrahydro-2*H*-thiopyran-2-carboxylate 1,1-dioxide **27b** (8 mg, 7%), methyl (2*R**,3*R**)-3-(4'-fluorobenzyl)tetrahydrothiophene-2-carboxylate 1,1-dioxide **S27a** (14 mg, 12%, of which contains <10% **S27b**, s at 3.83), and methyl 2-(4'-(4''-fluorophenyl)but-3'-en-1'-yl)sulfonyl)acetate **S28** (1 mg, 1%). **S27a** or **S27b** were not isolated as single pure compounds, but as a mixture. An enriched **S27b** sample was obtained by taking the first few test tubes of the second least polar component, and an enriched **S27a** sample was obtained by taking the last few test tubes of the third least polar component. **27a**, most polar component, white solid; **m.p.** 154–155 °C; **Spec. Rot.:** $[\alpha]_D^{20}$ −31.1 (*c* 1.0, CH_2Cl_2); **HPLC:** 90% ee (determined by chiral phase HPLC, see **Table SI.6** for HPLC conditions); **^{1}H NMR (300 MHz, $CDCl_3$):** δ= 1.62–1.80 (1H, m), 2.00–2.11 (1H, m), 2.14–2.39 (2H, m), 3.04–3.18 (1H, m), 3.26 (1H, dt, *J* 14.2, 3.7), 3.56–3.70 (4H, m containing s at 3.59), 4.15 (1H, d, *J* 12.2), 6.95–7.06 (2H, m), 7.14–7.23 (2H, m); **^{1}H NMR (300 MHz, C_6D_6):** δ= 0.73–0.90 (1H, m), 1.07–1.30 (2H, m), 1.64–1.82 (1H, m), 2.31 (1H, td, *J* 13.8, 3.6), 2.47–2.58 (1H, m), 3.07 (3H, s), 3.48 (1H, td, *J* 12.4, 3.4), 3.83 (1H, d, *J* 12.1), 6.71 (4H, d, *J* 7.0); **^{19}F NMR (282.4 MHz, $CDCl_3$):** δ= −114.3 (1F, s); **^{13}C NMR (75.5 MHz, $CDCl_3$):** δ= 23.1, 32.6, 44.7, 52.1, 53.1, 71.4, 115.8 (d, $^2J_{CF}$ 21.5), 128.8 (d, $^3J_{CF}$ 8.2), 135.9 (d, $^4J_{CF}$ 3.3), 162.0 (d, $^1J_{CF}$ 247.0), 163.0; **IR (neat):** 1727, 1312, 1291, 1122; **HRMS (ESI-TOF):** *m*/*z* $[M+Na]^+$ calcd for $C_{13}H_{15}FO_4SNa$, 309.0567, found 309.0564; **Elemental Analysis:** calcd (%) for $C_{13}H_{15}FO_4S$: C, 54.53; H, 5.28, found: C, 54.60; H, 5.34.

Methyl (2*S*,3*S*)-3-(4'-fluorophenyl)tetrahydro-2*H*-thiopyran-2-carboxylate 1,1-dioxide[19a] (27b)

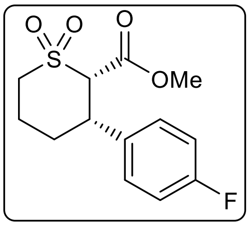

Methyl (2*S*,3*S*)-3-(4'-fluorophenyl)tetrahydro-2*H*-thiopyran-2-carboxylate 1,1-dioxide **27b** (8 mg, 7%) was isolated as a white solid (least polar fraction). Spectroscopic characteristics were consistent with previously reported data.[19a] **m.p.** 155–157 °C (lit 155–156 °C);[19a] **HPLC:** 6% ee (determined by chiral phase HPLC, see **Table SI.6** for HPLC conditions); **^{1}H NMR (300 MHz, $CDCl_3$):** δ= 1.84 (1H, dq, *J* 13.9, 3.4), 2.10–2.36 (2H, m), 2.58 (1H, qd, *J* 13.3, 4.1), 3.05 (1H, dq, *J* 14.0, 3.3), 3.56 (3H, s, OC*H*$_3$), 3.60–3.73 (2H, m), 3.96 (1H, dd, *J* 4.5, 2.9), 6.97–7.07 (2H, m), 7.12–7.21 (2H, m); **^{1}H NMR (300 MHz, C_6D_6):** δ= 1.11 (1H, dq, *J* 13.9, 3.4), 1.28–1.41 (1H, m), 1.59–1.78 (1H, m), 2.18 (1H, qd, *J* 13.5, 3.6), 2.40 (1H, dq, *J* 14.0, 3.3), 2.97 (3H, s), 3.21–3.39 (2H, m), 3.87 (1H, dd, *J* 4.5, 2.9), 6.51–6.69 (4H, m); **^{19}F NMR (282.4 MHz, $CDCl_3$):** δ= −114.1 (1F, s); **^{13}C NMR (75.5 MHz, $CDCl_3$):** δ= 23.0, 23.9, 43.8, 47.8, 52.8, 70.5, 115.9 (d, $^2J_{CF}$ 21.4), 128.6 (d, $^3J_{CF}$ 8.0), 135.4 (d, $^4J_{CF}$ 3.3), 162.2 (d, $^1J_{CF}$ 247.2), 166.5; **IR (neat):** 1735, 1309, 1220, 1173, 1118; **HRMS (ESI-TOF):** *m/z* [M+Na]$^+$ calcd for $C_{13}H_{15}FO_4SNa$, 309.0567, found 309.0562.

Methyl (2*R,3*R**)-3-(4'-fluorobenzyl)tetrahydrothiophene-2-carboxylate 1,1-dioxide (S27a)**

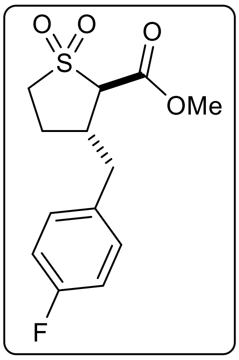

Methyl (2*R**,3*R**)-3-(4'-fluorobenzyl)tetrahydrothiophene-2-carboxylate 1,1-dioxide **S27a** (14 mg, 12%, of which contains <10% **S27b**, s at 3.83) was isolated as an opaque oil (an enriched **S27a** sample was obtained by taking the last few test tubes of the third least polar component); **^{1}H NMR (300 MHz, $CDCl_3$):** δ= 1.76–1.95 (1H, m), 2.20–2.33 (1H, m), 2.71 (1H, dd, *J* 13.6, 8.0), 2.89 (1H, dd, *J* 13.6, 6.5), 2.95–3.13 (2H, m), 3.28 (1H, qd, *J* 13.0, 7.1, 2.1), 3.65 (1H, d, *J* 9.8), 3.76 (3H, s), 6.95–7.05 (2H, m), 7.08–7.17 (2H, m); **^{1}H NMR (300 MHz, C_6D_6):** δ= 0.89–1.08 (1H, m), 1.14–1.27 (1H, m), 1.87 (1H, dd, *J* 13.4, 8.5), 2.19–2.33 (2H, m), 2.42 (1H, qd, *J* 12.9, 7.2, 2.3), 2.53–2.69 (1H, m), 3.29 (3H, s), 3.39 (1H, d, *J* 9.9), 6.51–6.60 (2H, m), 6.66–6.77 (2H, m); **^{19}F NMR (282.4 MHz, $CDCl_3$):** δ= −115.6 (1F, s); **^{13}C NMR (75.5 MHz, $CDCl_3$):** δ= 26.2, 39.2, 41.6, 52.6, 53.4, 70.2, 115.6 (d, $^2J_{CF}$ 21.3), 130.5 (d, $^3J_{CF}$ 8.0), 132.9 (d, $^4J_{CF}$ 3.3), 161.9 (d, $^1J_{CF}$ 245.4), 165.4; **IR (neat):** 1741, 1317, 1266, 1219, 1158, 1120; **HRMS (ESI-TOF):** *m/z* [M+Na]$^+$ calcd for $C_{13}H_{15}FO_4SNa$, 309.0567, found 309.0564.

Methyl (2*R,3*S**)-3-(4'-fluorobenzyl)tetrahydrothiophene-2-carboxylate 1,1-dioxide (S27b)**

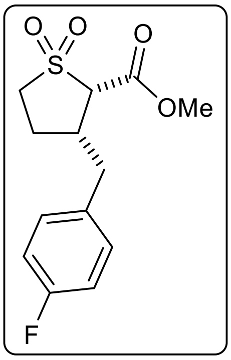

Methyl (2*R**,3*S**)-3-(4'-fluorobenzyl)tetrahydrothiophene-2-carboxylate 1,1-dioxide **S27b** was isolated as a mixture with **S27a** (an enriched **S27b** sample was obtained by taking the first few test tubes of the second least polar component); characteristic peaks of **S27b**: **^{1}H NMR (300 MHz, $CDCl_3$):** 3.39–3.50 (1H, m), 3.83 (3H, s, C*H*$_3$).

Methyl 2-((4'-(4''-fluorophenyl)but-3'-en-1'-yl)sulfonyl)acetate (S28)

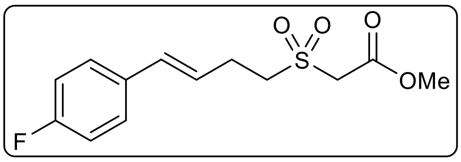

Methyl 2-(4'-(4''-fluorophenyl)but-3'-en-1'-yl)sulfonyl)acetate **S28**; colourless oil, second most polar fraction; **^{1}H NMR (300 MHz, $CDCl_3$):** δ= 2.74–2.85 (2H, m), 3.38–3.47 (2H, m), 3.83 (3H, s), 3.99 (2H, s), 6.10 (1H, dt, *J* 15.8, 7.0), 6.50 (1H, d, *J* 15.8), 6.95–7.05 (2H, m), 7.24–7.36 (2H, m); **^{1}H NMR (300 MHz, C_6D_6):** δ= 2.35–2.46 (2H, m), 2.89–2.98 (2H, m), 3.15 (3H, s), 3.35 (2H, s), 5.57 (1H, dt, *J* 15.8, 7.0), 5.99 (1H, d, *J* 15.7), 6.69–6.80 (2H, m), 6.83–6.93 (2H, m); **^{19}F NMR (282.4 MHz, $CDCl_3$):** δ= −114.2 (1F, s); **^{13}C NMR (75.5 MHz, $CDCl_3$):** δ= 25.6, 53.0, 53.4, 57.7, 115.6 (d, $^2J_{CF}$ 21.7), 124.6 (d, $^6J_{CF}$ 2.3), 127.8 (d, $^3J_{CF}$ 8.0), 132.0, 132.7 (d, $^4J_{CF}$ 3.3), 162.4 (d, $^1J_{CF}$ 246.7), 163.6; **IR (neat):** 1744, 1313, 1259, 1227, 1110; **HRMS (ESI-TOF):** *m/z* $[M+Na]^+$ calcd for $C_{13}H_{15}FO_4SNa$, 309.0567, found 309.0567.

4.2.5. Cyclisation of α-diazo-β-oxosulfone 22

((2*R,3*S**)-1,1-Dioxido-3-phenyltetrahydro-2*H*-thiopyran-2-yl)(phenyl)methanone (28a)**

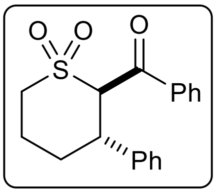

An oven dried 60 mL Schlenk tube (27 × 150 mm) containing 4Å molecular sieves (~600–800 mg) and a magnetic stir bar was flame dried and cooled under nitrogen. Rh_2(2*S*-F-2'-NA)$_4$ **9b** (10.8 mg, 6.92 μmol) was charged as a solid to the Schlenk tube. 2-Diazo-1-phenyl-2-((4'-phenylbutyl)sulfonyl)ethan-1-one **22** (237 mg, 0.69 mmol) was charged into a dry 25 mL roundbottom flask. The Schlenk tube and roundbottom flask were both placed under vacuum and back filled with nitrogen 3 times. The Rh_2(2*S*-F-2'-NA)$_4$ **9b** was dissolved in deoxygenated DCM (5 ml), and the 2-diazo-1-phenyl-2-((4'-phenylbutyl)sulfonyl)ethan-1-one **22** was dissolved in deoxygenated DCM (10 ml). The Schlenk tube containing the rhodium solution was placed into an oil bath set at 55 °C. Once the rhodium solution began to reflux, the diazo solution **22** was added dropwise over 120 minutes via syringe to the Schlenk tube using a syringe pump. The reaction solution was stirred overnight while heating under reflux before cooling to room temperature. The reaction solution was filtered through a short pad of Celite® and activated charcoal, and concentrated under reduced pressure to give the crude product, which was then analysed by ^{1}H NMR spectroscopy in $CDCl_3$. The ^{1}H NMR in $CDCl_3$ showed that compound **S29** was the major product, d at δ_H 4.62 (*J* 9.7) and d at δ_H 4.77 (*J* 6.6), with minor compound **28a**, d at δ_H 5.26. The crude product mixture, which was loaded using Celite®, was purified using column chromatography on silica gel, employing ethyl acetate/hexane (10:90 to 40:60) as eluent, and gave ((2*R**,3*S**)-1,1-dioxido-3-phenyltetrahydro-2*H*-thiopyran-2-yl)(phenyl)methanone **28a** (3 mg, 1%), ((2*R**,3*R**)-1,1-dioxido-3-phenyltetrahydro-2*H*-thiopyran-2-yl)(phenyl)methanone **28b** (4 mg, 2%), ((2*R**,3*R**)-3-benzyl-1,1-dioxidotetrahydrothiophen-2-yl)(phenyl) methanone **S29a** (52 mg, 25% of which contains ~5% **S29b**, d at 4.77). **28a**, most polar fraction, white solid; **m.p.** 244–245 °C; **HPLC:** 69% ee (determined by chiral phase HPLC, see **Table SI.6** for HPLC conditions); **Spec. Rot.:** $[\alpha]_D^{20}$ +25.50 (*c* 0.5, CH_2Cl_2); **^{1}H NMR (600 MHz, $CDCl_3$):** δ=1.77–1.89 (1H, m), 2.09–2.19 (1H, m), 2.20–2.29 (1H, m), 2.31–2.43 (1H, m), 3.21 (1H, td, *J* 14.0, 3.8), 3.32 (1H, dt, *J* 14.2, 3.5), 3.95 (1H, td, *J* 12.4, 3.3), 5.26 (1H, d, *J* 11.7), 7.09–7.22 (5H, m), 7.36–7.42 (2H, m), 7.49–7.55 (1H, m), 7.82–7.87 (2H, m); **^{13}C NMR (150.9 MHz, $CDCl_3$):** δ= 23.3, 33.0, 45.5, 53.0, 70.9, 127.3, 127.4, 128.6, 128.86, 128.88, 133.8, 137.7, 140.5, 189.1; **IR (neat):** 1671, 1322, 1291, 1139; **HRMS (ESI-TOF):** *m/z* $[M+H]^+$ calcd for $C_{18}H_{19}O_3S$, 315.1049, found 315.1052.

((2*R,3*R**)-1,1-Dioxido-3-phenyltetrahydro-2H-thiopyran-2-yl)(phenyl)methanone**[19] **(28b)**

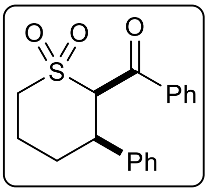

((2*R**,3*R**)-1,1-dioxido-3-phenyltetrahydro-2*H*-thiopyran-2-yl)(phenyl)methanone **28b** (4 mg, 2%) was isolated as an impure colourless oil (least polar fraction). Spectroscopic characteristics were consistent with previously reported data.[19] **^{1}H NMR (400 MHz, $CDCl_3$):** δ= 1.88 (1H, dq, *J* 14.0, 3.3), 2.20–2.42 (2H, m), 2.72 (1H, dq, *J* 13.5, 4.0), 3.09 (1H, dq, *J* 13.9, 3.2), 3.79–3.90 (2H, m), 5.07 (1H, dd, *J* 4.5, 2.8), 7.06–7.18 (5H, m), 7.20–7.30 (2H, m), 7.36–7.46 (3H, m); **IR (neat):** 1668, 1324, 1297, 1245, 1125; **HRMS (ESI-TOF):** *m*/*z* $[M+H]^+$ calcd for $C_{18}H_{19}O_3S$, 315.1049, found 315.1053.

((2*R,3*R**)-3-Benzyl-1,1-dioxidotetrahydrothiophen-2-yl)(phenyl)methanone (S29a)**

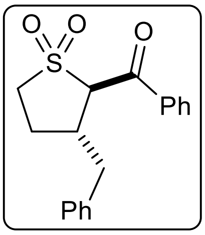

((2*R**,3*R**)-3-Benzyl-1,1-dioxidotetrahydrothiophen-2-yl)(phenyl)methanone **S29a** (52 mg, 25% of which contains ~5% **S29b**, d at 4.77) was isolated as a white solid; **m.p.** 111–113 °C; **^{1}H NMR (400 MHz, $CDCl_3$):** δ= 1.91–2.06 (1H, m), 2.27–2.39 (1H, m), 2.72 (1H, dd, *J* 13.4, 8.2), 2.88 (1H, dd, *J* 13.4, 6.1), 3.07–3.19 (1H, m), 3.28–3.48 (2H, m), 4.61 (1H, d, *J* 9.7), 7.08–7.29 (5H, m), 7.45–7.55 (2H, m), 7.58–7.66 (1H, m), 7.92–8.01 (2H, m); **^{13}C NMR (100.6 MHz, $CDCl_3$):** δ= 26.2, 40.0, 41.5, 53.5, 71.0, 127.0, 128.7, 128.9, 129.0, 129.1, 134.3, 136.5, 137.4, 189.8; **IR (neat):** 1688, 1303, 1267, 1121; **HRMS (ESI-TOF):** *m*/*z* $[M+H]^+$ calcd for $C_{18}H_{19}O_3S$, 315.1049, found 315.1049.

*Only trace amount of the cis-sulfolane **S29b** was evident from a d at δ_H 4.77 (J 6.6)*

4.2.6. Cyclisation of α-diazo-β-oxosulfone 23

Benzyl (2*R*,3*R*)-3-octyltetrahydro-2*H*-thiopyran-2-carboxylate 1,1-dioxide[19] **(29a)**

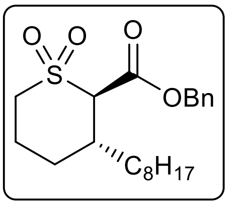

The title compound was prepared following the procedure described for methyl (2*R*,3*S*)-3-phenyltetrahydro-2*H*-thiopyran-2-carboxylate 1,1-dioxide **17a** using benzyl 2-diazo-2-(dodecylsulfonyl)acetate **23** (161 mg, 0.39 mmol), Rh_2(2*S*-F-2′-NA)$_4$ **9b** (6.1 mg, 3.94 μmol) in deoxygenated DCM (15 mL). The diazo **23** solution was added dropwise over 120 minutes via syringe to the Schlenk tube using a syringe pump. The reaction solution was stirred overnight at −20 °C before warming to room temperature. The reaction solution was filtered through a short pad of Celite® and activated charcoal, and concentrated under reduced pressure to give the crude product, which was then analysed by ^{1}H NMR spectroscopy in $CDCl_3$ and in C_6D_6. The ^{1}H NMR in $CDCl_3$ showed that compound **29a**, d at δ_H 3.70 (*J* 10.5), and **S30b**, m at 3.37–3.50, were the major products, with the minor compound, **29b**, m at δ_H 3.52–3.64, also evident. There was no evidence of **S30a**. Both the $CDCl_3$ and C_6D_6 ^{1}H NMR spectra contained overlapping peaks making it difficult to determine accurately the thiopyran diastereomeric ratio. The crude product mixture, which was loaded using Celite®, was purified using column chromatography on silica gel, employing ethyl acetate/hexane (5:95 to 20:80) as eluent, and gave benzyl (2*R**,3*R**)-3-octyltetrahydro-2*H*-thiopyran-2-carboxylate 1,1-dioxide **29a** (46 mg, 31%), benzyl (2*R*,3*S*)-3-octyltetrahydro-2*H*-thiopyran-2-carboxylate 1,1-dioxide **29b** (20 mg, 13%), and benzyl (2*R**,3*R**)-3-nonyltetrahydrothiophene-2-carboxylate 1,1-dioxide **S30a** (44 mg, 29%, of

which contains ~32% **S30b**, m at 3.37–3.50). **S30a** or **S30b** were not isolated as single pure compounds, but as a mixture. An enriched **S30b** sample was obtained by taking the first few test tubes of the second least polar component, and an enriched **S30a** sample was obtained by taking the last few test tubes of the second most polar component. **29a**, most polar component, white solid. Spectroscopic characteristics were consistent with previously reported data.[19] **m.p.** 85–87 °C (lit. 86–88 °C);[19b] **Spec. Rot.:** $[\alpha]_D^{20}$ −14.3 (*c* 1.0, CH_2Cl_2); **HPLC:** 56% ee (determined by chiral phase HPLC, see **Table SI.6** for HPLC conditions); **^{1}H NMR (300 MHz, $CDCl_3$):** δ= 0.83–0.93 (3H, m), 1.07–1.38 (15H, m), 1.97–2.18 (3H, m), 2.34–2.50 (1H, m), 2.83–3.00 (1H, m), 3.19 (1H, dt, *J* 14.1, 4.2), 3.70 (1H, d, *J* 10.5), 5.27 (2H, s), 7.28–7.43 (5H, m); **^{13}C NMR (75.5 MHz, $CDCl_3$):** δ= 14.0, 22.4, 22.6, 26.2, 28.3, 29.1, 29.30, 29.34, 31.8, 33.3, 38.9, 51.9, 67.9, 71.3, 128.4, 128.48, 128.54, 134.9, 164.0; **IR (neat):**1727, 1302, 1129; **HRMS (ESI-TOF):** *m*/*z* $[M+Na]^+$ calcd for $C_{21}H_{32}O_4SNa$, 403.1914, found 403.1919; **Elemental Analysis:** calcd (%) for $C_{21}H_{32}O_4S$: C, 66.28; H, 8.48. Found: C, 66.28; H, 8.41.

Benzyl (2*R*,3*S*)-3-octyltetrahydro-2H-thiopyran-2-carboxylate 1,1-dioxide[19] (29b)

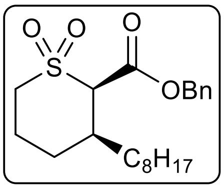

Benzyl (2*R*,3*S*)-3-octyltetrahydro-2H-thiopyran-2-carboxylate 1,1-dioxide **29b** (20 mg, 13%) was isolated as a colourless oil (least polar fraction). Spectroscopic characteristics were consistent with previously reported data.[19] **HPLC:** 53% ee (determined by chiral phase HPLC); **Spec. Rot.:** $[\alpha]_D^{20}$ −11.2 (*c* 1.0, CH_2Cl_2); **^{1}H NMR (300 MHz, $CDCl_3$):** δ= 0.83–0.93 (3H, m), 1.09–1.35 (14H, m), 1.62 (1H, dq, *J* 14.1, 3.6), 1.70–1.86 (1H, m), 1.96–2.18 (2H, m), 2.27–2.42 (1H, m), 2.94 (1H, dq, *J* 13.9, 3.3), 3.52–3.64 (1H, m), 3.90 (1H, dd, *J* 4.5, 2.9), 5.14 (1H, d, *J* 12.1), 5.31 (1H, d, *J* 12.1), 7.36 (5H, br s); **^{13}C NMR (75.5 MHz, $CDCl_3$):** δ= 14.1, 22.6, 23.0, 25.0, 26.5, 29.2, 29.3, 31.8, 34.1, 39.6, 48.2, 67.8, 68.5, 128.5, 128.7, 134.8, 166.1; **IR (neat):** 1727, 1322, 1172, 1118; **HRMS (ESI-TOF):** *m*/*z* $[M+H]^+$ calcd for $C_{21}H_{33}O_4S$, 381.2094, found 381.2094.

Benzyl (2*R,3*R**)-3-nonyltetrahydrothiophene-2-carboxylate 1,1-dioxide (S30a)**

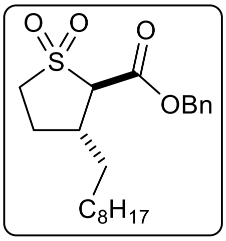

Benzyl (2*R**,3*R**)-3-nonyltetrahydrothiophene-2-carboxylate 1,1-dioxide **S30a** (44 mg, 29%, of which contains ~32% **S30b**, m at 3.36–3.50) was isolated as a colourless oil (an enriched **S30a** sample was obtained by taking the last few test tubes of the second most polar component); **^{1}H NMR (300 MHz, $CDCl_3$):** δ= 0.82–0.94 (3H, m), 1.15–1.35 (14H, m), 1.38–1.61 (2H, m), 1.71–1.90 (1H, m), 2.30–2.43 (1H, m), 2.70–2.86 (1H, m), 3.09 (1H, td, *J* 12.8, 6.9), 3.26 (1H, qd, *J* 12.9, 6.9, 1.9), 3.61 (1H, d, *J* 9.4), 5.23 (1H, d, *J* 12.3), 5.30 (1H, d, *J* 12.3), 7.29–7.43 (5H, m); **^{13}C NMR (75.5 MHz, $CDCl_3$):** δ= 14.1, 22.7, 26.6, 26.8, 29.2, 29.4, 29.5, 31.8, 34.4, 40.2, 52.7, 68.3, 71.0, 128.4, 128.55, 128.6, 134.9, 165.5; **IR (neat):** 1740, 1308, 1267, 1179, 1114; **HRMS (ESI-TOF):** *m*/*z* $[M+H]^+$ calcd for $C_{21}H_{33}O_4S$, 381.2094, found 381.2092.

Benzyl (2*R,3*S**)-3-nonyltetrahydrothiophene-2-carboxylate 1,1-dioxide (S30b)**

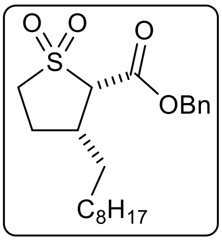

Benzyl (2*R**,3*S**)-3-nonyltetrahydrothiophene-2-carboxylate 1,1-dioxide **S30b** was isolated as a mixture with **S30a** (an enriched **S30b** sample was obtained by taking the first few test tubes of the second least polar component); characteristic peaks of **S30b**: **^{1}H NMR (300 MHz, $CDCl_3$):** δ= 3.37–3.50 (1H, m); **^{13}C NMR (75.5 MHz, $CDCl_3$):** δ= 27.6, 31.5, 40.0, 51.5, 67.7, 67.8, 165.2.

4.2.7. Cyclisation of α-diazo-β-oxosulfone 24

Methyl (1*R*,4a*S*,8a*R*)-octahydro-1*H*-isothiochromene-1-carboxylate 2,2-dioxide[20] (30a)

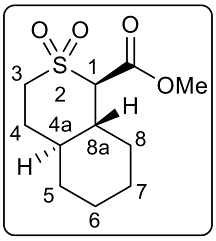

The title compound was prepared following the procedure described for methyl (2*R*,3*S*)-3-phenyltetrahydro-2*H*-thiopyran-2-carboxylate 1,1-dioxide **17a** using methyl 2-((2'-cyclohexylethyl)sulfonyl)-2-diazoacetate **24** (534 mg, 1.95 mmol), Rh_2(2*S*-F-2′-NA)$_4$ **9b** (30.3 mg, 19.5 µmol) in deoxygenated DCM (15 mL). The diazo **24** solution was added dropwise over 120 minutes via syringe to the Schlenk tube using a syringe pump. The reaction solution was stirred overnight at −20 °C before warming to room temperature. The reaction solution was filtered through a short pad of Celite® and activated charcoal, and concentrated under reduced pressure to give the crude product, which was then analysed by ^{1}H NMR spectroscopy in $CDCl_3$. The ^{1}H NMR in $CDCl_3$ showed very efficient C–H insertion in the synthesis of **30**, with a thiopyran diastereomeric ratio (96:4 dr, **30a**:**30b**, s at δ_H 3.85: m at δ_H 3.58–3.77). The crude product mixture, which was loaded using Celite®, was purified using column chromatography on silica gel, employing ethyl acetate/hexane (30:70) as eluent, and gave methyl (1*R*,4a*S*,8a*R*)-octahydro-1*H*-isothiochromene-1-carboxylate 2,2-dioxide **30a** (436 mg, 91%), and methyl (1*R**,4a*R**,8a*S**)-octahydro-1*H*-isothiochromene-1-carboxylate 2,2-dioxide **30b** (10 mg, 2%); **30a**, most polar component, white solid; **m.p.** 163–164 °C (lit. 163–164 °C);[20] **Spec. Rot.:** $[\alpha]_D^{20}$ −22.6 (*c* 1.0, CH_2Cl_2); **HPLC:** 84% ee (determined by chiral phase HPLC, see **Table SI.6** for HPLC conditions); **^{1}H NMR (300 MHz, $CDCl_3$):** δ= 0.96–1.39 (5H, m), 1.58–1.86 (4H, m), 1.90–2.03 (2H, m), 2.13 (1H, qd, *J* 11.7, 3.2), 2.93–3.22 (2H, m), 3.63 (1H, d, *J* 11.7), 3.85 (3H, s); **^{13}C NMR (75.5 MHz, $CDCl_3$):** δ= 24.9, 25.6, 30.75, 30.83, 32.7, 40.5, 42.9, 52.4, 53.2, 71.2, 164.0; **IR (neat):** 1733, 1291, 1127; **HRMS (ESI-TOF):** *m/z* [M+H]$^+$ calcd for $C_{11}H_{19}O_4S$ 247.0993, found 247.0995. **Elemental Analysis:** calcd (%) for $C_{11}H_{18}O_4S$: C, 53.64; H, 7.37. Found: C, 53.55; H, 7.25.

Methyl (1*R,4a*R**,8a*S**)-octahydro-1H-isothiochromene-1-carboxylate 2,2-dioxide[20] (30b)**

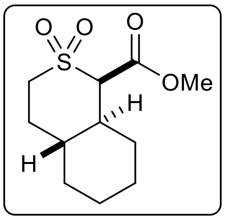

Methyl (1*R**,4a*R**,8a*S**)-octahydro-1*H*-isothiochromene-1-carboxylate 2,2-dioxide **30b** (10 mg, 2%) was isolated as a white solid (least polar component); **HPLC:** 0% ee (determined by chiral phase HPLC, see **Table SI.6** for HPLC conditions); **^{1}H NMR (300 MHz, $CDCl_3$):** δ= 0.92–1.11 (2H, m), 1.14–1.36 (2H, m), 1.62–2.14 (8H, m), 2.96 (1H, dq, *J* 14.0, 3.2), 3.58–3.77 (2H, m containing dd, *J* 4.6, 3.1), 3.80 (3H, s); **^{13}C NMR (75.5 MHz, $CDCl_3$):** δ= 25.4, 25.5, 30.9, 31.1, 32.9, 33.6, 43.0, 48.5, 52.8, 68.9, 166.9; **IR (neat):** 1731,1315, 1288, 1229, 1167, 1109; **HRMS**

(ESI-TOF): *m*/*z* $[M+H]^+$ calcd for $C_{11}H_{19}O_4S$ 247.1004; found 247.1008; **Elemental Analysis:** calcd (%) for $C_{11}H_{18}O_4S$: C, 53.64; H, 7.37. Found: C, 53.46; H, 7.37.

5. Synthesis of α-diazoacetamides

Procedure B

A 100 ml three-neck round-bottom flask was charged with water (10 ml), dichloromethane (5 ml) and sodium azide (5.5 eq.). The flask was cooled to 0 °C, after which trifluoromethanesulfonyl anhydride (1.1 eq.) was slowly added via syringe over 15 minutes while vigorously stirring the solution. The resulting solution was vigorously stirred at 0 °C for two hours after which the layers were separated. The aqueous layer was extracted with dichloromethane (5 ml) and the combined organic layer was washed with aqueous sodium carbonate (10 ml, 10%) and dried with magnesium sulfate. The dried solution of triflyl azide was added slowly to a solution of the relevant *α-cyanoacetamide* (1.0 eq) and triethylamine (1.1 eq.) in dichloromethane (30 ml for 10 mmol scale of α-cyanoacetamide) while stirring at 0 °C. The solution was allowed to slowly reach room temperature and reaction progression was monitored by IR spectroscopy. After complete disappearance of the azide stretch (~2100 cm^{-1}), the solution was carefully concentrated under reduced pressure. The desired α-cyano-α-diazoacetamide was afforded following purification by flash chromatography on silica gel using hexane:ethyl acetate (80:20) as eluent.[21]

Note: The resonance for the diazo carbon is a weak signal in the ^{13}C NMR spectra and often is not observed for the derivatives across the series.

***N*,*N*-Dibenzyl-2-cyano-2-diazoacetamide[21] (31)**

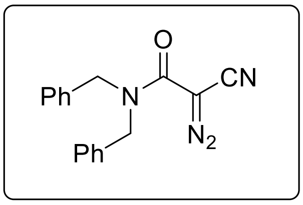

This title compound was prepared according to ***Procedure B*** from *N,N*-(dibenzyl)-2-cyanoacetamide (2.84 g, 10.80 mmol), sodium azide (6.60 g, 100 mmol), trifluoromethanesulfonic anhydride (1.9 mL, 11.2 mmol) and triethylamine (1.70 mL, 12.0 mmol). The crude product was purified by flash chromatography on silica gel with hexane:ethyl acetate as eluent. Spectroscopic characteristics were consistent with previously reported data.[21] Yellow oil, 2.05 g (66%); **^{1}H NMR (400 MHz, $CDCl_3$):** δ= 4.59 (4H, s), 7.19–7.38 (10H, m); **^{13}C NMR (100.6 MHz, $CDCl_3$):** δ= 50.3, 109.7, 127.7, 128.1, 128.9, 135.5, 159.9; **IR (neat):** 2214, 2117, 1626.

***N*-(*tert*-Butyl)-2-cyano-2-diazo-*N*-(4'-fluorobenzyl)acetamide[21] (33)**

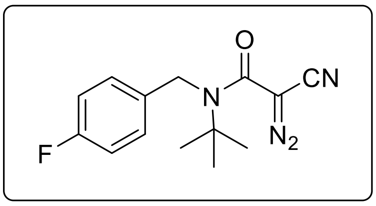

This title compound was prepared according to ***Procedure B*** from *N*-(*tert*-butyl)-2-cyano-*N*-(4′-fluorobenzyl)acetamide (3.12 g, 12.5 mmol), sodium azide (5.581 g, 85.80 mmol), trifluoromethanesulfonic anhydride (2.30 mL, 14.0 mmol) and triethylamine (1.80 mL, 13.0 mmol). The crude product was purified by flash chromatography on silica gel with hexane:ethyl acetate as eluent. Spectroscopic characteristics were consistent with previously reported data.[21] Yellow crystals, 2.22 g (65%); **m.p.** 105–107 °C; **^{1}H NMR (400 MHz, $CDCl_3$):** δ= 1.42 (9H, s), 4.67 (2H, s), 7.01–7.11 (2H, m), 7.13–7.22 (2H, m); **^{19}F NMR (376.5 MHz, $CDCl_3$):** δ= –114.9; **^{13}C NMR (100.6 MHz, $CDCl_3$):** δ= 28.5, 49.1, 60.4, 109.8, 115.7 ($^{2}J_{CF}$ 21.7), 127.6 ($^{3}J_{CF}$ 8.1), 134.1 ($^{4}J_{CF}$ 3.2), 160.9, 162.0 ($^{1}J_{CF}$ 246.0); **IR (neat):** 2213 (CN), 2121 (CN_2) 1635 (CO).

N-(tert-Butyl)-2-cyano-2-diazo-N-(4'-bromobenzyl)acetamide[21] (34)

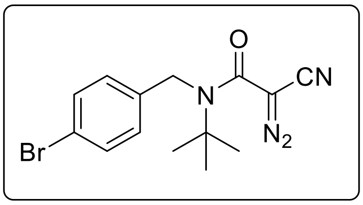

This title compound was prepared according to ***Procedure B*** from *N*-(*tert*-butyl)-2-cyano-*N*-(4′-bromobenzyl)acetamide (2.07 g, 6.700 mmol), sodium azide (2.60 g, 40.0 mmol), trifluoromethanesulfonic anhydride (1.2 mL, 7.0 mmol) and triethylamine (0.98 mL, 7.00 mmol). The crude product was purified by flash chromatography on silica gel with hexane:ethyl acetate as eluent. Spectroscopic characteristics were consistent with previously reported data.[21] Yellow crystals, 2.03 g (90%); **m.p.** 109–111 °C (Lit.,[21] 109–110 °C); **^{1}H NMR (400 MHz, $CDCl_3$):** δ= 1.41 (9H, s), 4.65 (2H, s), 7.10 (2H, d, *J* 8.4), 7.50 (2H, d, *J* 8.4); **^{13}C NMR (100.6 MHz, $CDCl_3$):** δ= 28.5, 49.3, 54.8, 60.4, 109.7, 121.3, 127.7, 131.9, 137.6, 160.9; **IR (neat):** 2213, 2119, 1634.

N-(tert-Butyl)-2-cyano-2-diazo-N-(4'-chlorobenzyl)acetamide[21] (35)

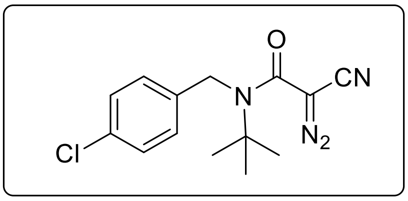

This compound was prepared according to ***Procedure B*** from *N*-(*tert*-butyl)-2-cyano-*N*-(4′-chlorobenzyl)acetamide (2.50 g, 9.40 mmol), sodium azide (5.37 g, 83.0 mmol), trifluoromethanesulfonic anhydride (1.63 mL, 10.0 mmol) and triethylamine (1.35 mL, 10.0 mmol). The crude product was purified by flash chromatography on silica gel with hexane:ethyl acetate as eluent. Spectroscopic characteristics were consistent with previously reported data.[21] Yellow crystals, 1.76 g (64%); **m.p.** 108–110 °C (Lit.,[21] 109–113 °C); **^{1}H NMR (400 MHz, $CDCl_3$):** δ= 1.41 (9H, s), 4.67 (2H, s), 7.16 (2H, d, *J* 8.4), 7.34 (2H, d, *J* 8.4); **^{13}C NMR (100.6 MHz, $CDCl_3$):** δ= 28.5, 49.2, 54.8, 60.4, 109.7, 127.4, 128.9, 133.2, 137.1, 160.9; **IR (neat):** 2213, 2118, 1634.

N-(tert-Butyl)-2-cyano-2-diazo-N-(2',6'-dichlorobenzyl)acetamide (36)

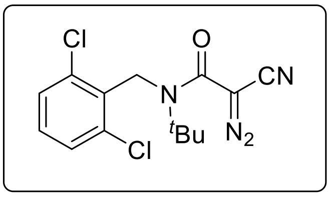

This title compound was prepared according to ***Procedure B*** from *N*-(*tert*-butyl)-2-cyano-*N*-(2′,6′-dichlorobenzyl)acetamide (2.41 g, 8.10 mmol), sodium azide (4.00 g, 62.0 mmol), trifluoromethanesulfonic anhydride (1.41 mL, 8.50 mmol) and triethylamine (0.85 mL, 8.50 mmol). The crude product was purified by flash chromatography on silica gel with hexane:ethyl acetate as eluent. Yellow crystals, 2.50 g (94%); **m.p.** 109–110 °C; **^{1}H NMR (400 MHz, $CDCl_3$):** δ= 1.33 (9H, s), 4.92 (2H, s), 7.17–7.24 (1H, m), 7.34 (2H, d, *J* 8.0); **^{13}C NMR (100.6 MHz, $CDCl_3$):** δ= 28.3, 48.1, 59.9, 110.0, 129.5, 129.6, 132.3, 135.8, 162.9; **IR (neat):** 2214, 2118, 1642; **HRMS (ESI-TOF):** *m*/*z* $[M+Na]^+$ calcd for $C_{14}H_{14}{}^{35}Cl_2N_4ONa$ 347.0437, found 347.0439.

N-(tert-Butyl)-2-cyano-2-diazo-N-(4'-methylbenzyl)acetamide[21] (37)

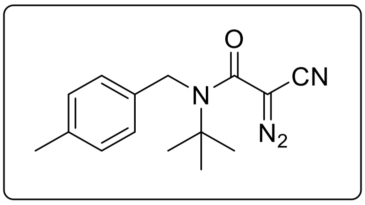

This title compound was prepared according to ***Procedure B*** from *N*-(*tert*-butyl)-2-cyano-*N*-(4′-methylbenzyl)acetamide (1.72 g, 7.00 mmol), sodium azide (2.60 g, 40.0 mmol), trifluoromethanesulfonic anhydride (1.3 mL, 7.2 mmol) and triethylamine (1.00 mL, 7.20 mmol). The crude product was purified by flash chromatography on silica gel with hexane:ethyl acetate as eluent. Spectroscopic characteristics were consistent with previously reported data.[21] Yellow crystals, 1.56 g (83%); **m.p.** 86–88 °C

(Lit.,[21] 85–86 °C); **^{1}H NMR (400 MHz, $CDCl_3$):** δ= 1.42 (9H, s), 2.34 (3H, s), 4.67 (2H, s), 7.09 (2H, d, *J* 8.1), 7.17 (2H, d, *J* 8.0); **^{13}C NMR (100.6 MHz, $CDCl_3$):** δ= 21.1, 28.5, 49.5, 54.7, 60.3, 109.9, 125.9, 129.4, 135.3, 137.1, 160.9; **IR (neat):** 2213, 2117, 1634.

***N*-(*tert*-Butyl)-2-cyano-2-diazo-*N*-(3',5'-dimethylbenzyl)acetamide (38)**

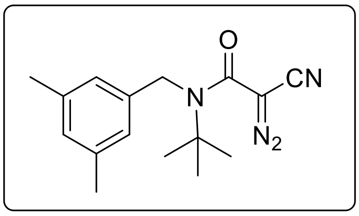

This title compound was prepared according to ***Procedure B*** from *N*-(*tert*-butyl)-2-cyano-*N*-(3′,5′-dimethylbenzyl) acetamide (2.78 g, 10.8 mmol), sodium azide (6.00 g, 92.0 mmol), trifluoromethanesulfonic anhydride (1.90 mL, 11.2 mmol) and triethylamine (1.60 mL, 11.2 mmol). Purified by flash chromatography on silica gel with hexane:ethyl acetate as eluent. Yellow crystals, 1.79 g (58%); **m.p.** 105–107 °C; **^{1}H NMR (400 MHz, $CDCl_3$):** δ= 1.42 (9H, s), 2.31 (6H, s), 4.64 (2H, s), 6.79 (2H, s), 6.90 (1H, s); **^{13}C NMR (100.6 MHz, $CDCl_3$):** δ= 21.4, 28.5, 49.7, 60.3, 109.9, 123.8, 129.1, 138.2, 138.3, 160.8; **IR (neat):** 2213, 2119, 1634; **HRMS (ESI-TOF):** *m*/*z* $[M+Na]^+$ calcd for $C_{16}H_{20}N_4ONa$ 307.1529, found 307.1533.

***N*-(*tert*-Butyl)-2-cyano-2-diazo-*N*-(2',4',6'-trimethylbenzyl)acetamide (39)**

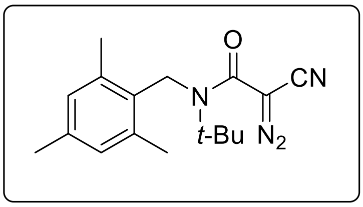

This title compound was prepared according to ***Procedure B*** from *N*-(*tert*-butyl)-2-cyano-*N*-(2′,4′,6′-trimethylbenzyl) acetamide (1.78 g, 6.50 mmol), sodium azide (3.60 g, 55.0 mmol), trifluoromethanesulfonic anhydride (1.10 mL, 6.80 mmol) and triethylamine (0.95 mL, 6.80 mmol). The crude product was purified by flash chromatography on silica gel with hexane:ethyl acetate as eluent. Yellow crystals, 1.18 g (61%); **m.p.** 91–92 °C; **^{1}H NMR (400 MHz, $CDCl_3$):** δ= 1.31 (9H, s), 2.26 (3H, s), 2.32 (6H, s), 4.64 (2H, s), 6.82 (2H, s); **^{13}C NMR (100.6 MHz, $CDCl_3$):** δ= 20.7, 20.8, 28.2, 47.8, 59.9, 109.8, 130.3, 131.0, 136.8, 137.2, 163.1; **IR (neat):** 2215, 2119, 1635; **HRMS (ESI-TOF):** *m*/*z* $[M+Na]^+$ calcd for $C_{17}H_{22}N_4ONa$ 321.1686, found 321.1685.

***N*-Benzyl-*N*-(*tert*-butyl)-2-cyano-2-diazoacetamide[21] (40)**

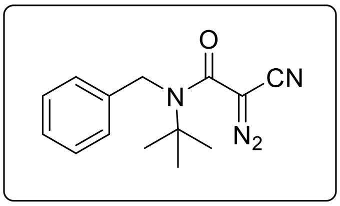

This title compound was prepared according to ***Procedure B*** from *N*-(*tert*-butyl)-2-cyano-*N*-(benzyl)acetamide (2.04 g, 8.90 mmol), sodium azide (3.65 g, 60.80 mmol), trifluoromethanesulfonic anhydride (1.5 mL, 9.5 mmol) and triethylamine (1.3 mL, 9.5 mmol). The crude product was purified by flash chromatography on silica gel with hexane:ethyl acetate as eluent. Spectroscopic characteristics were consistent with previously reported data.[21] Yellow crystals, 1.65 g (72%); **m.p.** 88–92 °C (Lit.,[21] 89–91 °C); **^{1}H NMR (400 MHz, $CDCl_3$):** δ= 1.43 (9H, s), 4.71 (2H, s), 7.18–7.42 (5H, m); **^{13}C NMR (100.6 MHz, $CDCl_3$):** δ= 28.5, 49.7, 60.3, 109.8, 126.0, 127.5, 128.7, 138.4, 160.9; **IR (neat):** 2214, 2121, 1634.

***N*-(*tert*-Butyl)-2-cyano-2-diazo-*N*-(4'-nitrobenzyl)acetamide[21] (41)**

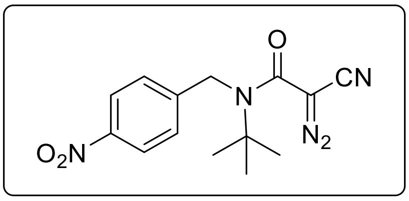

This title compound was prepared according to ***Procedure B*** from *N*-(*tert*-butyl)-2-cyano-*N*-(4′-nitrobenzyl)acetamide (2.89 g, 10.5 mmol), sodium azide (6.00 g, 92.0 mmol), trifluoromethanesulfonic anhydride (1.85 mL, 11.0 mmol) and triethylamine (1.50 mL, 11.0 mmol). This crude product was purified by flash chromatography on silica gel with hexane:ethyl acetate as eluent. Spectroscopic characteristics were consistent with previously reported data.[21] Yellow crystals, 1.570 g (50%); **m.p.** 125–127 °C (Lit.,[21] 122–124 °C); **^{1}H NMR (400 MHz, CDCl$_3$):** δ= 1.43 (9H, s), 4.80 (2H, s), 7.43 (2H, d, *J* 8.8), 8.23–8.28 (2H, m); **^{13}C NMR (100.6 MHz, CDCl$_3$):** δ= 28.4, 49.4, 54.8, 60.6, 109.6, 124.0, 126.8, 146.3, 147.3, 161.1; **IR (neat):** 2214, 2124, 1519, 1342.

***N*-(*tert*-Butyl)-2-cyano-2-diazo-*N*-(4'-carbomethoxybenzyl)acetamide[21] (42)**

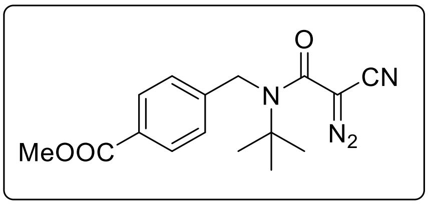

This title compound was prepared according to ***Procedure B*** from *N*-(*tert*-butyl)-2-cyano-*N*-(4′-carbomethoxy benzyl)acetamide (0.79 g, 2.70 mmol), sodium azide (1.17 g, 18.0 mmol), trifluoromethanesulfonic anhydride (0.50 mL, 3.0 mmol) and triethylamine (0.43 mL, 3.0 mmol). The crude product was purified by flash chromatography on silica gel with hexane:ethyl acetate as eluent. Spectroscopic characteristics were consistent with previously reported data.[21] Yellow crystals, 1.86 g (81%); **m.p.** 88–91 °C (Lit.,[21] 90–93 °C); **^{1}H NMR (400 MHz, CDCl$_3$):** δ= 1.42 (9H, s), 3.92 (3H, s), 4.75 (2H, s), 7.31 (2H, d, *J* 8.2), 8.05 (2H, d, *J* 8.3); **^{13}C NMR (100.6 MHz, CDCl$_3$):** δ= 28.5, 49.7, 52.2, 60.5, 109.6, 126.0, 129.4, 130.1, 143.8, 161.0, 166.8; **IR (neat):** 2214, 2125, 1719, 1636.

***N*-(*tert*-Butyl)-2-cyano-2-diazo-*N*-(4'-trifluoromethylbenzyl)acetamide[21] (43)**

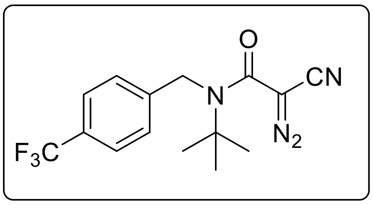

This title compound was prepared according to ***Procedure B*** from *N*-(*tert*-butyl)-2-cyano-*N*-(4′-trifluoromethylbenzyl) acetamide (2.00 g, 6.70 mmol), sodium azide (2.60 g, 40.8 mmol), trifluoromethanesulfonic anhydride (1.2 mL, 7.0 mmol) and triethylamine (0.98 mL, 7.00 mmol). The crude product was purified by flash chromatography on silica gel with hexane:ethyl acetate as eluent. Spectroscopic characteristics were consistent with previously reported data.[21] Yellow crystals, 1.95 g (90%); **m.p.** 68–69 °C (Lit.,[21] 65–67 °C); **^{1}H NMR (400 MHz, CDCl$_3$):** δ= 1.43 (9H, s), 4.76 (2H, s), 7.35 (2H, d, *J* 8.3), 7.64 (2H, d, *J* 8.3); **^{19}F NMR (376.5 MHz, CDCl$_3$):** δ= –62.5; **^{13}C NMR (100.6 MHz, CDCl$_3$):** δ= 28.5, 49.4, 60.5, 109.6, 124.0 (q, $^{1}J_{CF}$ 272.0), 125.8 (q, $^{3}J_{CF}$ 3.8), 126.3, 129.8 ($^{2}J_{CF}$ 32.6), 142.7, 161.0; **IR (neat):** 2214, 2122, 1640.

***N*-(*tert*-Butyl)-2-cyano-2-diazo-*N*-(4'-cyanobenzyl)acetamide (44)**

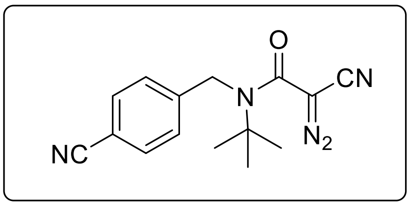

This title compound was prepared according to ***Procedure B*** from *N*-(*tert*-butyl)-2-cyano-*N*-(4′-cyanobenzyl) acetamide (3.19 g, 12.5 mmol), sodium azide (5.58 g, 85.80 mmol), trifluoromethanesulfonic anhydride (2.30 mL, 14.0 mmol) and triethylamine (1.80 mL, 13.0 mmol). The crude product was purified by flash chromatography on silica gel with hexane:ethyl acetate as eluent. Yellow crystals, 1.99 g (57%); **m.p.** 148–150 °C; **^{1}H NMR (400 MHz, $CDCl_3$):** δ= 1.42 (9H, s), 4.75 (2H, s), 7.36 (2H, d, *J* 7.8), 7.69 (2H, d, *J* 8.1); **^{13}C NMR (100.6 MHz, $CDCl_3$):** δ= 28.5, 49.5, 60.6, 109.6, 111.5, 118.5, 126.7, 132.6, 144.2, 161.1; **IR (neat):** 2228, 2214, 2123, 1638; **HRMS (ESI-TOF):** *m*/*z* $[M+H]^+$ calcd for $C_{15}H_{16}N_5O$ 304.1169, found 304.1172.

***N*-(*tert*-Butyl)-2-cyano-2-diazo-*N*-(4'-methoxybenzyl)acetamide[21] (45)**

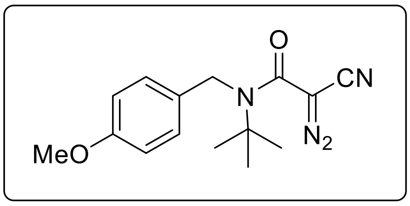

This title compound was prepared according to ***Procedure B*** from *N*-(*tert*-butyl)-2-cyano-*N*-(4′-methoxybenzyl) acetamide (3.27 g, 12.50 mmol), sodium azide (5.58 g, 85.8 mmol), trifluoromethanesulfonic anhydride (2.20 mL, 13.0 mmol) and triethylamine (1.82 mL, 13.0 mmol). The crude product was purified by flash chromatography on silica gel with hexane:ethyl acetate as eluent. Spectroscopic characteristics were consistent with previously reported data.[21] Yellow crystals, 2.72 g (76%); **m.p.** 108–111 °C; **^{1}H NMR (400 MHz, $CDCl_3$):** δ= 1.41 (9H, s), 3.81 (3H, s), 4.65 (2H, s), 6.90 (2H, d, *J* 8.6), 7.12 (2H, d, *J* 8.5); **^{13}C NMR (100.6 MHz, $CDCl_3$):** δ= 28.5, 49.2, 55.3, 60.2, 109.9, 114.1, 127.2, 130.2, 158.9, 160.8; **IR (neat):** 2213, 2120, 1633.

6. Aromatic Addition

Table SI.5: Aromatic addition catalyst screen of α-cyano-α-diazoacetamide **31**

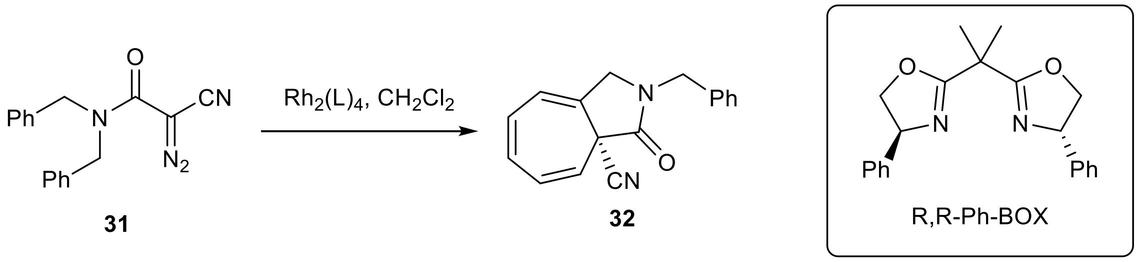

Entry	Catalyst[a]	Time (h)	32 Yield (%)[b]	32 % ee[c]
**1**[d]	$Rh_2(OAc)_4$	1.0	90	-
**2**	Rh_2(2*S*-F-2'-NA)$_4$ **9b**	1.5	85	67
**3**	Rh_2(2*S*-MBrPA)$_4$ **9h**	1.5	84	53
**4**	Rh_2(2*S*-M-2'-NA)$_4$ **9g**	1.5	87	51
**5**	Rh_2(2*S*-FBrPA)$_4$ **9d**	1.5	88	59
**6**	Rh_2(2*S*-FOMePA)$_4$ **9e**	1.5	90	73
**7**[d,e]	Rh_2(2*S*-FOMePA)$_4$ **9e**	12.0	75	60
**8**	Rh_2(2*S*-F-1'-NA)$_4$ **9c**	1.5	87	62
**9**	Rh_2(*S*-DOSP)$_4$ **2**	1.5	80	17
**10**	Rh_2(*S*-PTTL)$_4$ **3**	1.5	85	11[f]
**11**	Rh_2(*S*-TCPTTL)$_4$ **4**	1.5	80	14
**12**	Rh_2(*S*-TFPTTL)$_4$	1.5	82	32[f]
**13**	$CuPF_6$–((*R*,*R*)-Ph-BOX)	12	88	36

[a] 1.0 mol% of catalyst.[b] Purified by flash chromatography. [c] Enantiomeric excess determined by chiral HPLC analysis; dextrorotatory (+), 1*S* enantiomer predominates in each case, unless otherwise stated. [d] Reaction performed at room temperature. [e] Toluene used as solvent. [f] Levorotatory (–), 1*R* enantiomer

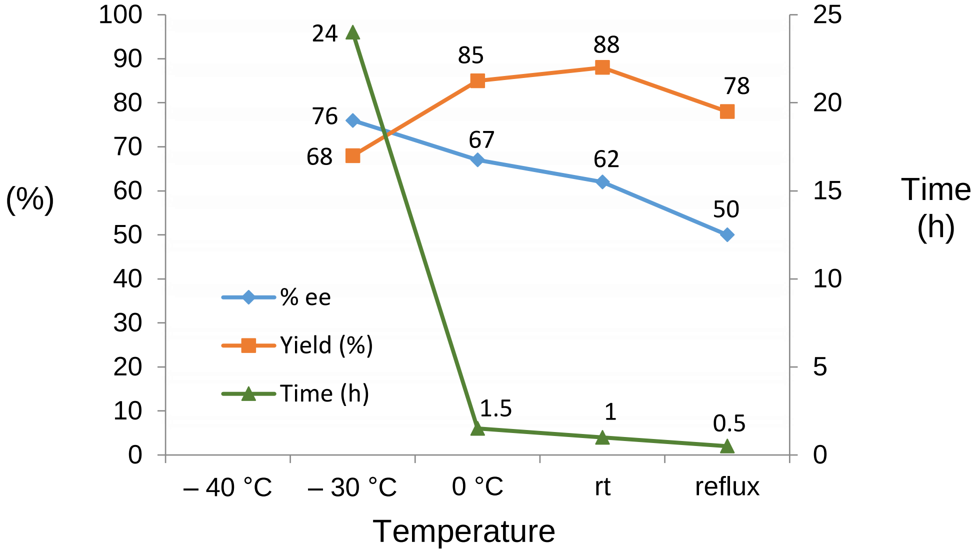

Figure SI.2: Graph of time, yield and enantiopurity versus temperature for the Rh_2(2S-F-2'-NA)$_4$ **9b** catalysed transformation of α-cyano-α-diazoacetamide **31.**

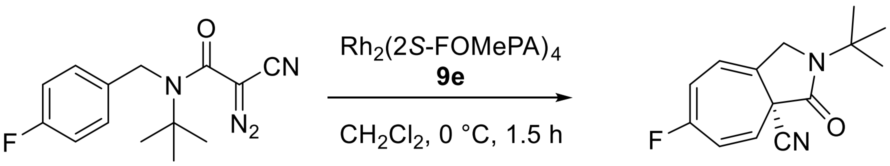

33 **46**

1.20 mol%[Rh] 83%, 87% ee
0.60 mol%[Rh] 83%, 87% ee
0.20 mol%[Rh] 85%, 87% ee
0.05 mol%[Rh] 91%, 88% ee

Scheme SI.2: Impact of catalyst loading on the aromaticaddition of affording α-cyano-α-diazoacetamide **33**.

Table SI.6: Rhodium acetate catalyzed aromatic addition reactions of α-cyano-α-diazoacetamides **31**, **33–45** [a]

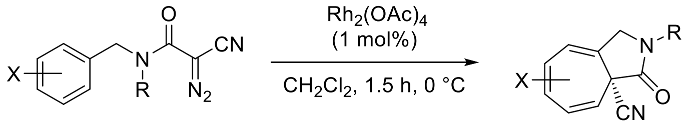

31, 33–45 → **32, 46–58**

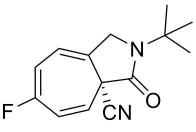

46
92%

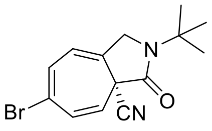

47
80%

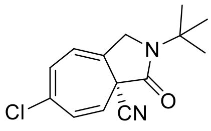

48
80%

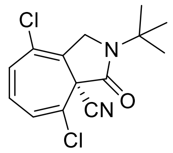

49
98%

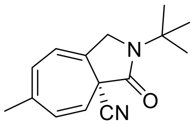

50
76%

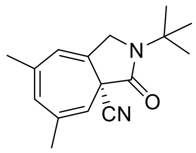

51
78%

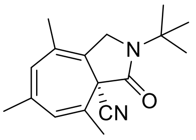

52
86%

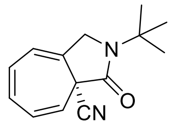

53
83%

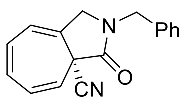

32
90%

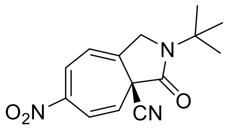

54
75%

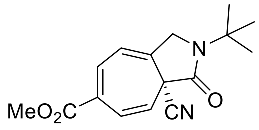

55
77%

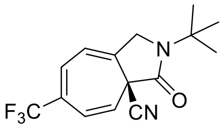

56
82%

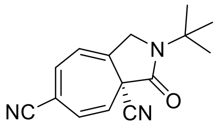

57
95%

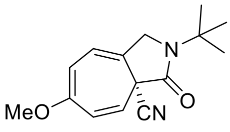

58
89%

[a]Isolated yields after chromatography.

Procedure C

A round bottom flask was charged with dichloromethane (35 mL) and Rh_2(2*S*-FOMePA)$_4$ **9e** (1.0 mol%). The flask was cooled to 0 °C, after which a solution of α-diazoacetamide (0.100 g) in CH_2Cl_2 (15 mL) was added over the course of 15 minutes. The reaction progress was monitored by IR spectroscopy. Following complete disappearance of the diazo stretch (2119–2129 cm^{-1}), the solution was concentrated under reduced pressure and the resulting residue was purified by silica gel chromatography using hexane:ethyl acetate (75:25) as eluent.

9-Aza-9-benzyl-1(*S*)-cyanobicyclo[5.3.0]deca-2,4,6-trien-10-one[21] (32)

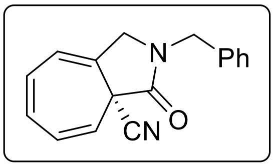

This title compound was prepared according to ***Procedure C*** from *N*,*N*-dibenzyl-α-cyano-α-diazoacetamide (0.10 g, 0.38 mmol) and Rh_2(2*S*-FOMePA)$_4$ **9e** (5.0 mg, 1.0 mol%) in dichloromethane (50 mL). Colourless crystals (0.080 g, 90%). Spectroscopic characteristics were consistent with previously reported data.[21] **m.p.** 172–175 °C (Lit.,[21] 172–174 °C); **Spec. Rot.:** $[\alpha]_D^{20}$ +136.00 (*c* 0.200, $CHCl_3$); **HPLC:** 73% ee (determined by chiral phase HPLC, see **Table SI.6** for HPLC conditions); **^{1}H NMR (400 MHz, $CDCl_3$):** δ= 3.93 (1H, d, *J* 14.9), 4.16 (1H, d, *J* 14.9) 4.59 (2H, s), 5.14 (1H, d, *J* 8.8), 6.28–6.37 (1H, m,), 6.40–6.49 (1H, m), 6.68–6.77 (2H, m), 7.22–7.41 (5H); **^{13}C NMR (100.6 MHz, $CDCl_3$):** δ= 41.9, 47.3, 49.7, 107.3 br, 113.0 br, 114.1, 122.6, 128.3, 129.6, 130.3, 128.5, 128.27, 129.1, 134.9, 167.4; **IR (neat):** 2238, 1704.

9-Aza-9-*tert*-butyl-1(*S*)-cyano-4-fluorobicyclo[5.3.0]deca-2,4,6-trien-10-one[21] (46)

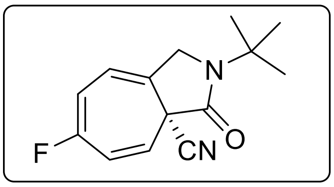

This title compound was prepared according to ***Procedure C*** from *N*-(*tert*-butyl)-2-cyano-2-diazo-*N*-(4'-fluorobenzyl) acetamide (0.10 g, 0.39 mmol) and Rh_2(2*S*-FOMePA)$_4$ **9e** (5.0 mg, 1.0 mol%) in dichloromethane (50 mL). Spectroscopic characteristics were consistent with previously reported data.[21] Colourless crystals (0.075 g, 83%); **m.p.** 175–177 °C (Lit.,[21] 177–178 °C); **Spec. Rot.:** $[\alpha]_D^{20}$ +119.67 (*c* 0.300, $CHCl_3$); **HPLC:** 88% ee (determined by chiral phase HPLC, see **Table SI.6** for HPLC conditions); **^{1}H NMR (400 MHz, $CDCl_3$):** δ= 1.48 (9H, s), 4.19 (1H, d, *J* 15.3), 4.43 (1H, d, *J* 15.3), 5.44 (1H, dd, *J* 9.7, 4.9), 6.34–6.44 (2H, m), 6.51 (1H, dd, *J* 16.0, 7.7); **^{13}C NMR (100.6 MHz, $CDCl_3$):** δ= 27.4, 45.5, 48.7, 55.7, 112.0 ($^2J_{CF}$ 28.7), 114.5, 115.7 br, 117.0 br, 120.0 (d, $^3J_{CF}$ 11.3), 122.7 (d, $^2J_{CF}$ 35.7) 161.0 (d, $^1J_{CF}$ 248.6) 166.2; **^{19}F NMR (376.5 MHz, $CDCl_3$):** δ= –97.5; **IR (neat):** 2239, 1704.

9-Aza-9-*tert*-butyl-1(*S*)-cyano-4-bromobicyclo[5.3.0]deca-2,4,6-trien-10-one[21] (47)

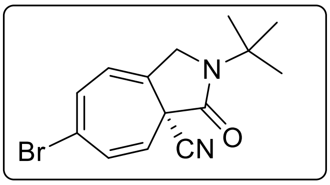

This title compound was prepared according to ***Procedure C*** from *N*-(*tert*-butyl)-2-cyano-2-diazo-*N*-(4'-bromobenzyl) acetamide (0.10 g, 0.33 mmol) and Rh_2(2*S*-FOMePA)$_4$ **9e** (5.0 mg, 1.0 mol%) in dichloromethane (50 mL). Spectroscopic characteristics were consistent with previously reported data.[21] Colourless crystals (0.089 g, 72%); **m.p.** 167–169 °C (Lit.,[21] 169–171 °C); **Spec. Rot.:** $[\alpha]_D^{20}$ +148.58 (*c* 0.140, $CHCl_3$); **HPLC:** 85% ee (determined by chiral phase HPLC, see **Table SI.6** for HPLC conditions); **^{1}H NMR (400 MHz, $CDCl_3$):** δ= 1.47 (9H, s), 4.15 (1H, d, *J* 15.4), 4.36 (1H, dd, *J* 15.3, 1.1), 5.14 (1H, d, *J* 9.3), 6.23 (1H, d, *J* 7.1), 6.57 (1H, d *J* 9.4), 7.12 (1H, d, *J* 7.1); **^{13}C NMR (100.6 MHz, $CDCl_3$):** δ= 27.4, 44.1 br, 48.7, 55.7, 111.9 br, 114.1, 117.5 br, 122.0, 124.2, 131.9, 132.1, 166.2; **IR (neat):** 2238, 1702.

9-Aza-9-*tert*-butyl-1(*S*)-cyano-4-chlorobicyclo[5.3.0]deca-2,4,6-trien-10-one[21] (48)

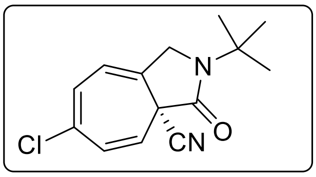

This title compound was prepared according to ***Procedure C*** from *N*-(*tert*-butyl)-2-cyano-2-diazo-*N*-(4'-chlorobenzyl)acetamide (0.10 g, 0.38 mmol) and Rh_2(2*S*-FOMePA)$_4$ **9e** (5.0 mg, 1.0 mol%) in dichloromethane (50 mL). Spectroscopic characteristics were consistent with previously reported data.[21] Colourless crystals (0.079 g, 83%); **m.p.** 172–175 °C (Lit.,[21] 178–179 °C); **Spec. Rot.:** $[\alpha]_D^{20}$ +152.50 (*c* 0.080, $CHCl_3$); **HPLC:** 84% ee (determined by chiral phase HPLC, see **Table SI.6** for HPLC conditions); **^{1}H NMR (400 MHz, $CDCl_3$):** δ= 1.47 (9H, s), 4.17 (1H, d, *J* 15.2), 4.38 (1H, dd, *J* 15.2, 1.4), 5.19 (1H, d, *J* 9.3), 6.30–6.35 (1H, m), 6.43 (1H, dd, *J* 9.3, 1.0), 6.88 (1H, d, *J* 7.1); **^{13}C NMR (100.6 MHz, $CDCl_3$):** δ= 27.4, 43.7, 48.8, 55.7, 110.7 br, 114.1, 115.5 br, 121.4, 128.7, 129.6, 135.2, 166.2; **IR (neat):** 2237, 1703.

9-Aza-9-*tert*-butyl-1(*R*)-cyano-2,6-dichlorobicyclo[5.3.0]deca-2,4,6-trien-10-one (49)

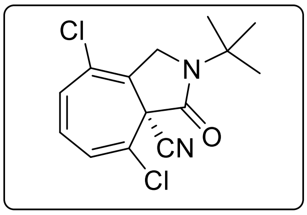

This title compound was prepared according to ***Procedure C*** from *N*-(*tert*-butyl)-2-cyano-2-diazo-*N*-(2',6'-dichlorobenzyl) acetamide (0.10 g, 0.34 mmol) and Rh_2(2*S*-FOMePA)$_4$ **9e** (5.0 mg, 1.0 mol%) in dichloromethane (50 mL). White crystals (0.099 g, 98%); **m.p.** 122–124 °C; **Spec. Rot.:** $[\alpha]_D^{20}$ +157.50 (*c* 0.080, $CHCl_3$); **HPLC:** 83% ee (determined by chiral phase HPLC, see **Table SI.6** for HPLC conditions); **^{1}H NMR (400 MHz, $CDCl_3$):** δ= 1.52 (9H, s), 4.23 (1H, d, *J* 16.6), 4.38 (1H, d, *J* 16.5), 6.63 (1H, d, *J* 6.7), 6.67–6.74 (1H), 6.82 (1H, d, *J* 11.4); **^{13}C NMR (100.6 MHz, $CDCl_3$):** δ= 27.4, 48.7, 51.0, 56.3, 112.5, 117.4, 119.0, 126.2, 127.5, 129.6, 130.0, 162.9; **IR (neat):** 2242, 1715; **HRMS (ESI-TOF):** *m/z* $[M+H]^+$ calcd for $C_{14}H_{15}{}^{35}Cl_2N_2O$, 297.0556, found 297.0559.

9-Aza-9-*tert*-butyl-1(*S*)-cyano-4-methylbicyclo[5.3.0]deca-2,4,6-trien-10-one[21] (50)

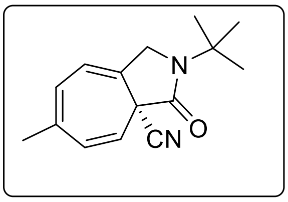

This title compound was prepared according to ***Procedure C*** from *N*-(*tert*-butyl)-2-cyano-2-diazo-*N*-(4'-methylbenzyl) acetamide (0.10 g, 0.41 mmol) and Rh_2(2*S*-FOMePA)$_4$ **9e** (5.0 mg, 1.0 mol%) in dichloromethane (50 mL). Spectroscopic characteristics were consistent with previously reported data.[21] Colourless crystals (0.086 g, 89%); **m.p.** 133–135 °C (Lit.,[21] 132–133 °C); **Spec. Rot.:** $[\alpha]_D^{20}$ +144.00 (*c* 0.100, $CHCl_3$); **HPLC:** 87% ee (determined by chiral phase HPLC, see Table **SI.6** for HPLC conditions); **^{1}H NMR (400 MHz, $CDCl_3$):** δ= 1.45 (9H, s), 2.11 (3H, s), 4.03 (1H, d, *J* 14.0), 4.25 (1H, d, *J* 14.0), 4.65 br (1H, d, *J* 8.2), 6.17 (1H, d, *J* 8.3), 6.22 (1H, d, *J* 7.2), 6.46 (1H, d, *J* 7.2); **^{13}C NMR (100.6 MHz, $CDCl_3$):** δ= 23.7, 27.4, 38.6 br, 49.0, 55.2, 96.2 br, 114.5, 122.1, 127.8, 128.1, 138.9, 167.4; **IR (neat):** 2238, 1702.

Note: Resonance for C*(2) not detected due to signal broadening*

9-Aza-9-*tert*-butyl-1(*S*)-cyano-3,5-dimethylbicyclo[5.3.0]deca-2,4,6-trien-10-one[21] (51)

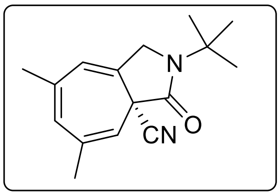

This title compound was prepared according to ***Procedure C*** from *N*-(*tert*-butyl)-2-cyano-2-diazo-*N*-(3',5'-dimethylbenzyl) acetamide (0.10 g, 0.39 mmol) and Rh_2(2*S*-FOMePA)$_4$ **9e** (5.0 mg, 1.0 mol%) in dichloromethane (50 mL). Spectroscopic characteristics were consistent with previously reported data.[21] Colourless crystals (0.080 g, 88%); **m.p.** 191–192 °C; **Spec. Rot.:** $[\alpha]_D^{20}$ +261.50 (*c* 0.100, $CHCl_3$); **HPLC:** 68% ee (determined by chiral phase HPLC, see **Table SI.6** for HPLC conditions); **^{1}H NMR (400 MHz, CDCl$_3$):** δ= 1.41 (9H, s), 2.00, 2.02 (2 × 3H, 2 × s), 3.38 (1H, br s), 3.73 (1H, d, *J* 12.2), 3.93 (1H, d, *J* 12.1), 5.77 (1H, s), 6.17 (1H, s); **^{13}C NMR (100.6 MHz, CDCl$_3$):** δ= 22.5, 22.6, 27.5, 49.8, 54.9, 113.4, 117.8, 128.1, 134.2, 136.3, 168.0; **IR (neat):** 2237, 1677; **HRMS (ESI-TOF):** *m/z* [M+H]$^+$ calcd for $C_{16}H_{21}N_2O$ 257.1648, found 257.1647.

Note: Resonances for C*(1),* C*(2)H and* C*(7) not detected due to signal broadening*

9-Aza-9-*tert*-butyl-1(*S*)-cyano-2,4,6-trimethylbicyclo[5.3.0]deca-2,4,6-trien-10-one[21] (52)

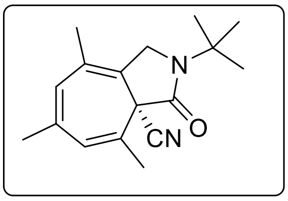

This title compound was prepared according to ***Procedure C*** from *N*-(*tert*-butyl)-2-cyano-2-diazo-*N*-(2',4',6'-trimethylbenzyl) acetamide (0.10 g, 0.37 mmol) and Rh_2(2*S*-FOMePA)$_4$ **9e** (5.0 mg, 1.0 mol%) in dichloromethane (50 mL). Spectroscopic characteristics were consistent with previously reported data.[21] Colourless crystals (0.070 g, 76%); **m.p.** 184–185 °C; **Spec. Rot.:** $[\alpha]_D^{20}$ +256.50 (*c* 0.100, $CHCl_3$); **HPLC:** 63% ee (determined by chiral phase HPLC, see **Table SI.6** for HPLC conditions); **^{1}H NMR (400 MHz, CDCl$_3$):** δ= 1.50 (9H, s), 1.84 (3H, s), 2.00 (3H, s), 2.10 (3H, s), 4.09 (1H, d, *J* 14.9), 4.28 (1H, d, *J* 14.8), 5.97 (1H, s), 6.41 (1H, s); **^{13}C NMR (100.6 MHz, CDCl$_3$):** δ= 17.8, 19.5, 24.3, 27.5, 48.3, 50.0, 55.4, 114.8, 116.1, 124.9, 127.8, 128.9, 130.0, 138.8, 166.8; **IR (neat):** 2236, 1699; **HRMS (ESI-TOF):** *m/z* [M+H]$^+$ calcd for $C_{17}H_{23}N_2O$ 271.1805, found 271.1799.

9-Aza-9-*tert*-butyl-1(*S*)-cyanobicyclo[5.3.0]deca-2,4,6-trien-10-one[21] (53)

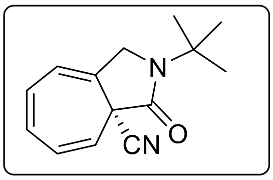

This title compound was prepared according to ***Procedure C*** from *N*-(*tert*-butyl)-2-cyano-2-diazo-*N*-benzyl-acetamide (0.10 g, 0.44 mmol) and Rh_2(2*S*-FOMePA)$_4$ **9e** (5.0 mg, 1.0 mol%) in dichloromethane (50 mL). Spectroscopic characteristics were consistent with previously reported data.[21] Colourless crystals (0.080 g, 89%); **m.p.** 147–149 °C (Lit.,[21] 147–148 °C); **Spec. Rot.:** $[\alpha]_D^{20}$ +159.00 (*c* 0.200, $CHCl_3$); **HPLC:** 72% ee (determined by chiral phase HPLC, see **Table SI.6** for HPLC conditions); **^{1}H NMR (400 MHz, CDCl$_3$):** δ= 1.47 (9H, s), 4.17 (H, dd, *J* 14.9, 1.1), 4.38 (H, dd, *J* 14.9, 1.9), 5.08 (1H, d, *J* 8.8), 6.34–6.44 (2H, m), 6.69–6.74 (2H, m); **^{13}C NMR (100.6 MHz, CDCl$_3$):** δ= 27.4, 43.3, 48.9, 55.4, 107.9 br, 113.7 br, 114.5, 122.2, 128.2, 129.4, 130.2, 167.1; **IR (neat):** 2236, 1700.

9-Aza-9-*tert*-butyl-1(*R*)-cyano-4-nitrobicyclo[5.3.0]deca-2,4,6-trien-10-one (54)

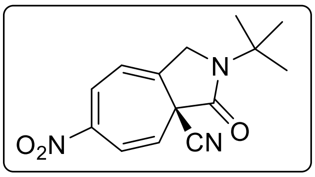

This title compound was prepared according to ***Procedure C*** from *N*-(*tert*-butyl)-2-cyano-2-diazo-*N*-(4'-nitrobenzyl) acetamide (0.10 g, 0.37 mmol) and Rh_2(2*S*-FOMePA)$_4$ **9e** (5.0 mg, 1.0 mol%) in dichloromethane (50 mL). Colourless crystals (0.063 g, 50%); **m.p.** 124–126 °C (Lit.,[21] 125–127 °C); **Spec. Rot.:** $[\alpha]_D^{20}$ –79.17

(c 0.120, $CHCl_3$); **HPLC:** 32% ee (determined by chiral phase HPLC, see **Table SI.6** for HPLC conditions); **^{1}H NMR (400 MHz, $CDCl_3$):** δ= 1.50 (9H, s), 4.32 (1H, d, J 16.7), 4.56 (1H, dd, J 16.7, 1.4), 5.55 (1H, d, J 9.7), 6.66 (1H, d, J 7.3), 7.33 (1H, d J 9.8), 8.12 (1H, d J 7.3); **^{13}C NMR (100.6 MHz, $CDCl_3$):** δ= 27.4, 45.2, 49.2, 56.0, 113.3, 115.5, 120.0, 123.3, 128.0, 129.4, 149.3, 165.3; **IR (neat):** 2243, 1704, 1520, 1335.

9-Aza-9-*tert*-butyl-1(*S*)-cyano-4-methoxycarbonylbicyclo[5.3.0]deca-2,4,6-trien-10-one[21] (55)

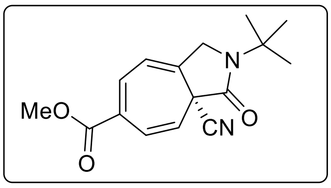

This title compound was prepared according to ***Procedure C*** from *N*-(*tert*-butyl)-2-cyano-2-diazo-*N*-(4'-carbomethoxybenzyl) acetamide (0.10 g, 0.35 mmol) and Rh_2(2*S*-FOMePA)$_4$ **9e** (5.0 mg, 1.0 mol%) in dichloromethane (50 mL). Spectroscopic characteristics were consistent with previously reported data.[21] Colourless crystals (0.084 g, 82%); **m.p.** 170–172 °C (Lit.,[21] 173–174 °C); **Spec. Rot.:** $[\alpha]_D^{20}$ +35.83 (c 0.120, $CHCl_3$); **HPLC:** 43% ee (determined by chiral phase HPLC, see **Table SI.6** for HPLC conditions); **^{1}H NMR (400 MHz, $CDCl_3$):** δ= 1.48 (9H, s), 3.87 (CH_3, s) 4.19 (1H, d, J 15.5), 4.42 (1H, dd, J 15.7, 1.5), 5.11 (1H, d, J 9.0), 6.50 (1H, d, J 7.2), 7.07 (1H, d J 9.0), 7.68 (1H, d, J 7.2); **^{13}C NMR (100.6 MHz, $CDCl_3$):** δ= 27.4, 41.9, 49.1, 52.6, 55.6, 105.3 br, 113.7, 115.5 br, 121.6, 128.2, 131.9, 133.7, 166.3, 166.4; **IR (neat):** 2240, 1704.

9-Aza-9-*tert*-butyl-1(*R*)-cyano-4-trifluoromethylbicyclo[5.3.0]deca-2,4,6-trien-10-one[21] (56)

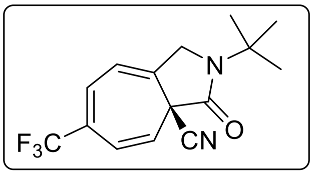

This title compound was prepared according to ***Procedure C*** from *N*-(*tert*-butyl)-2-cyano-2-diazo-*N*-(4'-trifluoromethylbenzyl) acetamide (0.10 g, 0.34 mmol) and Rh_2(2*S*-FOMePA)$_4$ **9e** (5.0 mg, 1.0 mol%) in dichloromethane (50 mL). Spectroscopic characteristics were consistent with previously reported data.[21] Colourless crystals (0.074 g, 76%); **m.p.** 153–155 °C (Lit.,[21] 154–156 °C); **Spec. Rot.:** $[\alpha]_D^{20}$ –24.29 (c 0.140, $CHCl_3$); **HPLC:** 27% ee (determined by chiral phase HPLC, see **Table SI.6** for HPLC conditions); **^{1}H NMR (400 MHz, $CDCl_3$):** δ= 1.48 (9H, s), 4.15 (1H, d, J 15.1), 4.39 (1H, d, J 15.2), 5.08 (1H, d, J 8.8), 6.47 (1H, d, J 7.1), 6.63 (1H, d J 8.9), 7.11 (1H, d, J 7.2); **^{13}C NMR (100.6 MHz, $CDCl_3$):** δ= 27.4, 40.3 br, 49.1, 55.7, 103.9 br, 113.1, 121.4, 123.1 (q, $^1J_{CF}$ 273.5), 124.2 (q, $^3J_{CF}$ 3.0), 127.6 (q, $^3J_{CF}$ 5.0), 131.7 (q, $^2J_{CF}$ 30.7), 166.1; **^{19}F NMR (376.5 MHz, $CDCl_3$):** δ= –65.8; **IR (neat):** 2245 (CN), 1703 (CO).

9-Aza-9-*tert*-butyl-1(*S*),4-dicyanobicyclo[5.3.0]deca-2,4,6-trien-10-one (57)

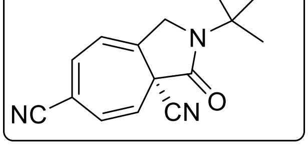

This title compound was prepared according to ***Procedure C*** from *N*-(*tert*-butyl)-2-cyano-2-diazo-*N*-(4'-cyanobenzyl) acetamide (0.10 g, 0.40 mmol) and Rh_2(2*S*-FOMePA)$_4$ **9e** (5.0 mg, 1.0 mol%) in dichloromethane (50 mL). Colourless crystals (0.073 g, 80%); **m.p.** 153–155 °C; **Spec. Rot.:** $[\alpha]_D^{20}$ +27.09 (c 0.120, $CHCl_3$); **HPLC:** 26% ee (determined by chiral phase HPLC, see **Table SI.6** for HPLC conditions); **^{1}H NMR (400 MHz, $CDCl_3$):** δ= 1.48 (9H, s), 4.23 (1H, d, J 16.1), 4.46 (1H, dd, J 16.2, 1.6), 5.23 (1H, d, J 9.0), 6.53 (1H, d, J 7.0), 6.61 (1H, d J 8.9), 7.26 (1H, d, J 6.5); **^{13}C NMR (100.6 MHz, $CDCl_3$):** δ= 27.4, 43.0, 49.1, 55.9, 111.0 br, 113.1, 115.1, 118.1, 120.7 br, 121.7, 128.4, 137.9 165.5; **IR (neat):** 2227, 1701; **HRMS (ESI-TOF):** m/z $[M+H]^+$ calcd for $C_{15}H_{16}N_3O$ 254.1288, found 254.1289.

9-Aza-9-*tert*-butyl-1(*S*)-cyano-4-methoxybicyclo[5.3.0]deca-2,4,6-trien-10-one[21] (58)

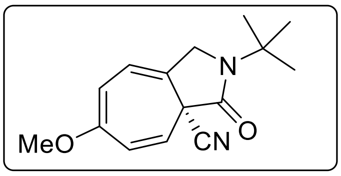

This title compound was prepared according to ***Procedure C*** from *N*-(*tert*-butyl)-2-cyano-2-diazo-*N*-(4'-methoxybenzyl)acetamide (0.10 g, 0.39 mmol) and Rh_2(2*S*-FOMePA)$_4$ **9e** (5.0 mg, 1.0 mol%) in dichloromethane (50 mL). Spectroscopic characteristics were consistent with previously reported data.[21] Colourless crystals (0.080 g, 88%); **m.p.** 118–120 °C (Lit.,[21] 119–120 °C); **Spec. Rot.:** $[\alpha]_D^{20}$ +36.50 (*c* 0.200, $CHCl_3$); **HPLC:** 20% ee (determined by chiral phase HPLC, see **Table SI.6** for HPLC conditions); **^{1}H NMR (400 MHz, $CDCl_3$):** δ= 1.45 (9H, s), 3.69 (3H, s), 4.03 (1H, d, *J* 13.7), 4.25 (1H, d, *J* 13.8), 4.83 (1H, br s), 5.95–6.03 (2H, m), 6.25 (1H, d *J* 8.1); **^{13}C NMR (100.6 MHz, $CDCl_3$):** δ= 27.4, 49.0, 55.1, 55.3, 109.1 br, 114.5, 117.7 br, 122.0, 158.6, 167.2; **IR (neat):** 2235, 1703.

2-Benzyl-3,6,8-trioxo-7-phenyl-2,3,3b,4,7,8-hexahydro-6H,10H-4,10-ethenopyrrolo-[3',4':1,3]cyclopropa[1,2-d][1,2,4]triazolo[1,2-a]pyridazine-3a(1H)-carbonitrile (59)

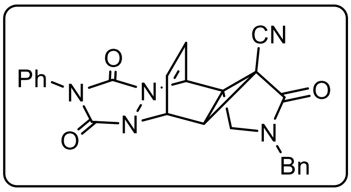

A 100 mL round bottom flask was charged with **32** (0.16 g, 0.62 mmol) and CH_2Cl_2 (20 mL). The flask was cooled to 0 °C after which 4-phenyl-1,2,4-triazolin-3,5-dione (0.108 g, 0.62 mmol) was added in one portion. The reaction solution was warmed to room temperature. The reaction solution was concentrated under reduced pressure and the solid residue was washed with chloroform to afford the pure cycloadduct **xx**. Colourless powder (0.136 g, 50%); **m.p.** 200–203 °C; **^{1}H NMR (400 MHz, DMSO-*d*$_6$):** δ= 2.48–2.53 (2H, m), 3.45 (1H, s, *J* 10.4), 3.86 (1H, s, *J* 10.4), 4.39 (2H, ABq, *J* 24.7, H_A δ = 4.46, H_B δ = 4.32), 5.49 (1H, dd, J 6.0, 1.4), 5.59–5.66 (1H, m), 6.43–6.51 (1H, m), 6.56–6.64 (1H, m), 7.24–7.55 (10H, m); **^{13}C NMR (100.6 MHz, DMSO-*d*$_6$):** δ= 25.7, 28.2, 31.1, 46.2, 47.6, 51.7, 53.4, 114.9, 126.1, 127.7, 128.0, 128.1, 128.7, 129.1, 129.2, 130.7, 135.8, 155.7, 156.0, 164.5; **IR (neat):** 2238, 1706, 1402; **HRMS (ESI-TOF):** *m*/*z* $[M+H]^+$ calcd for $C_{26}H_{21}N_4O_3$ 438.1561, found 438.1562.

9-Aza-9-*tert*-butyl-1-cyano-4-(4-methylphenyl)bicyclo[5.3.0]deca-2,4,6-trien-10-one (60)

Prepared according to literature procedure[22]

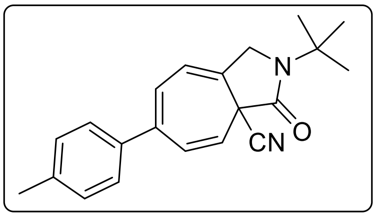

A Schlenk tube was charged with 9-aza-9-*tert*-butyl-1-cyano-4-bromobicyclo(5.3.0)deca-2,4,6-trien-10-one **47** (0.37 g, 1.21 mmol), palladium(II) acetate (0.014 g, 5 mol%), 4-tolylboronic acid (0.33 g, 2.44 mmol), SPhos (0.050 g, 10 mol%) and dioxane (12 mL). The reaction mixture was sparged with N_2 gas for 20 minutes after which aqueous K_3PO_4 (2.0 mL, 3.0 M) was added. The reaction mixture was stirred for 16 hours in a 25 °C oil bath. The solution was concentrated under reduced pressure and the residue purified by silica gel chromatography using hexane:ethyl acetate (80:20) as eluent; White crystals (0.246 g, 65%); **m.p.** 200–201 °C; **^{1}H NMR (400 MHz, $CDCl_3$):** δ= 1.47 (9H, s), 2.36 (3H, s), 4.10 (1H, d, *J* 14.3), 4.31 (1H, d, *J* 14.9), 4.88 (1H, d, *J* 8.5), 6.40 (1H, d, *J* 7.4), 6.50 (1H, d, *J* 8.5), 6.92 (1H, d, *J* 7.4), 7.19 (2H, d, *J* 8.0), 7.34 (2H, d, *J* 8.1); **^{13}C NMR (100.6 MHz, $CDCl_3$):** δ= 21.2, 27.5, 39.2 br, 49.1, 55.4, 98.1 br, 102.2 br, 114.3, 122.5, 126.8, 127.0, 127.2, 129.4, 137.8, 138.0, 142.3, 167.2; **IR (neat):** 2977, 2238, 1700; **HRMS (ESI-TOF):** *m*/*z* $[M+Na]^+$ calcd for $C_{21}H_{22}N_2ONa$ 341.1624, found 314.1627.

Methyl 9-aza-9-*tert*-butyl-4-methylbicyclo(5.3.0)deca-2,4,6-trien-10-one-1(*R*)-carboxylate (61)

Prepared according to literature procedure[21]

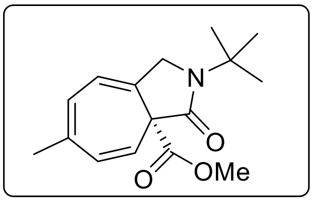

A 50 mL round bottom flask was charged with 9-aza-9-*tert*-butyl-1-cyano-4-methylbicyclo(5.3.0)deca-2,4,6-trien-10-one **50** (0.37 g, 1.5 mmol, 85% ee) and methanolic potassium hydroxide (10 mL, 2.2 M). The reaction solution was stirred at room temperature for 12 hours after which the solution was diluted with water (20 mL) and extracted with CH_2Cl_2 (2 × 20 mL). The organic solution was dried, concentrated under reduced pressure and the residue purified by silica gel chromatography using hexane:ethyl acetate (75:25) as eluent; White crystals (0.163 g, 40%); **m.p.** 143–145 °C; **Spec. Rot.:** $[\alpha]_D^{20}$ +320.72 (*c* 0.070, $CHCl_3$); **HPLC**: 77% ee (determined by chiral phase HPLC, see **Table SI.6** for HPLC conditions); **^{1}H NMR (400 MHz, $CDCl_3$):** δ= 1.45 (9H, s), 2.01 (3H, s), 3.62 (3H, s), 4.18 (1H, d, *J* 14.6), 4.38 (1H, d, *J* 14.6), 5.50 (1H, d, *J* 10.2), 6.11 (1H, d, *J* 6.5), 6.22 (1H, d, *J* 6.5), 6.36 (1H, d, *J* 10.2); **^{13}C NMR (100.6 MHz, $CDCl_3$):** δ= 24.4, 27.4, 49.6, 53.9, 54.6, 60.5, 119.4, 122.5, 126.0, 130.3, 132.3, 137.5, 166.8, 172.1; **IR (neat):** 2975, 1696, 1654; **HRMS (ESI-TOF):** *m*/*z* $[M+Na]^+$ calcd for $C_{16}H_{21}NO_3Na$ 298.1414, found 298.1411.

7. Synthesis of 2-allyl-2-methoxycarbonyl-2,3-dihydrobenzofuran-3-one

7.1 Synthesis of α-diazo-β-keto ester

Methyl 2-diazo-3-(2-allyloxyphenyl)-3-oxopropionate[23] (62)

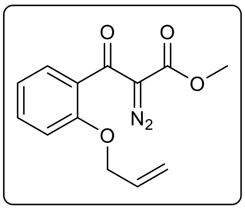

Methyl 3-(2-allyloxyphenyl)-3-oxopropanoate (0.64 g, 2.7 mmol), *p*-ABSA (0.70 g, 2.7 mmol), potassium carbonate (0.49 g, 3.6 mmol) and acetonitrile (30 mL) were used following the procedure described for **16** to give, following column chromatography on silica gel employing hexane/ethyl acetate (90:10) as the eluent, the α-diazo-β-keto ester **62** (0.59 g, 83%) as a yellow oil. Spectroscopic characteristics were consistent with previously reported data.[23] **^{1}H NMR (400 MHz, $CDCl_3$):** δ= 3.74 (3H, s), 4.51–4.57 (2H, finely split dt, *J* 5.0. 1.5), 5.27 (1H, dd, *J* 10.7, 1.3), 5.37 (1H, dd, *J* 17.3, 1.5), 5.92–6.04 (1H, m), 6.90 (1H, d, *J* 8.4), 6.98–7.04 (1H, m), 7.35 (1H, dd, *J* 7.6, 1.5), 7.37–7.45 (1H, m); **IR (neat):** 2124 (CN_2), 1727, 1697 (CO), 1622, 1311, 752.

7.2 Dihydrobenzofuranone synthesis

General procedure for rhodium catalysed oxonium ylide formation-[2,3]-sigmatropic rearrangements to afford dihydrobenzofuranones

A solution of α-diazocarbonyl (100 mg, 1 equiv.) in solvent (5 mL) was added dropwise over ~15 min to a stirring solution of Rh(II) catalyst (1 mol%) in solvent (5 mL). The mixture was stirred at the temperature indicated until reaction completion was indicated by IR spectroscopy. The reaction mixture was then cooled to room temperature, concentrated under reduced pressure to give the crude product and a ^{1}H NMR spectrum obtained. Purification by column chromatography employing ethyl acetate in hexane as eluent gave the pure cyclisation product.

2-Allyl-2-methoxycarbonyl-2,3-dihydrobenzofuran-3-one[24] (63)

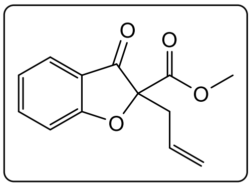

A solution of methyl 2-diazo-3-(2-allyloxyphenyl)-3-oxopropionate **62** (100 mg, 0.38 mmol) in toluene (5 mL) was added dropwise over ~15 min to a solution of **9b** (6 mg, 1 mol%) in toluene(5 mL). The mixture was heated under reflux while stirring for 2 h then cooled to room temperature and concentrated under reduced pressure. Column chromatography on silica gel employing hexane/ethyl acetate (95:5) as the eluent gave the pure dihydrobenzofuranone **63** (64.3 mg, 72%) as a white solid. Spectroscopic characteristics were consistent with previously reported data.[24] **m.p.** 58–60 °C; **HPLC**: 74% ee (determined by chiral phase HPLC, see **Table SI.6** for HPLC conditions); **^{1}H NMR (400 MHz, $CDCl_3$):** δ= 2.84 (1H, dd, *J* 14.5, 7.1), 3.07 (1H, dd, *J* 14.5, 7.1), 3.77 (3H, s), 5.12 (1H, d, *J* 10.1), 5.23 (1H, dd, *J* 17.0, 0.8), 5.61–5.74 (1H, m), 7.13 (1H, t, *J* 7.4), 7.23 (1H, d, *J* 8.7), 7.62–7.70 (2H, m); **IR (neat):** 1748, 1709 (CO), 1607, 1250, 929, 752.

8. NMR Spectra

2-Phenylacetic acid (S1)

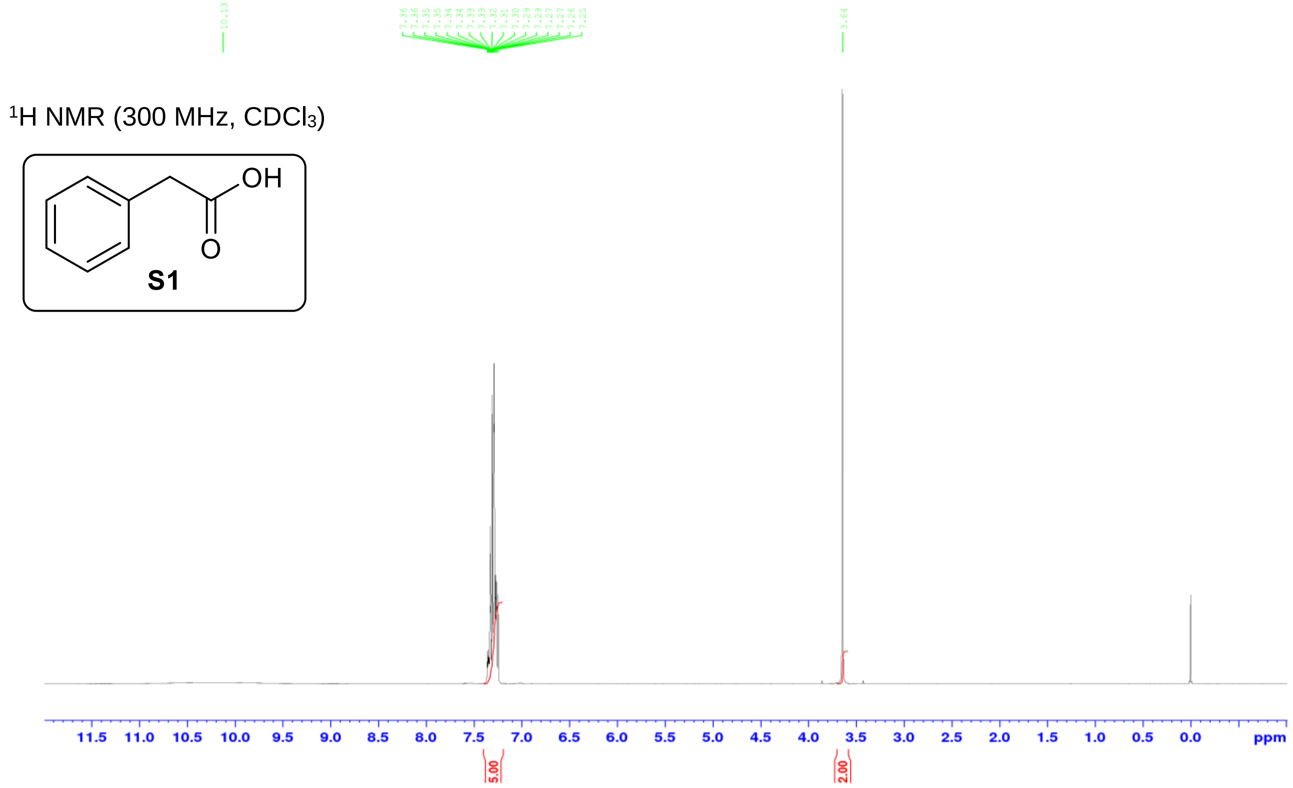

tert-Butyl 2-phenylacetate (S2)

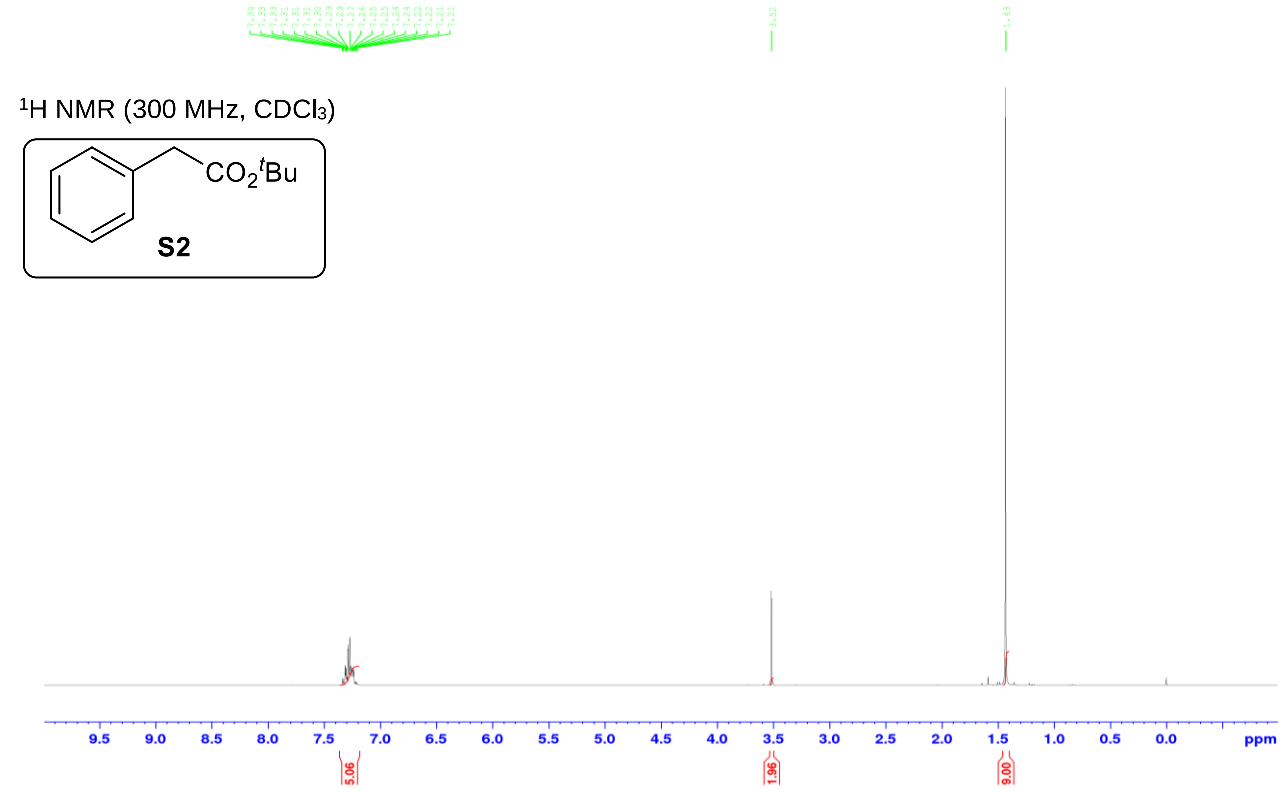

tert-Butyl 2-(naphthalen-2-yl)acetate (S3)

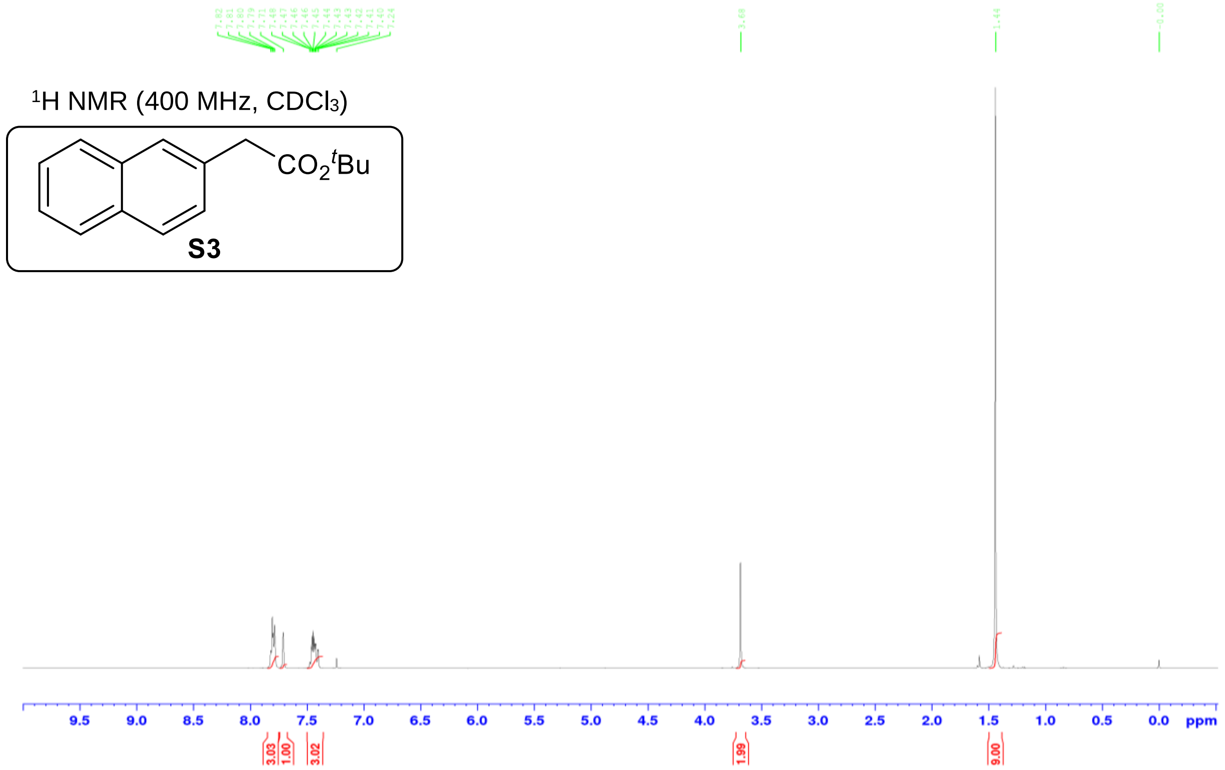

tert-Butyl 2-(naphthalen-1-yl)acetate (S4)

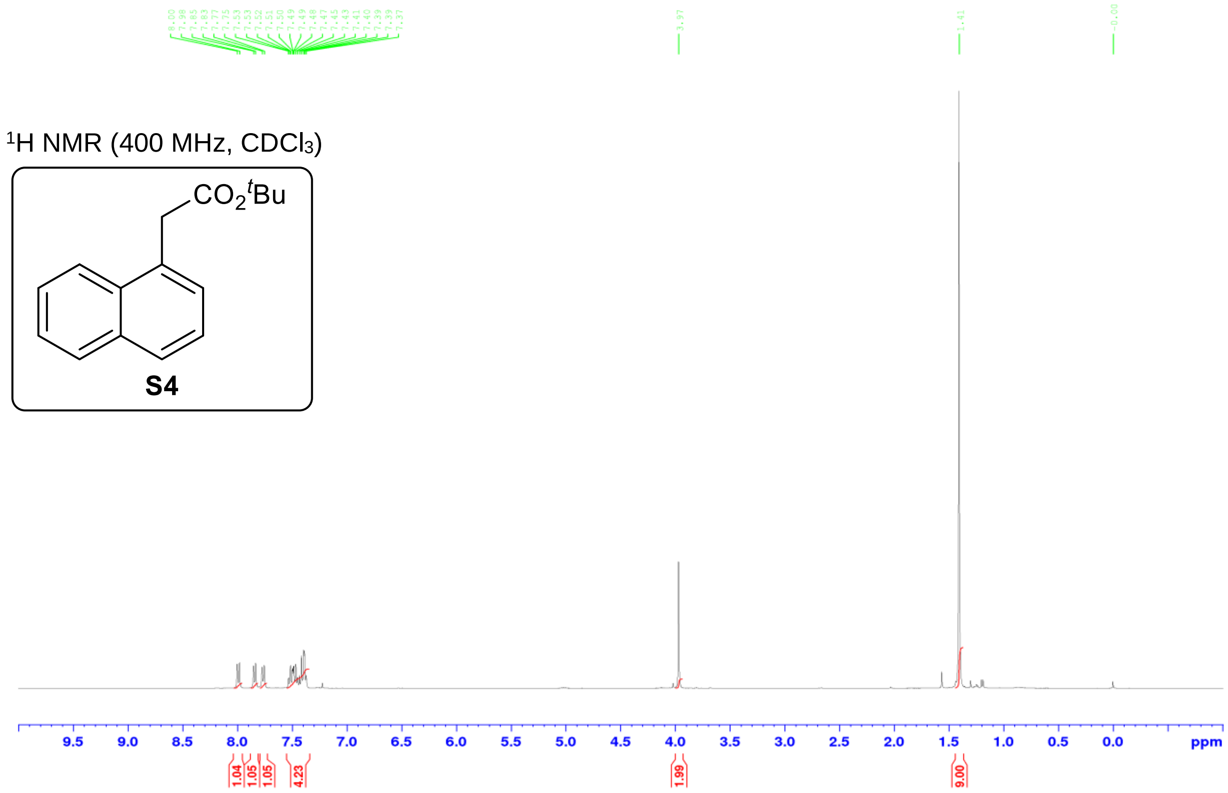

tert-Butyl 2-(4-bromophenyl)acetate (S5)

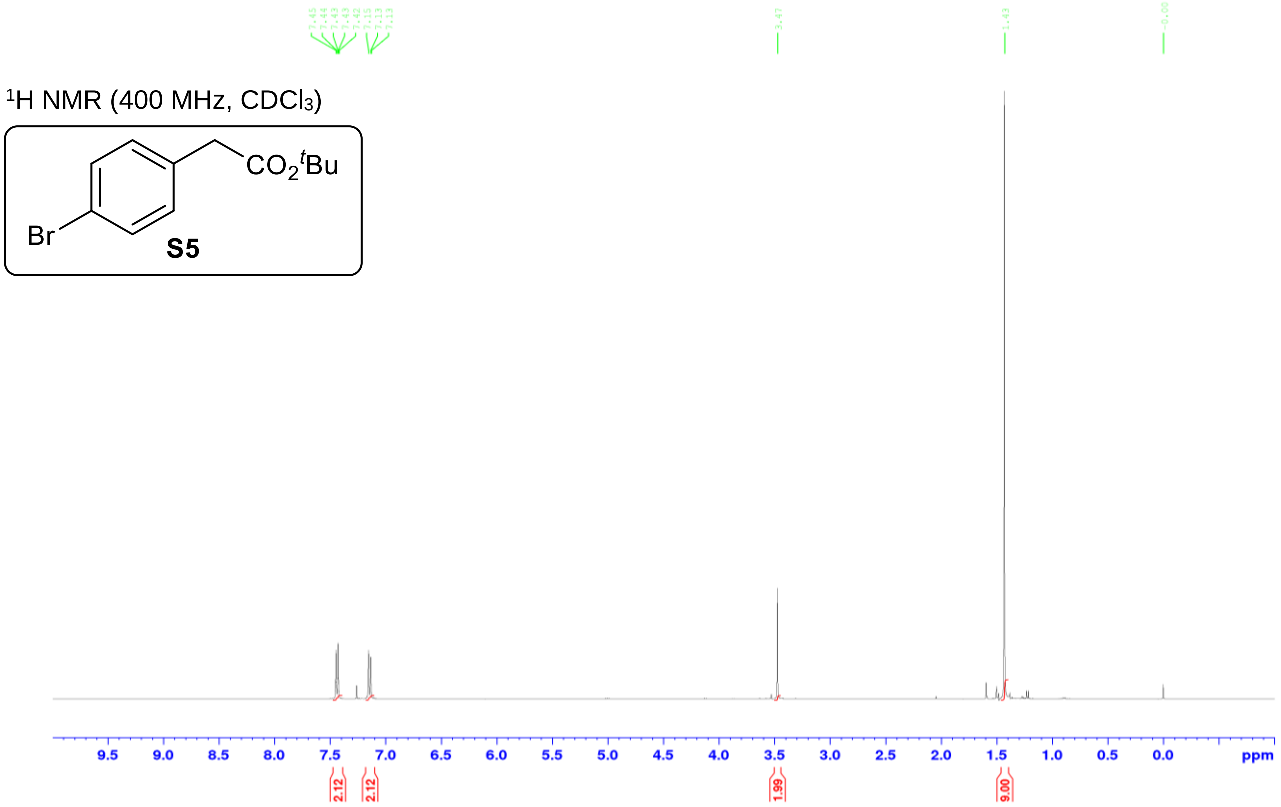

tert-Butyl 2-(4-methoxyphenyl)acetate (S6)

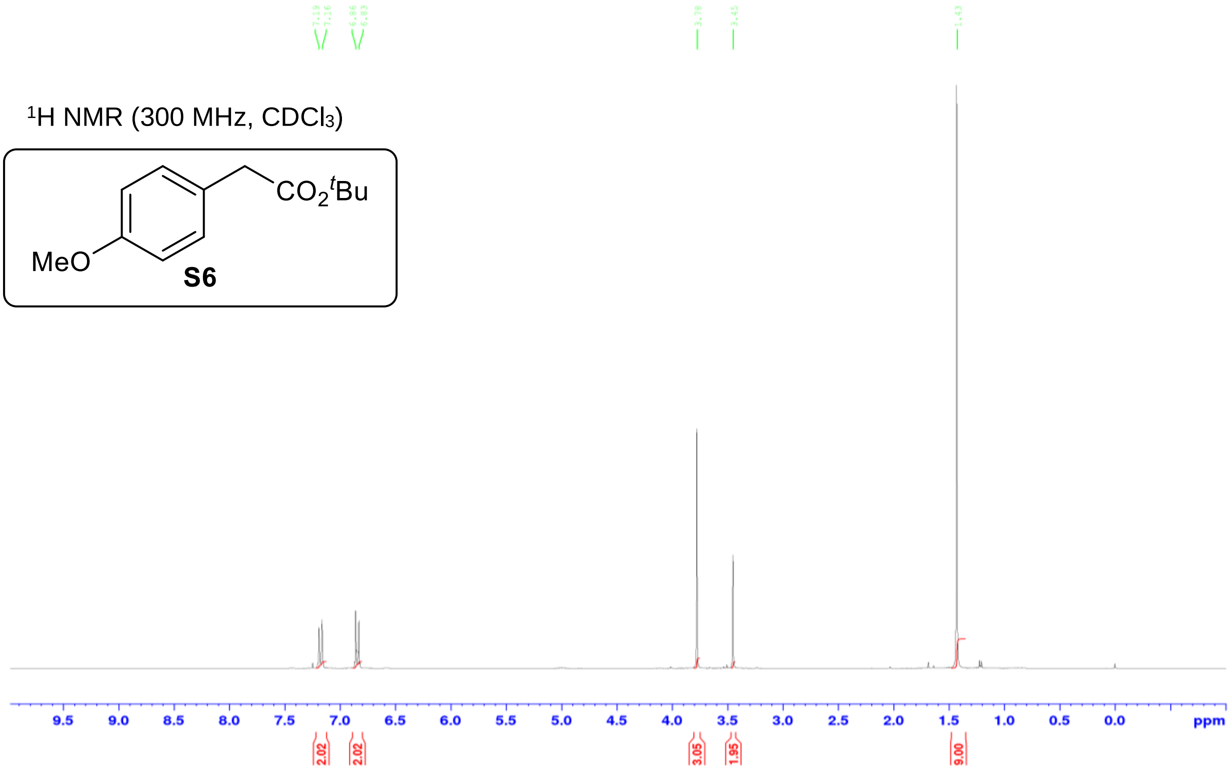

tert-Butyl 2-diazo-2-phenylacetate (6a)

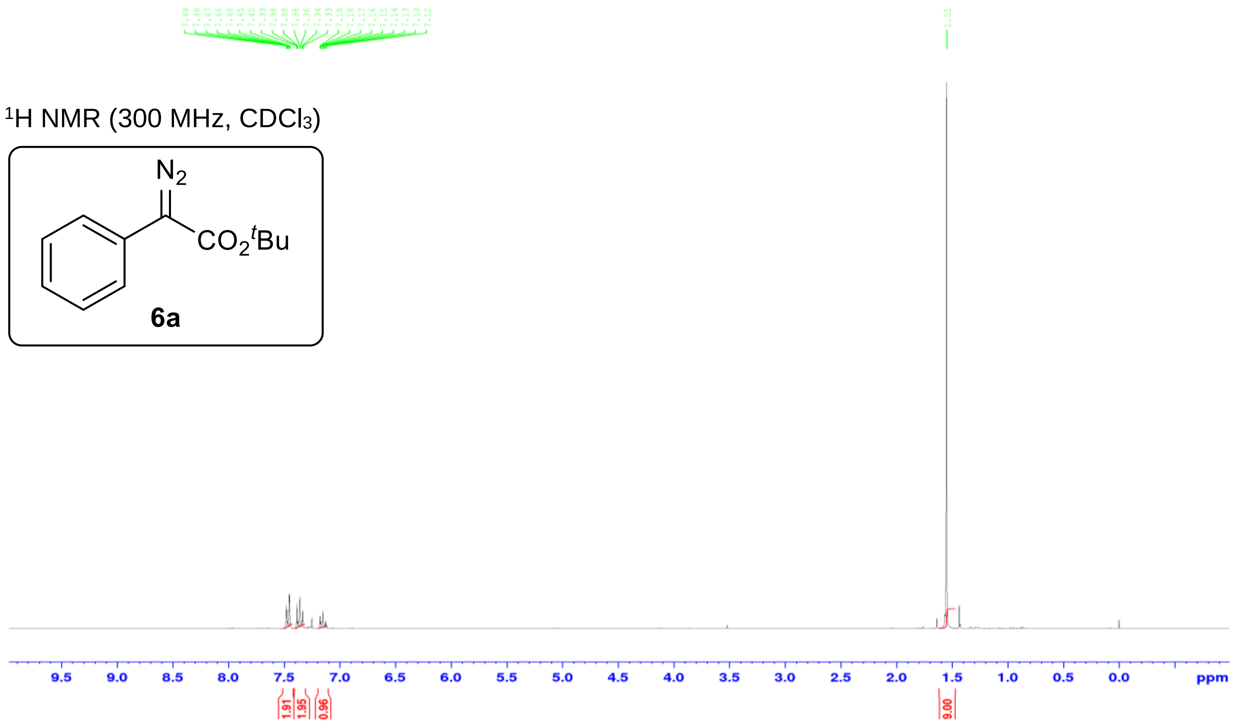

tert-Butyl 2-diazo-2-(naphthalen-2-yl)acetate (6b)

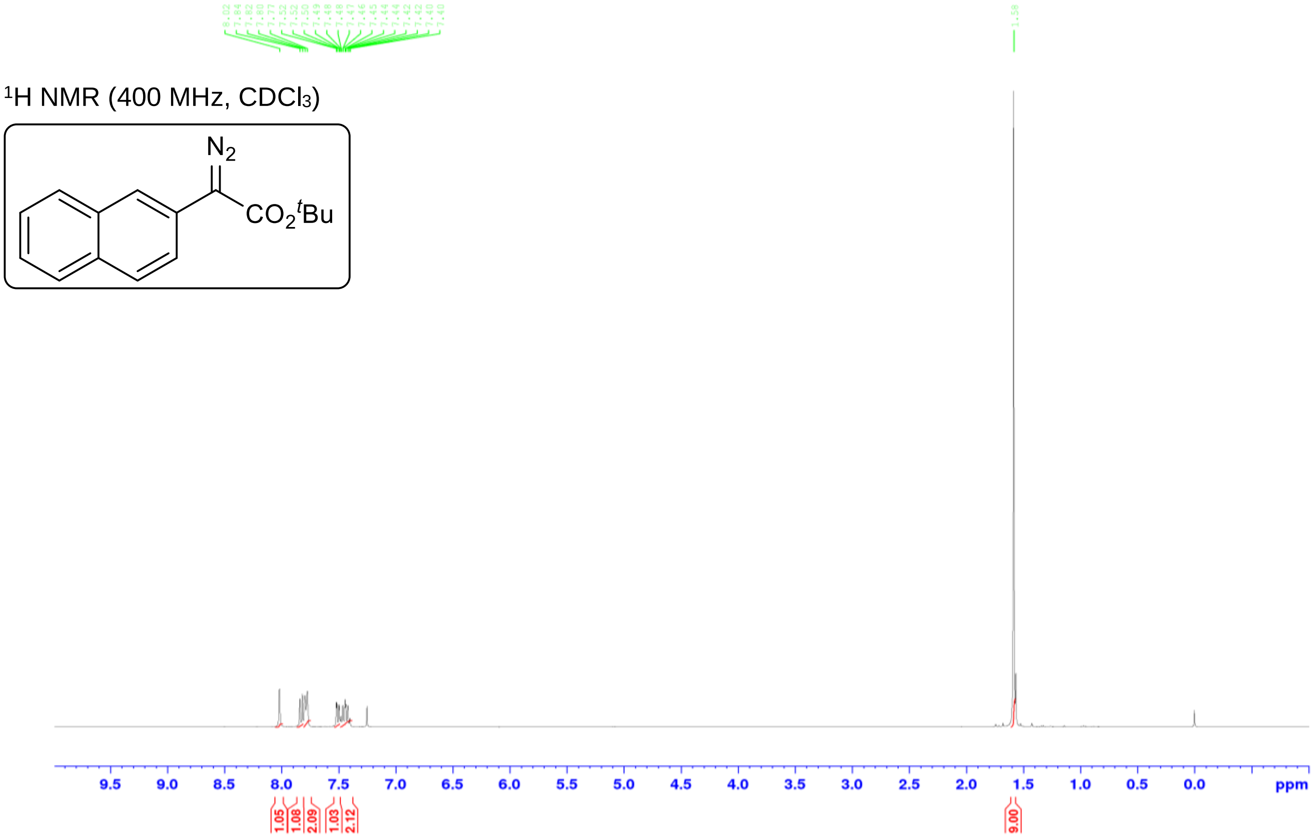

tert-Butyl 2-diazo-2-(naphthalen-1-yl)acetate (6c)

^{1}H NMR (400 MHz, $CDCl_3$)

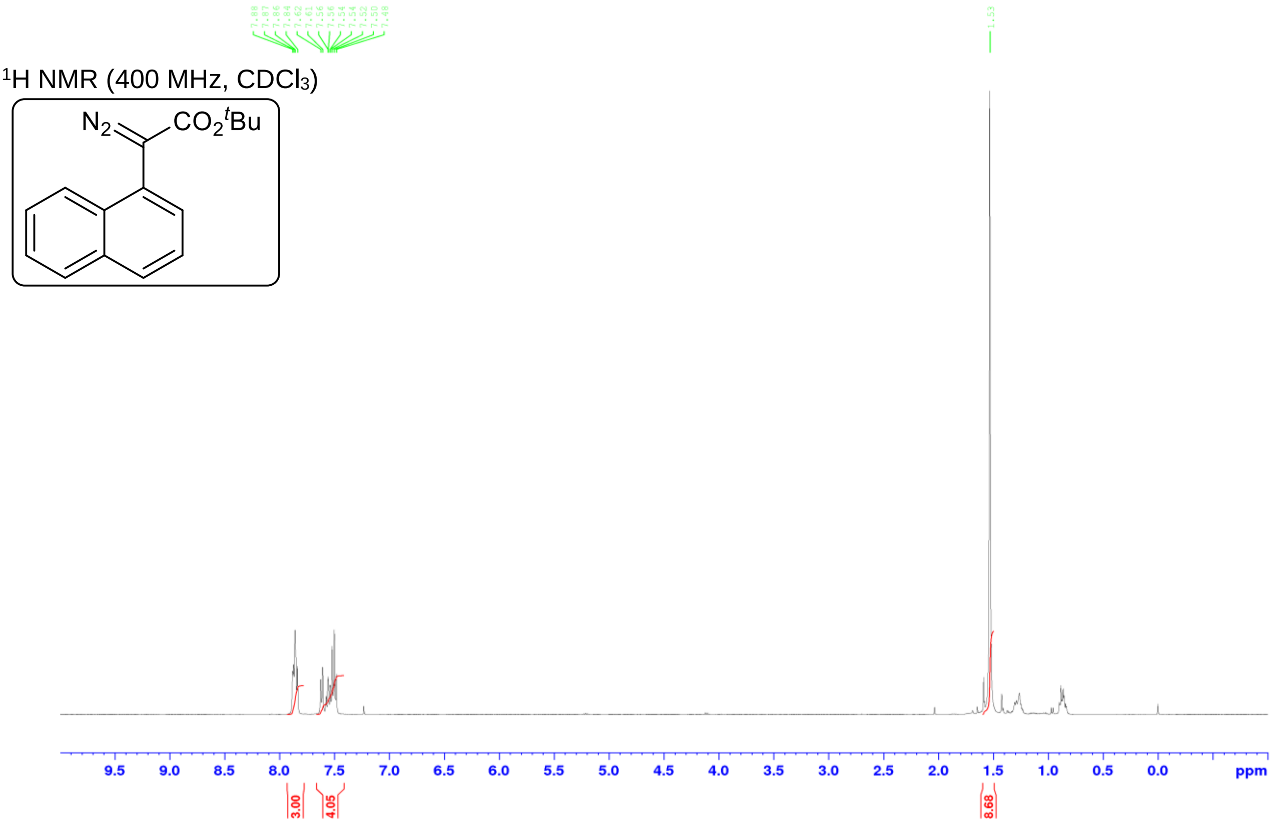

^{13}C NMR (75.5 MHz, $CDCl_3$)

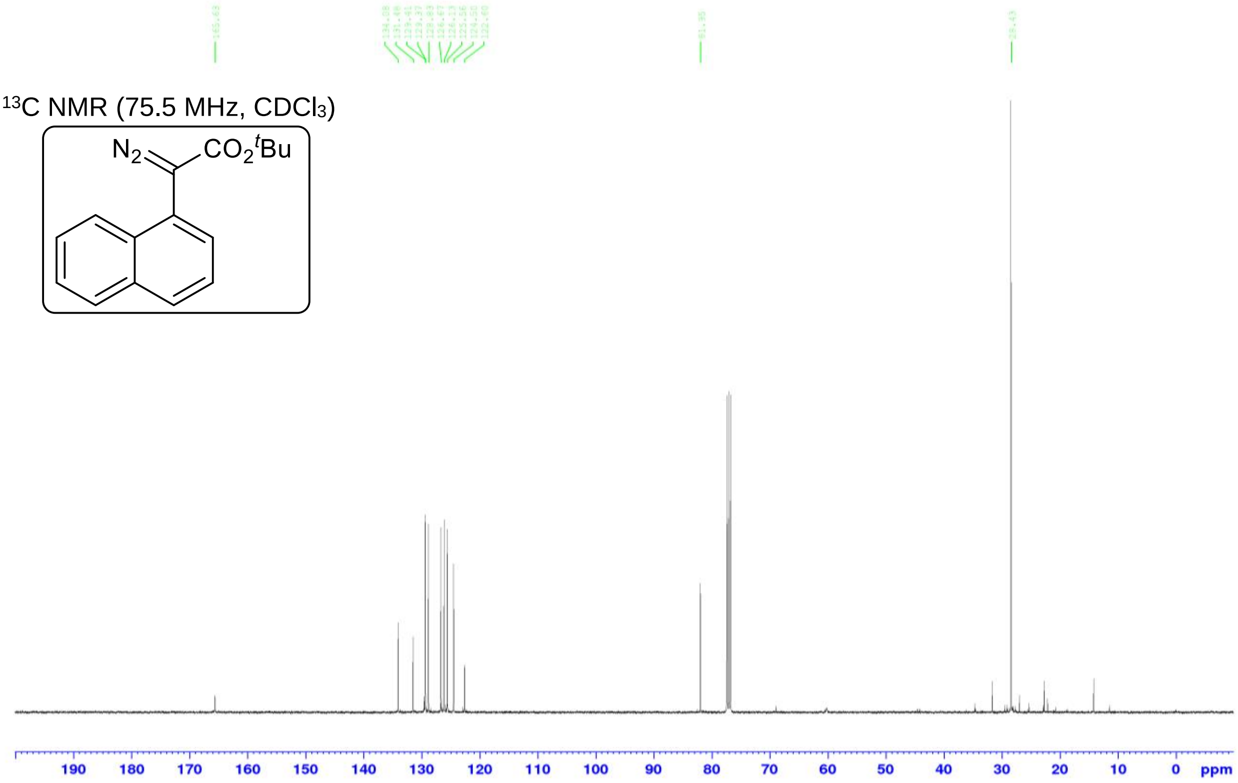

tert-Butyl 2-(4-bromophenyl)-2-diazoacetate (6d)

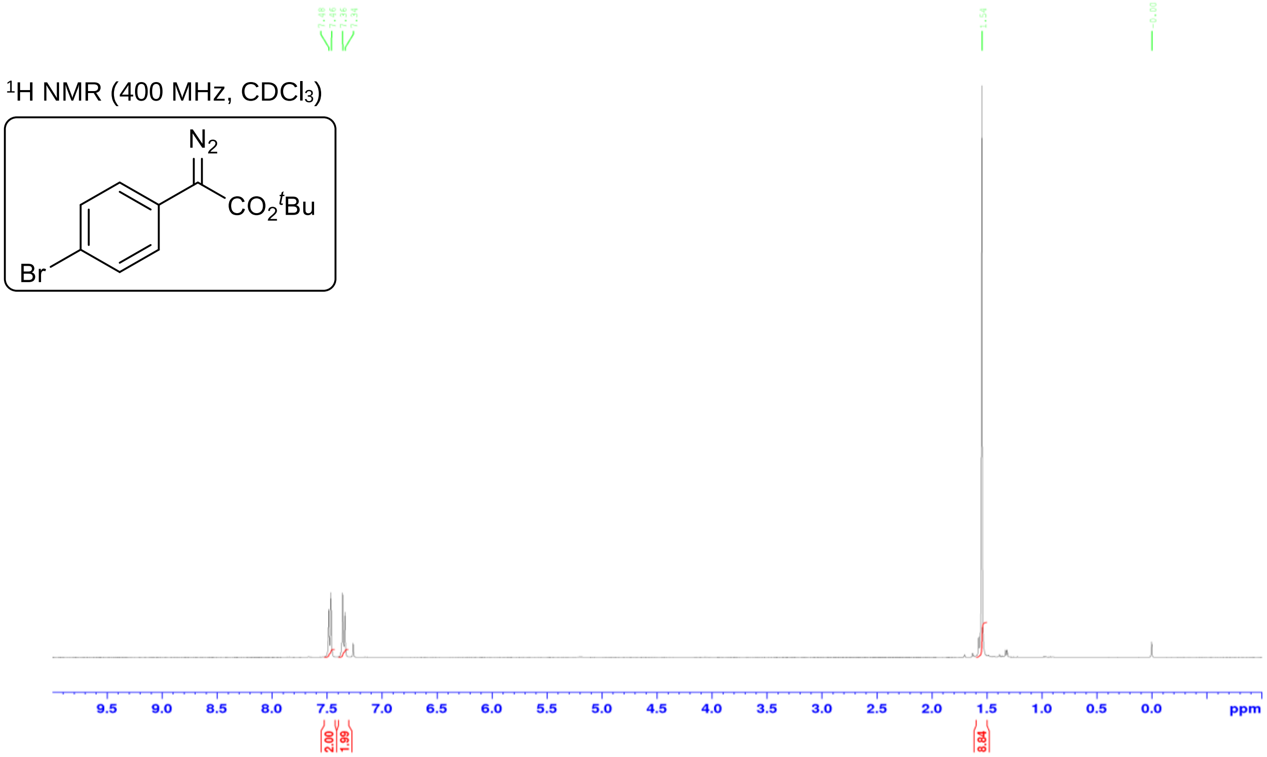

tert-Butyl 2-(4-methoxyphenyl)-2-diazoacetate (6e)

^{1}H NMR (400 MHz, $CDCl_3$)

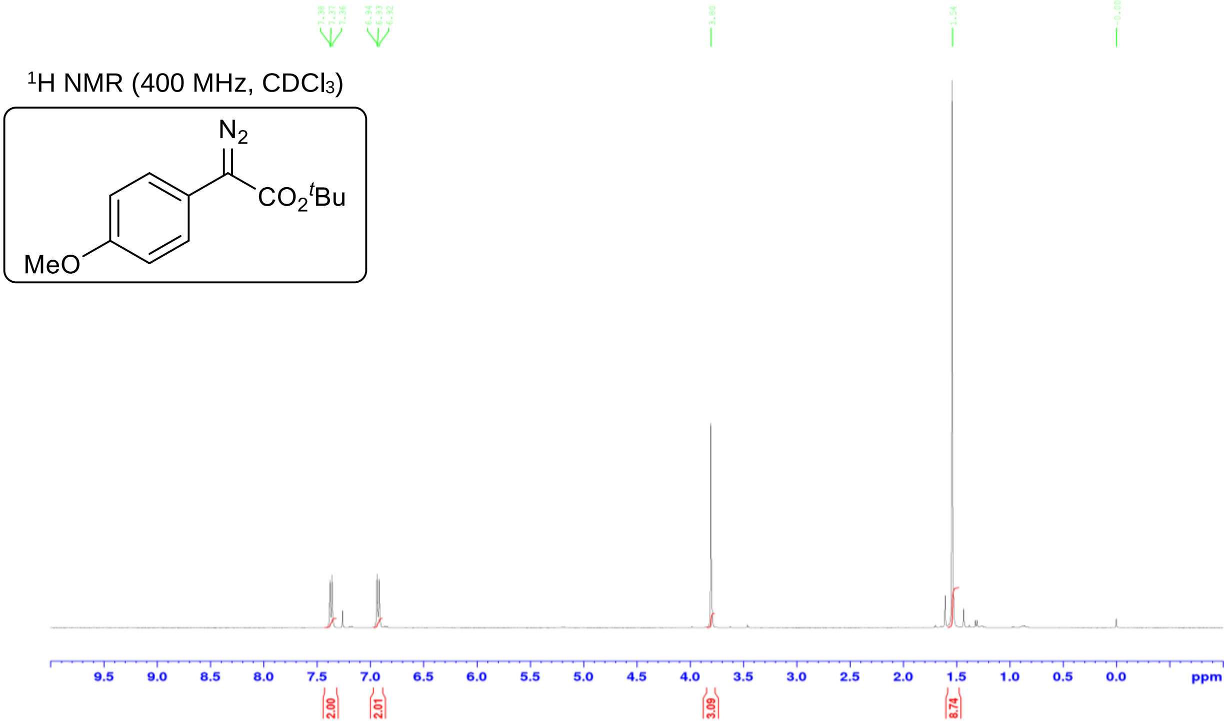

^{13}C NMR (75.5 MHz, $CDCl_3$)

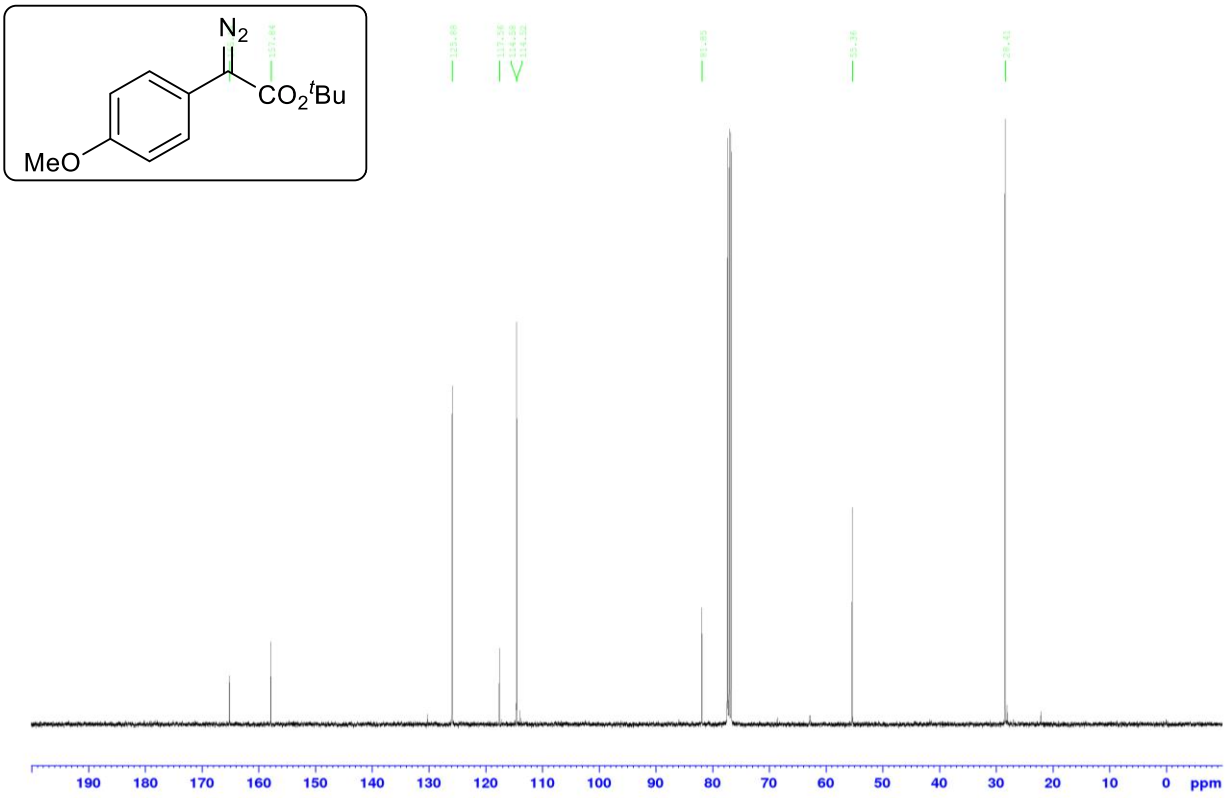

tert-Butyl (2*S*)-2-(1″*R*,2″*R*,4″*S*)-fenchyloxy-2-phenylacetate (7a)

^{1}H NMR (300 MHz, $CDCl_3$)

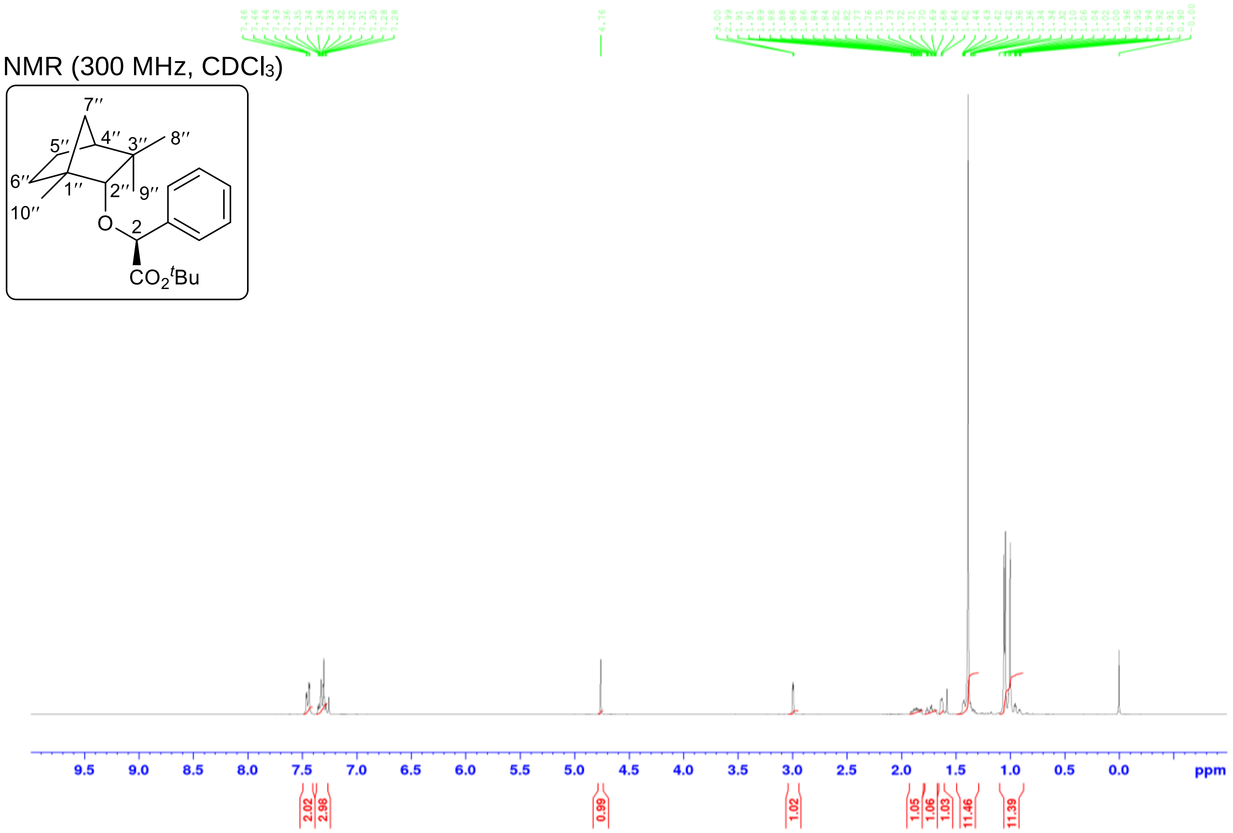

^{13}C NMR (75.5 MHz, $CDCl_3$)

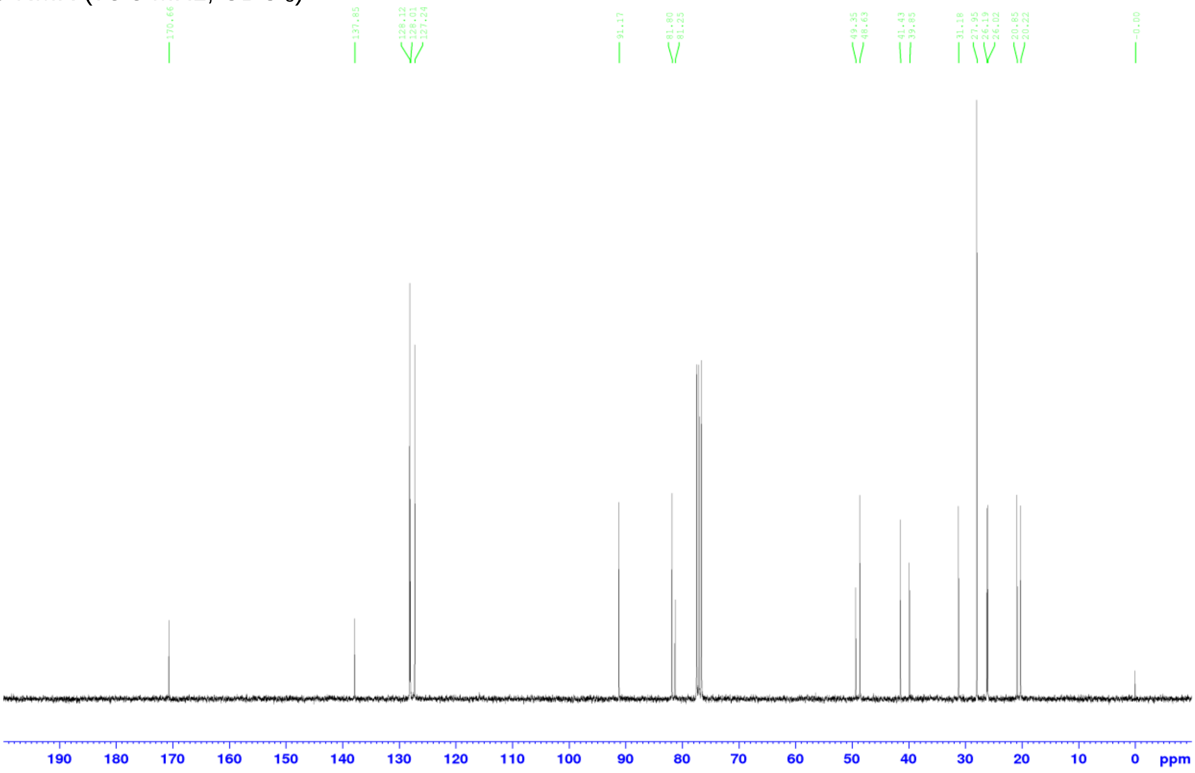

tert-Butyl (2_S_)-2-(1″_R_,2″_R_,4″_S_)-fenchyloxy-2-(naphthalen-2′-yl)acetate (7b)

^{1}H NMR (400 MHz, $CDCl_3$)

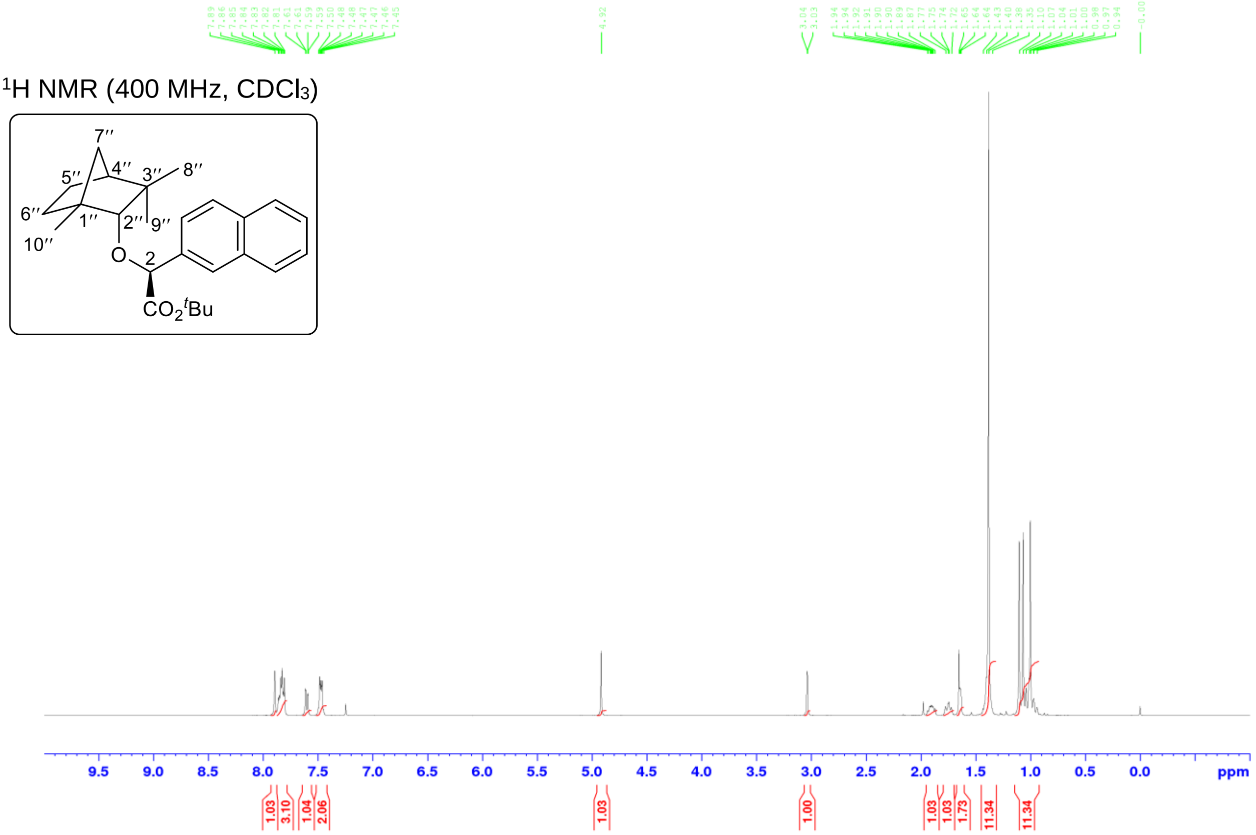

^{13}C NMR (100.6 MHz, $CDCl_3$)

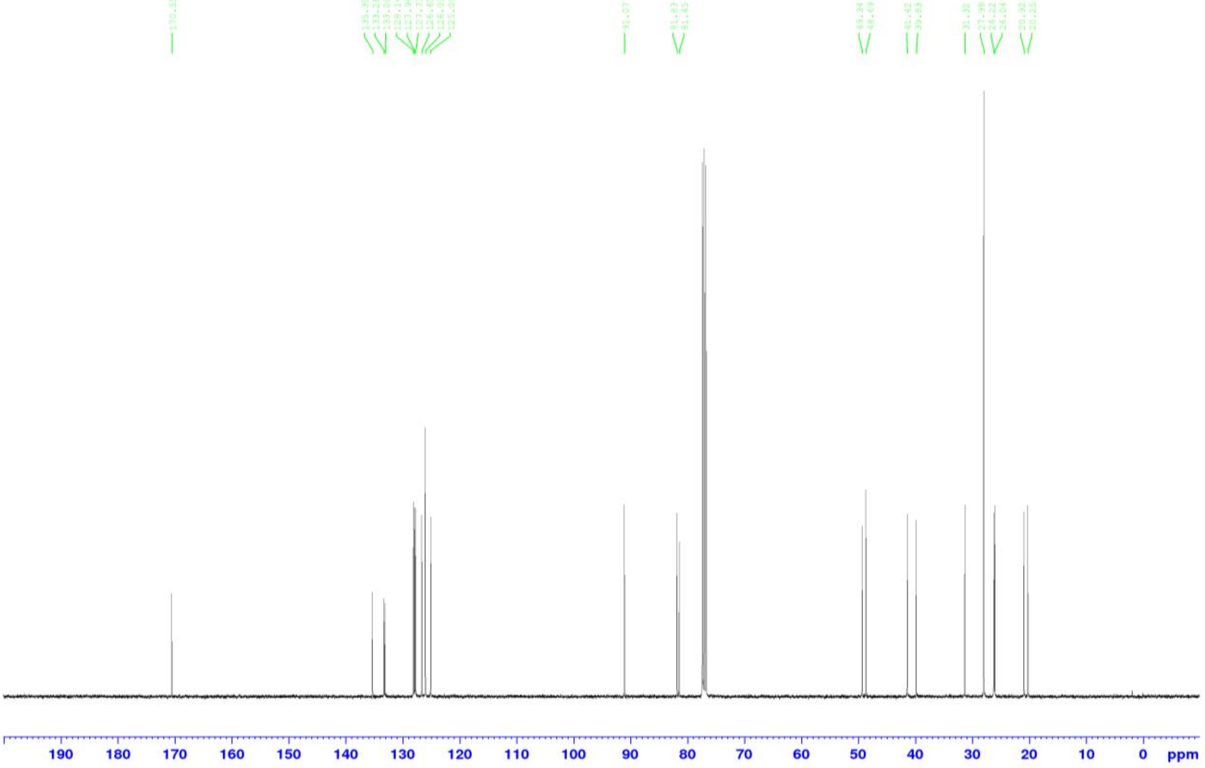

tert-Butyl (2*S*)-2-(1″*R*,2″*R*,4″*S*)-fenchyloxy-2-(naphthalen-1′-yl)acetate (7c)

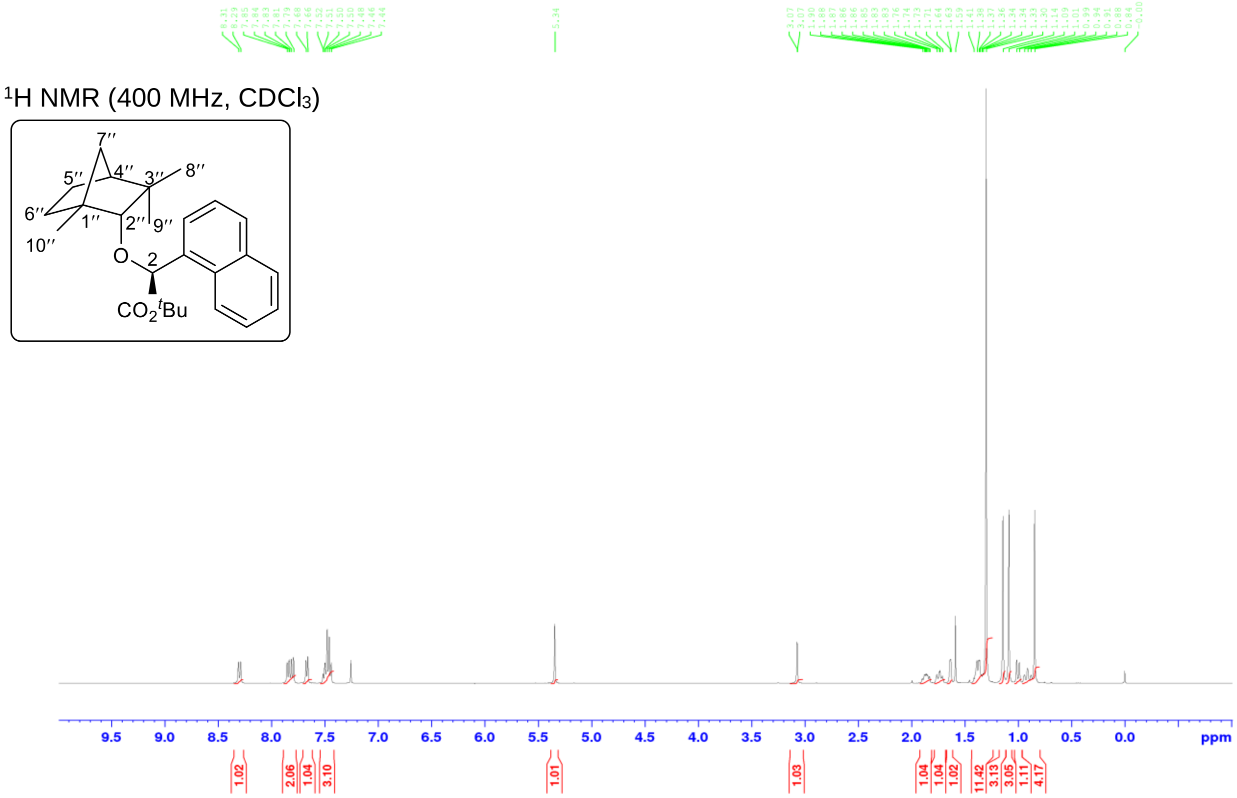

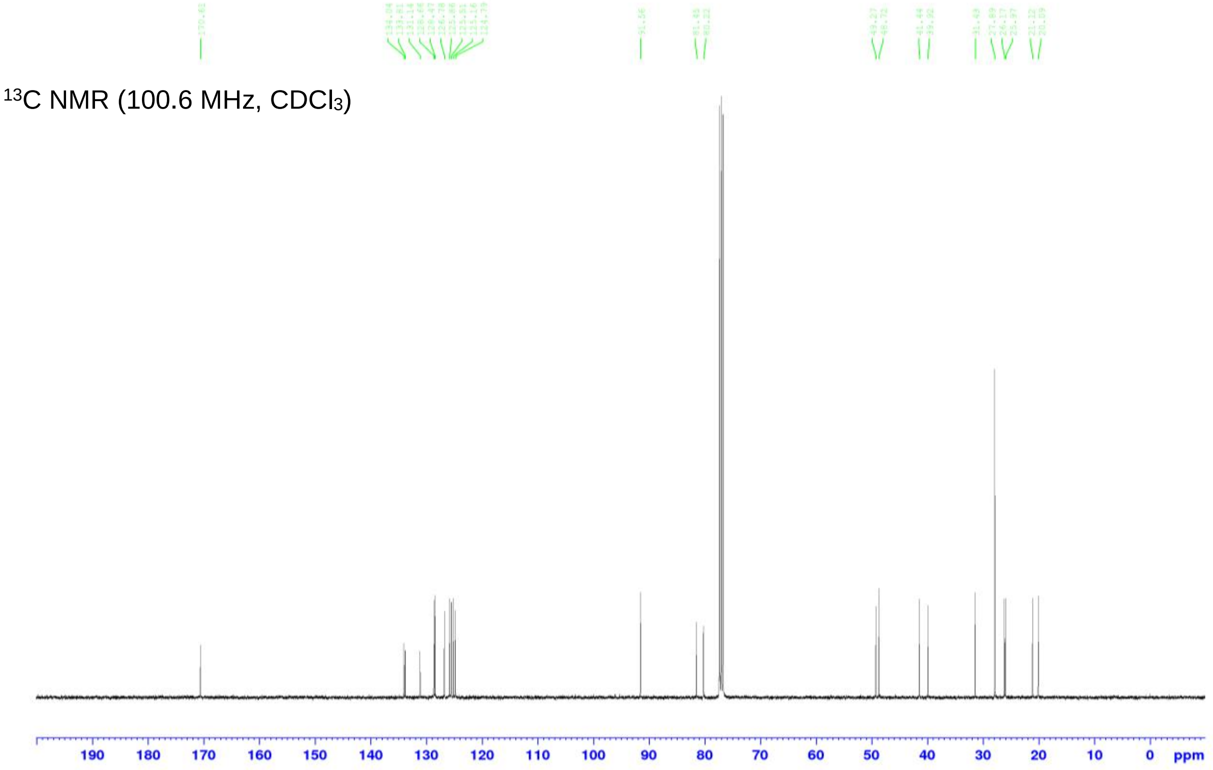

tert-Butyl (2*S*)-2-(4′-bromophenyl)-2-(1″*R*,2″*R*,4″*S*)-fenchyloxyacetate (7d)

^{1}H NMR (400 MHz, $CDCl_3$)

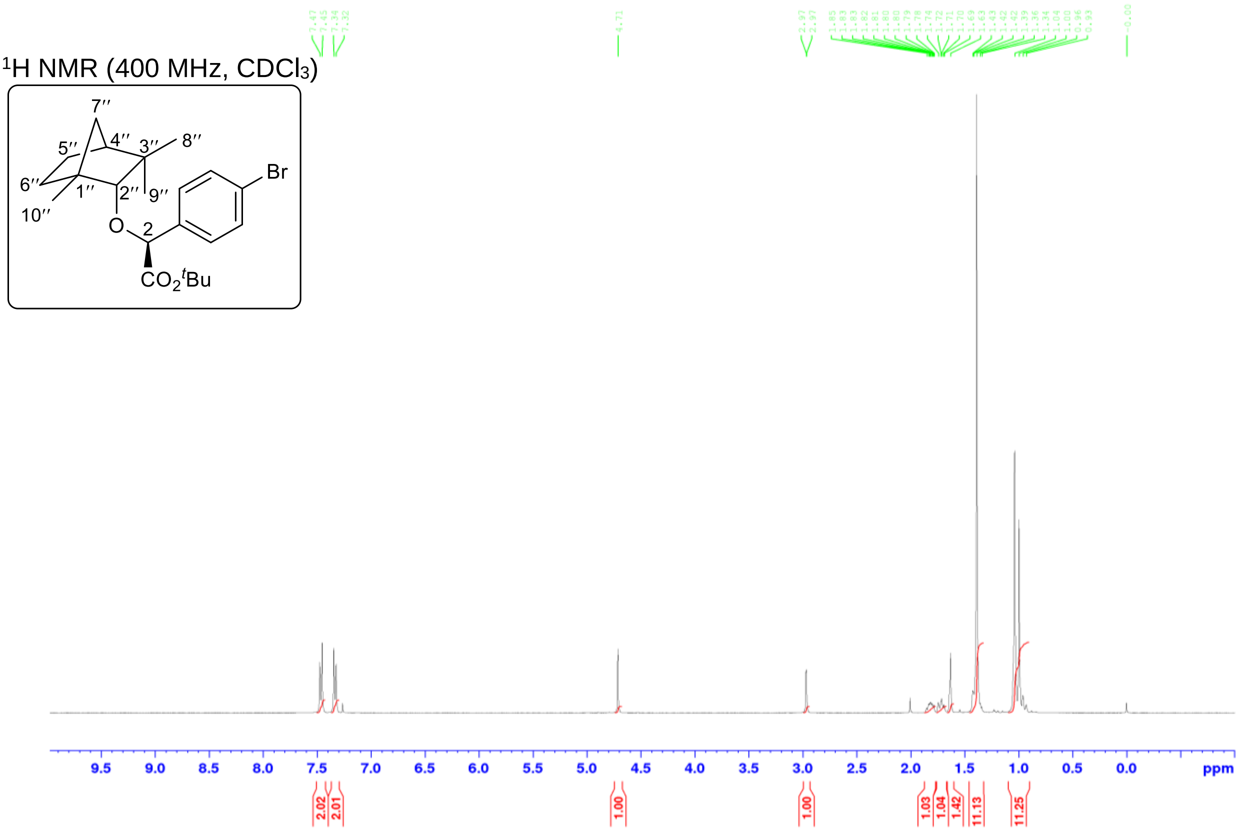

^{13}C NMR (100.6 MHz, $CDCl_3$)

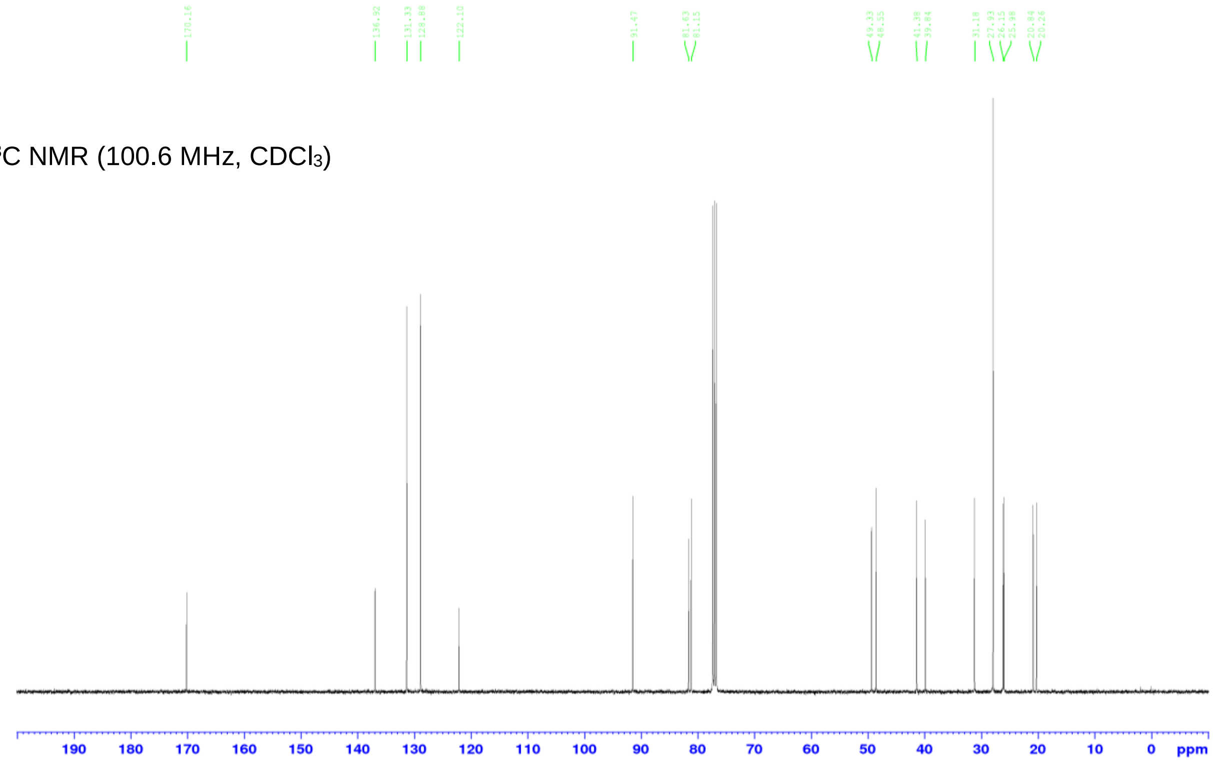

tert-Butyl (2_S_)-2-(1″_R_,2″_R_,4″_S_)-fenchyloxy-2-(4′-methoxyphenyl)acetate (7e)

^{1}H NMR (400 MHz, $CDCl_3$)

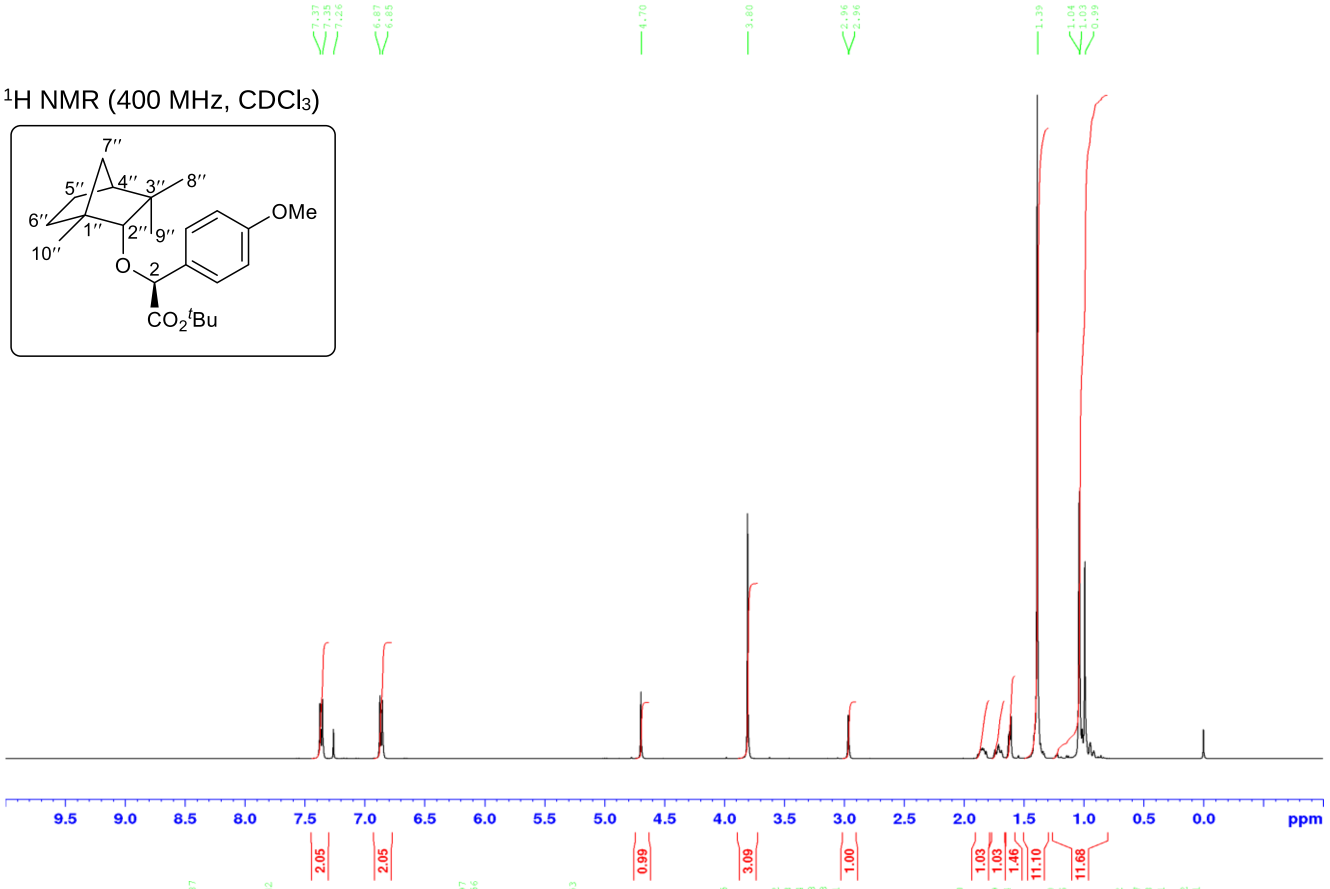

^{13}C NMR (100.6 MHz, $CDCl_3$)

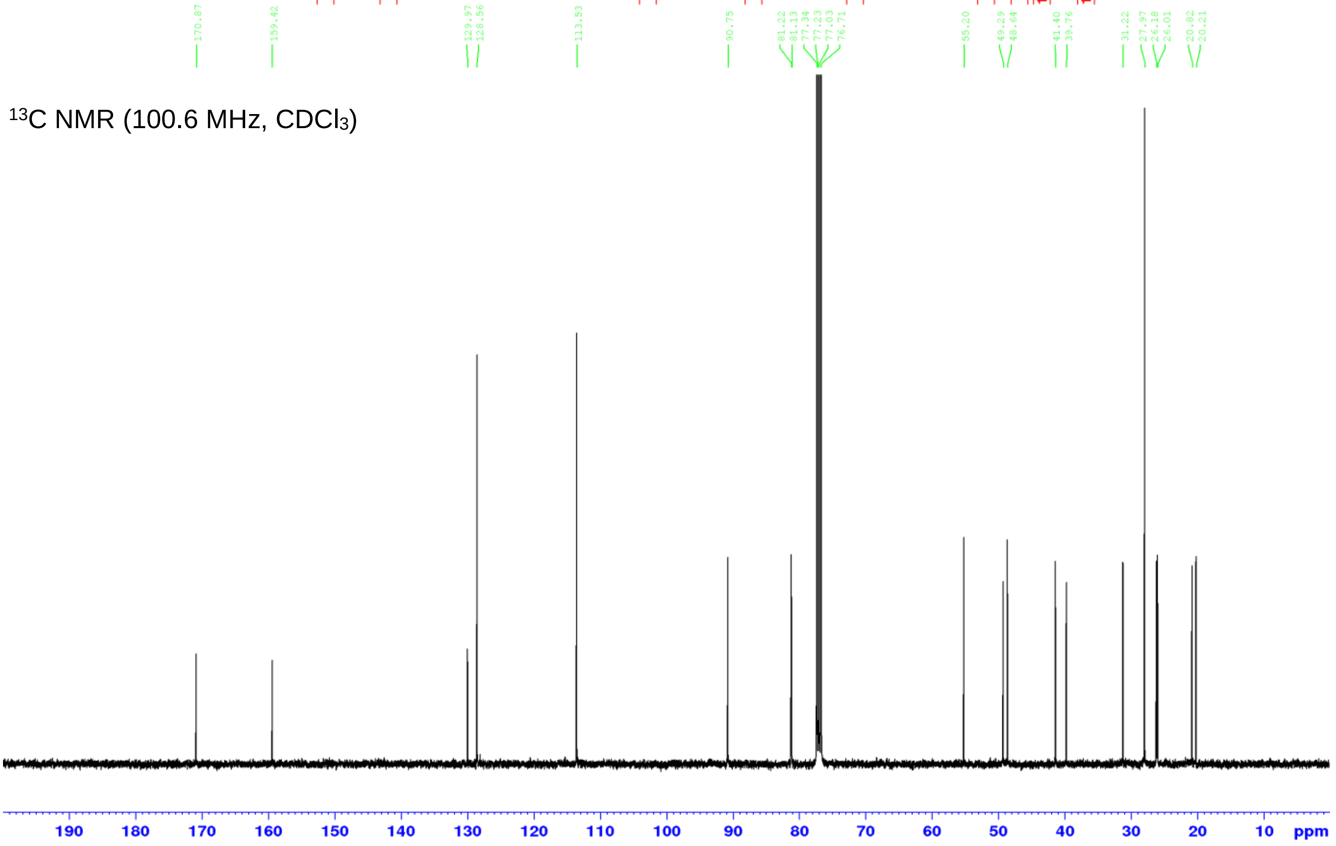

tert-Butyl (2*S*)-2-(1″*R*,2″*S*,5″*R*)-menthyloxy-2-phenylacetate (7f)

^{1}H NMR (300 MHz, $CDCl_3$)

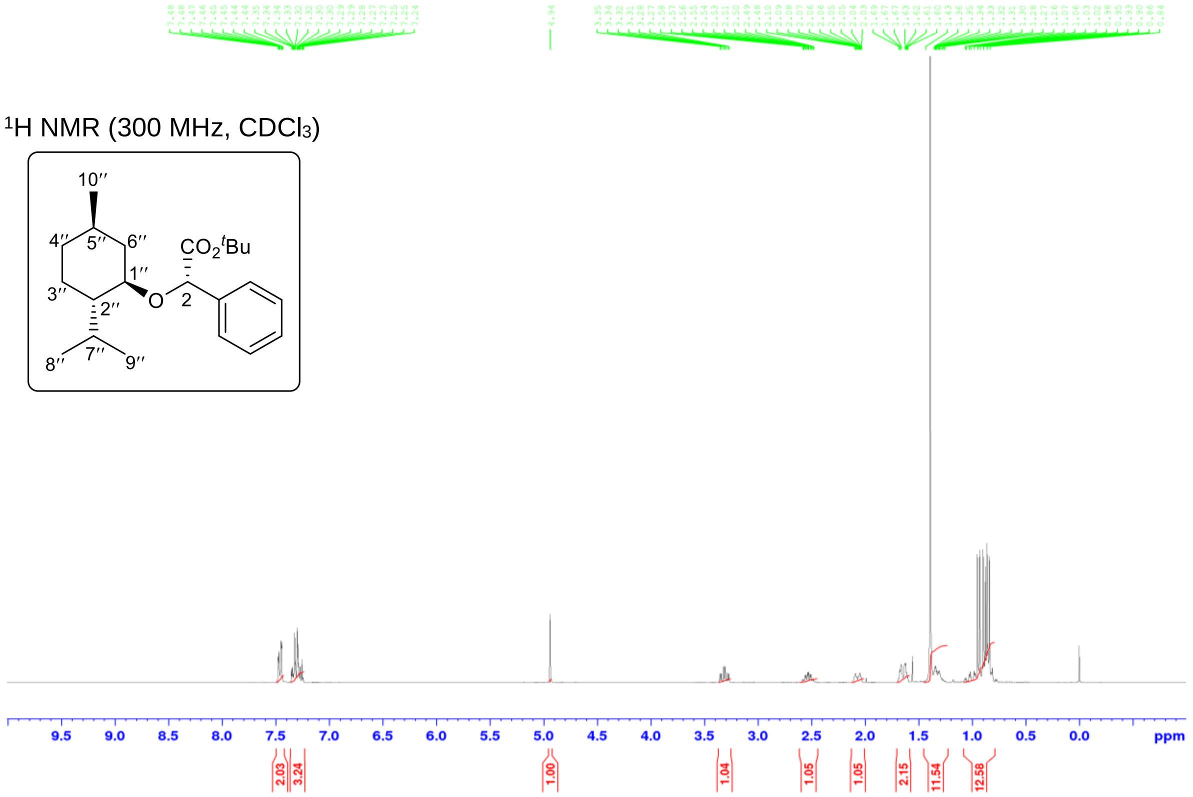

^{13}C NMR (75.5 MHz, $CDCl_3$)

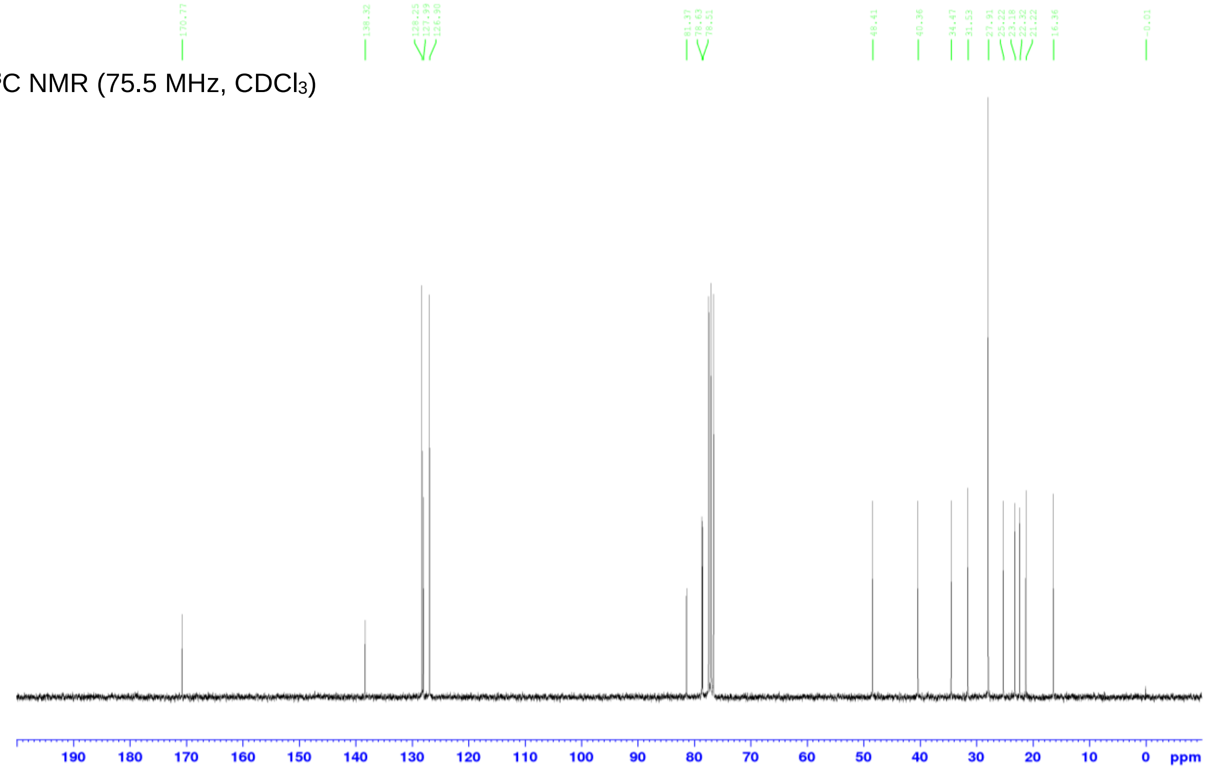

tert-Butyl 2-(1″R,2″R,5″S)-menthyloxy-2-(naphthalen-2-yl)acetate (7g)

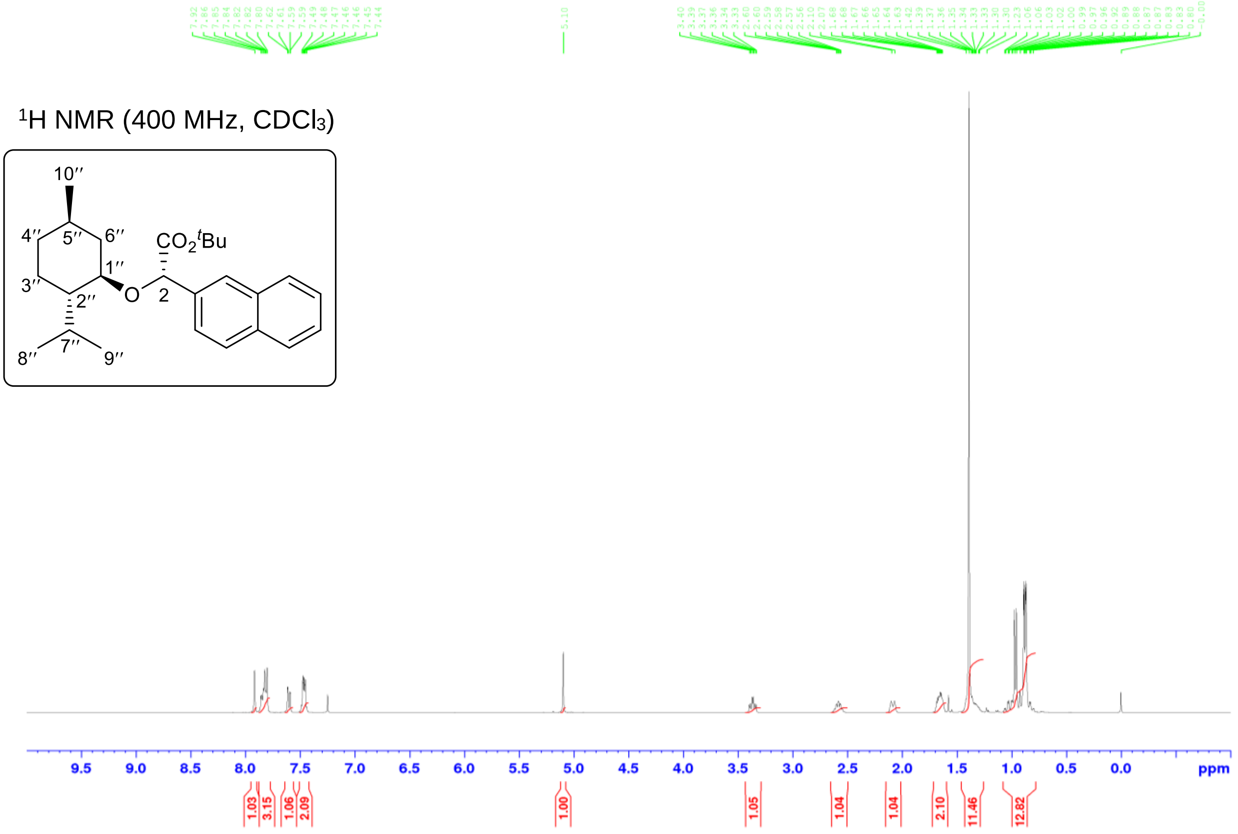

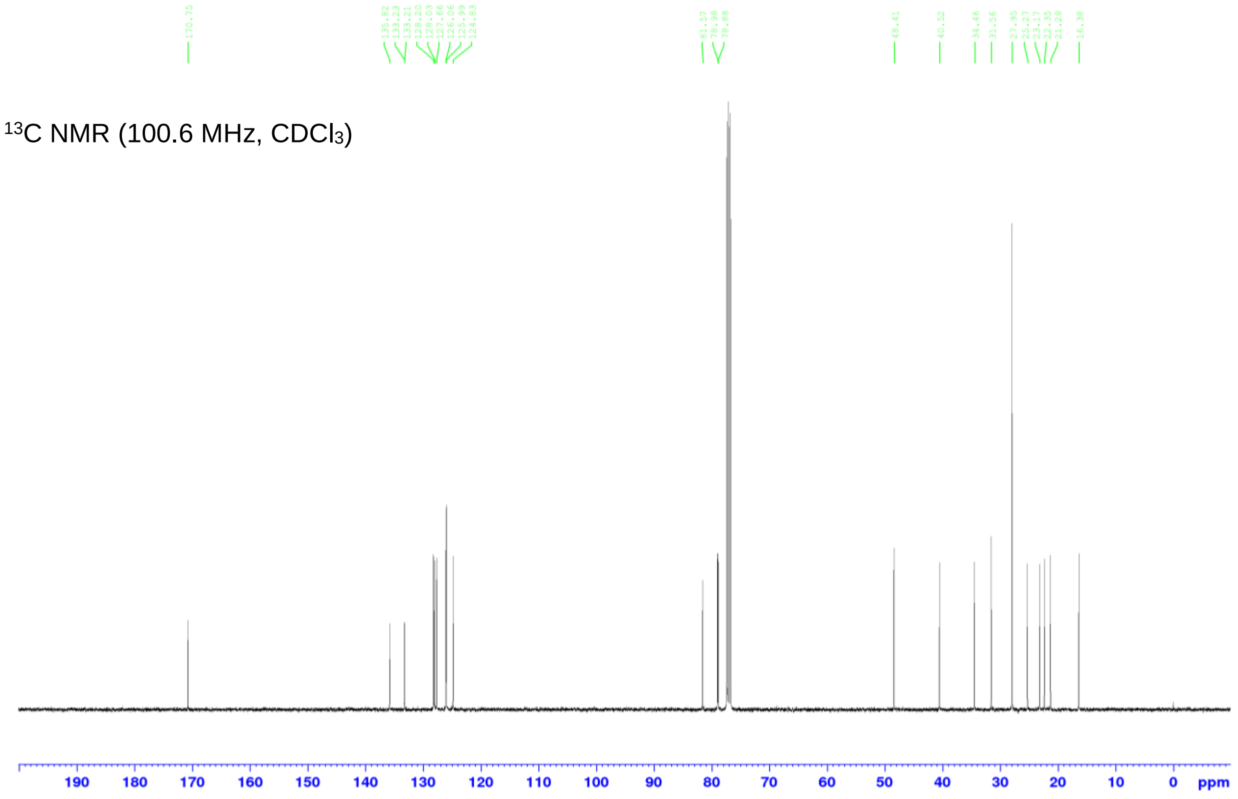

tert-Butyl (2*S*)-2-(4′-bromophenyl)-2-(1″*R*,2″*R*,5″*S*)-menthyloxyacetate (7h)

^{1}H NMR (400 MHz, CDCl$_3$)

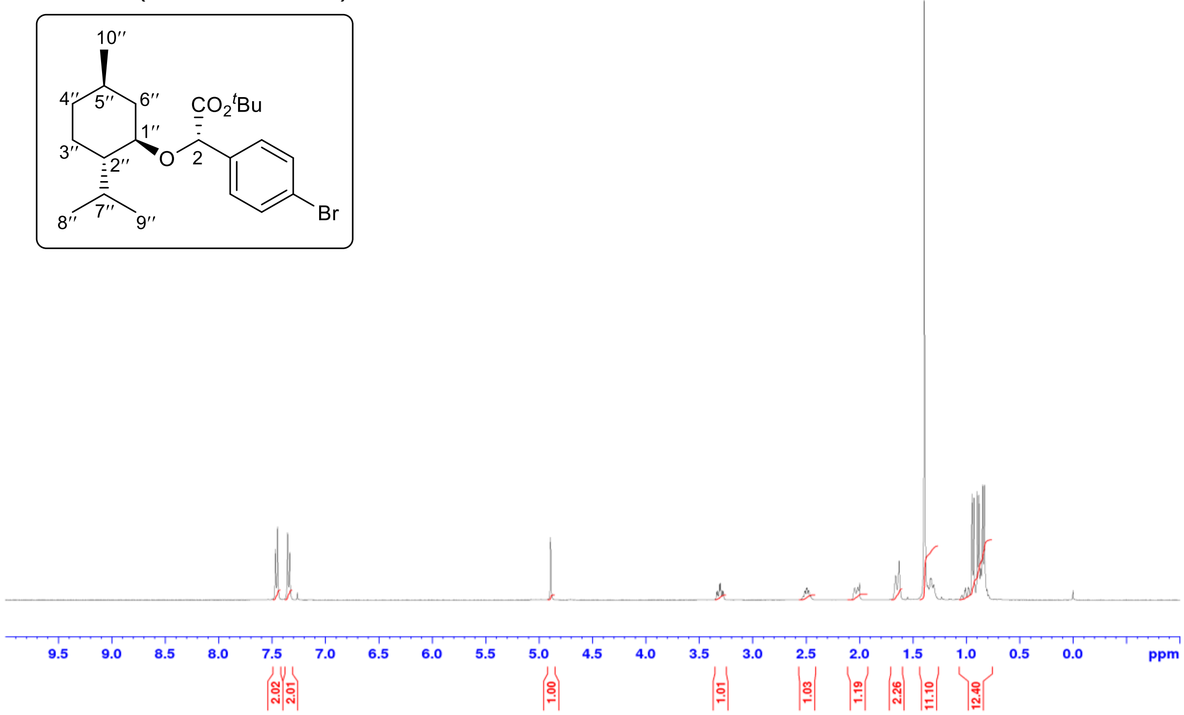

^{13}C NMR (100.6 MHz, CDCl$_3$)

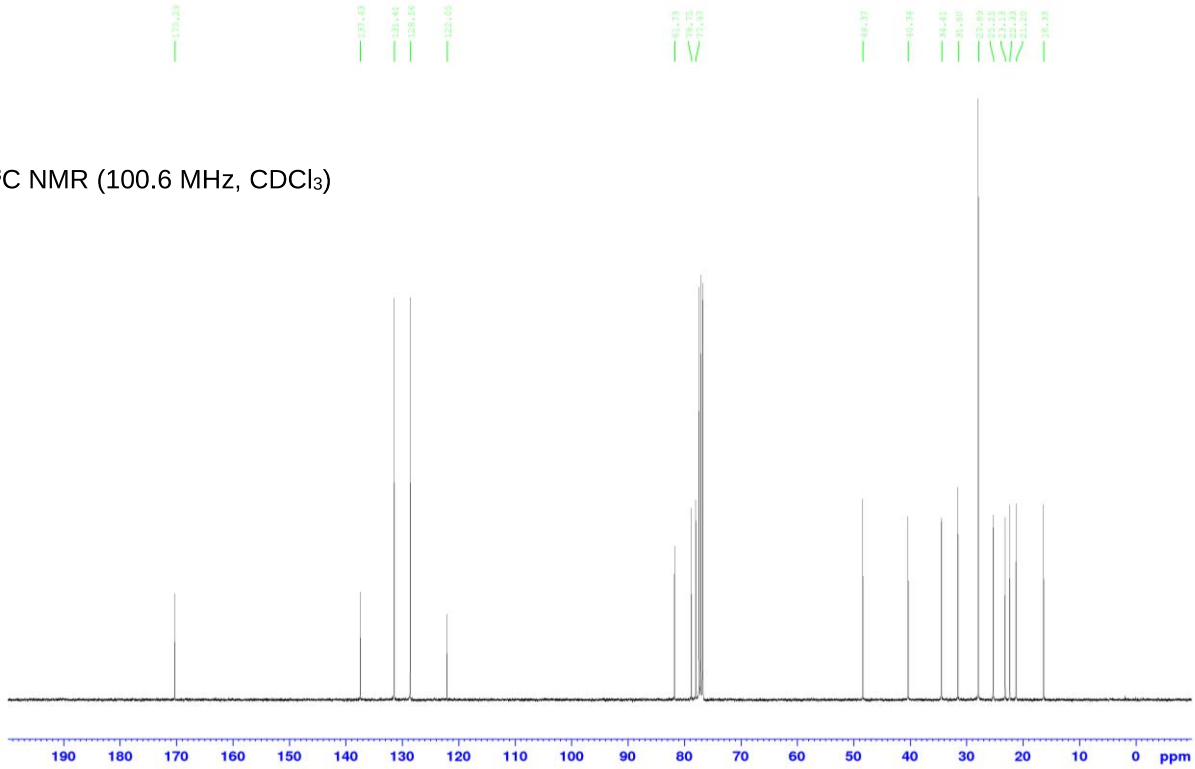

(2*S*)-2-(1″*R*,2″*R*,4″*S*)-Fenchyloxy-2-phenylacetic acid (8a)

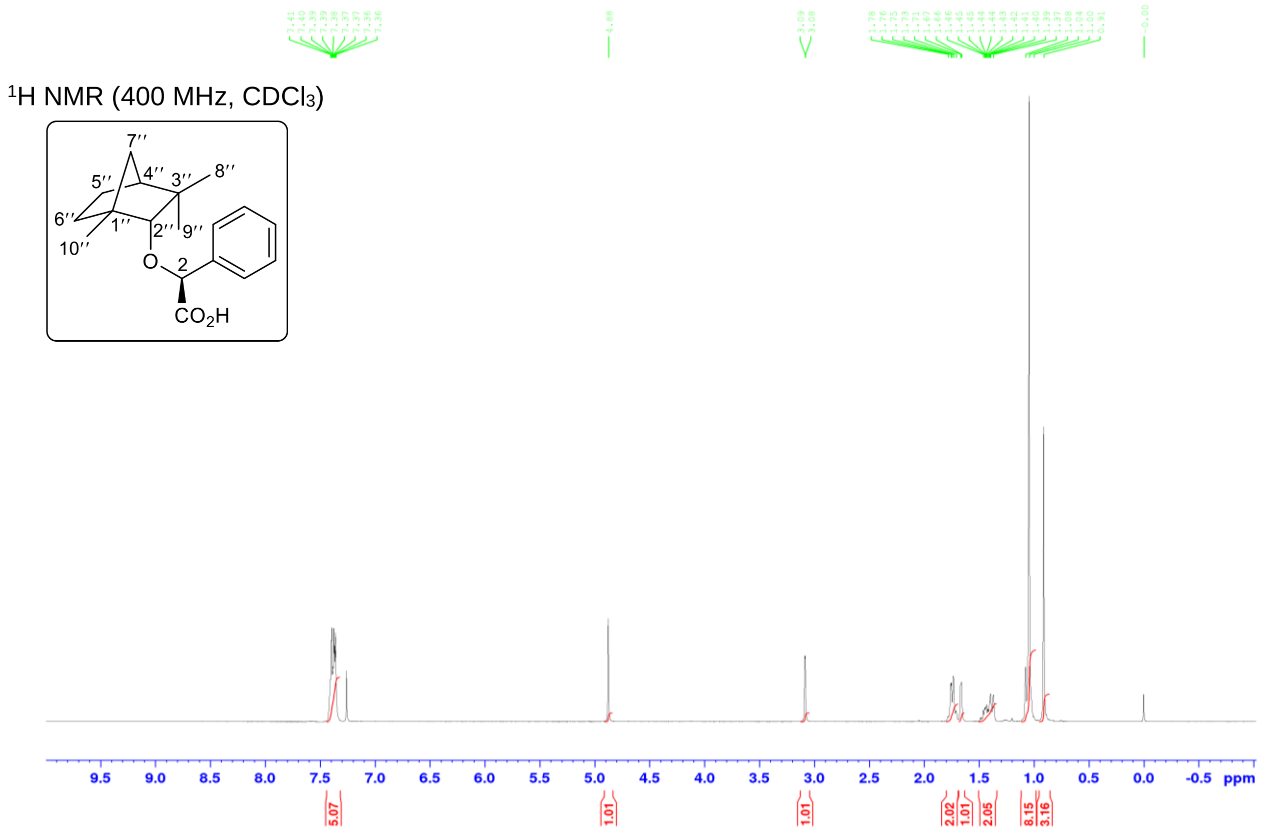

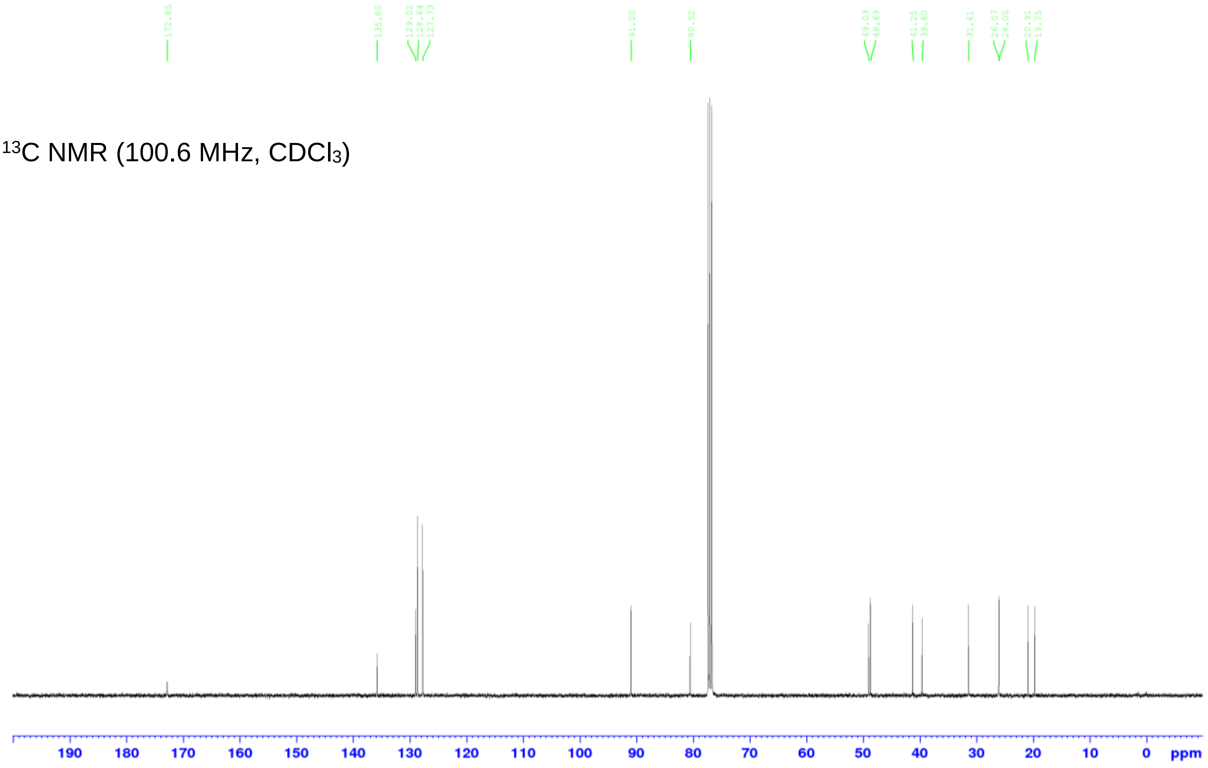

(2*S*)-2-(1″*R*,2″*R*,4″*S*)-Fenchyloxy-2-(naphthalen-2′-yl)acetic acid (8b)

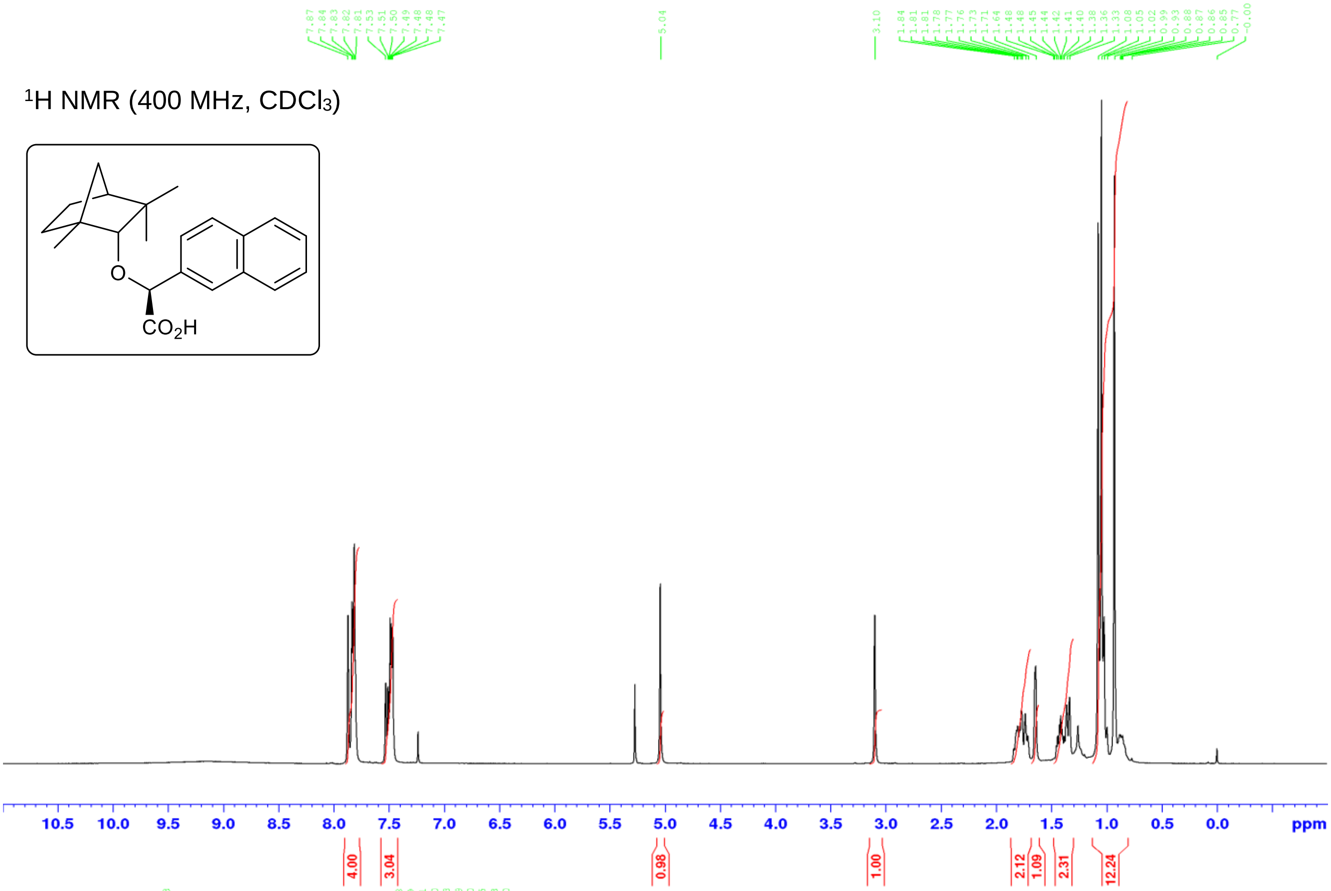

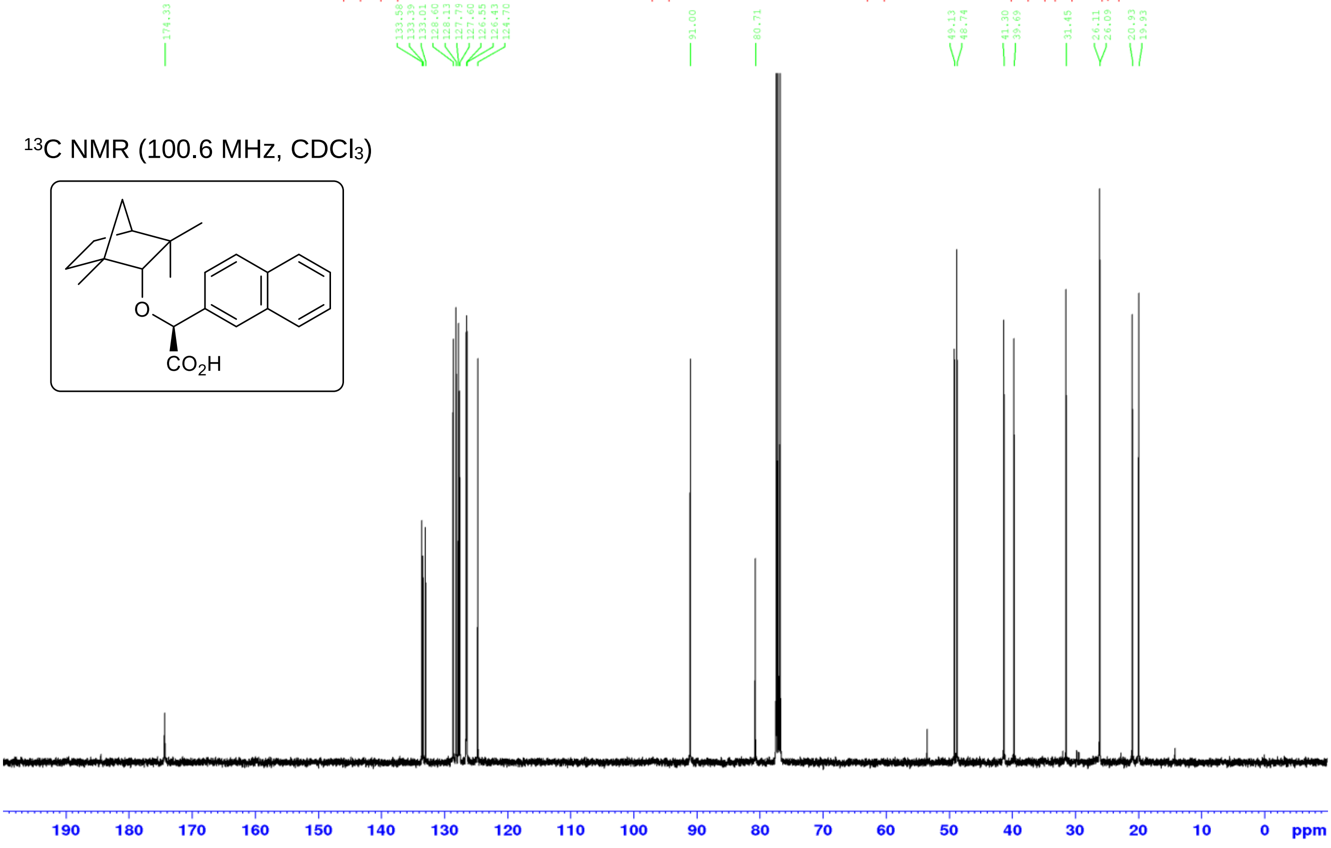

(2*S*)-2-(1″*R*,2″*R*,4″*S*)-Fenchyloxy-2-(naphthalen-1′-yl)acetic acid (8c)

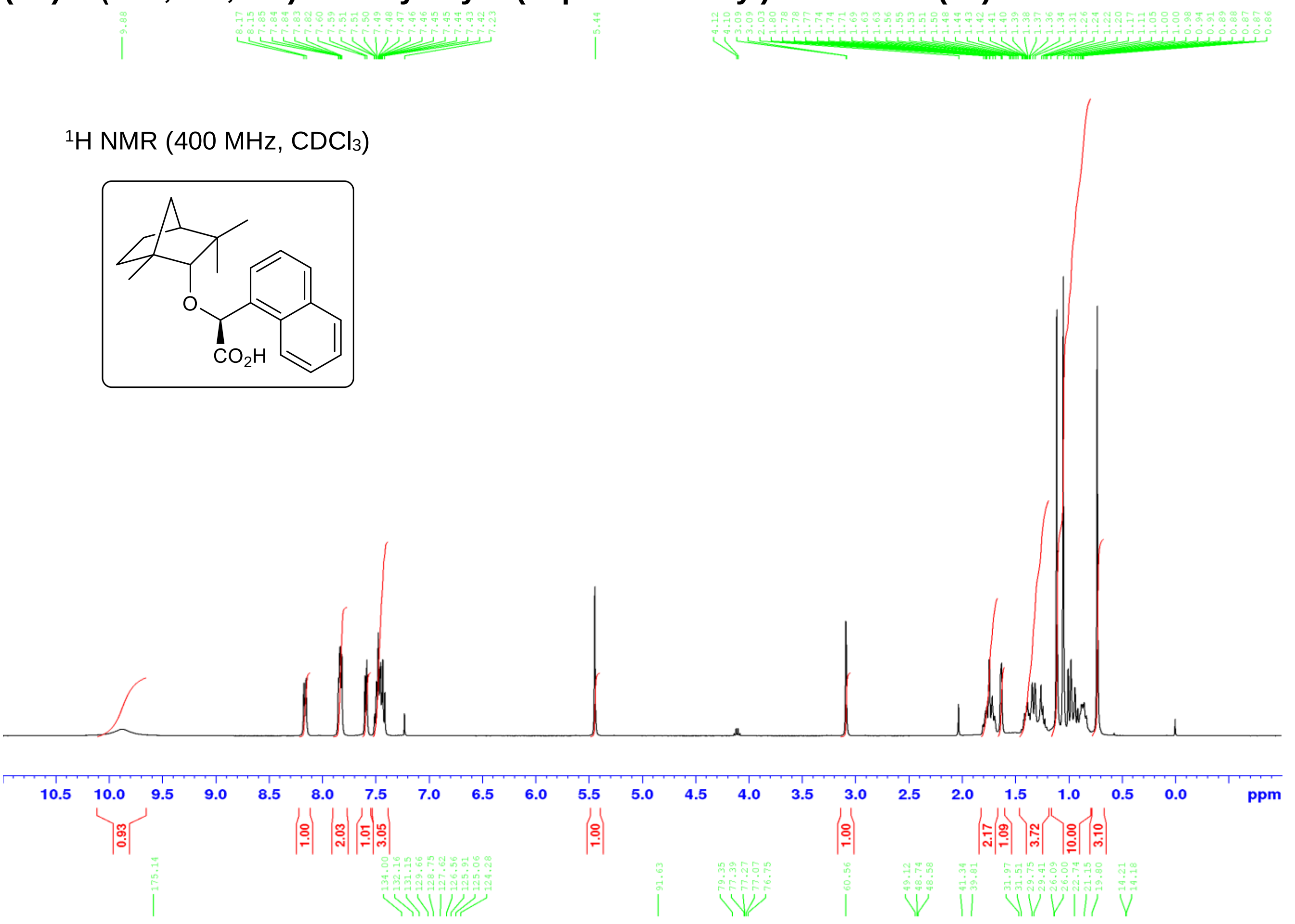

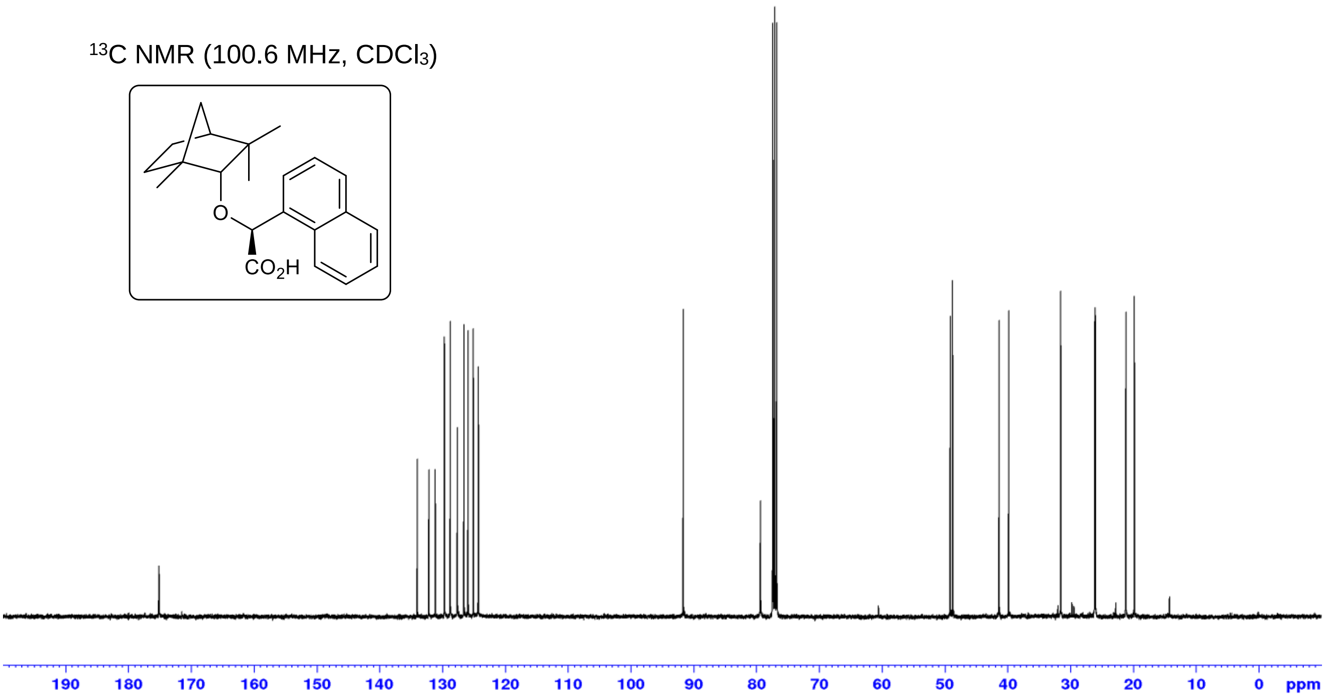

(2*S*)- 2-(4′-Bromophenyl)-2-(1″*R*,2″*R*,4″*S*)-fenchyloxyacetic acid (8d)

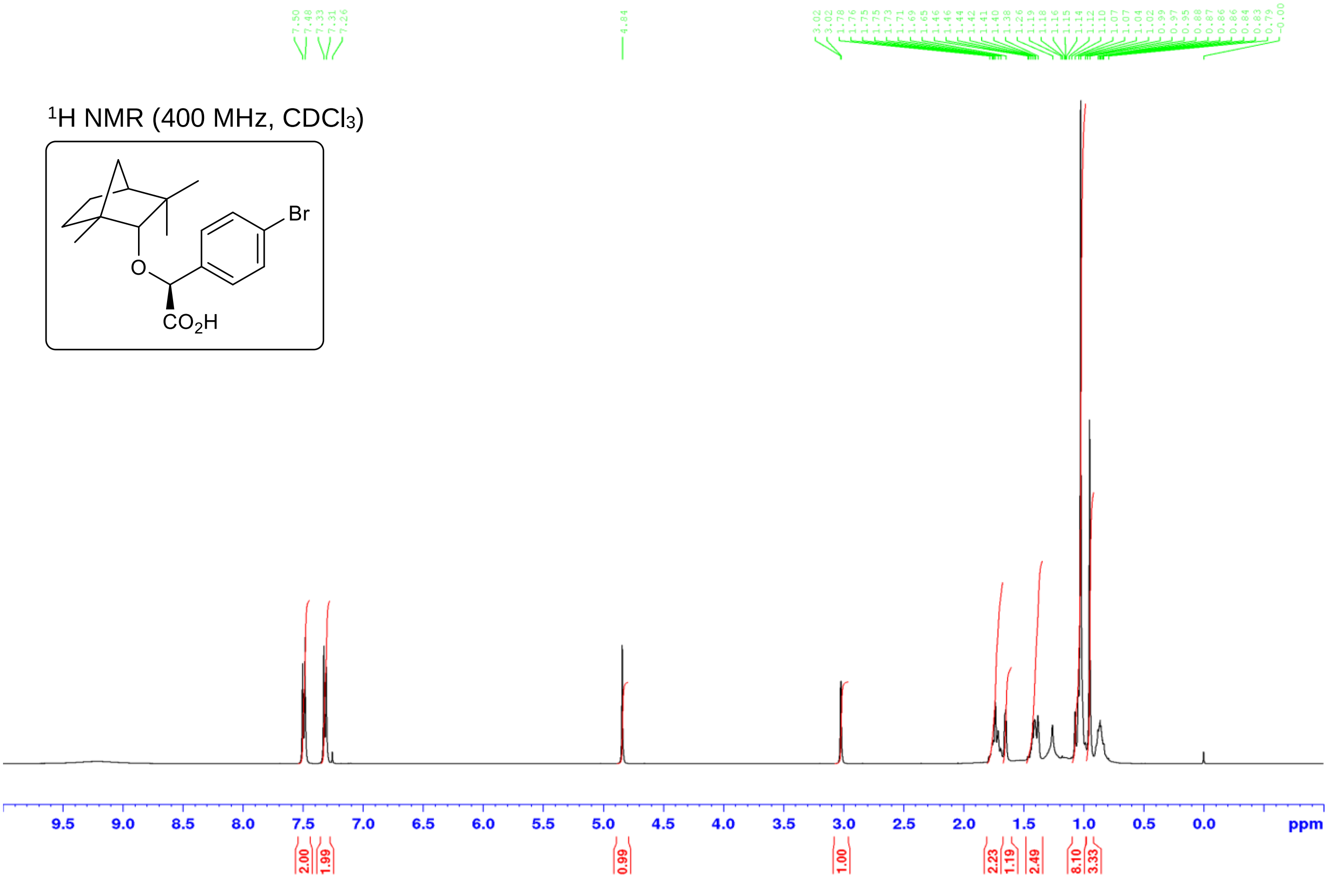

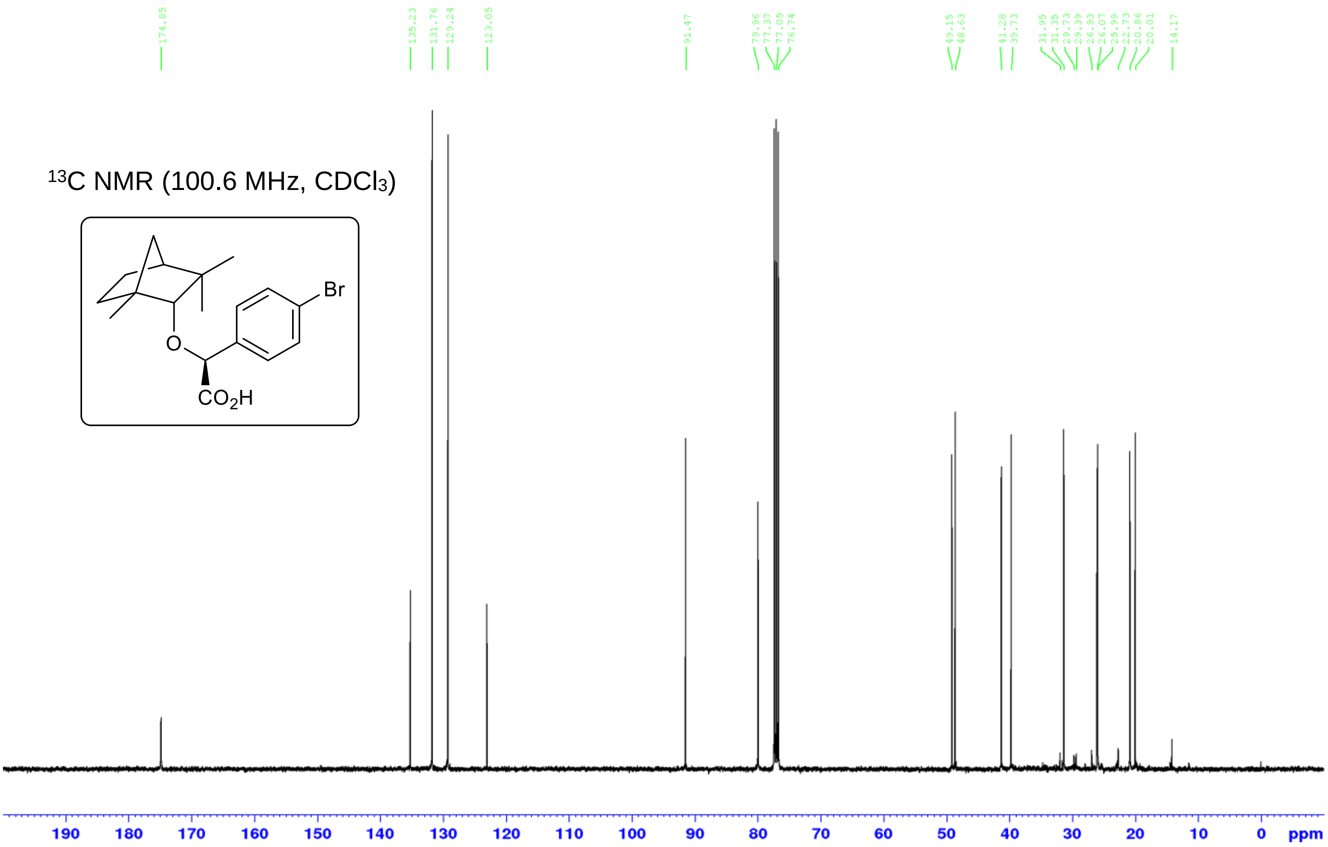

(2*S*)-2-(1″*R*,2″*R*,4″*S*)-Fenchyloxy-2-(4′-methoxy)phenylacetic acid (8e)

^{1}H NMR (400 MHz, $CDCl_3$)

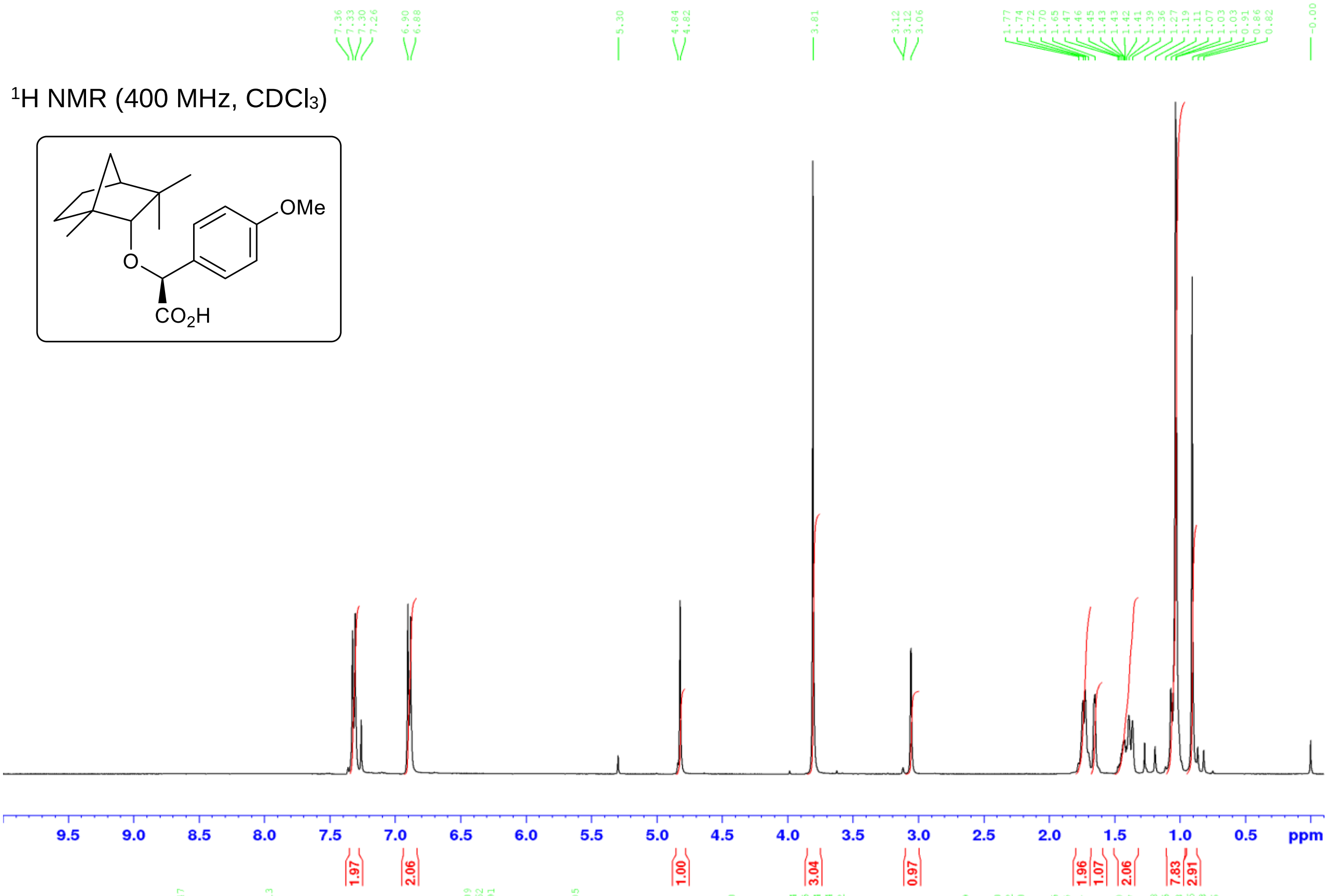

^{13}C NMR (100.6 MHz, $CDCl_3$)

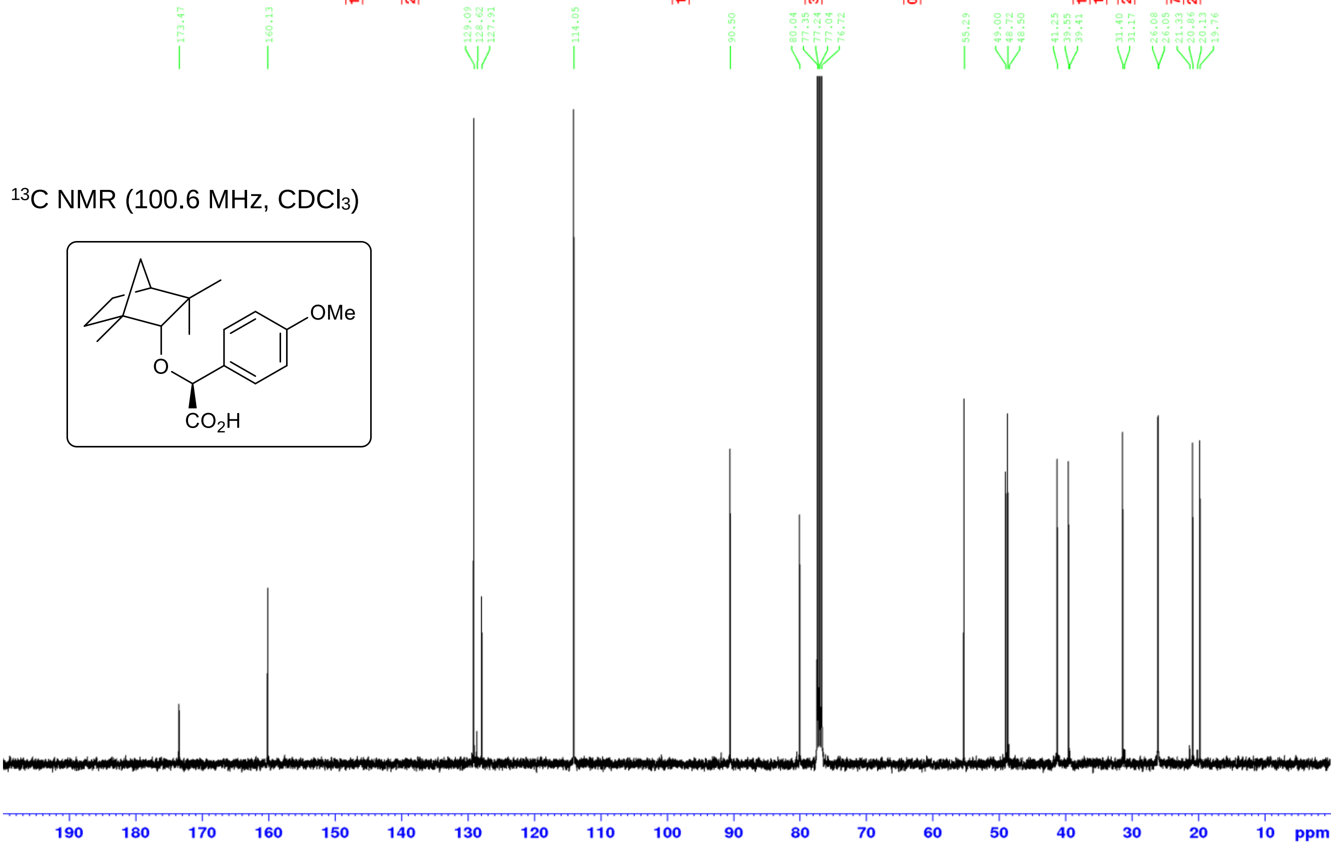

(2*S*)-2-(1″*R*,2″*S*,5″*S*)-Menthyloxy-2-phenylacetic acid (8f)

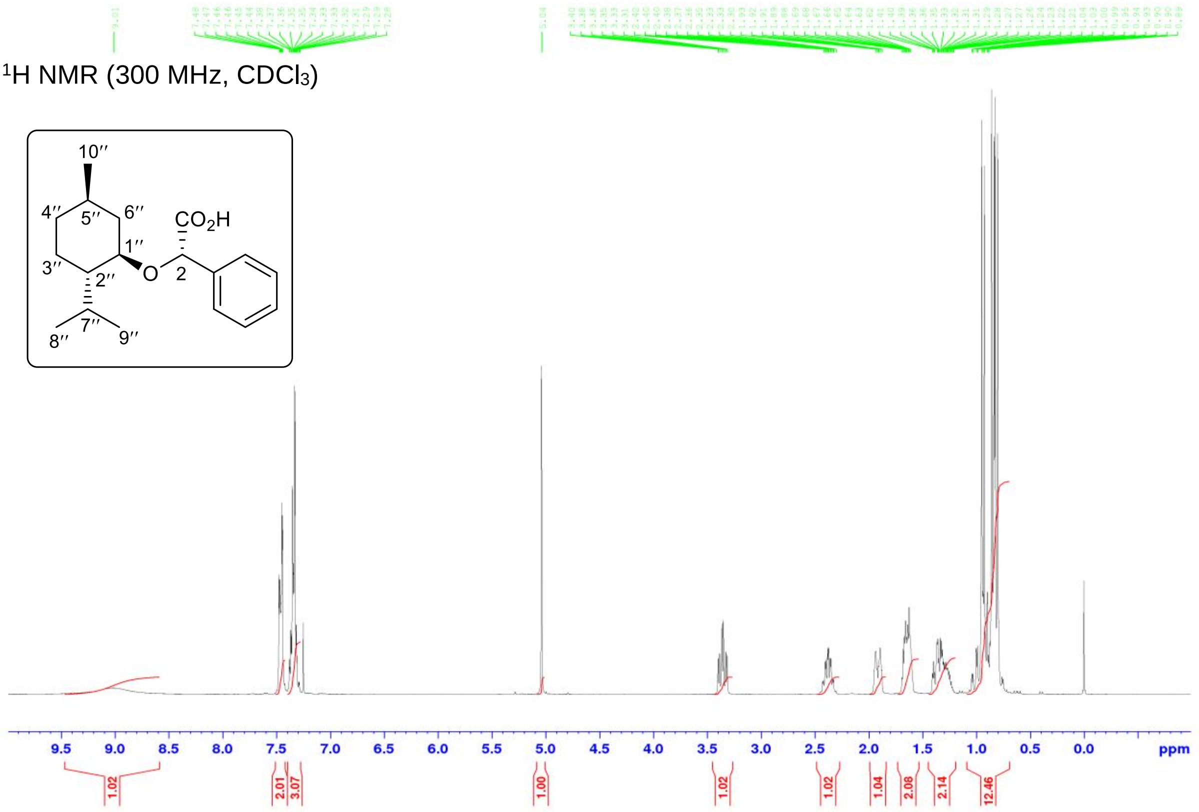

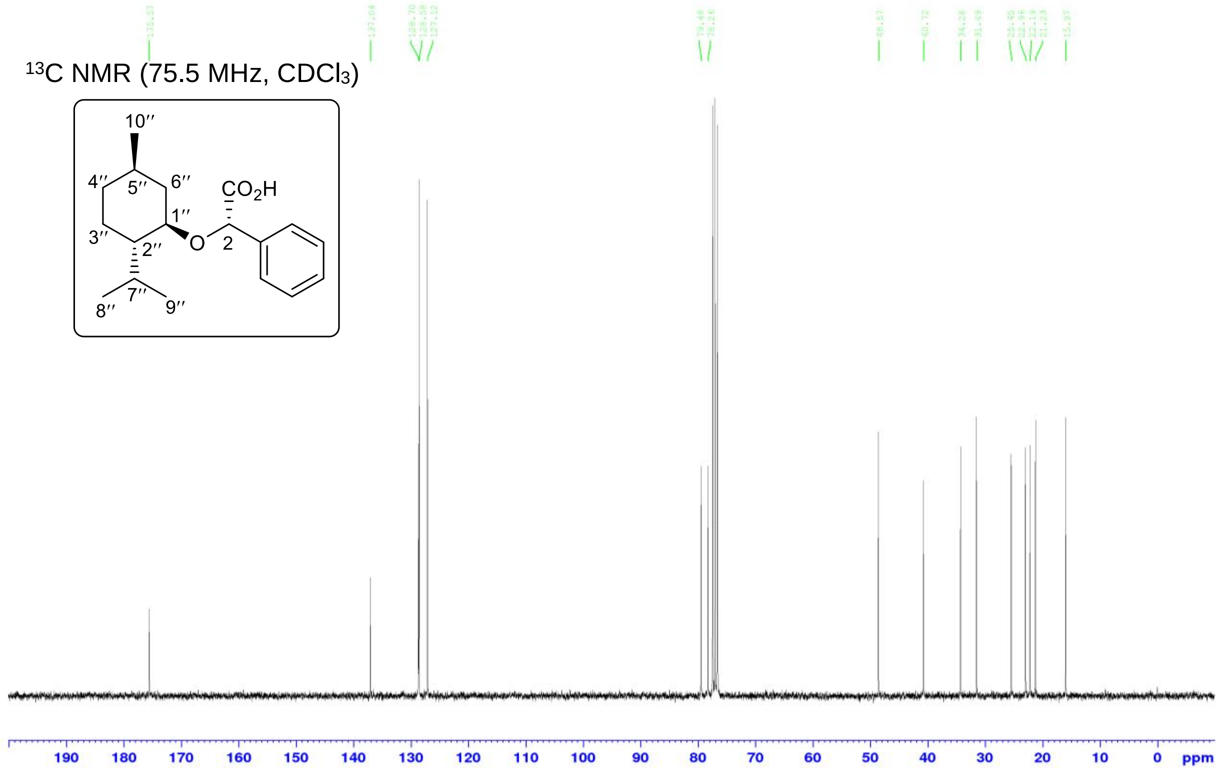

(2*S*)-2-(1″*R*,2″*S*,5″*R*)-Menthyloxy-2-(naphthalen-2′-yl)acetic acid (8g)

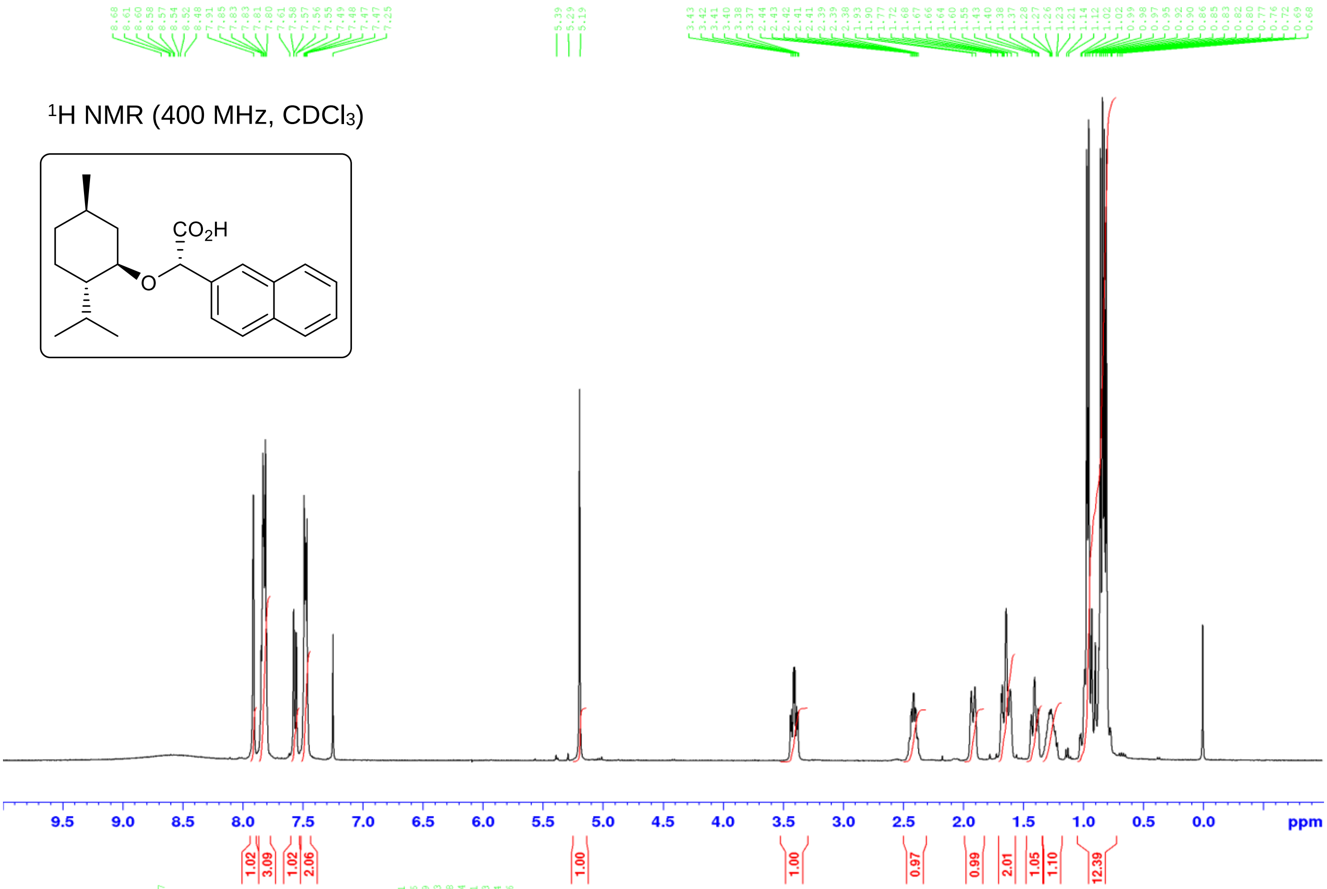

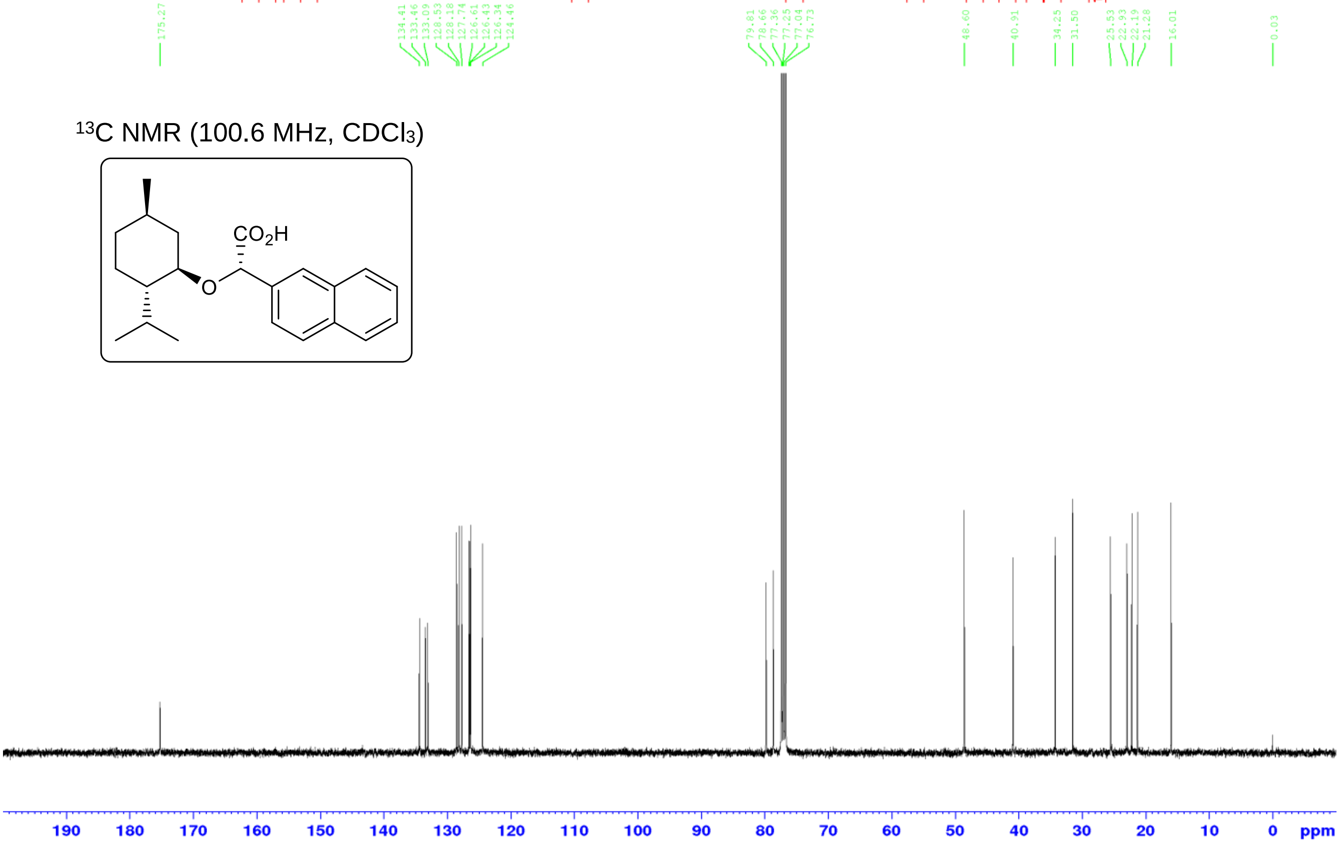

(2*S*)- 2-(4′-Bromophenyl)-2-(1″*R*,2″*S*,5″*R*)-menthyloxyacetic acid (8h)

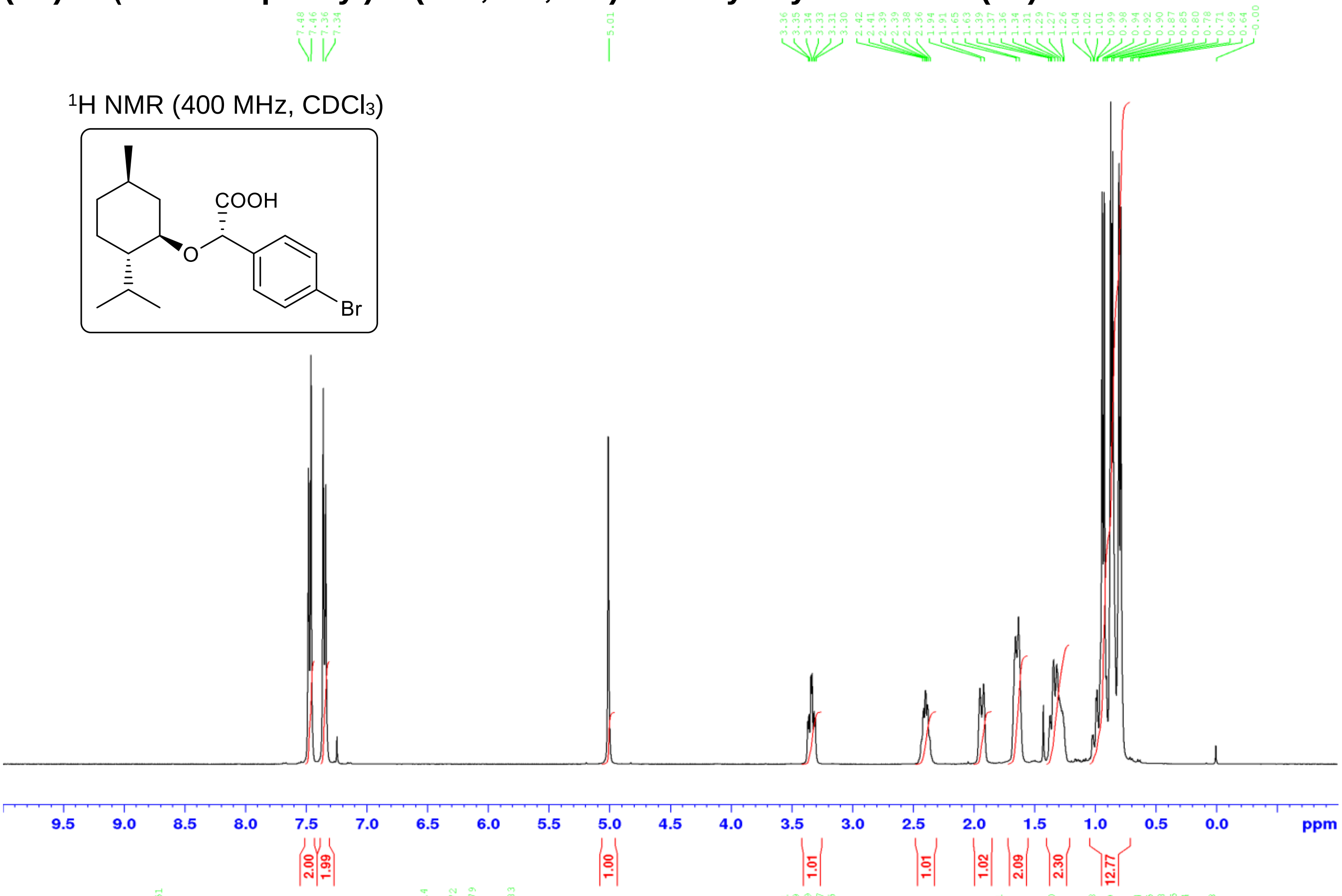

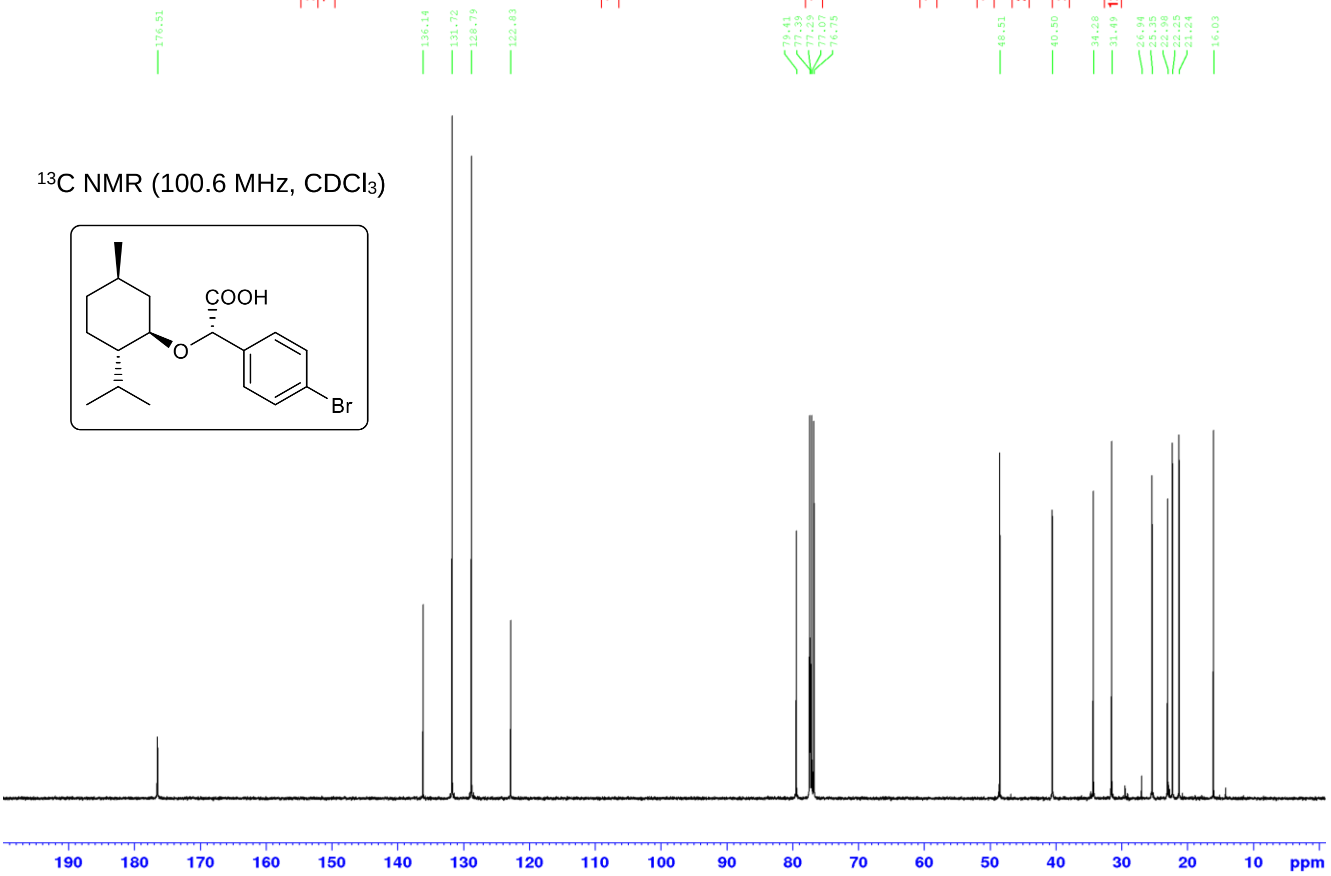

Dirhodium tetrakis [(2*S*)-2-(1″*R*,2″*R*,4″*S*)-fenchyloxy-2-phenylacetate] (2*S*-FPA) (9a)

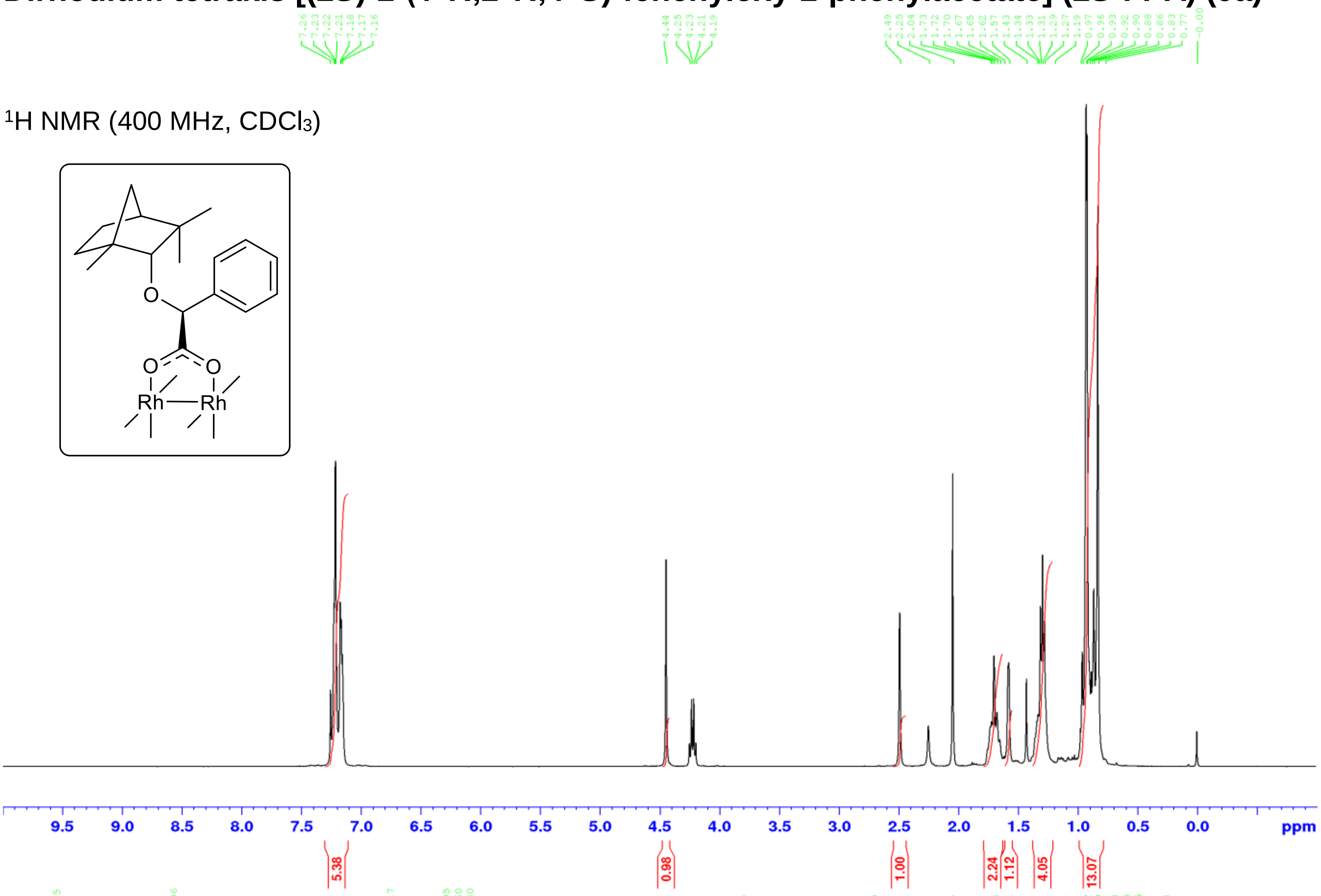

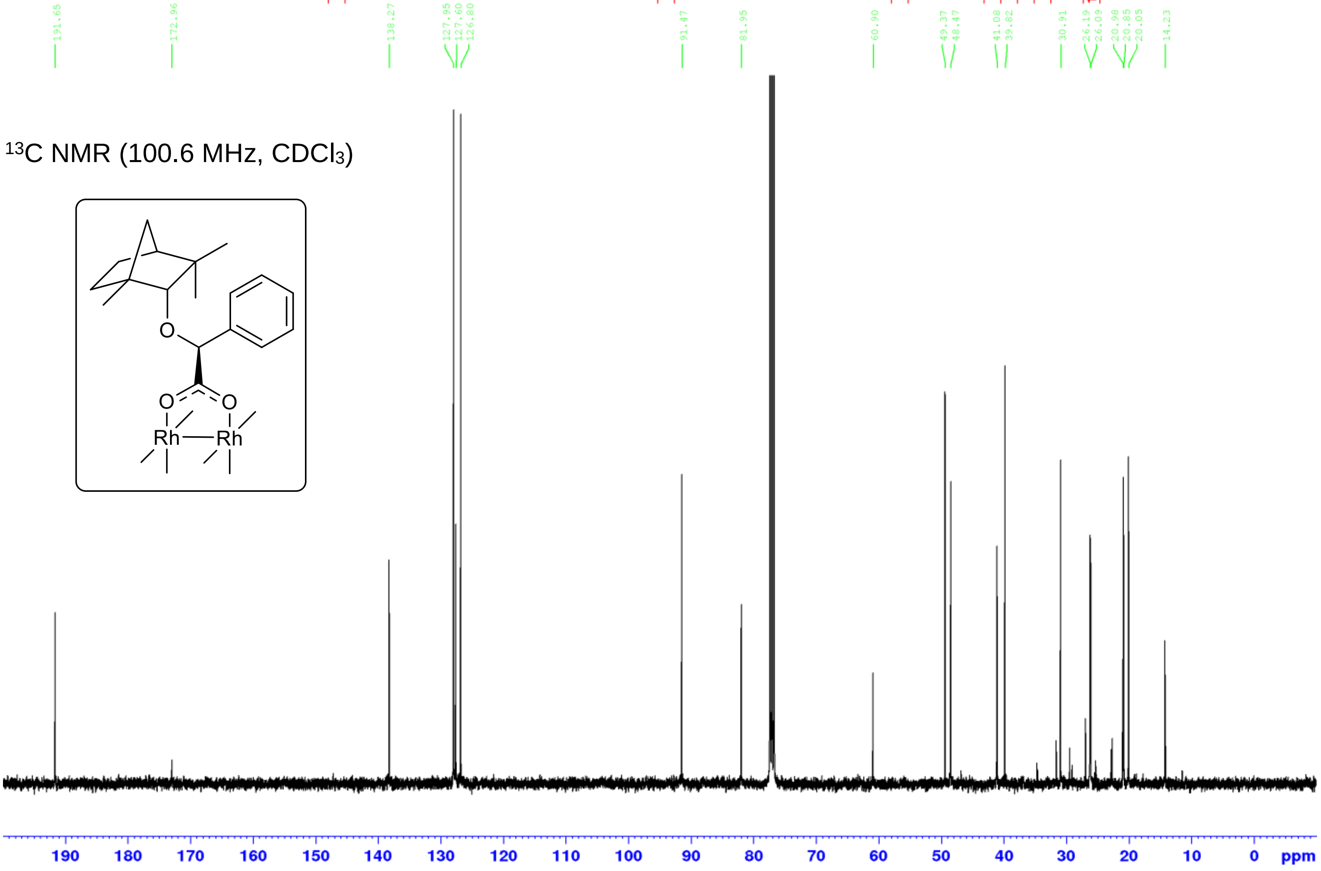

Dirhodium tetrakis [(2*S*)-2-(1″*R*,2″*R*,4″*S*)-fenchyloxy-2-(naphthalen-2′-yl)acetate] (2*S*-F-2′-NA) (9b)

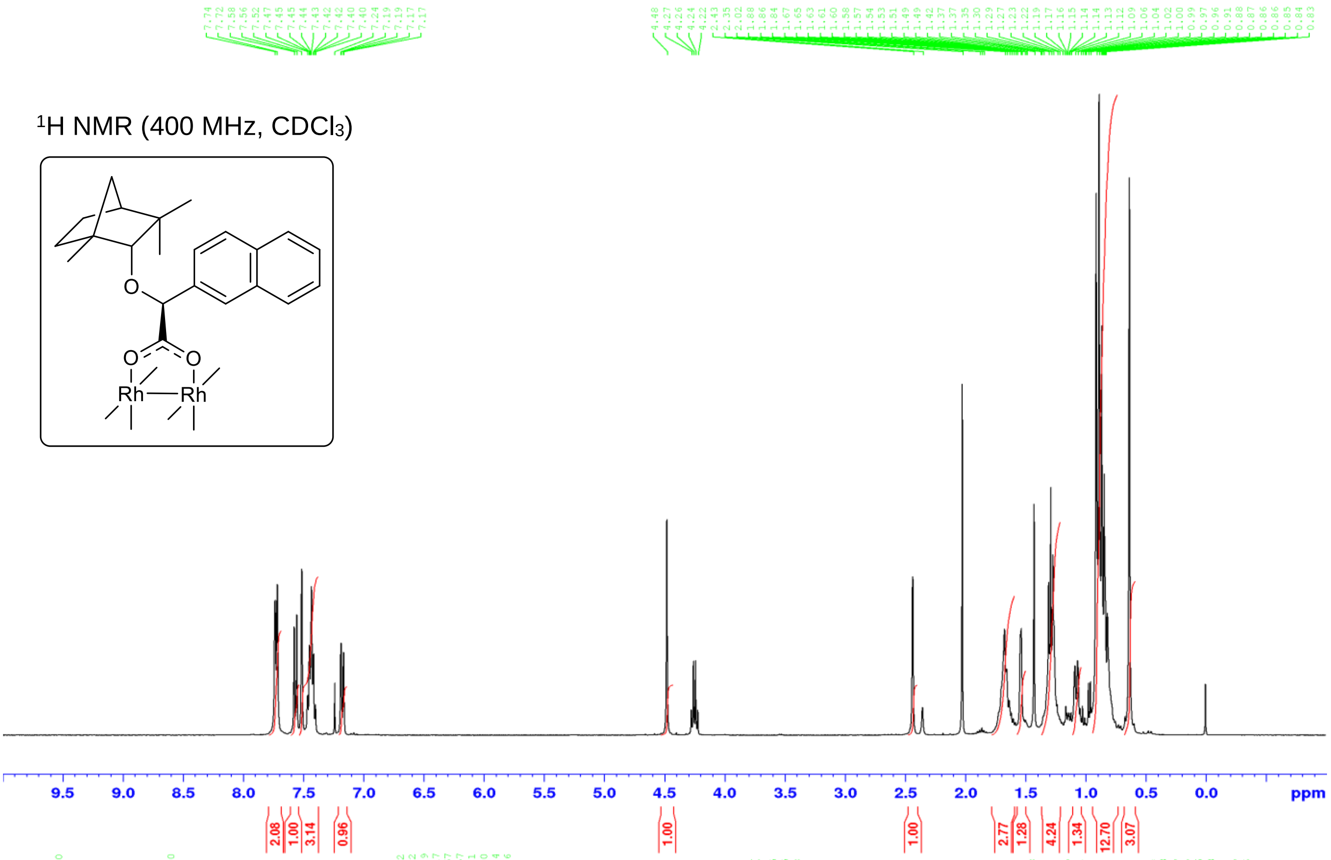

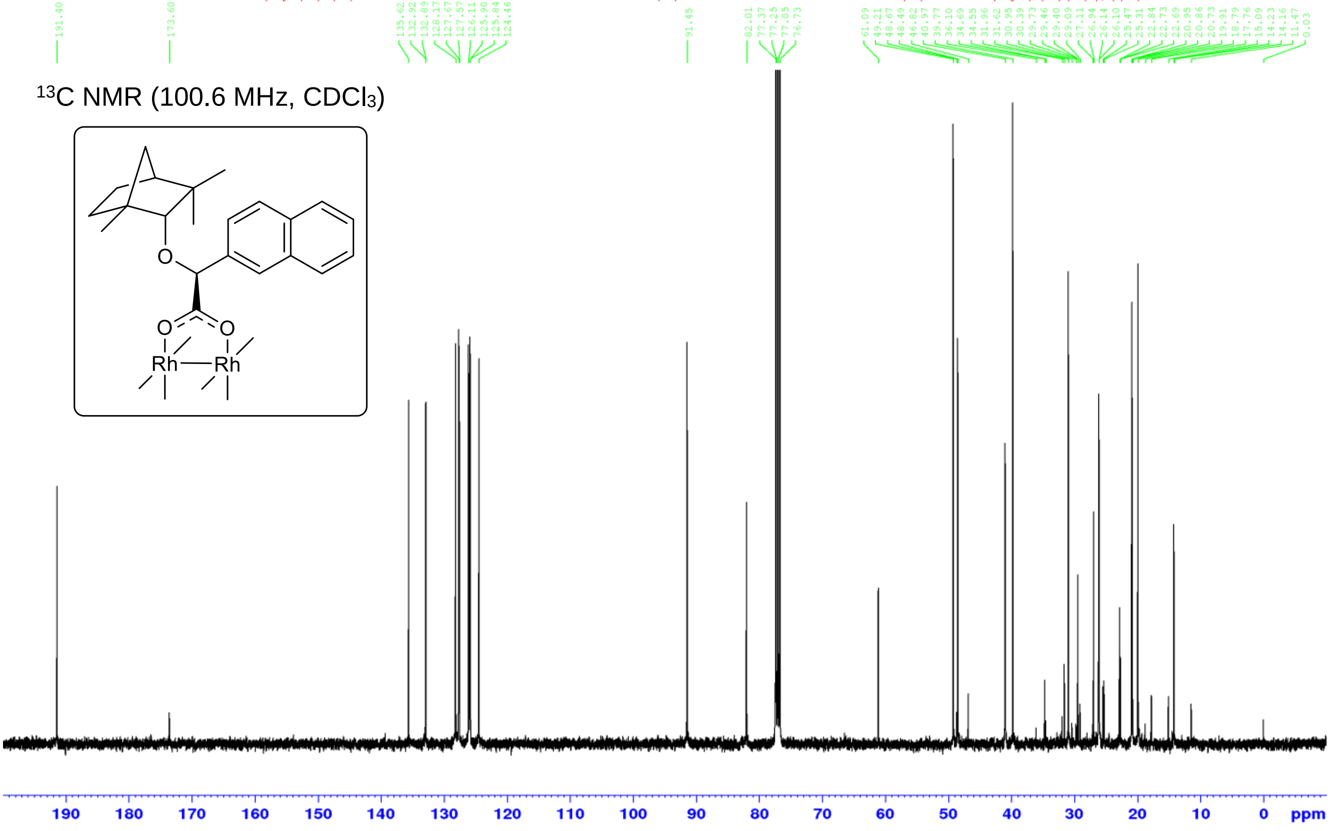

Dirhodium tetrakis [(2*S*)-2-(1″*R*,2″*R*,4″*S*)-fenchyloxy-2-(naphthalen-1′-yl)acetate] (2*S*-F-1′-NA) (9c)

^{1}H NMR (400 MHz, $CDCl_3$)

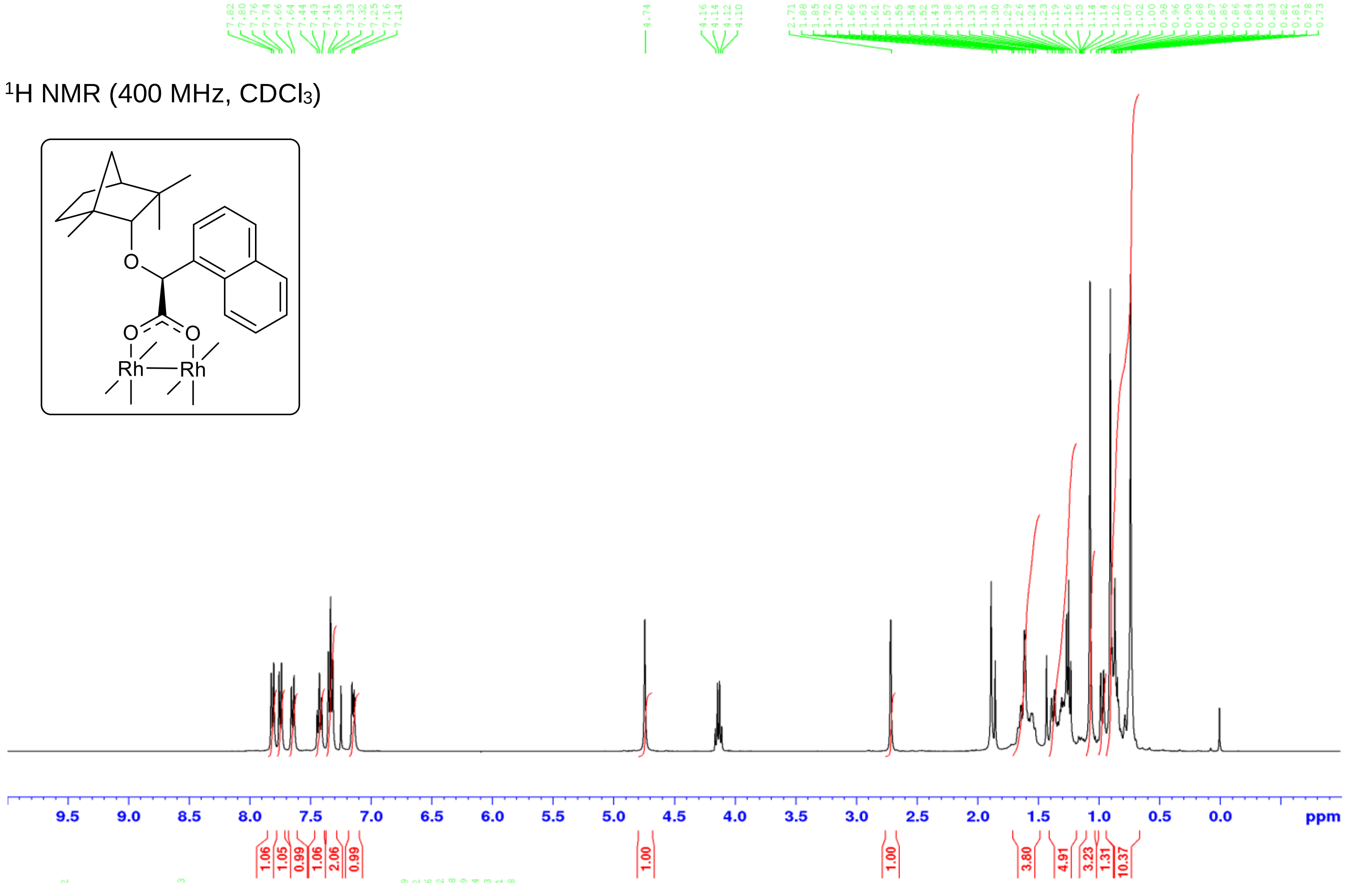

^{13}C NMR (100.6 MHz, $CDCl_3$)

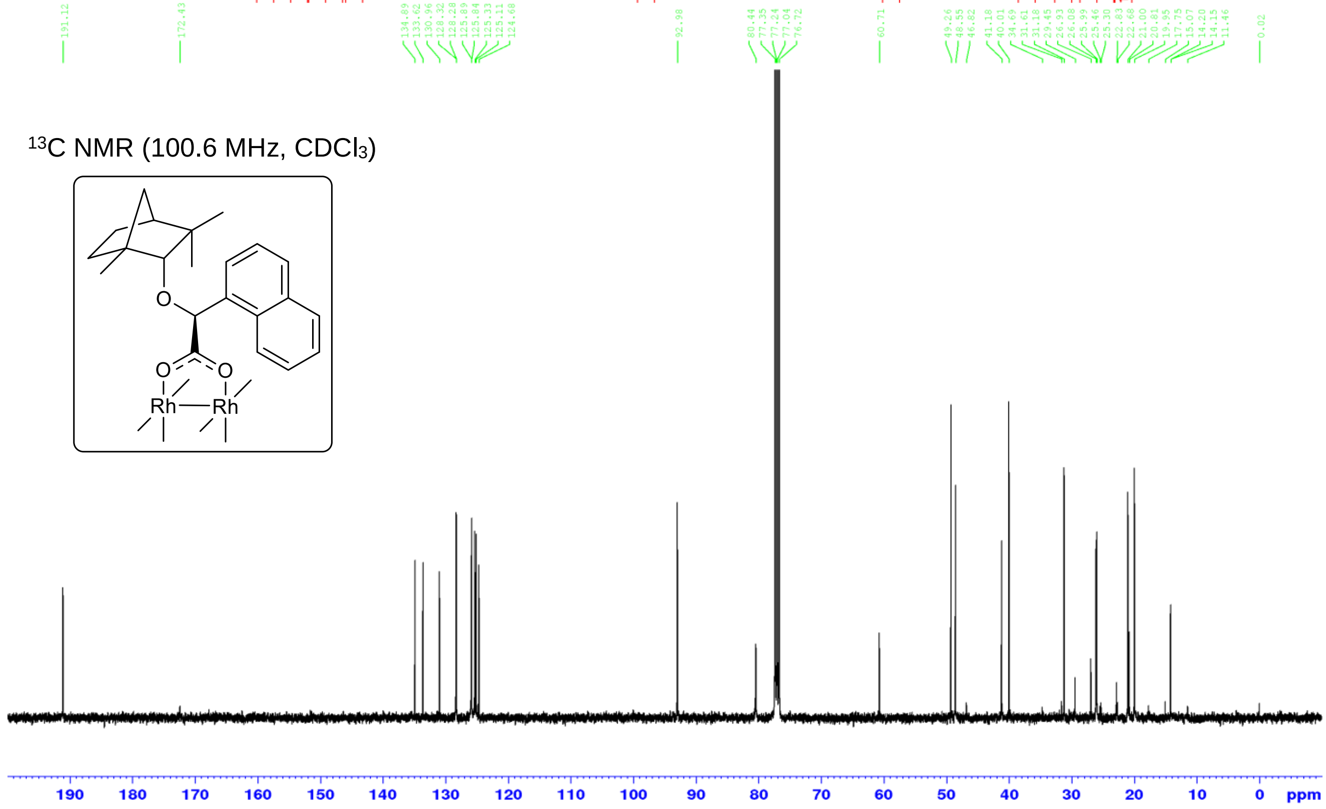

Dirhodium tetrakis [(2*S*)-2-(4′-bromophenyl)-2-(1″*R*,2″*R*,4″*S*)-fenchyloxyacetate] (2*S*-FBrPA) (9d)

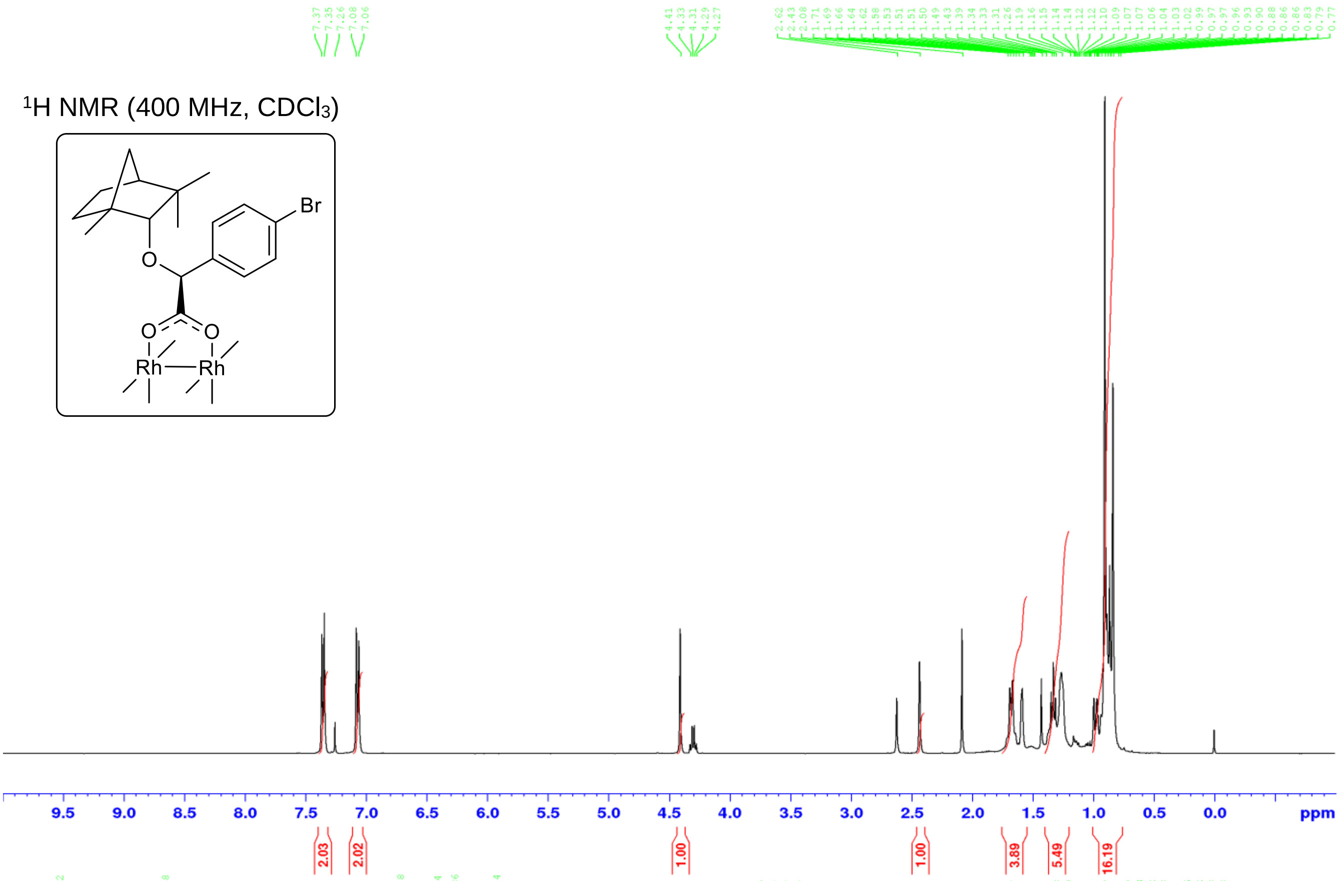

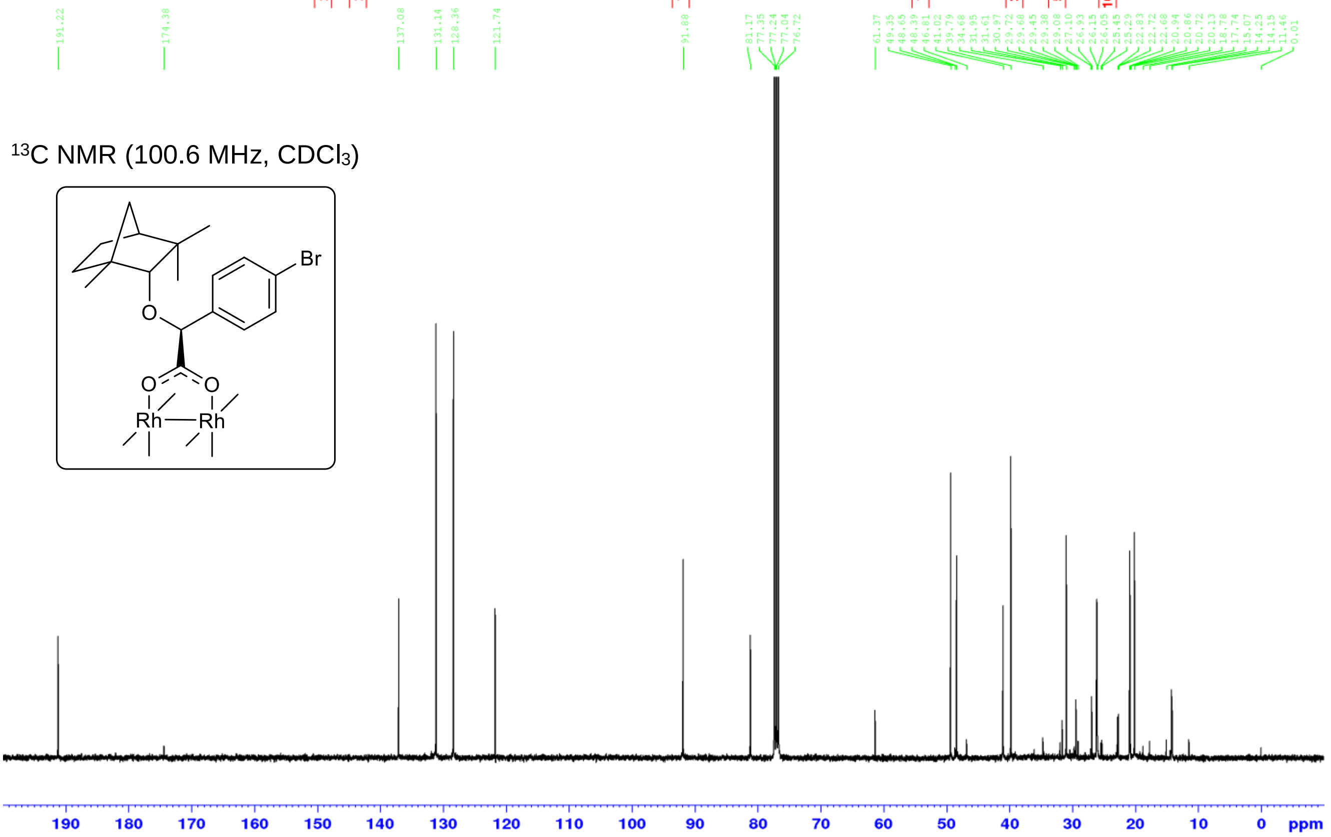

Dirhodium tetrakis [(2*S*)-2-(1″*R*,2″*R*,4″*S*)-fenchyloxy-2-(4′-methoxyphenyl)acetate] (2*S*-FMeOPA) (9e)

^{1}H NMR (400 MHz, $CDCl_3$)

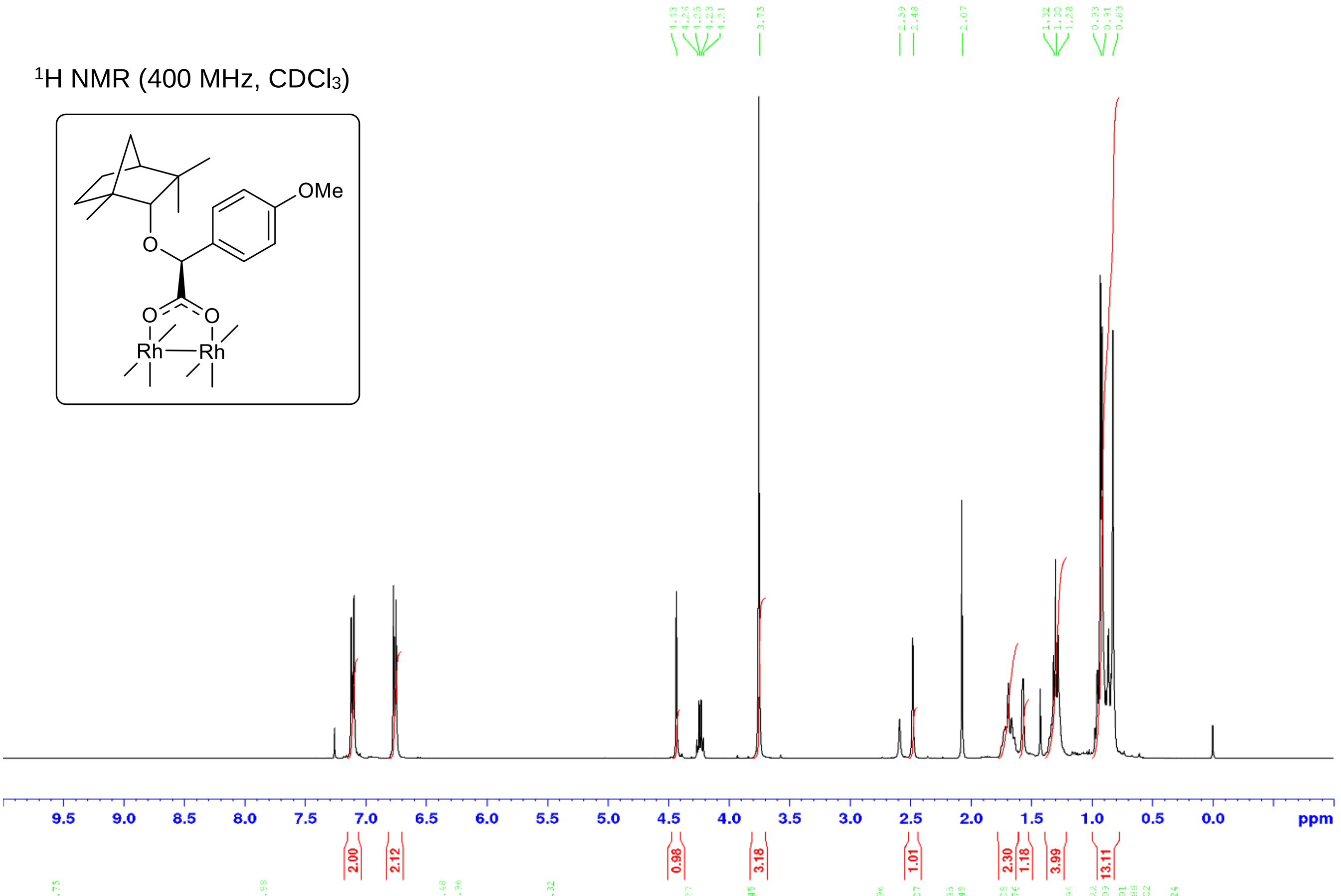

^{13}C NMR (100.6 MHz, $CDCl_3$)

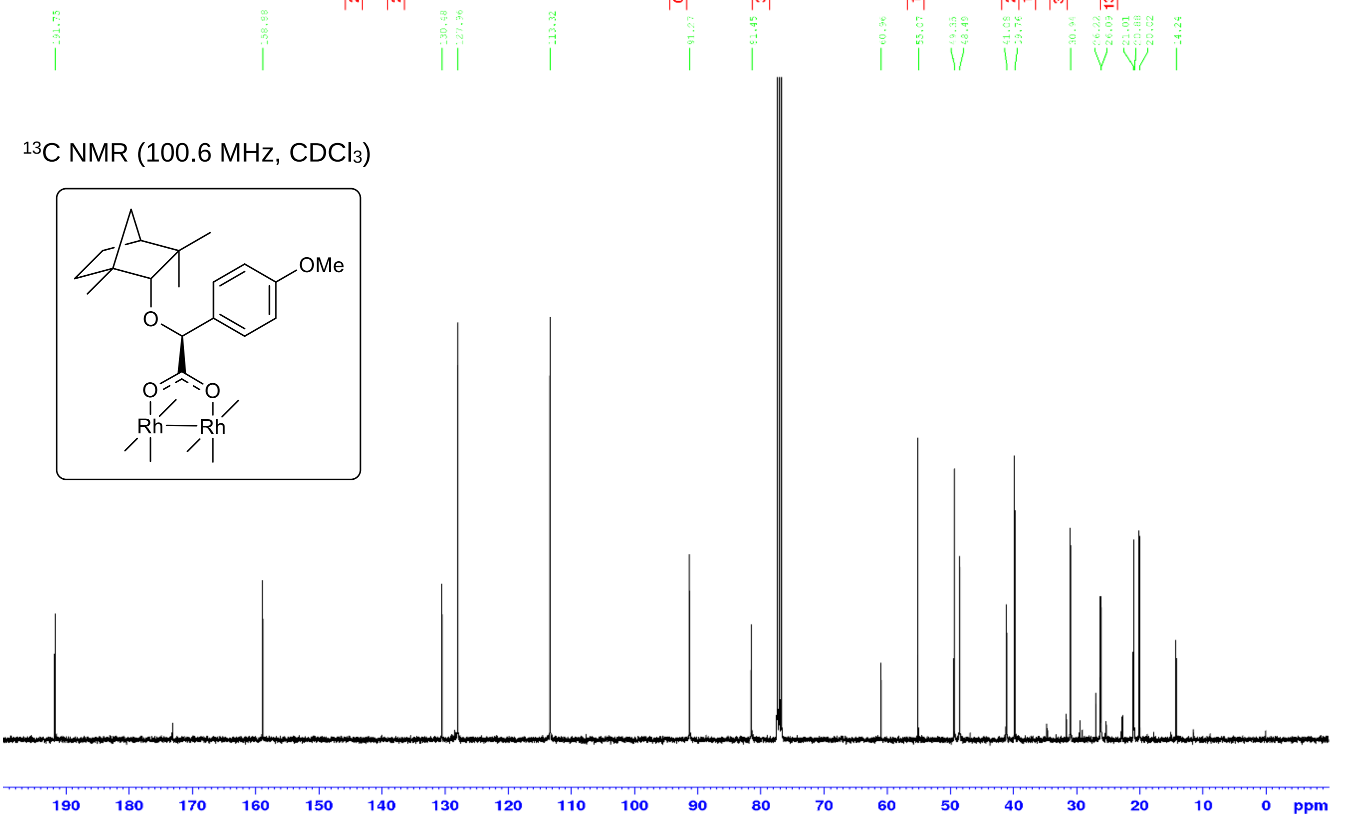

Dirhodium tetrakis [(2*S*)-2-(1″*R*,2″*S*,5″*R*)-menthyloxy-2-phenylacetate] (2*S*-MPA) (9f)

^{1}H NMR (300 MHz, $CDCl_3$)

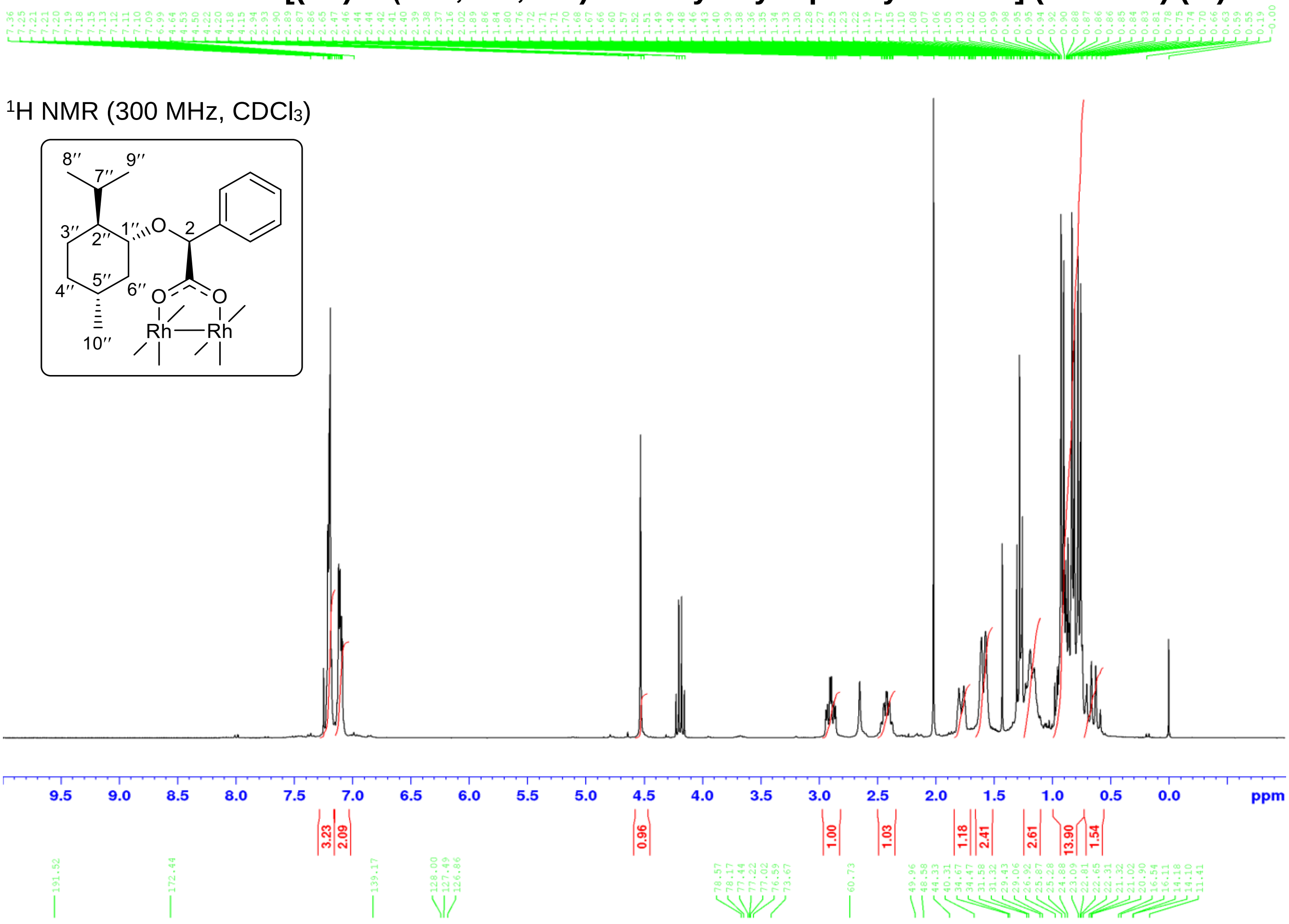

^{13}C NMR (75.5 MHz, $CDCl_3$)

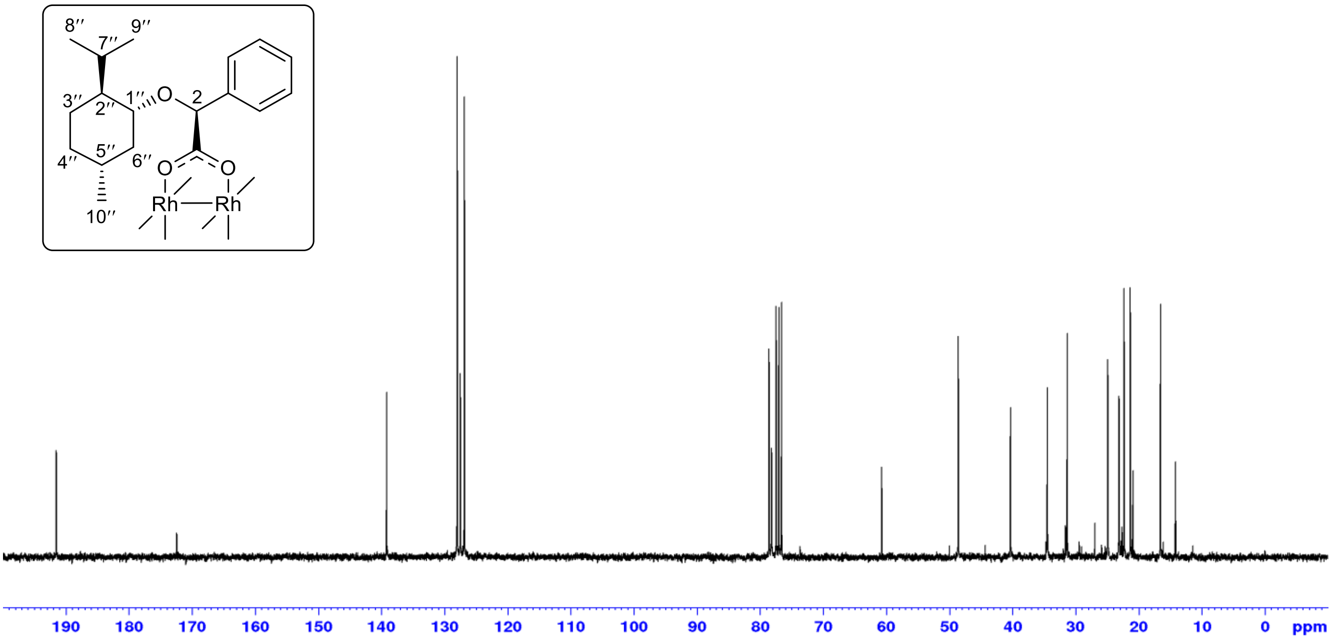

Dirhodium tetrakis [(2*S*)-2-(1″*R*,2″*S*,5″*R*)-menthyloxy-2-(naphthalen-2′-yl)acetate] (2*S*-M-2′-NA) (9g)

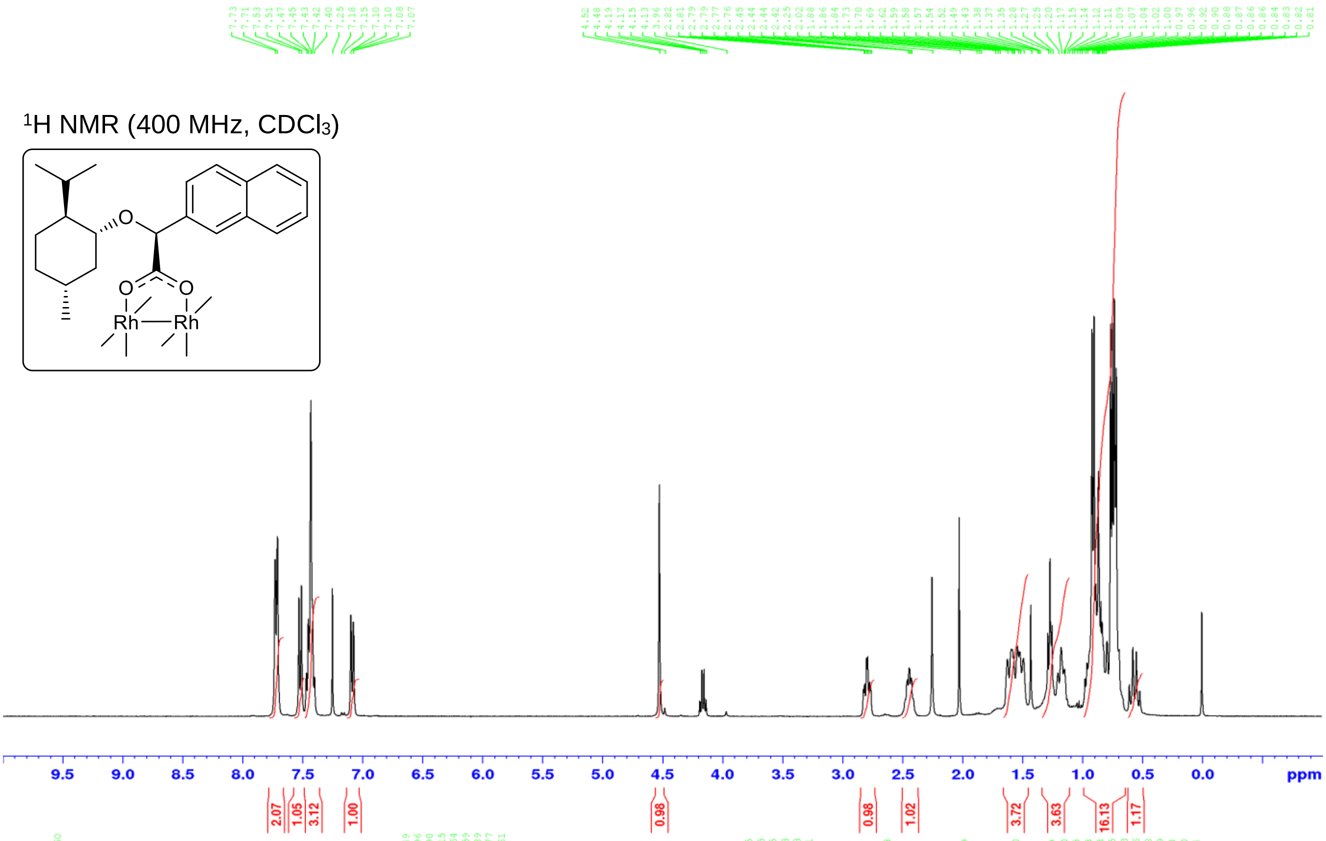

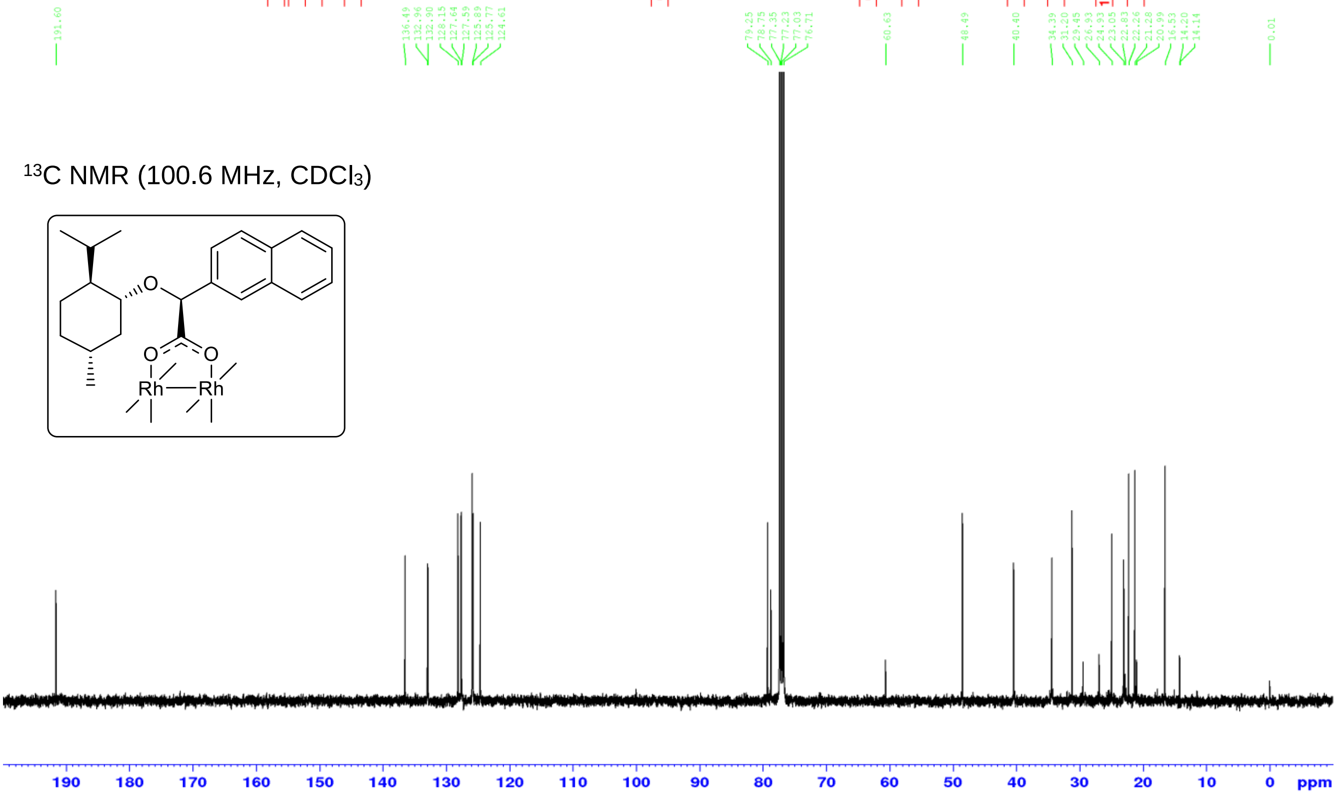

Dirhodium tetrakis [(2*S*)-2-(4′-bromophenyl)-2-(1″*R*,2″*S*,5″*R*)-menthyloxyacetate] (2*S*-MBrPA) (9h)

^{1}H NMR (400 MHz, $CDCl_3$)

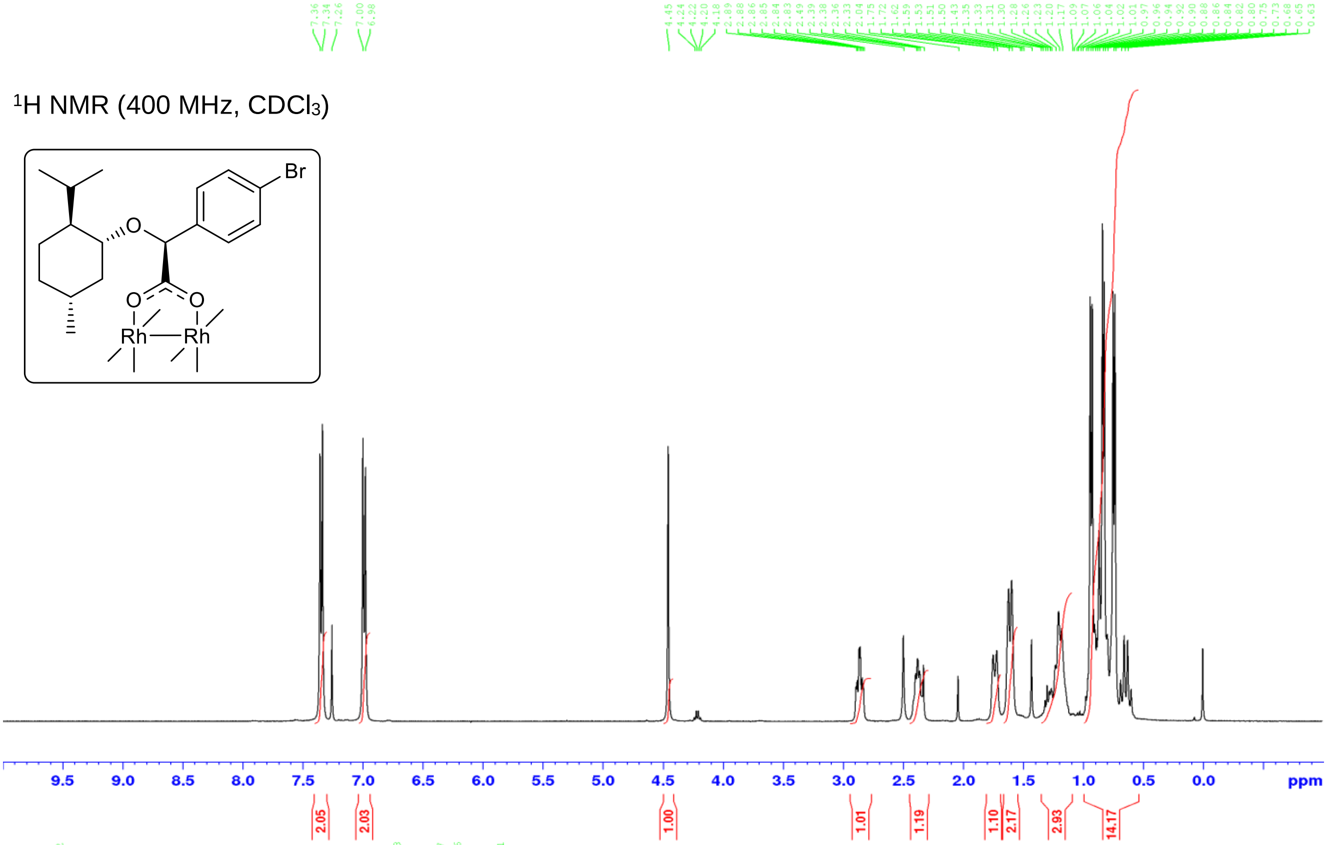

^{13}C NMR (100.6 MHz, $CDCl_3$)

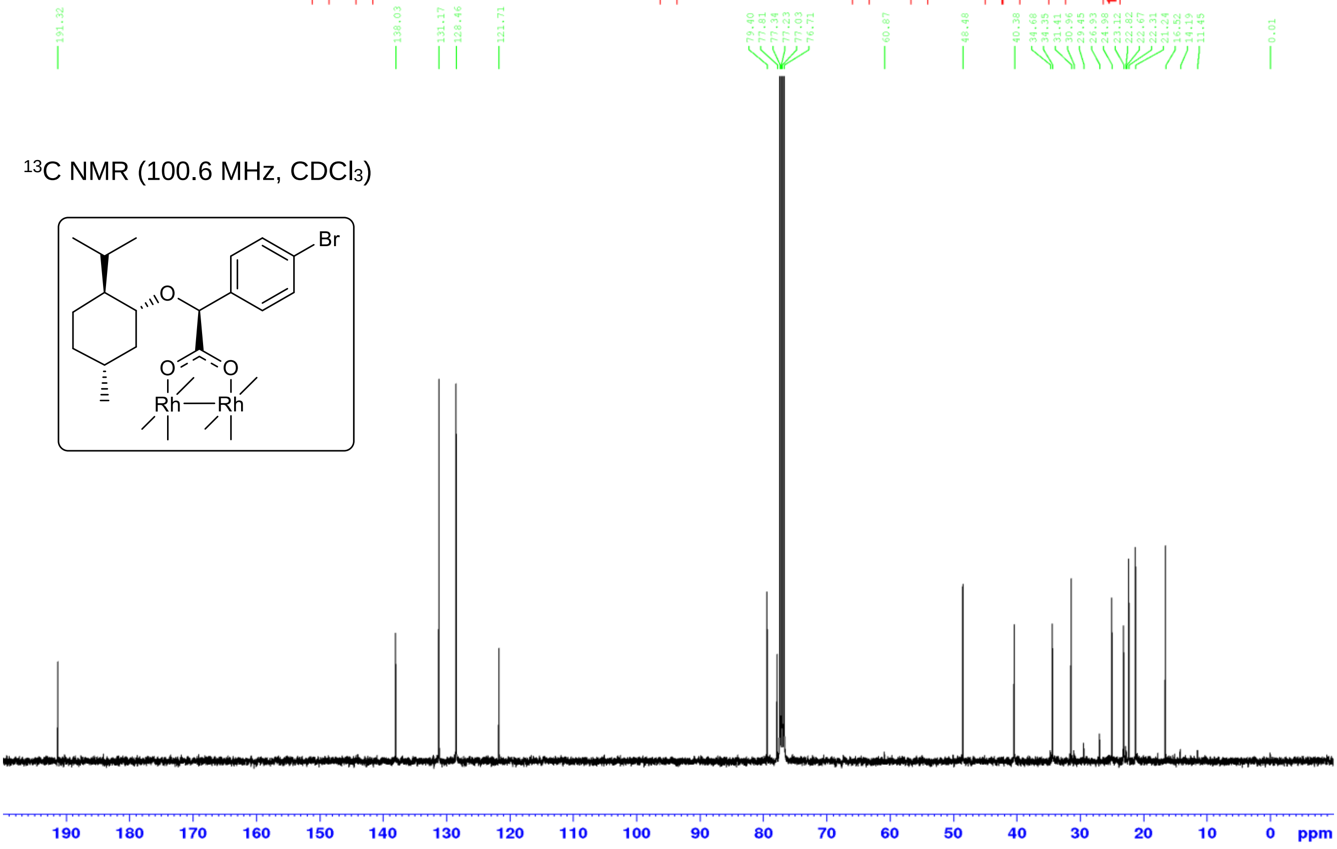

Methyl 2-(2-(benzyloxy)phenyl)acetate (S17)

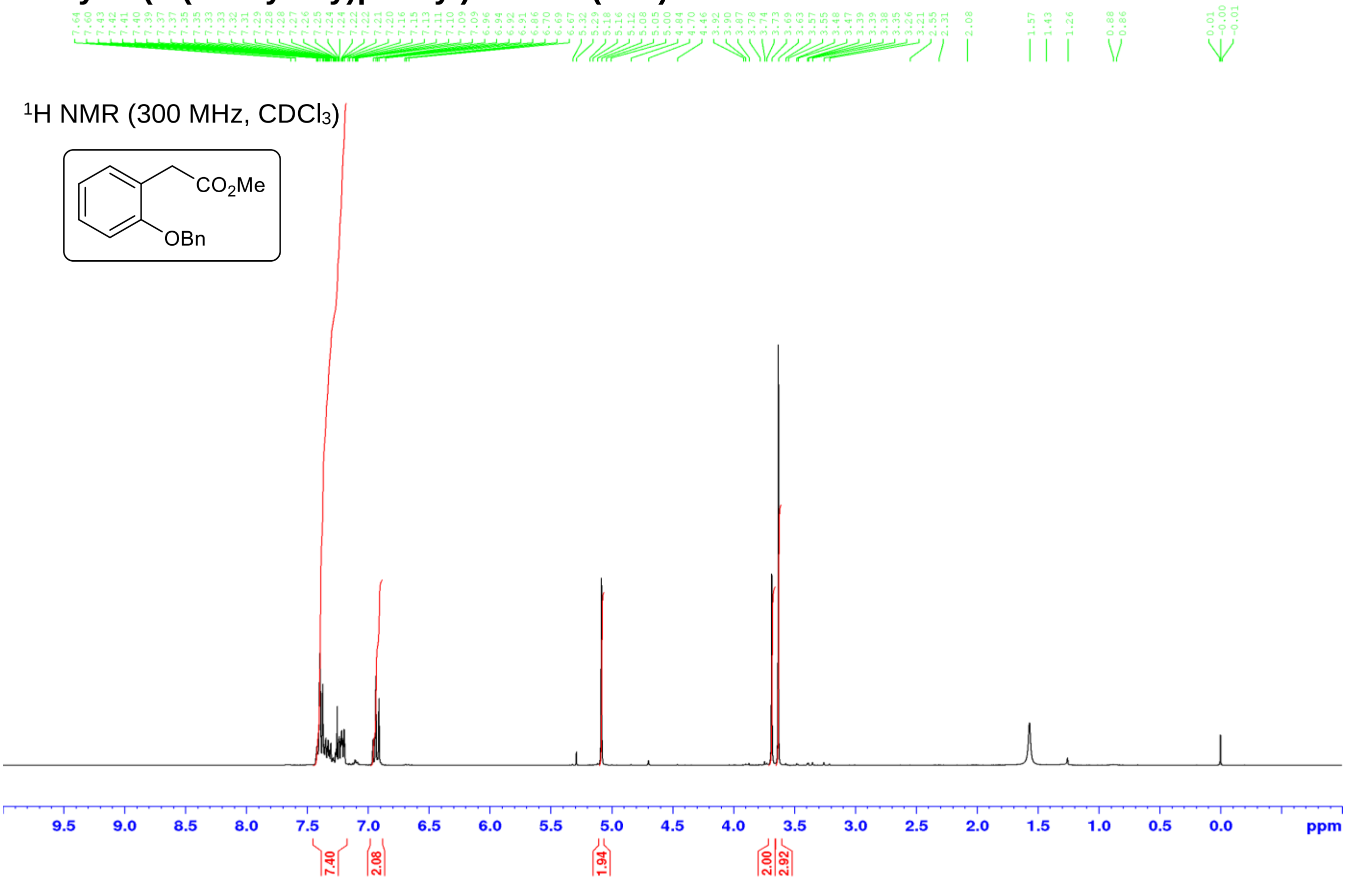

Benzyl 2-(2-(benzyloxy)phenyl)acetate (S18)

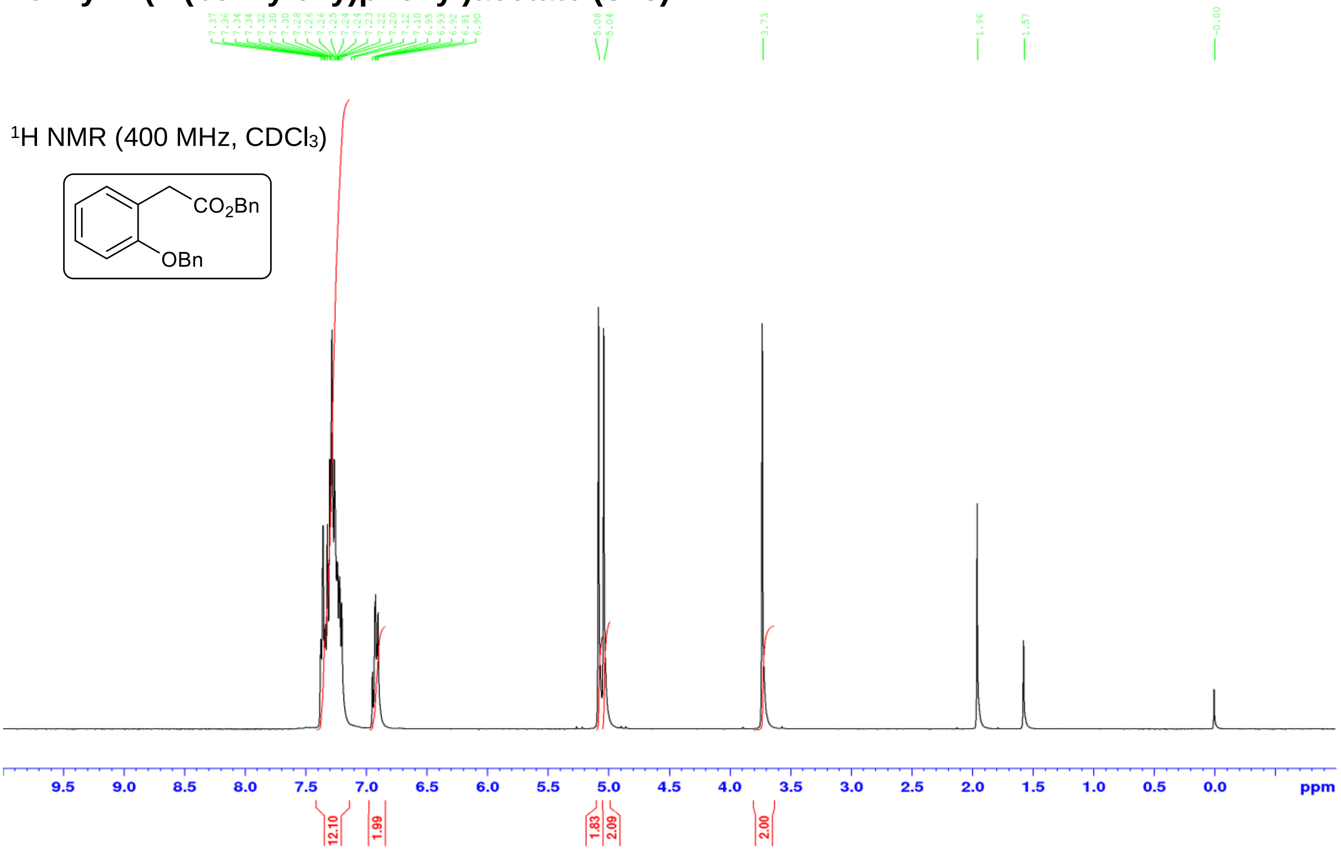

Methyl 2-(2-(benzyloxy)phenyl)-2-diazoacetate (10)

^{1}H NMR (400 MHz, $CDCl_3$)

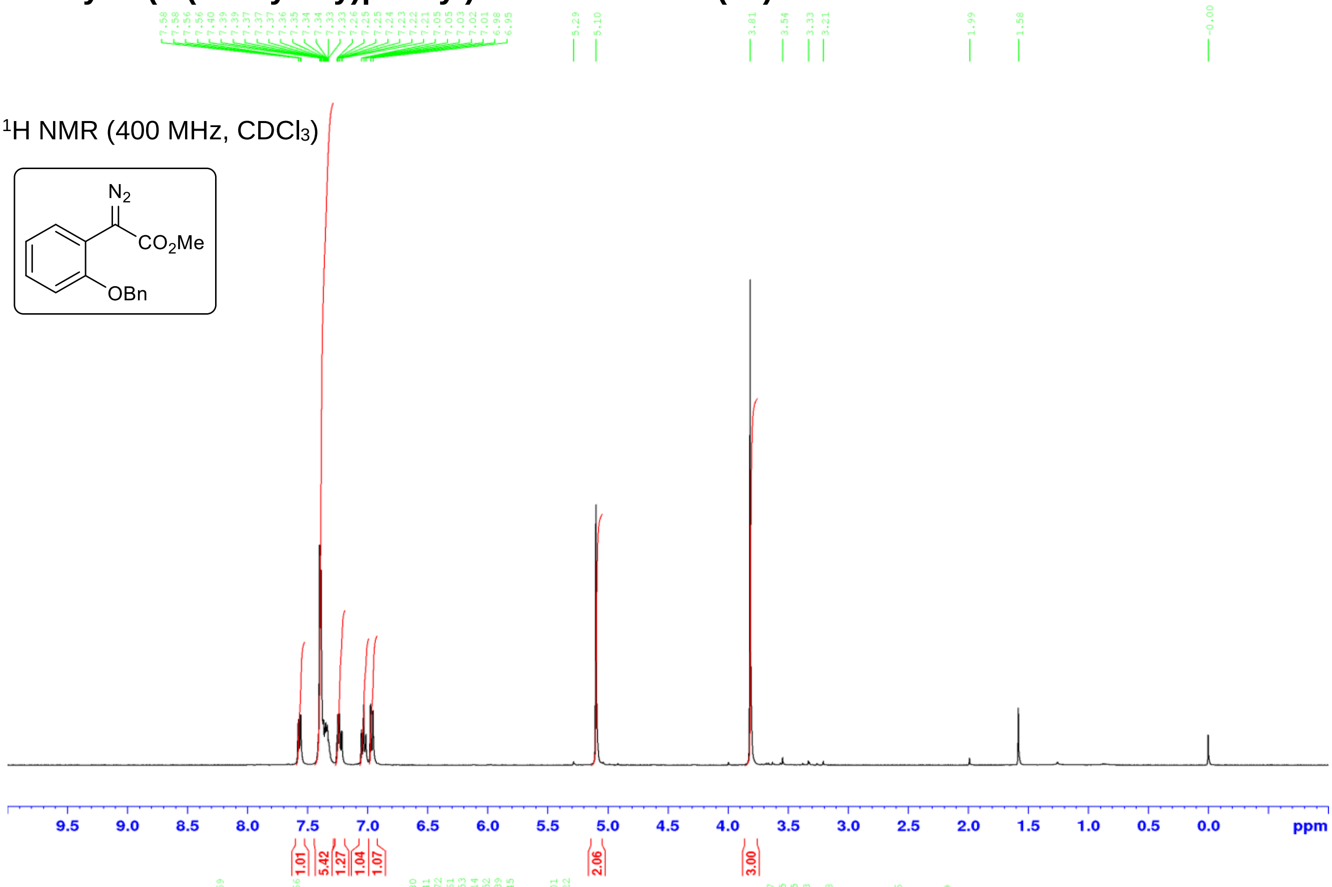

^{13}C NMR (100.6 MHz, $CDCl_3$)

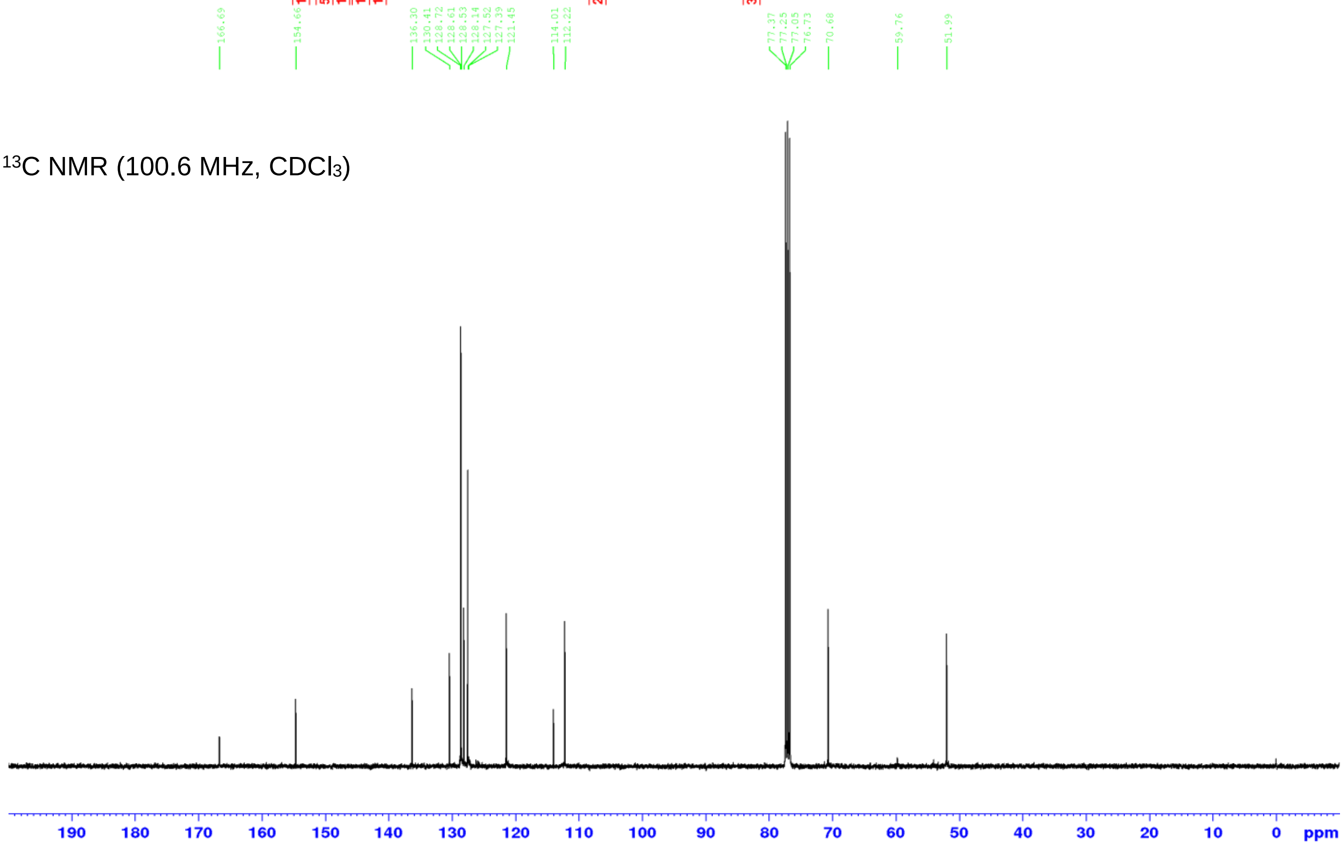

Benzyl 2-(2-(benzyloxy)phenyl)-2-diazoacetate (12)

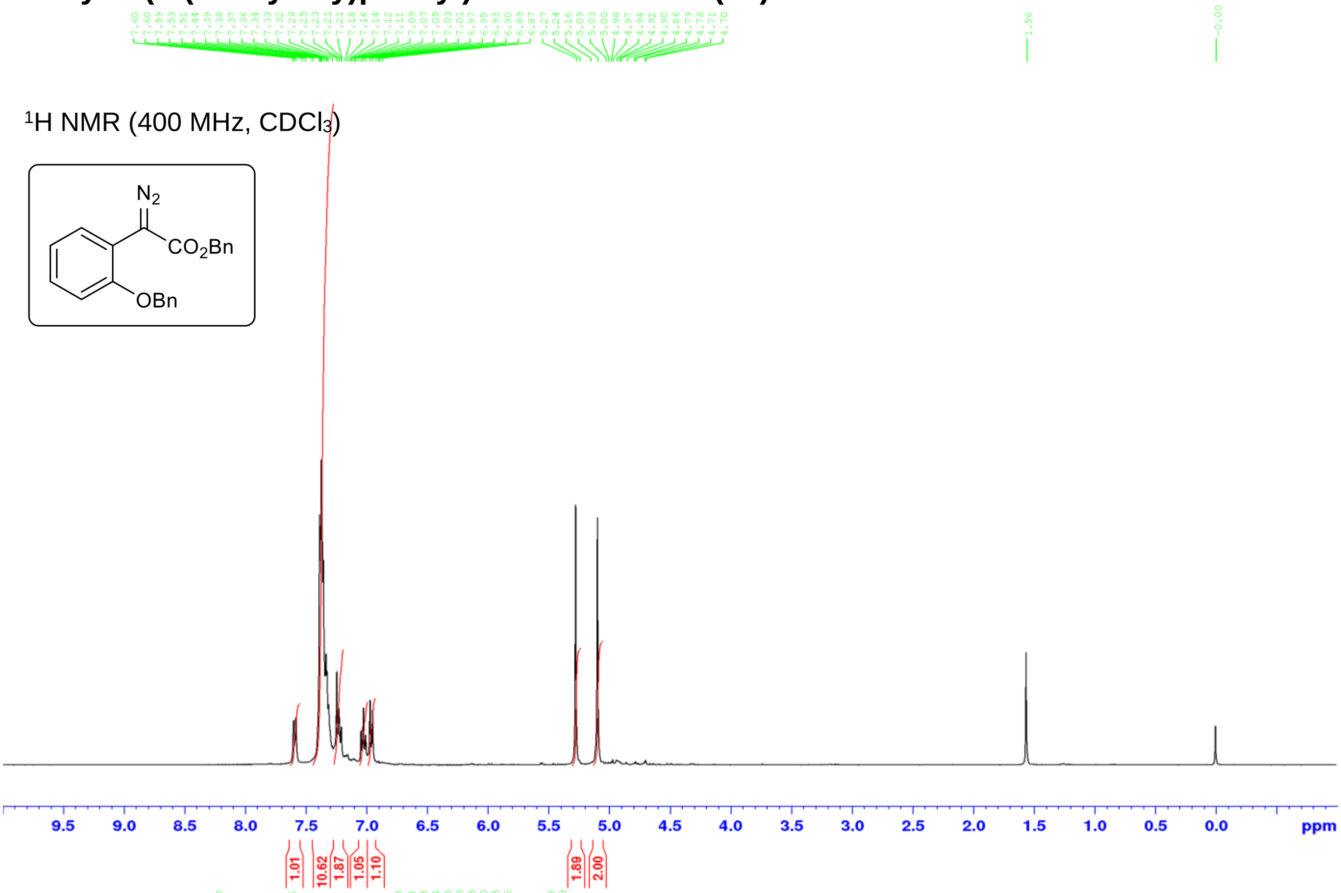

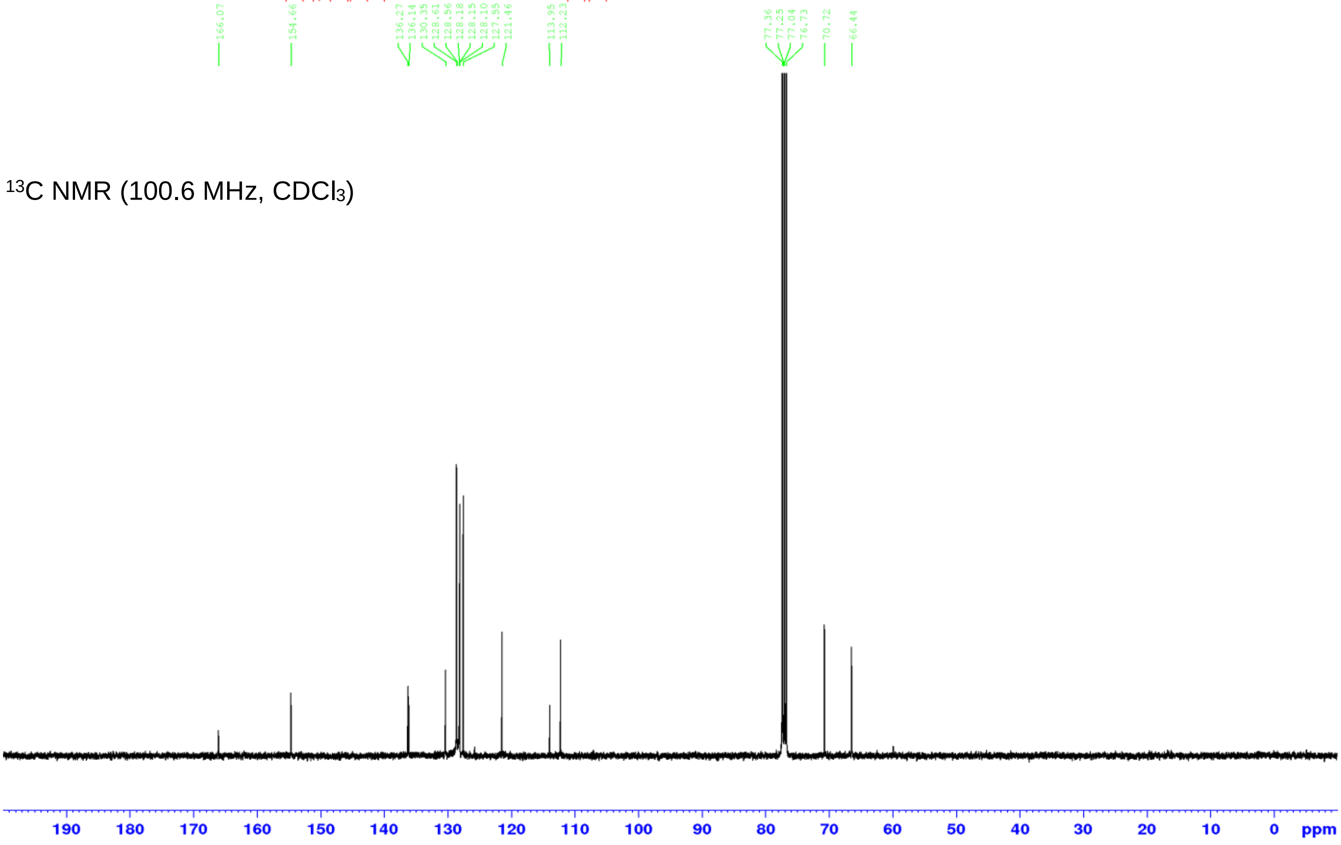

Isopropyl 2-(2-(benzyloxy)phenyl)-2-diazoacetate (13)

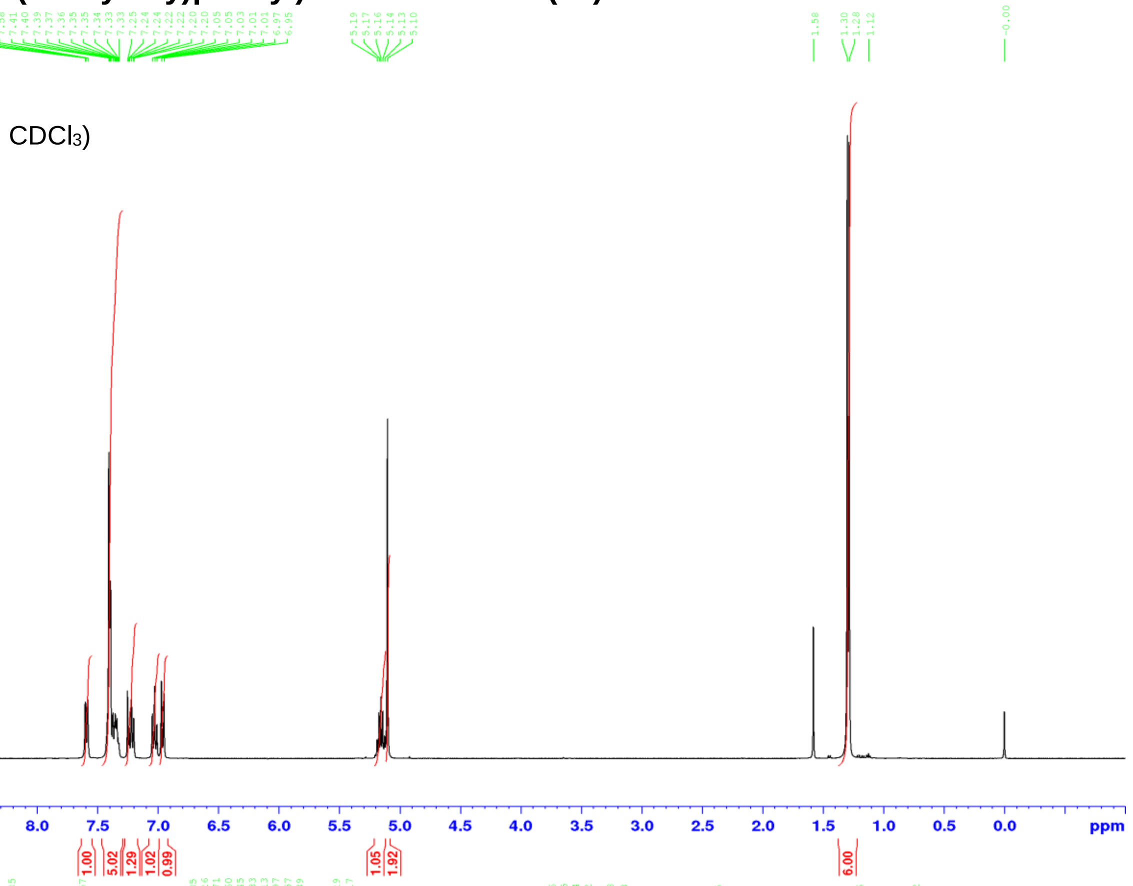

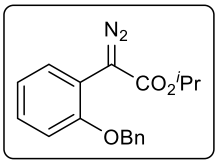

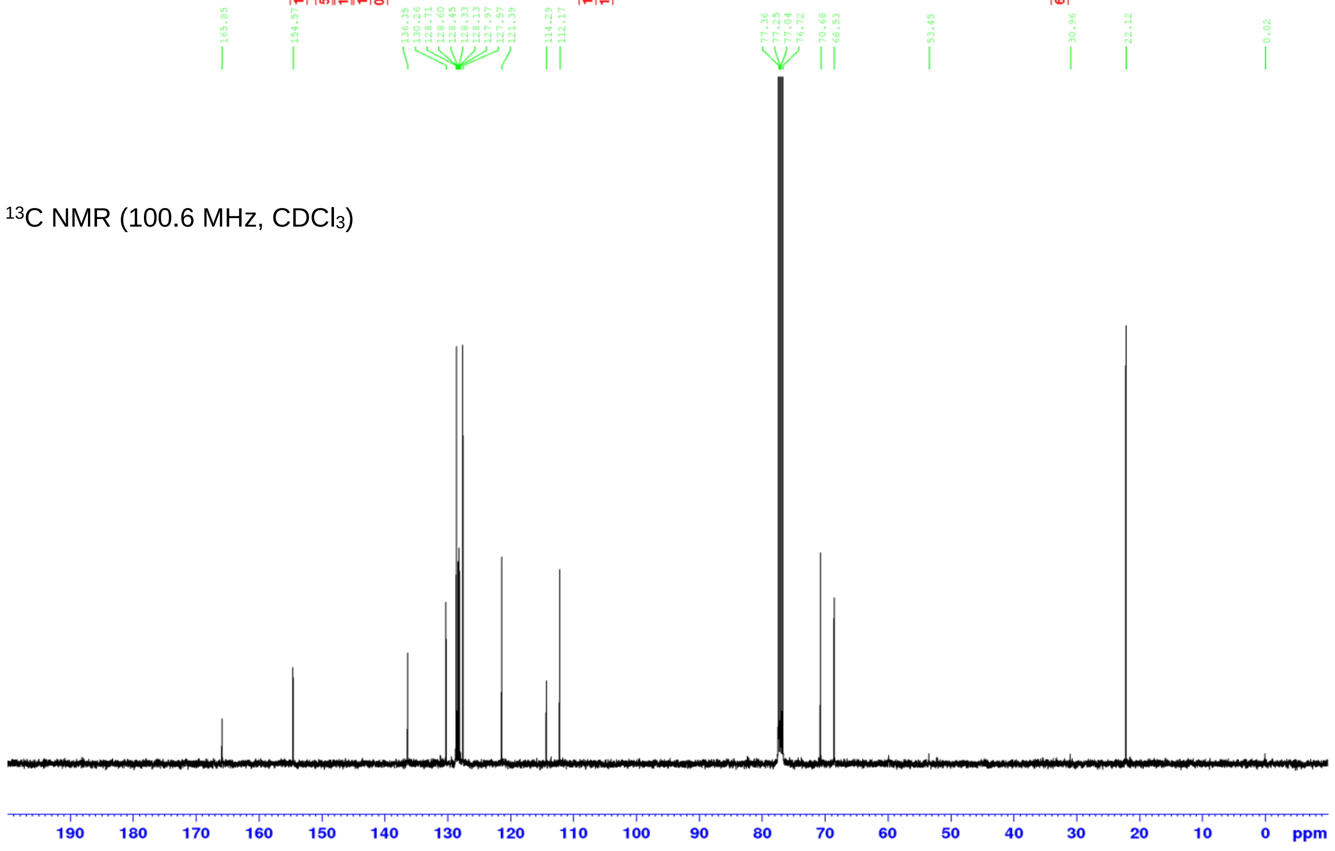

(2*R*,3*R*)-trans-3-Methoxycarbonyl-2-phenyl-2,3-dihydrobenzofuran[17] (11a)

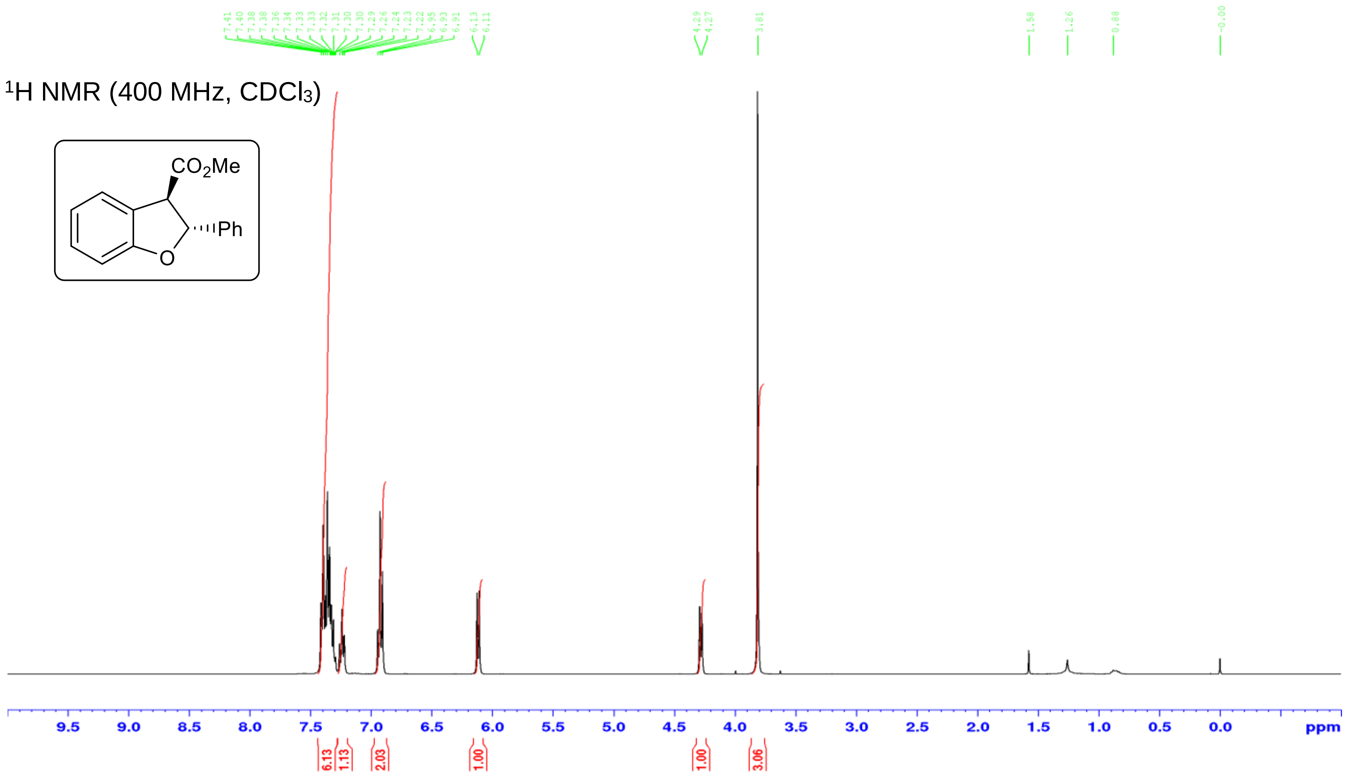

(2*S*,3*R*)-cis-3-Methoxycarbonyl-2-phenyl-2,3-dihydrobenzofuran[17] (11b)

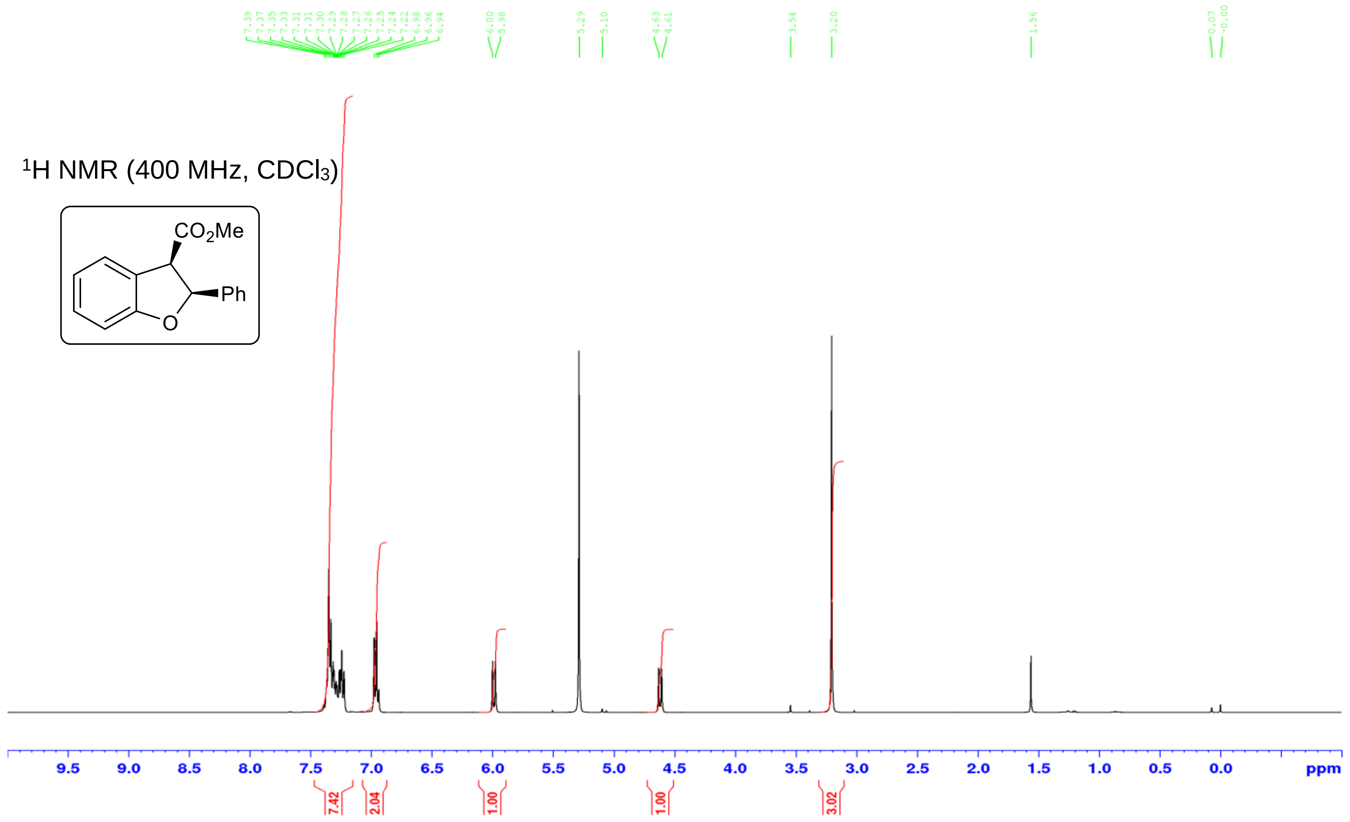

(2*R*,3*R*)-trans-3-Benzyloxycarbonyl-2-phenyl-2,3-dihydrobenzofuran (14a)

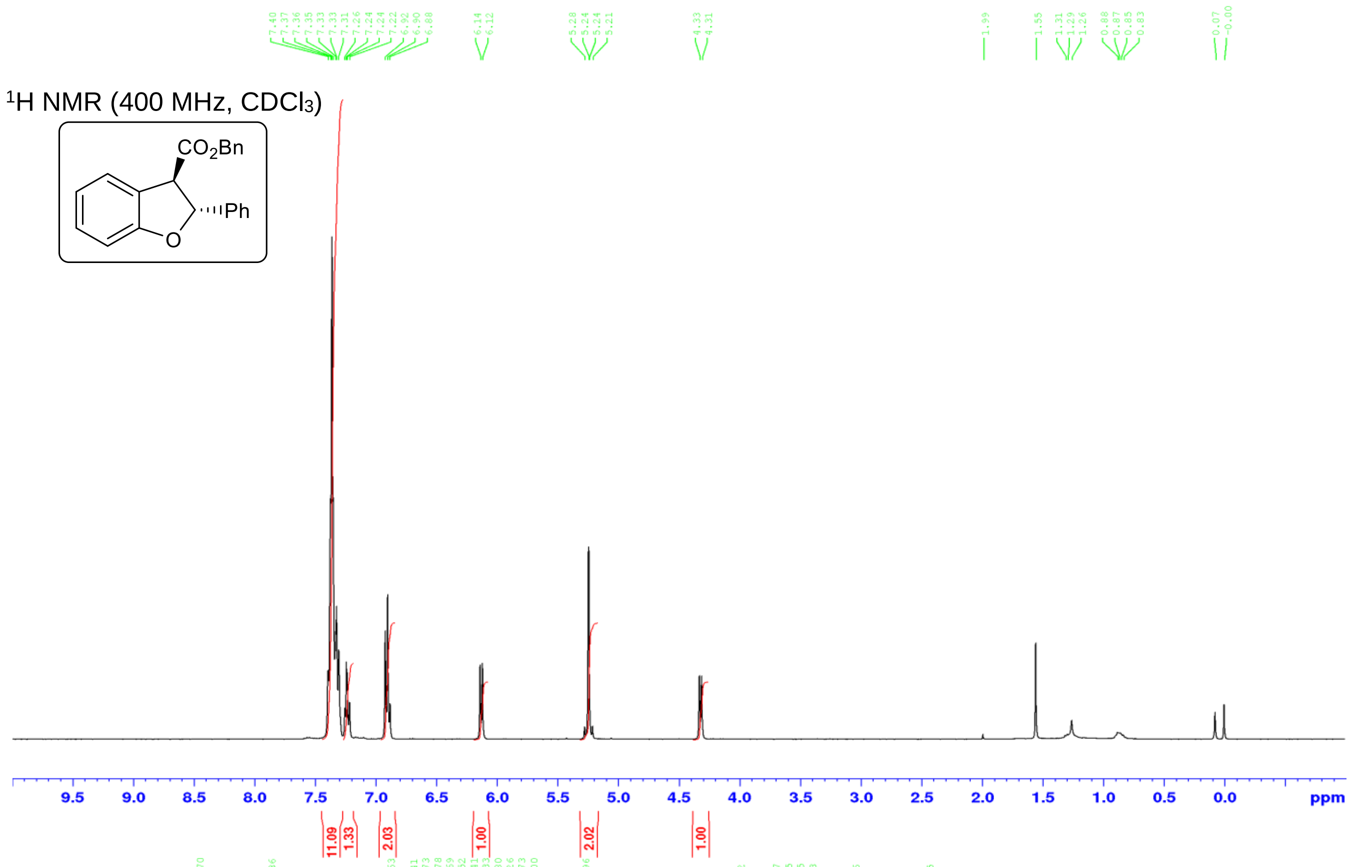

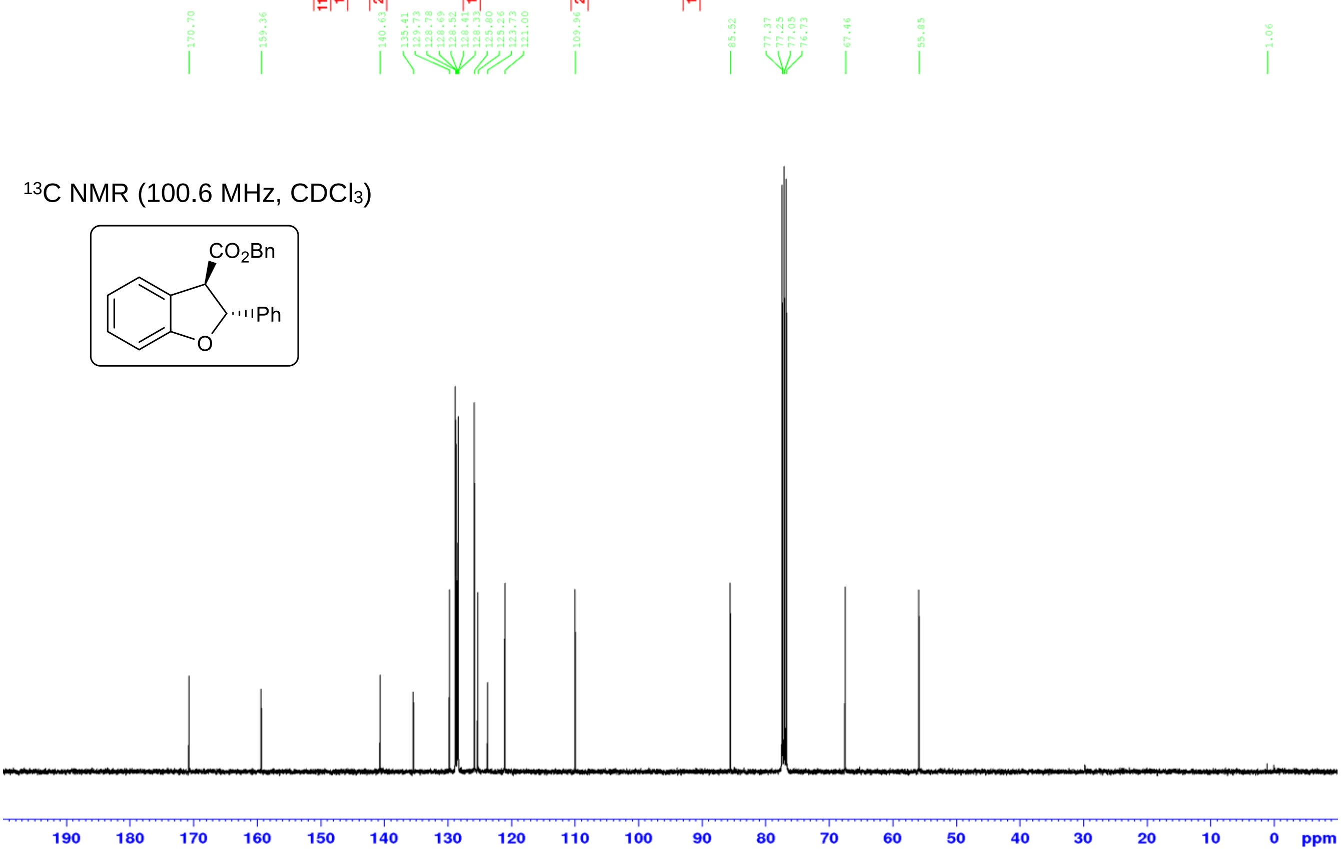

(2*S*,3*R*)-cis-3-Benzyloxycarbonyl-2-phenyl-2,3-dihydrobenzofuran (14b)

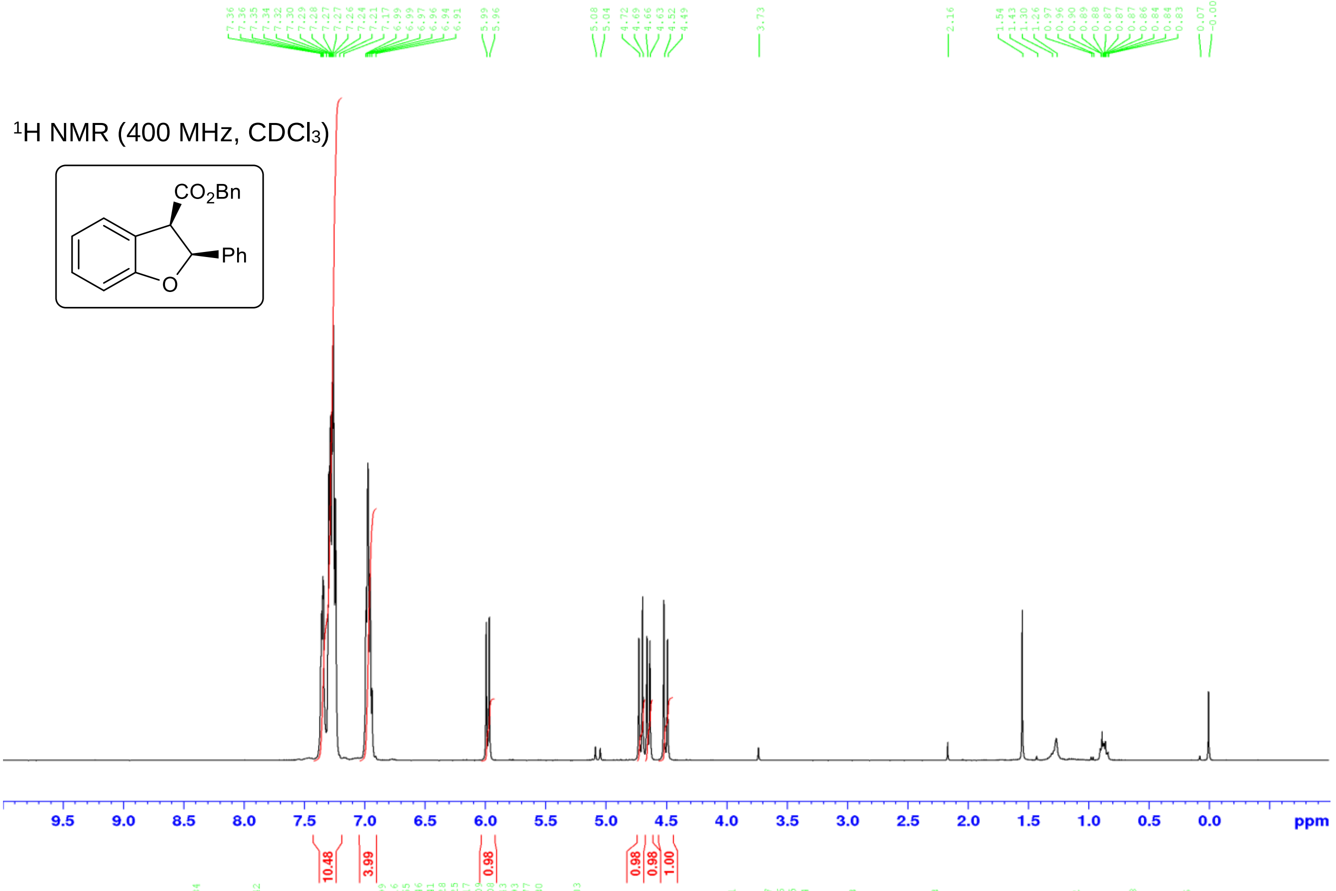

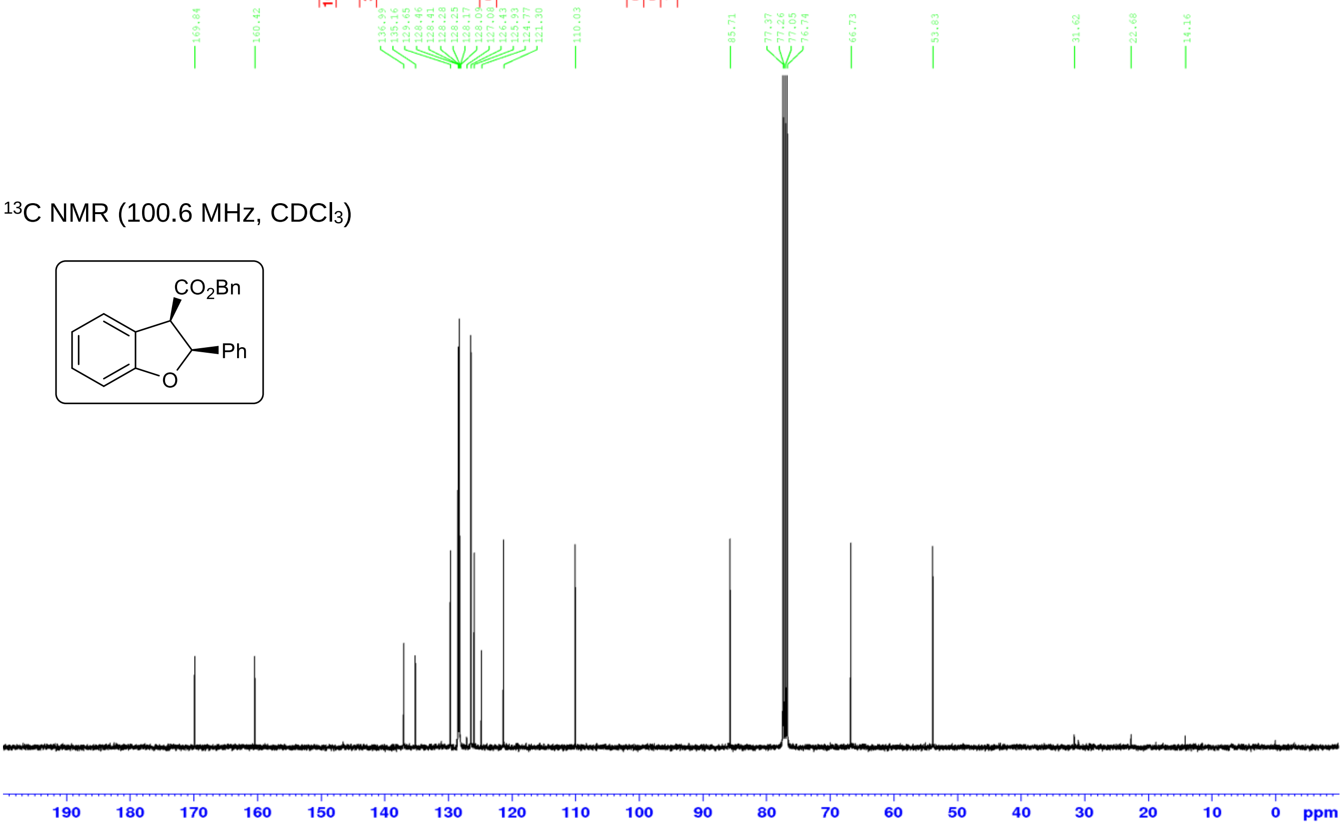

(2*R*,3*R*)-trans-3-Isopropyloxycarbonyl-2-phenyl-2,3-dihydrobenzofuran (15a)

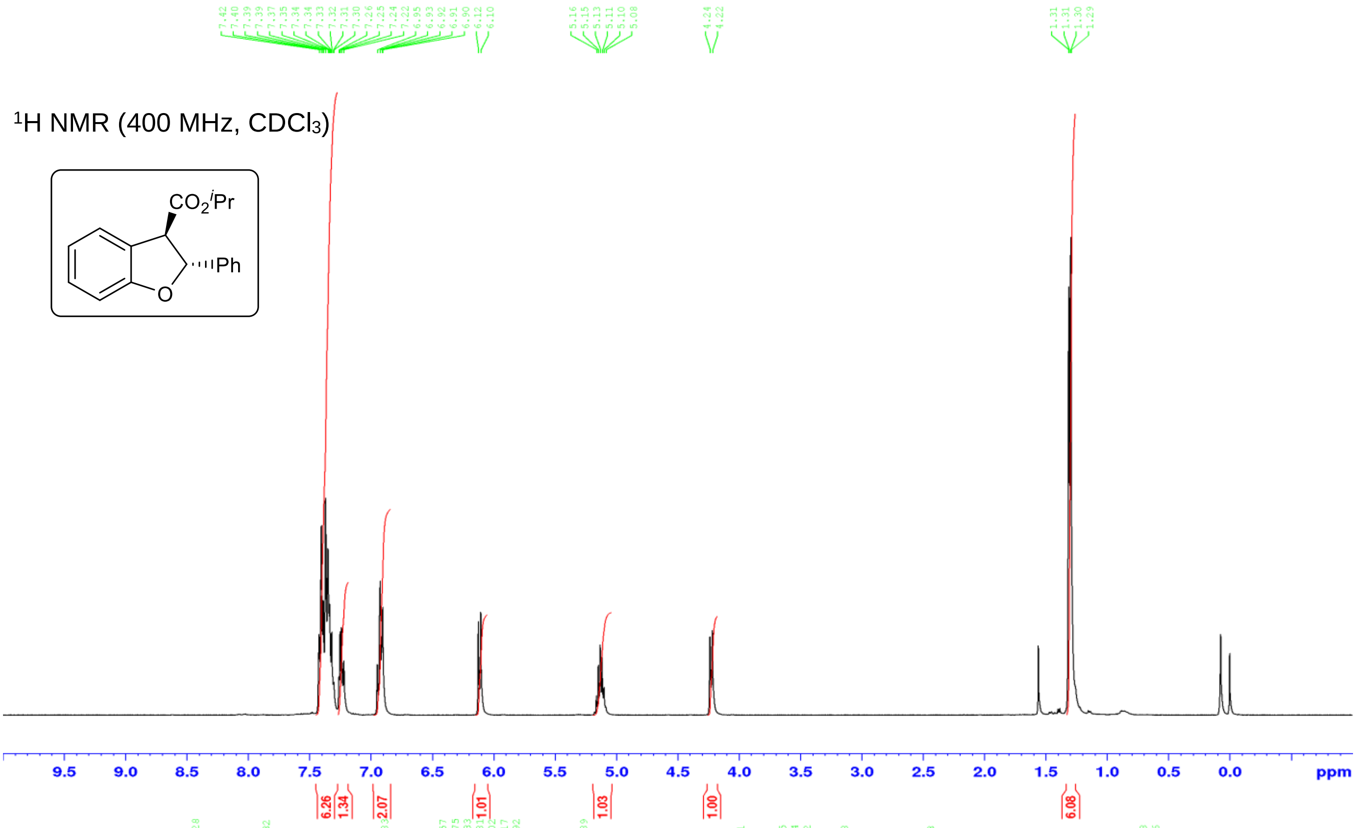

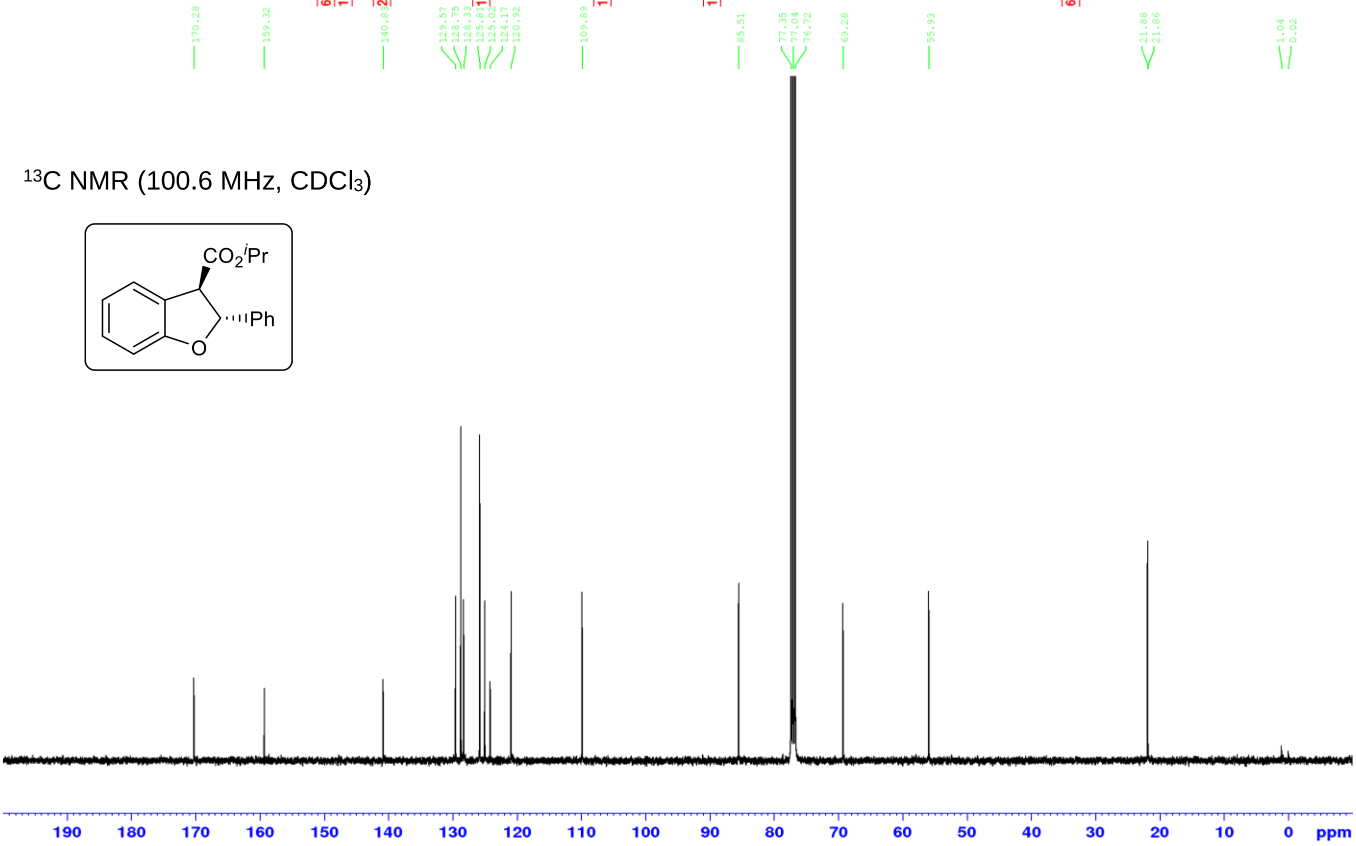

(2*S*,3*R*)-cis-3-Isopropyloxycarbonyl-2-phenyl-2,3-dihydrobenzofuran (15b)

^{1}H NMR (400 MHz, $CDCl_3$)

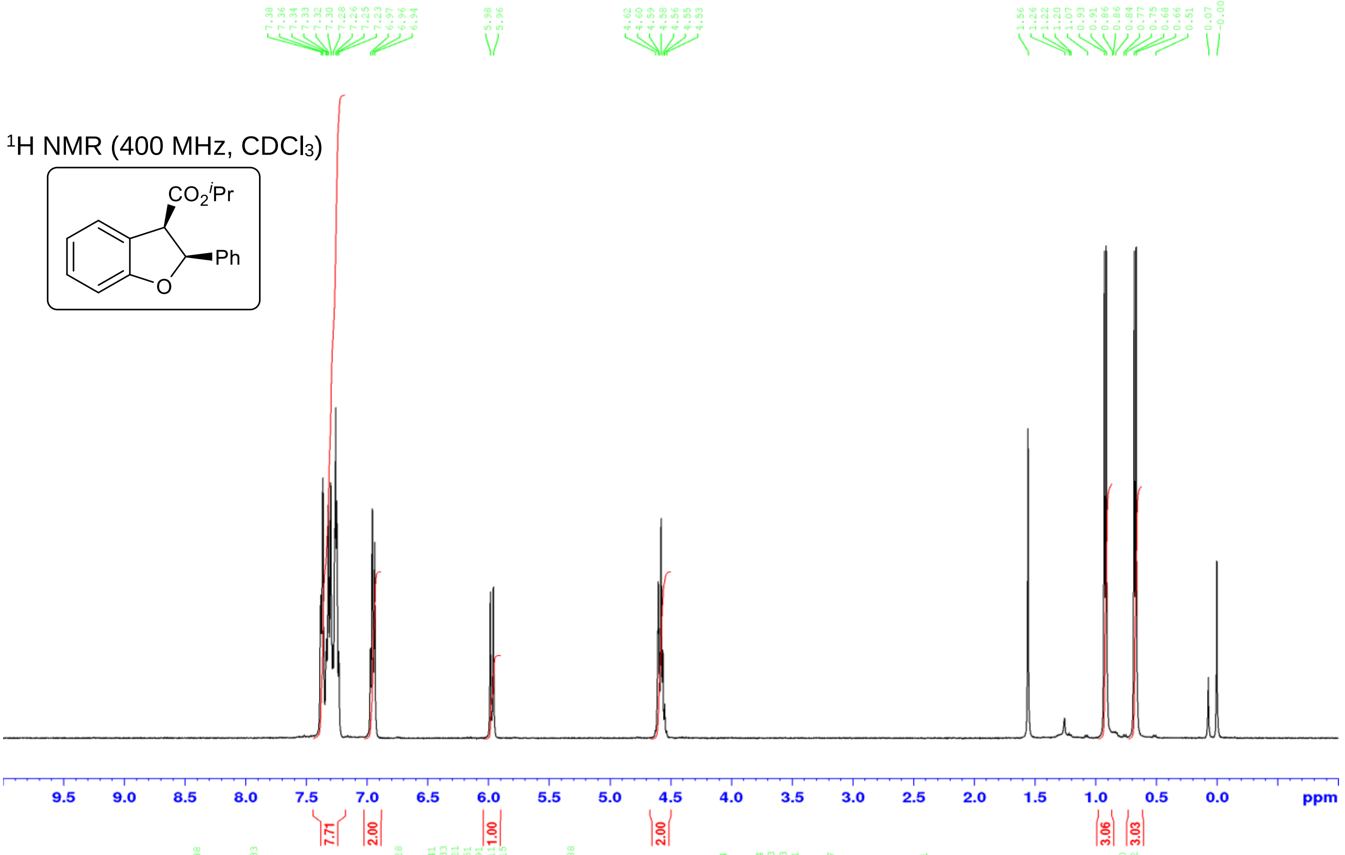

^{13}C NMR (100.6 MHz, $CDCl_3$)

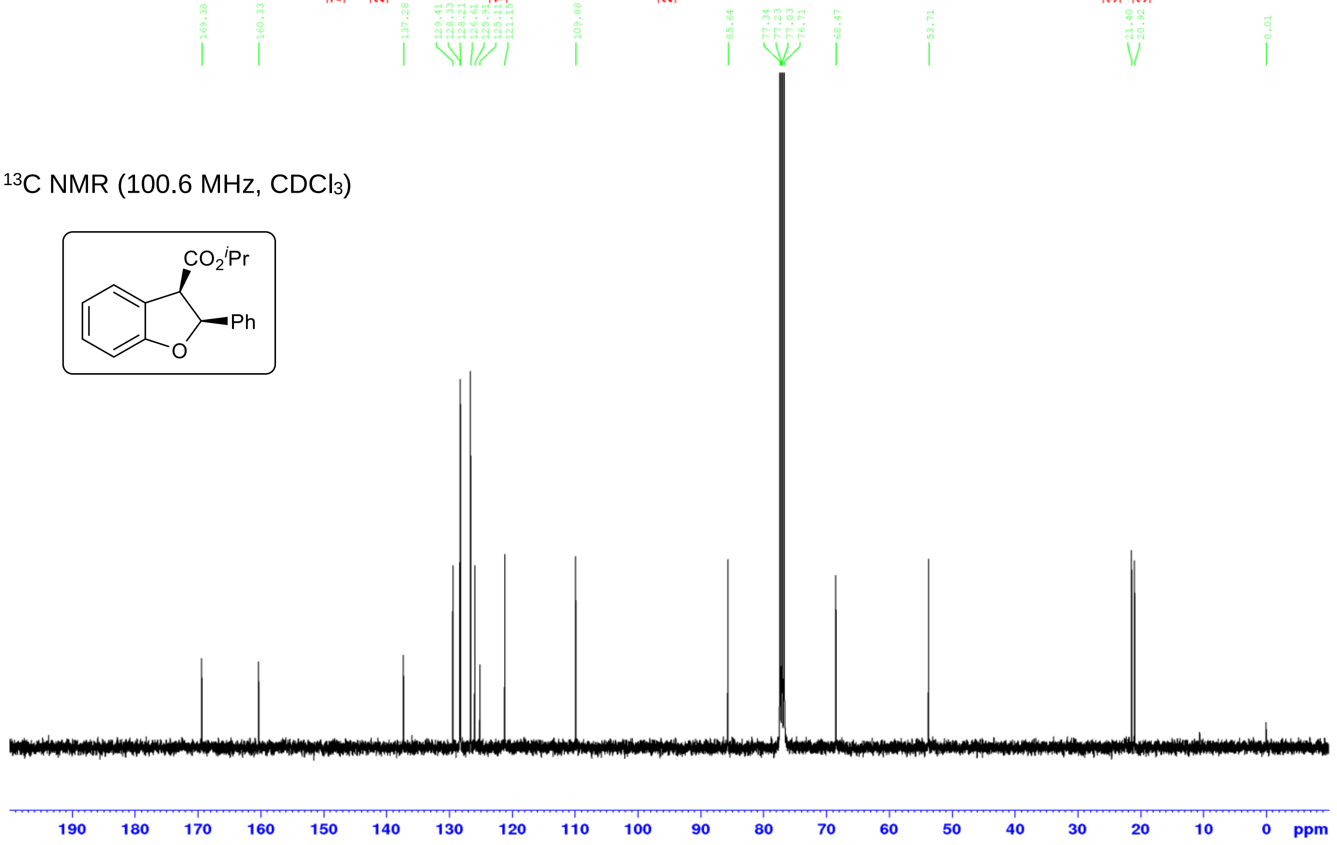

Methyl 2-(2-(benzyloxy)phenyl)-2-oxoacetate (S19)

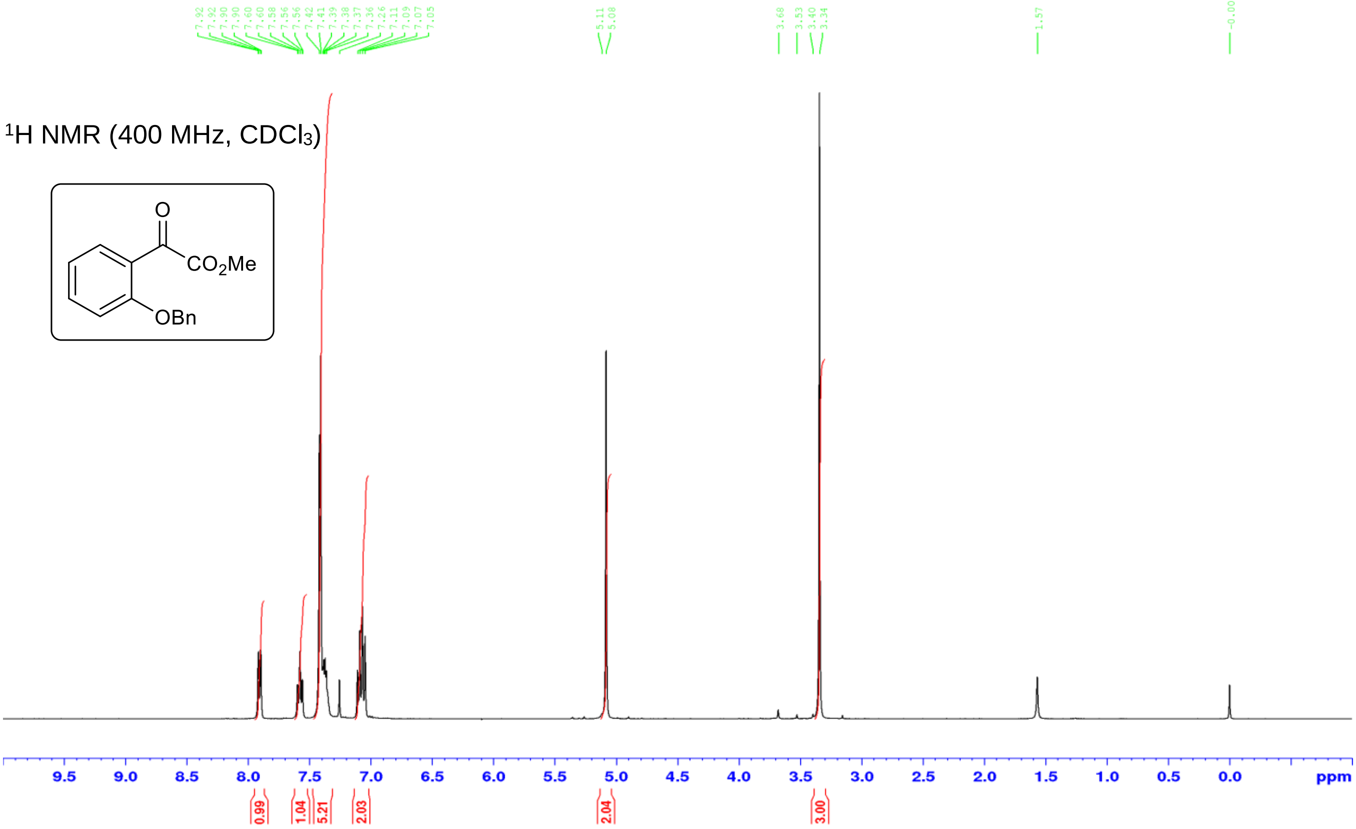

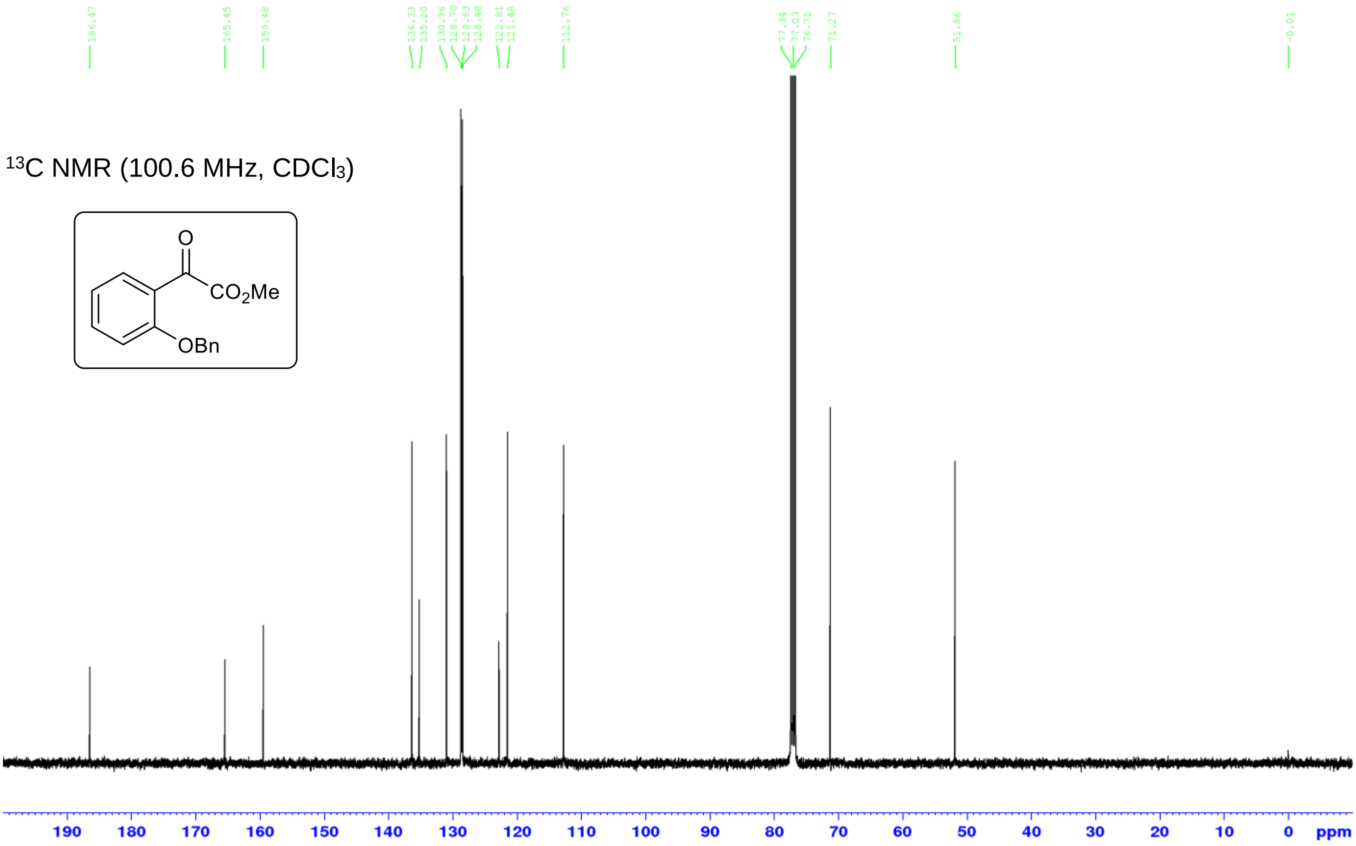

Methyl 2-(2-(benzyloxy)phenyl)-2-oxoacetate (S20)

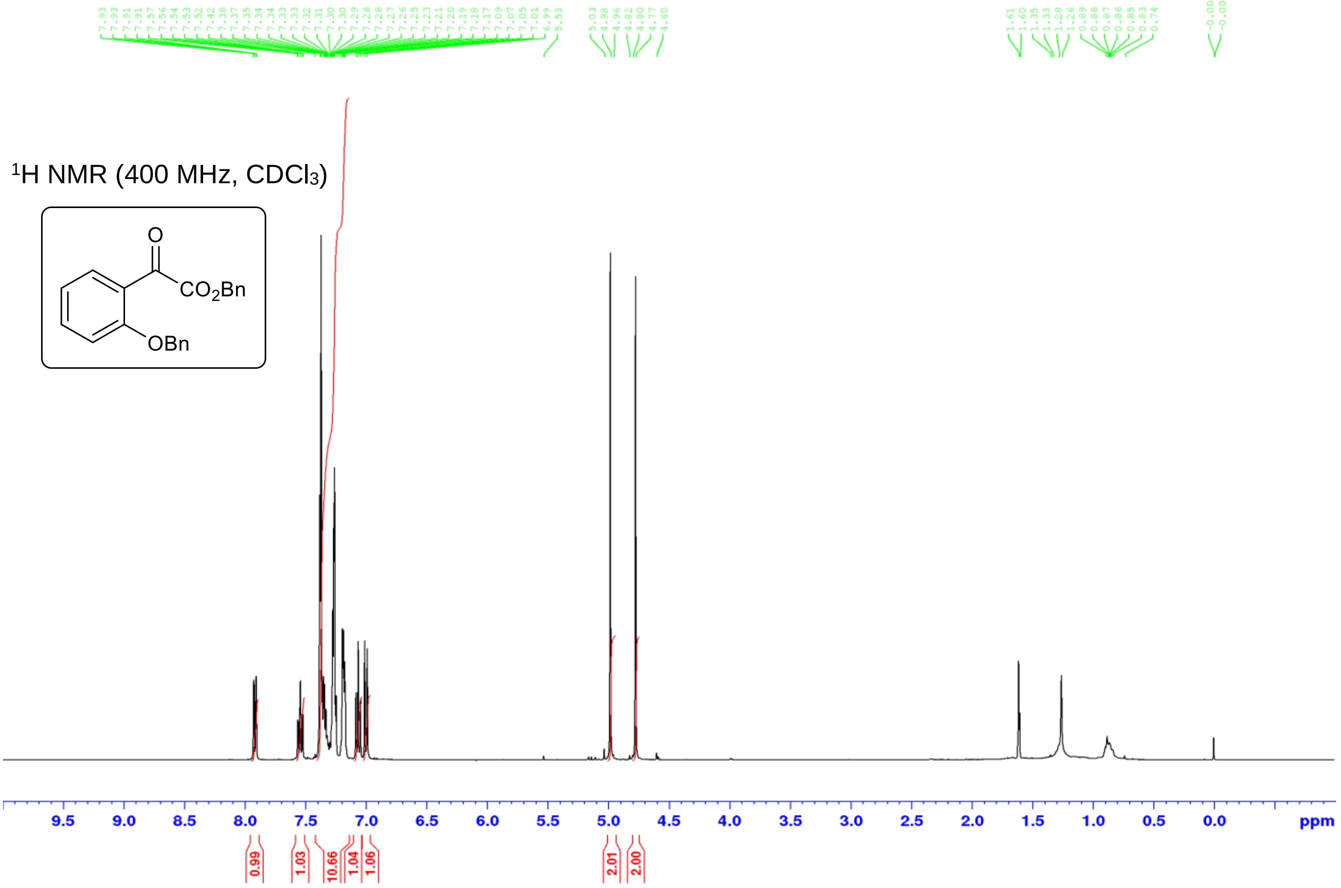

Benzyl 2-(2-(benzyloxy)phenyl)-2-hydroxyacetate (S21)

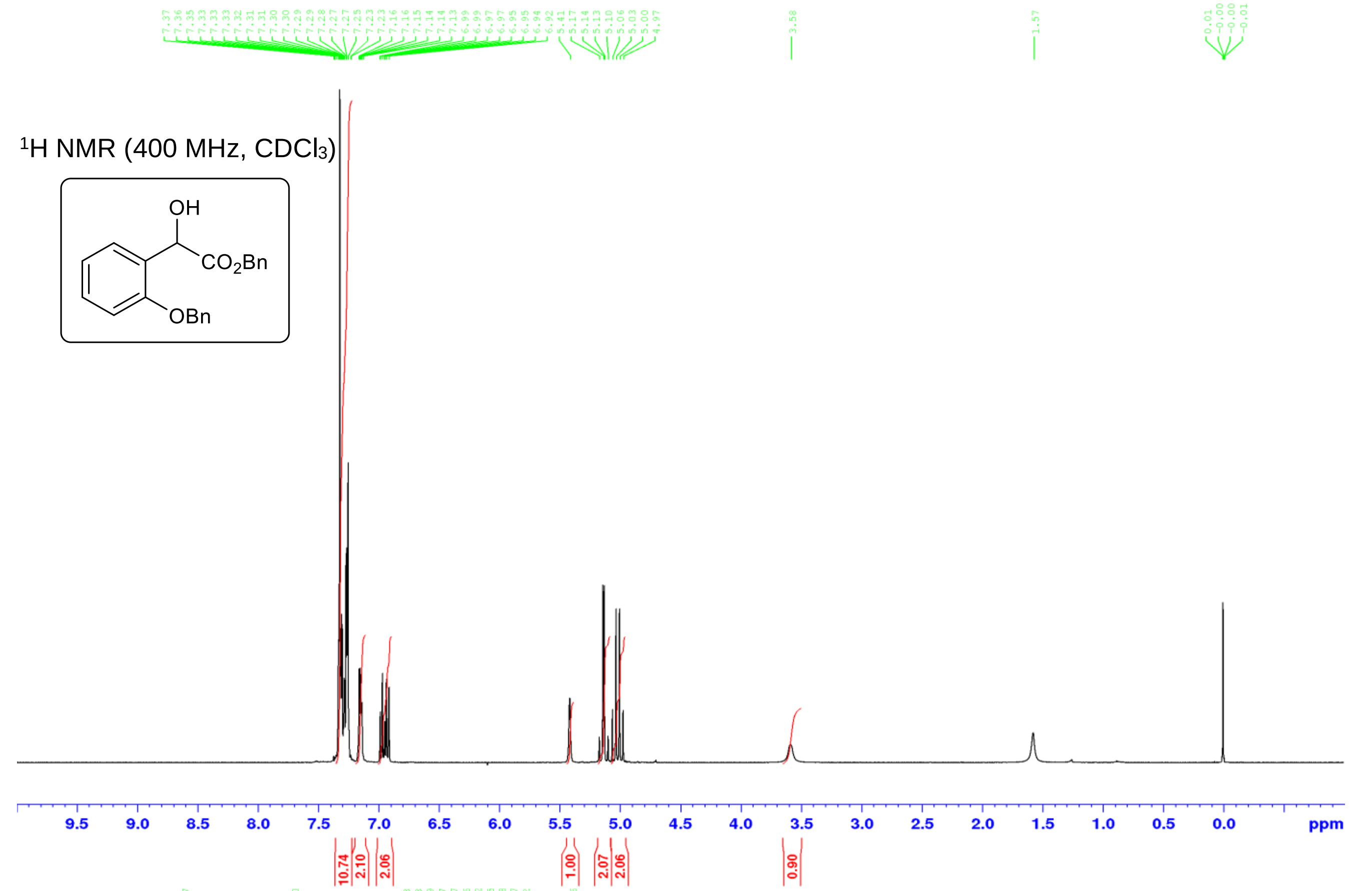

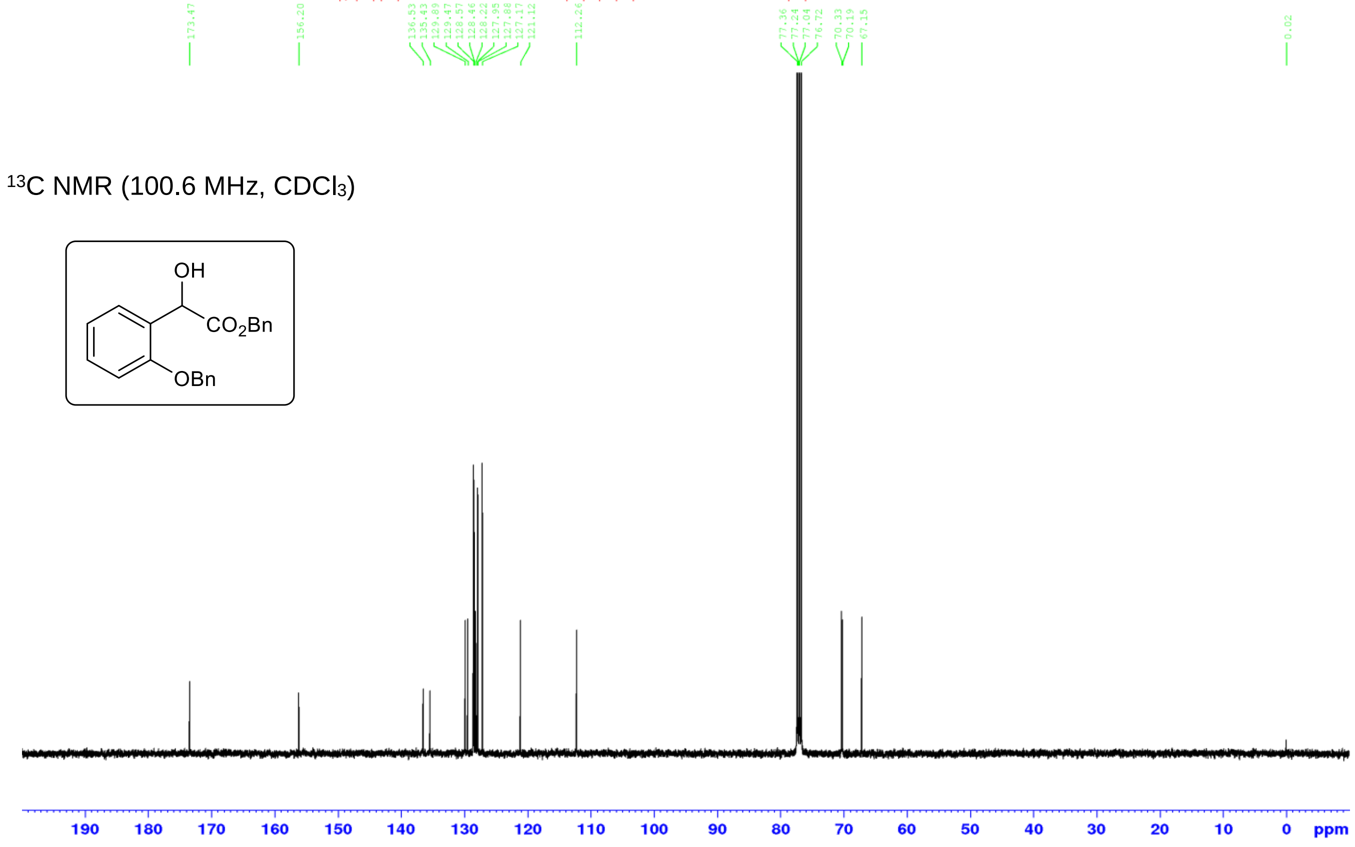

*The ^{1}H NMR and ^{13}C NMR spectra of the C–H insertion products isolated in this work are reported below. For the compounds we have previously reported, namely the cis thiopyran dioxides and the α-diazocarbonyl compounds (**16**, **19**–**24**) the spectroscopic details are in agreement with previously reported data.*[19-20]

Methyl 2-diazo-2-((4′-phenylbutyl)sulfonyl)acetate (16)

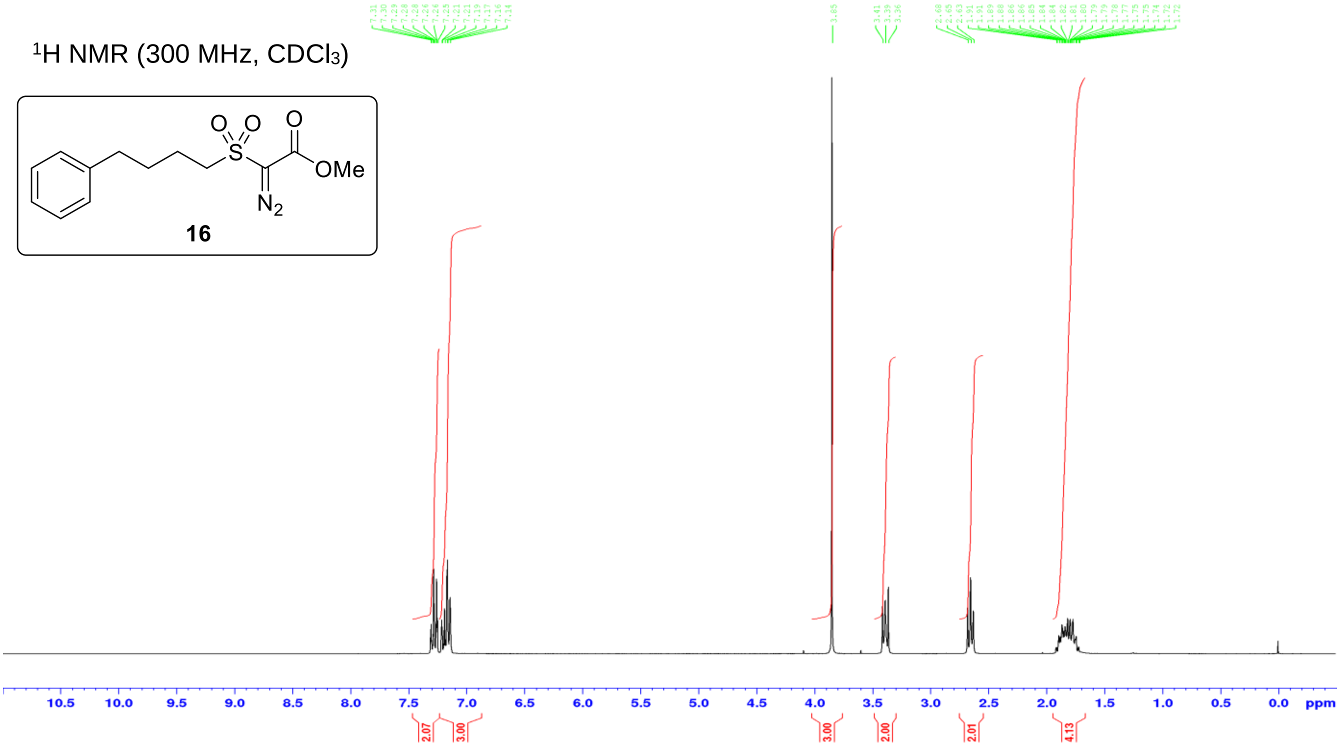

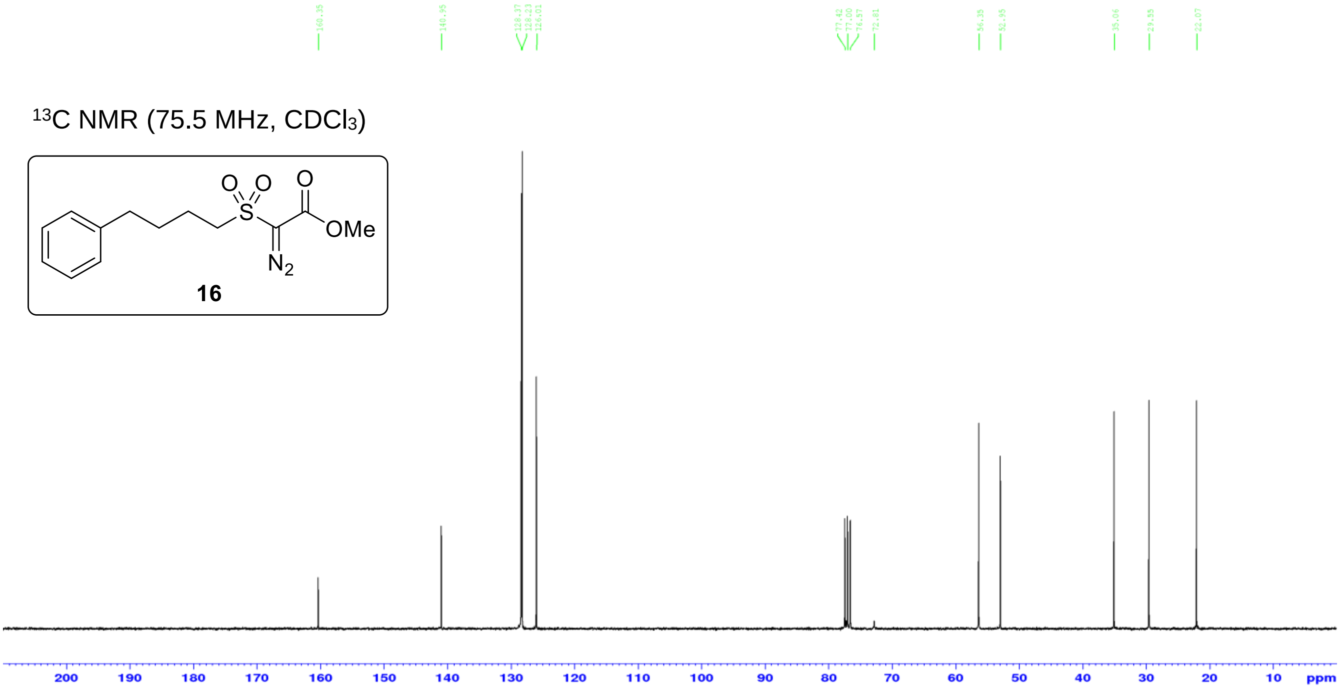

Methyl 2-diazo-2-((4'-(*p*-tolyl)butyl)sulfonyl)acetate (19)

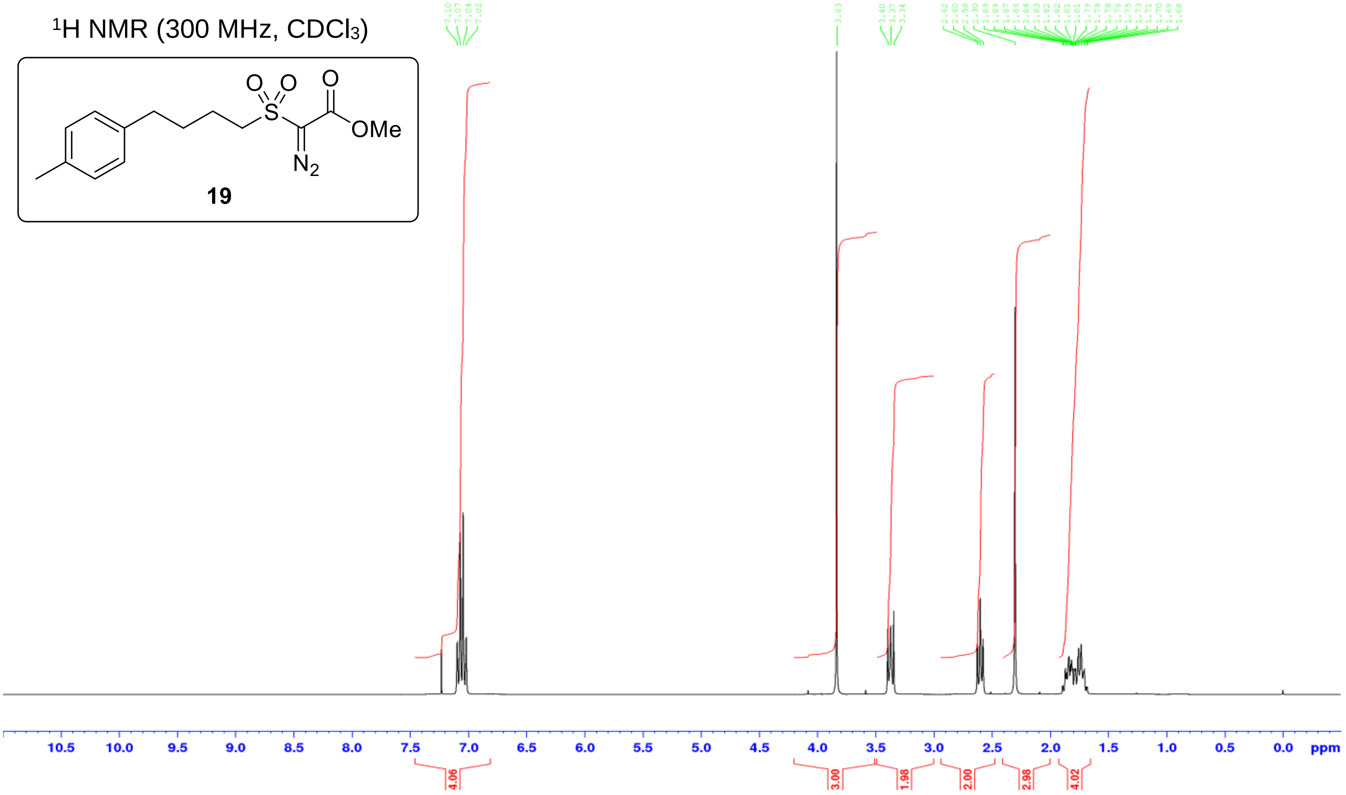

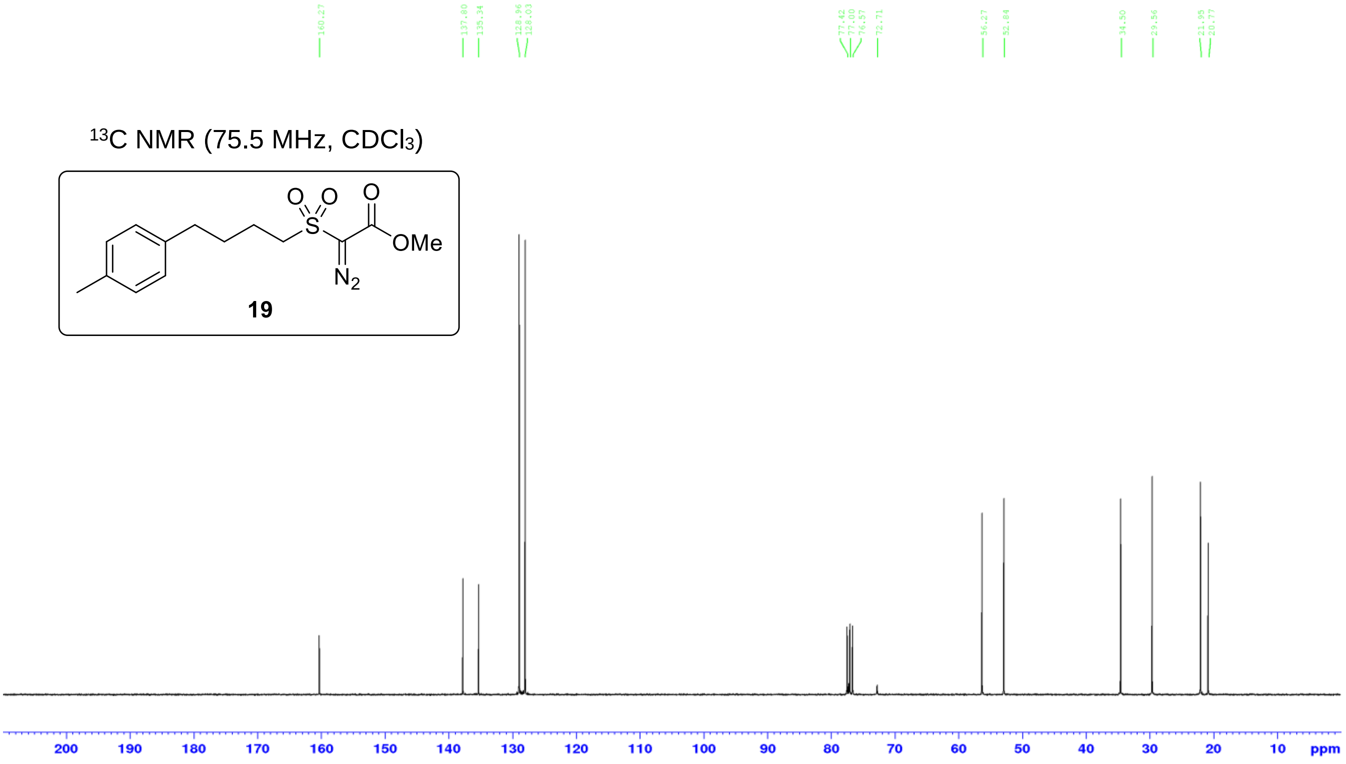

Methyl 2-diazo-2-((4'-(4″-methoxyphenyl)butyl)sulfonyl)acetate (20)

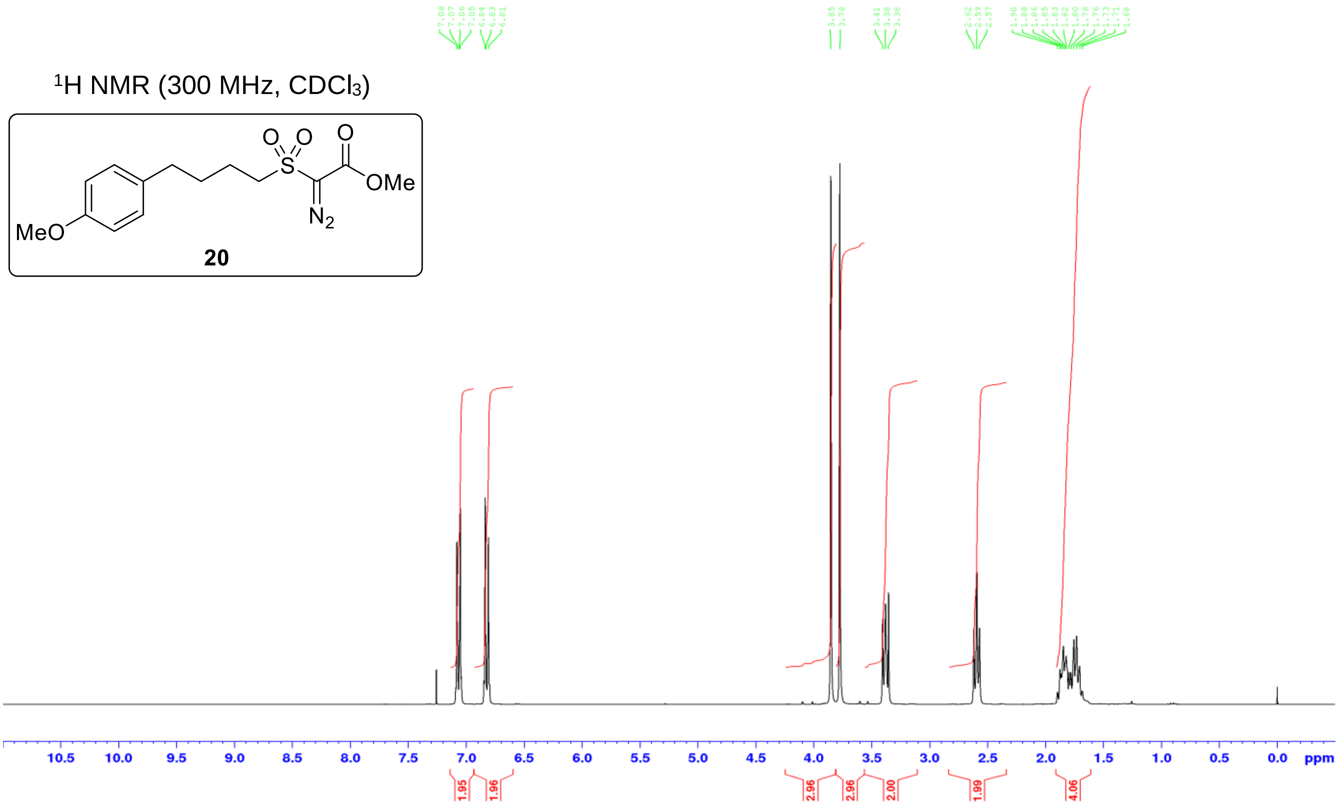

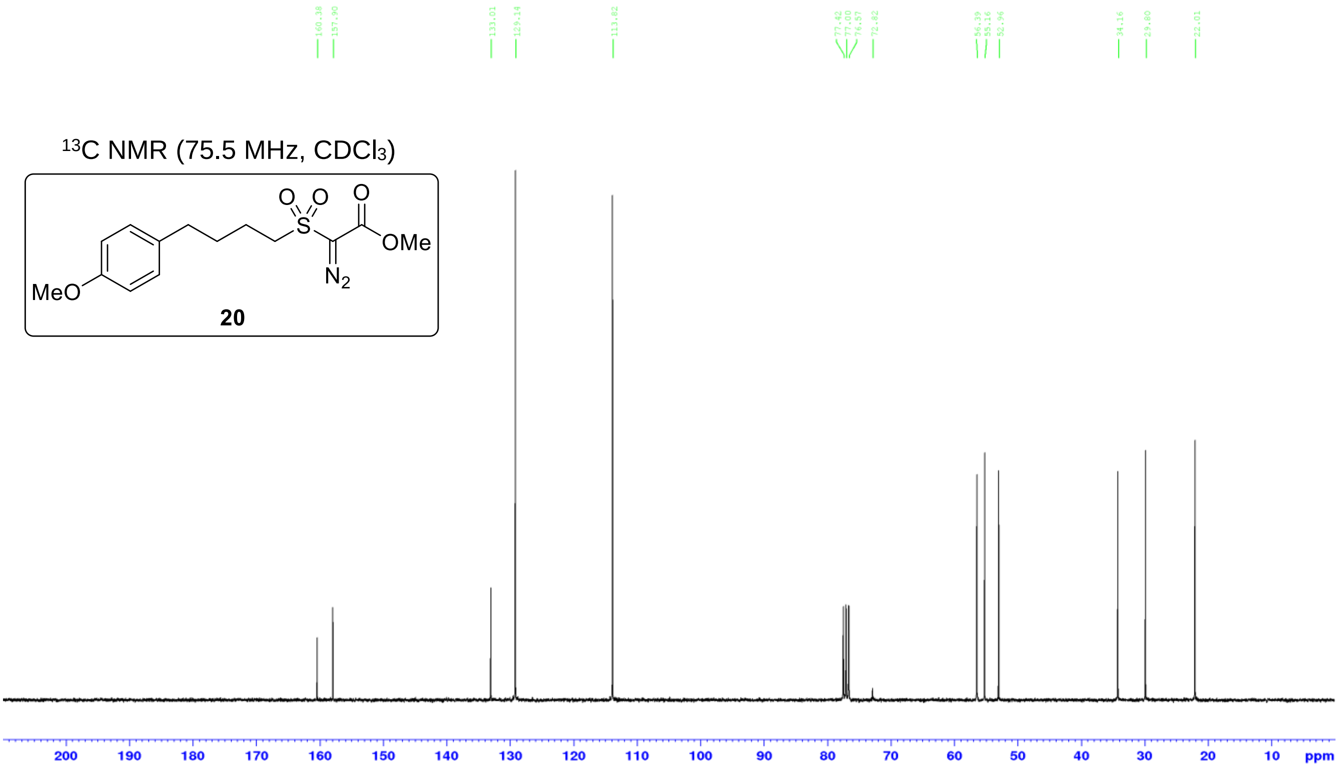

Methyl 2-diazo-2-((4'-(4″-fluorophenyl)butyl)sulfonyl)acetate (21)

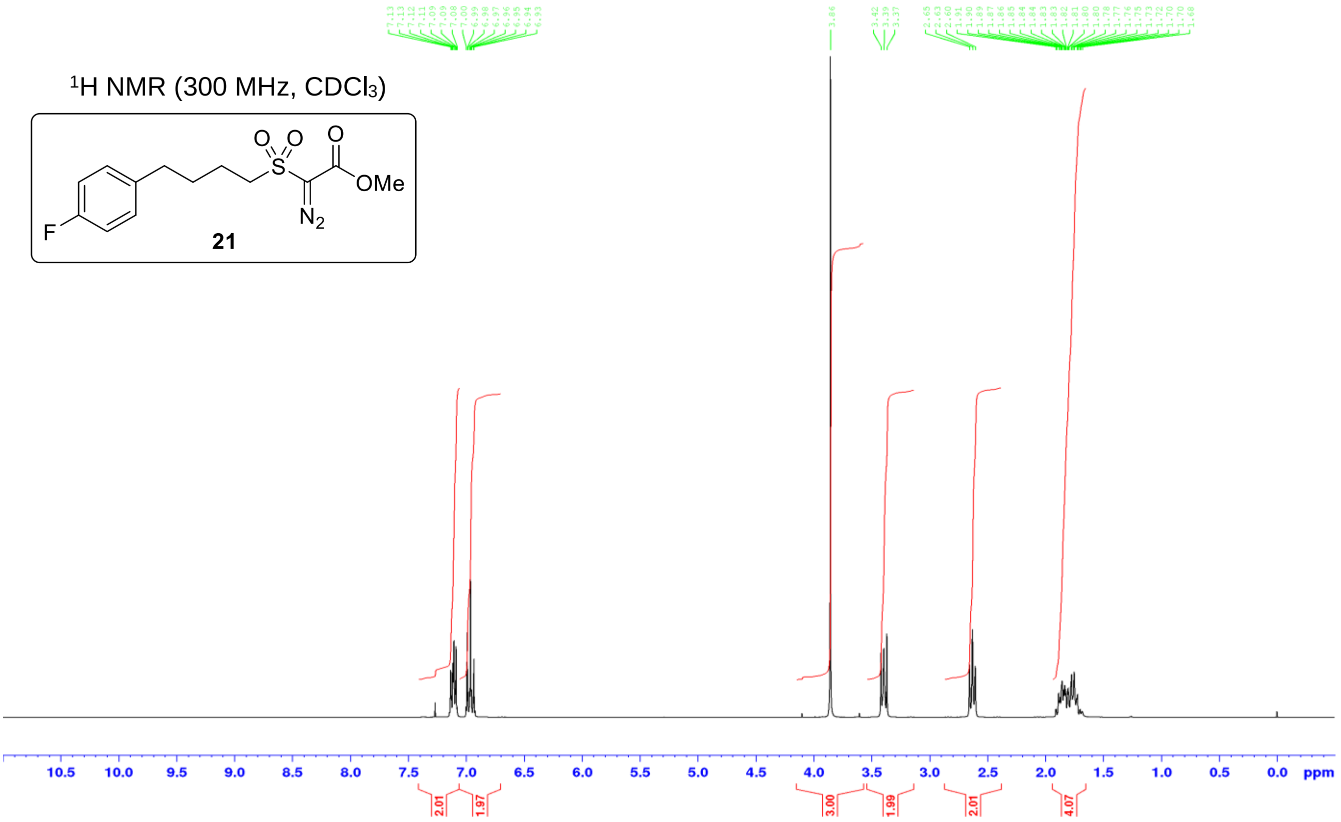

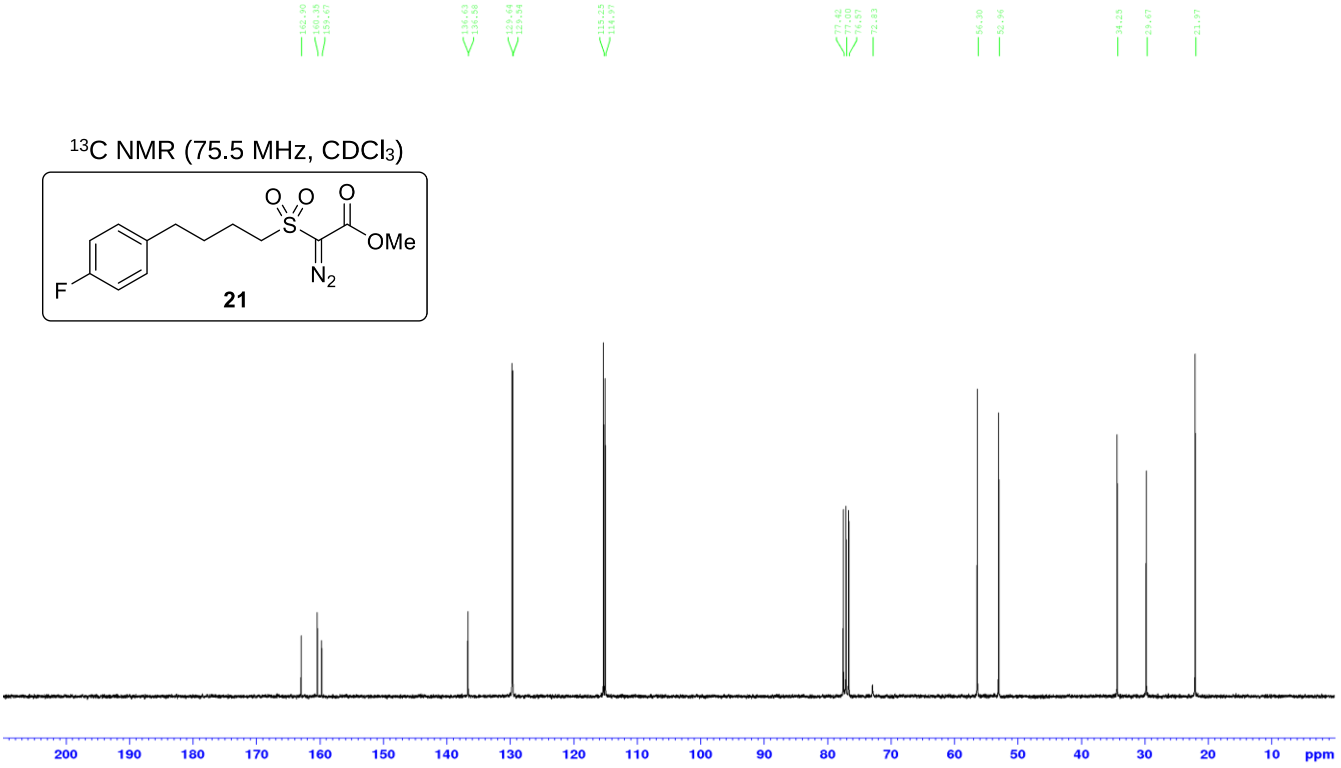

2-Diazo-1-phenyl-2-((4'-phenylbutyl)sulfonyl)ethan-1-one (22)

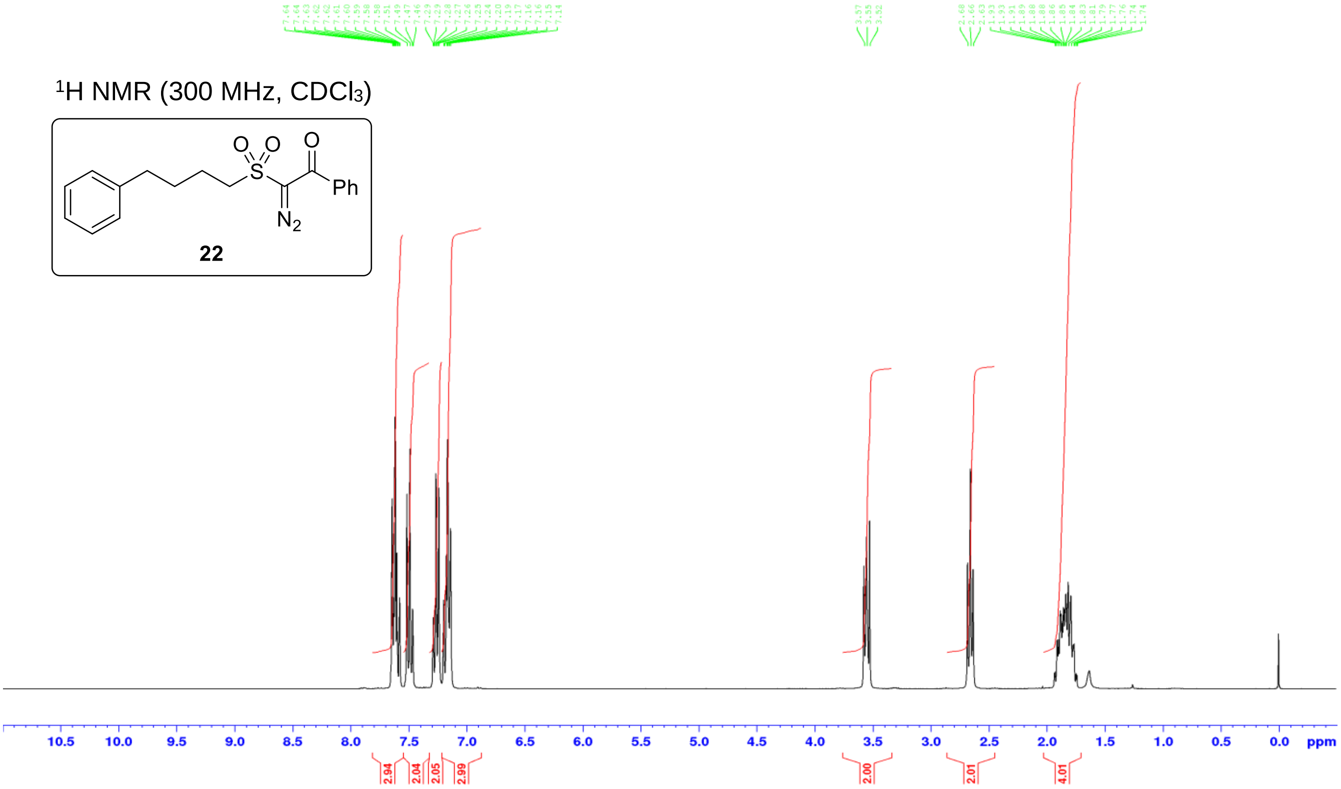

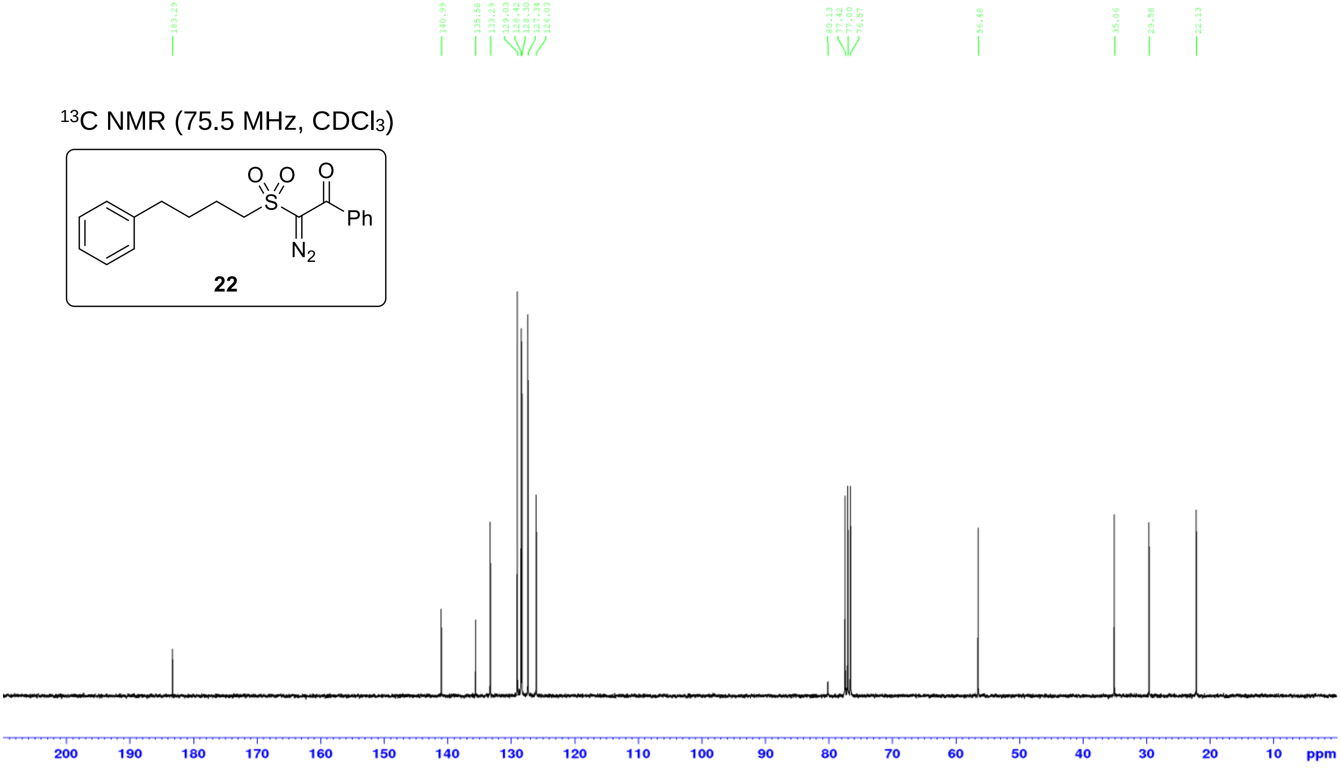

Benzyl 2-diazo-2-(dodecylsulfonyl)acetate (23)

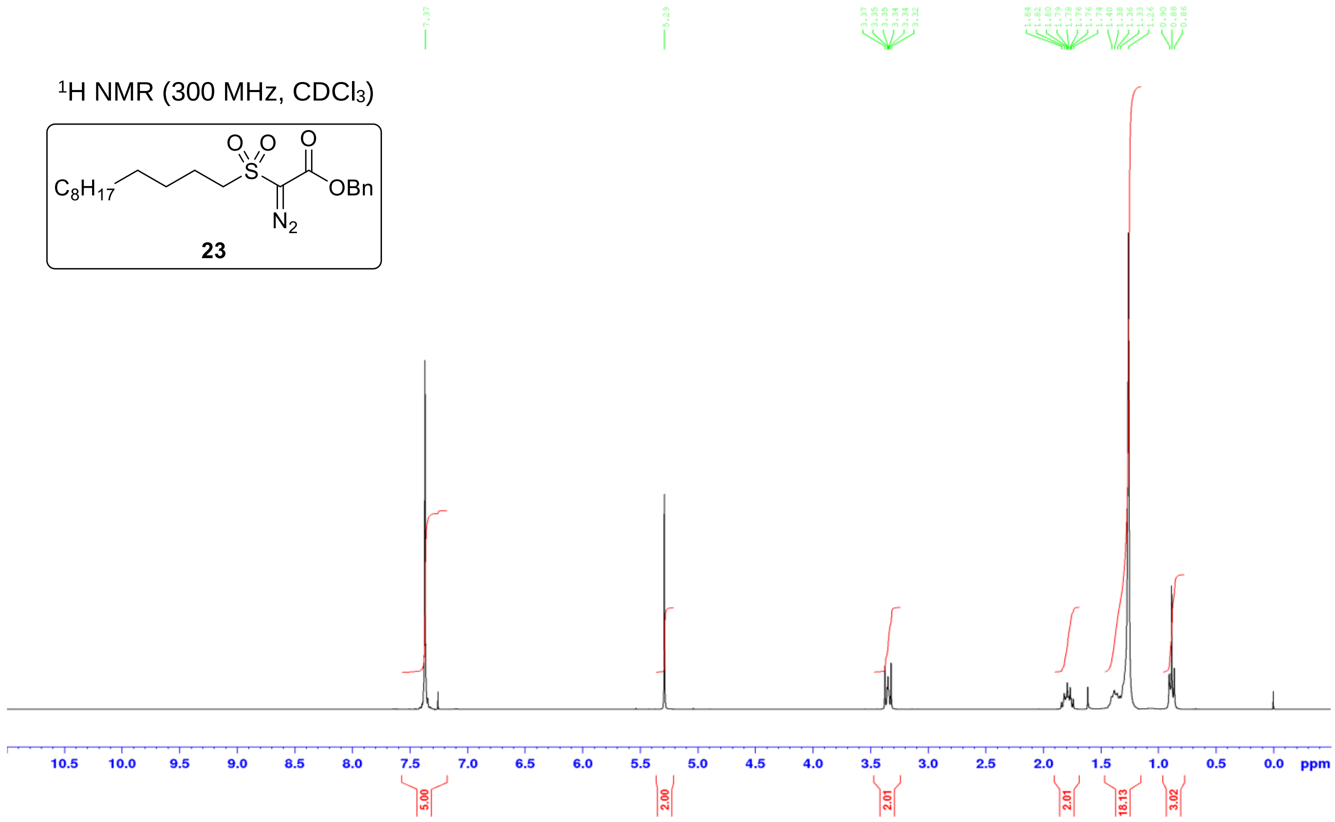

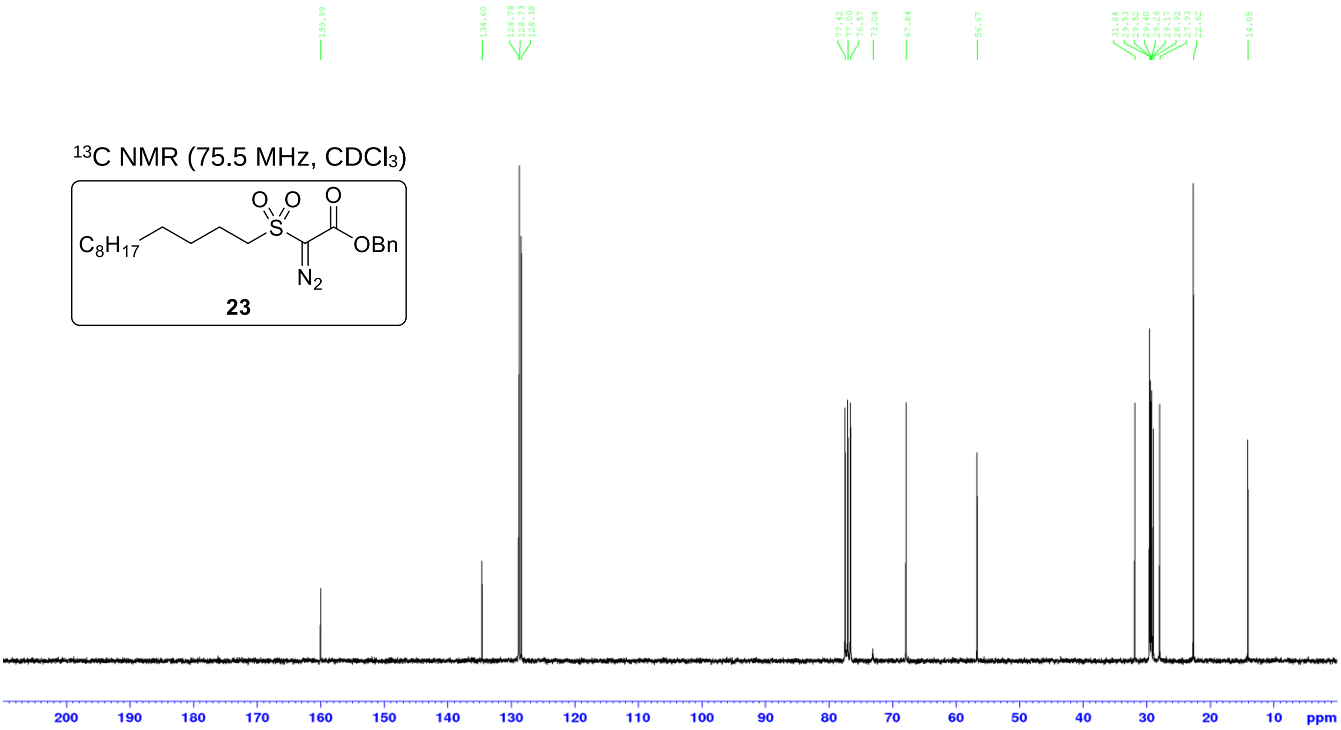

Methyl 2-((2'-cyclohexylethyl)sulfonyl)-2-diazoacetate (24)

^{1}H NMR (300 MHz, $CDCl_3$)

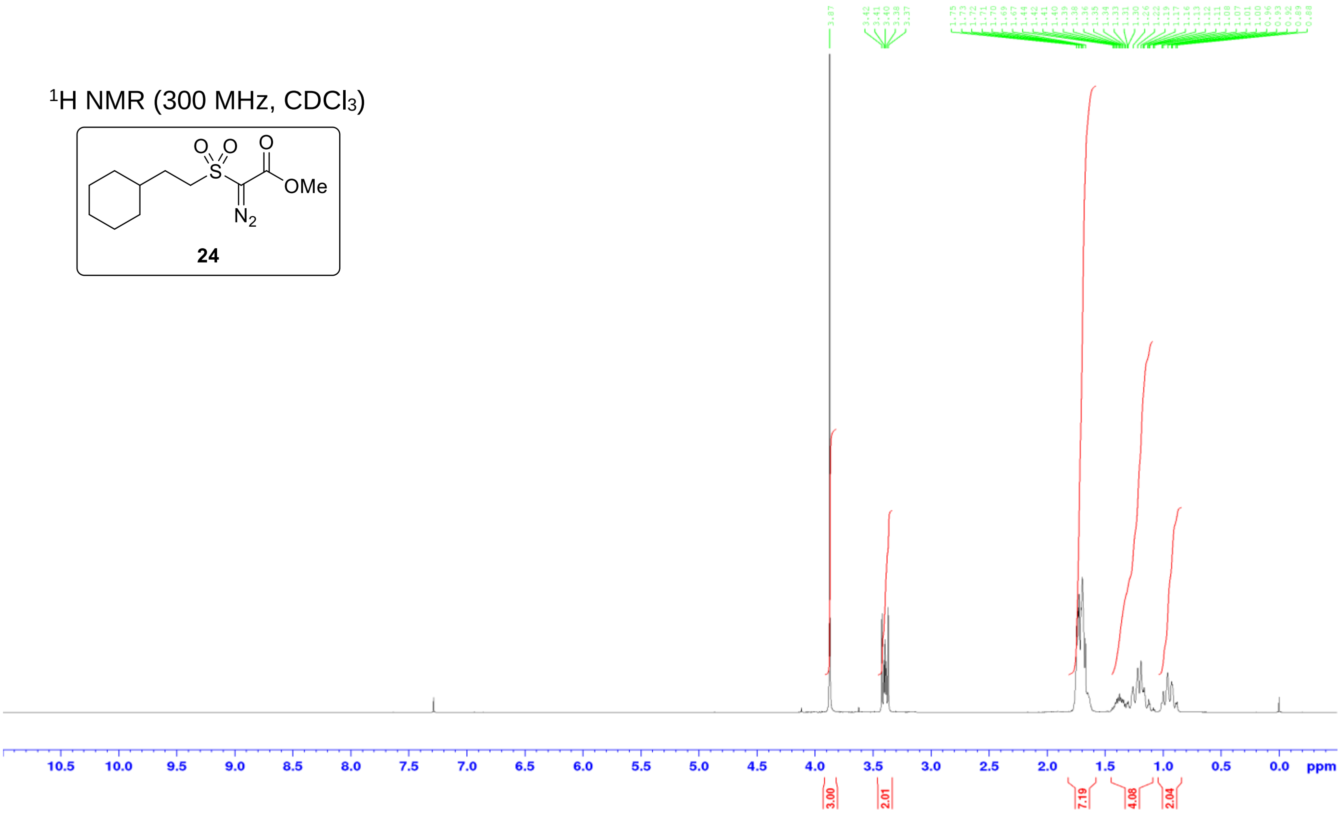

^{13}C NMR (75.5 MHz, $CDCl_3$)

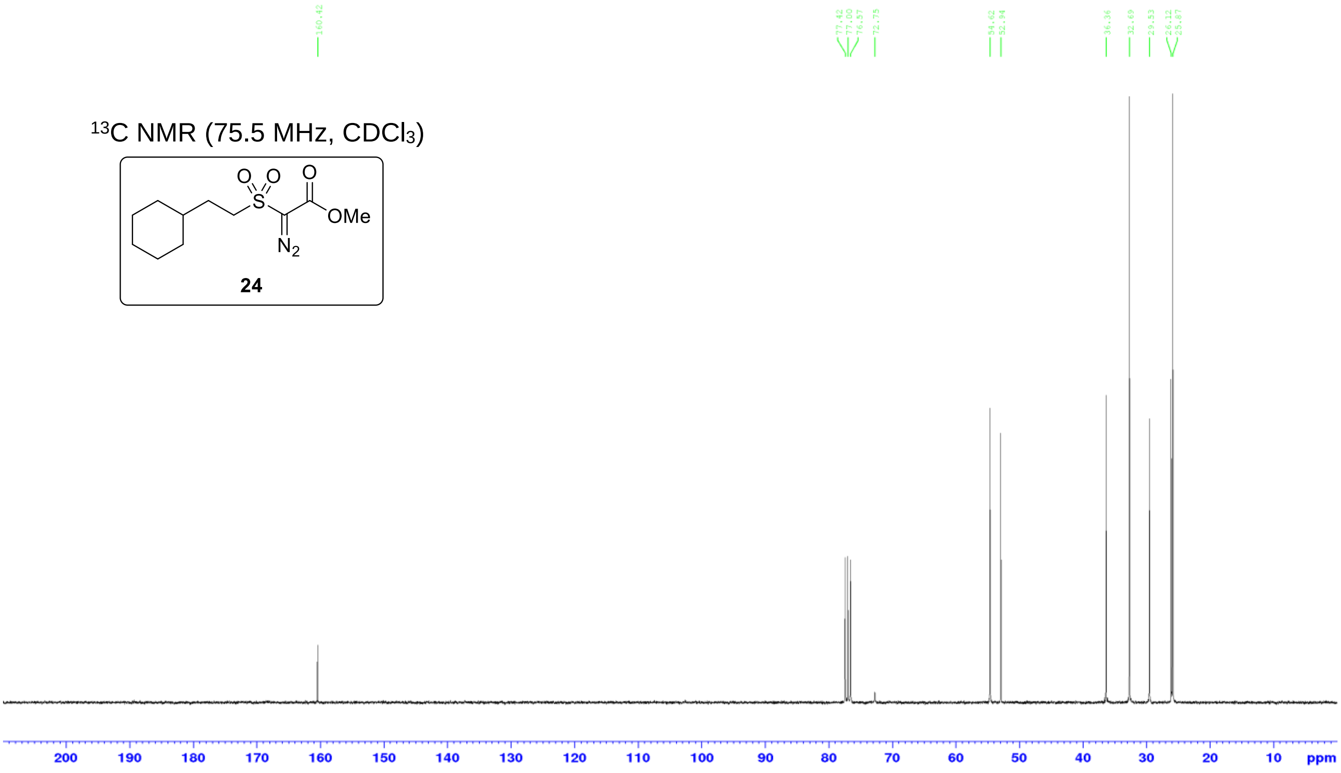

Methyl (2*R*,3*S*)-3-phenyltetrahydro-2*H*-thiopyran-2-carboxylate 1,1-dioxide (17a)

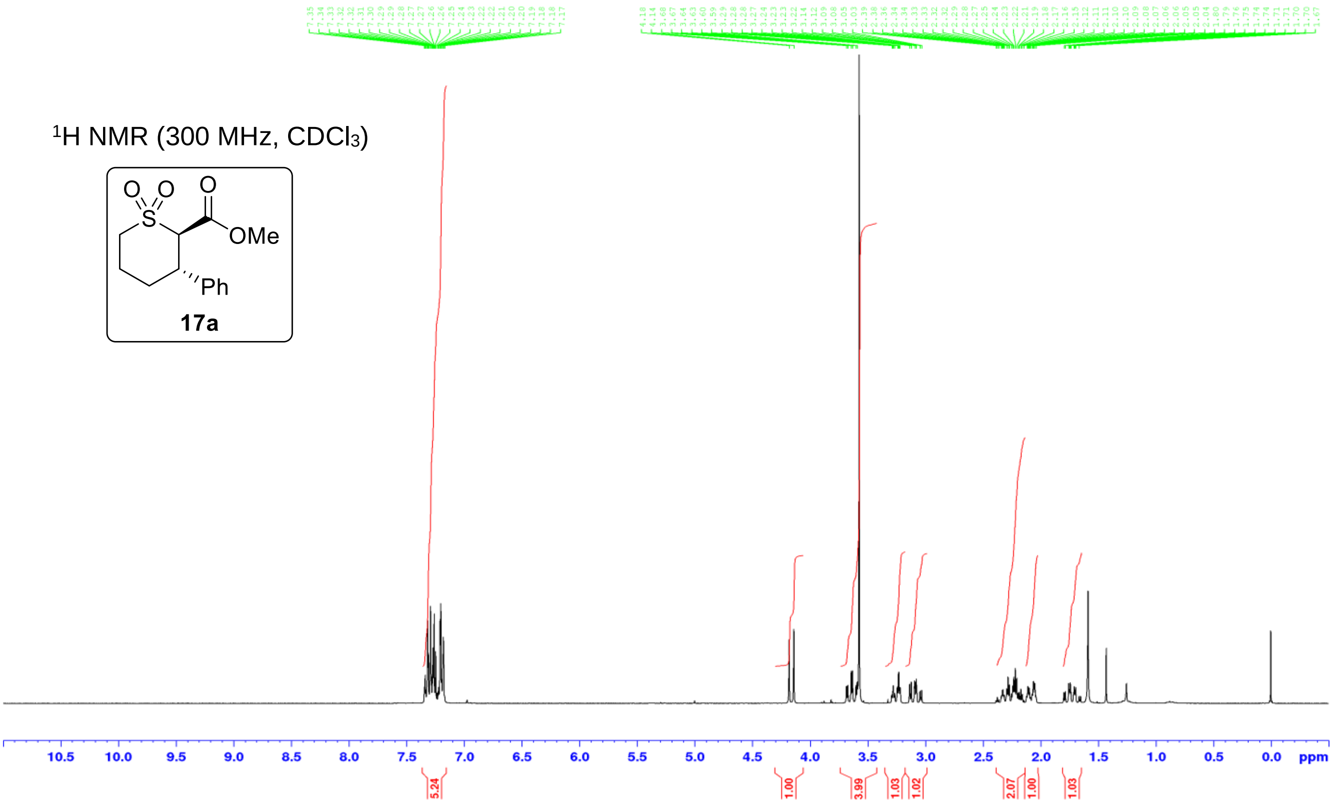

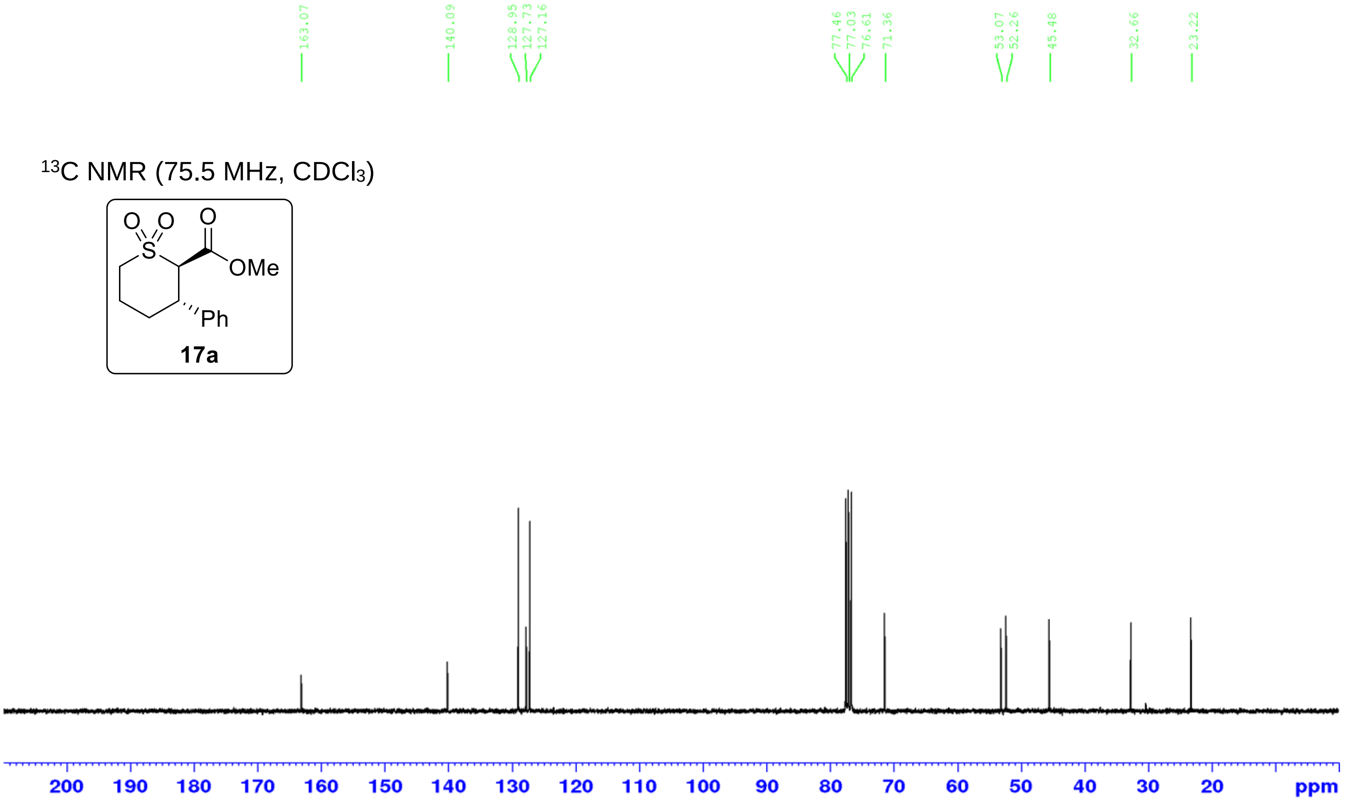

Methyl (2*S*,3*S*)-3-phenyltetrahydro-2*H*-thiopyran-2-carboxylate 1,1-dioxide (17b)

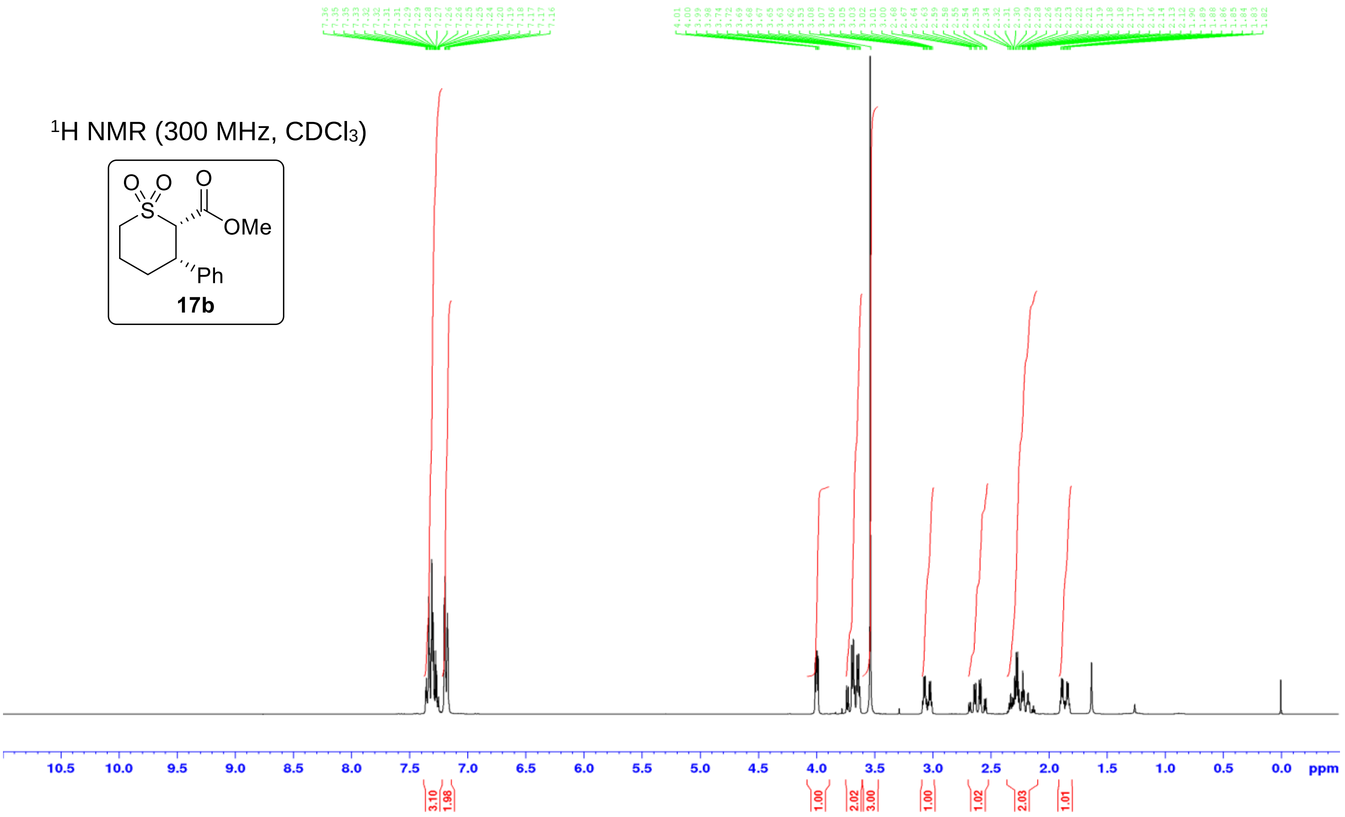

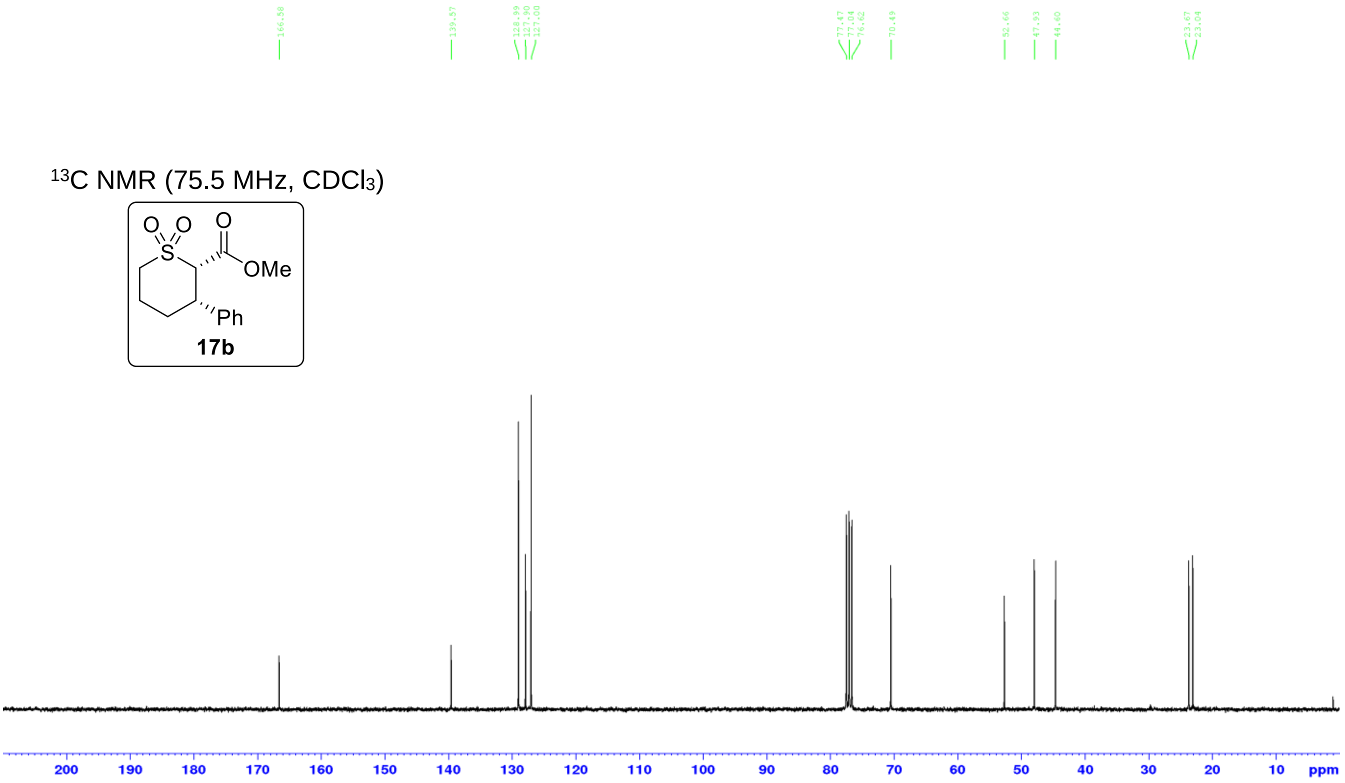

For the sulfolanes in some instances signals for the minor diastereoisomer can be seen in the spectra; these are reported in the experimetal section.

Methyl (2*R,3*R**)-3-benzyltetrahydrothiophene-2-carboxylate 1,1-dioxide (18a)**

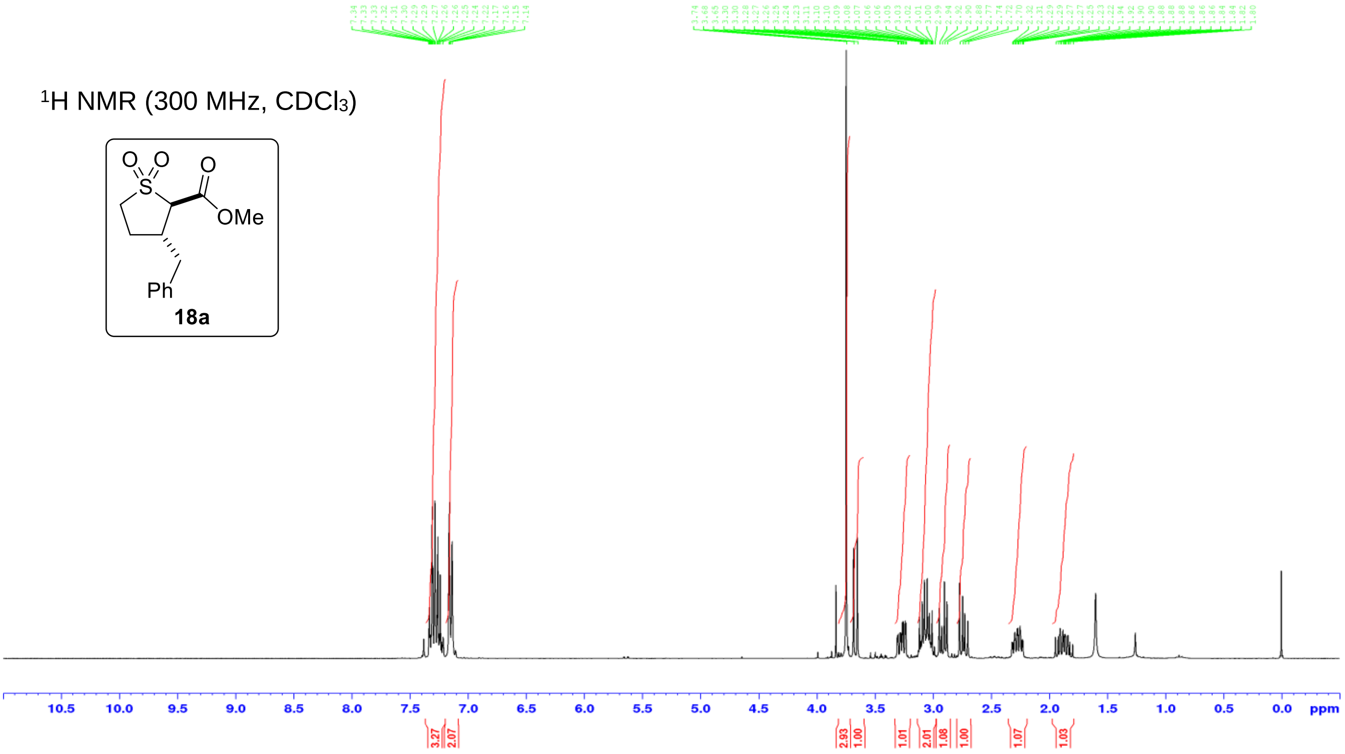

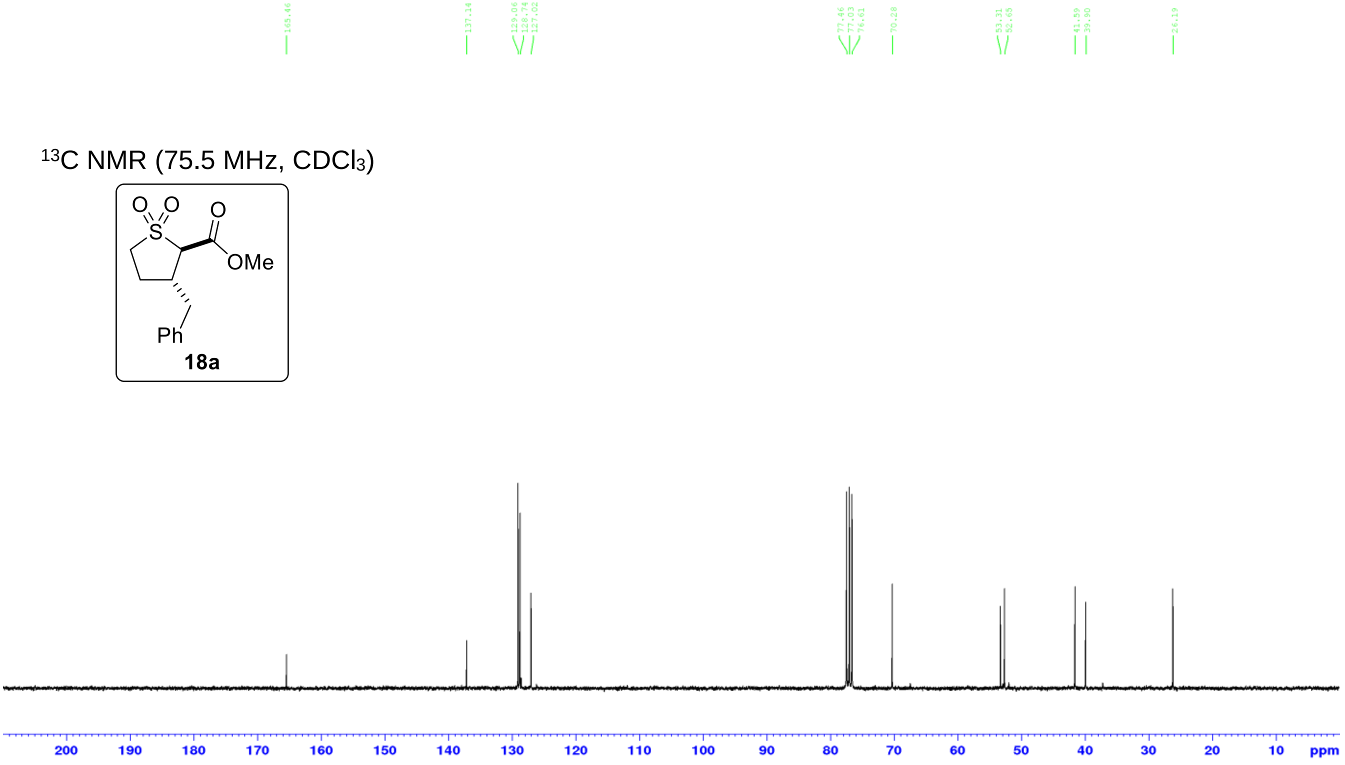

Methyl 2-((4'-phenylbut-3'-en-1'-yl)sulfonyl)acetate (S22)

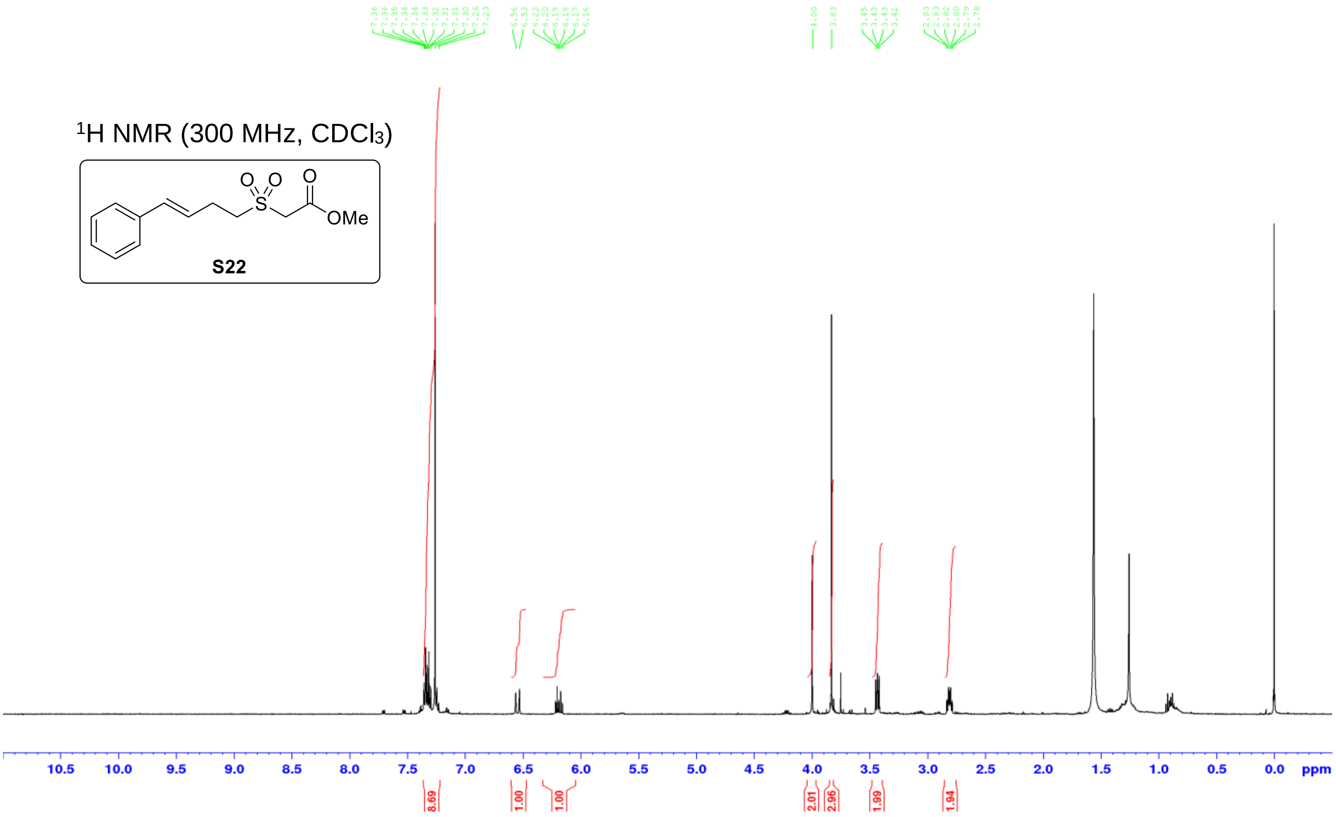

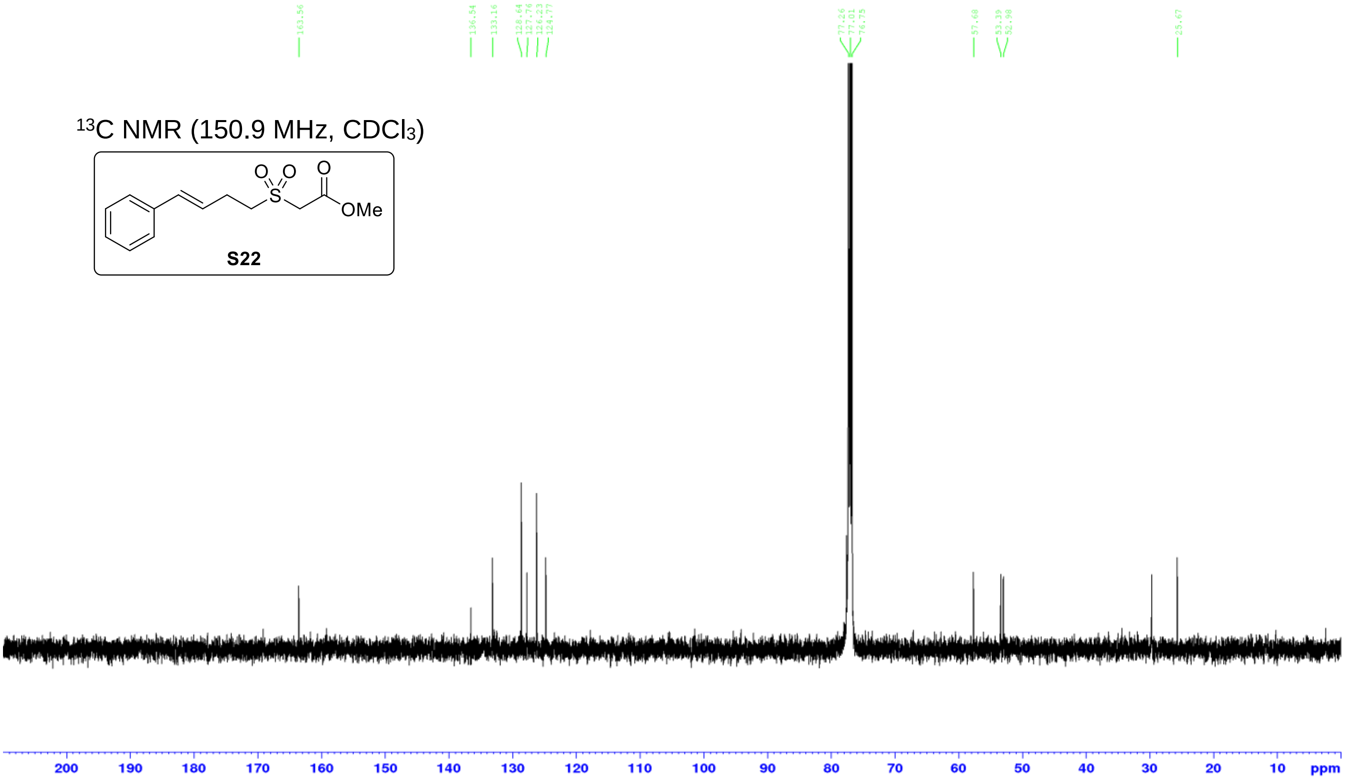

Methyl (2*R*,3*S*)-3-(*p*-tolyl)tetrahydro-2*H*-thiopyran-2-carboxylate 1,1-dioxide (25a)

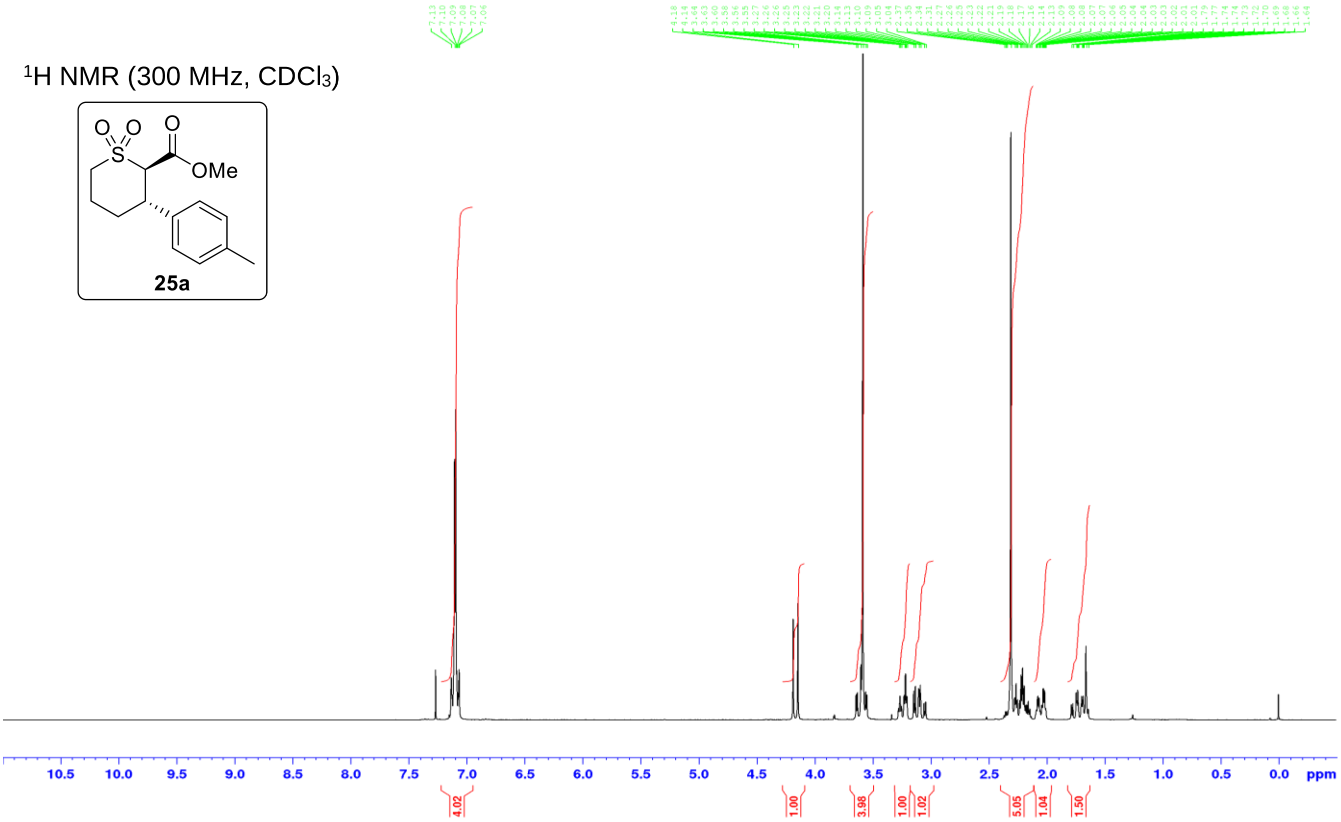

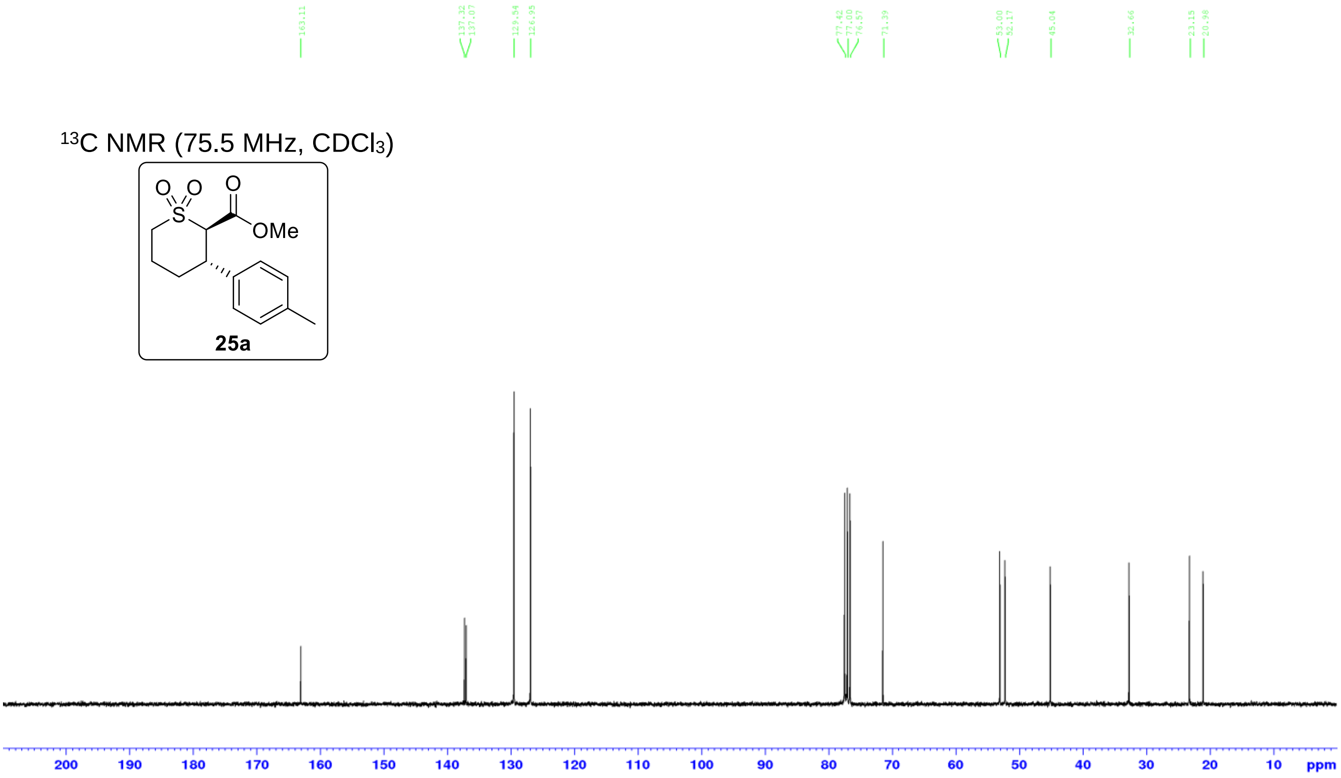

Methyl (2*R*,3*R*)-3-(*p*-tolyl)tetrahydro-2*H*-thiopyran-2-carboxylate 1,1-dioxide (25b)

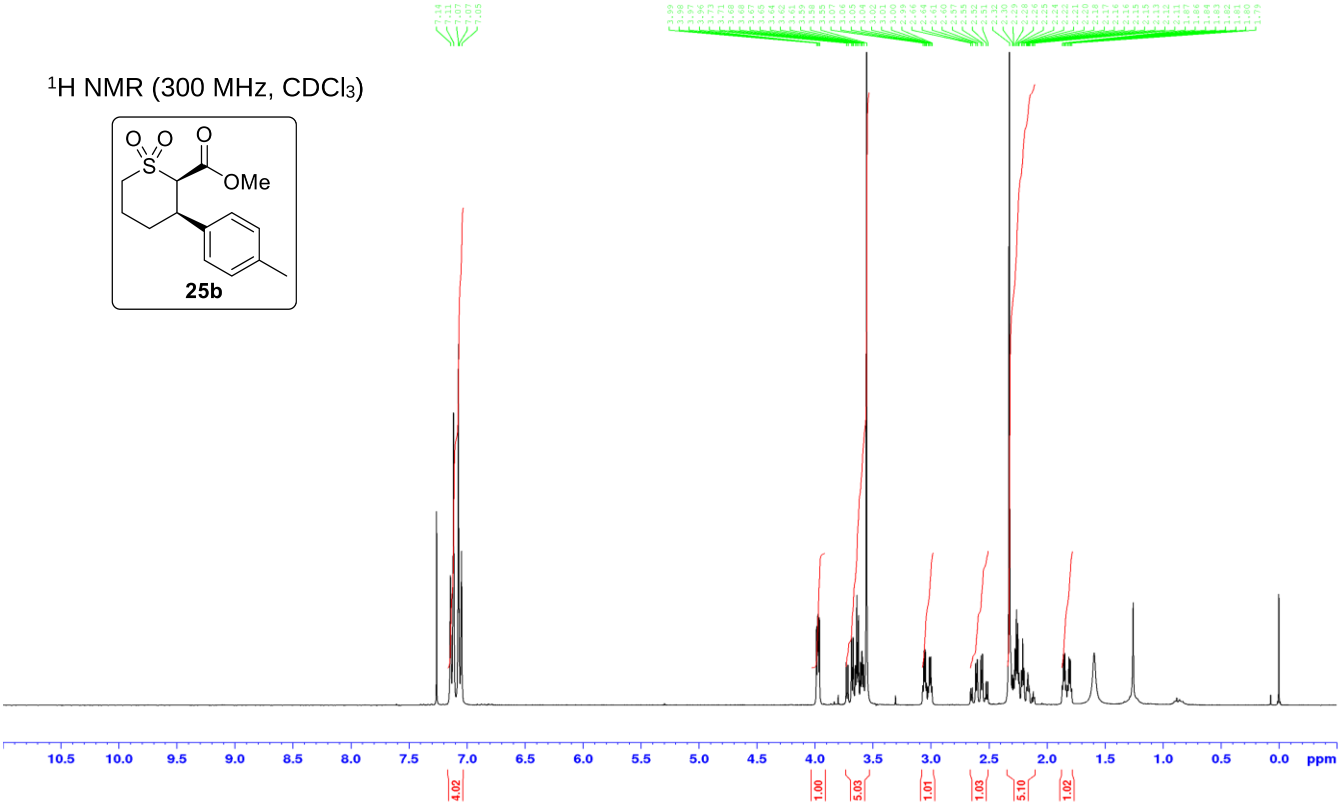

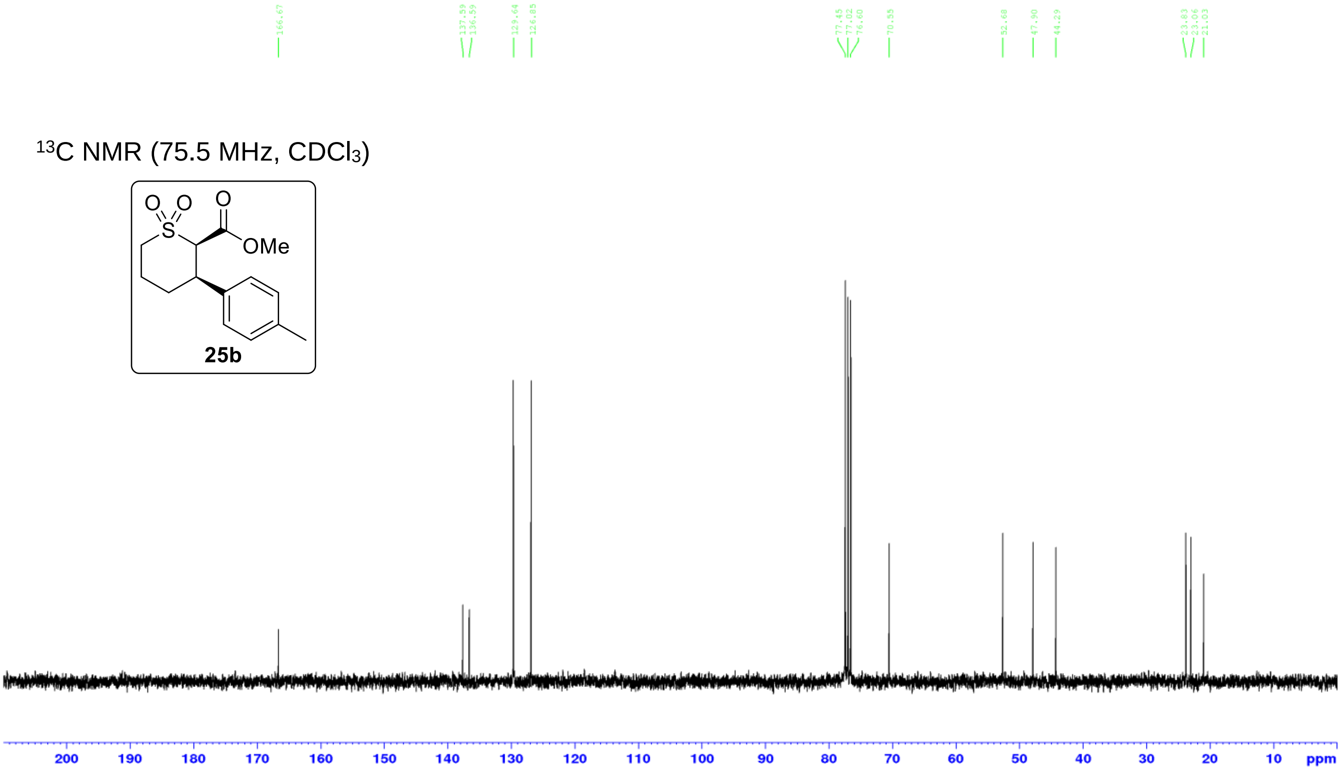

Methyl (2*R,3*R**)-3-(4'-methylbenzyl)tetrahydrothiophene-2-carboxylate 1,1-dioxide (S23a)**

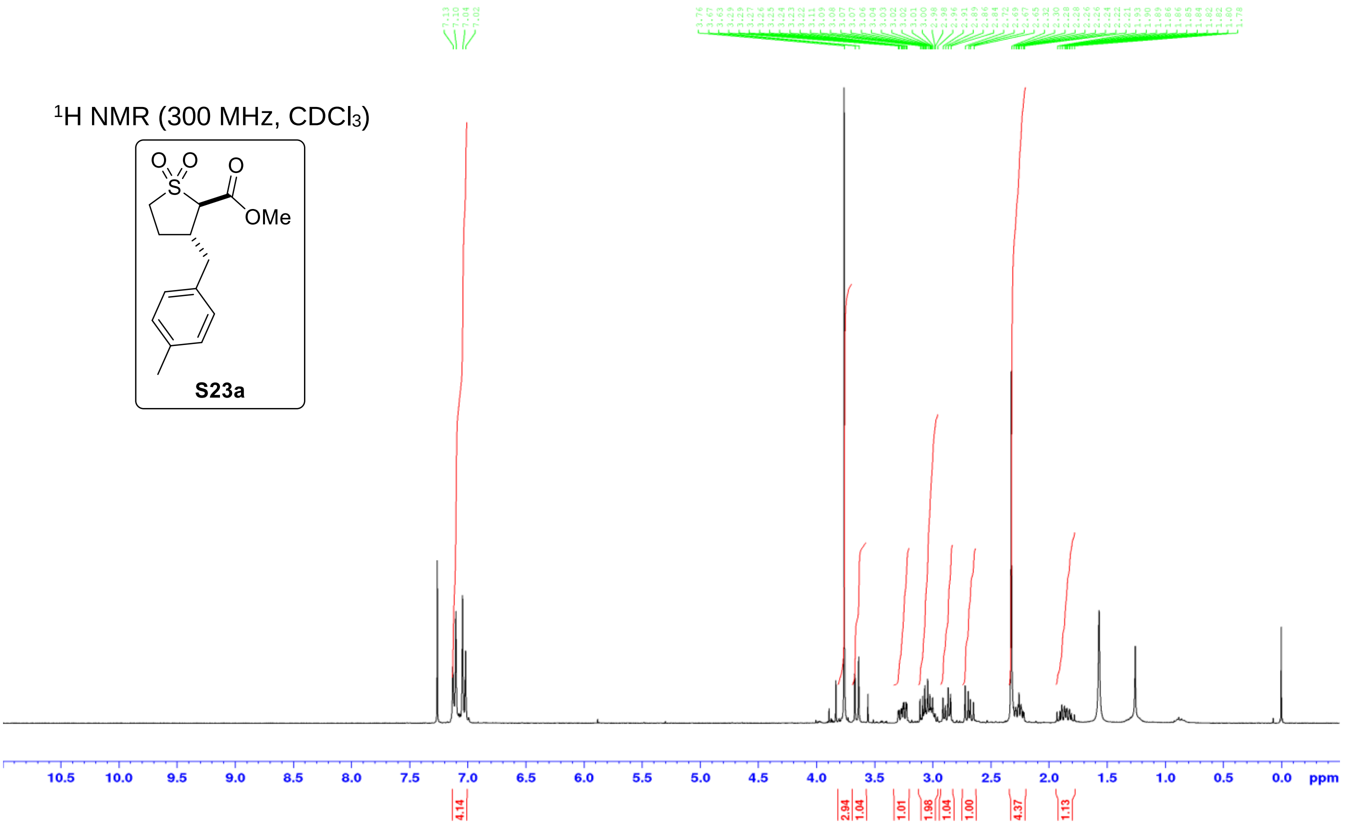

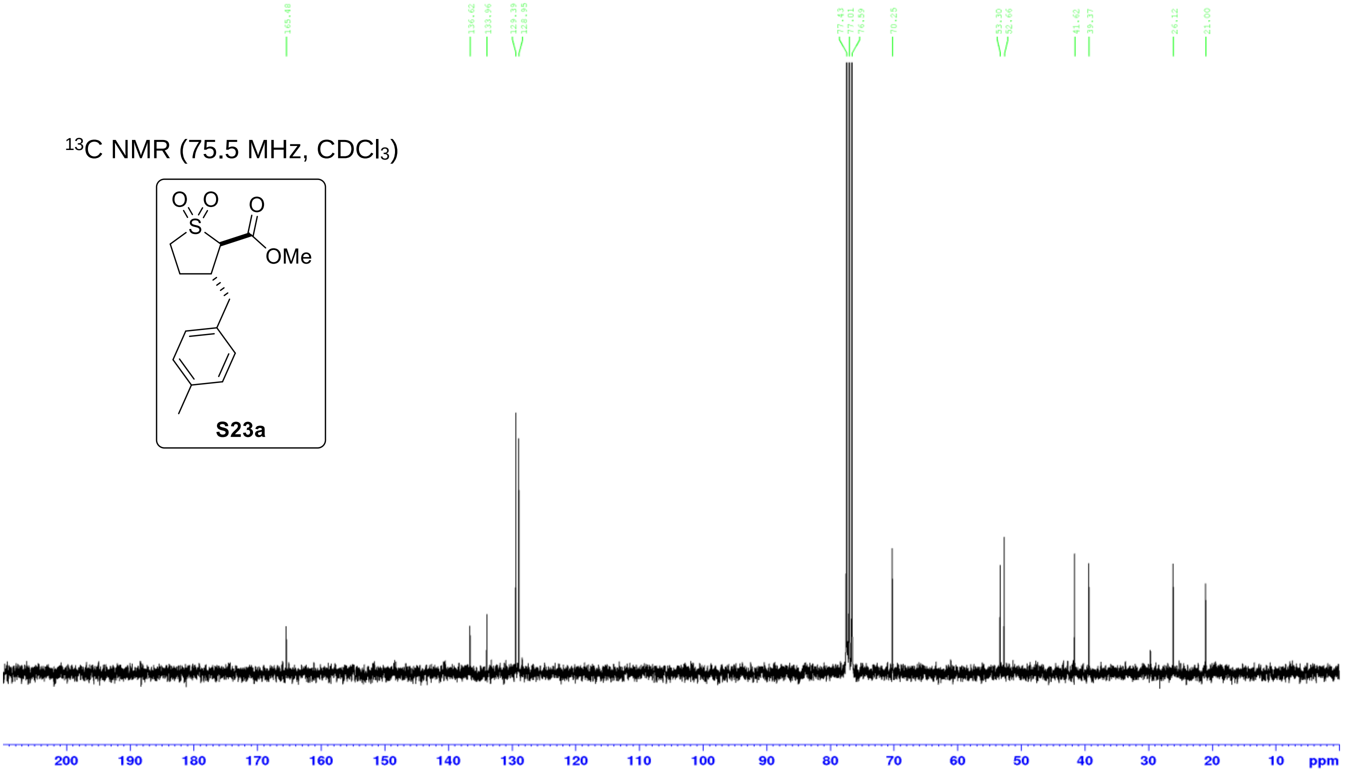

Methyl 2-((4'-(*p*-tolyl)but-3'-en-1'-yl)sulfonyl)acetate (S24)

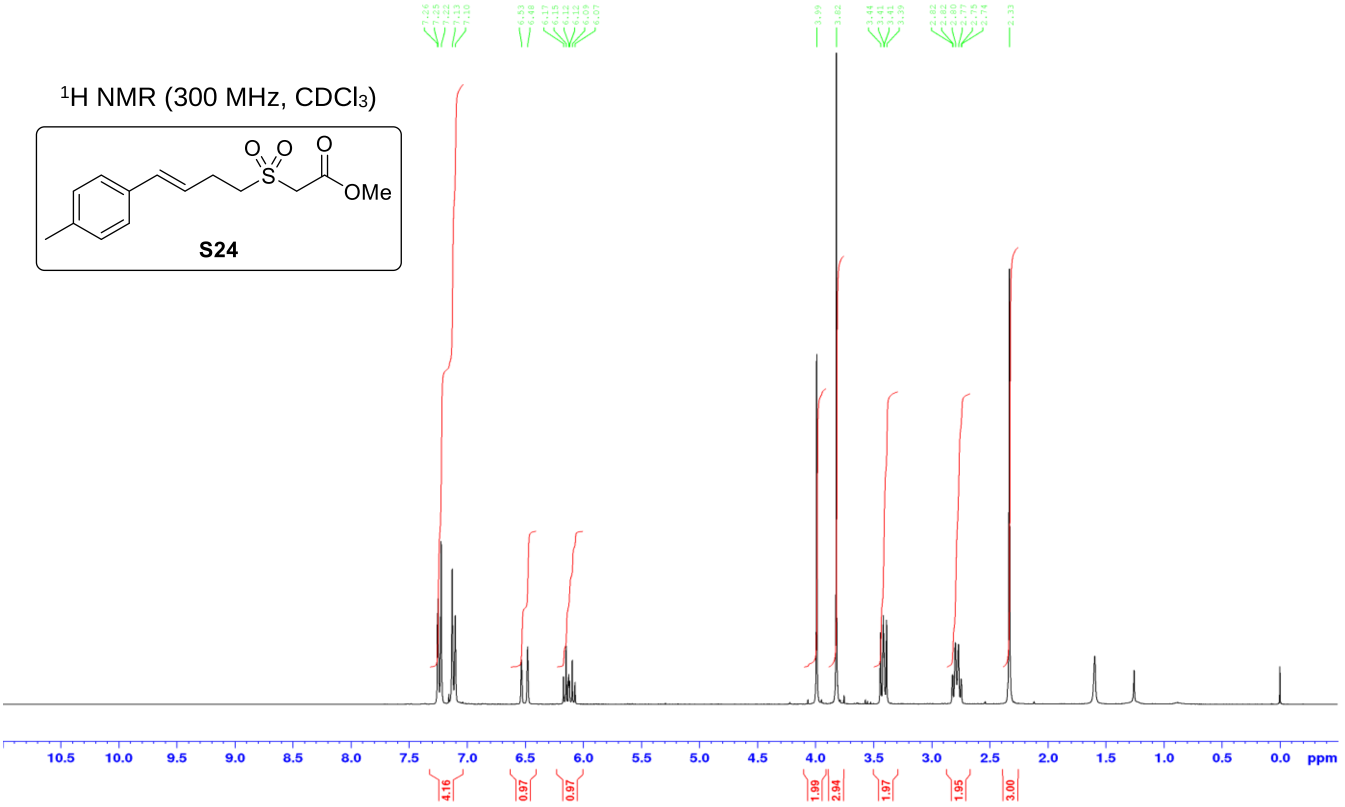

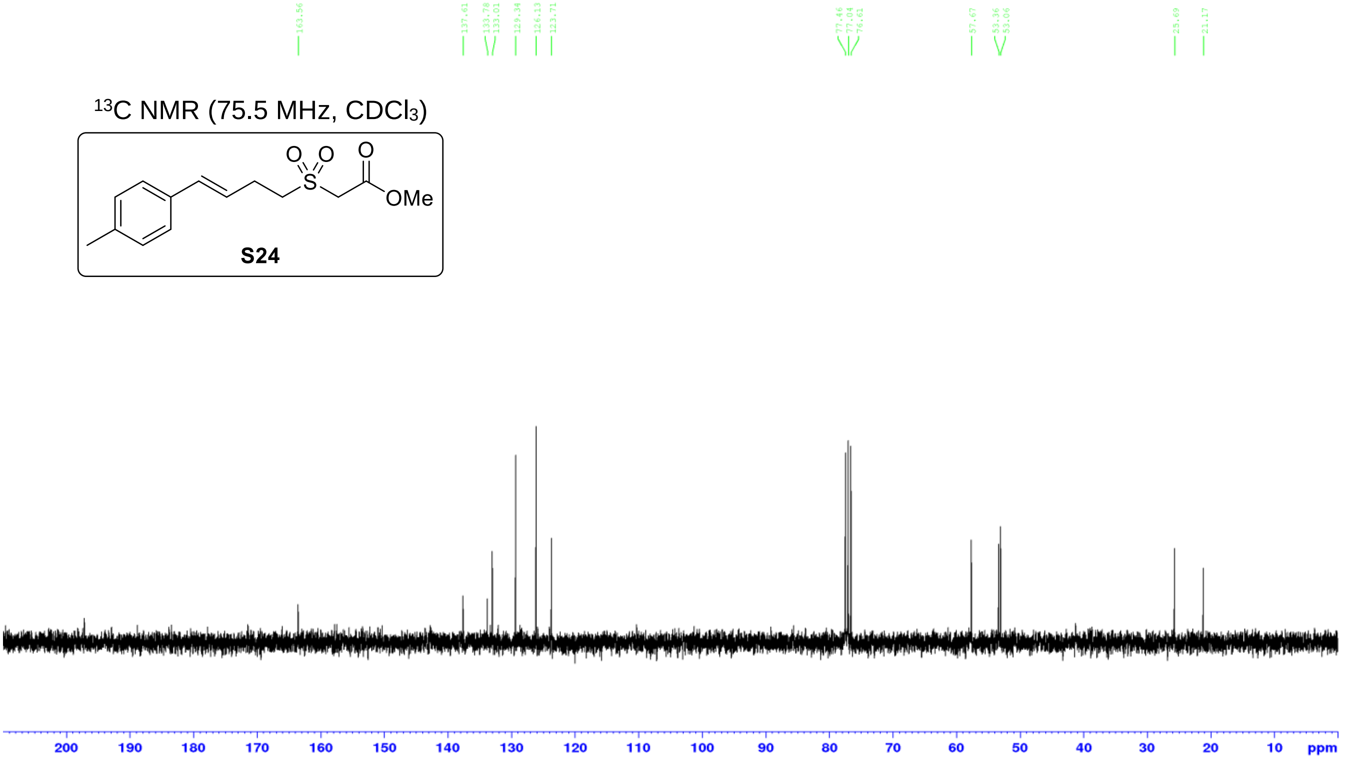

Methyl (2*R*,3*S*)-3-(4'-methoxyphenyl)tetrahydro-2*H*-thiopyran-2-carboxylate 1,1-dioxide (26a)

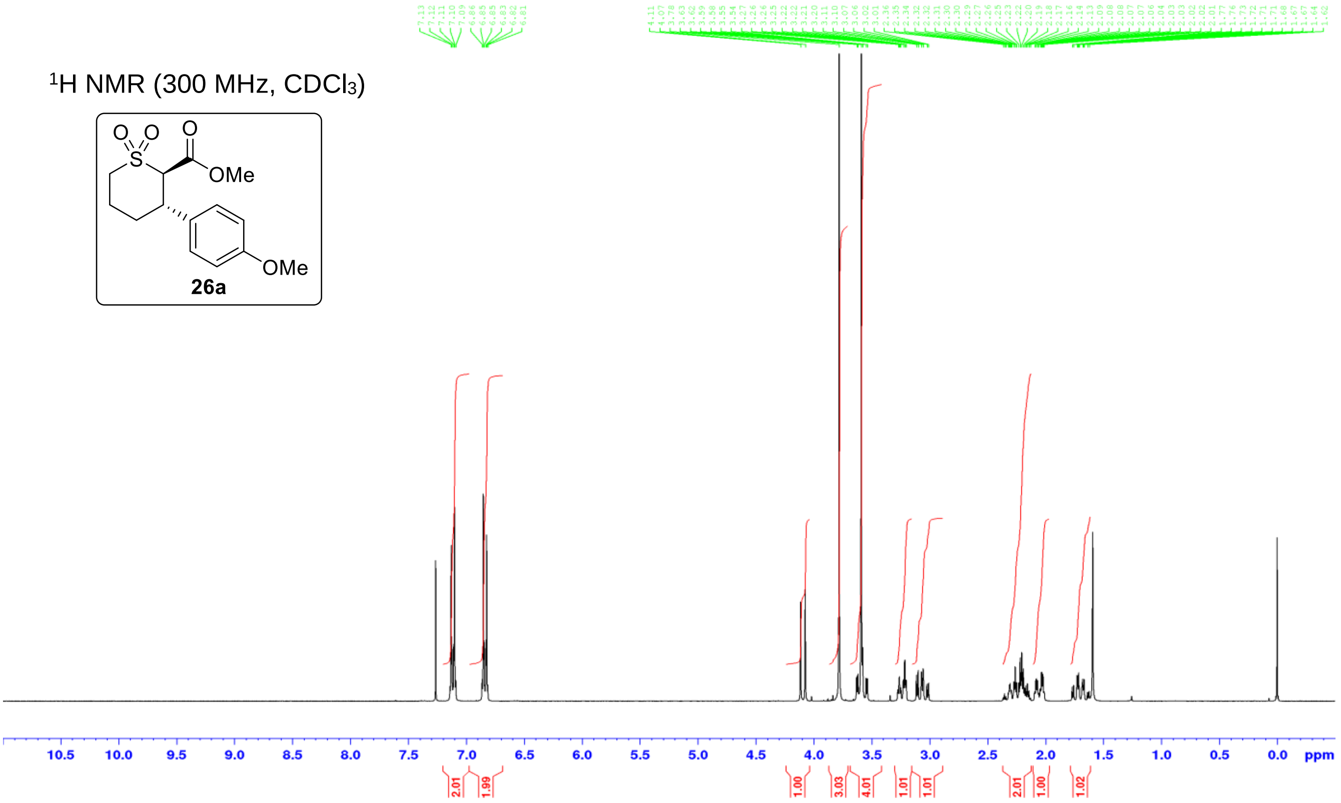

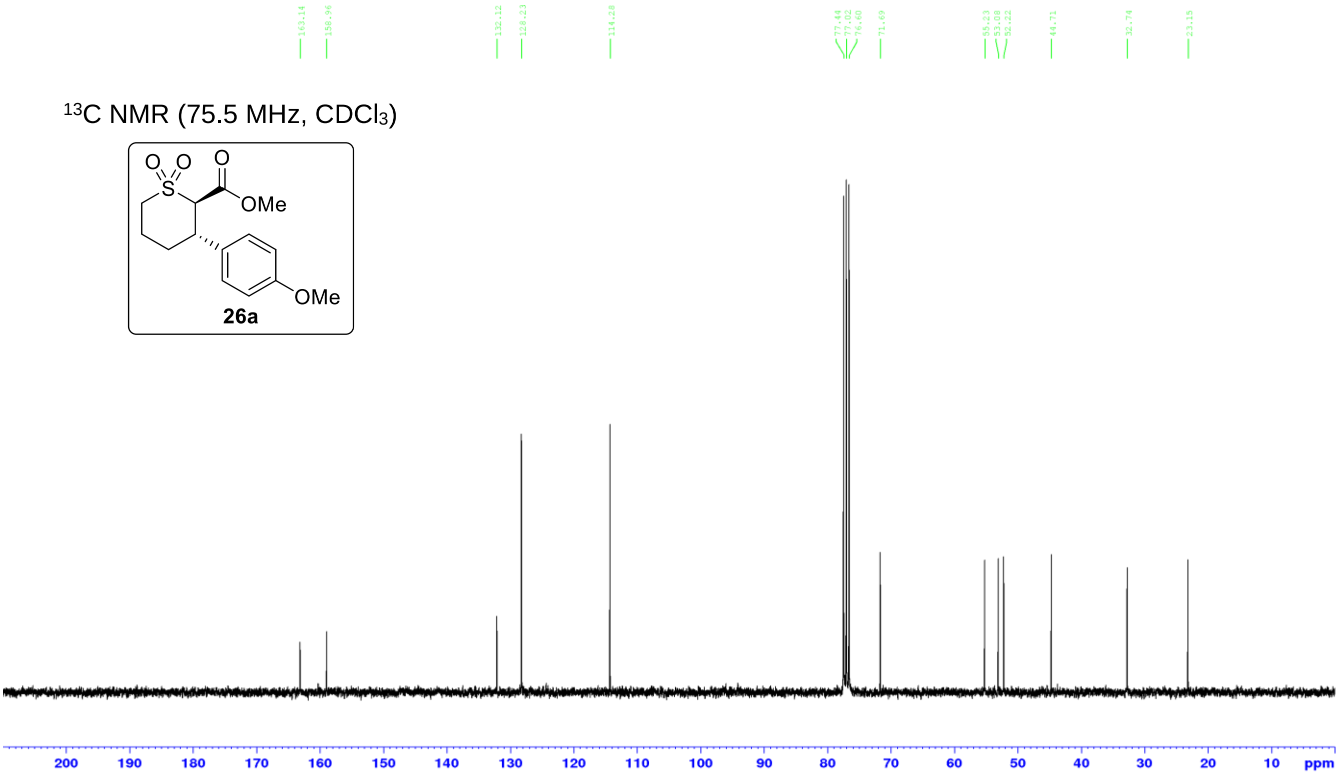

Methyl (2*S*,3*S*)-3-(4'-methoxyphenyl)tetrahydro-2*H*-thiopyran-2-carboxylate 1,1-dioxide (26b)

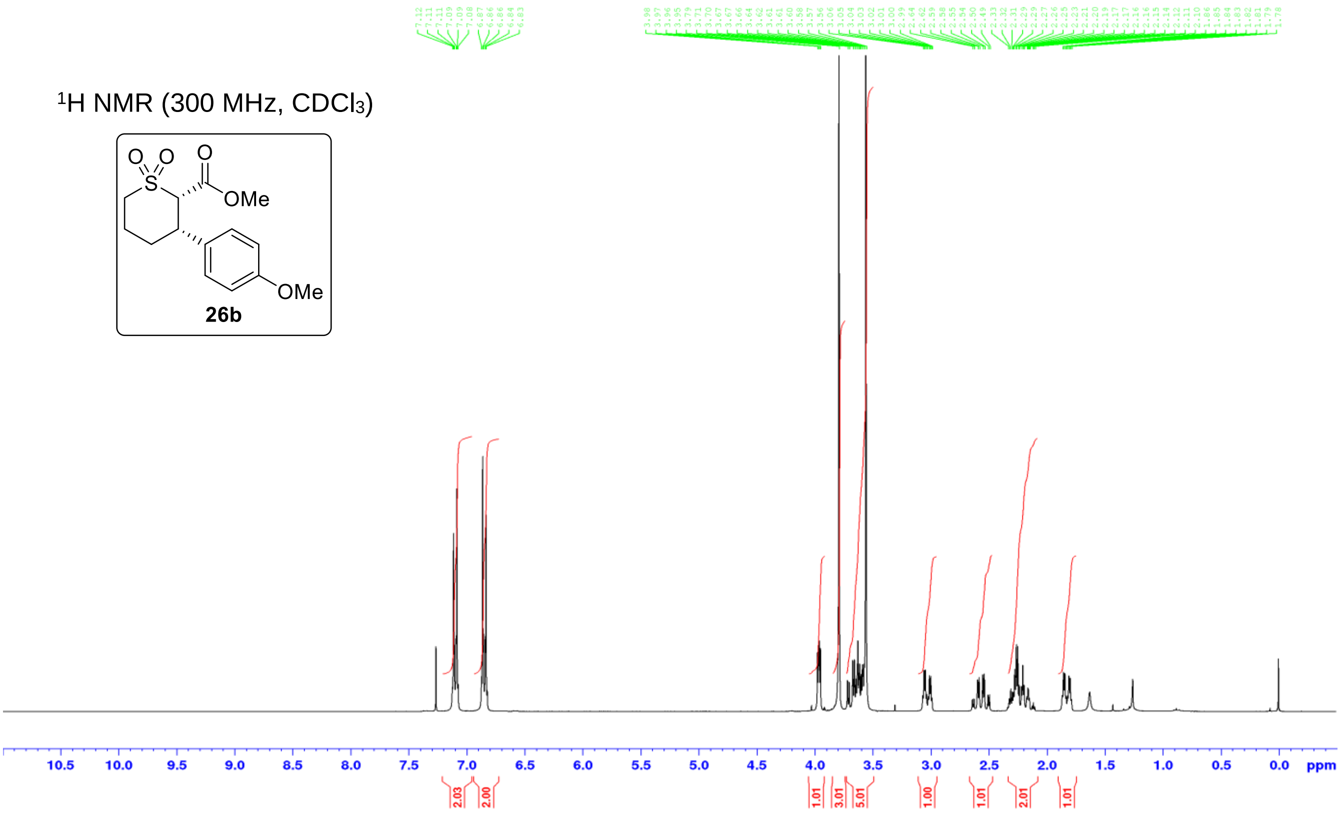

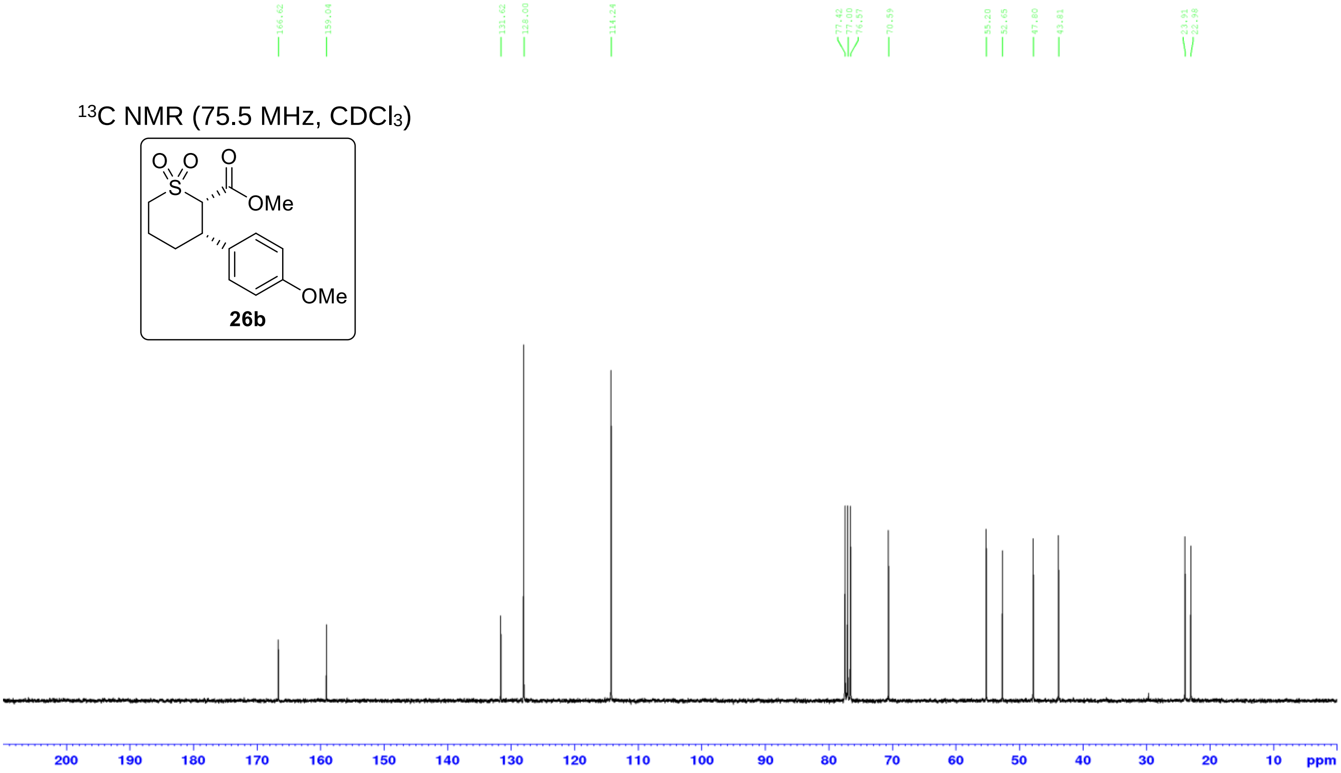

Methyl (2*R,3*R**)-3-(4'-methoxybenzyl)tetrahydrothiophene-2-carboxylate 1,1-dioxide (S25a)**

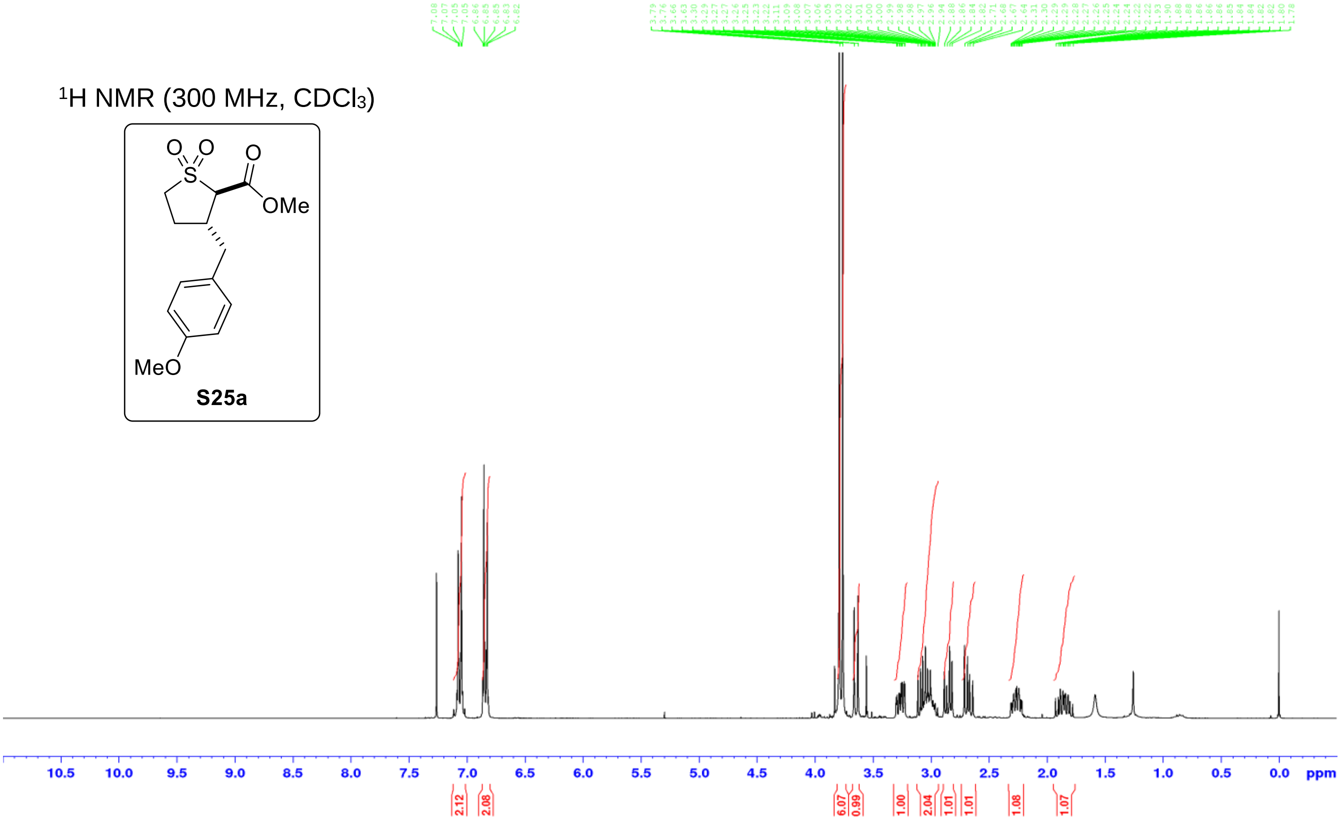

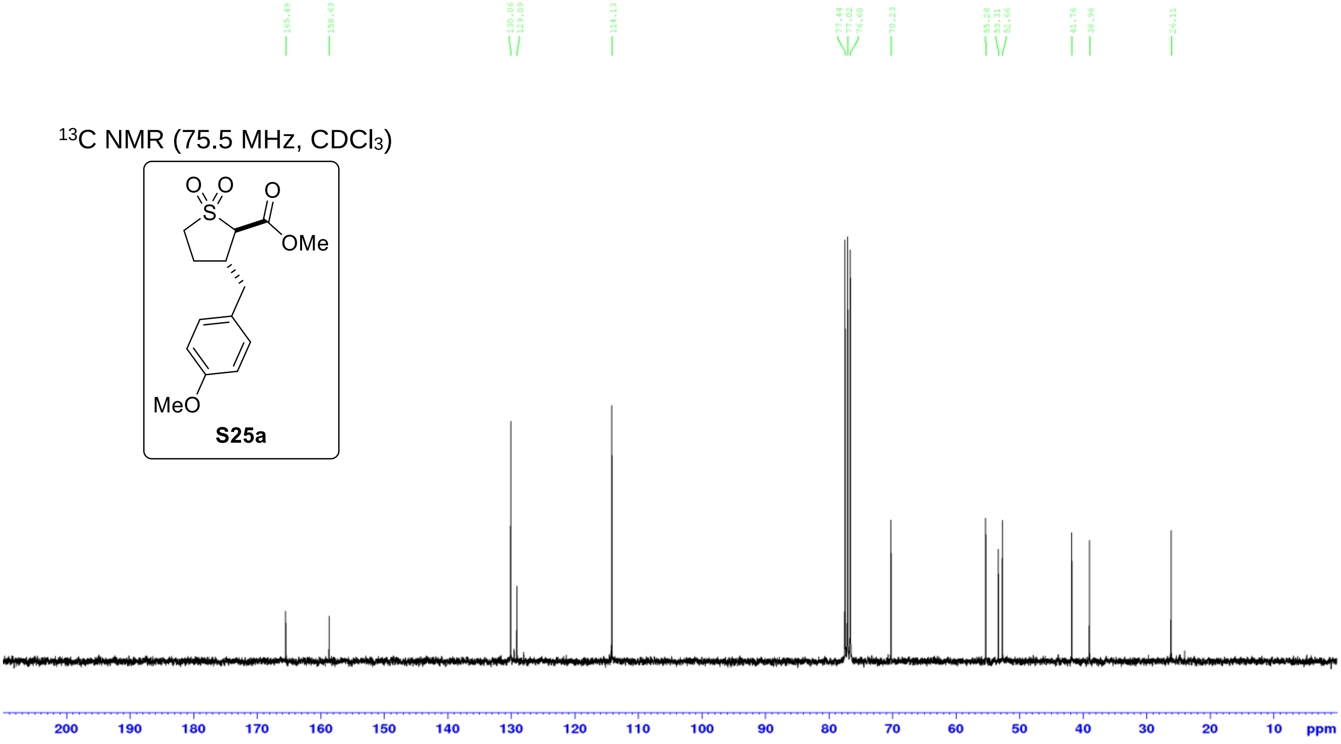

Methyl 2-((4'-(4''-methoxyphenyl)but-3'-en-1'-yl)sulfonyl)acetate (S26)

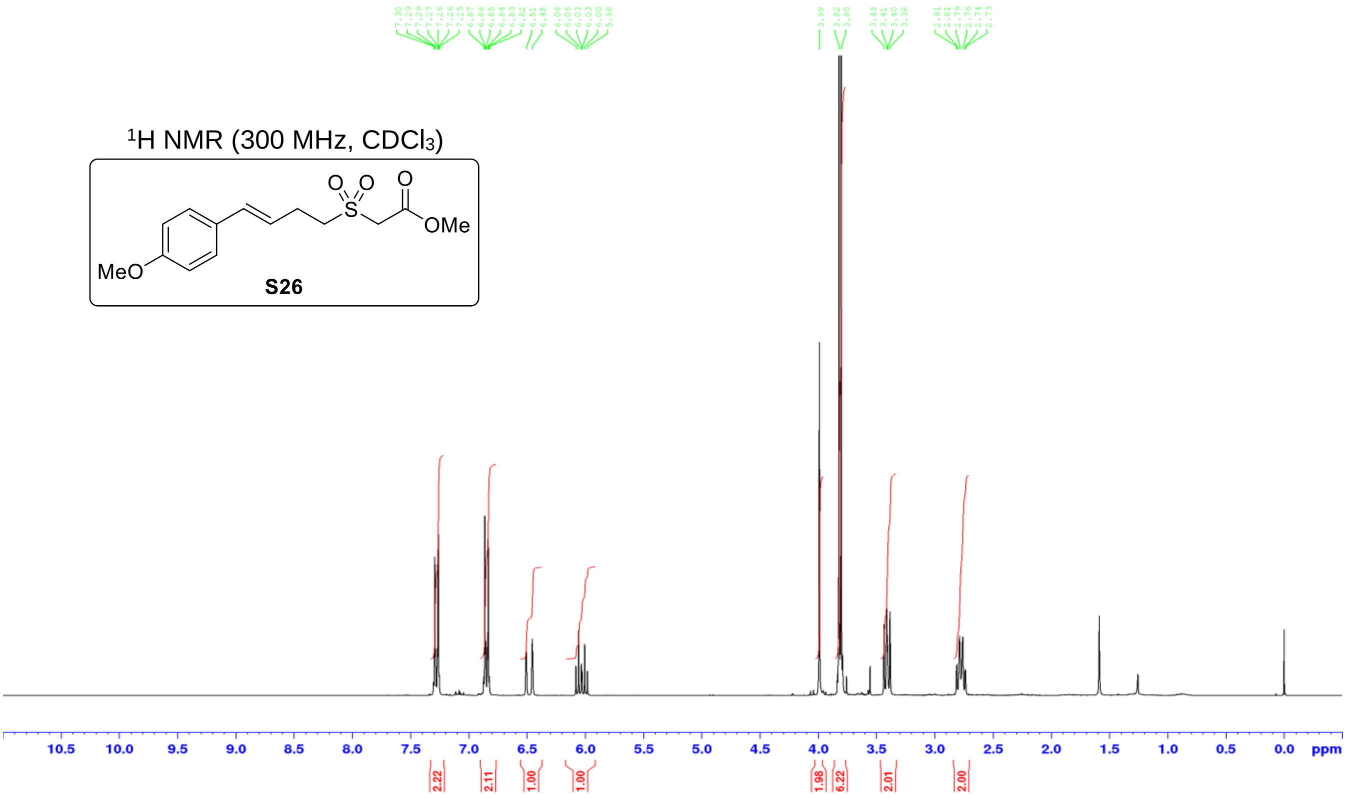

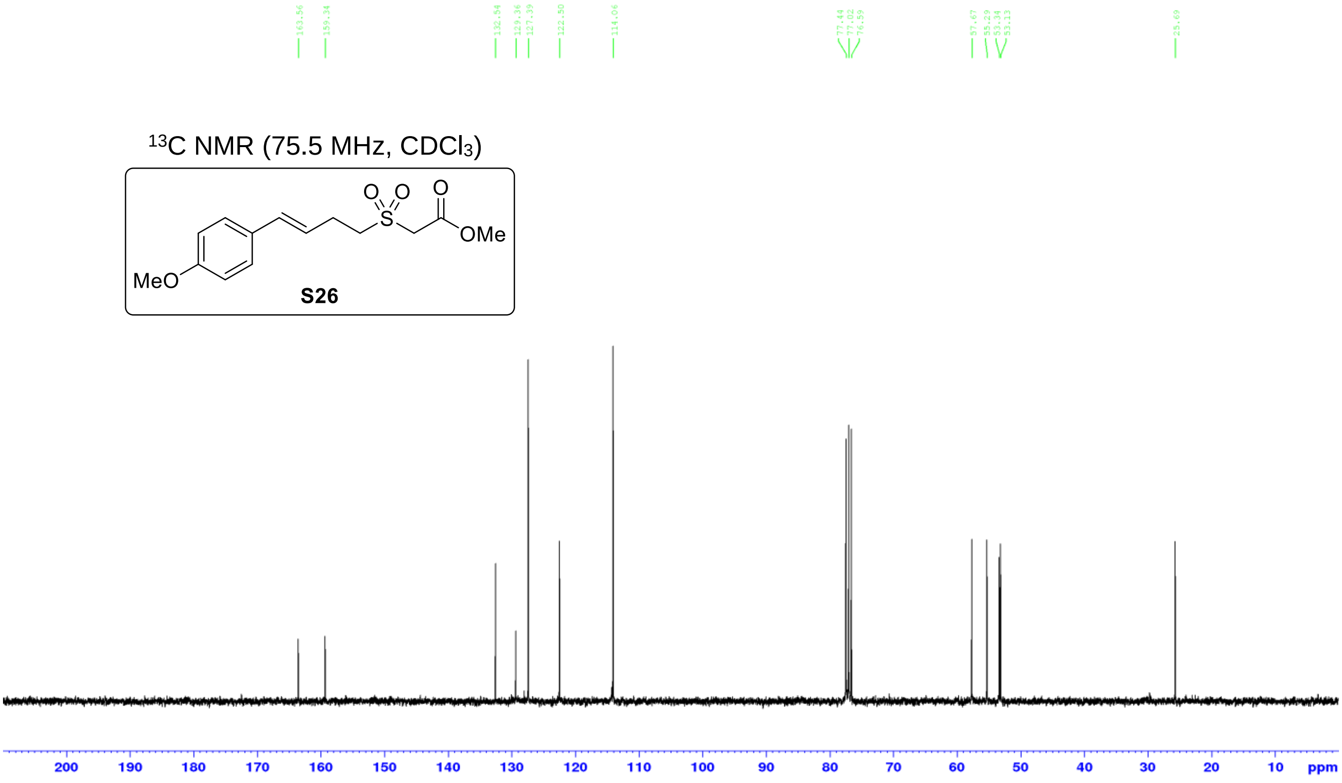

Methyl (2*R*,3*S*)-3-(4'-fluorophenyl)tetrahydro-2*H*-thiopyran-2-carboxylate 1,1-dioxide (27a)

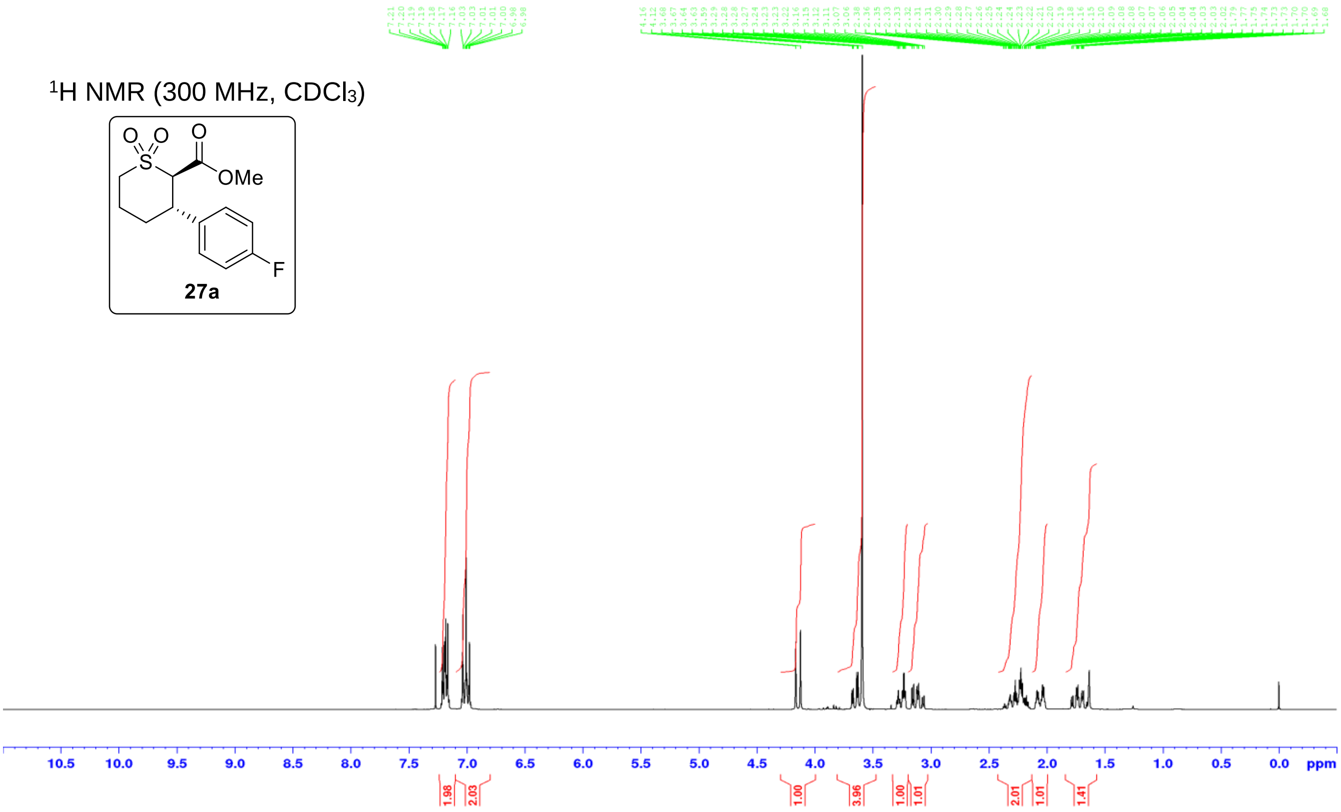

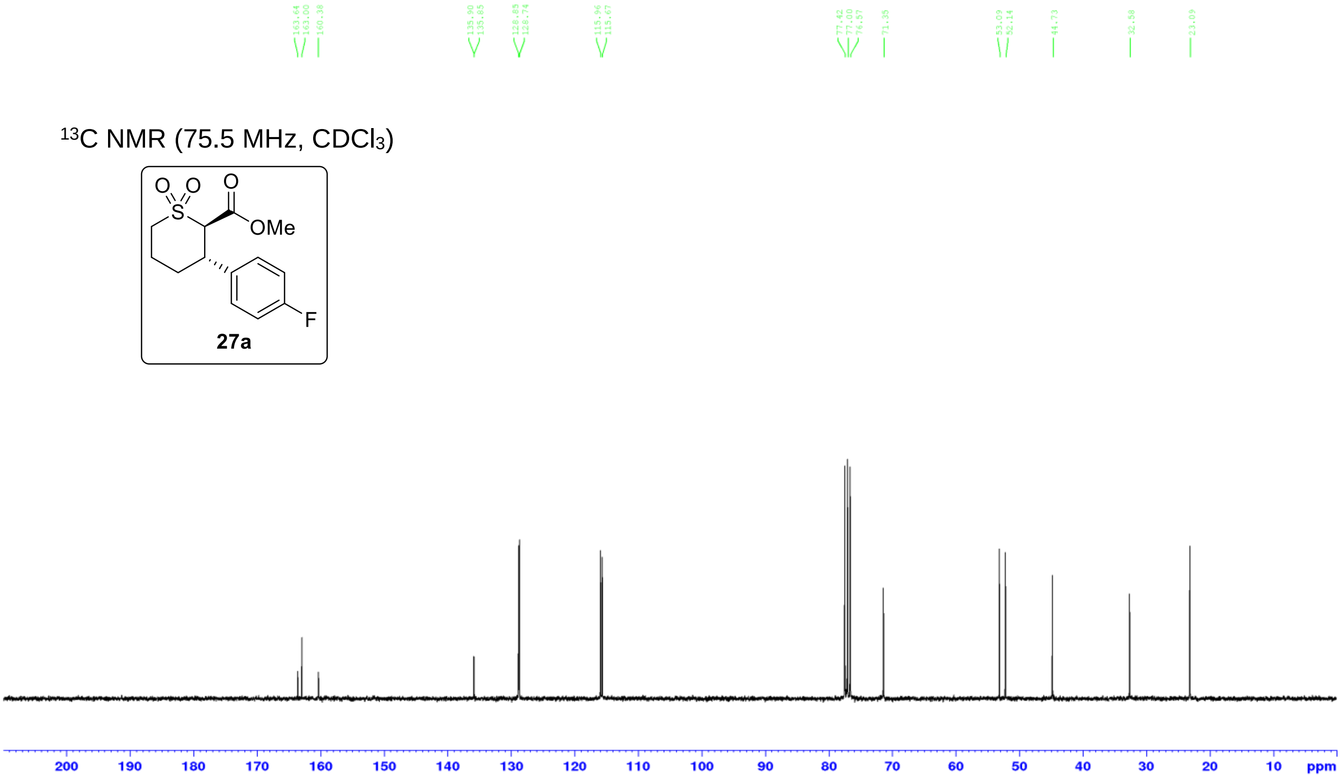

^{19}F NMR (282.4 MHz, $CDCl_3$)

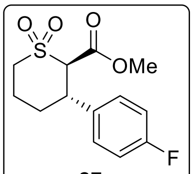

27a

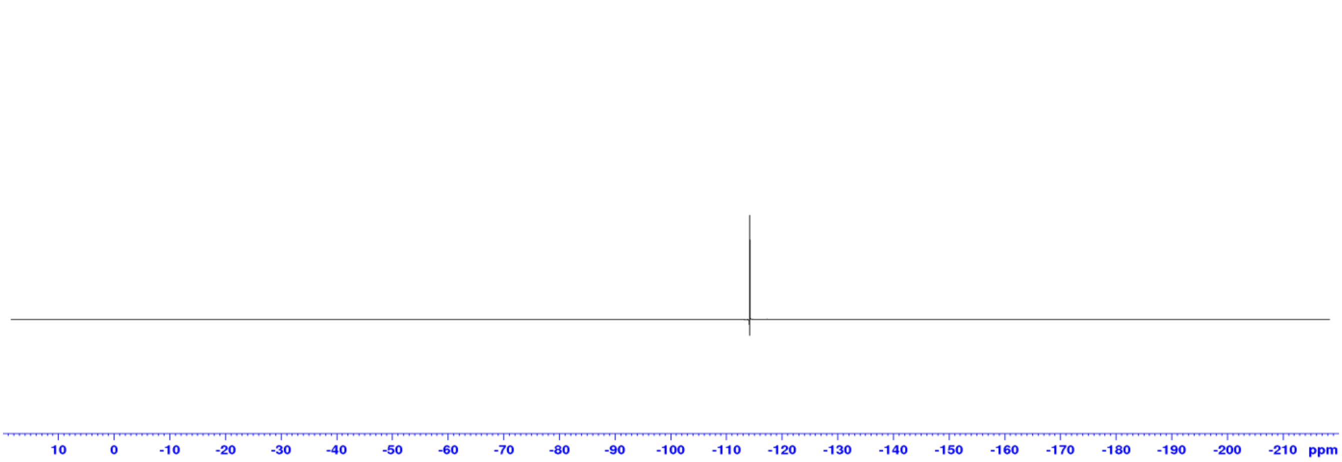

Methyl (2*S*,3*S*)-3-(4'-fluorophenyl)tetrahydro-2*H*-thiopyran-2-carboxylate 1,1-dioxide (27b)

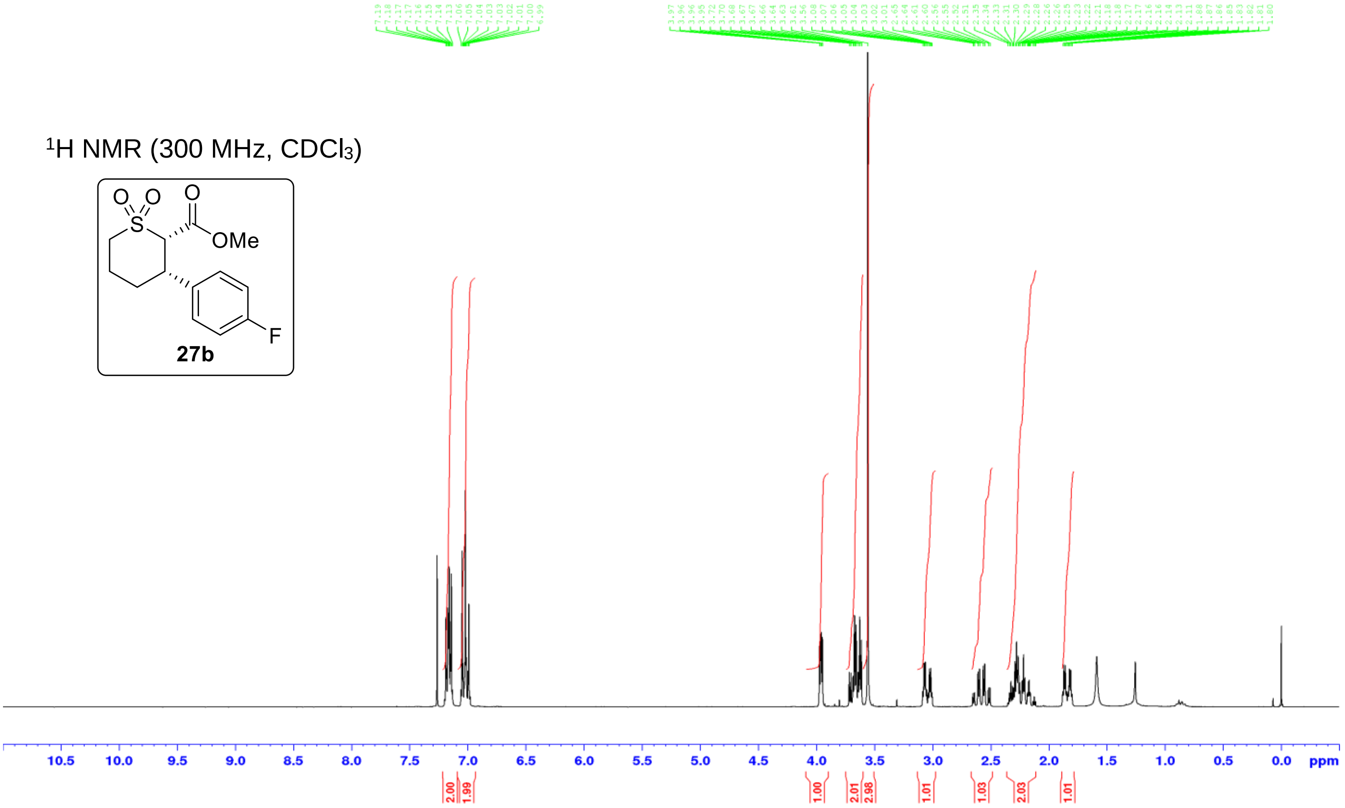

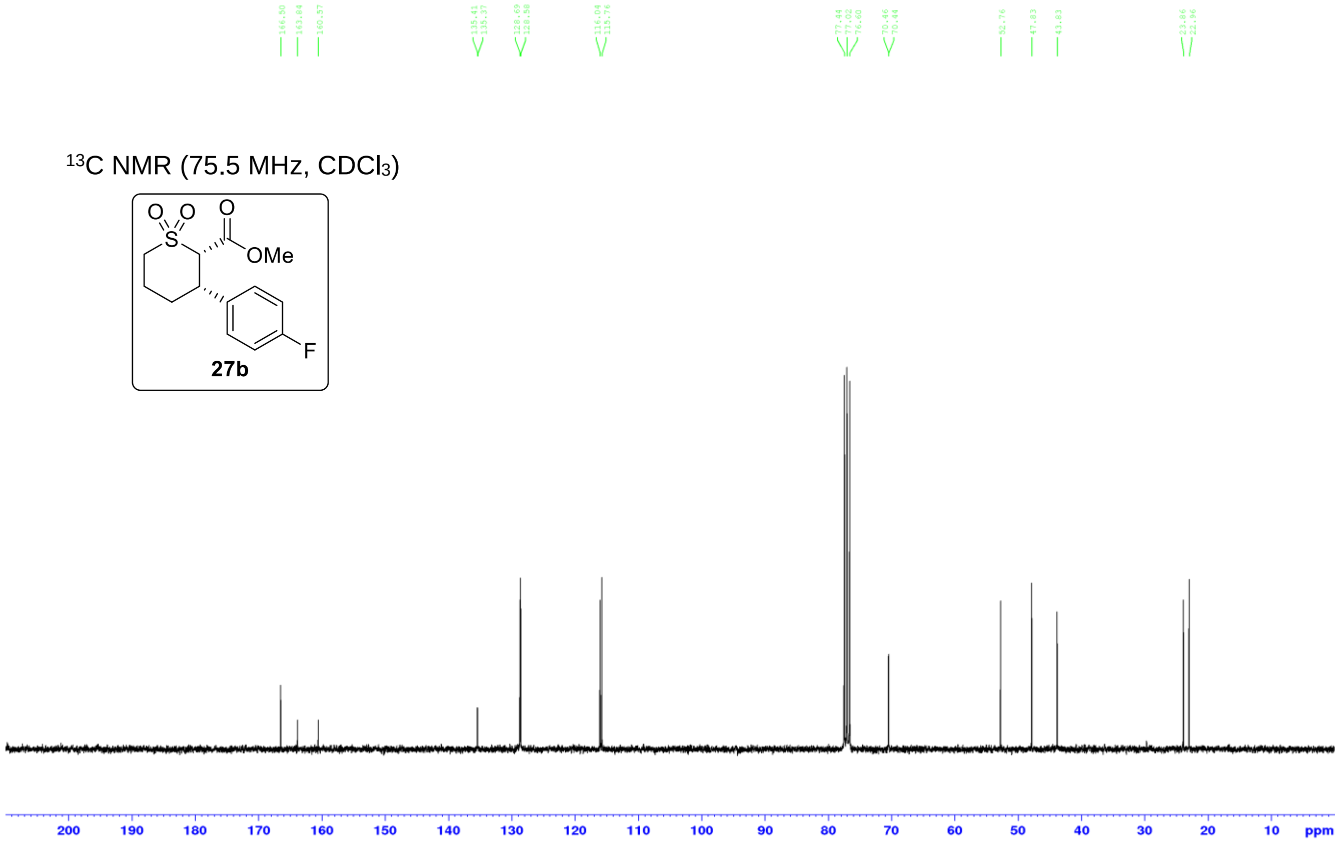

^{19}F NMR (282.4 MHz, $CDCl_3$)

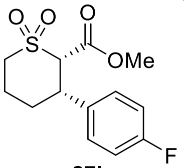

27b

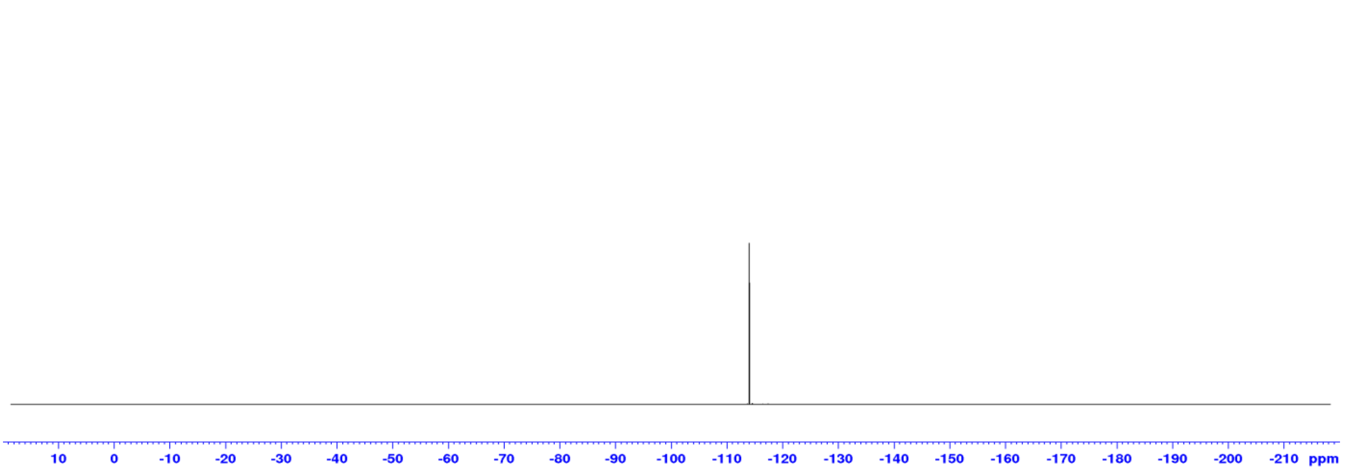

Methyl (2*R,3*R**)-3-(4'-fluorobenzyl)tetrahydrothiophene-2-carboxylate 1,1-dioxide (S27a)**

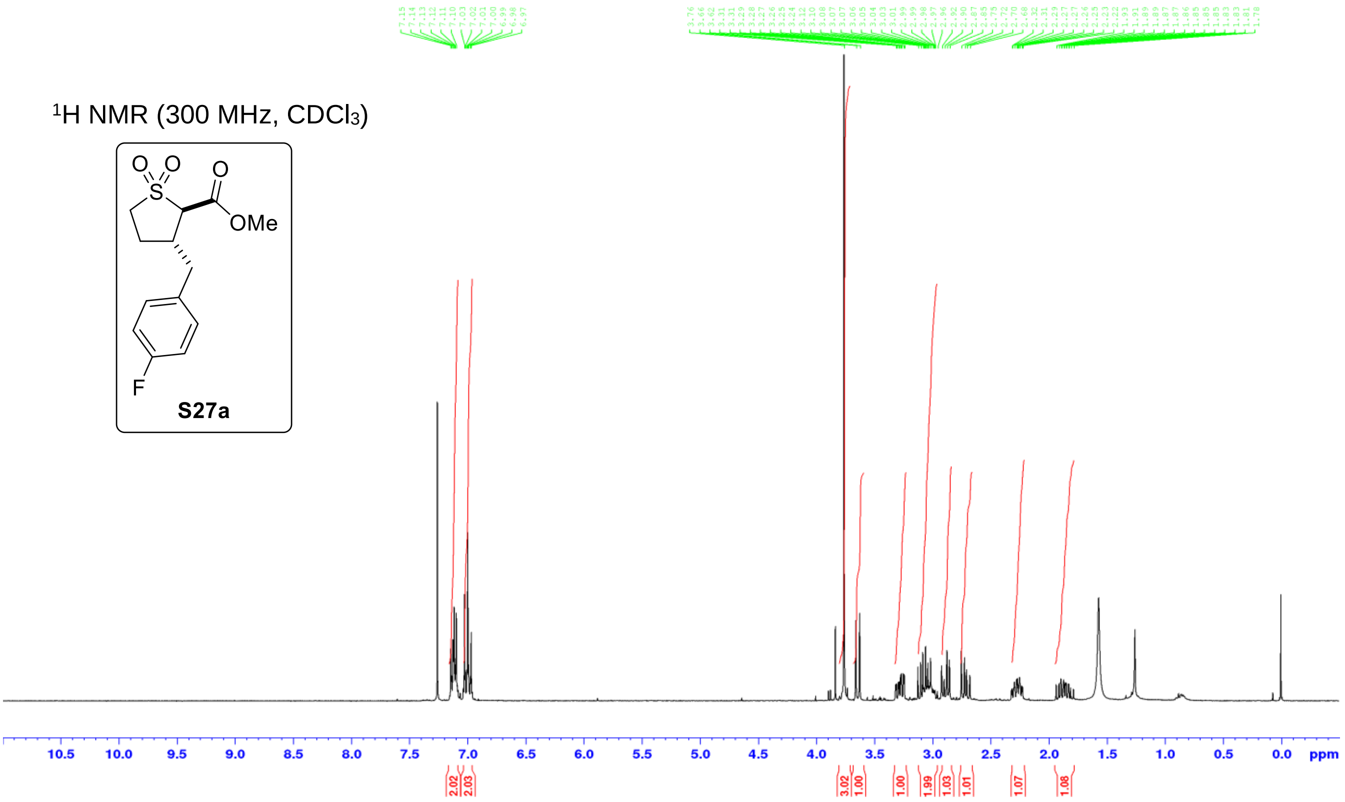

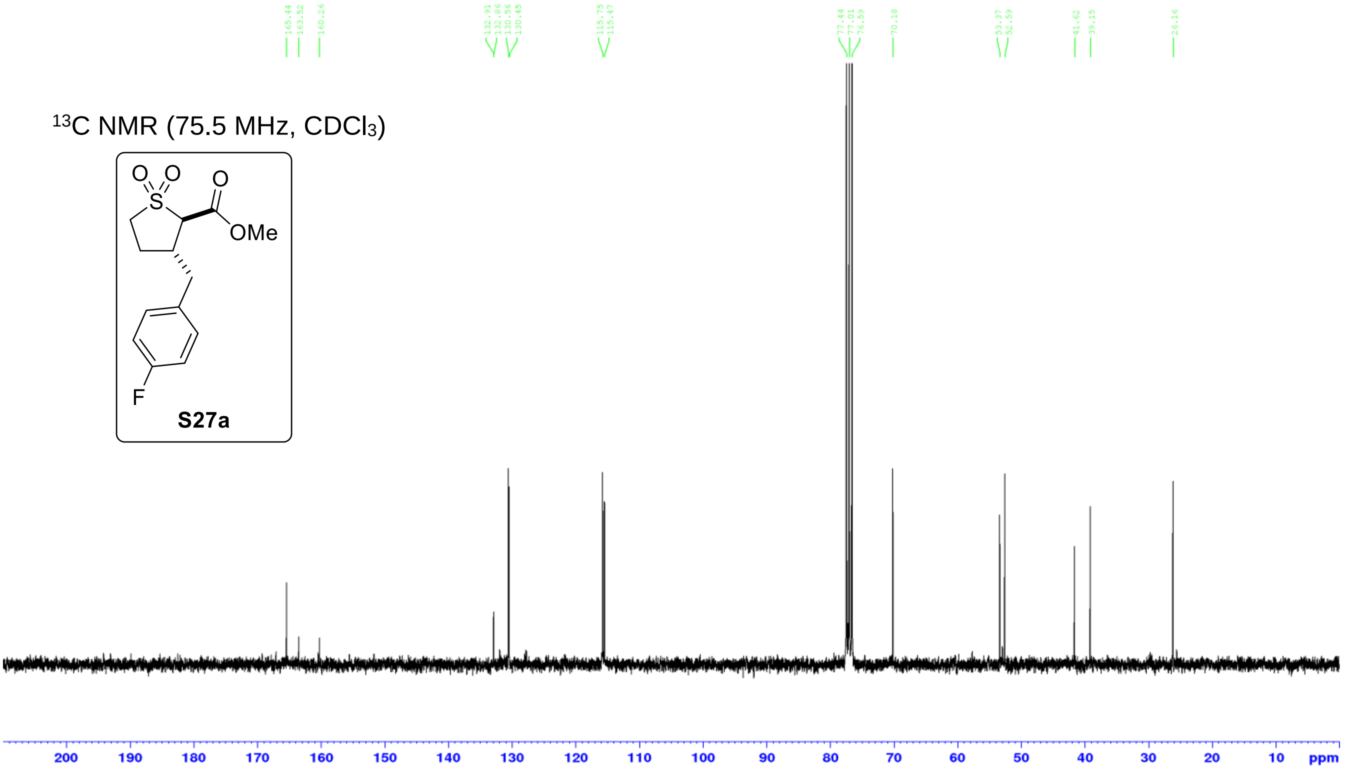

^{19}F NMR (282.4 MHz, $CDCl_3$)

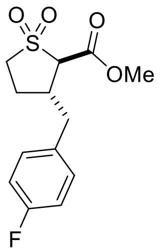

S27a

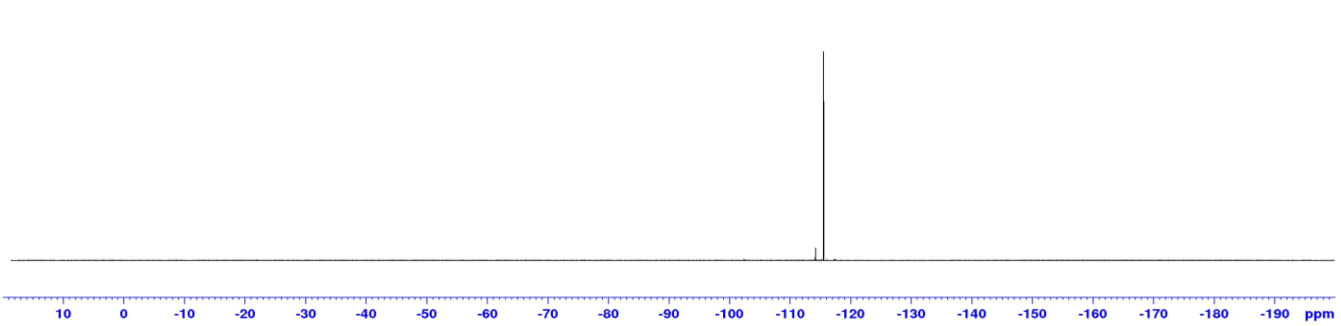

Methyl 2-((4'-(4''-fluorophenyl)but-3'-en-1'-yl)sulfonyl)acetate (S28)

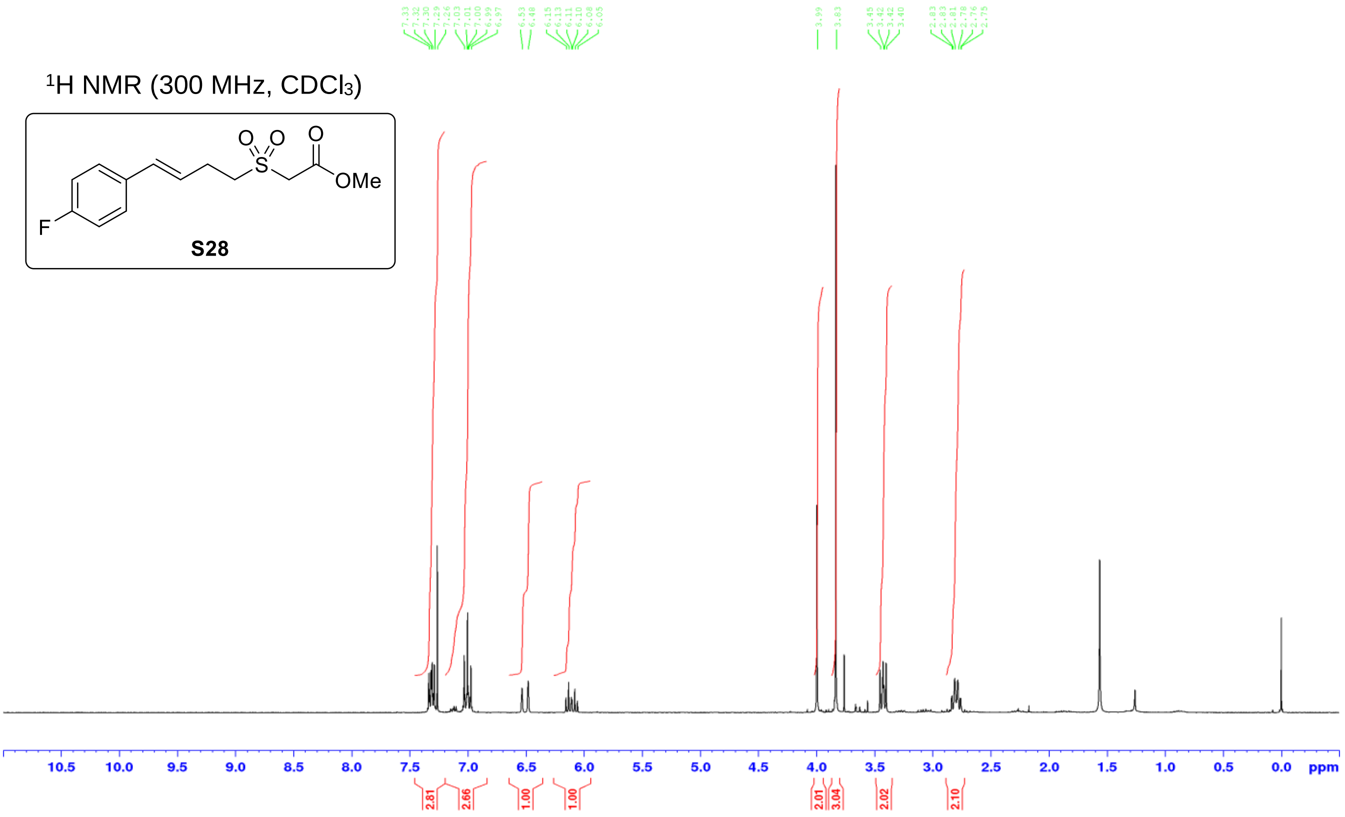

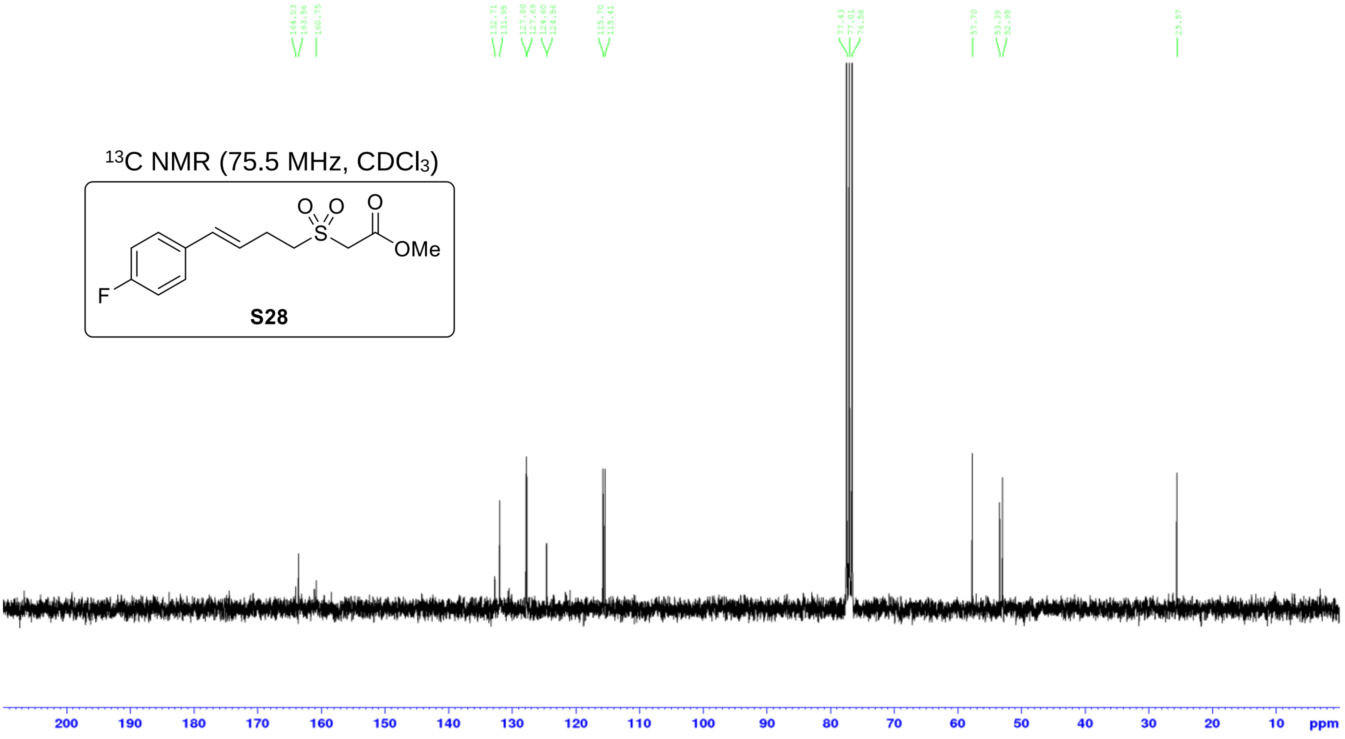

^{19}F NMR (282.4 MHz, $CDCl_3$)

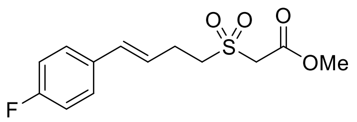

S28

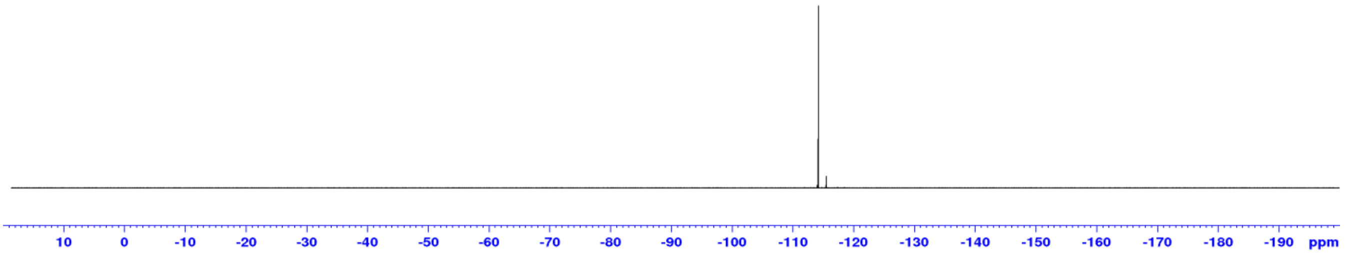

((2*R,3*S**)-1,1-Dioxido-3-phenyltetrahydro-2*H*-thiopyran-2-yl)(phenyl)methanone (28a)**

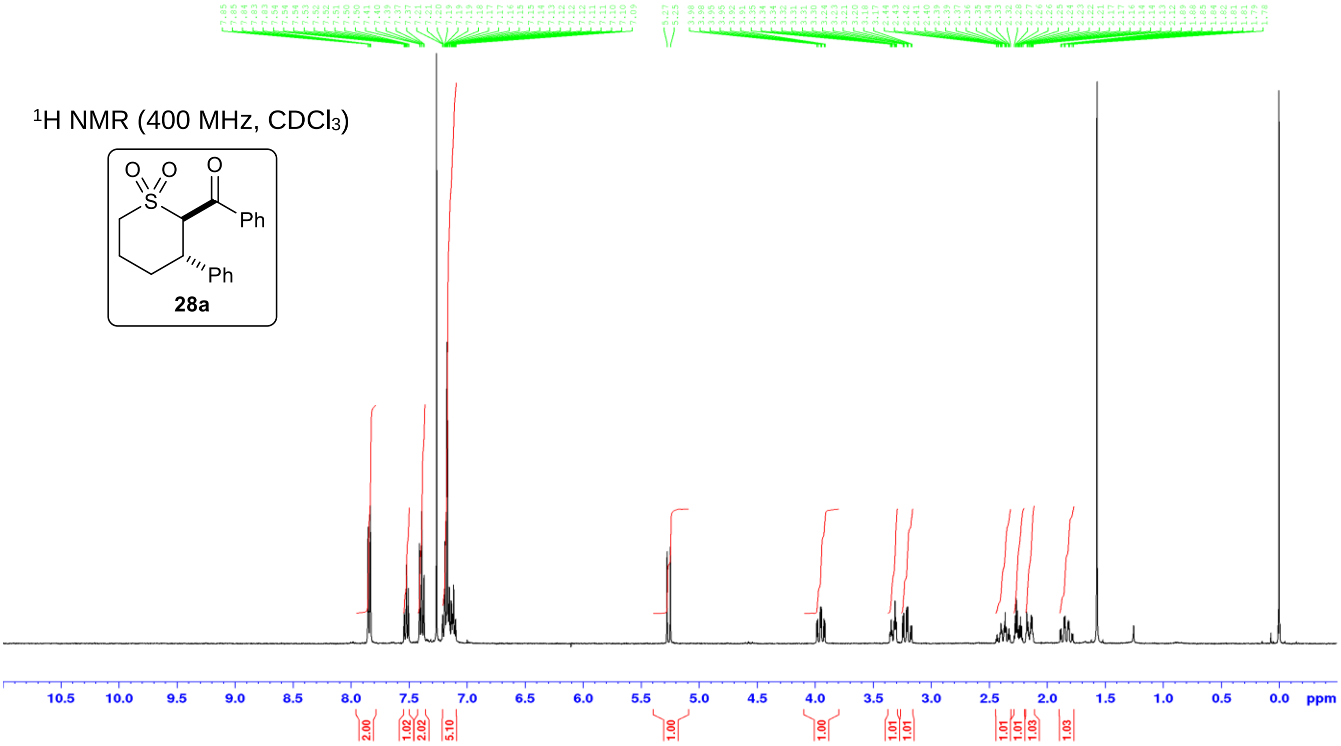

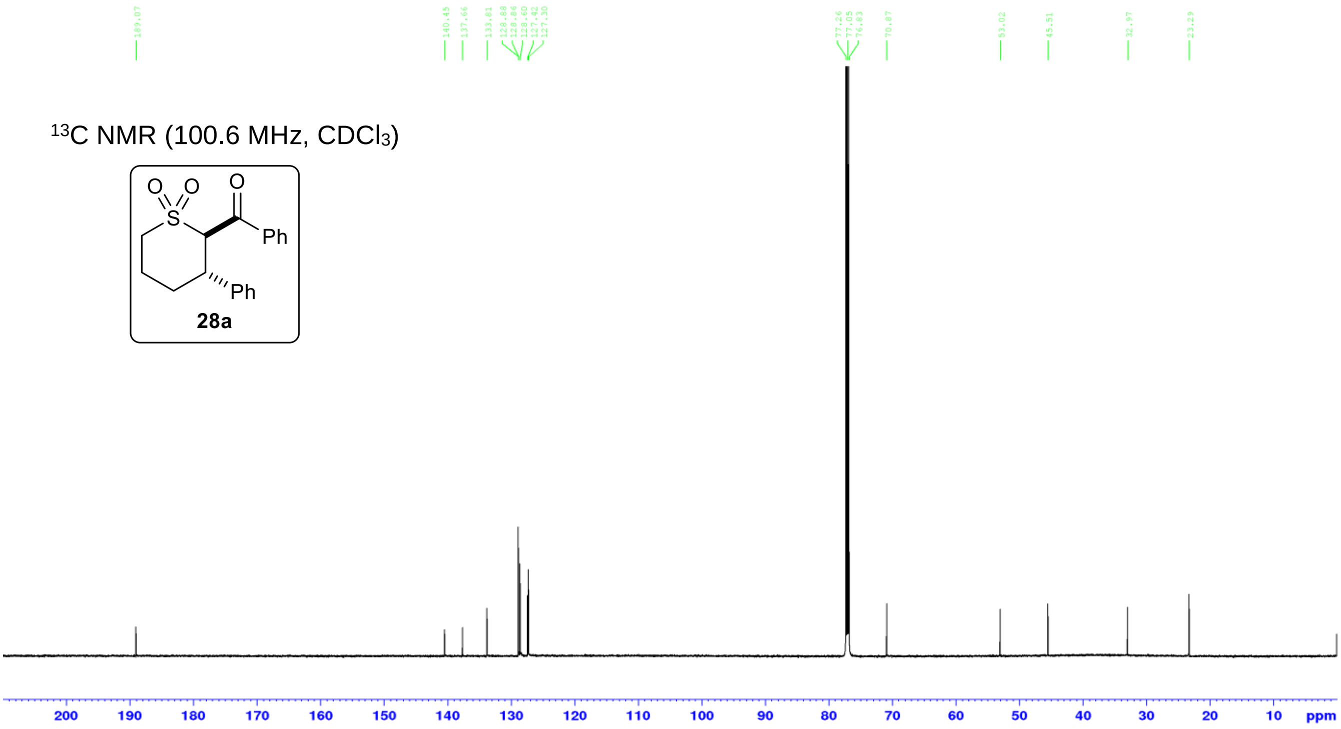

Benzyl (2*R*,3*R*)-3-octyltetrahydro-2*H*-thiopyran-2-carboxylate 1,1-dioxide (29a)

^{1}H NMR (300 MHz, $CDCl_3$)

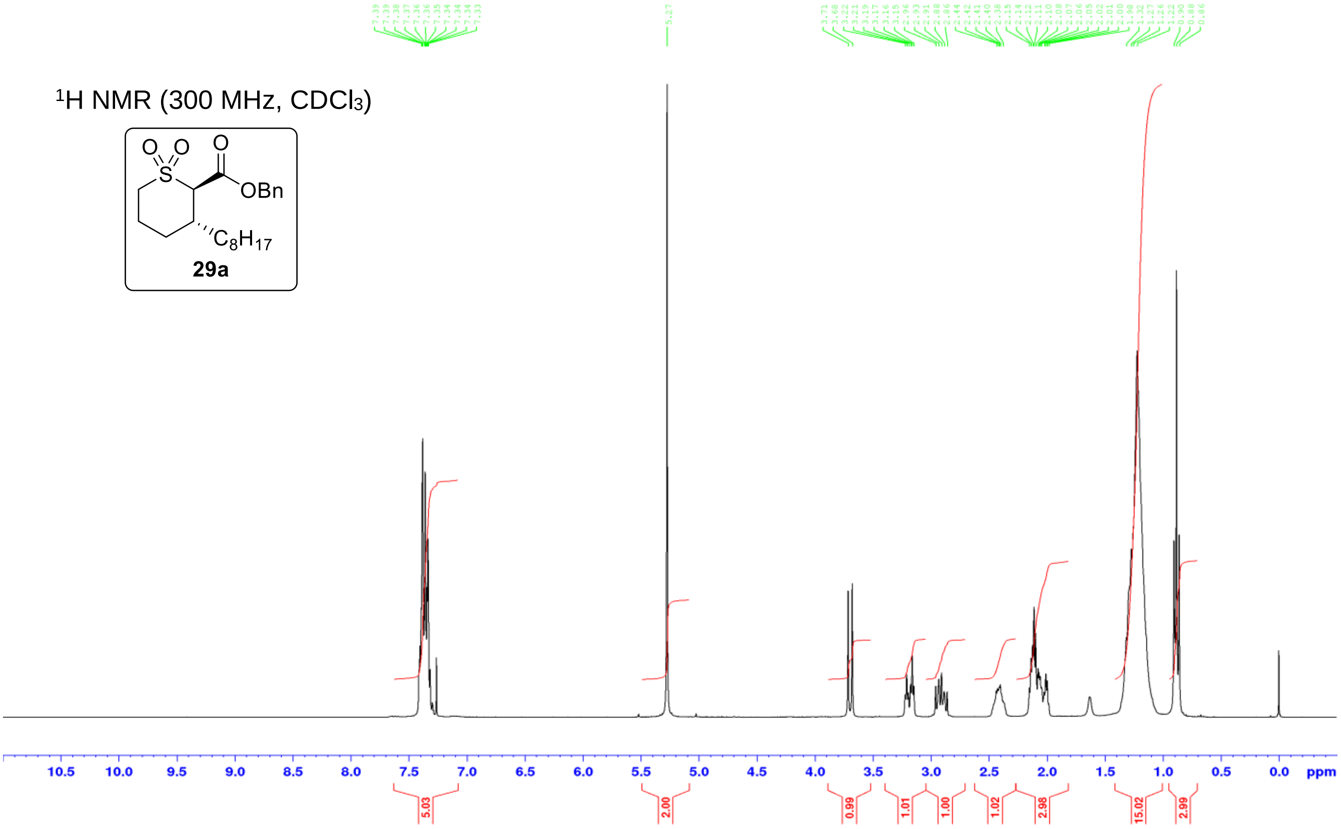

^{13}C NMR (75.5 MHz, $CDCl_3$)

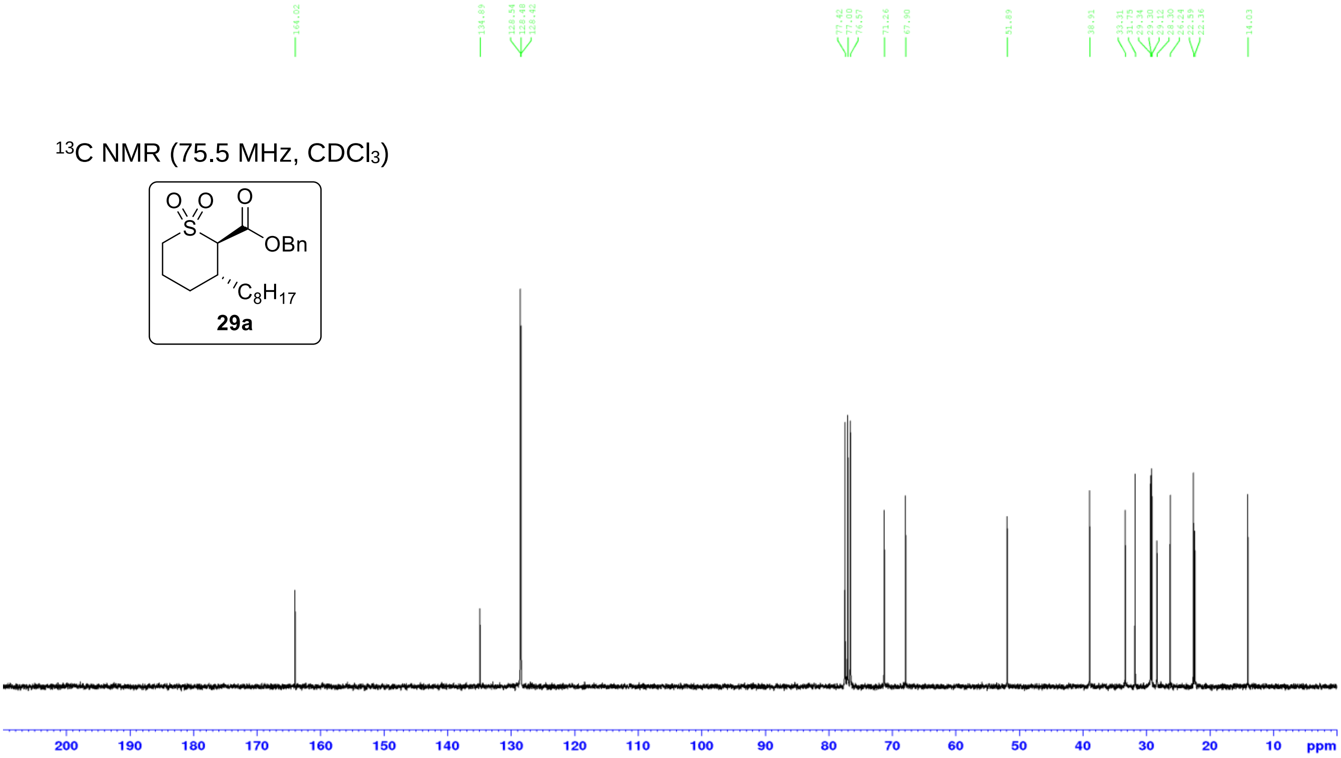

Benzyl (2*R*,3*S*)-3-octyltetrahydro-2H-thiopyran-2-carboxylate 1,1-dioxide (29b)

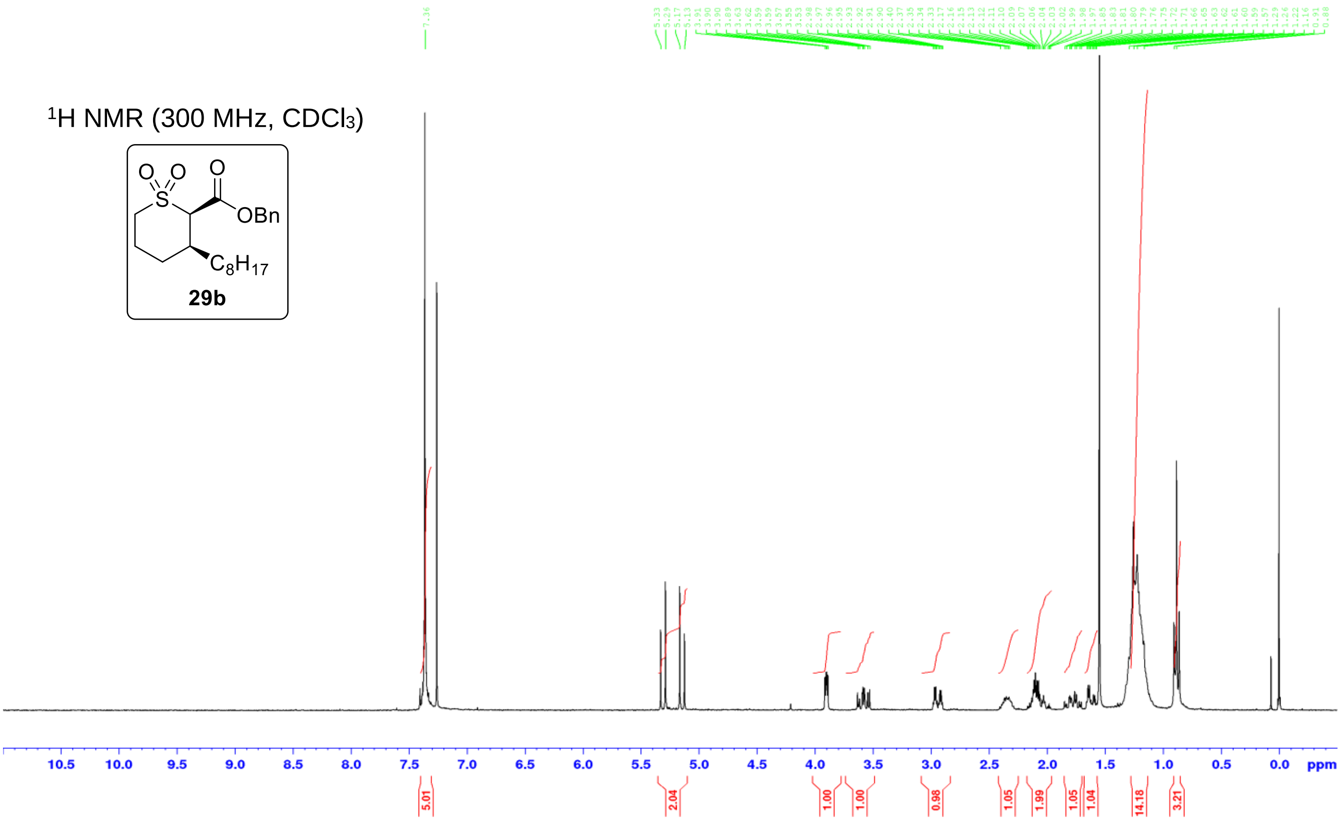

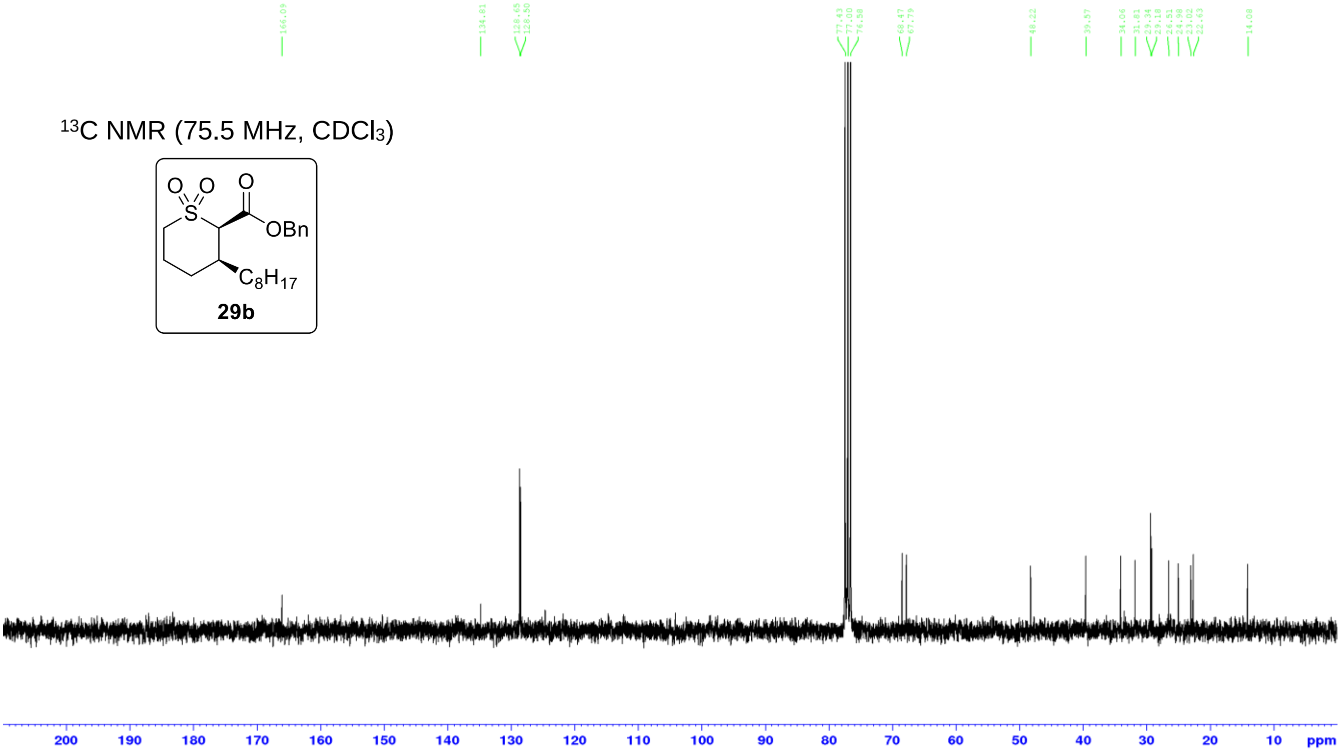

Benzyl (2*R,3*R**)-3-nonyltetrahydrothiophene-2-carboxylate 1,1-dioxide (S30a)**

^{1}H NMR (300 MHz, CDCl$_3$)

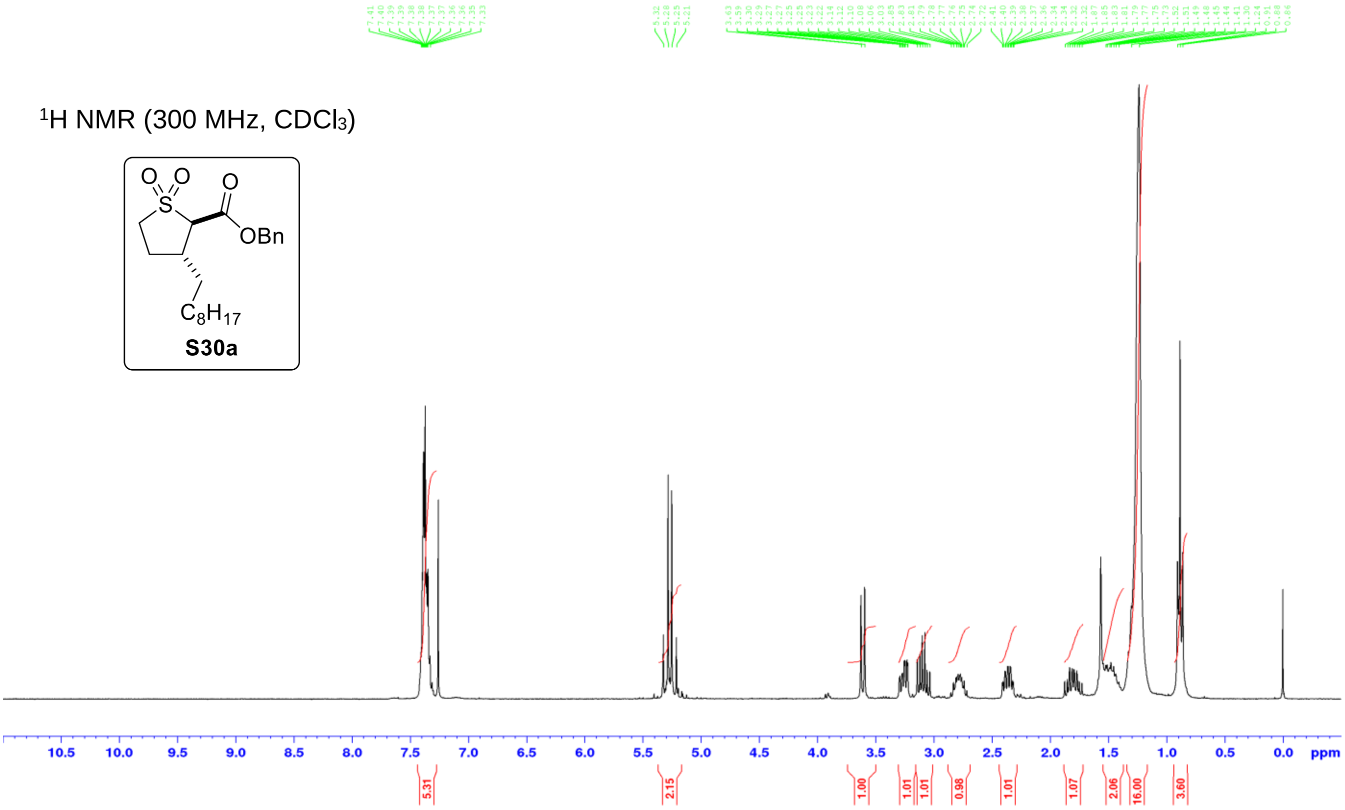

^{13}C NMR (75.5 MHz, CDCl$_3$)

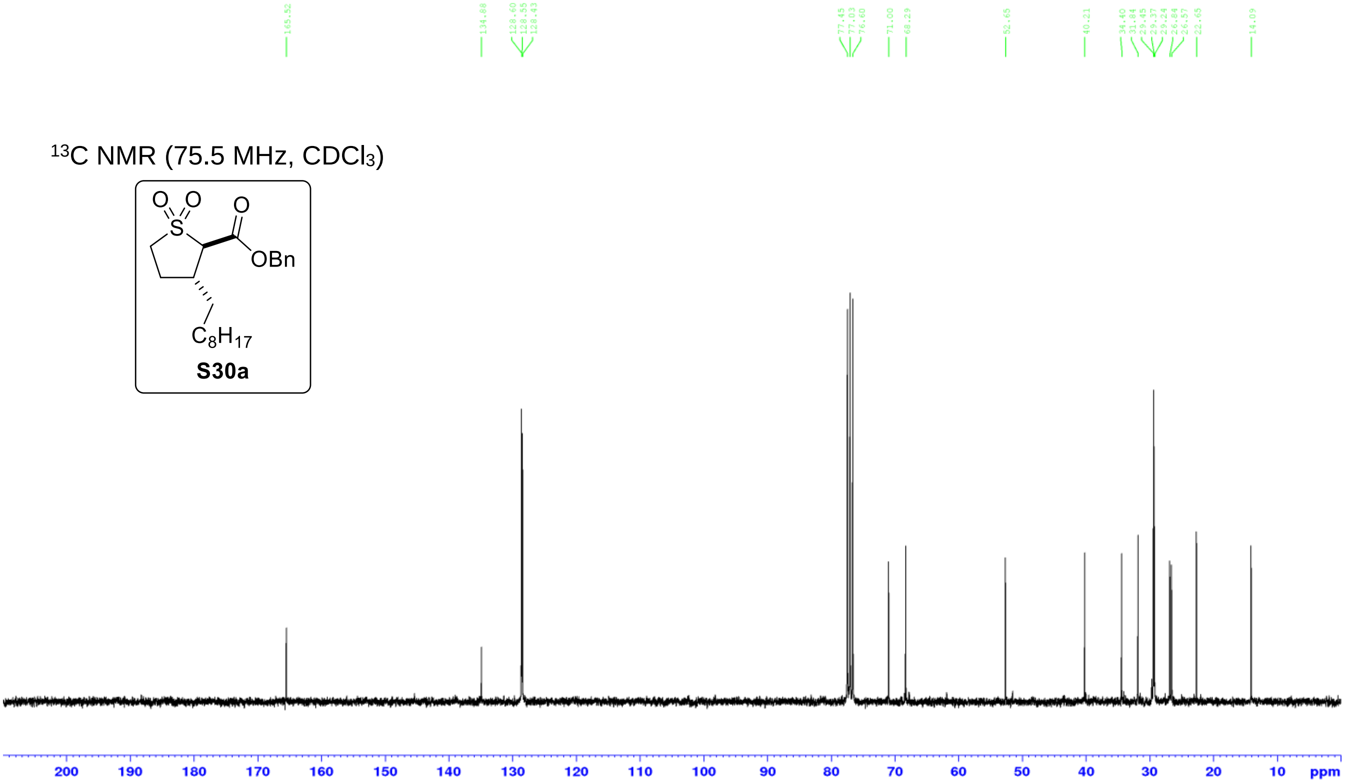

Methyl (1*R*,4a*S*,8a*R*)-octahydro-1*H*-isothiochromene-1-carboxylate 2,2-dioxide (30a)

^{1}H NMR (300 MHz, $CDCl_3$)

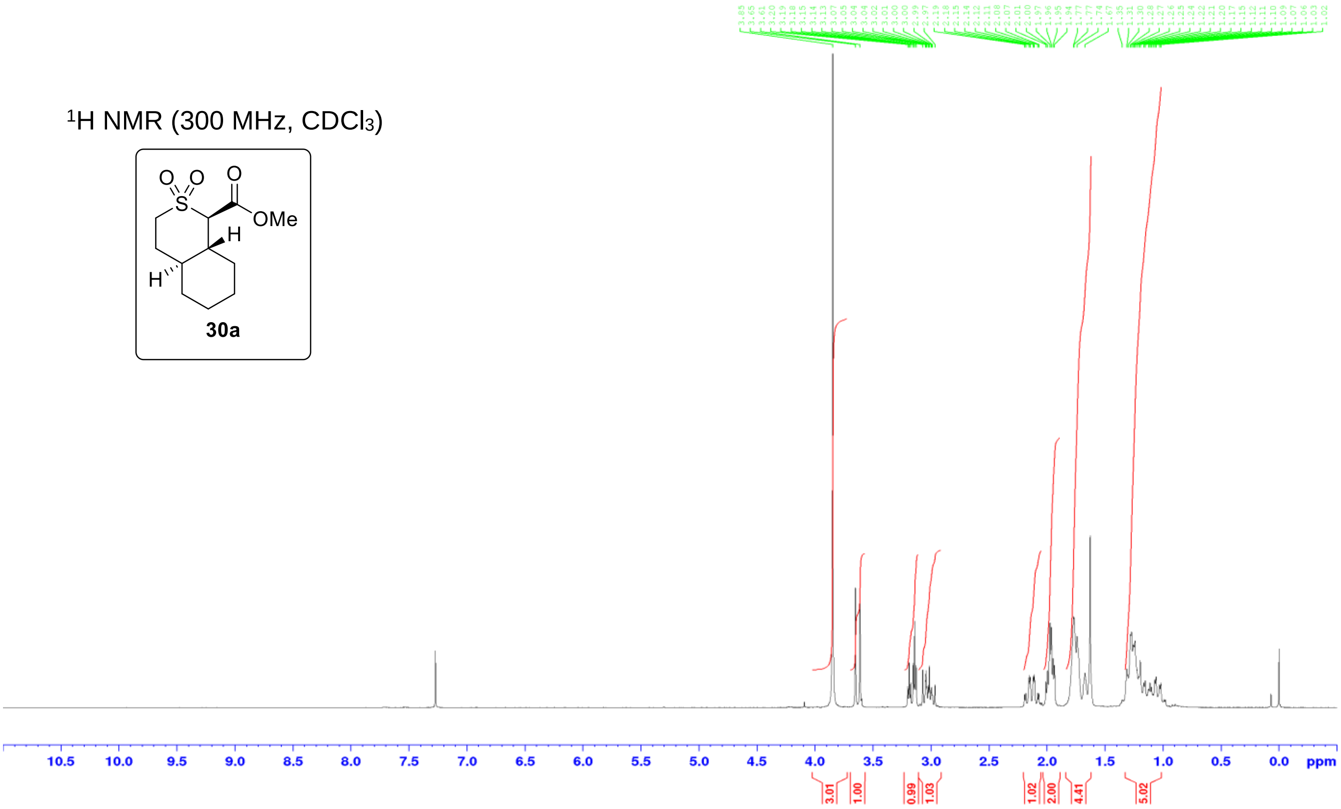

^{13}C NMR (75.5 MHz, $CDCl_3$)

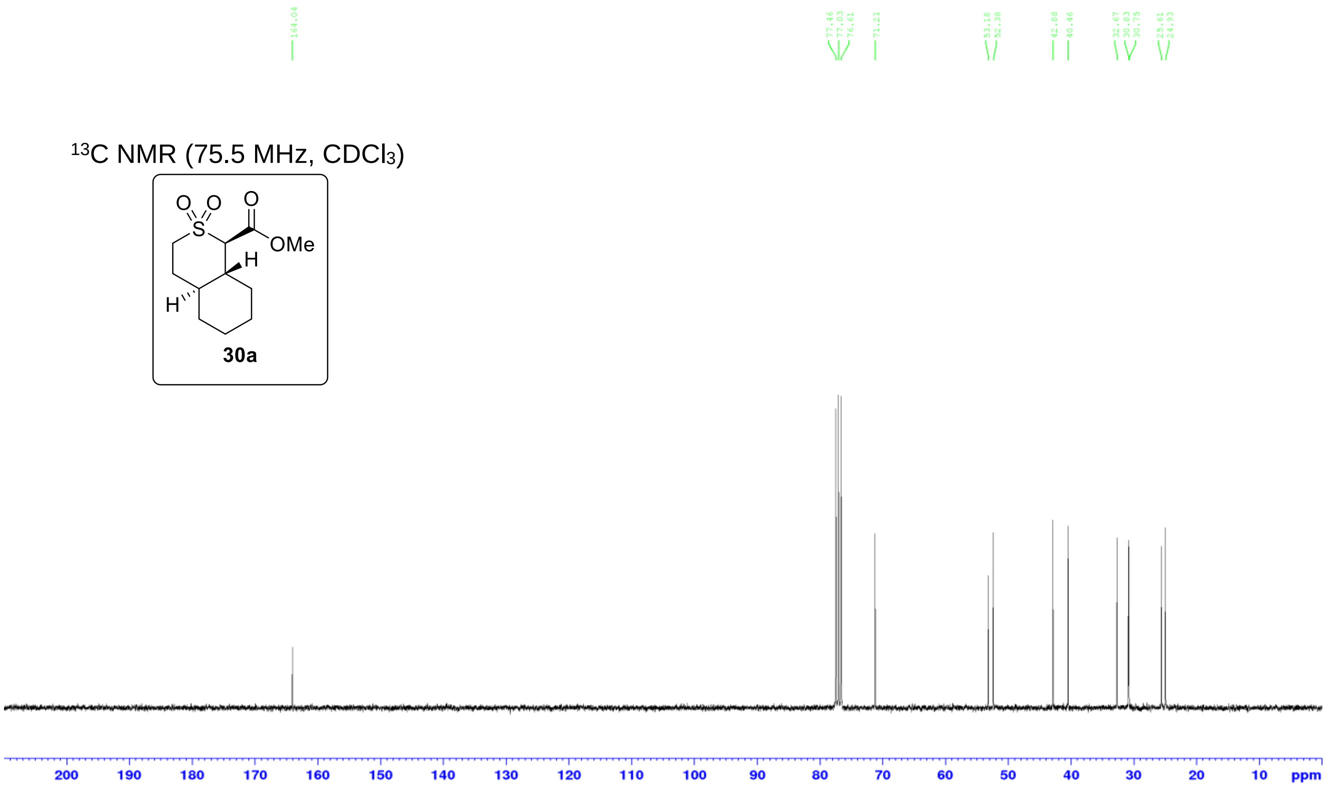

Methyl (1*R,4a*R**,8a*S**)-octahydro-1H-isothiochromene-1-carboxylate 2,2-dioxide (30b)**

^{1}H NMR (300 MHz, $CDCl_3$)

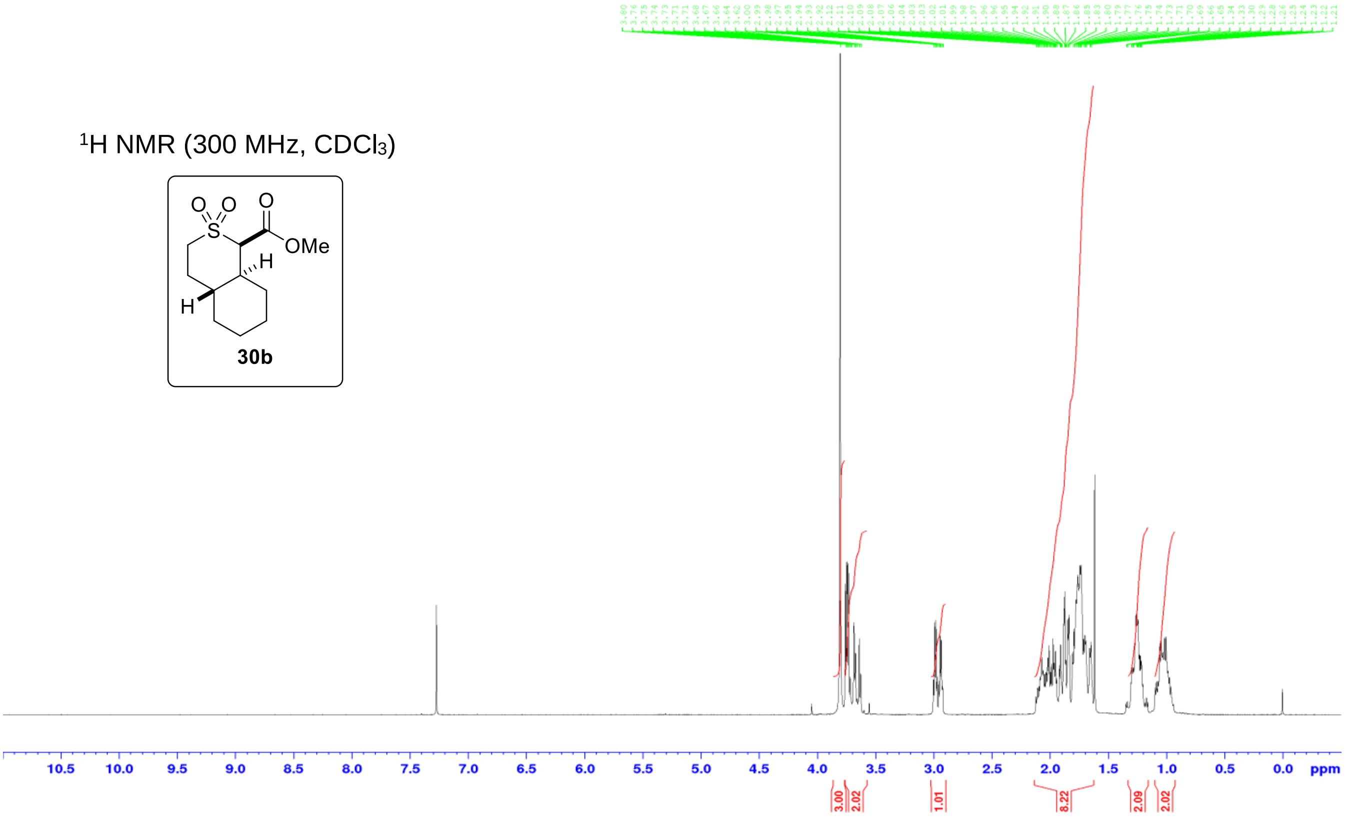

^{13}C NMR (75.5 MHz, $CDCl_3$)

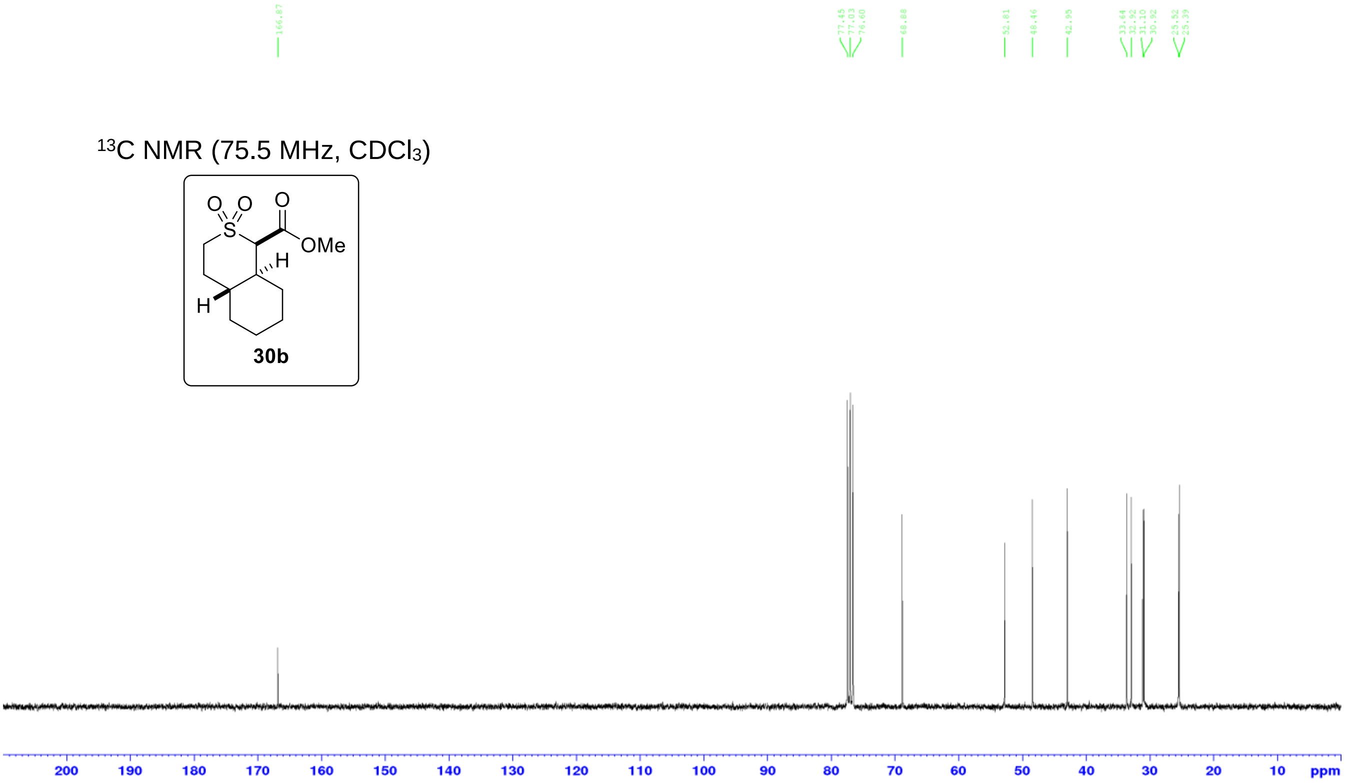

***N*,*N*-Dibenzyl-2-cyano-2-diazoacetamide (31)**

^{1}H NMR (400 MHz, $CDCl_3$)

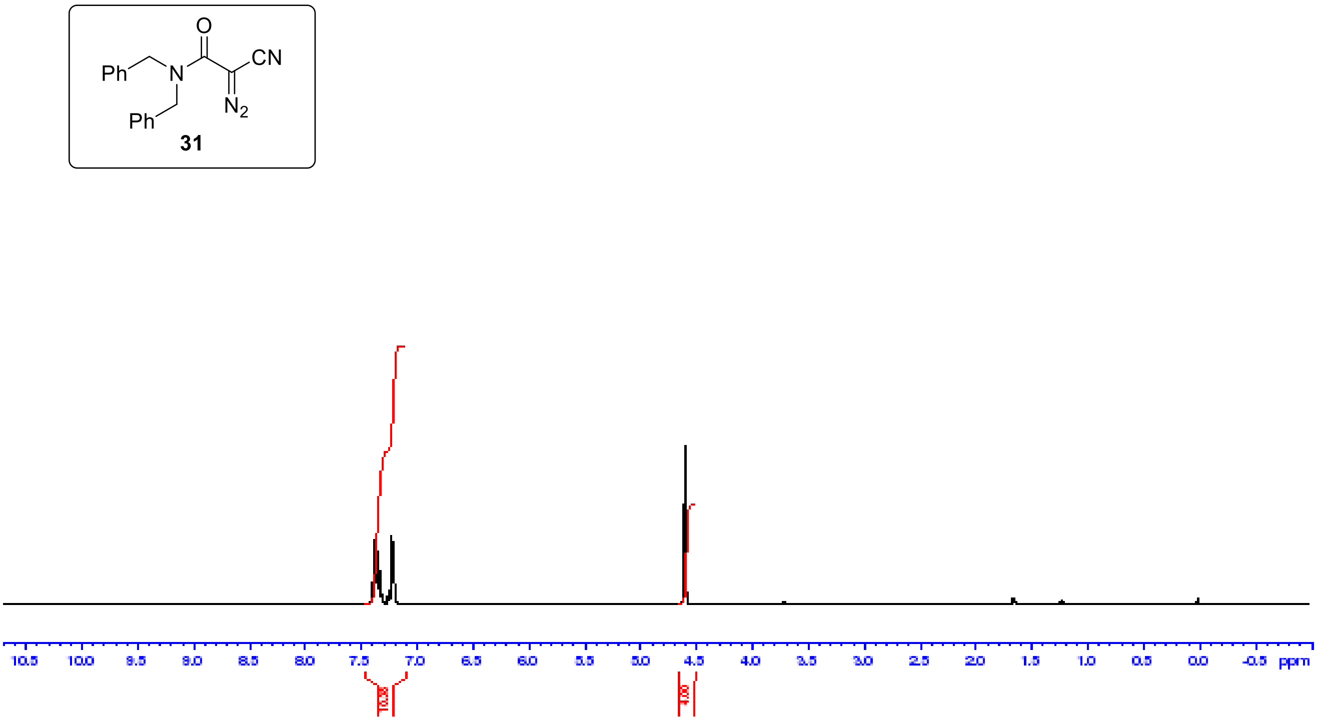

^{13}C NMR (100.6 MHz, $CDCl_3$)

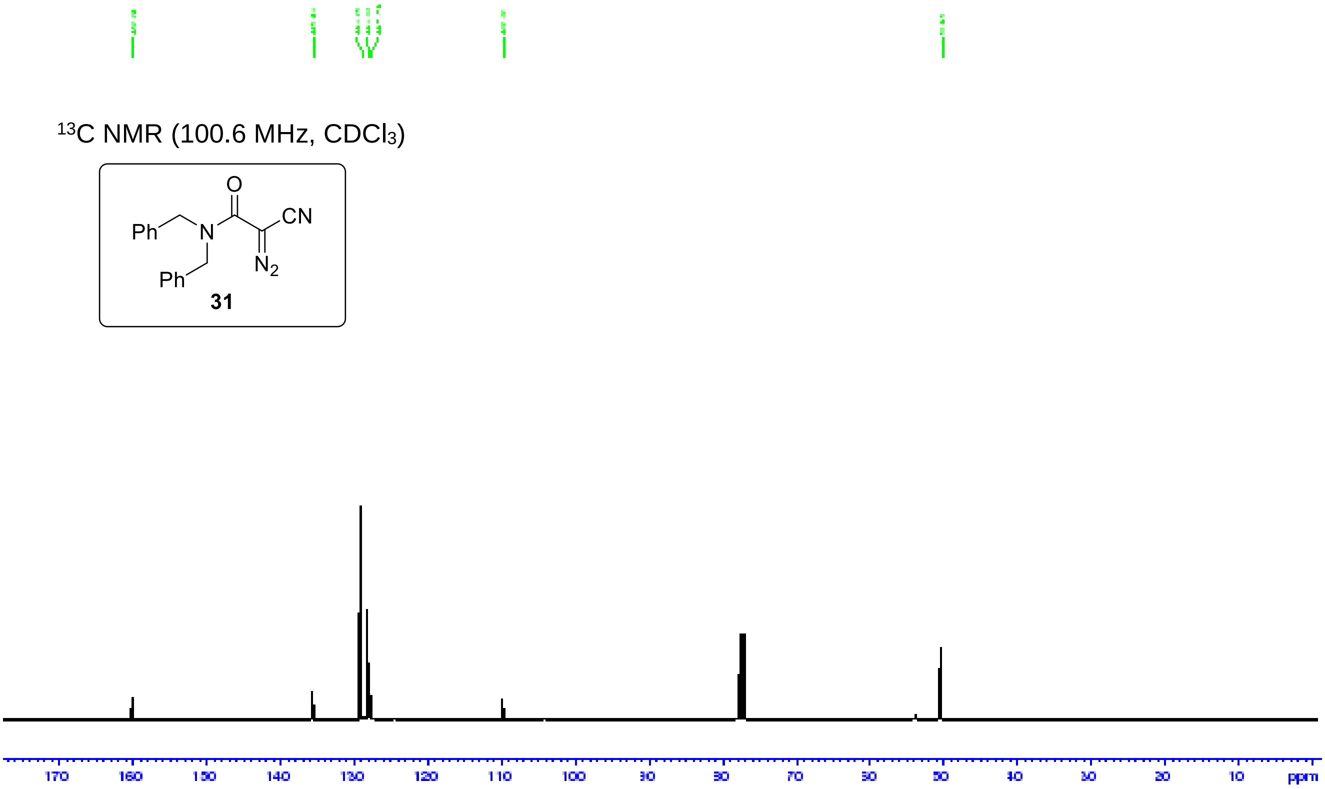

N-(*tert*-Butyl)-2-cyano-2-diazo-*N*-(4'-fluorobenzyl)acetamide (33)

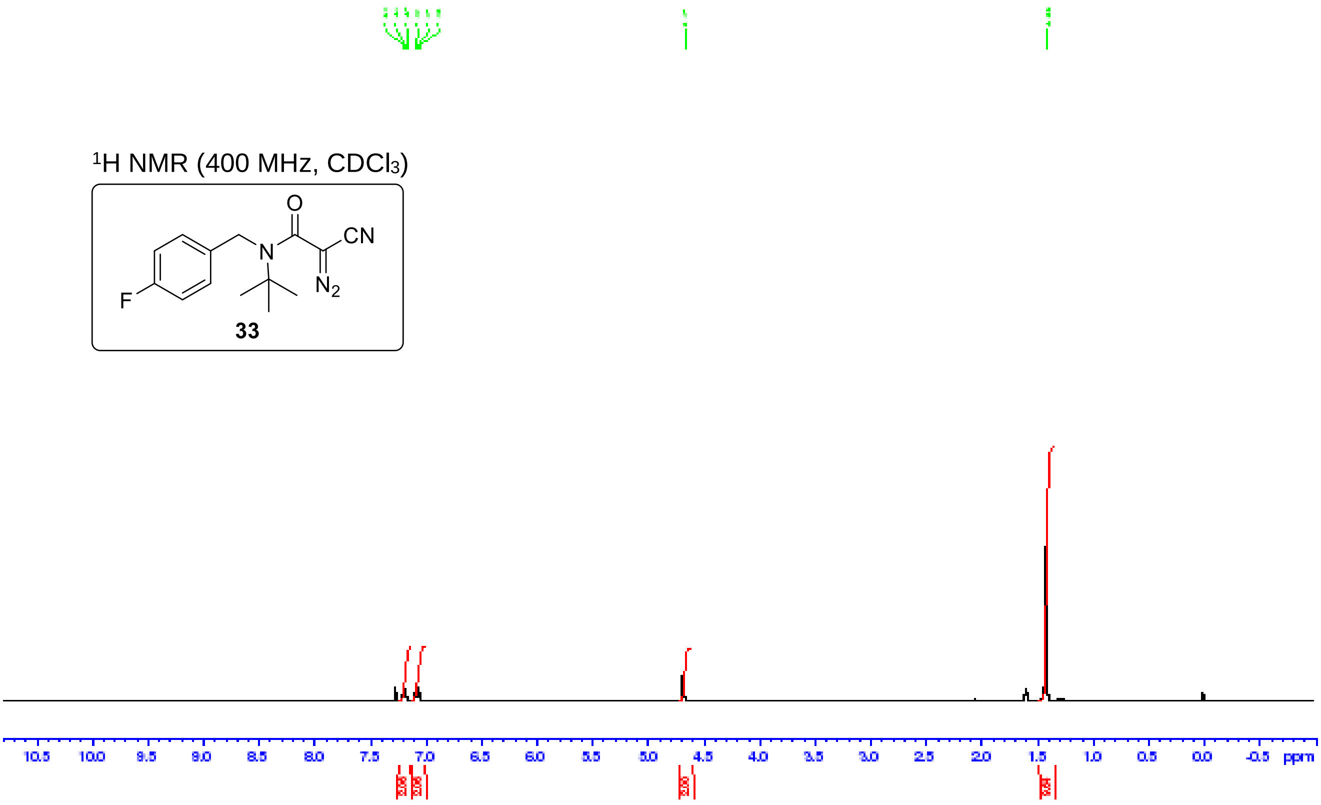

^{1}H NMR (400 MHz, $CDCl_3$)

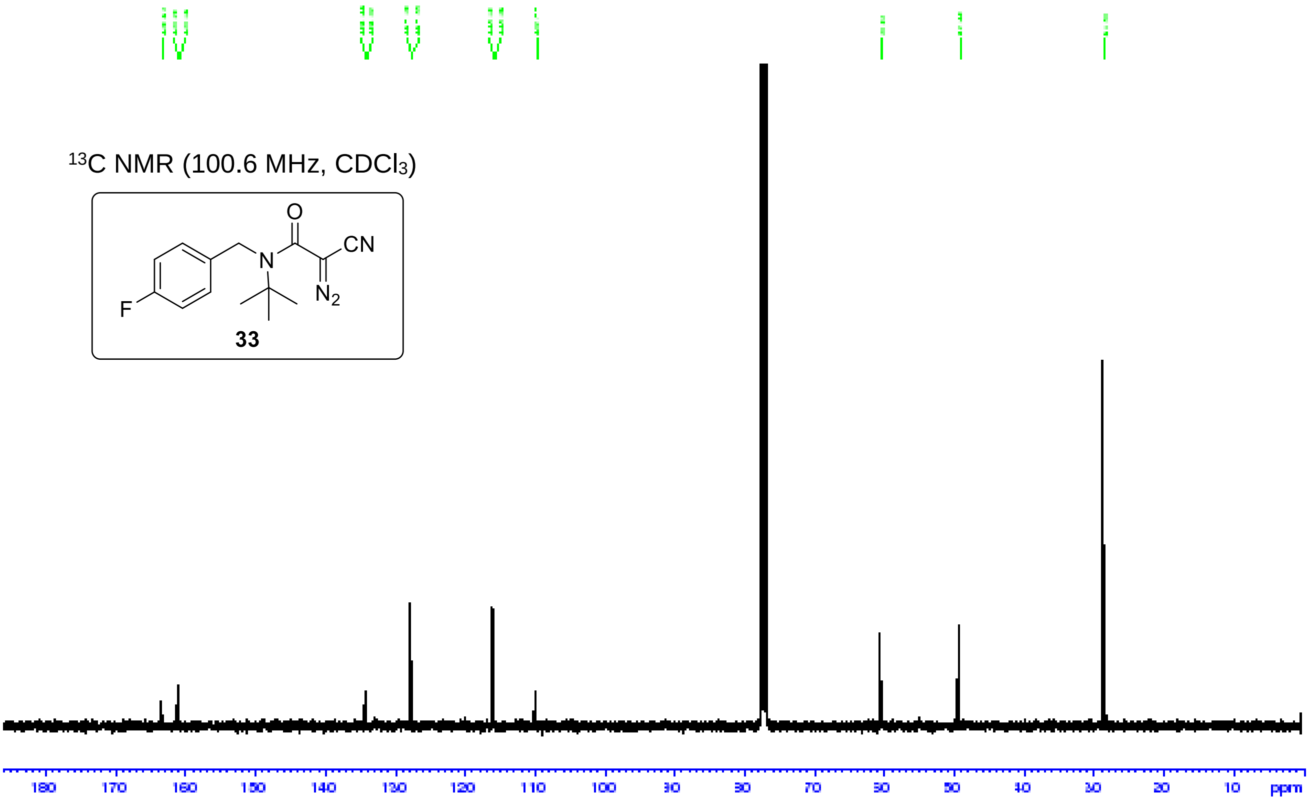

^{13}C NMR (100.6 MHz, $CDCl_3$)

^{19}F NMR (376.5 MHz, $CDCl_3$)

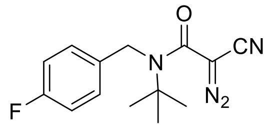

33

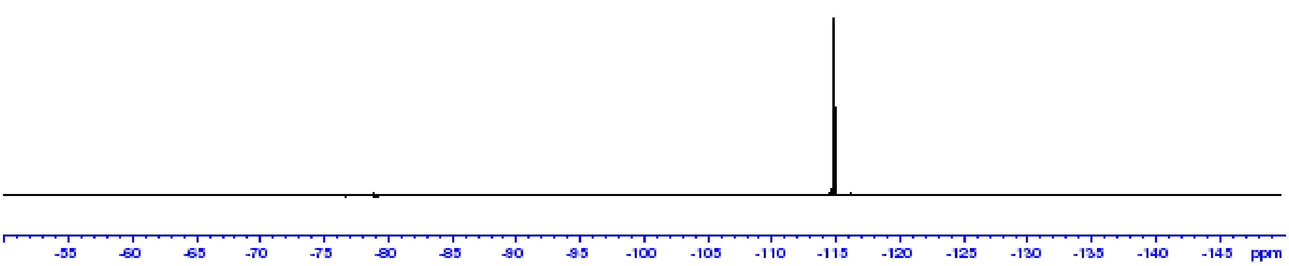

N-(*tert*-Butyl)-2-cyano-2-diazo-*N*-(4'-bromobenzyl)acetamide (34)

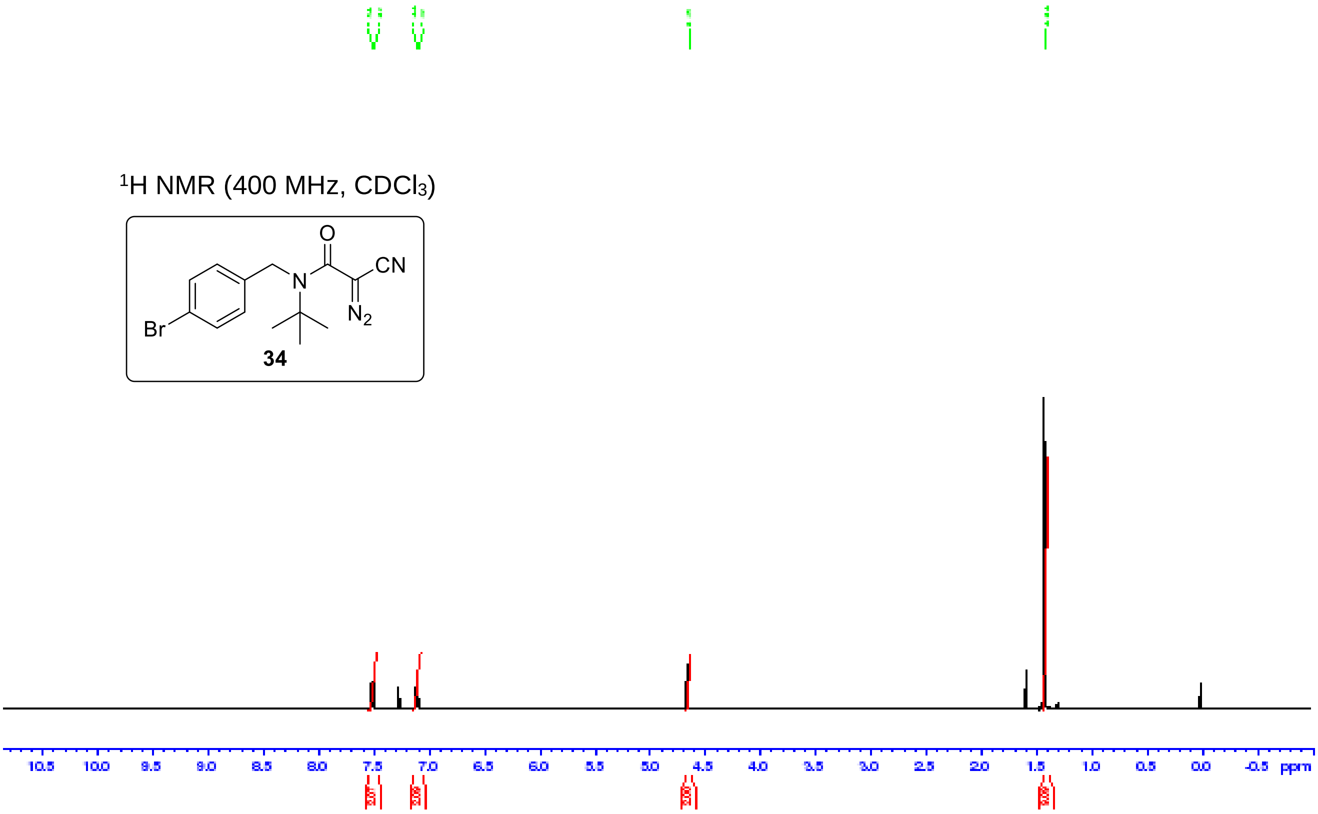

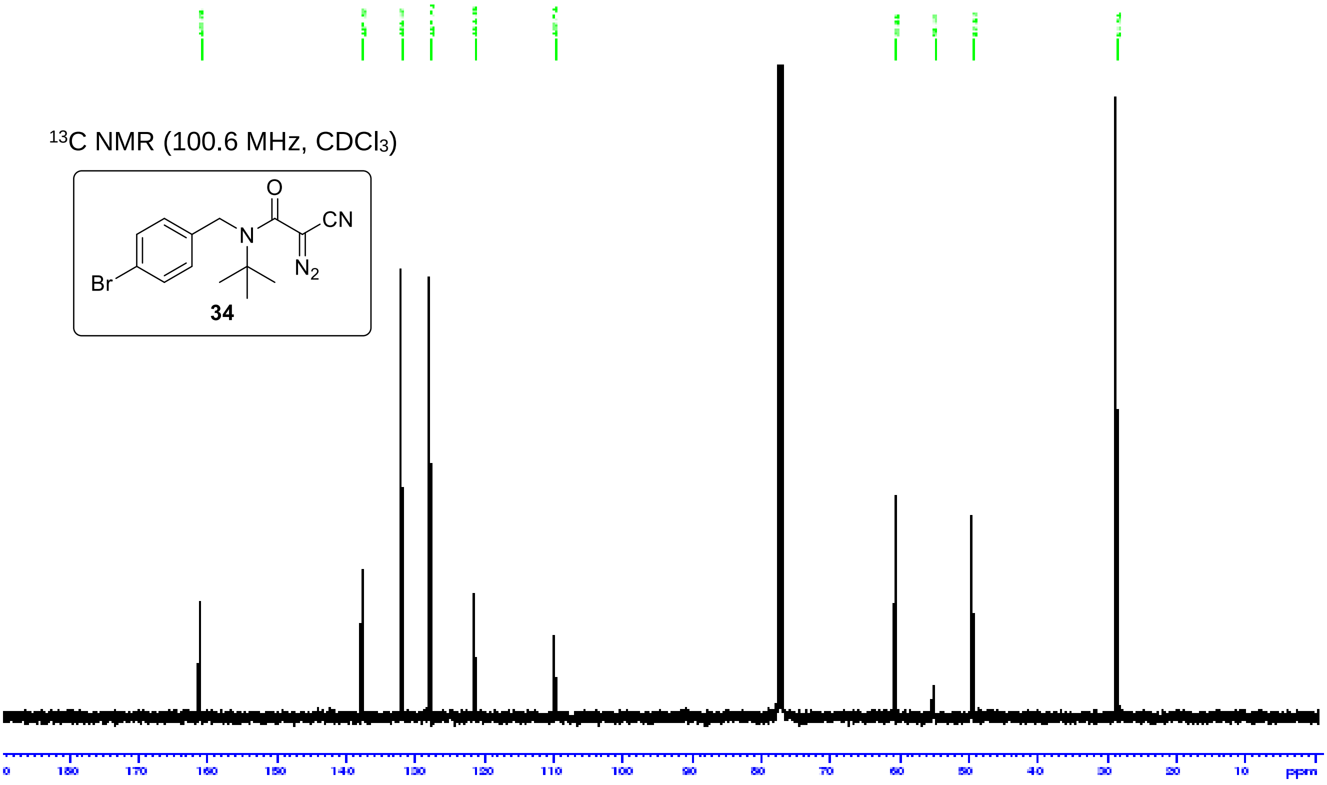

N-(*tert*-Butyl)-2-cyano-2-diazo-*N*-(4'-chlorobenzyl)acetamide (35)

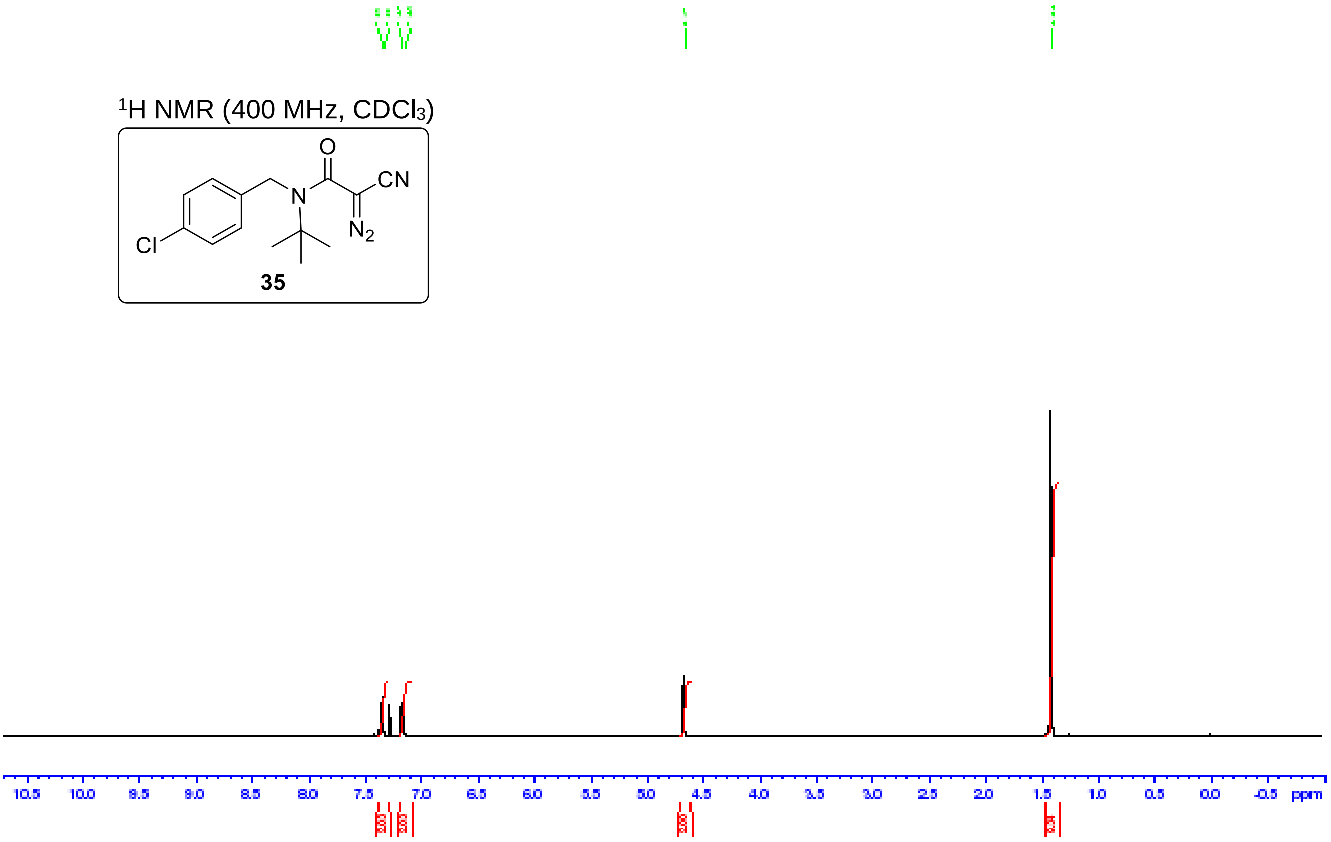

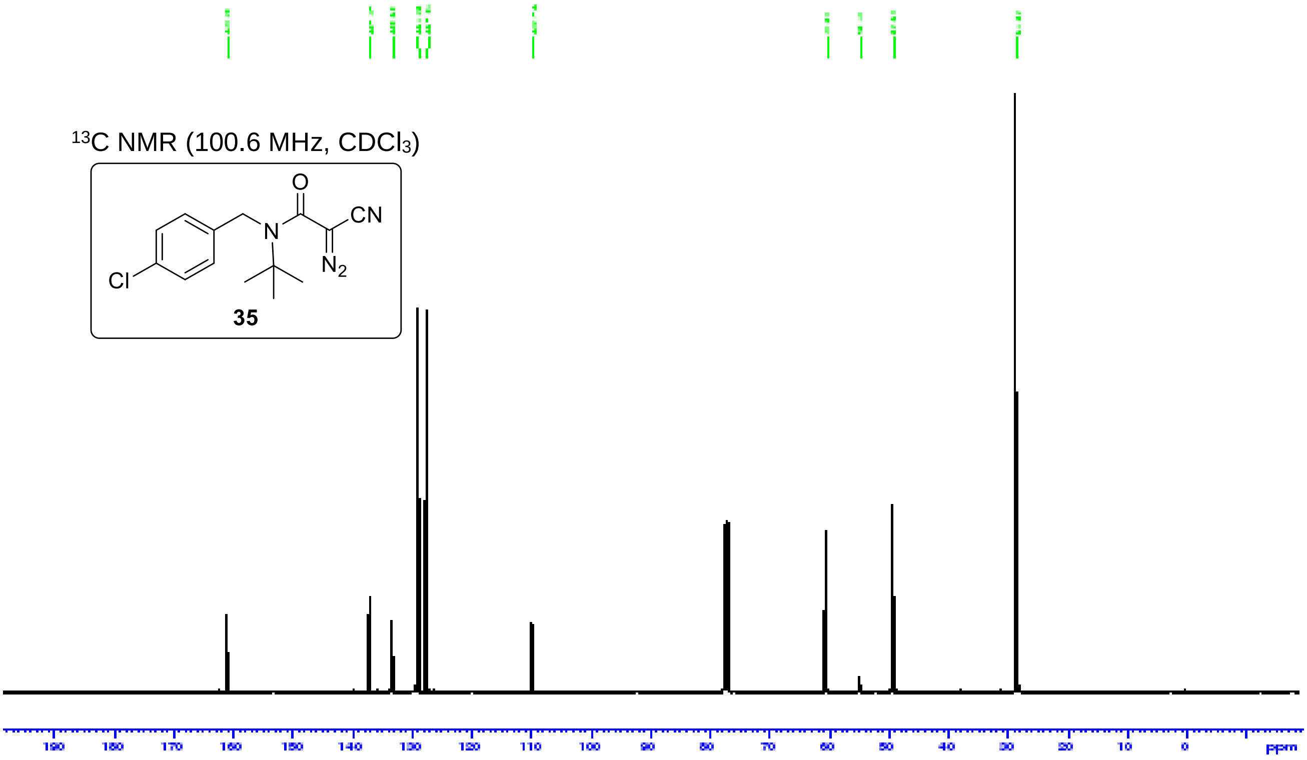

N-(*tert*-Butyl)-2-cyano-2-diazo-N-(2',6'-dichlorobenzyl)acetamide (36)

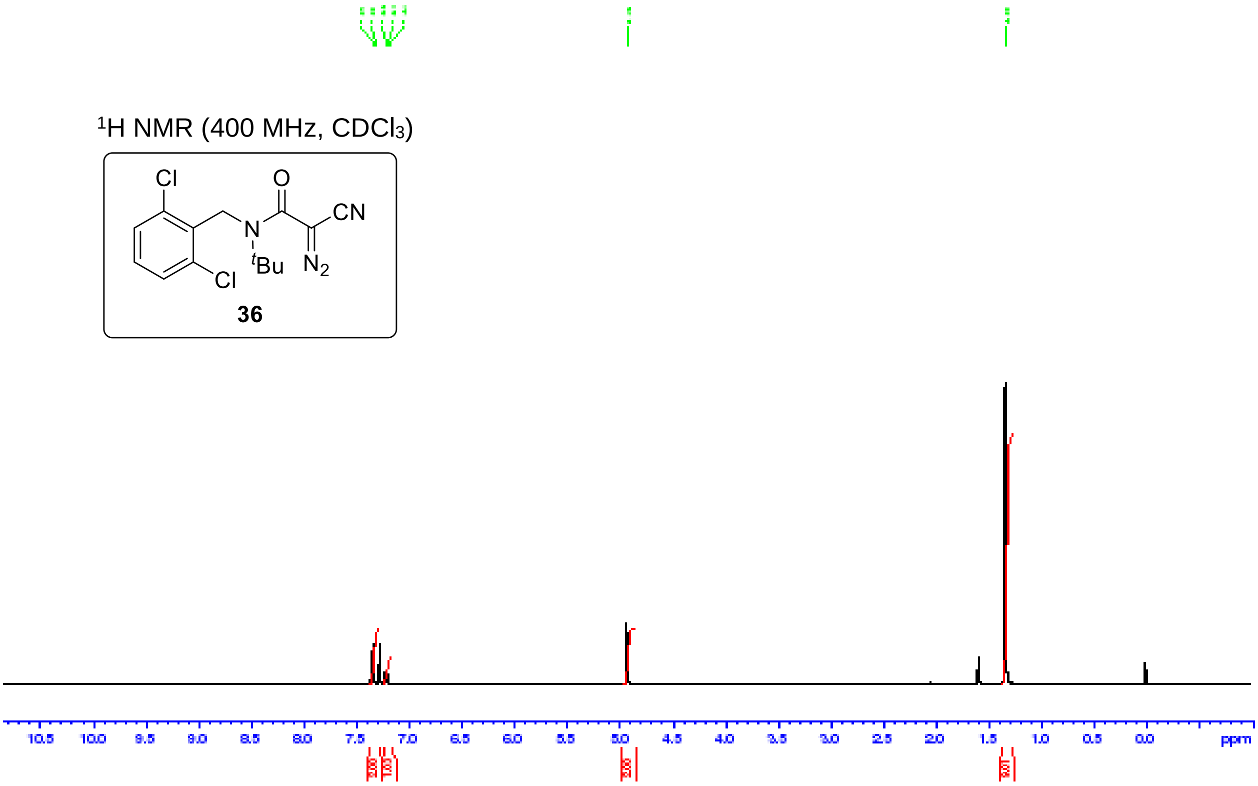

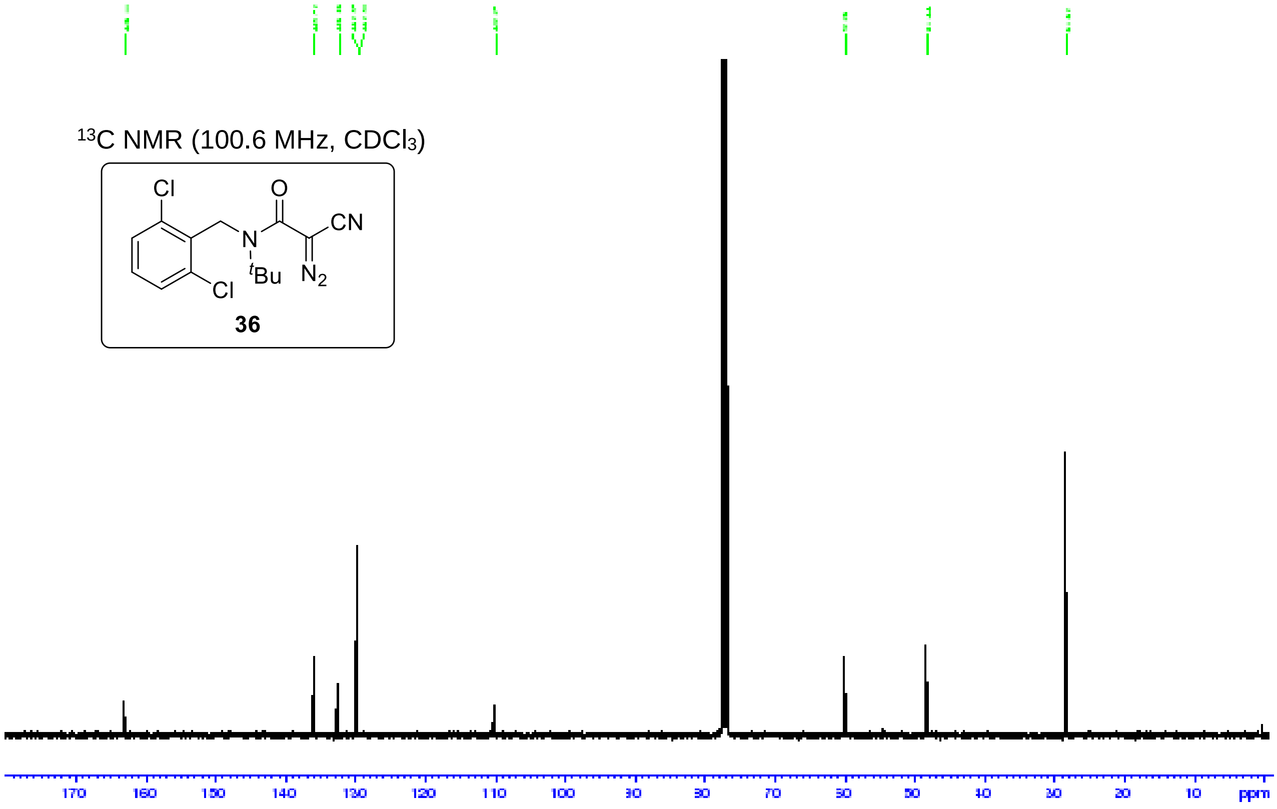

N-(*tert*-Butyl)-2-cyano-2-diazo-*N*-(4'-methylbenzyl)acetamide (37)

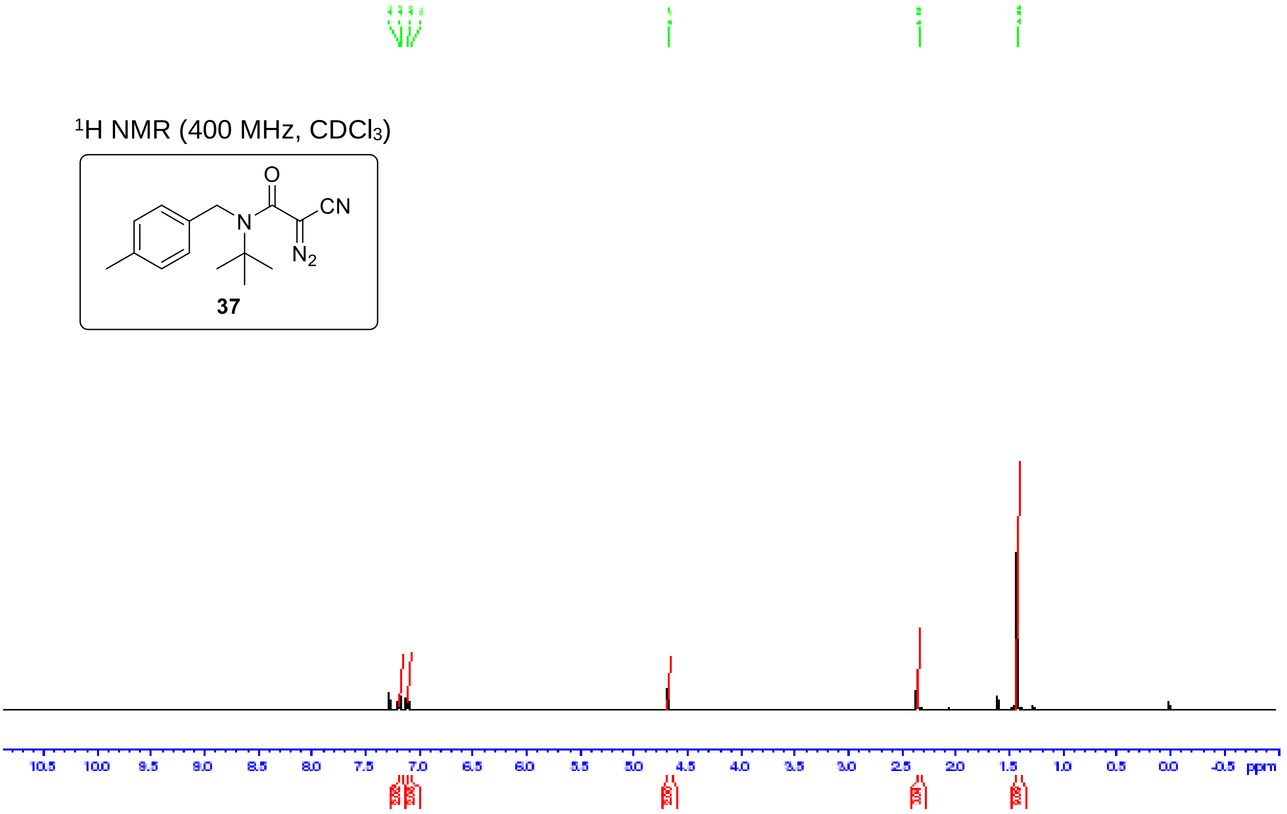

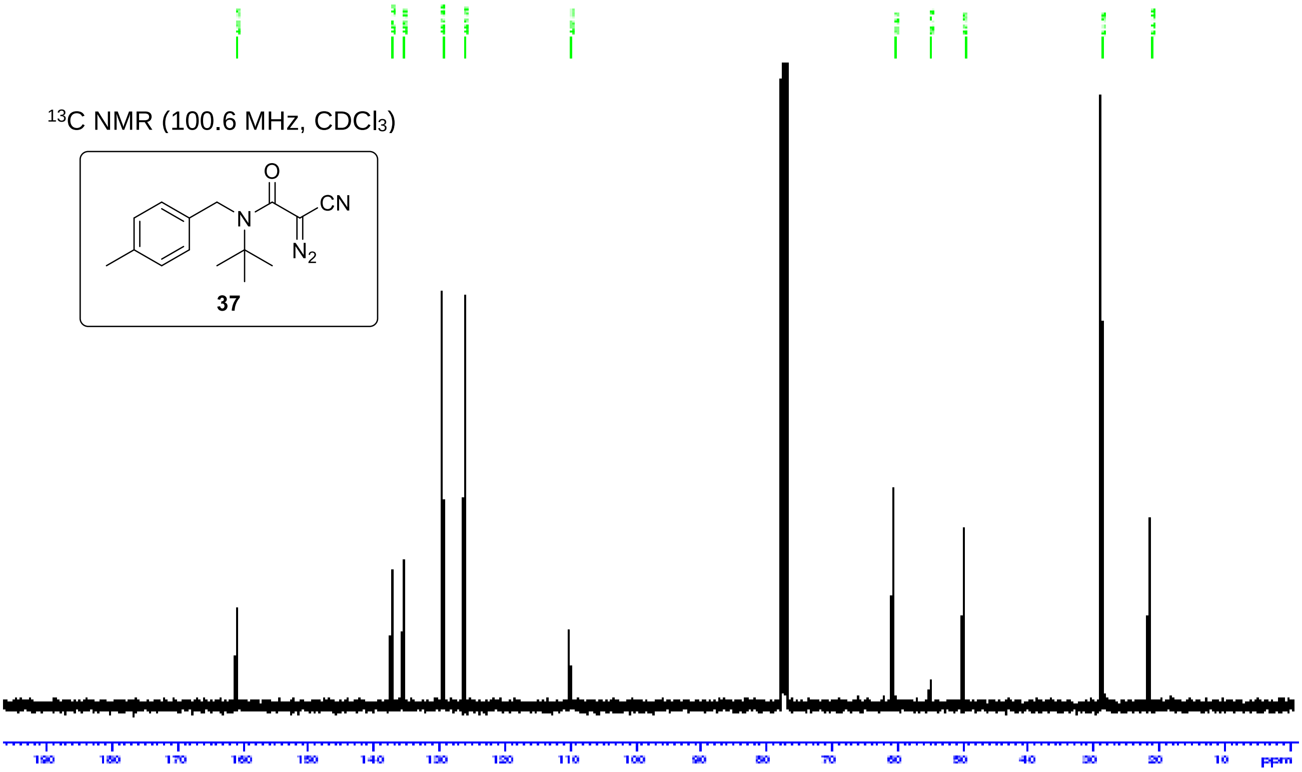

N-(tert-Butyl)-2-cyano-2-diazo-N-(3',5'-dimethylbenzyl)acetamide (38)

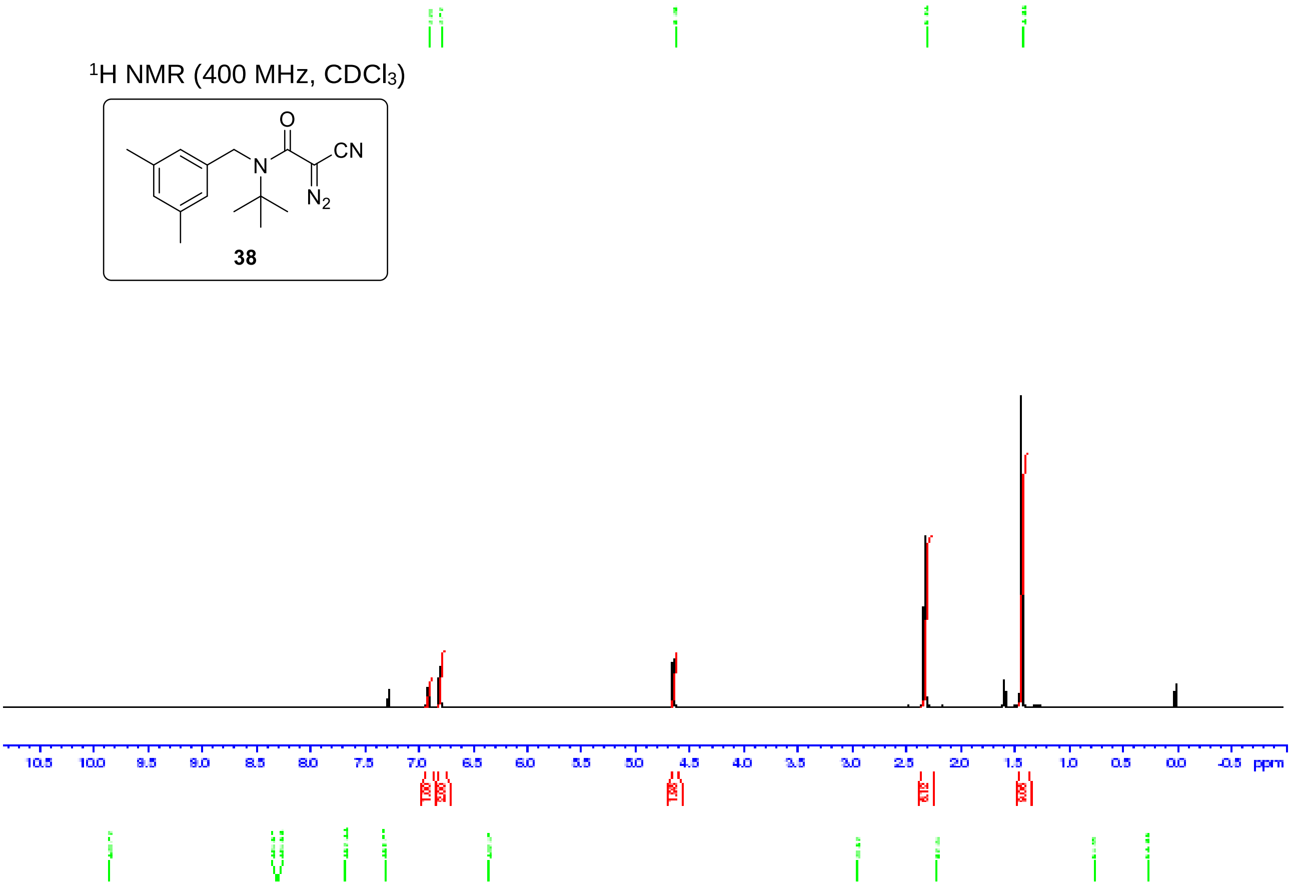

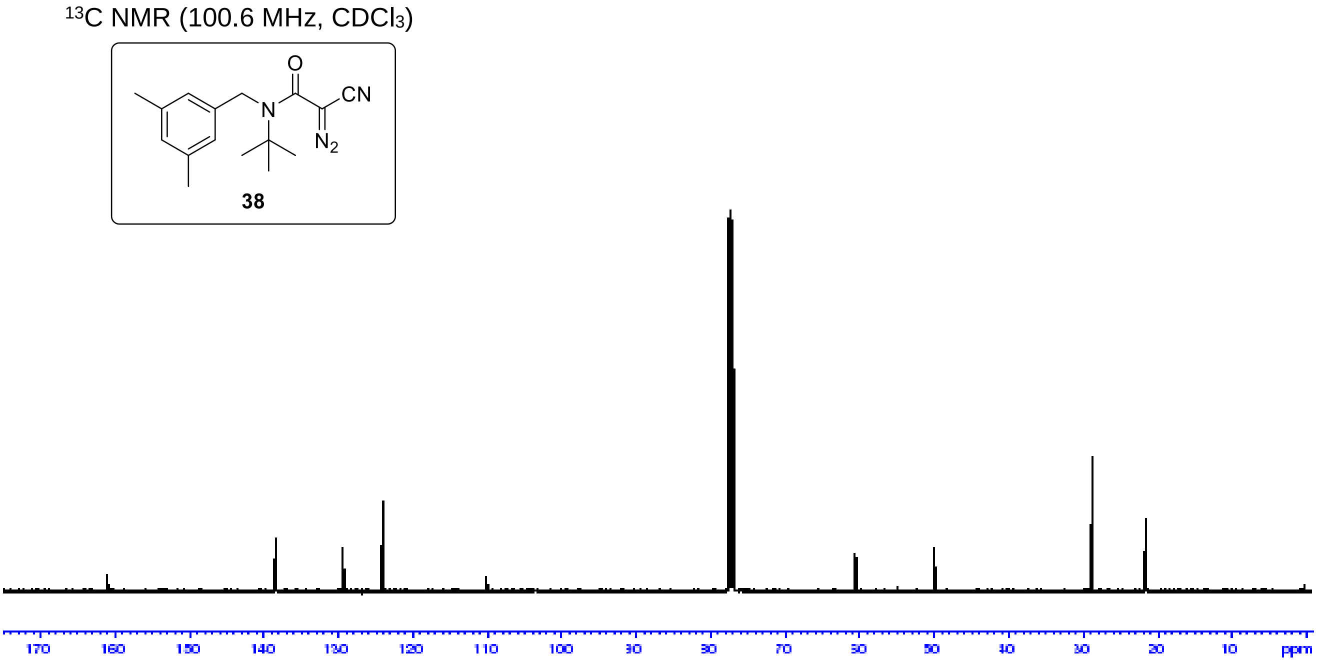

N-(*tert*-Butyl)-2-cyano-2-diazo-N-(2',4',6'-trimethylbenzyl)acetamide (39)

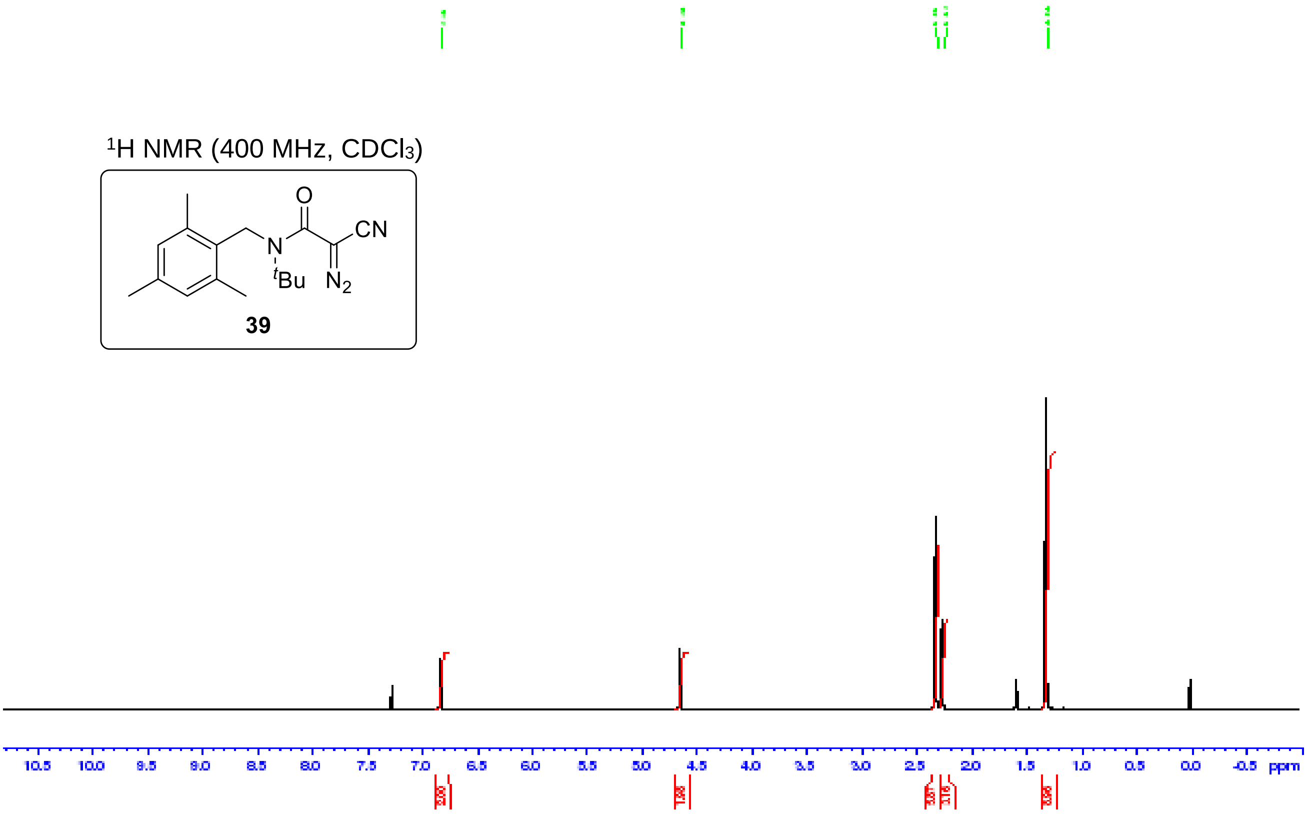

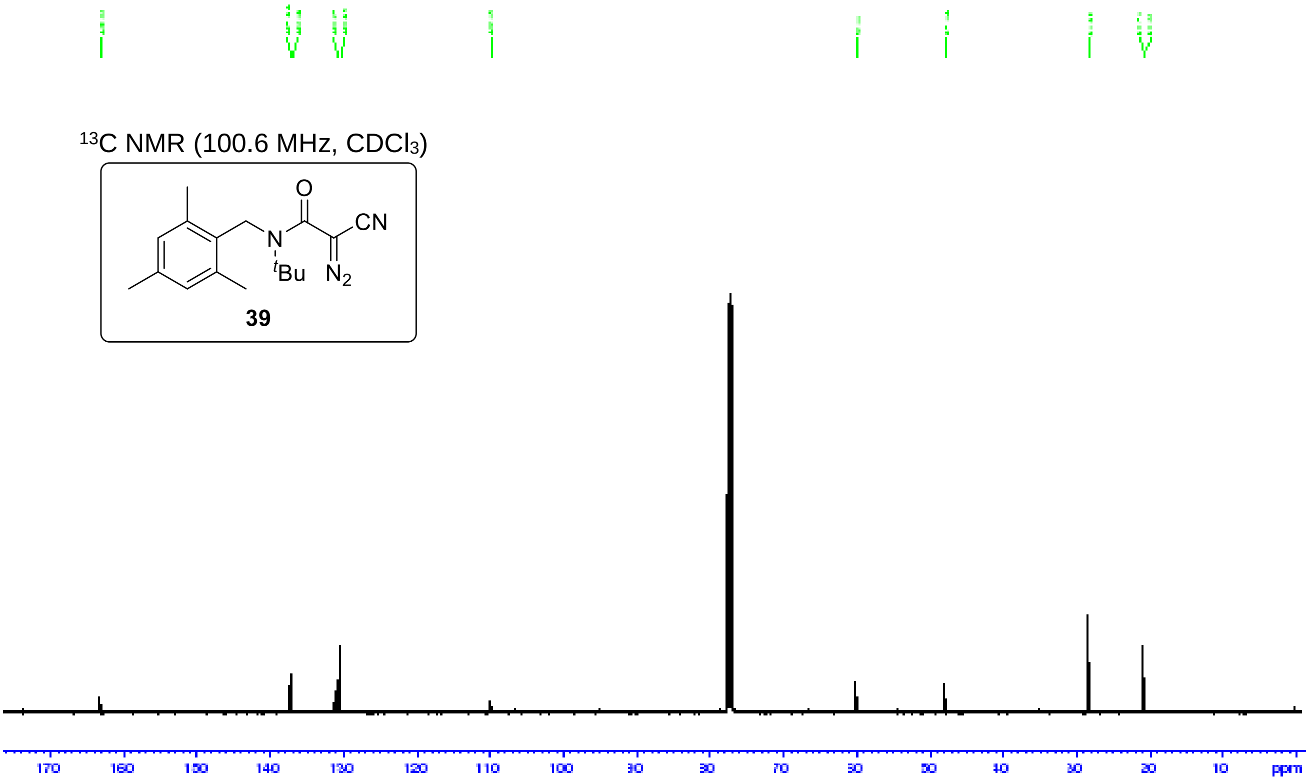

N-Benzyl-N-(tert-butyl)-2-cyano-2-diazoacetamide (40)

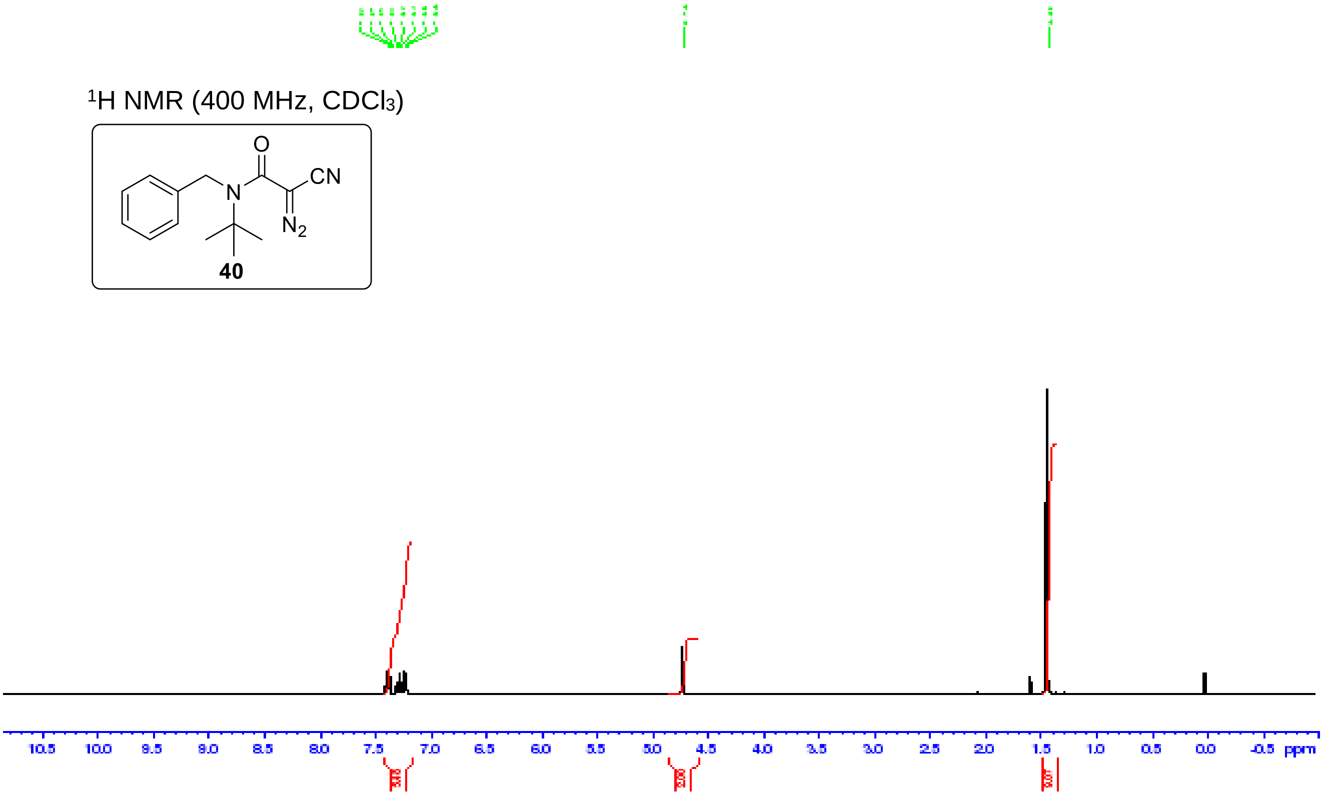

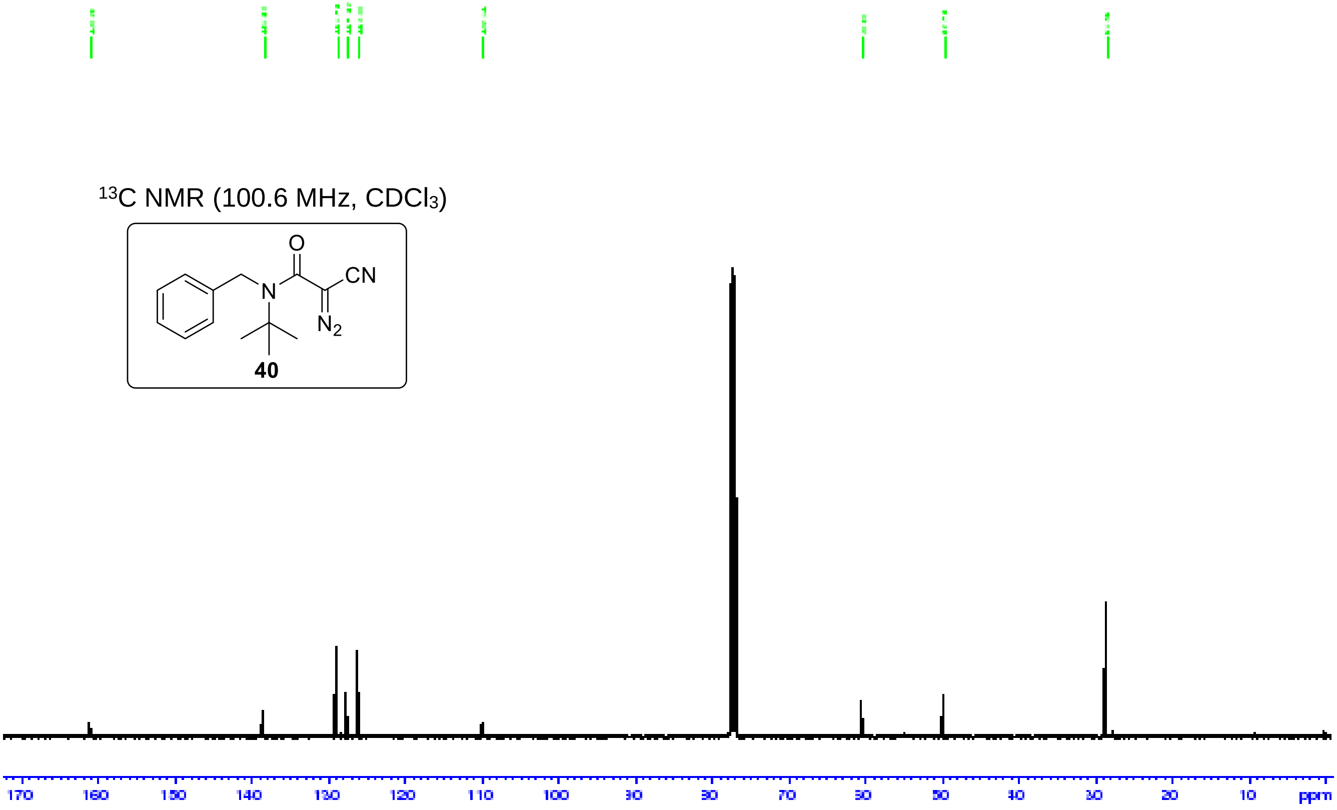

N-(*tert*-Butyl)-2-cyano-2-diazo-*N*-(4'-nitrobenzyl)acetamide (41)

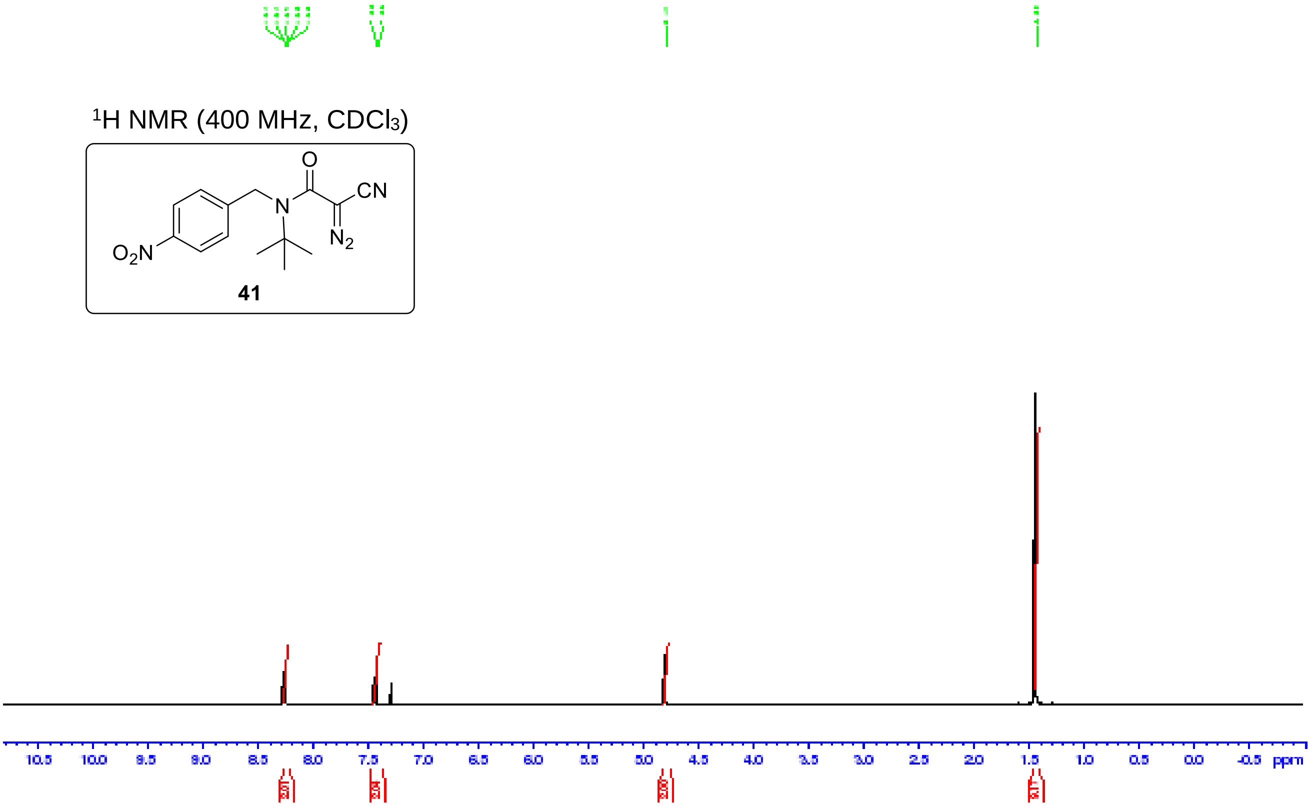

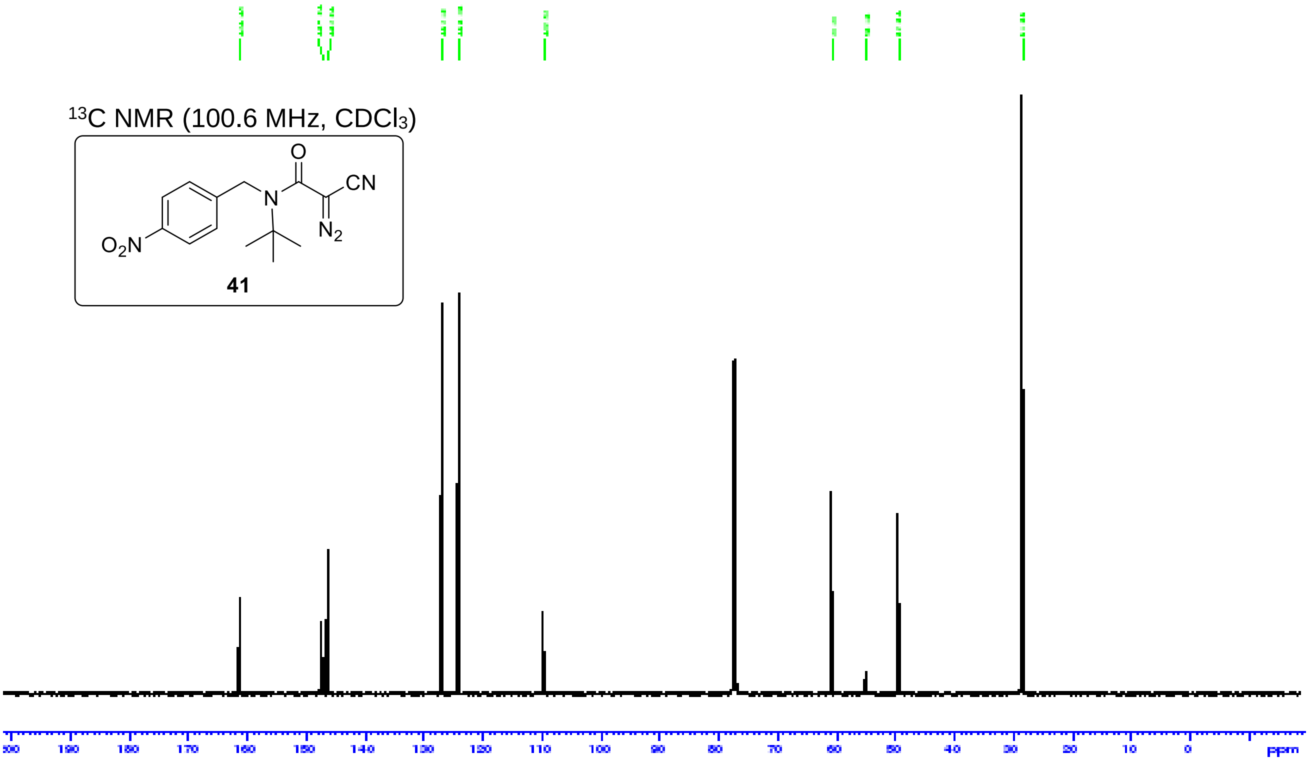

N-(*tert*-Butyl)-2-cyano-2-diazo-*N*-(4'-methoxycarbonylbenzyl)acetamide (42)

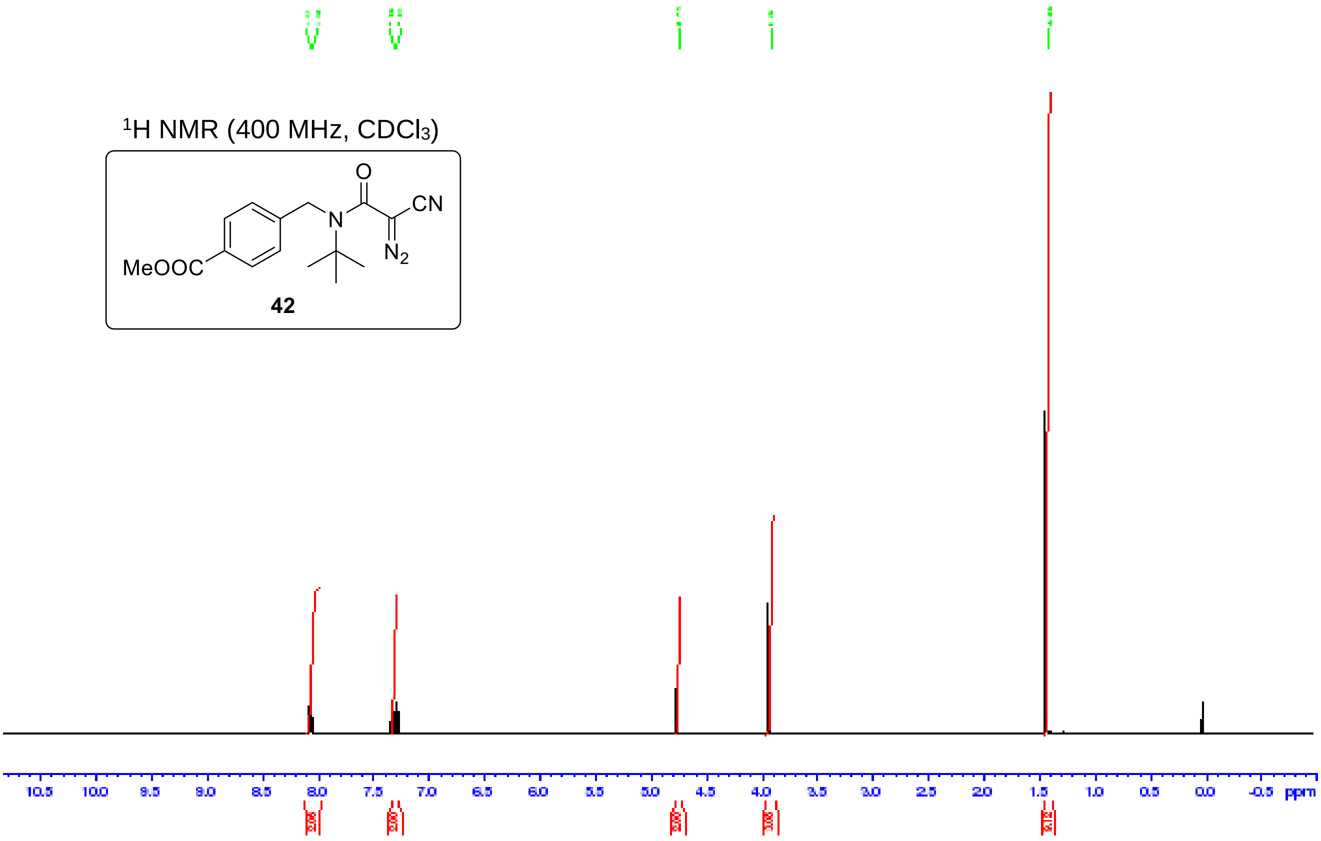

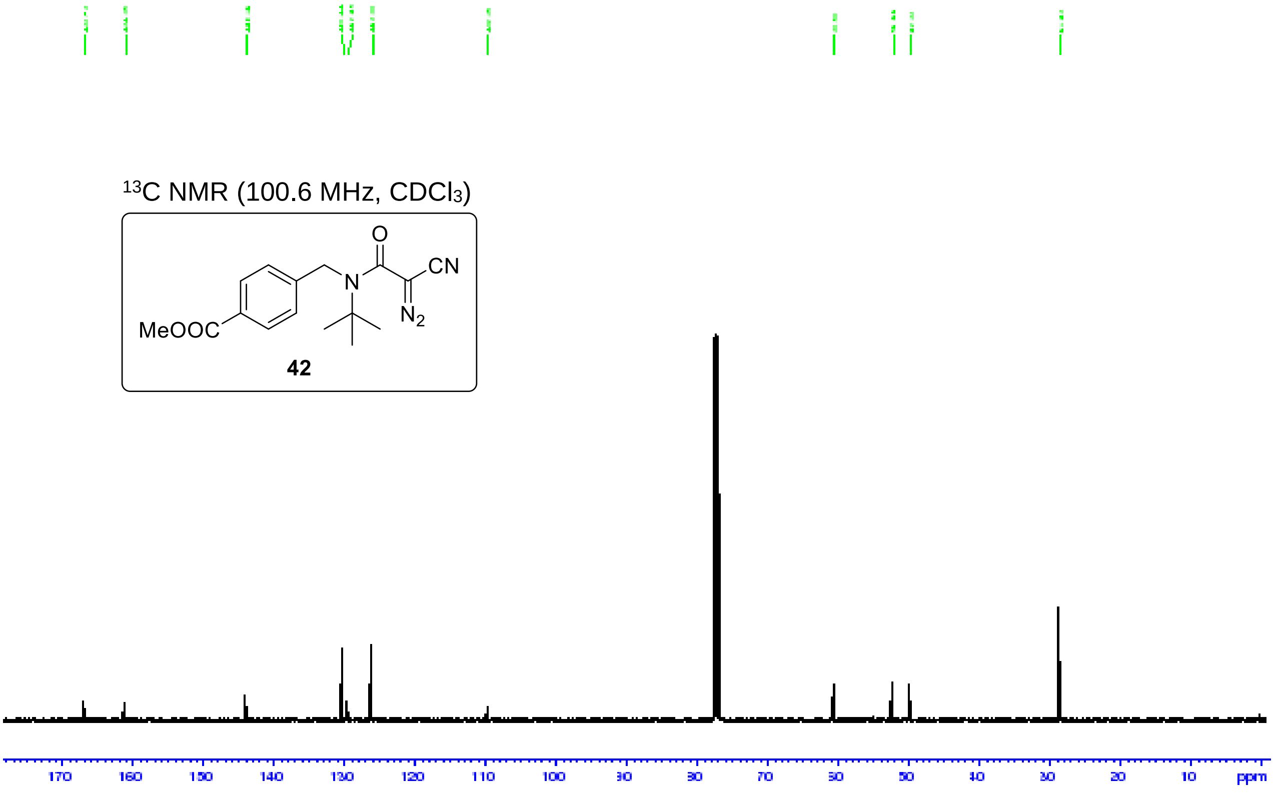

N-(tert-Butyl)-2-cyano-2-diazo-N-(4'-trifluoromethylbenzyl)acetamide (43)

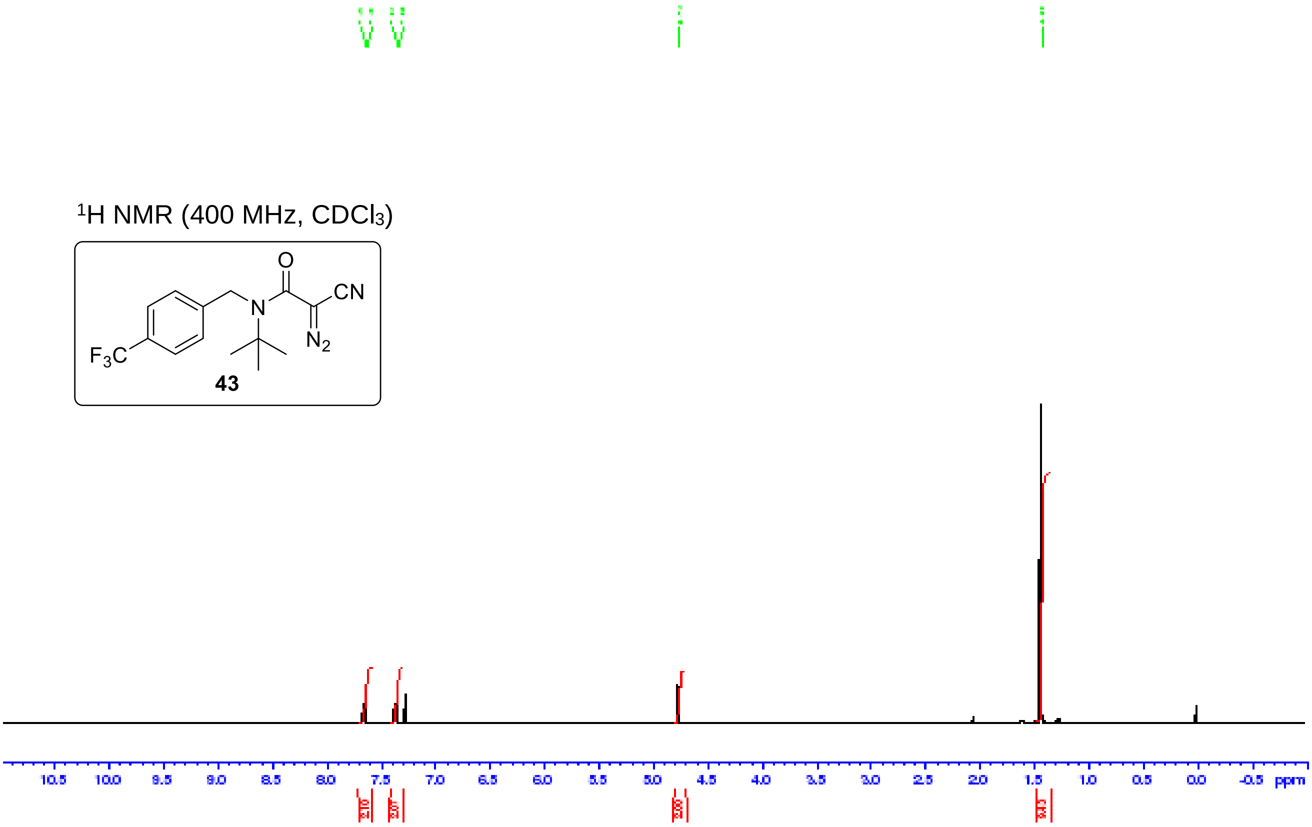

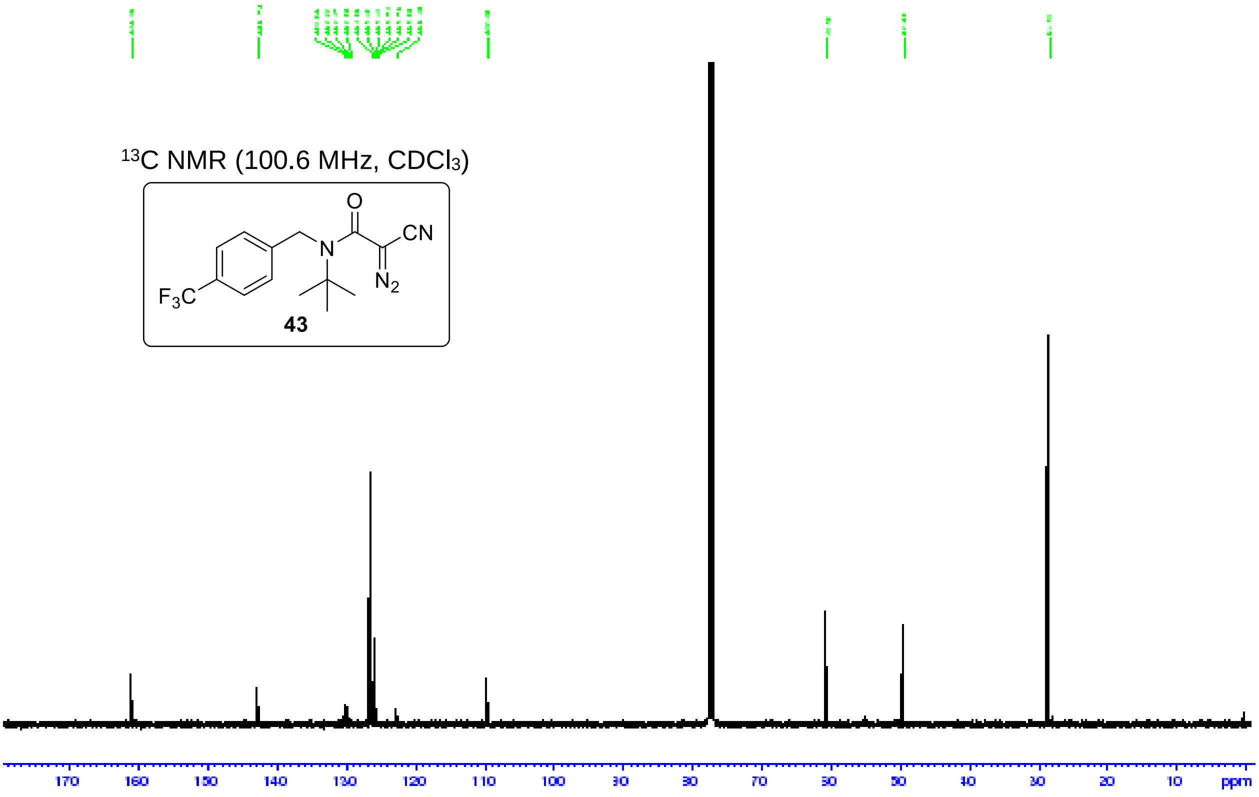

^{19}F NMR (376.5 MHz, $CDCl_3$)

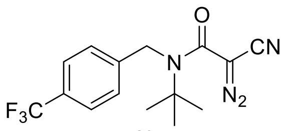

43

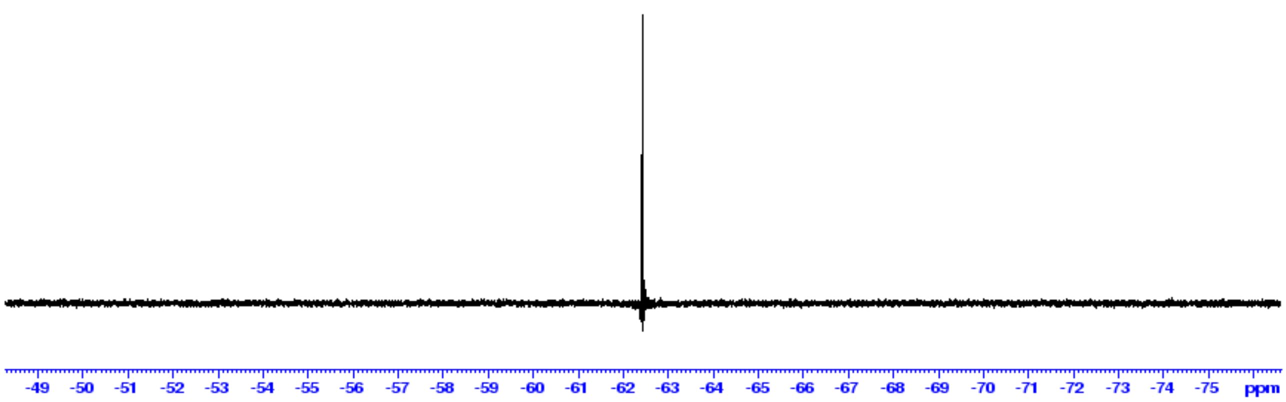

N-(*tert*-Butyl)-2-cyano-2-diazo-*N*-(4'-cyanobenzyl)acetamide (44)

^{1}H NMR (400 MHz, $CDCl_3$)

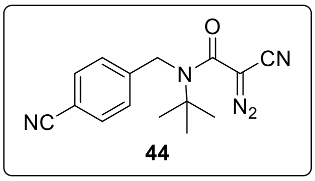

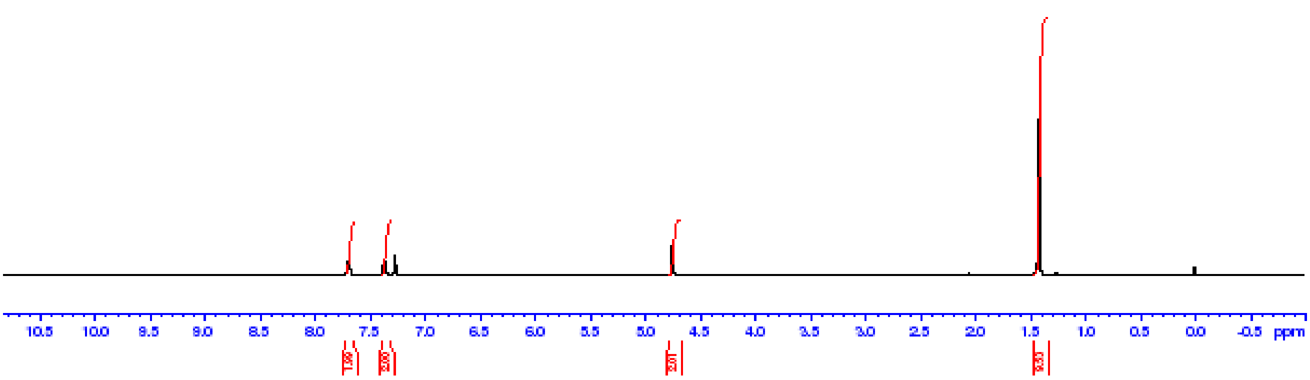

^{13}C NMR (100.6 MHz, $CDCl_3$)

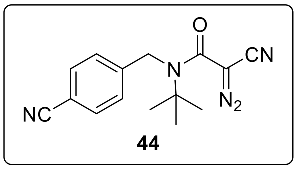

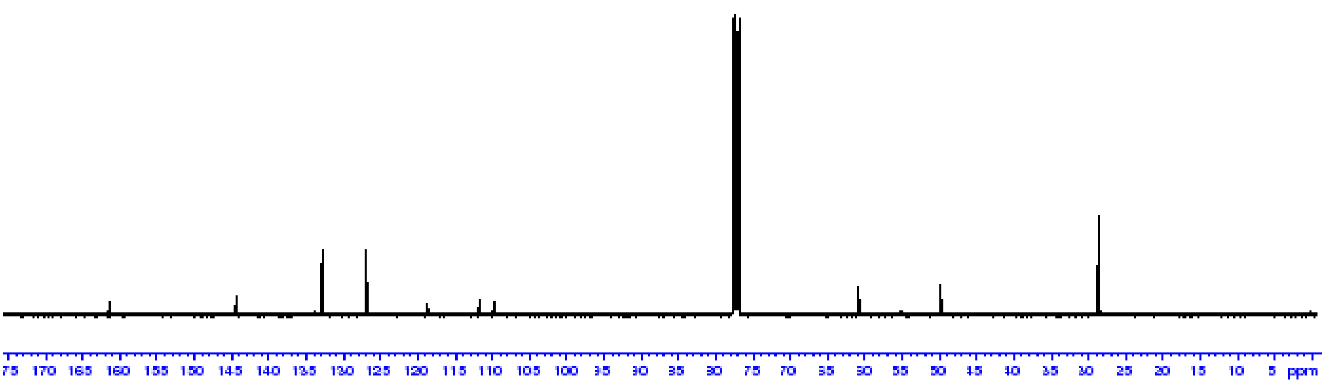

N-(*tert*-Butyl)-2-cyano-2-diazo-*N*-(4'-methoxybenzyl)acetamide (45)

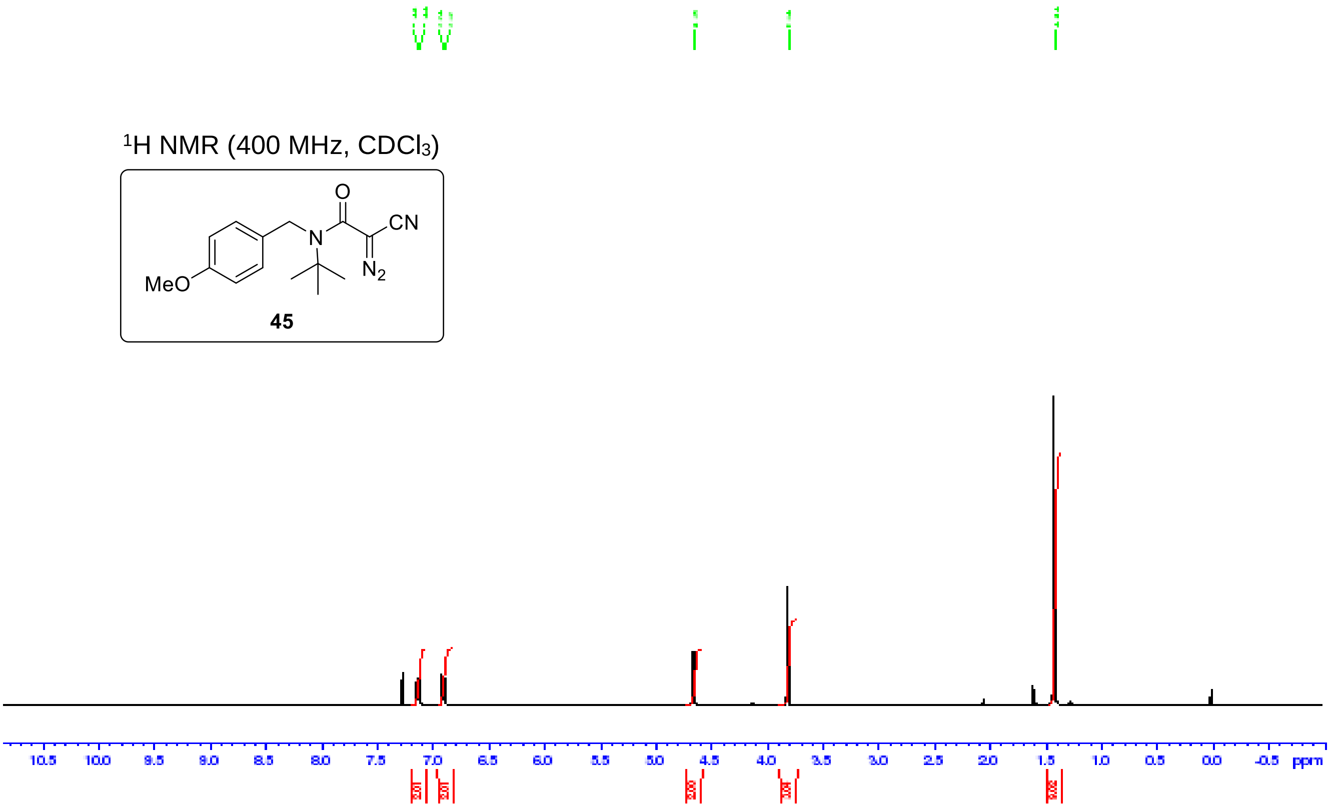

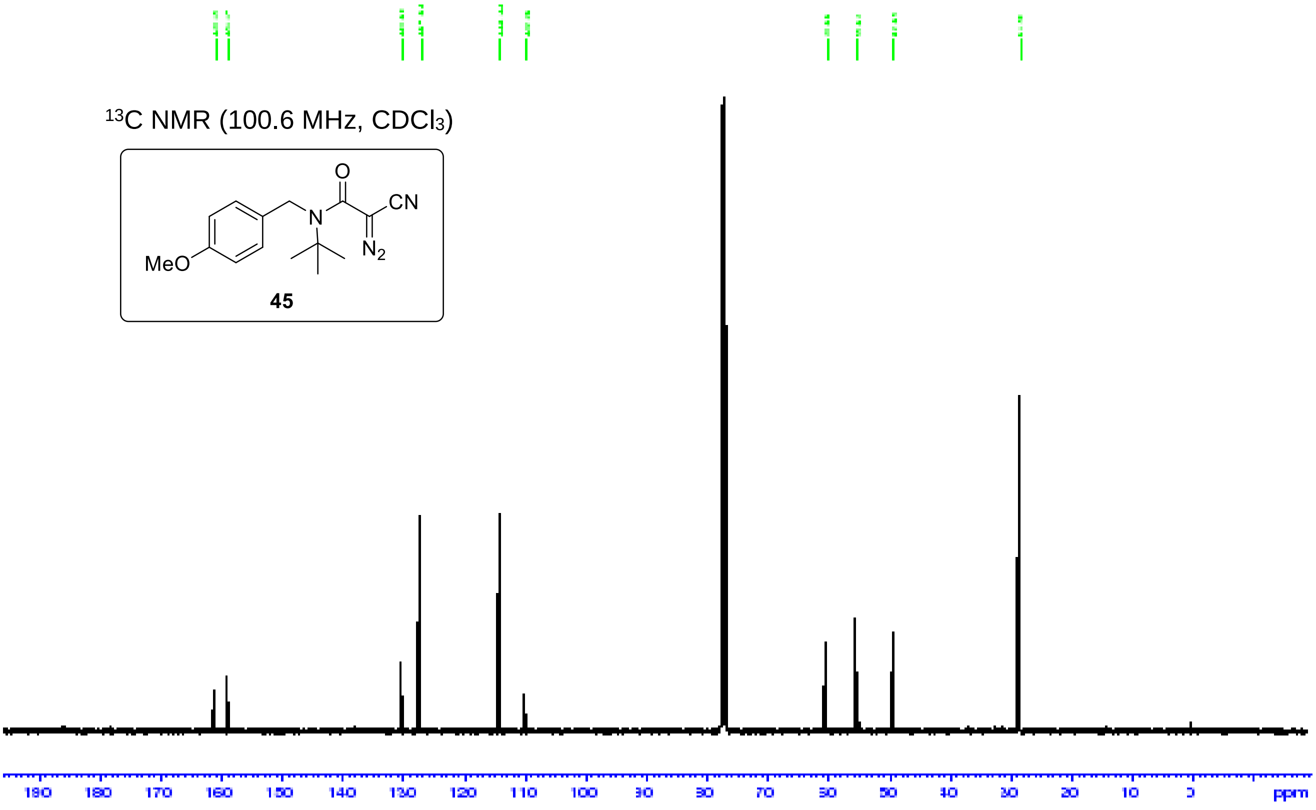

9-Aza-9-benzyl-1(*S*)-cyano-4-bicyclo[5.3.0]deca-2,4,6-trien-10-one (32)

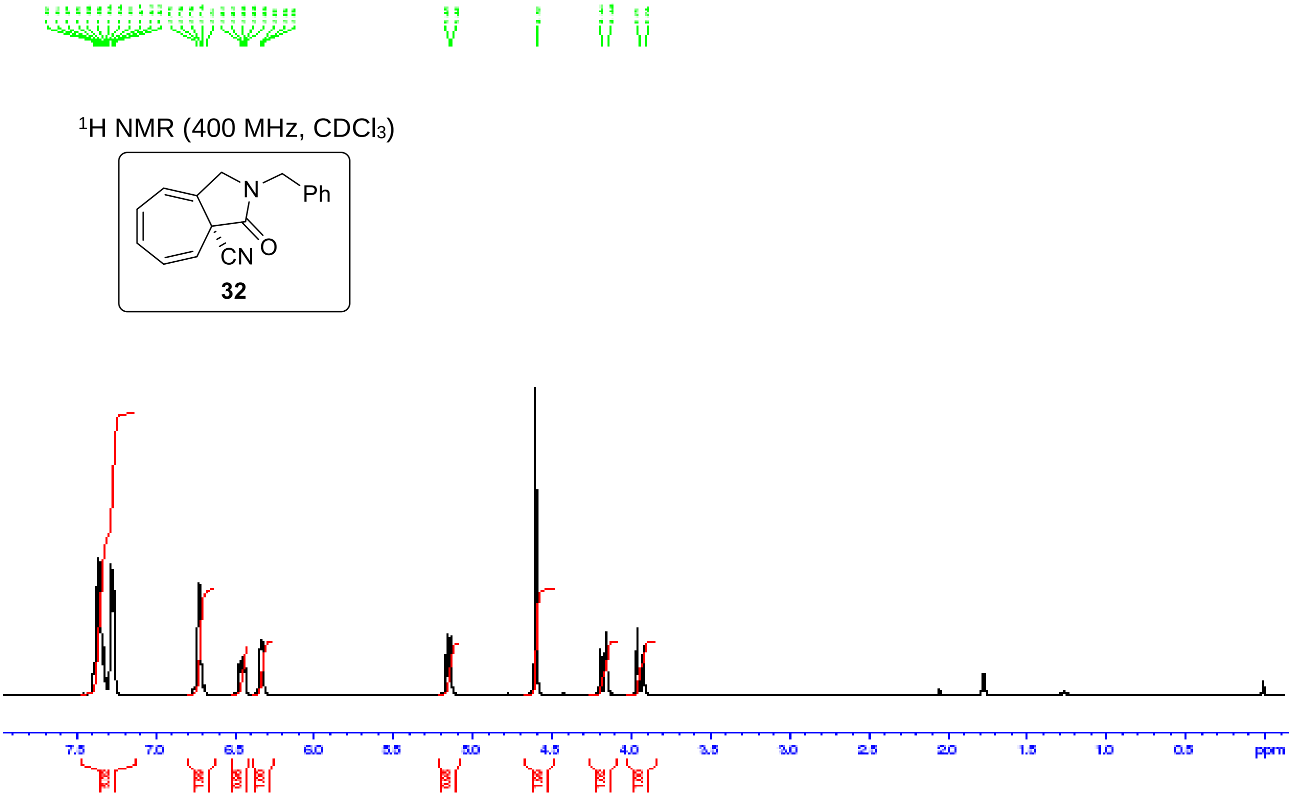

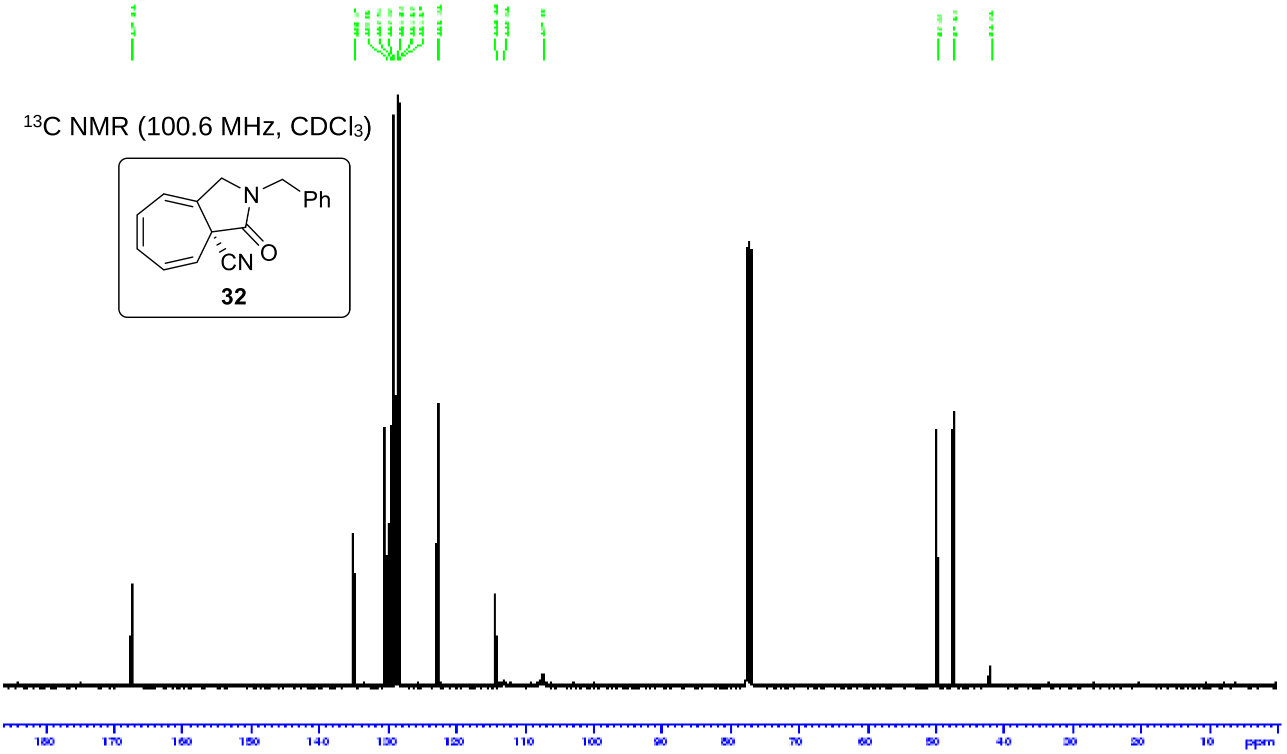

9-Aza-9-*tert*-butyl-1(*S*)-cyano-4-fluorobicyclo[5.3.0]deca-2,4,6-trien-10-one (46)

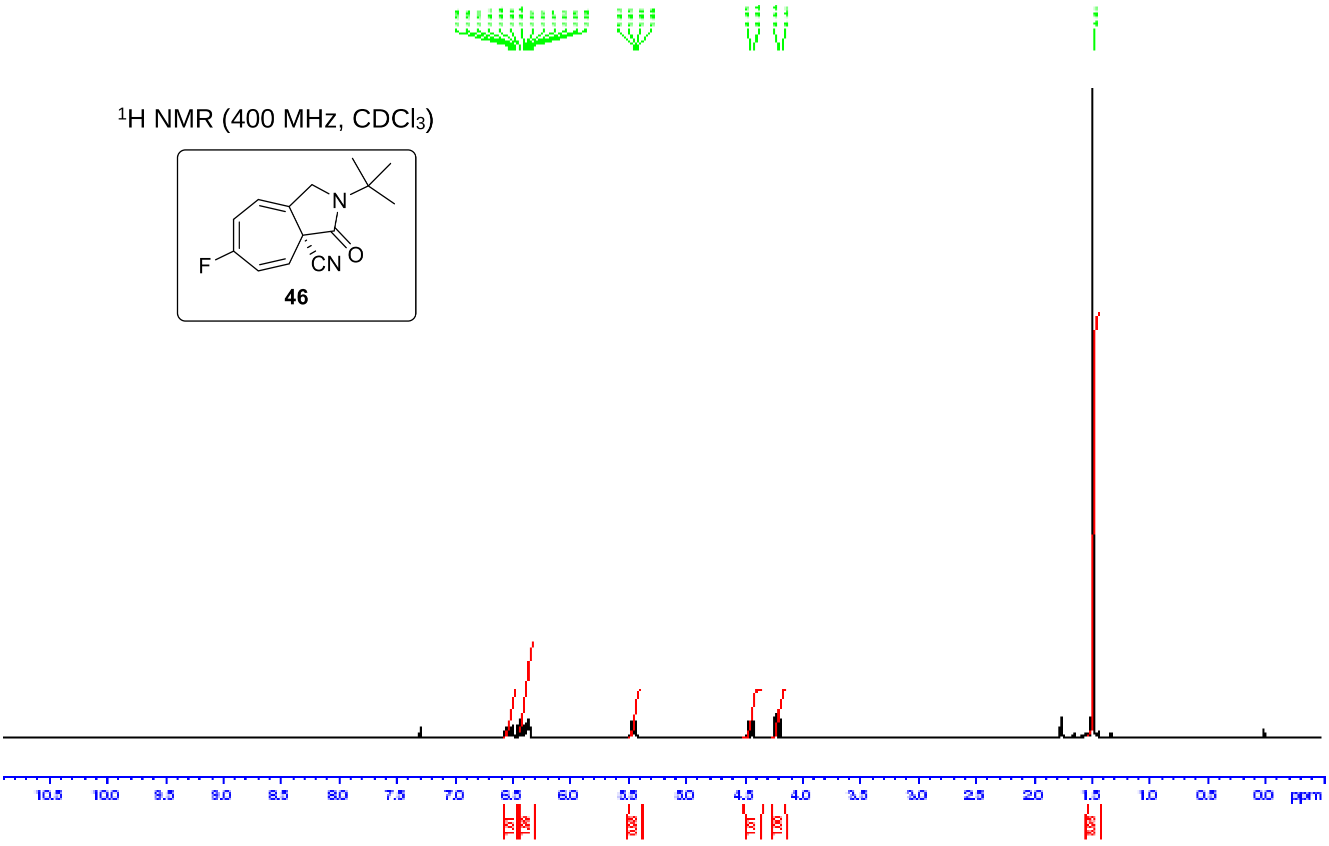

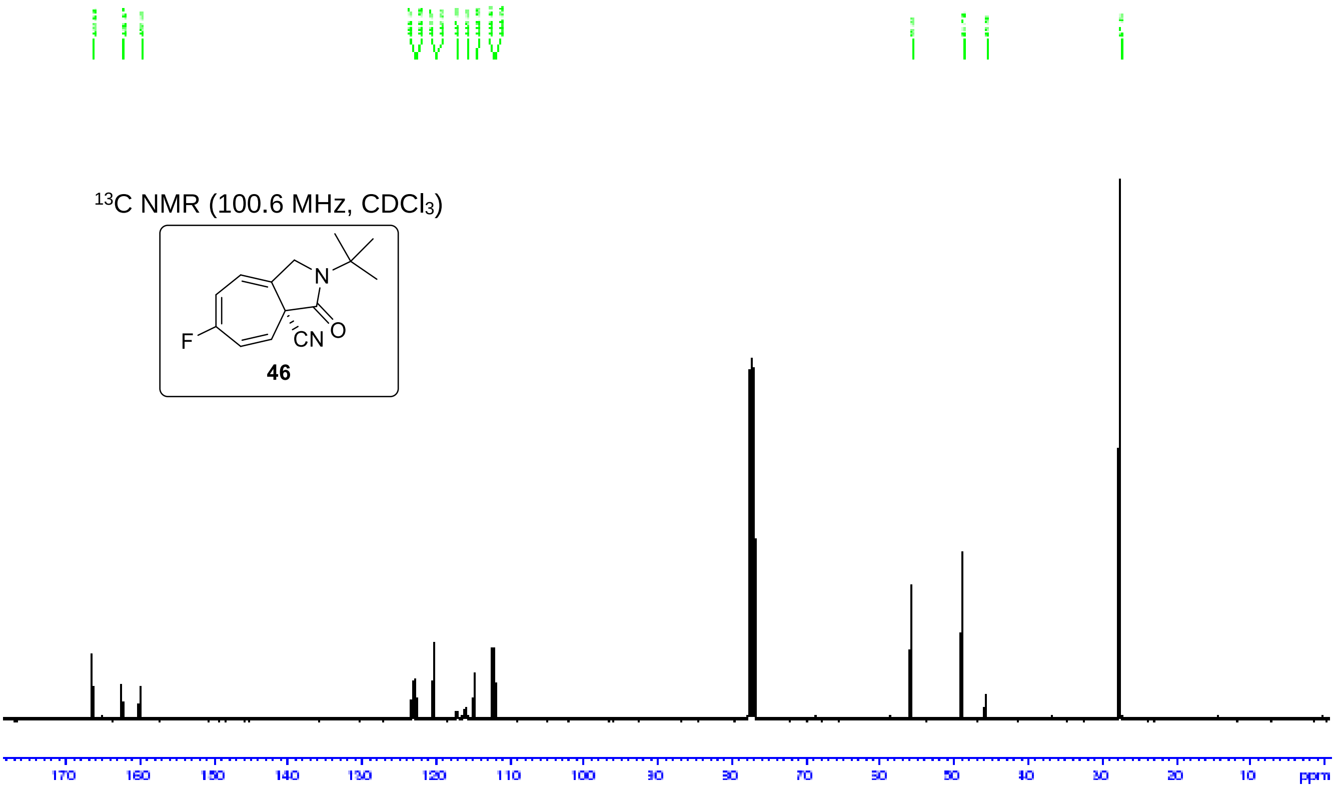

^{19}F NMR (376.5 MHz, $CDCl_3$)

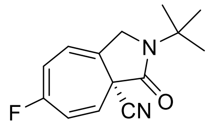

46

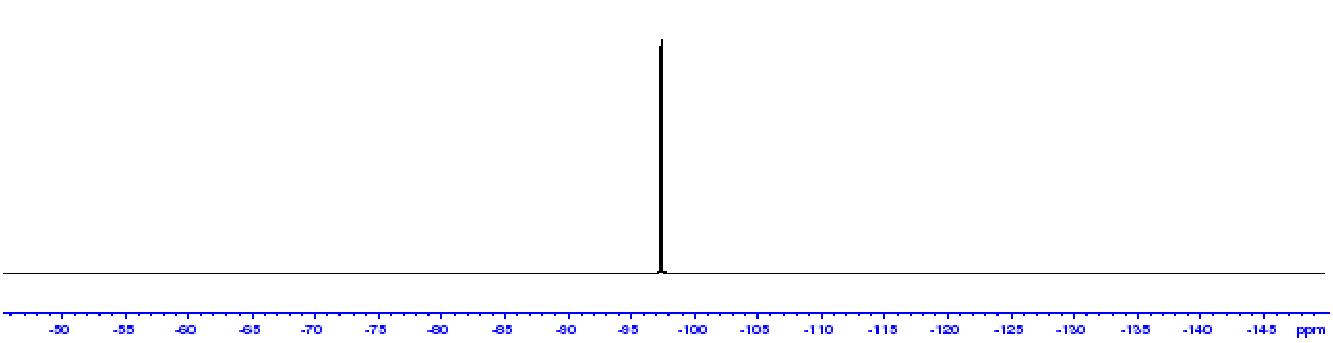

9-Aza-9-*tert*-butyl-1(*S*)-cyano-4-bromobicyclo[5.3.0]deca-2,4,6-trien-10-one (47)

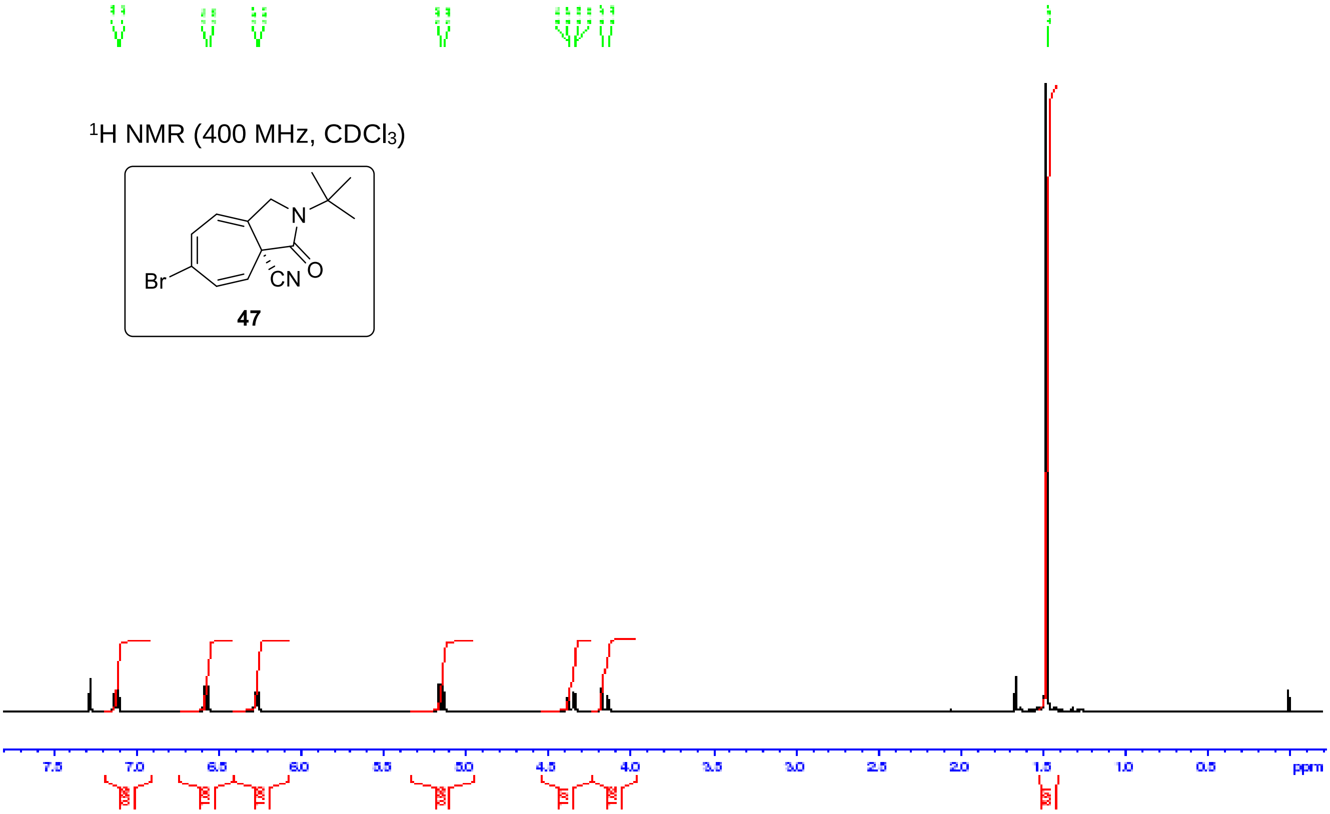

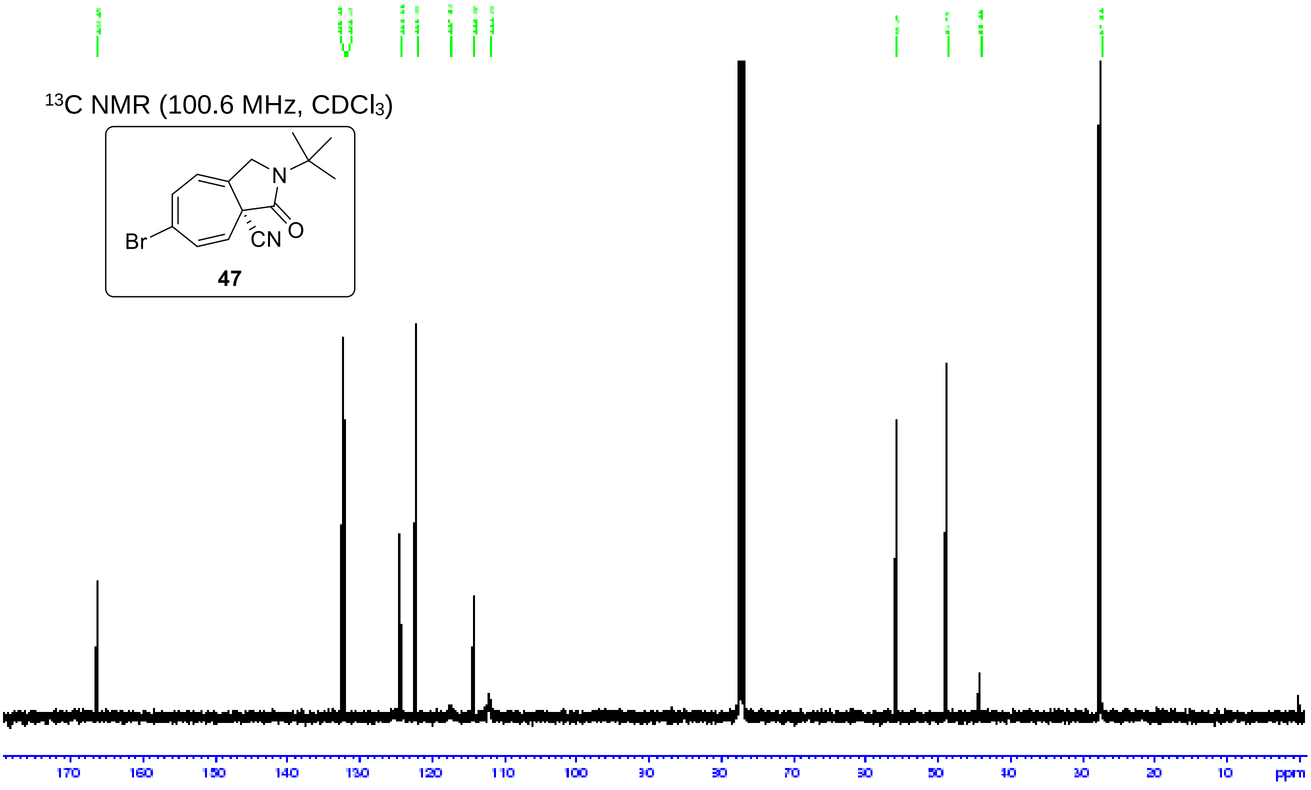

9-Aza-9-*tert*-butyl-1(*S*)-cyano-4-chlorobicyclo[5.3.0]deca-2,4,6-trien-10-one (48)

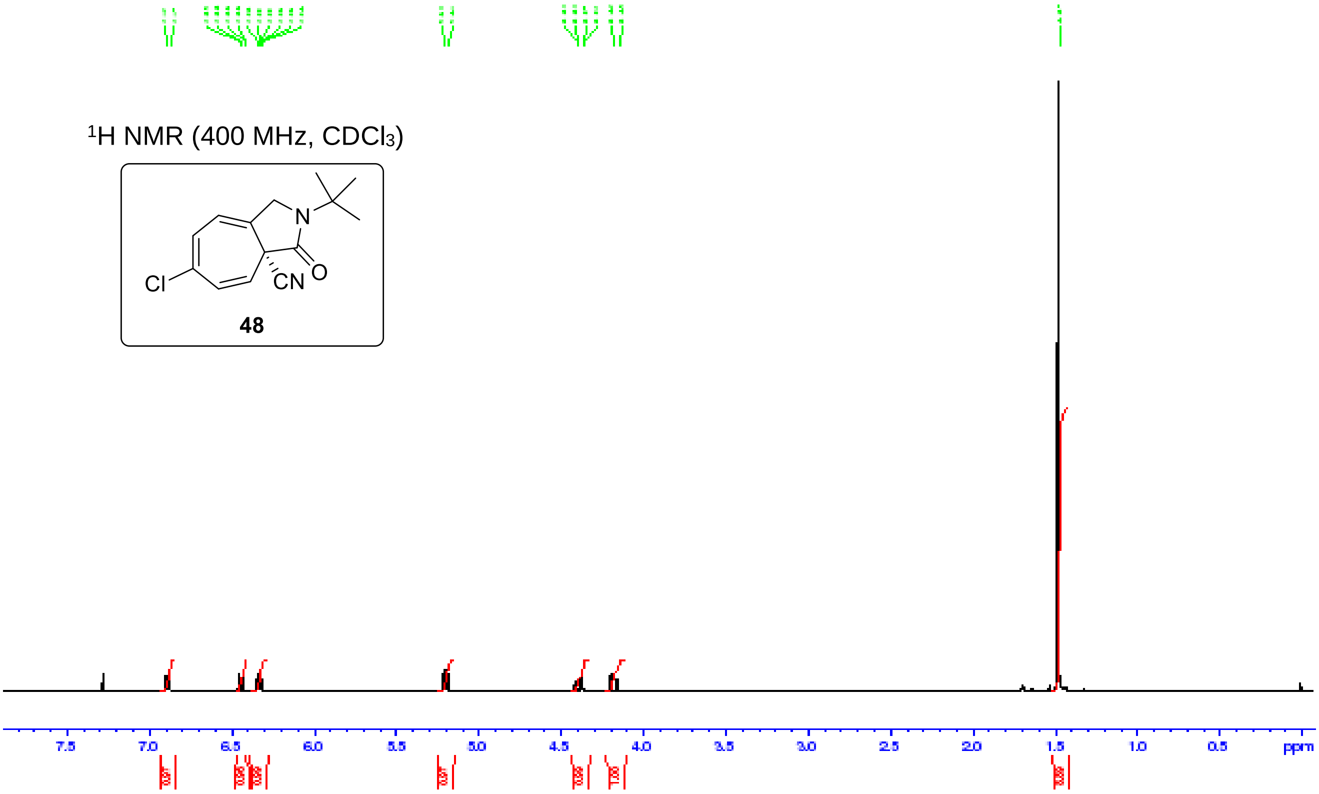

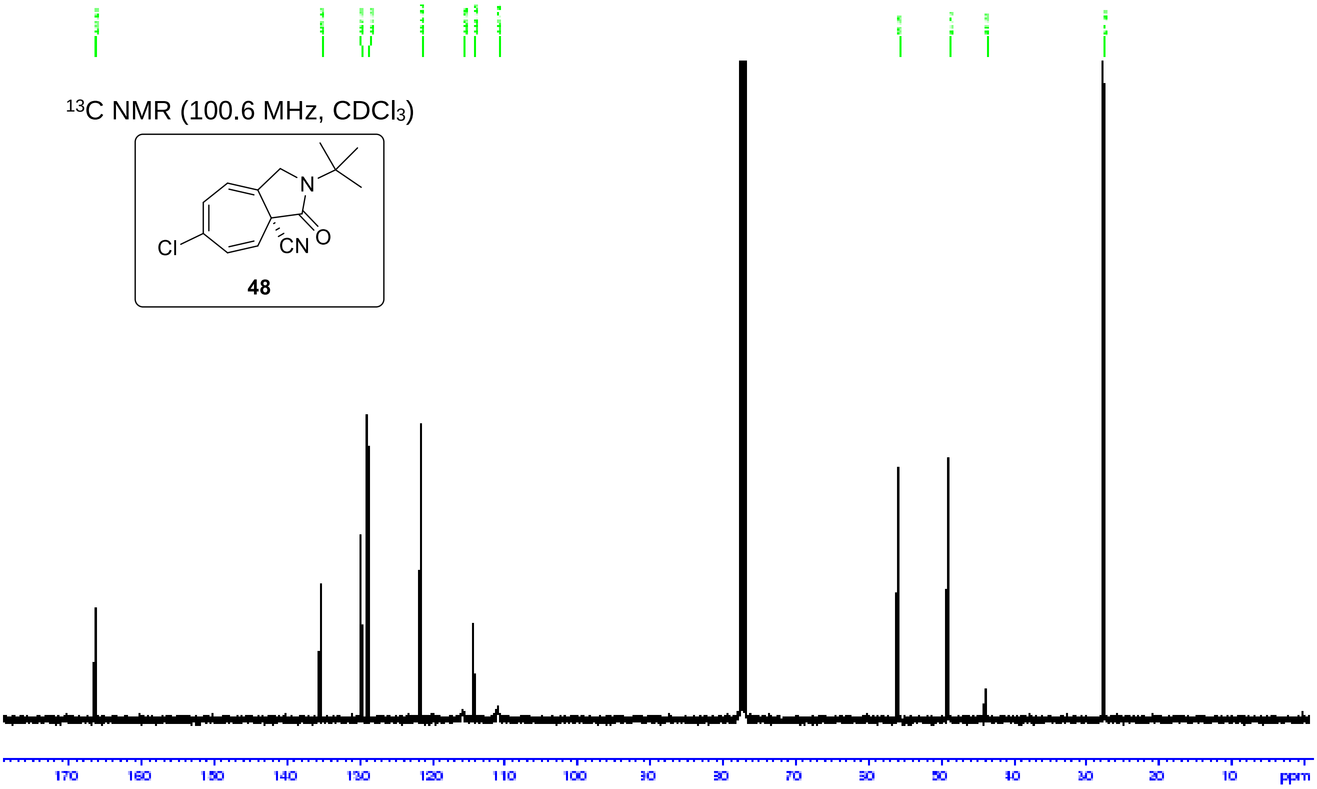

9-Aza-9-*tert*-butyl-1(*R*)-cyano-2,6-dichlorobicyclo[5.3.0]deca-2,4,6-trien-10-one (49)

^{1}H NMR (400 MHz, $CDCl_3$)

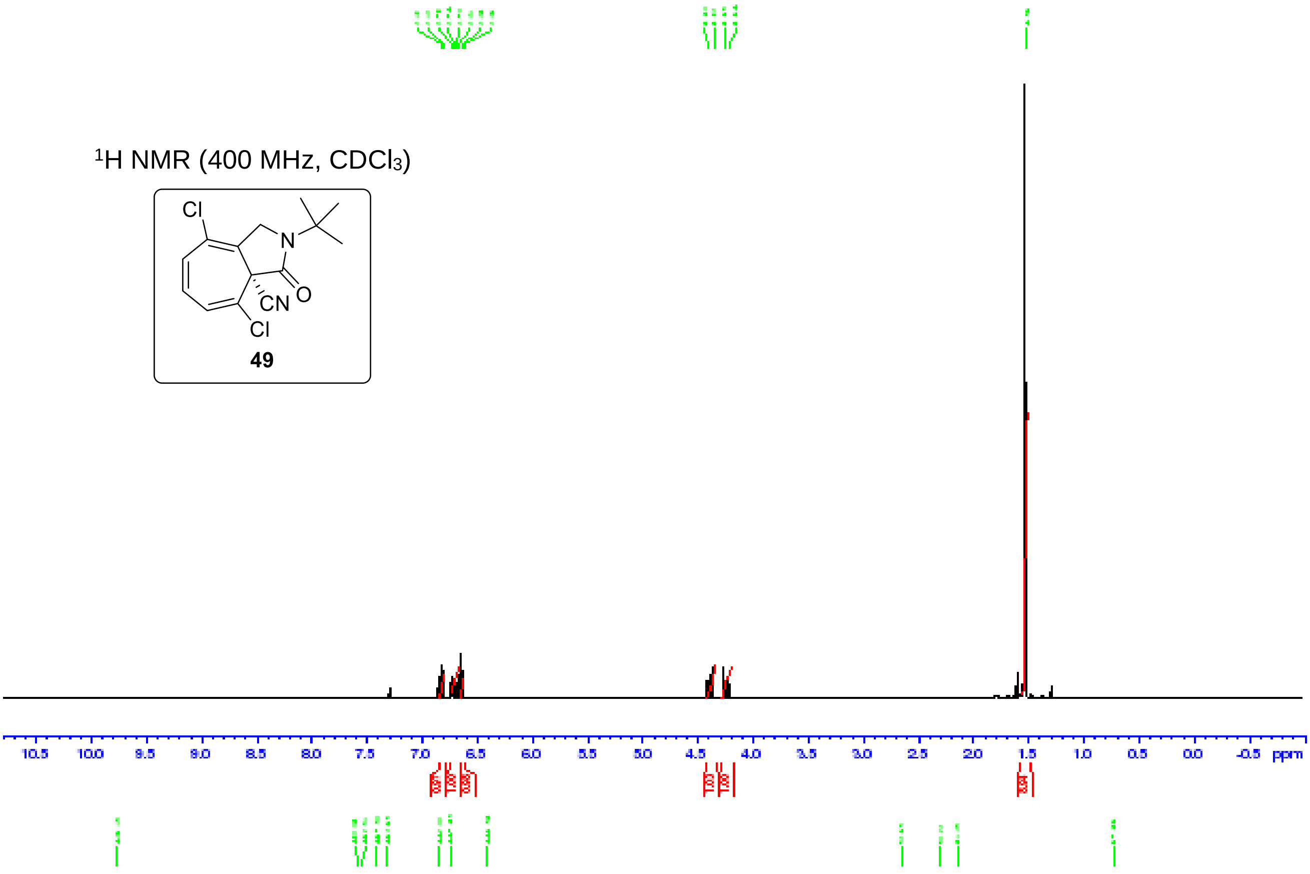

^{13}C NMR (100.6 MHz, $CDCl_3$)

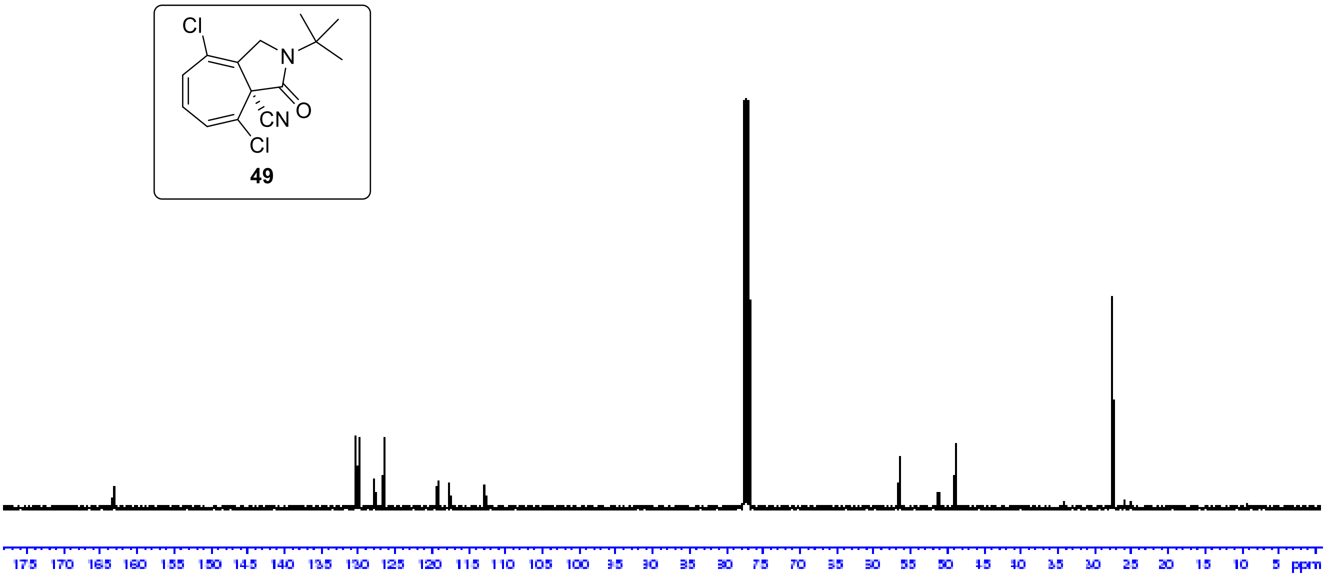

9-Aza-9-*tert*-butyl-1(*S*)-cyano-4-methylbicyclo[5.3.0]deca-2,4,6-trien-10-one (50)

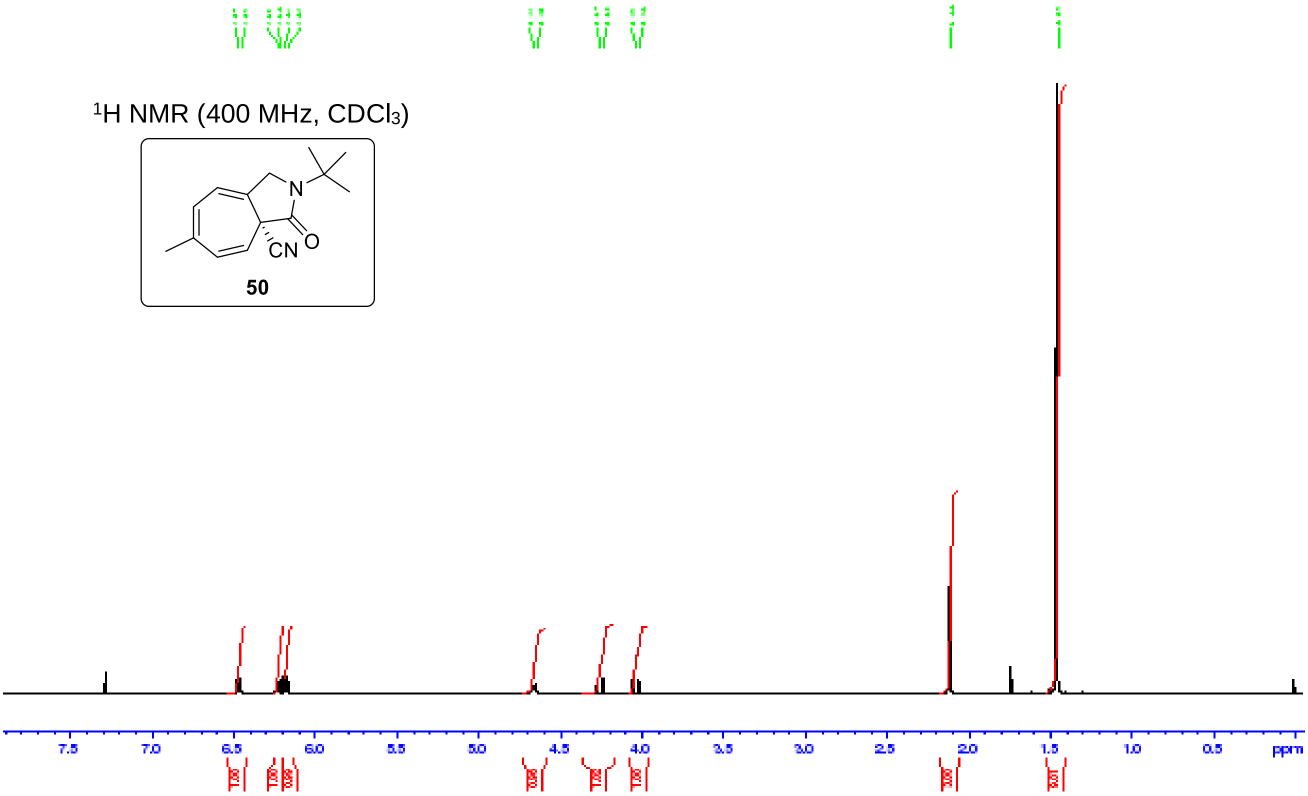

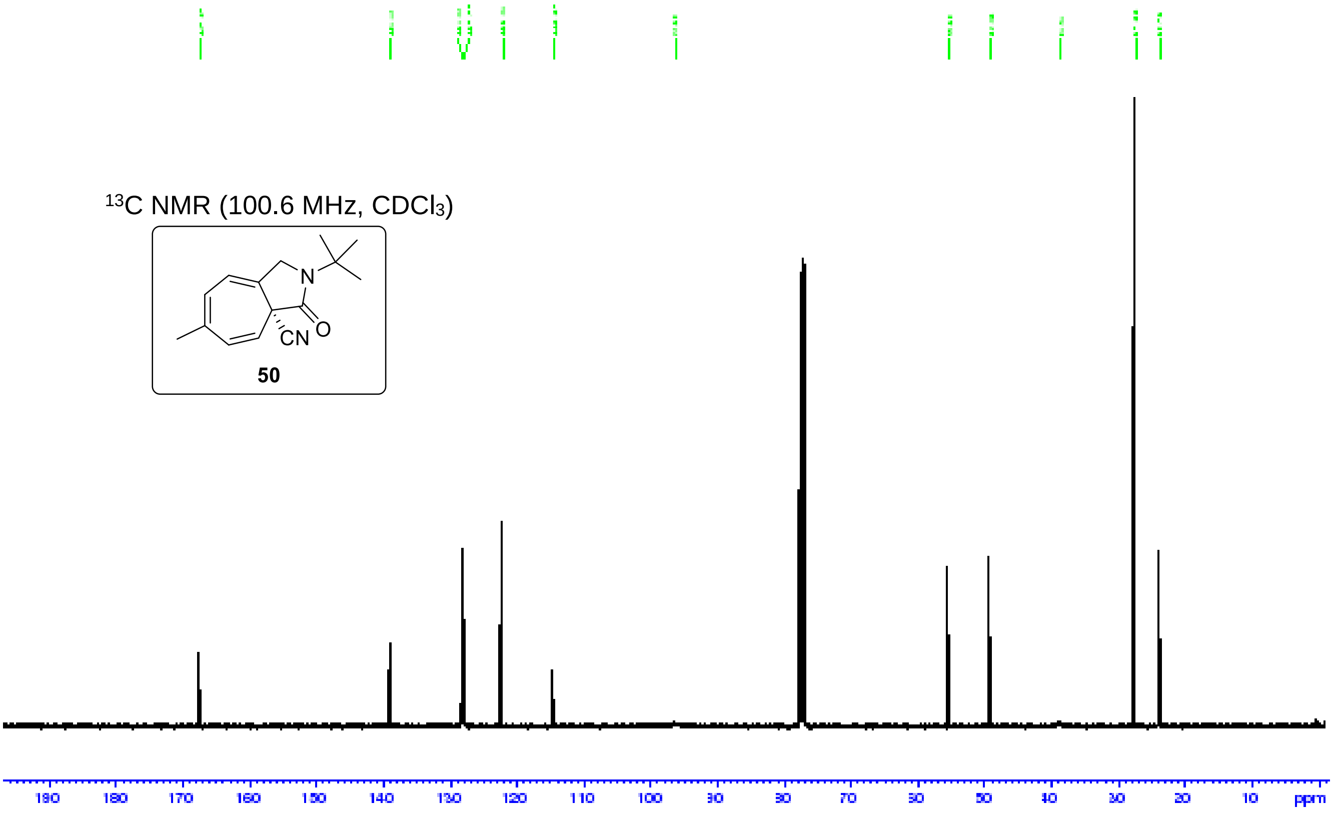

9-Aza-9-*tert*-butyl-1(*S*)-cyano-3,5-dimethylbicyclo[5.3.0]deca-2,4,6-trien-10-one (51)

^{1}H NMR (400 MHz, $CDCl_3$)

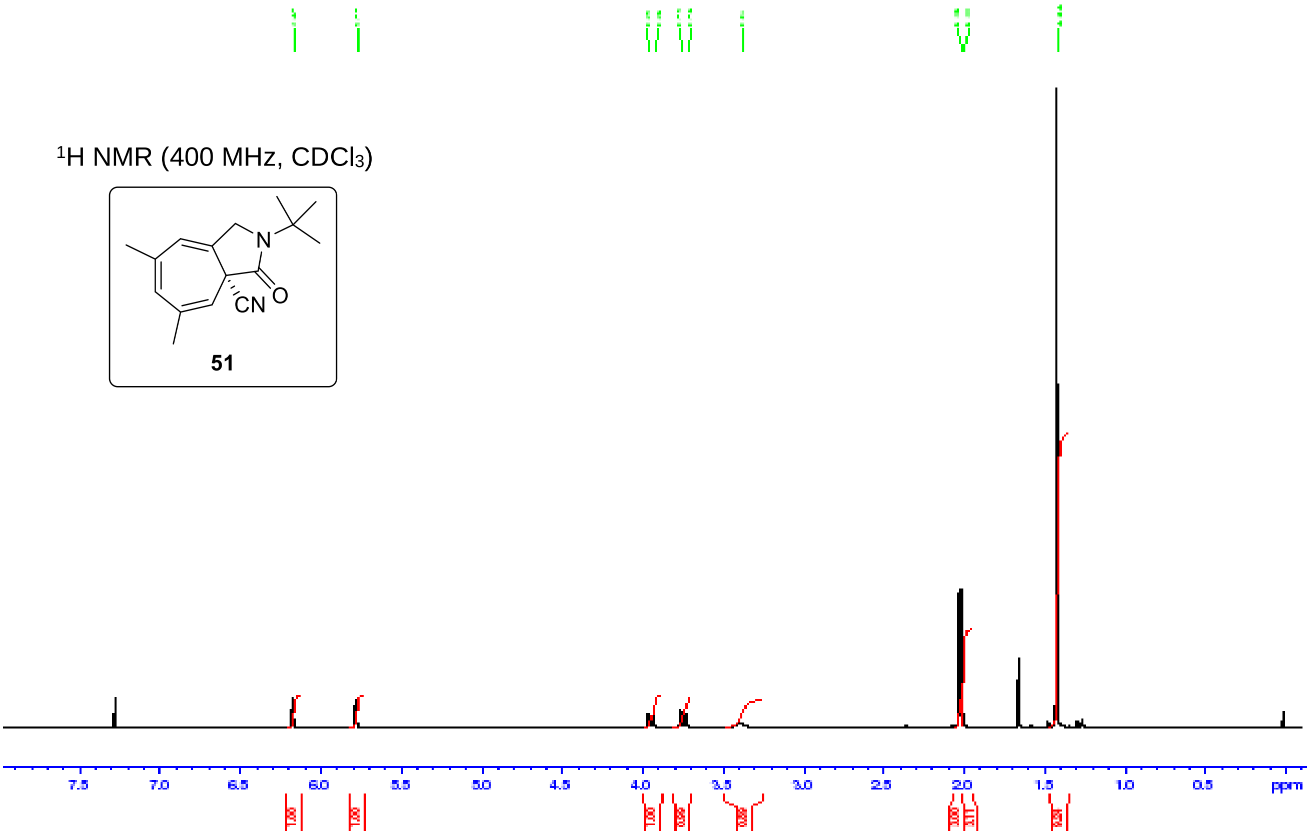

^{13}C NMR (100.6 MHz, $CDCl_3$)

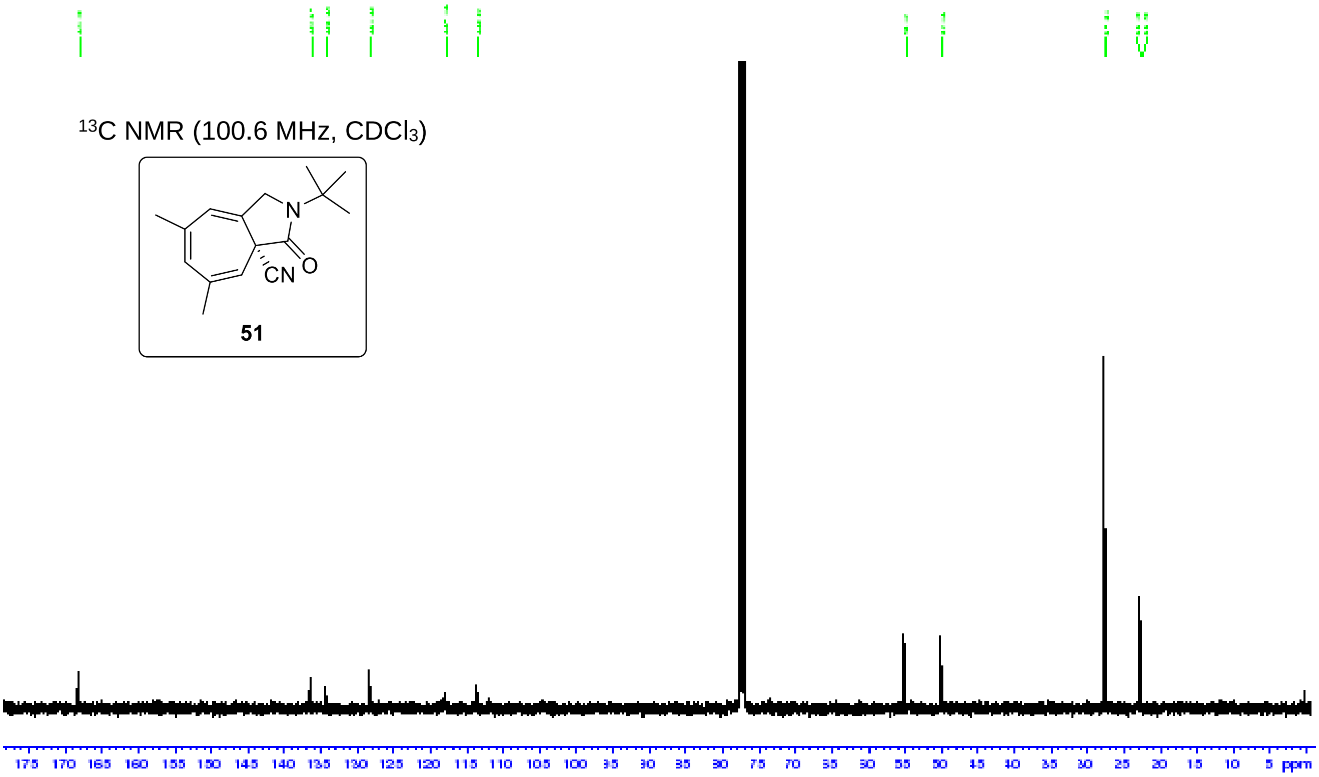

9-Aza-9-*tert*-butyl-1(*S*)-cyano-2,4,6-trimethylbicyclo[5.3.0]deca-2,4,6-trien-10-one (52)

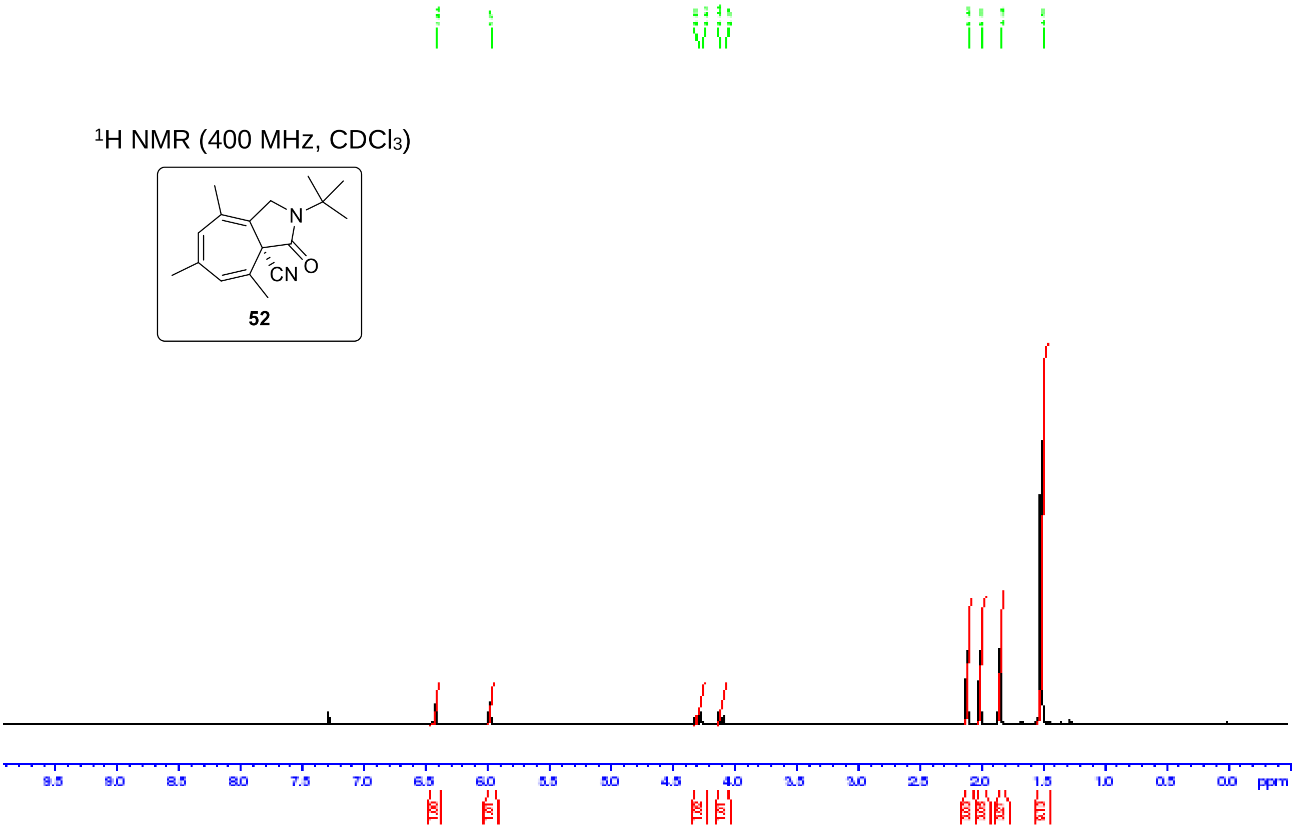

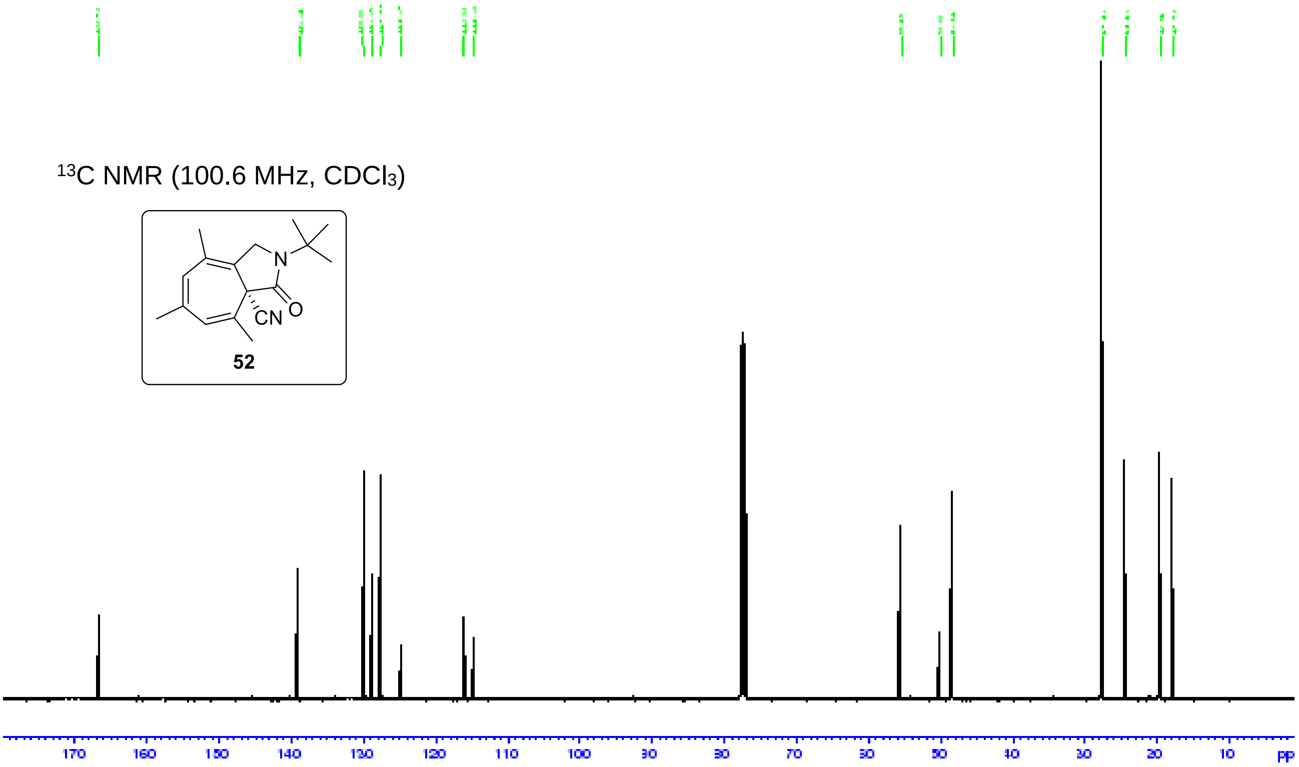

9-Aza-9-*tert*-butyl-1(*S*)-cyanobicyclo[5.3.0]deca-2,4,6-trien-10-one (53)

^{1}H NMR (400 MHz, $CDCl_3$)

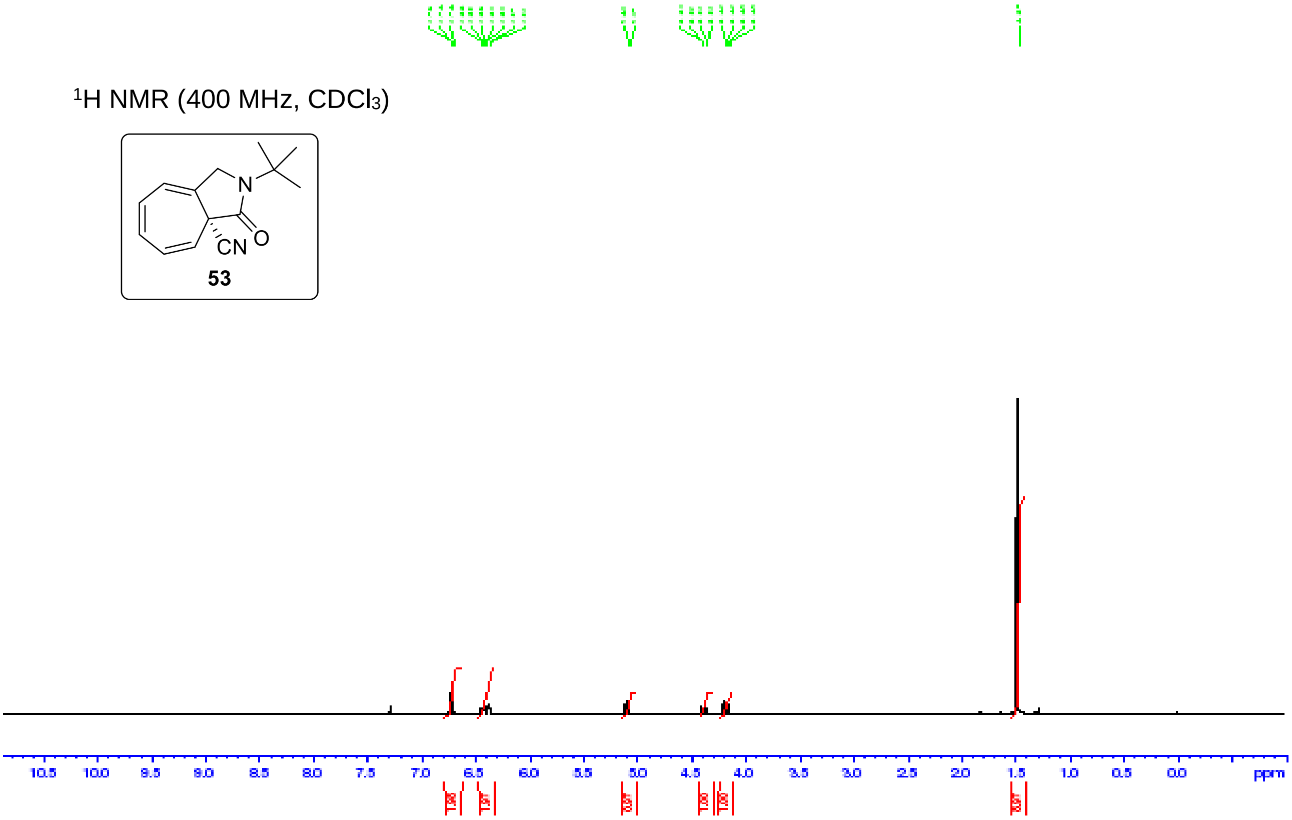

^{13}C NMR (100.6 MHz, $CDCl_3$)

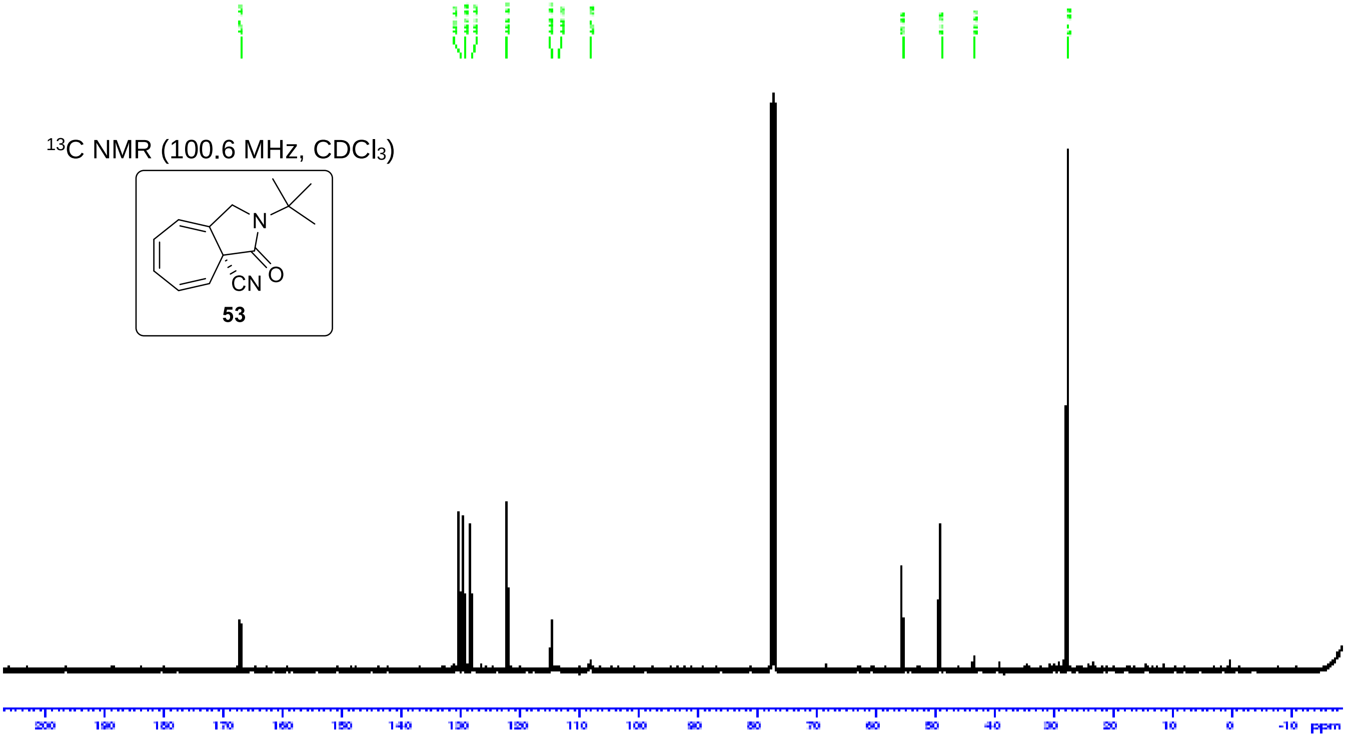

9-Aza-9-*tert*-butyl-1(*R*)-cyano-4-nitrobicyclo[5.3.0]deca-2,4,6-trien-10-one (54)

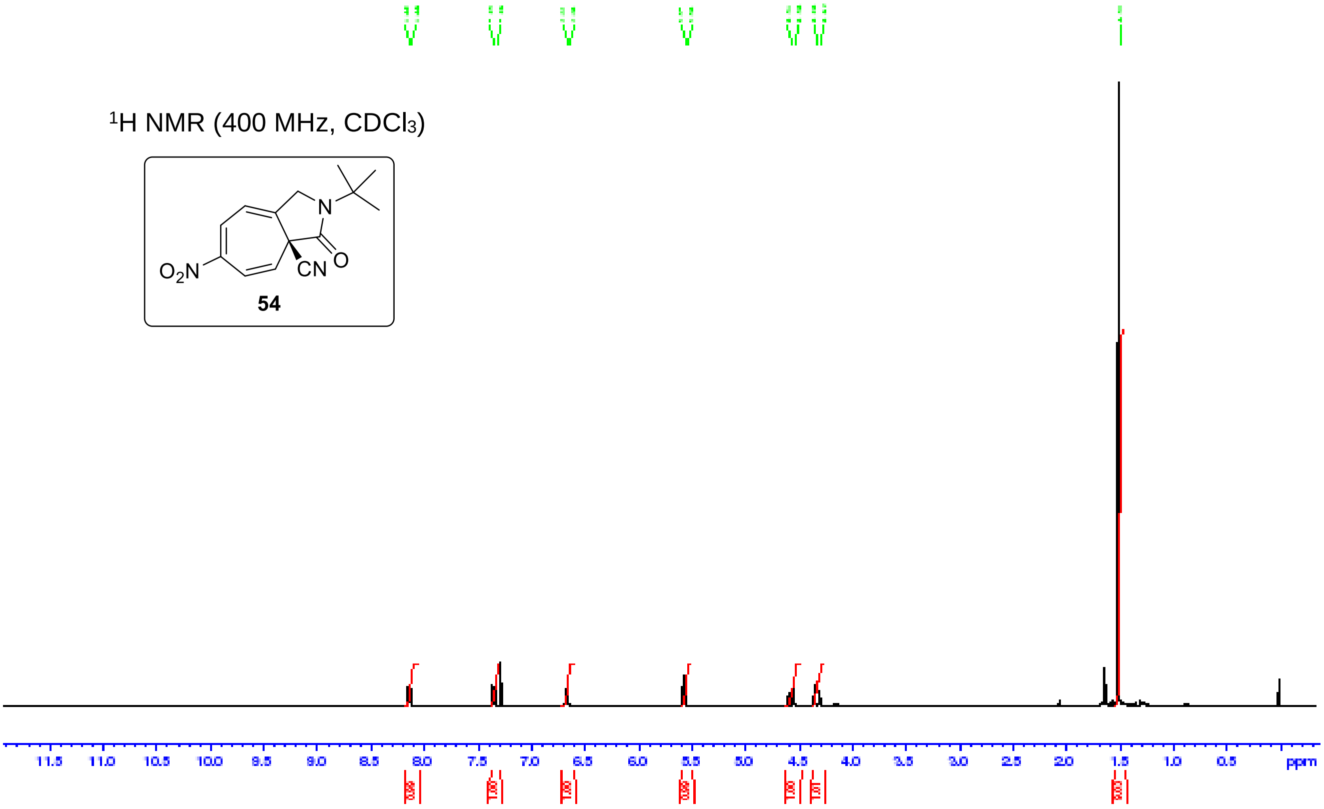

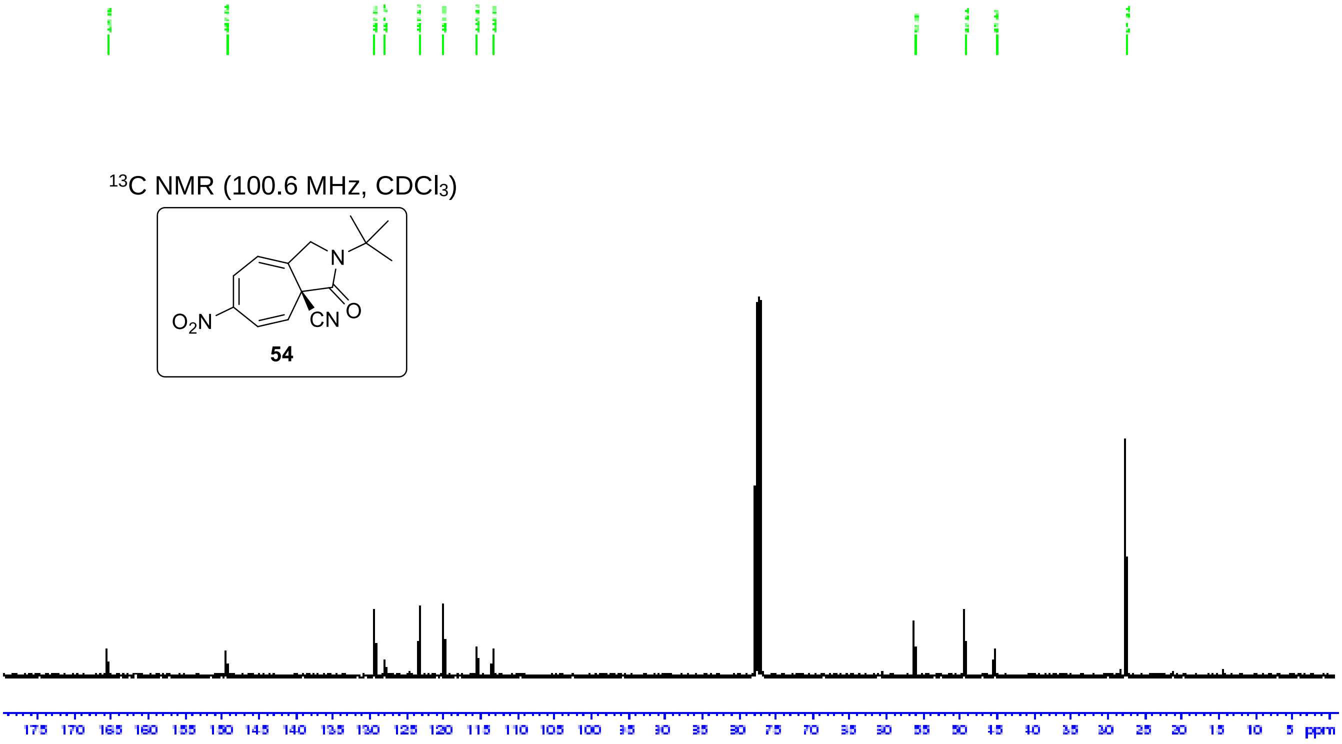

9-Aza-9-*tert*-butyl-1(*S*)-cyano-4-methoxycarbonylbicyclo[5.3.0]deca-2,4,6-trien-10-one (55)

^{1}H NMR (400 MHz, $CDCl_3$)

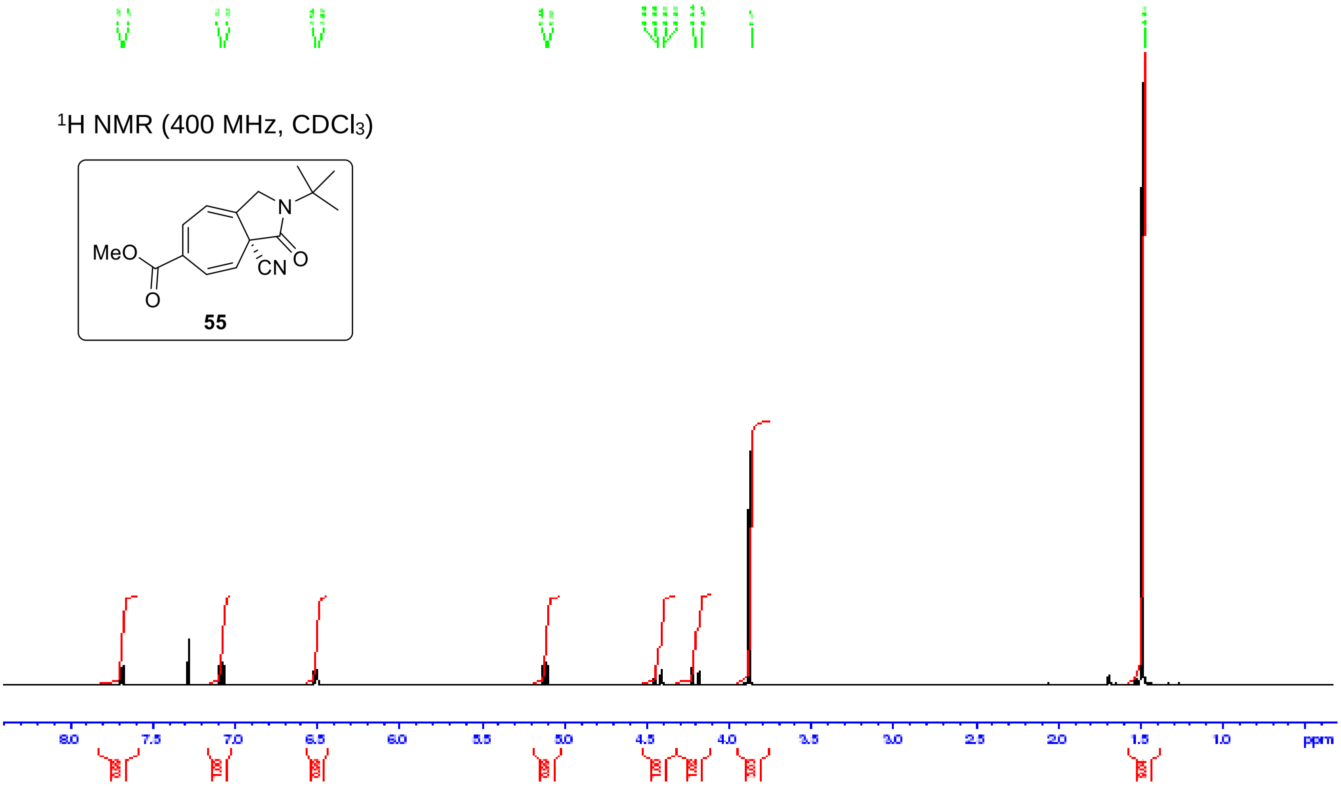

^{13}C NMR (100.6 MHz, $CDCl_3$)

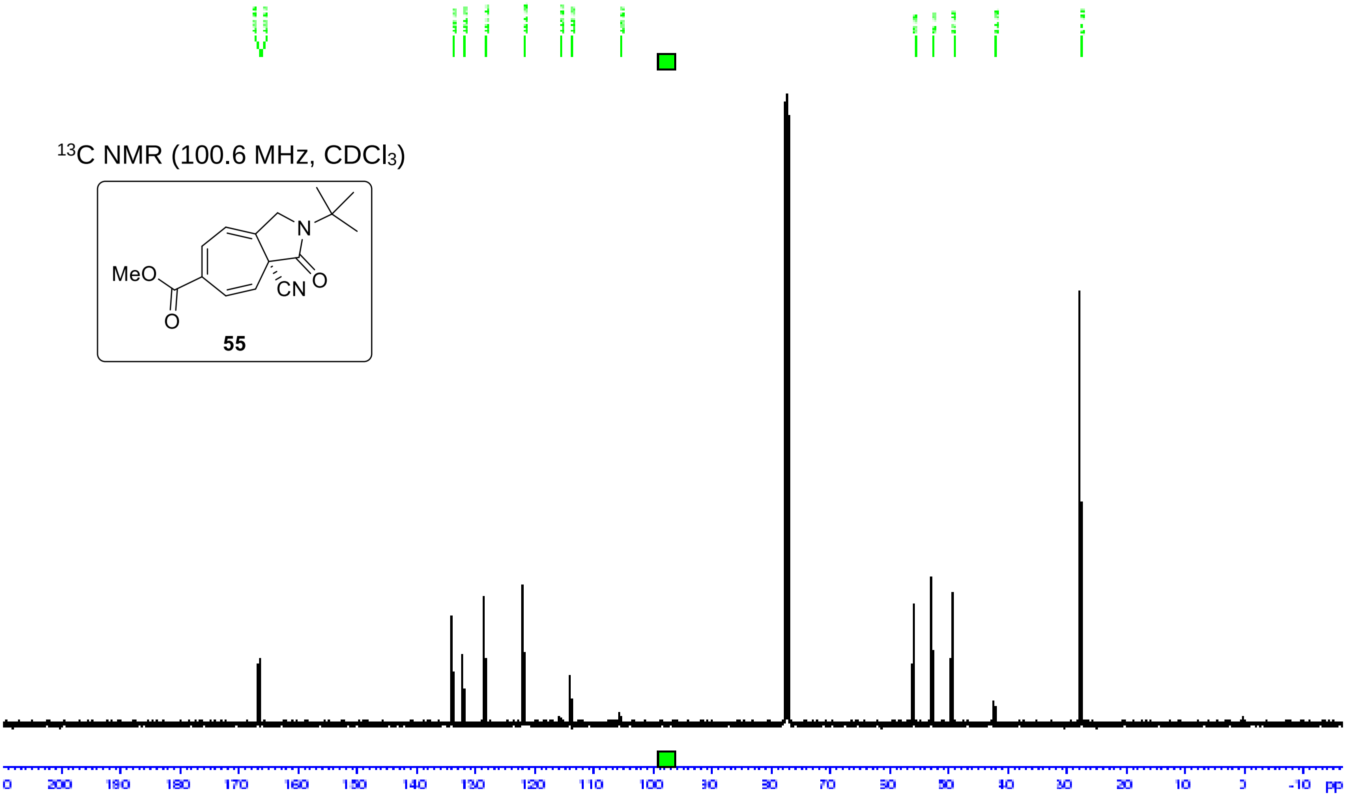

9-Aza-9-*tert*-butyl-1(*R*)-cyano-4-fluorobicyclo[5.3.0]deca-2,4,6-trien-10-one (56)

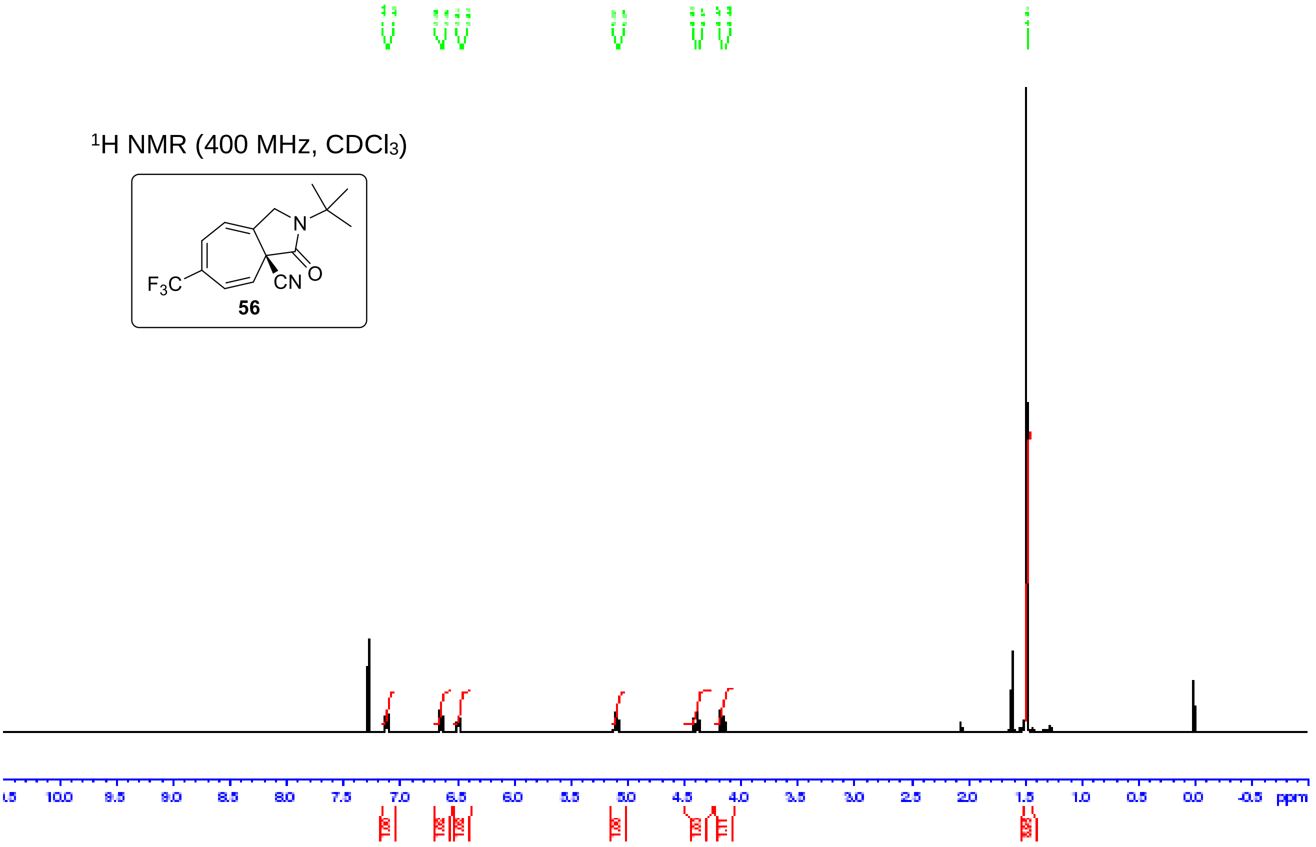

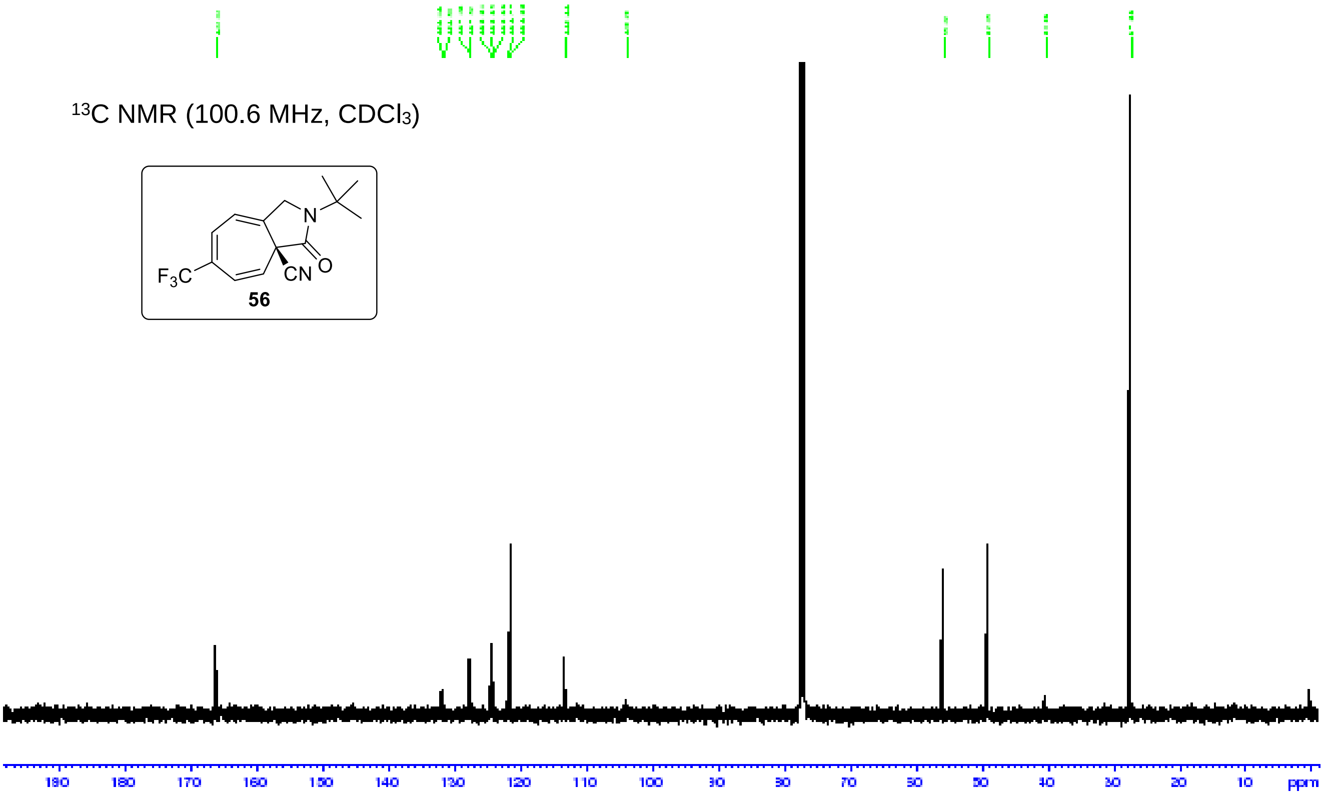

^{19}F NMR (376.5 MHz, $CDCl_3$)

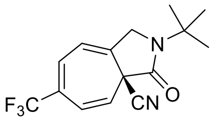

56

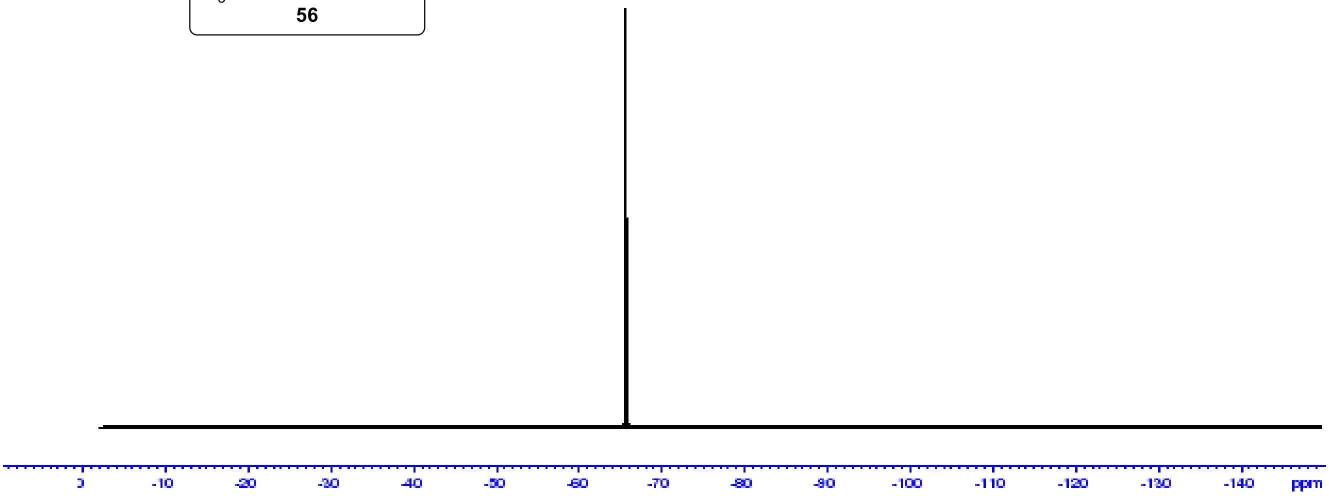

9-Aza-9-*tert*-butyl-1(*S*),4-dicyanobicyclo[5.3.0]deca-2,4,6-trien-10-one (57)

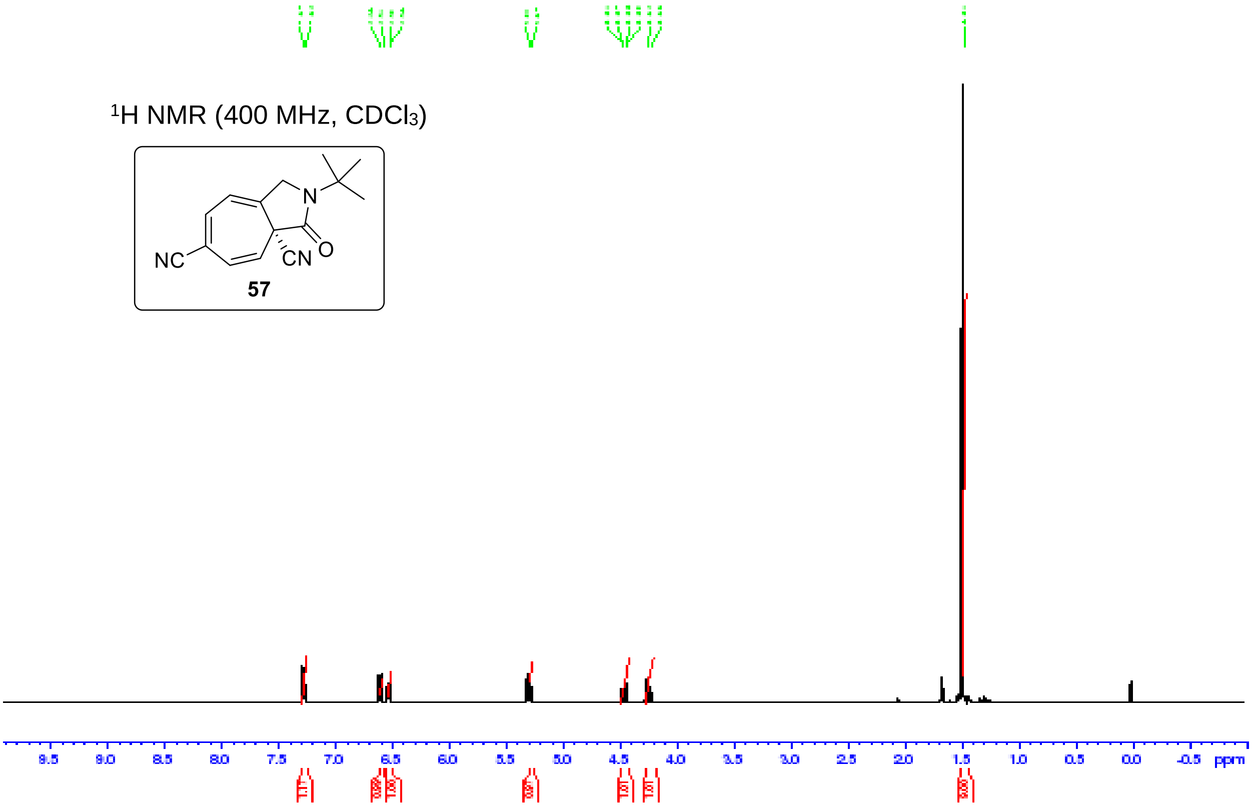

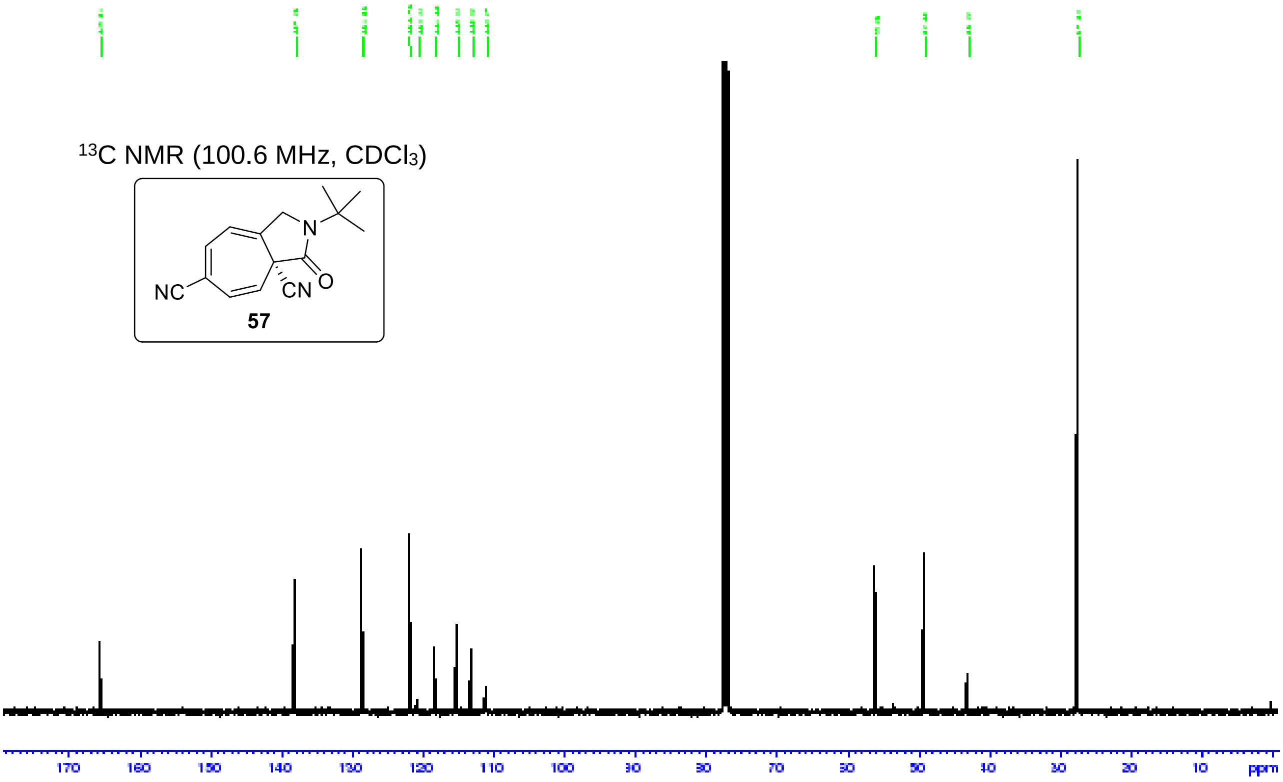

9-Aza-9-*tert*-butyl-1(*S*)-cyano-4-methoxybicyclo[5.3.0]deca-2,4,6-trien-10-one (58)

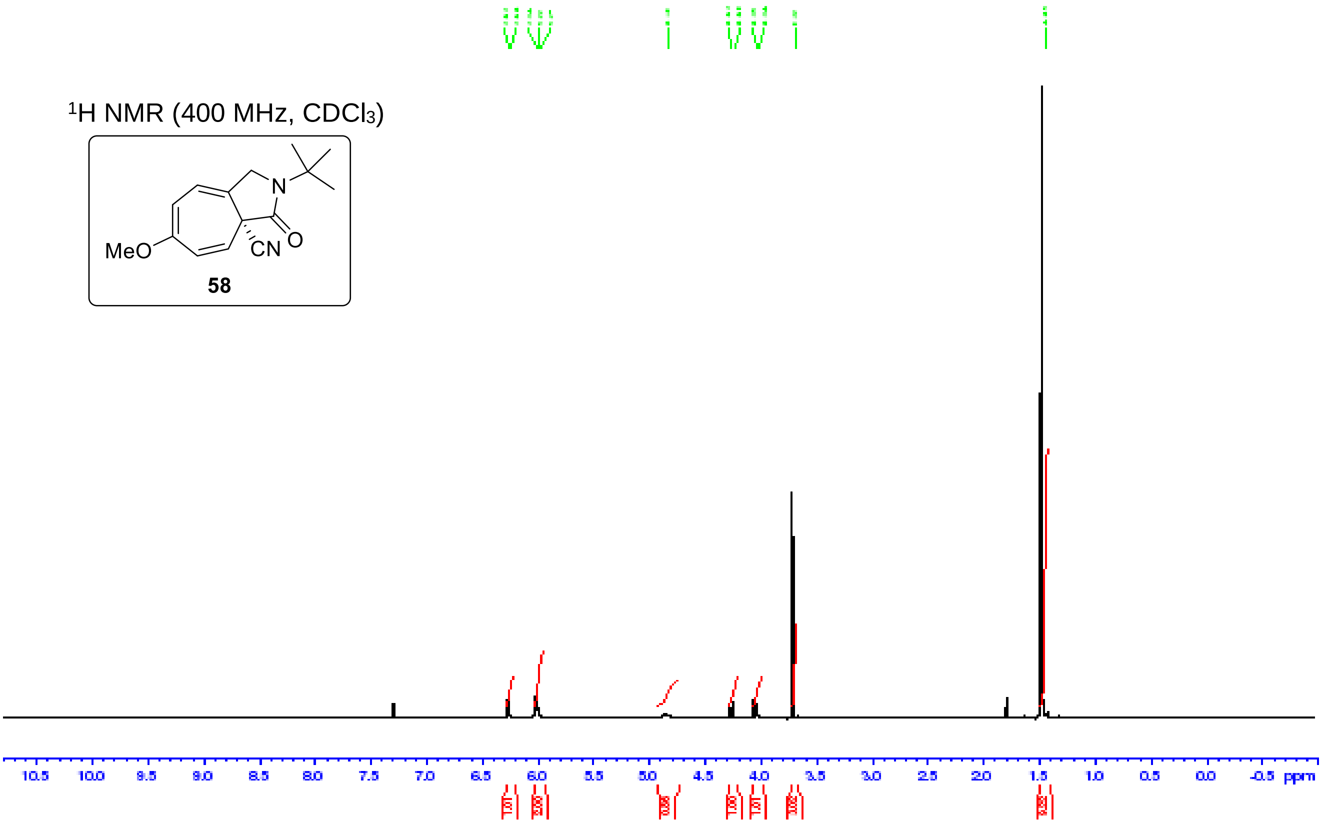

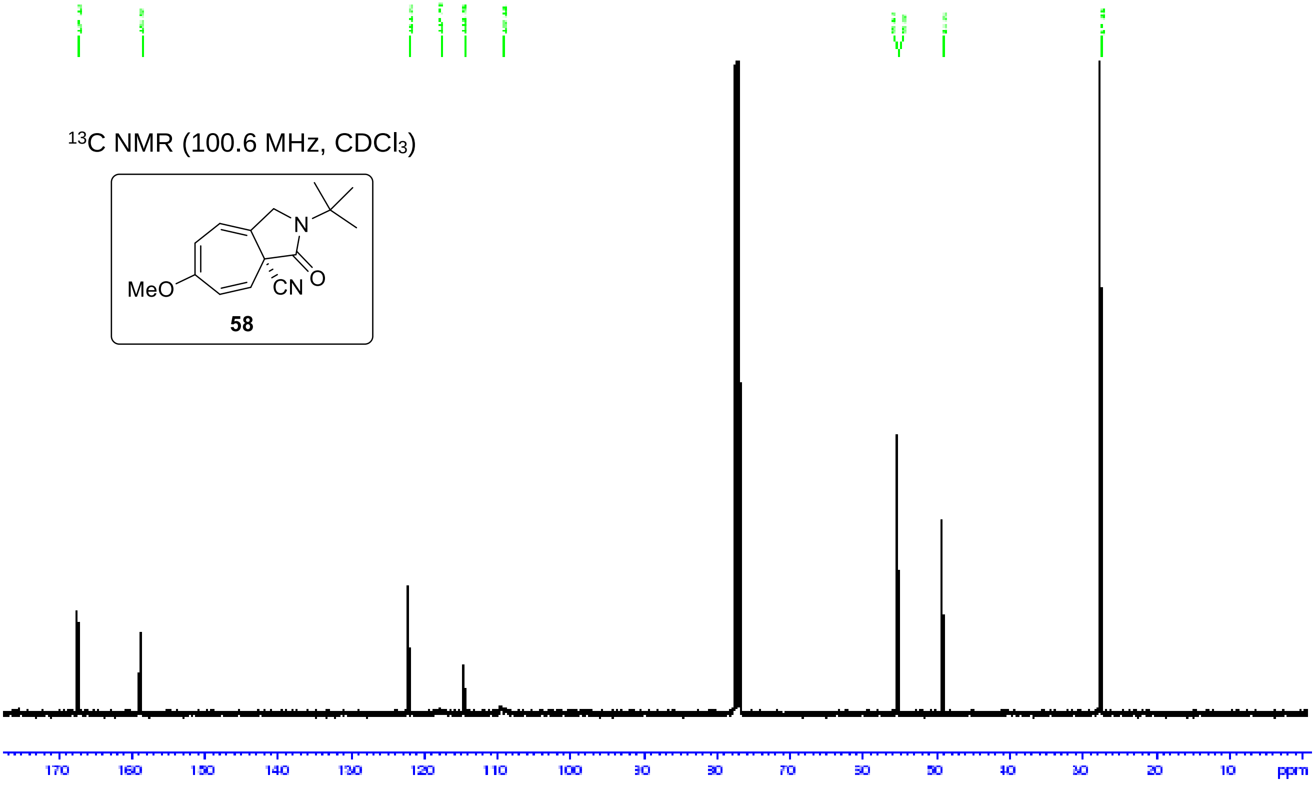

2-Benzyl-3,6,8-trioxo-7-phenyl-2,3,3b,4,7,8-hexahydro-6H,10H-4,10-ethenopyrrolo[3',4':1,3]cyclopropa[1,2-d][1,2,4]triazolo[1,2-a]pyridazine-3a(1H)-carbonitrile (59)

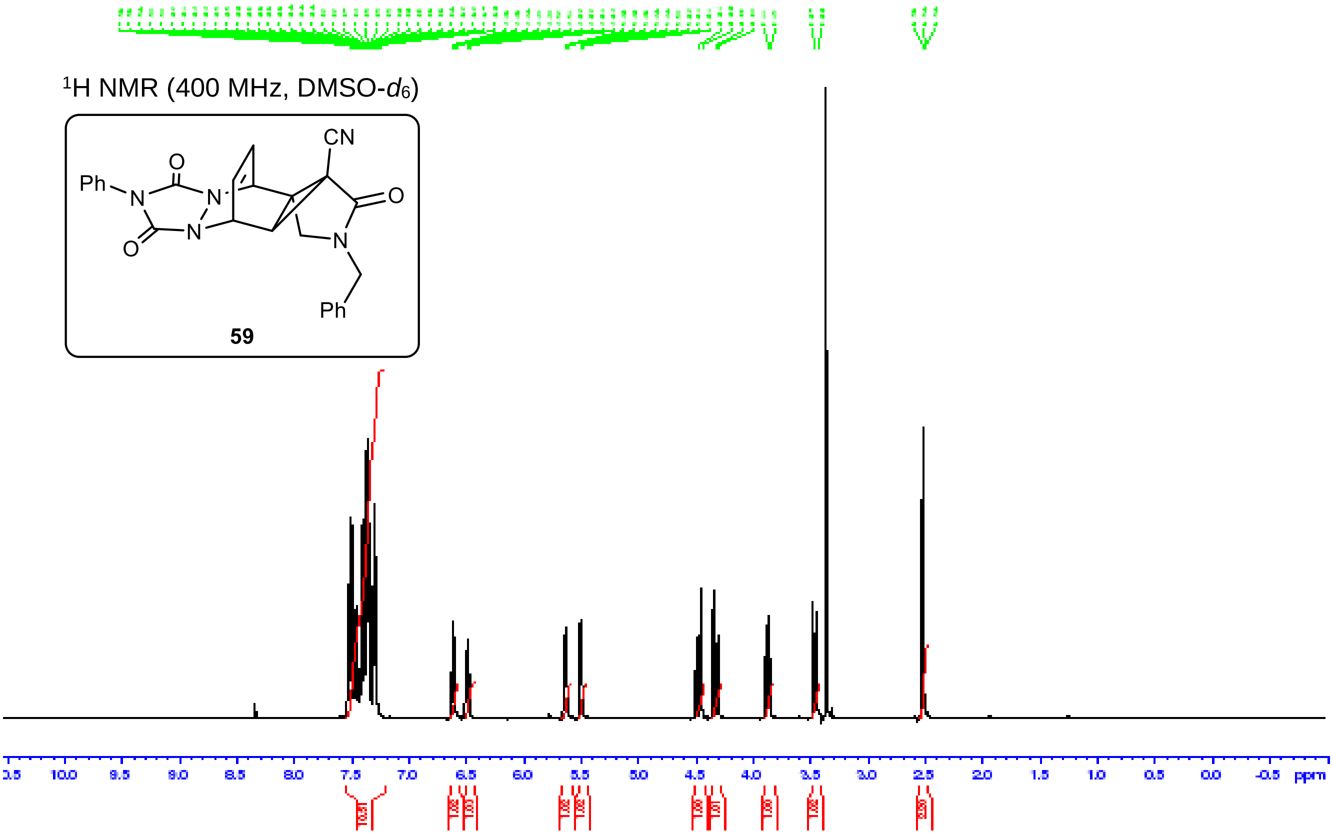

9-Aza-9-*tert*-butyl-1-cyano-4-(4-methylphenyl)bicyclo[5.3.0]deca-2,4,6-trien-10-one (60)

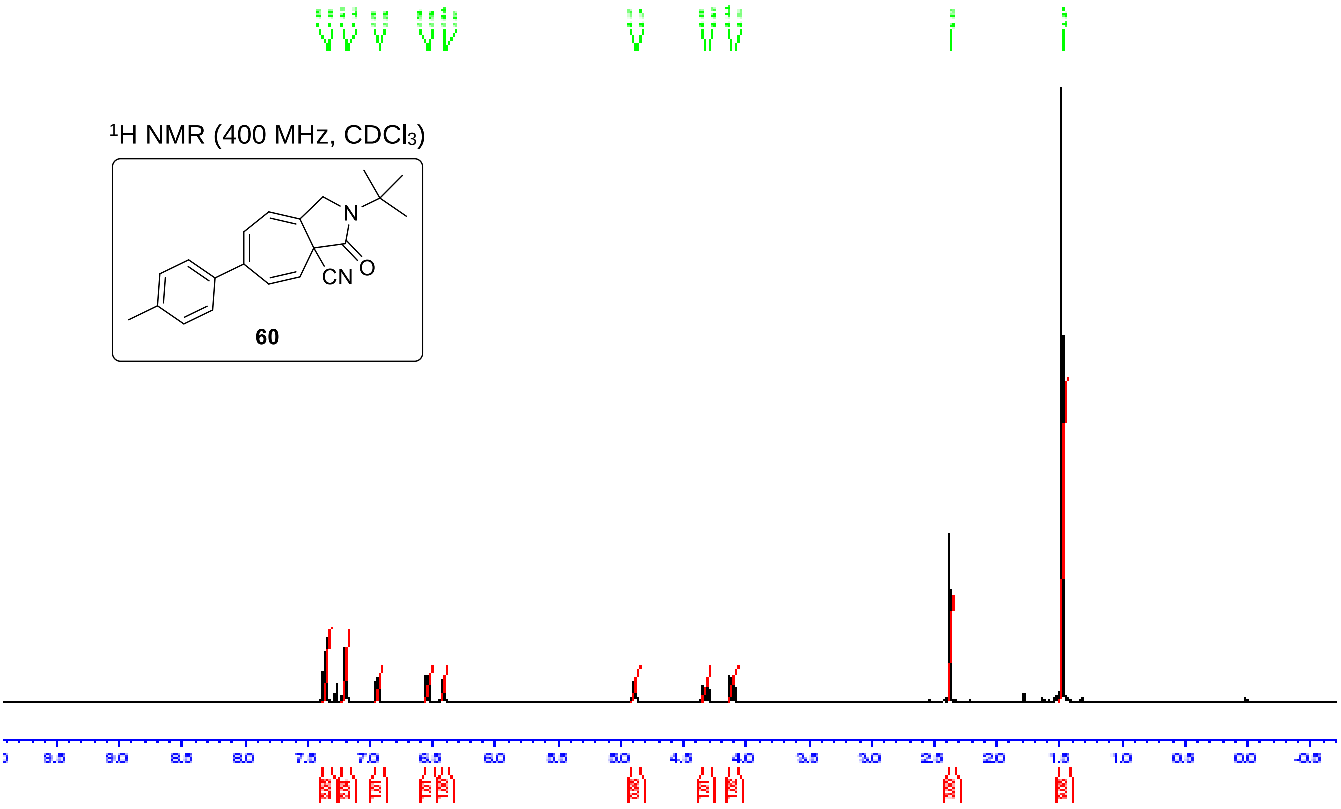

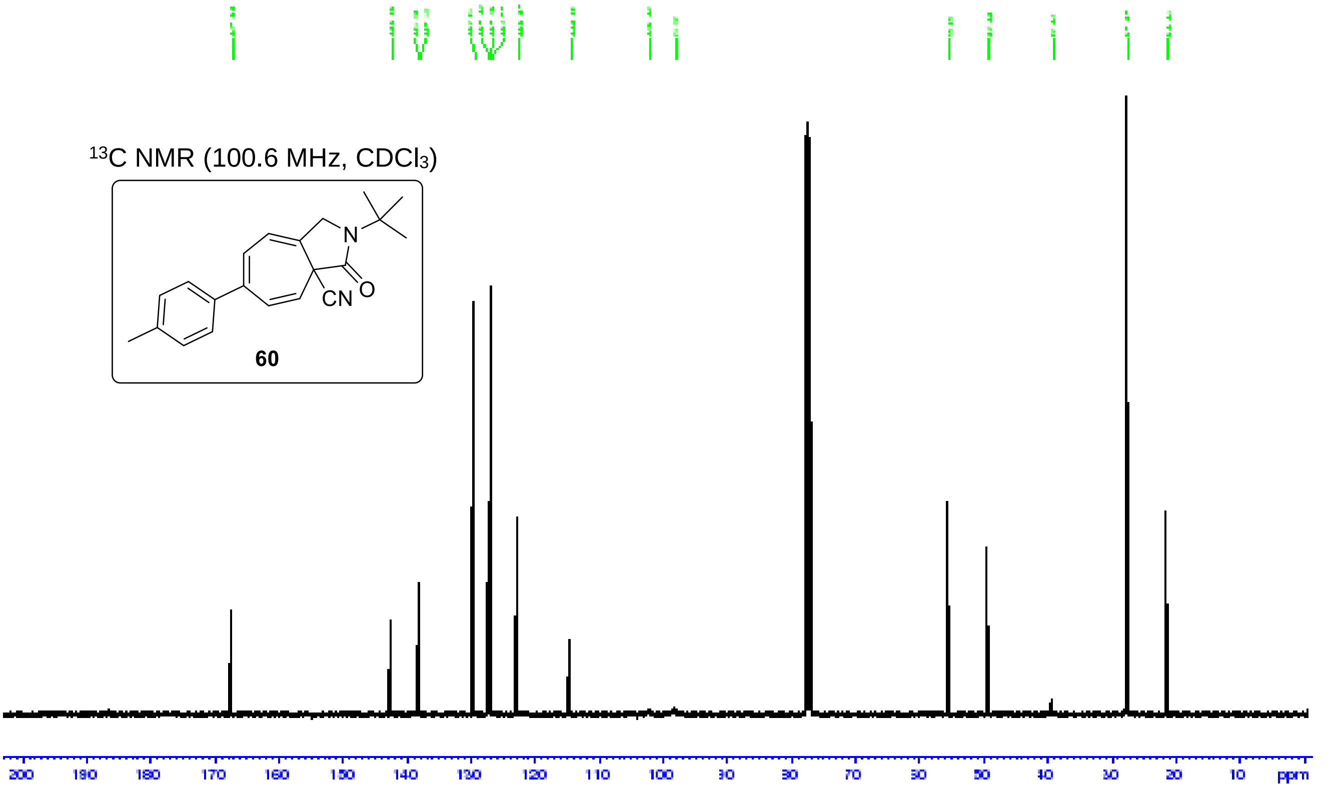

Methyl-9-aza-9-*tert*-butyl-4-methylbicyclo[5.3.0]deca-2,4,6-trien-10-one-1(*R*)-carboxylate (61)

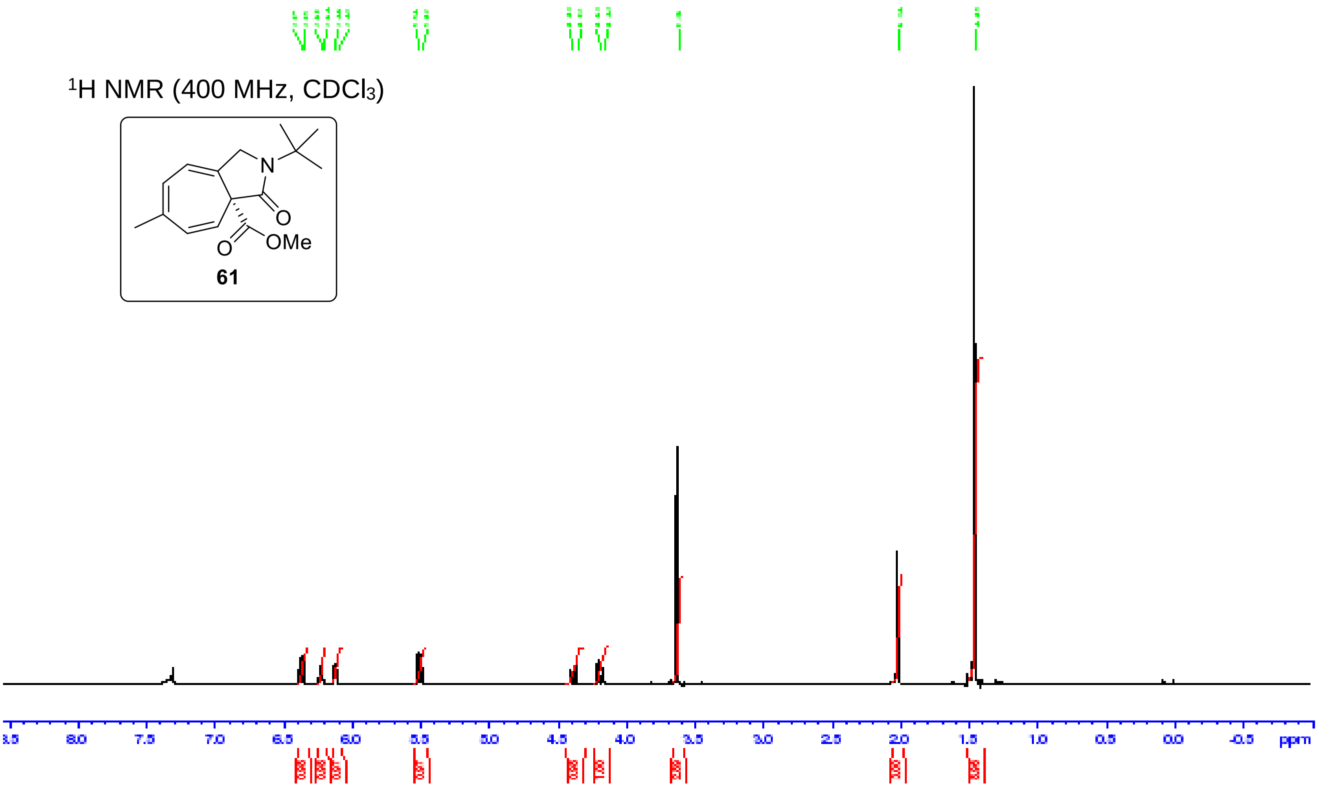

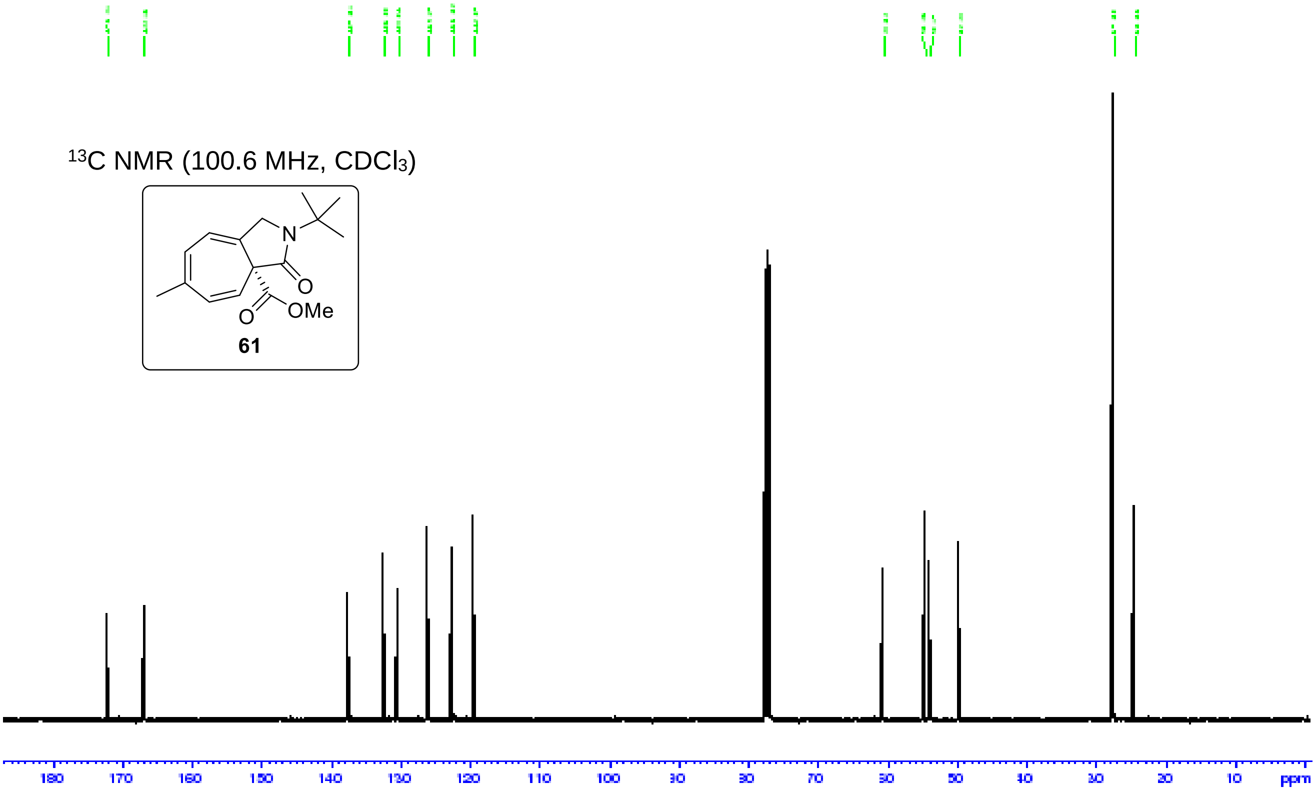

Methyl 2-diazo-3-(2-allyloxyphenyl)-3-oxopropionate[23] (62)

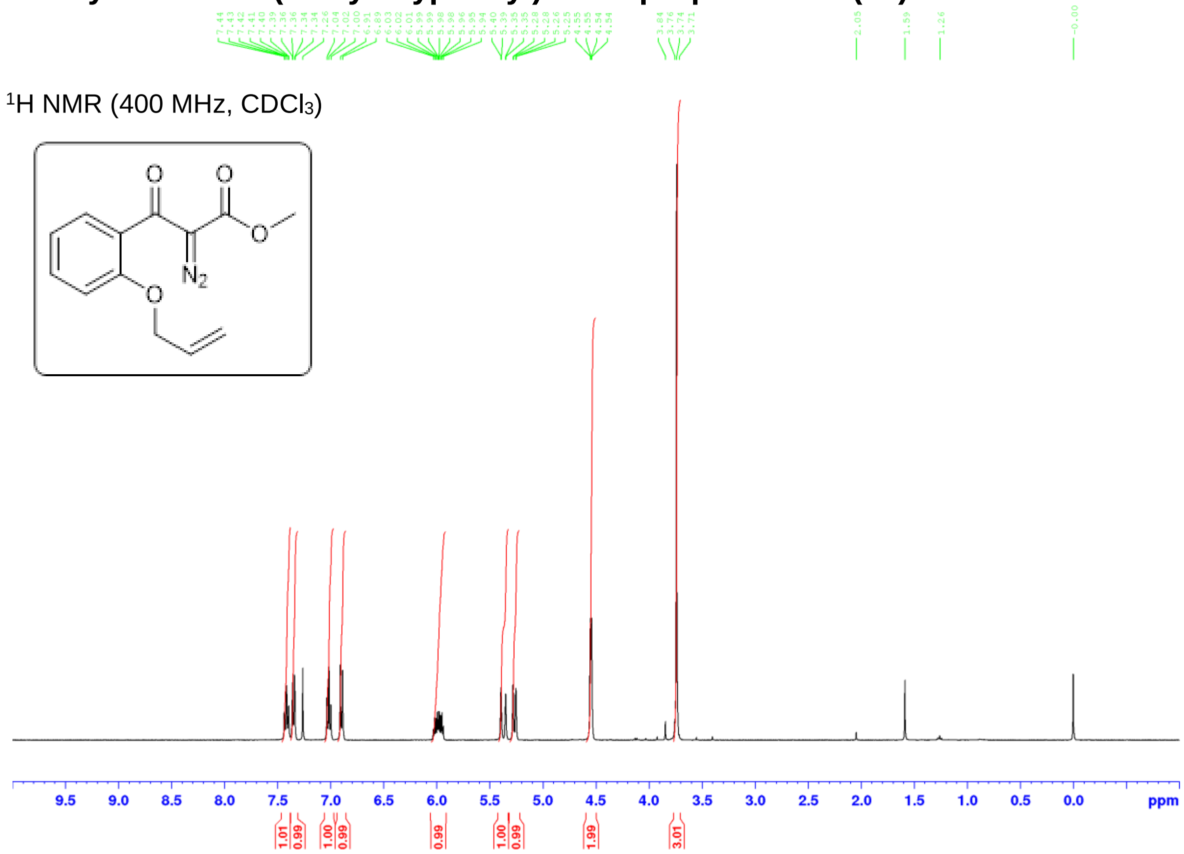

2-Allyl-2-methoxycarbonyl-2,3-dihydrobenzofuran-3-one[24] (63)

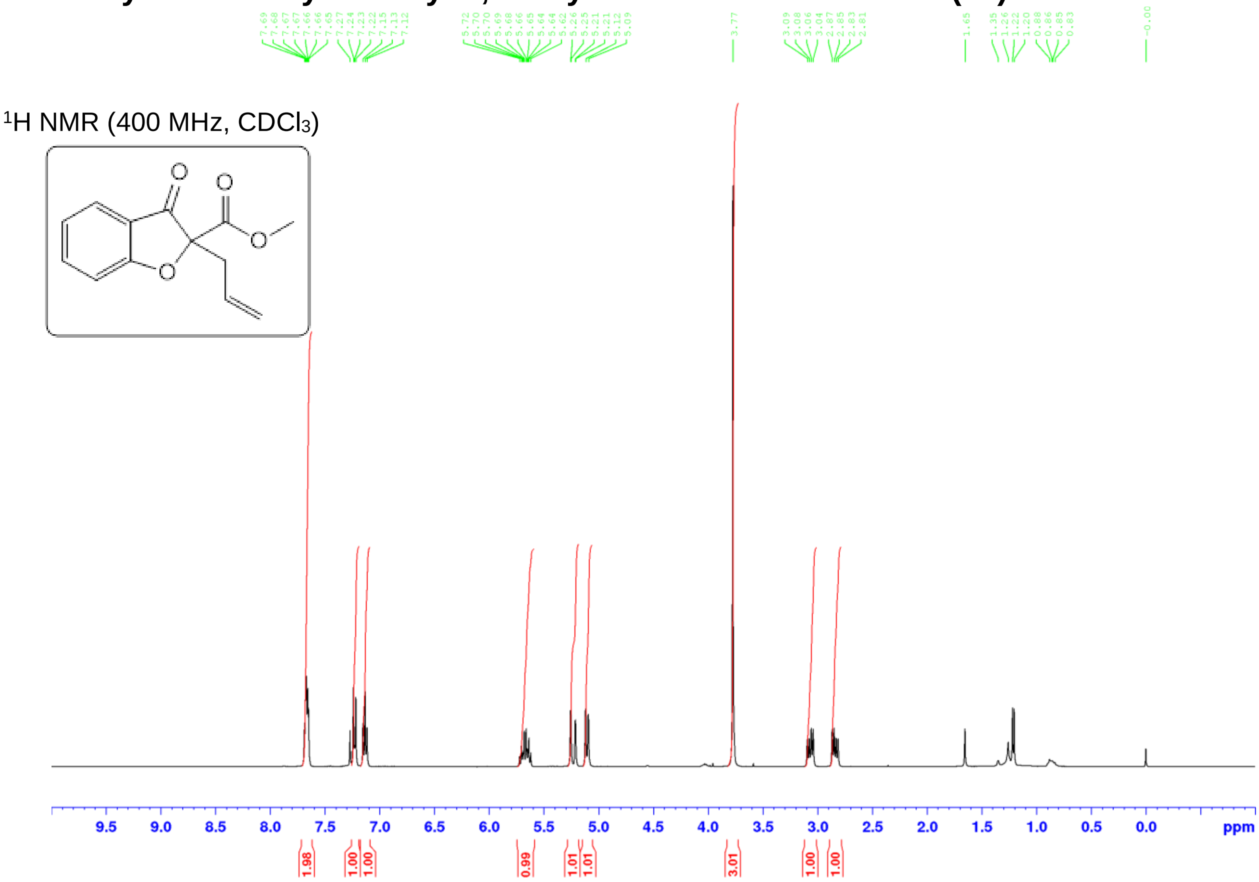

9. HPLC Chromatograms

Table SI.7 HPLC Chromatograms

Compound	Compound Number	Column	λ max (cm^{-1})	Mobile Phase (Hexane: IPA)	Temp (°C)	Flow Rate (mL/min)	Retention Time	
							Enantiomer	Min
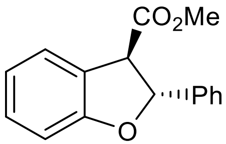	**11a**	Phenomenex Lux® 3*μ*m Amylose-1	210	99 : 1	25	1.0	(+)-(2*S*,3*S*)	11
							(−)-(2*R*,3*R*)	12
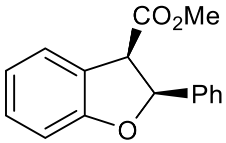	**11b**	Phenomenex Lux® 3*μ*m Amylose-1	210	99 : 1	25	1.0	(−)-(2*S*,3*R*)	15
							(+)-(2*R*,3*S*)	17
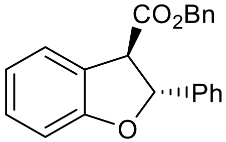	**14a**	Phenomenex Lux® 3*μ*m Amylose-1	210	99 : 1	25	1.0	(+)-(2*S*,3*S*)	17
							(−)-(2*R*,3*R*)	19
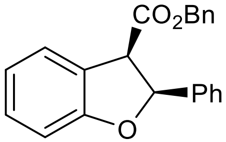	**14b**	Phenomenex Lux® 3*μ*m Amylose-1	210	95 : 5	25	1.0	(−)-(2*S*,3*R*)	12
							(+)-(2*R*,3*S*)	16
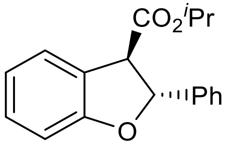	**15a**	Daicel Chiralcel® OJ-H	210	99 : 1	25	1.0	(−)-(2*R*,3*R*)	10
							(+)-(2*S*,3*S*)	14
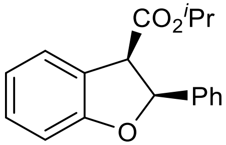	**15b**	Phenomenex Lux® 3*μ*m Amylose-1	210	95 : 5	25	1.0	(−)-(2*S*,3*R*)	6.5
							(+)-(2*R*,3*S*)	6.7
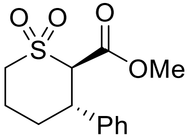	**17a**	Phenomenex Lux® 3*μ*m Amylose-1	209.8	90 : 10	25	1.0	(−)-(2*R*,3*S*)	35
							(+)-(2*S*,3*R*)	38

Compound	Compound Number	Column	λ max (cm^{-1})	Mobile Phase (Hexane: IPA)	Temp (°C)	Flow Rate (mL/min)	Retention Time	
							Enantiomer	Min
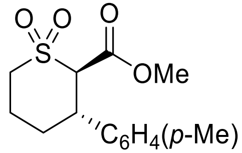	**25a**	Phenomenex Lux® 3μm Amylose-1	212	80 : 20	25	1.0	(−)-(2*R*,3*S*)	14
							(+)-(2*S*,3*R*)	16
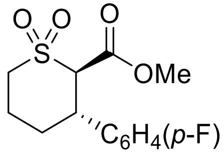	**27a**	Phenomenex Lux® 3μm Amylose-1	209.8	80 : 20	25	1.0	(−)-(2*R*,3*S*)	17
							(+)-(2*S*,3*R*)	20
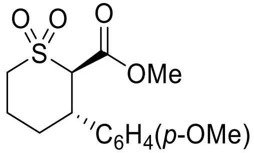	**26a**	Phenomenex Lux® 3μm Amylose-1	226.2	80 : 20	25	1.0	(−)-(2*R*,3*S*)	19
							(+)-(2*S*,3*R*)	23
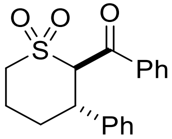	**28a**	Phenomenex Lux® 3μm Amylose-1	209.8	90 : 10	25	1.0	(−)	28
							(+)	41
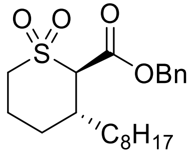	**29a**	Phenomenex Lux® 3μm Amylose-1	209.8	90 : 10	25	1.0	(+)-(2*S*,3*S*)	13
							(−)-(2*R*,3*R*)	18
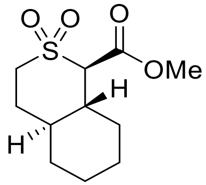	**30a**	Phenomenex Lux® 3μm Amylose-1	216	90 : 10	25	1.0	(+) (1*S*,4a*R*,8a*S*)	19
							(−) (1*R*,4a*S*,8a*R*)	25

Compound	Compound Number	Column	λ max (cm^{-1})	Mobile Phase (Hexane: IPA)	Temp (°C)	Flow Rate (mL/min)	Retention Time	
							Enantiomer	Min
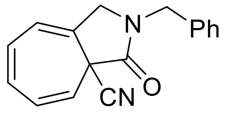	**32**	Phenomenex Lux® 3μm Amylose-1	209.8	85 : 25	25	1.0	(−)-(1*R*)	16
							(+)-(1*S*)	24
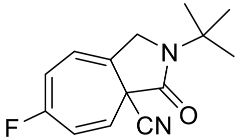	**46**	Phenomenex Lux® 3μm Amylose-1	209.8	85 : 25	25	1.0	(−)-(1*R*)	8
							(+)-(1*S*)	11
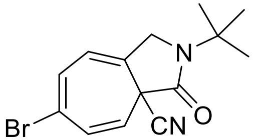	**47**	Phenomenex Lux® 3μm Amylose-1	209.8	85 : 25	25	1.0	(−)-(1*R*)	8
							(+)-(1*S*)	12
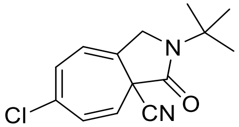	**48**	Phenomenex Lux® 3μm Amylose-1	209.8	85 : 25	25	1.0	(−)-(1*R*)	8
							(+)-(1*S*)	11
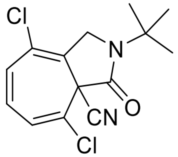	**49**	Phenomenex Lux® 3μm Amylose-1	214.5	95 : 05	25	0.5	(−)-(1*S*)	19
							(+)-(1*R*)	20
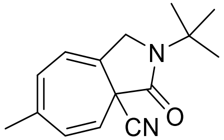	**50**	Phenomenex Lux® 3μm Amylose-1	209.8	85 : 25	25	1.0	(−)-(1*R*)	6
							(+)-(1*S*)	9

Compound	Compound Number	Column	λ max (cm^{-1})	Mobile Phase (Hexane: IPA)	Temp (°C)	Flow Rate (mL/min)	Retention Time Enantiomer	Min
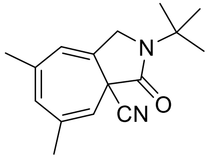	**51**	Phenomenex Lux® 3μm Amylose-1	209.8	85 : 25	25	1.0	(−)-(1*R*)	6
							(+)-(1*S*)	7
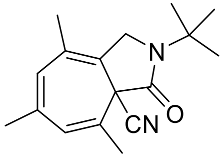	**52**	Phenomenex Lux® 3μm Amylose-1	211.0	90 : 10	25	0.5	(−)-(1*R*)	11
							(+)-(1*S*)	12
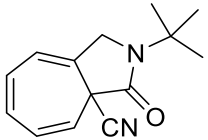	**53**	Phenomenex Lux® 3μm Amylose-1	209.8	85 : 25	25	1.0	(−)-(1*R*)	7
							(+)-(1*S*)	9
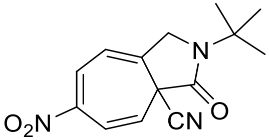	**54**	Phenomenex Lux® 3μm Amylose-1	209.8	85 : 25	25	1.0	(−)-(1*R*)	16
							(+)-(1*S*)	23
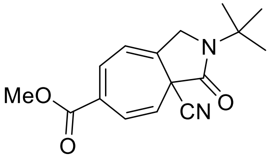	**55**	Phenomenex Lux® 3μm Amylose-1	209.8	85 : 25	25	1.0	(−)-(1*R*)	12
							(+)-(1*S*)	15
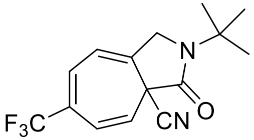	**56**	Phenomenex Lux® 3μm Amylose-1	209.8	85 : 25	25	1.0	(−)-(1*R*)	7
							(+)-(1*S*)	10
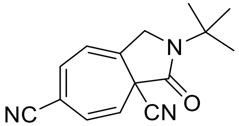	**57**	Phenomenex Lux® 3μm Amylose-1	209.8	90 : 10	25	0.5	(−)-(1*R*)	26
							(+)-(1*S*)	44

Compound	Compound Number	Column	λ max (cm^{-1})	Mobile Phase (Hexane: IPA)	Temp (°C)	Flow Rate (mL/min)	Retention Time	
							**Enantiomer**	**Min**
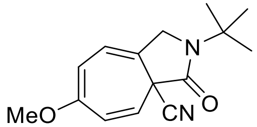	**58**	Phenomenex Lux® 3*μ*m Amylose-1	209.8	85 : 25	25	1.0	(−)-(1*R*)	8
							(+)-(1*S*)	11
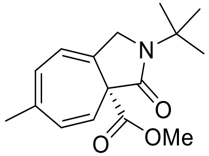	**61**	Phenomenex Lux® 3*μ*m Amylose-1	209.8	85 : 25	25	1.0	(+)-(1*R*)	30
							(−)-(1*S*)	32
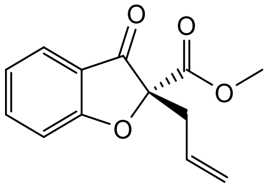	**63**	Phenomenex Lux® 3*μ*m Amylose-1	210	99 : 1	25	0.5	2*S*	29
							2*R*	31

(2*R**,3*R**)-trans-3-Methoxycarbonyl-2-phenyl-2,3-dihydrobenzofuran (11a)

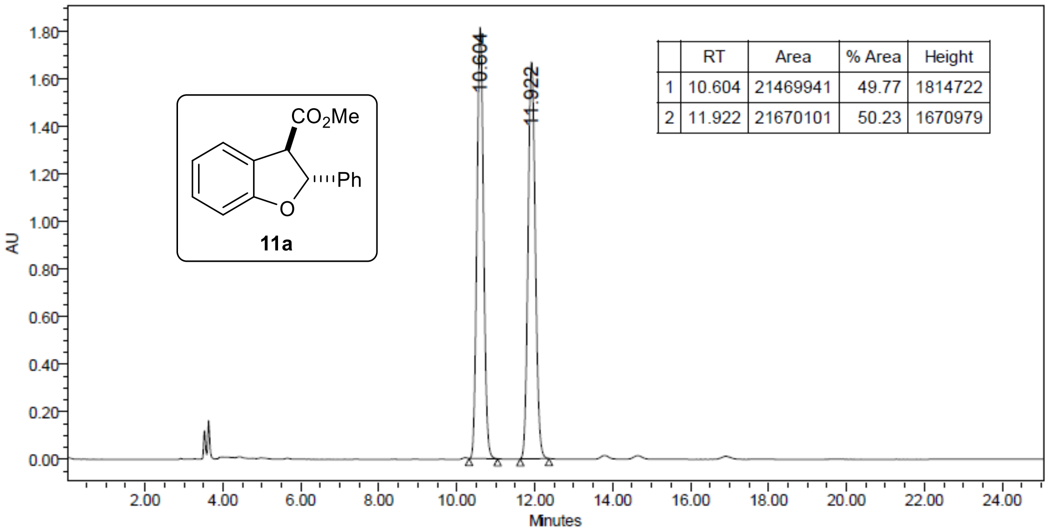

Isolated from the reaction of* ***10* *in the presence of* $Rh_2(OAc)_4$.

(2*R*,3*R*)-2,3-trans-3-Methoxycarbonyl-2-phenyl-2,3-dihydrobenzofuran (11a)

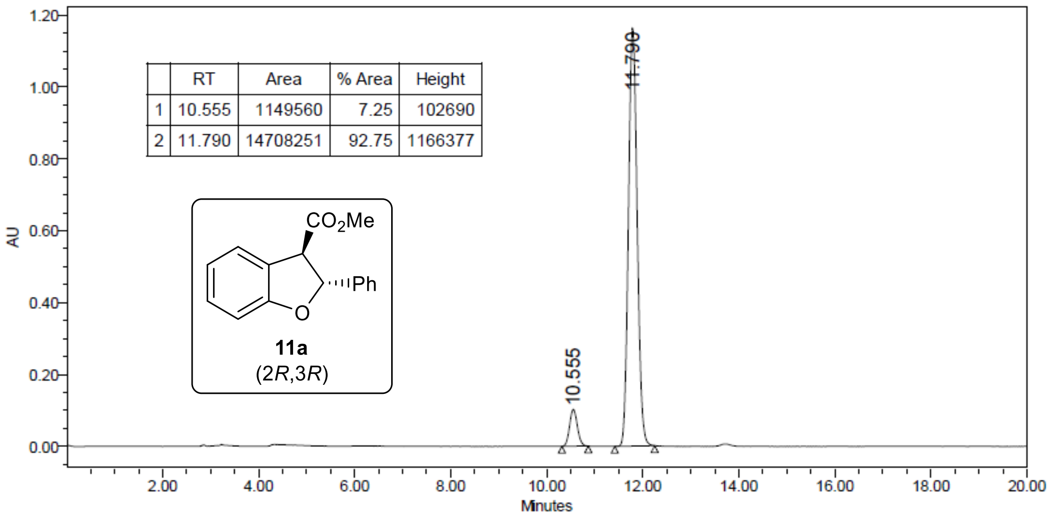

Isolated from the reaction of* ***10* *in the presence of* $Rh_2(2S\text{-}M\text{-}2'\text{-}NA)_4$ ***9g*** *(**Table 1**, entry 9).*

Compound	Column	λ max (nm)	Mobile Phase (Hex:IPA)	Temp (°C)	Flow (mL/min)	Retention Time	
						Enantiomer	min
**11a**	Phenomenex Lux® 3μm Amylose-1	210	99:1	25	1.0	(+) (2*S*,3*S*)	11
						(−) (2*R*,3*R*)	12

(2*S**,3*R**)-cis-3-Methoxycarbonyl-2-phenyl-2,3-dihydrobenzofuran (11b)

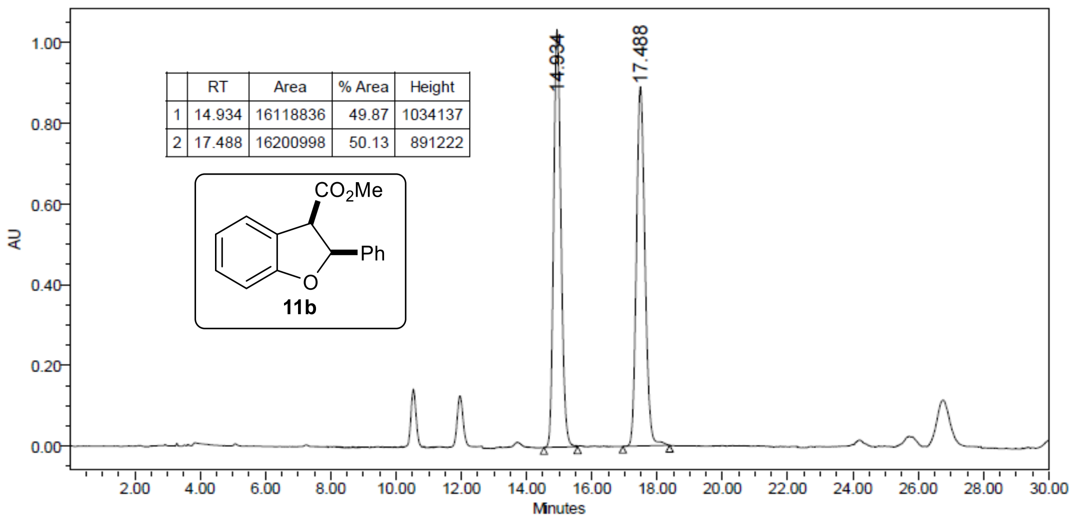

*Isolated from the reaction of **10** in the presence of $Rh_2(OAc)_4$.*

(2*S*,3*R*)-2,3-cis-3-Methoxycarbonyl-2-phenyl-2,3-dihydrobenzofuran (11b)

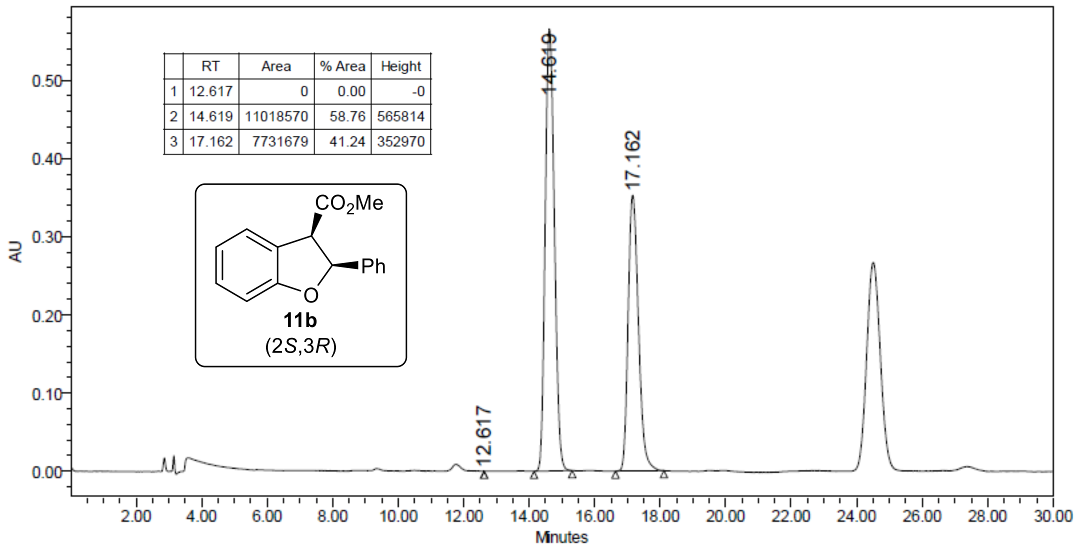

*Isolated from the reaction of **10** in the presence of Rh2(2S-M-2′-NA)4 **9g** (**Table 1**, entry 9).*

Compound	Column	λ max (nm)	Mobile Phase (Hex:IPA)	Temp (°C)	Flow (mL/min)	Retention Time	
						Enantiomer	min
**11b**	Phenomenex Lux® 3μm Amylose-1	210	99:1	25	1.0	(−) (2*S*,3*R*)	15
						(+) (2*R*,3*S*)	17

(2*R**,3*R**)-trans-3-Benzyloxycarbonyl-2-phenyl-2,3-dihydrobenzofuran (14a)

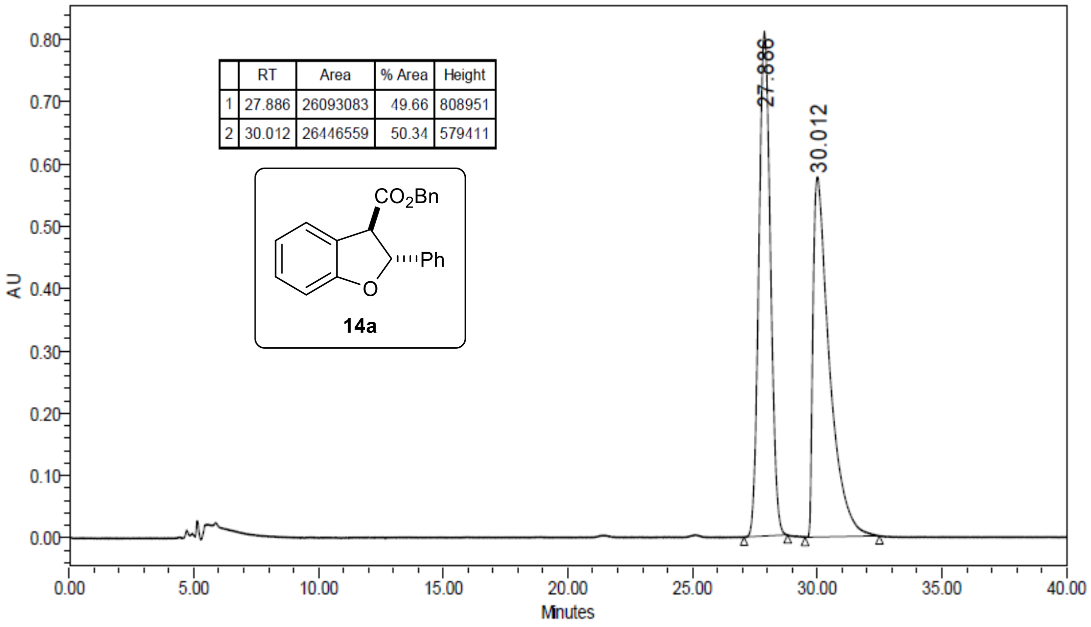

Isolated from the reaction of* ***12* *in the presence of $Rh_2(OAc)_4$.*

(2*R*,3*R*)-2,3-trans-3-Benzyloxycarbonyl-2-phenyl-2,3-dihydrobenzofuran (14a)

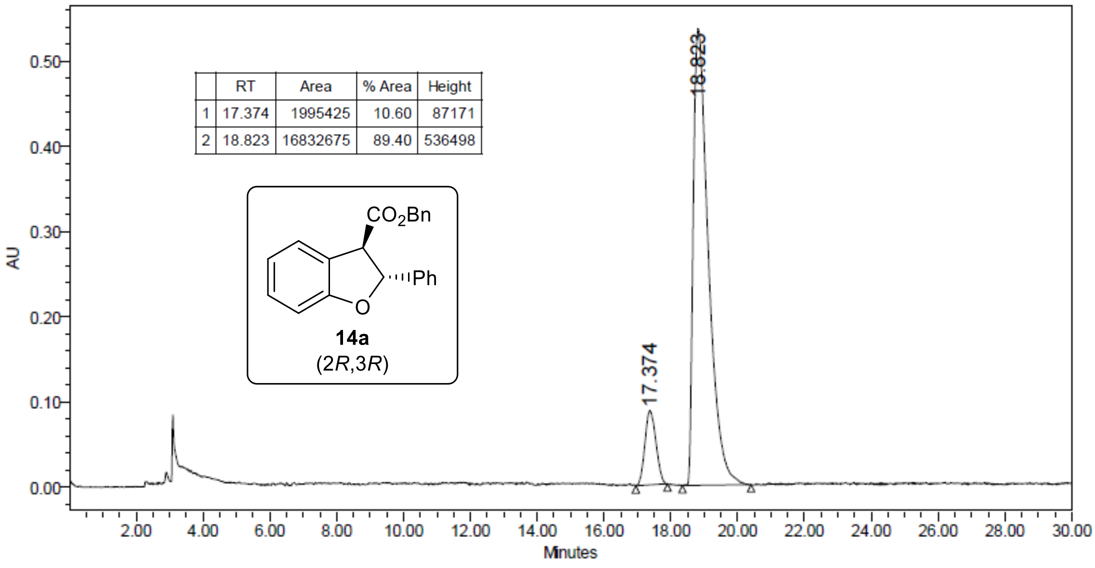

Isolated from the reaction of* ***12* *in the presence of $Rh_2(2S\text{-}MPA)_4$* ***9f*** *(****Table 2****, entry 14).*

Compound	Column	λ max (nm)	Mobile Phase (Hex:IPA)	Temp (°C)	Flow (mL/min)	Retention Time	
						Enantiomer	min
**14a**	Phenomenex Lux® 3*μ*m Amylose-1	210	99:1	25	1.0	(+) (2*S*,3*S*)	17
						(−) (2*R*,3*R*)	19

(2*S*,3*R***)-cis-3-Benzyloxycarbonyl-2-phenyl-2,3-dihydrobenzofuran (14b)**

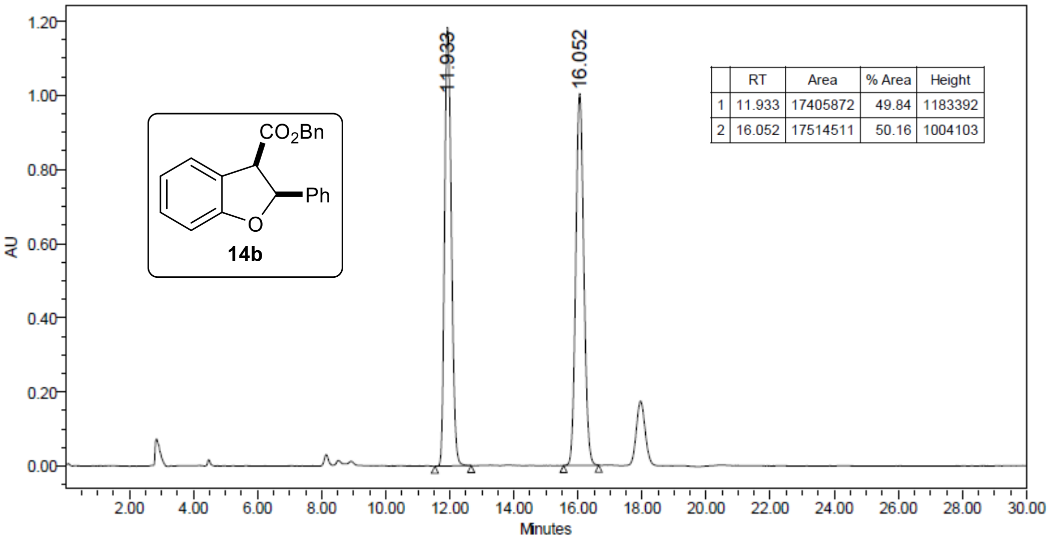

*Isolated from the reaction of **12** in the presence of $Rh_2(OAc)_4$.

(2*S*,3*R*)-2,3-cis-3-Benzyloxycarbonyl-2-phenyl-2,3-dihydrobenzofuran (14b)

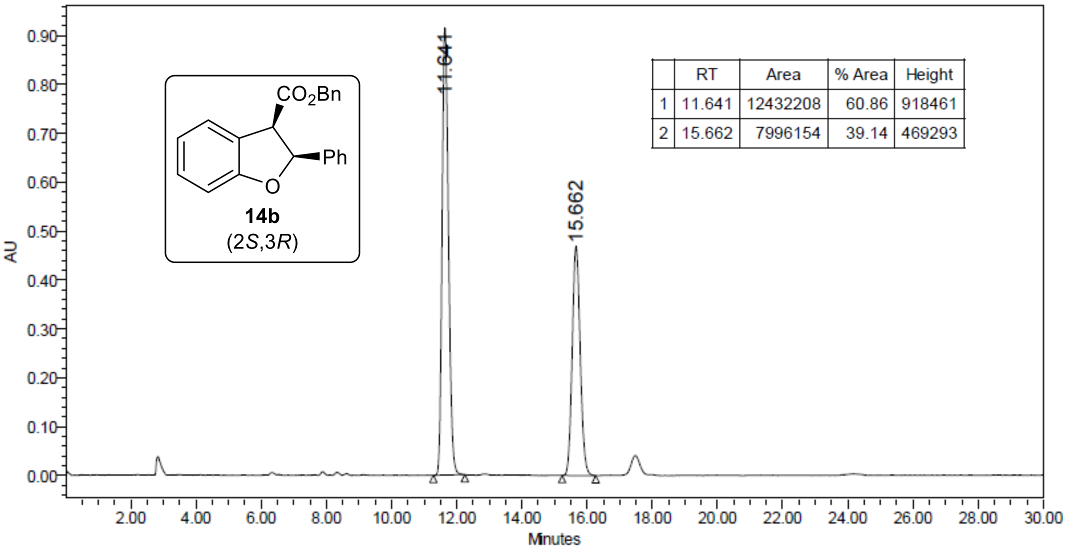

*Isolated from the reaction of **12** in the presence of $Rh_2(2S\text{-}MPA)_4$ ***9f*** (**Table 2**, entry 14).

Compound	Column	λ max (nm)	Mobile Phase (Hex:IPA)	Temp (°C)	Flow (mL/min)	Retention Time	
						**Enantiomer**	**min**
**14b**	Phenomenex Lux® 3μm Amylose-1	210	95:5	25	1.0	(−) (2*S*,3*R*)	12
						(+) (2*R*,3*S*)	16

(2*R,3*R**)-trans-3-Isopropyloxycarbonyl-2-phenyl-2,3-dihydrobenzofuran (15a)**

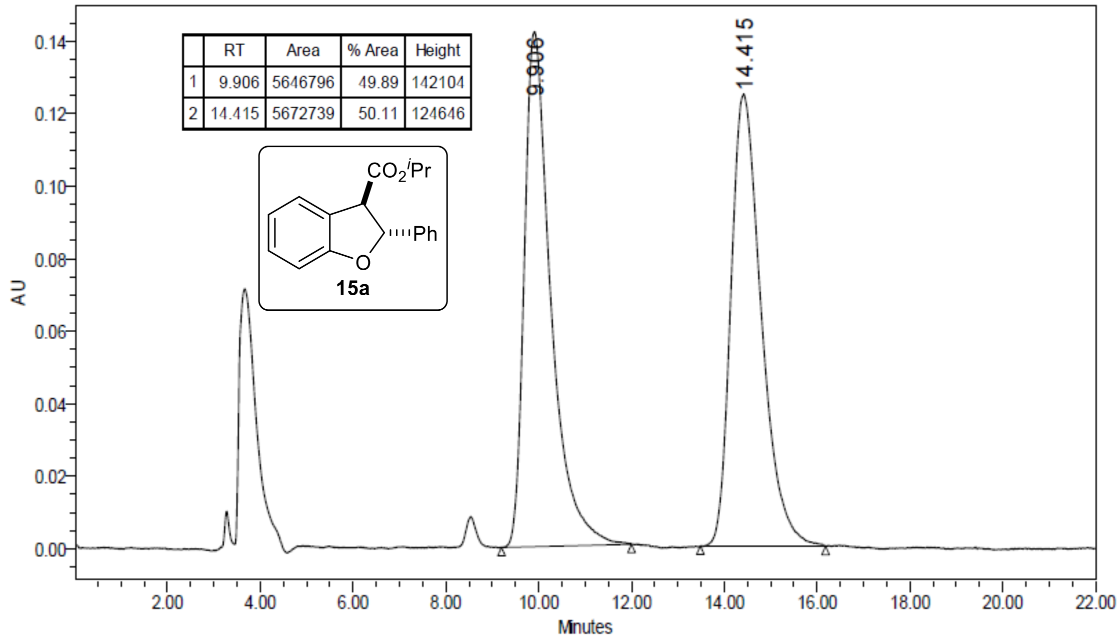

Isolated from the reaction of **13 in the presence of $Rh_2(OAc)_4$.*

(2*R*,3*R*)-2,3-trans-3-Isopropyloxycarbonyl-2-phenyl-2,3-dihydrobenzofuran (15a)

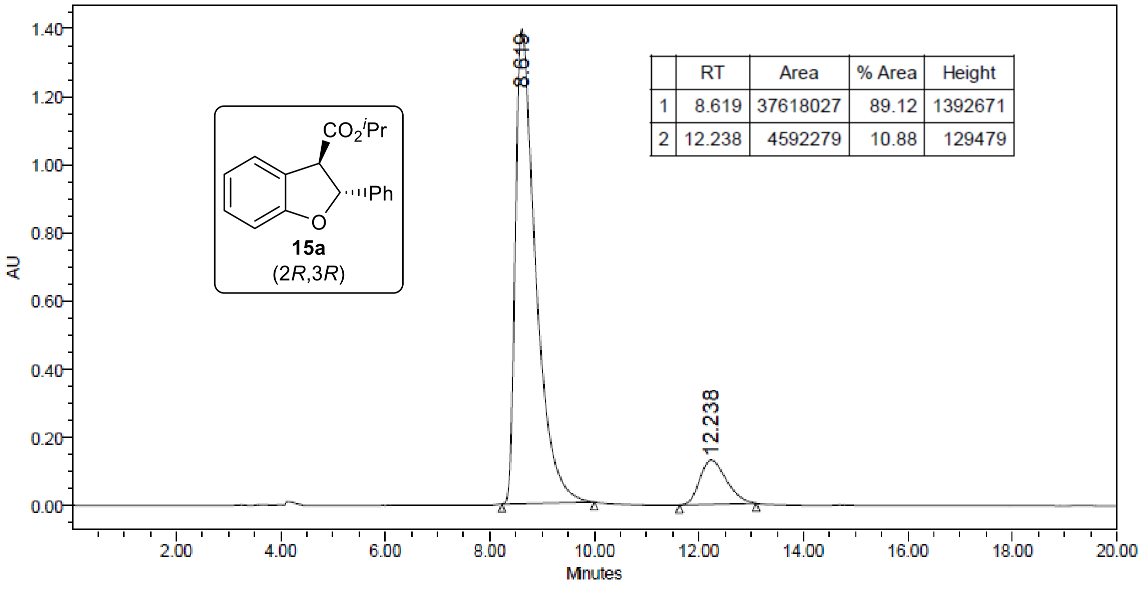

Isolated from the reaction of **13 in the presence of $Rh_2(2S\text{-}MPA)_4$ **9f** (**Table 2**, entry 22).*

Compound	Column	λ max (nm)	Mobile Phase (Hex:IPA)	Temp (°C)	Flow (mL/min)	Retention Time	
						Enantiomer	min
**15a**	Daicel Chiracel® OJ-H	210	99:1	25	1.0	(−) (2*R*,3*R*)	10
						(+) (2*S*,3*S*)	14

(2*S**,3*R**)-cis-3-Isopropyloxycarbonyl-2-phenyl-2,3-dihydrobenzofuran (15b)

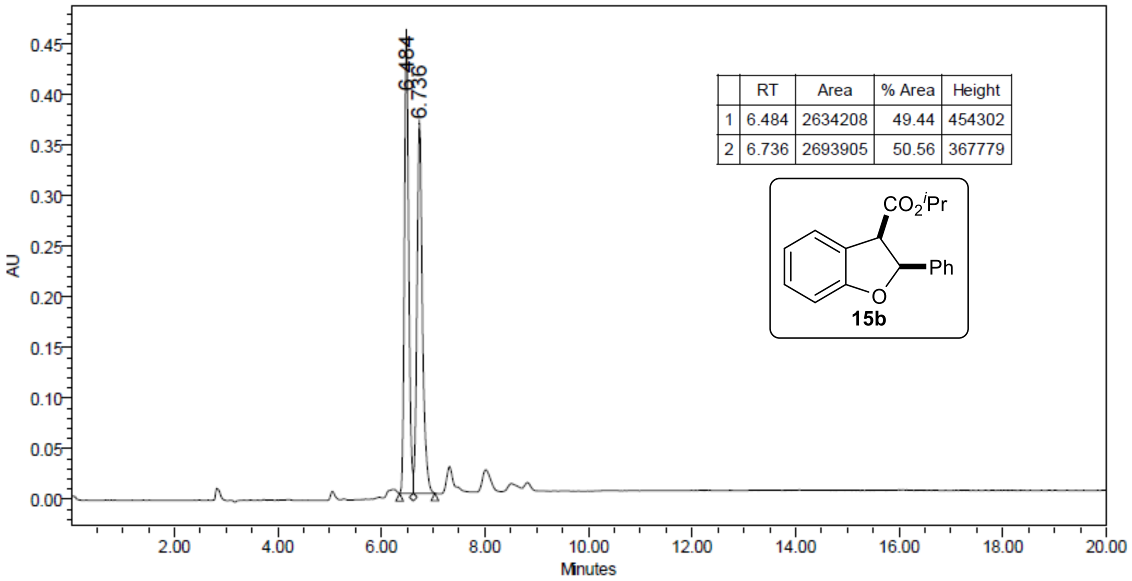

Isolated from the reaction of **13 in the presence of $Rh_2(OAc)_4$.*

(2*S*,3*R*)-2,3-cis-3-Isopropyloxycarbonyl-2-phenyl-2,3-dihydrobenzofuran (15b)

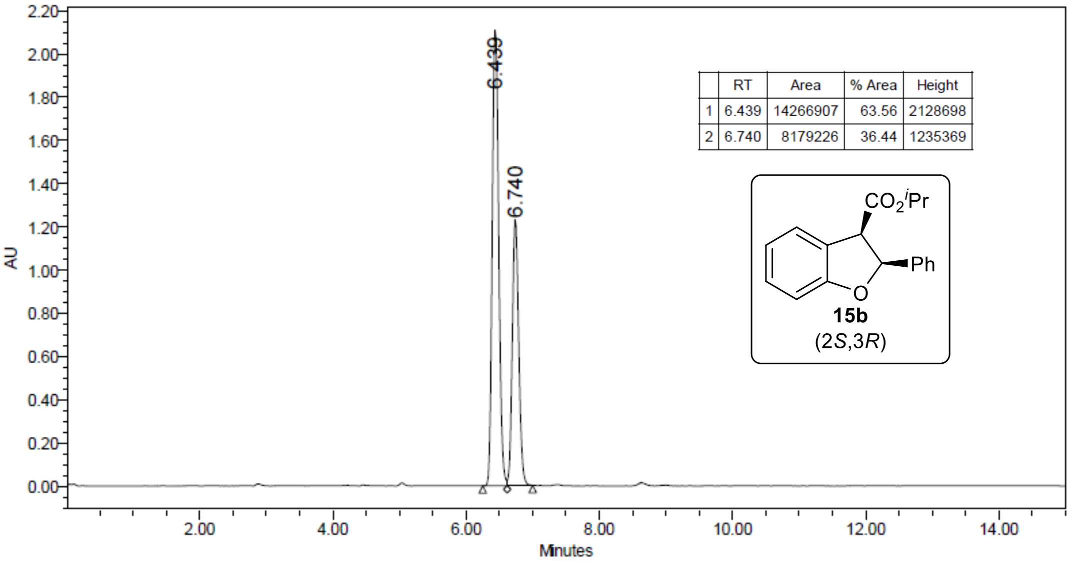

Isolated from the reaction of **13 in the presence of $Rh_2(2S\text{-}MPA)_4$ **9f** (**Table 2**, entry 22).*

Compound	Column	λ max (nm)	Mobile Phase (Hex:IPA)	Temp (°C)	Flow (mL/min)	Retention Time	
						Enantiomer	min
**15b**	Phenomenex Lux® 3μm Amylose-1	210	95:5	25	1.0	(−) (2*S*,3*R*)	6.5
						(+) (2*R*,3*S*)	6.7

Methyl (2*R,3*S**)-3-phenyltetrahydro-2*H*-thiopyran-2-carboxylate 1,1-dioxide (17a)**

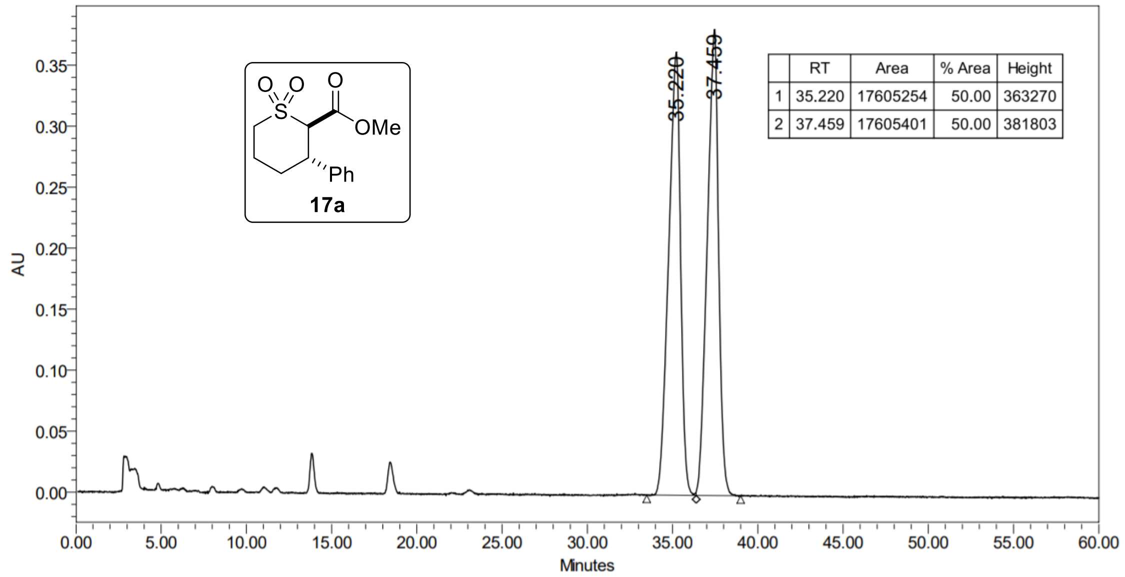

Isolated from reaction of **16 in the presence of $Rh_2(OAc)_4$ at room temperature.*

Methyl (2*R*,3*S*)-3-phenyltetrahydro-2*H*-thiopyran-2-carboxylate 1,1-dioxide (17a)

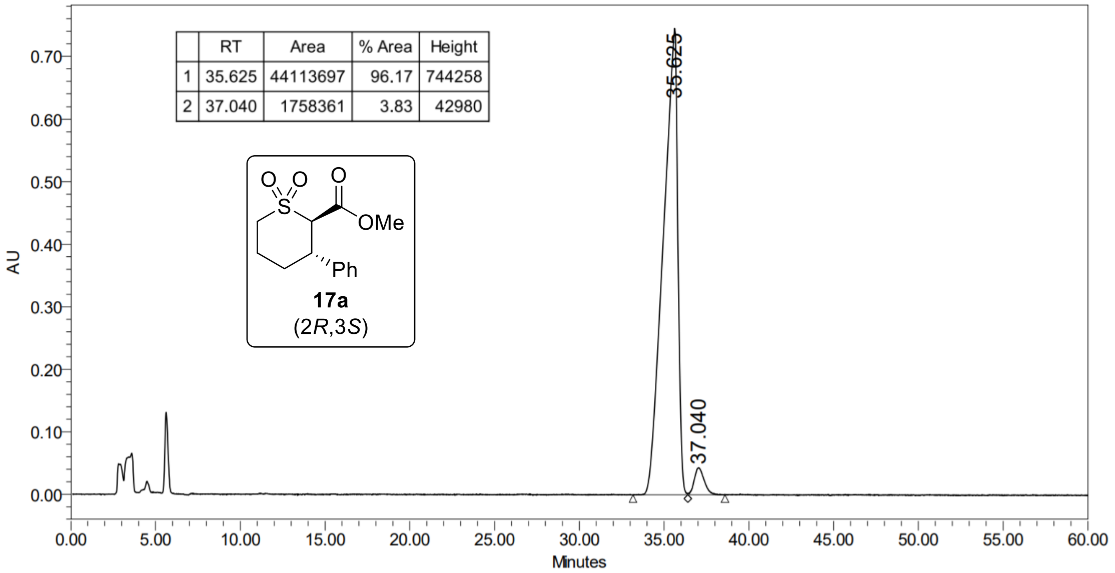

Isolated from reaction of **16 in the presence of $Rh_2(2S\text{-F-}2'\text{-NA})_4$ **9b** (**Table 4**).*

Compound	Column	λ max (nm)	Mobile Phase (Hex:IPA)	Temp (°C)	Flow (mL/min)	Retention Time	
						Enantiomer	min
**17a**	Phenomenex Lux® 3μm Amylose-1	209.8	90:10	25	1.0	(−) (2*R*,3*S*)	35
						(+) (2*S*,3*R*)	38

Methyl (2*R,3*S**)-3-(*p*-tolyl)tetrahydro-2*H*-thiopyran-2-carboxylate 1,1-dioxide (25a)**

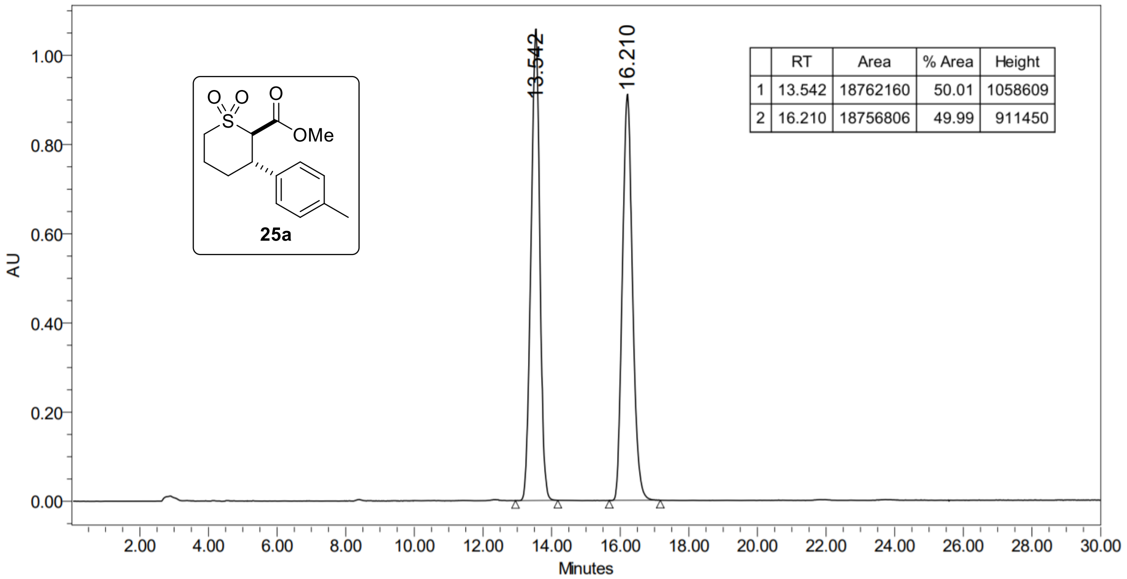

Isolated from reaction of **19 in the presence of $Rh_2(OAc)_4$ at room temperature.*

Methyl (2*R*,3*S*)-3-(*p*-tolyl)tetrahydro-2*H*-thiopyran-2-carboxylate 1,1-dioxide (25a)

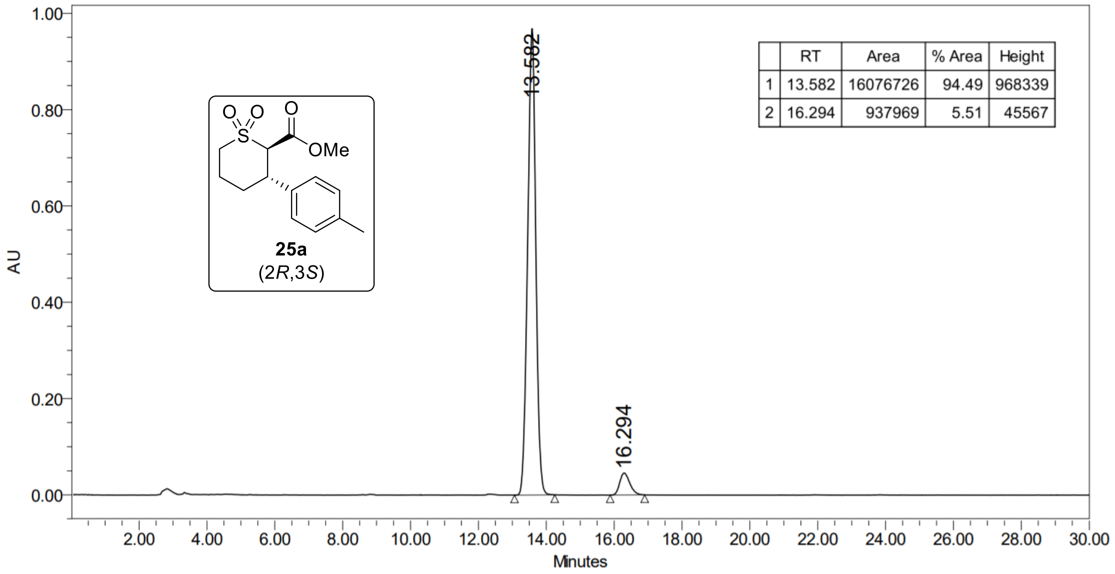

Isolated from reaction of **19 in the presence of Rh_2(2S-F-2'-NA)$_4$ **9b** (**Table 4**).*

Compound	Column	λ max (nm)	Mobile Phase (Hex:IPA)	Temp (°C)	Flow (mL/min)	Retention Time	
						Enantiomer	min
**25a**	Phenomenex Lux® 3μm Amylose-1	212	80:20	25	1.0	(−) (2*R*,3*S*)	14
						(+) (2*S*,3*R*)	16

Methyl (2*R,3*S**)-3-(4'-methoxyphenyl)tetrahydro-2*H*-thiopyran-2-carboxylate 1,1-dioxide (26a)**

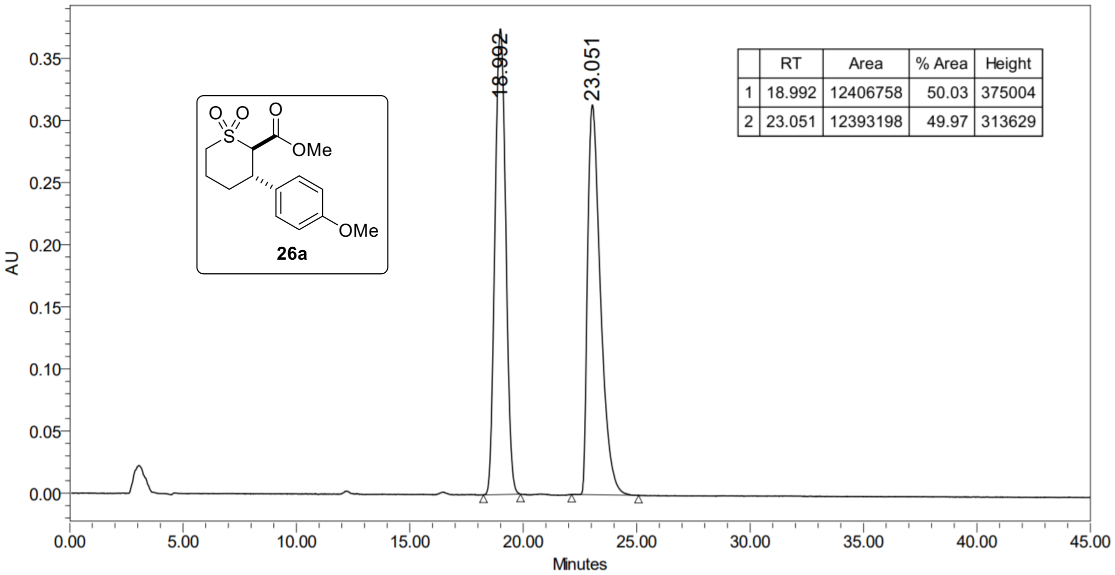

Isolated from reaction of **20 in the presence of $Rh_2(OAc)_4$ at room temperature.*

Methyl (2*R*,3*S*)-3-(4'-methoxyphenyl)tetrahydro-2*H*-thiopyran-2-carboxylate 1,1-dioxide (26a)

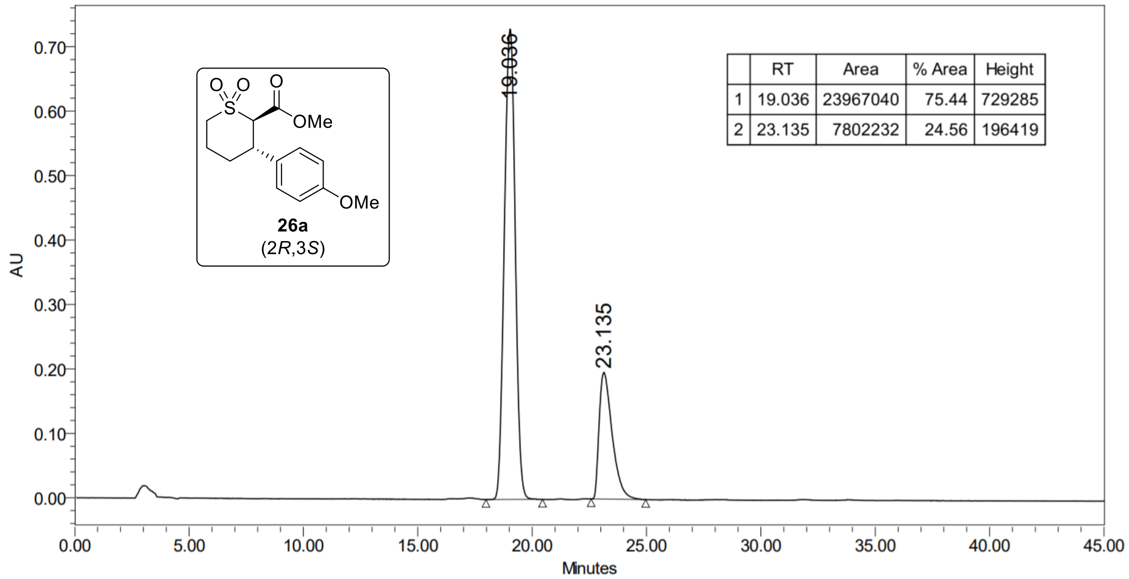

Isolated from reaction of **20 in the presence of Rh_2(2S-F-2'-NA)$_4$ **9b** (**Table 4**).*

Compound	Column	λ max (nm)	Mobile Phase (Hex:IPA)	Temp (°C)	Flow (mL/min)	Retention Time	
						Enantiomer	min
**26a**	Phenomenex Lux® 3μm Amylose-1	226.2	80:20	25	1.0	(−) (2*R*,3*S*)	19
						(+) (2*S*,3*R*)	23

Methyl (2*R**,3*S**)-3-(4'-fluorophenyl)tetrahydro-2*H*-thiopyran-2-carboxylate 1,1-dioxide (27a)

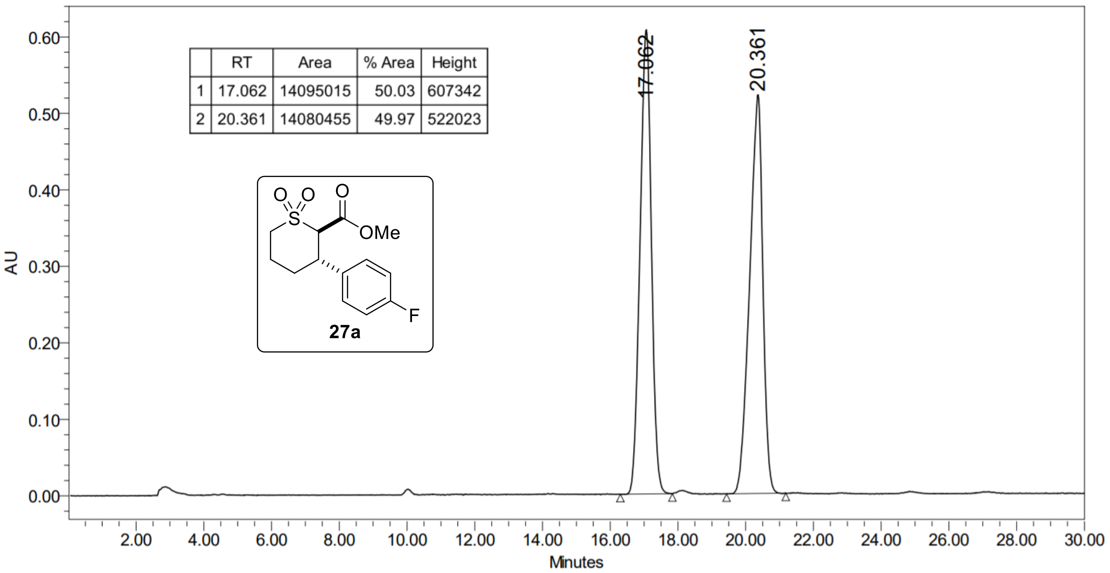

Isolated from reaction of **21 in the presence of $Rh_2(OAc)_4$ at room temperature.*

Methyl (2*R*,3*S*)-3-(4'-fluorophenyl)tetrahydro-2*H*-thiopyran-2-carboxylate 1,1-dioxide (27a)

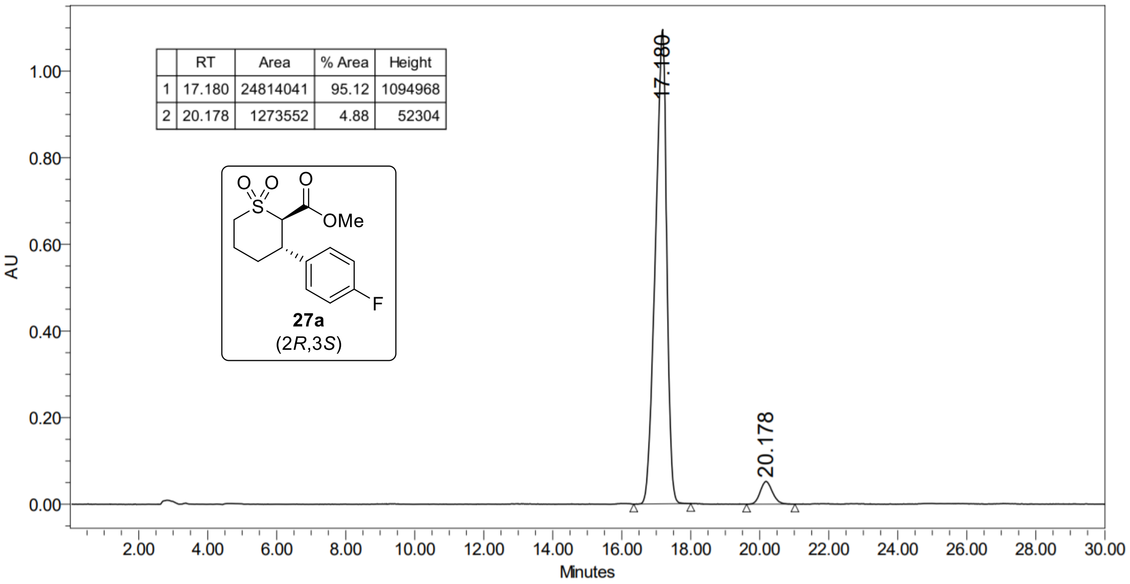

Isolated from reaction of **21 in the presence of Rh_2(2S-F-2'-NA)$_4$ **9b** (**Table 4**).*

Compound	Column	λ max (nm)	Mobile Phase (Hex:IPA)	Temp (°C)	Flow (mL/min)	Retention Time	
						Enantiomer	min
**27a**	Phenomenex Lux® 3µm Amylose-1	209.8	80:20	25	1.0	(−) (2*R*,3*S*)	17
						(+) (2*S*,3*R*)	20

((2*R,3*S**)-1,1-Dioxido-3-phenyltetrahydro-2*H*-thiopyran-2-yl)(phenyl)methanone (28a)**

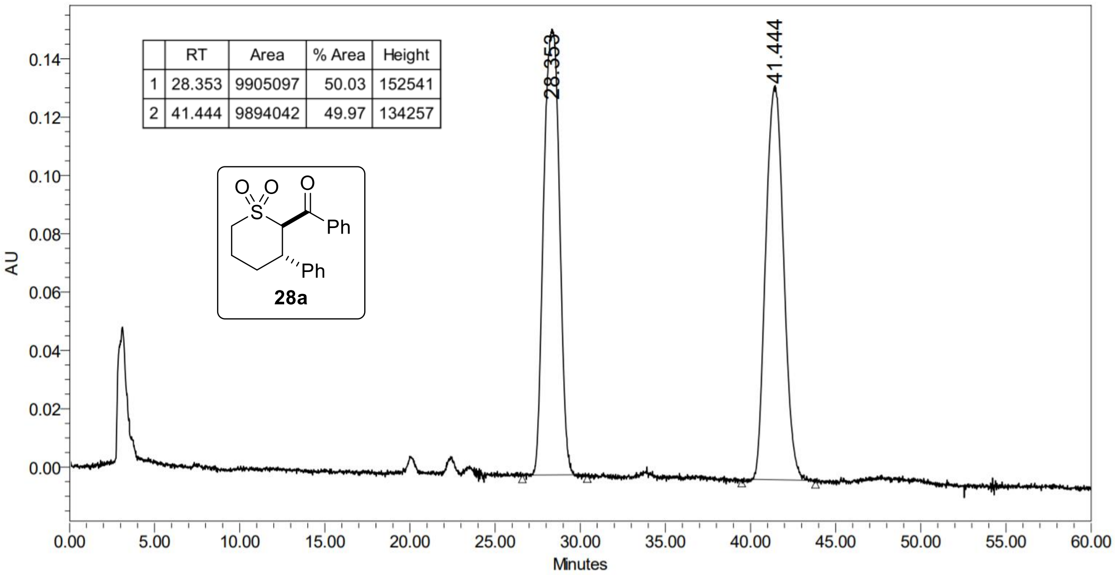

Isolated from reaction of **22 in the presence of $Rh_2(OAc)_4$ at room temperature.*

((2*R,3*S**)-1,1-Dioxido-3-phenyltetrahydro-2*H*-thiopyran-2-yl)(phenyl)methanone (28a)**

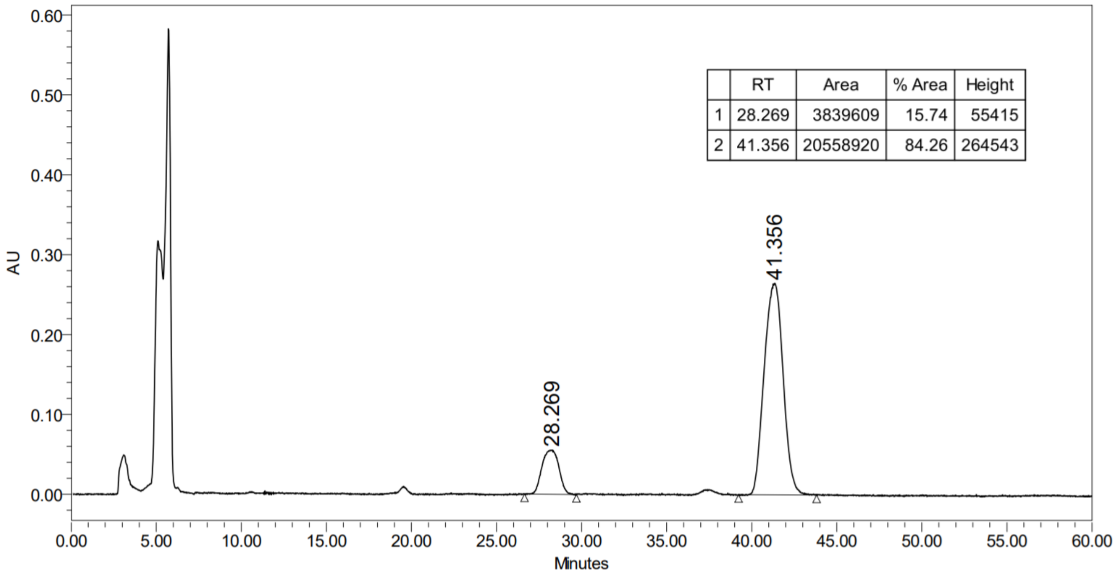

Isolated from reaction of **22 in the presence of $Rh_2(2S\text{-}F\text{-}2'\text{-}NA)_4$ **9b** (**Table 4**).*

Compound	Column	λ max (nm)	Mobile Phase (Hex:IPA)	Temp (°C)	Flow (mL/min)	Retention Time	
						Enantiomer	min
**28a**	Phenomenex Lux® 3μm Amylose-1	209.8	90:10	25	1.0	(−)	28
						(+)	41

Benzyl (2*R,3*R**)-3-octyltetrahydro-2*H*-thiopyran-2-carboxylate 1,1-dioxide (29a)**

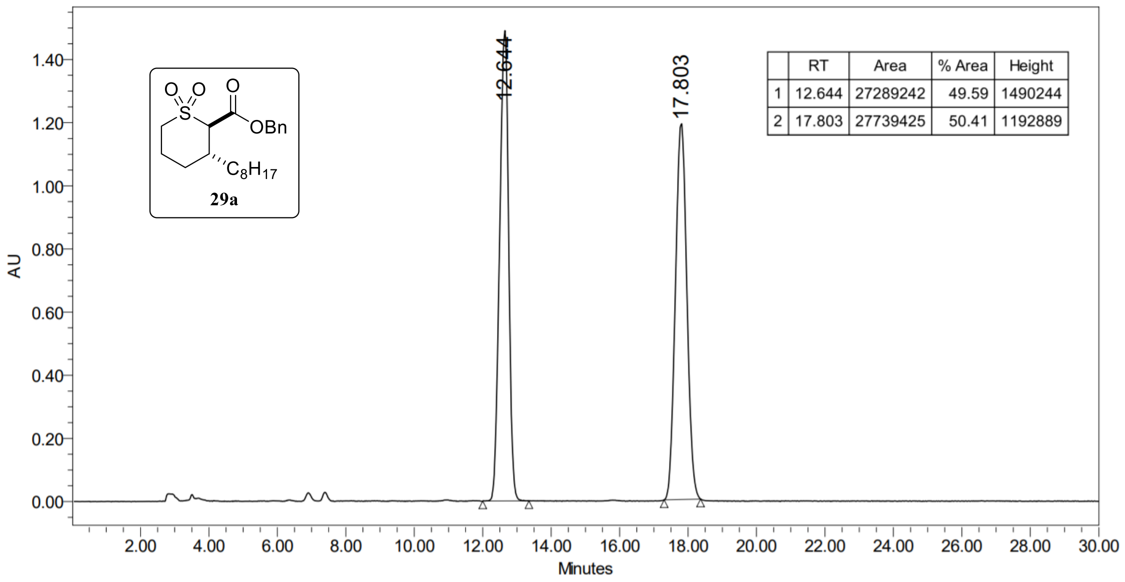

Isolated from reaction of **23 in the presence of $Rh_2(OAc)_4$ at room temperature.*

Benzyl (2*R*,3*R*)-3-octyltetrahydro-2*H*-thiopyran-2-carboxylate 1,1-dioxide (29a)

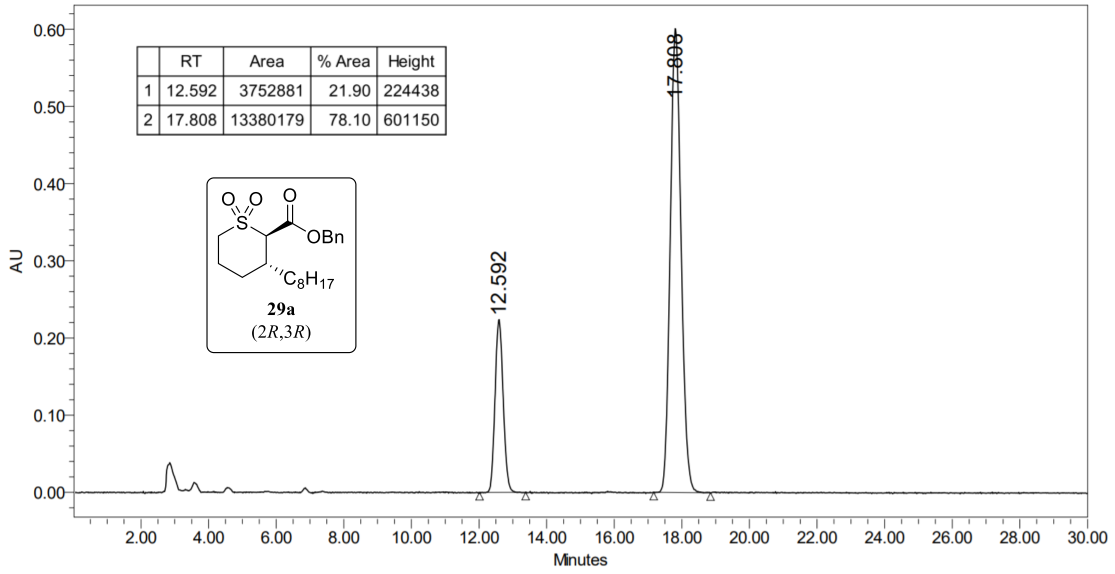

Isolated from reaction of **23 in the presence of $Rh_2(2S\text{-F-2'-NA})_4$ **9b** (**Table 4**).*

Compound	Column	λ max (nm)	Mobile Phase (Hex:IPA)	Temp (°C)	Flow (mL/min)	Retention Time	
						Enantiomer	min
**29a**	Phenomenex Lux® 3μm Amylose-1	209.8	90:10	25	1.0	(+) (2*S*,3*S*)	13
						(−) (2*R*,3*R*)	18

Methyl (1*R,4a*S**,8a*R**)-octahydro-1*H*-isothiochromene-1-carboxylate 2,2-dioxide (30a)**

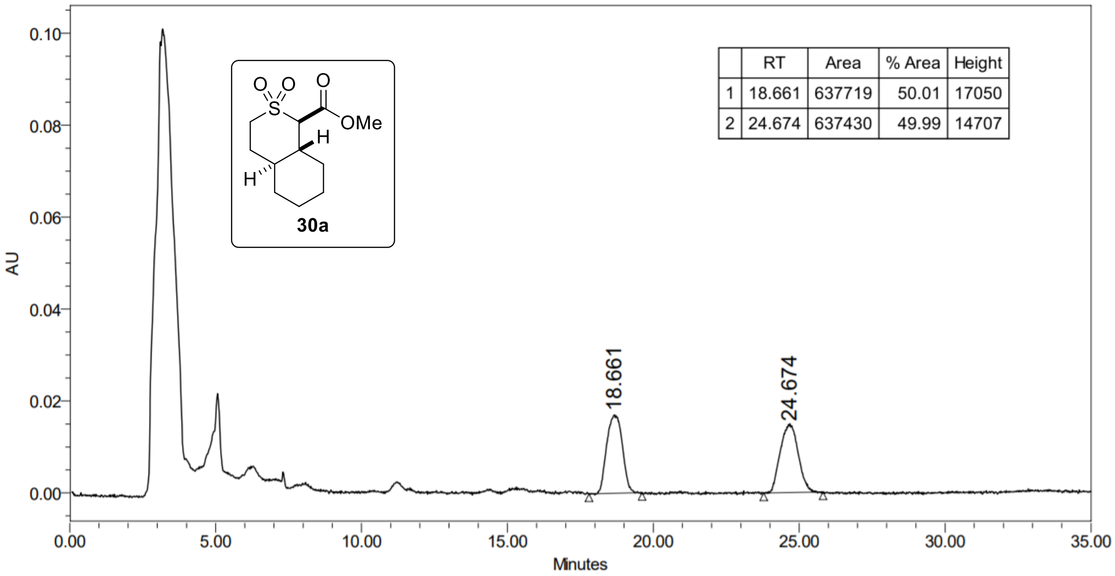

*Isolated from reaction of **24** in the presence of $Rh_2(OAc)_4$ at room temperature.

Methyl (1*R*,4a*S*,8a*R*)-octahydro-1*H*-isothiochromene-1-carboxylate 2,2-dioxide (30a)

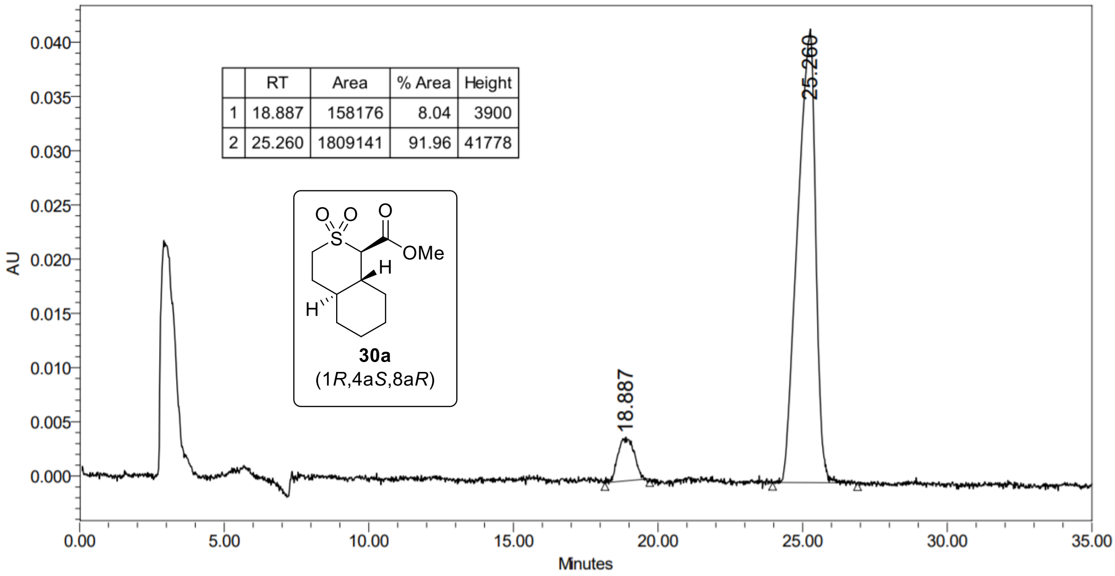

*Isolated from reaction of **24** in the presence of Rh_2(2S-F-2'-NA)$_4$ **9b** (**Table 4**).

Compound	Column	λ max (nm)	Mobile Phase (Hex:IPA)	Temp (°C)	Flow (mL/min)	Retention Time	
						Enantiomer	min
**30a**	Phenomenex Lux® 3*μ*m Amylose-1	216	90:10	25	1.0	(+) (1*S*,4a*R*,8a*S*)	19
						(−) (1*R*,4a*S*,8a*R*)	25

9-Aza-9-benzyl-1(*R)-cyanobicyclo[5.3.0]deca-2,4,6-trien-10-one (32)**

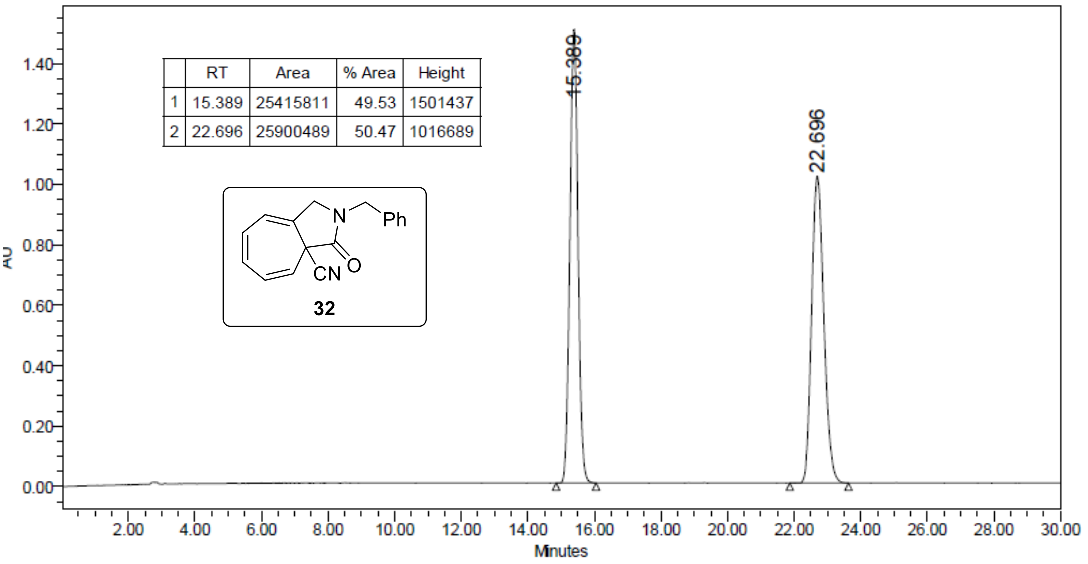

Isolated from reaction of **31 in the presence of $Rh_2(OAc)_4$ at room temperature.*

9-Aza-9-benzyl-1(*S*)-cyanobicyclo[5.3.0]deca-2,4,6-trien-10-one (32)

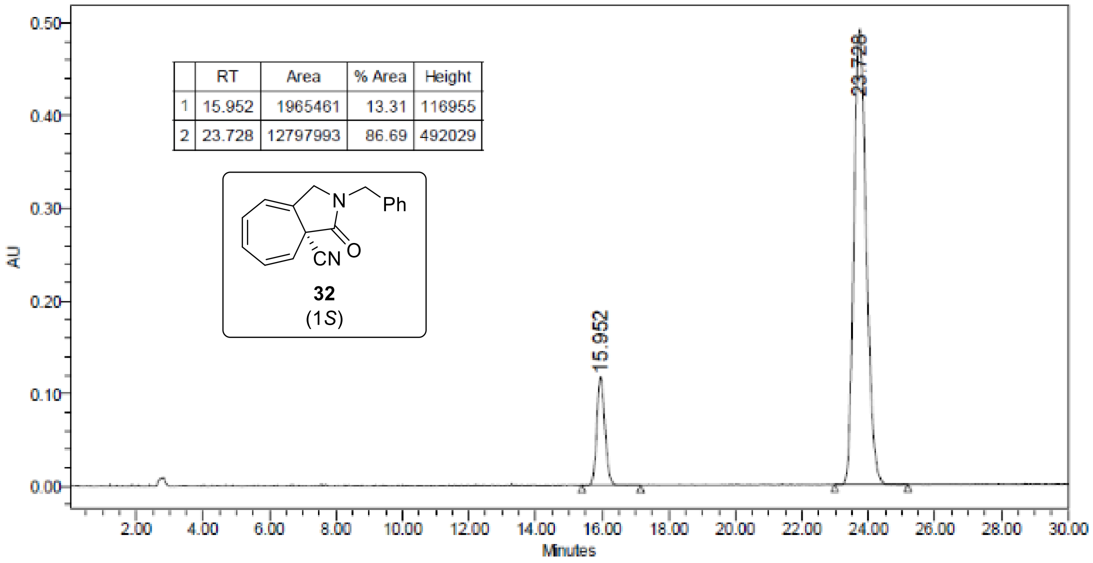

Isolated from reaction of **31 in the presence of Rh_2(2S-FOMePA)$_4$ **9e** (**Table 5**).*

Compound	Column	λ max (nm)	Mobile Phase (Hex:IPA)	Temp (°C)	Flow (mL/min)	Retention Time	
						Enantiomer	min
**32**	Phenomenex Lux® 3μm Amylose-1	209.8	85 : 25	25	1.0	(−) (1*R*)	16
						(+) (1*S*)	24

9-Aza-9-*tert*-butyl-1(*R**)-cyano-4-fluorobicyclo[5.3.0]deca-2,4,6-trien-10-one (46)

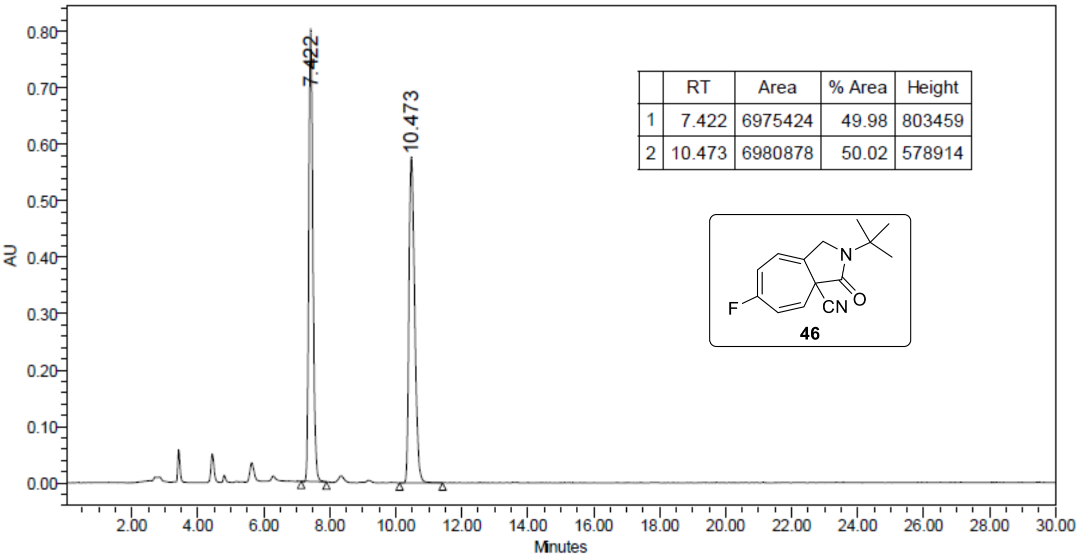

Isolated from reaction of **33 in the presence of $Rh_2(OAc)_4$ at room temperature.*

9-Aza-9-*tert*-butyl-1(*S*)-cyano-4-fluorobicyclo[5.3.0]deca-2,4,6-trien-10-one (46)

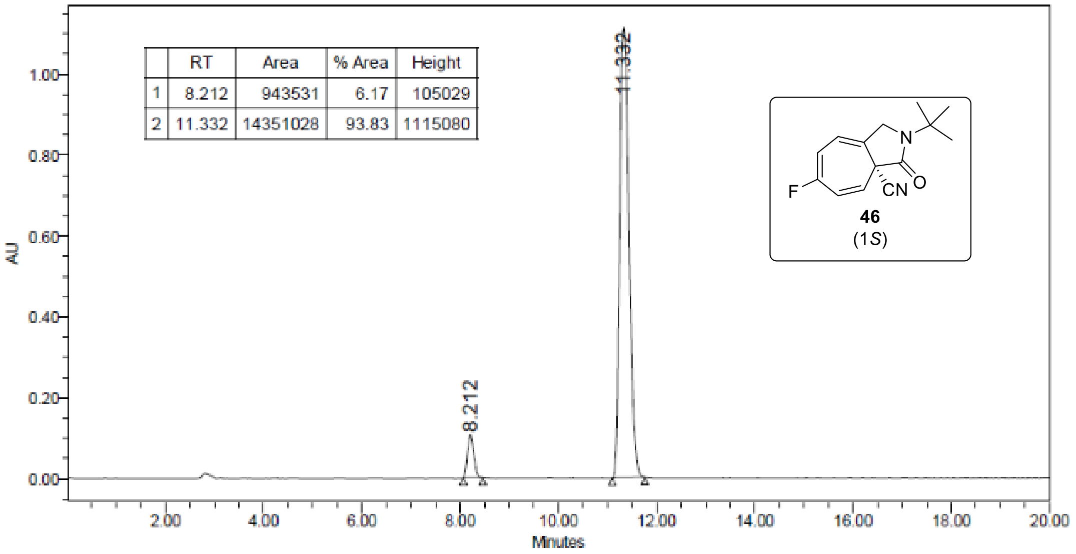

Isolated from reaction of **33 in the presence of Rh_2(2S-FOMePA)$_4$ **9e** (**Table 5**).*

Compound	Column	λ max (nm)	Mobile Phase (Hex:IPA)	Temp (°C)	Flow (mL/min)	Retention Time	
						Enantiomer	min
**46**	Phenomenex Lux® 3μm Amylose-1	209.8	85 : 25	25	1.0	(−) (1*R*)	8
						(+) (1*S*)	11

9-Aza-9-*tert*-butyl-1(*R)-cyano-4-bromobicyclo[5.3.0]deca-2,4,6-trien-10-one (47)**

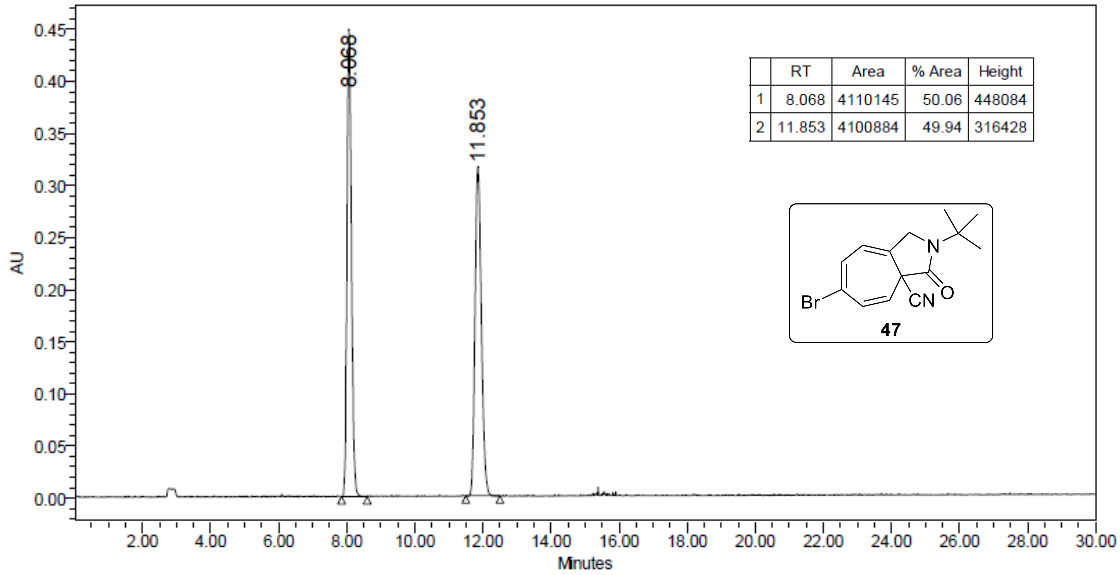

Isolated from reaction of **34 in the presence of $Rh_2(OAc)_4$ at room temperature.*

9-Aza-9-*tert*-butyl-1(*S*)-cyano-4-bromobicyclo[5.3.0]deca-2,4,6-trien-10-one (47)

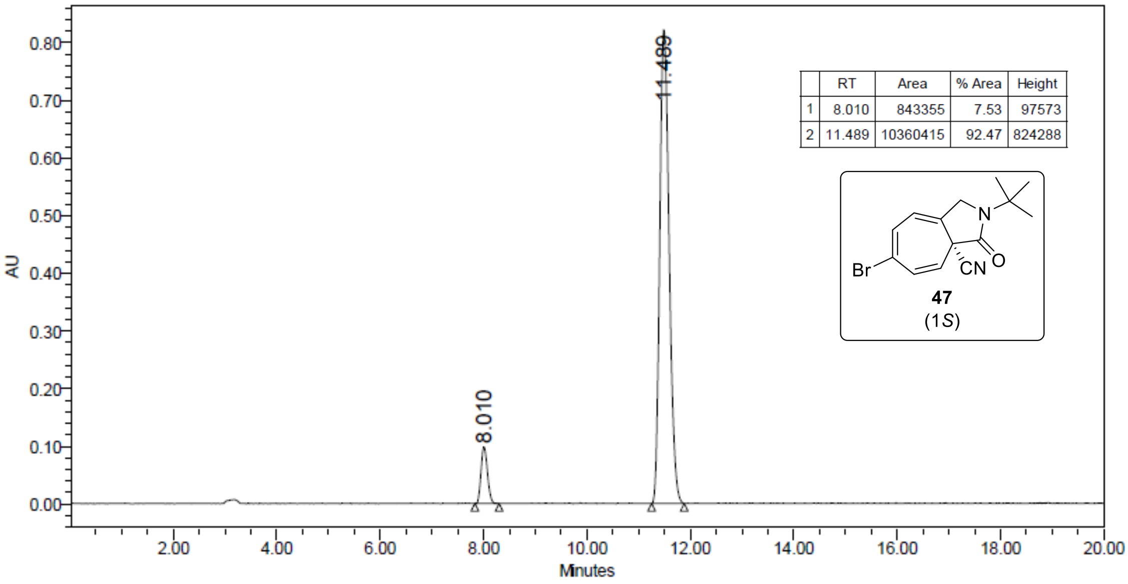

Isolated from reaction of **34 in the presence of Rh_2(2S-FOMePA)$_4$ **9e** (**Table 5**).*

Compound	Column	λ max (nm)	Mobile Phase (Hex:IPA)	Temp (°C)	Flow (mL/min)	Retention Time	
						Enantiomer	min
**47**	Phenomenex Lux® 3μm Amylose-1	209.8	85 : 25	25	1.0	(−) (1*R*)	8
						(+) (1*S*)	12

9-Aza-9-*tert*-butyl-1(*R)-cyano-4-chlorobicyclo[5.3.0]deca-2,4,6-trien-10-one (48)**

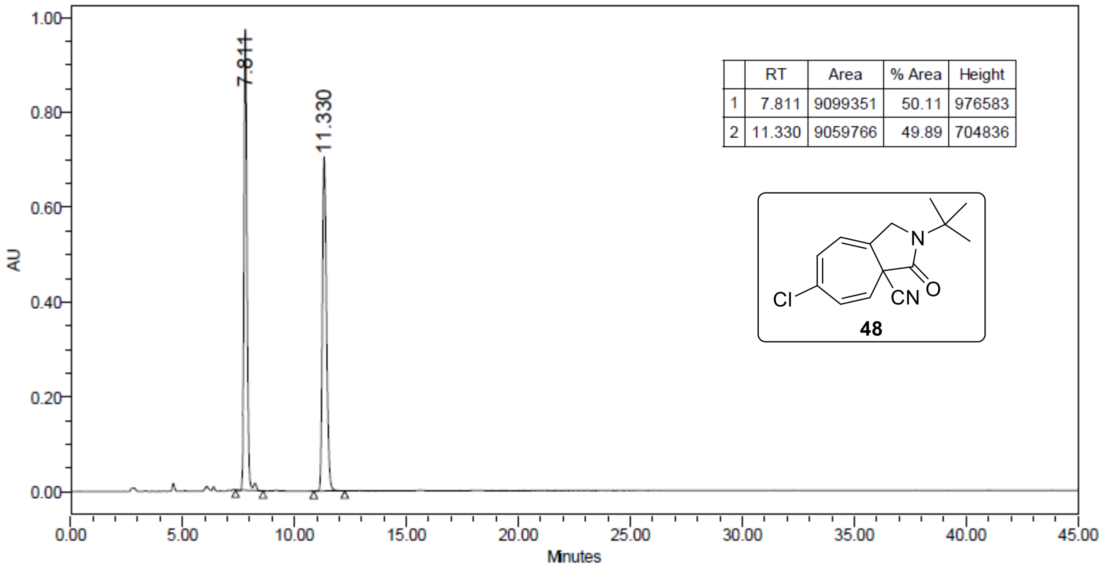

Isolated from reaction of **35 in the presence of $Rh_2(OAc)_4$ at room temperature.*

9-Aza-9-*tert*-butyl-1(*S*)-cyano-4-chlorobicyclo[5.3.0]deca-2,4,6-trien-10-one (48)

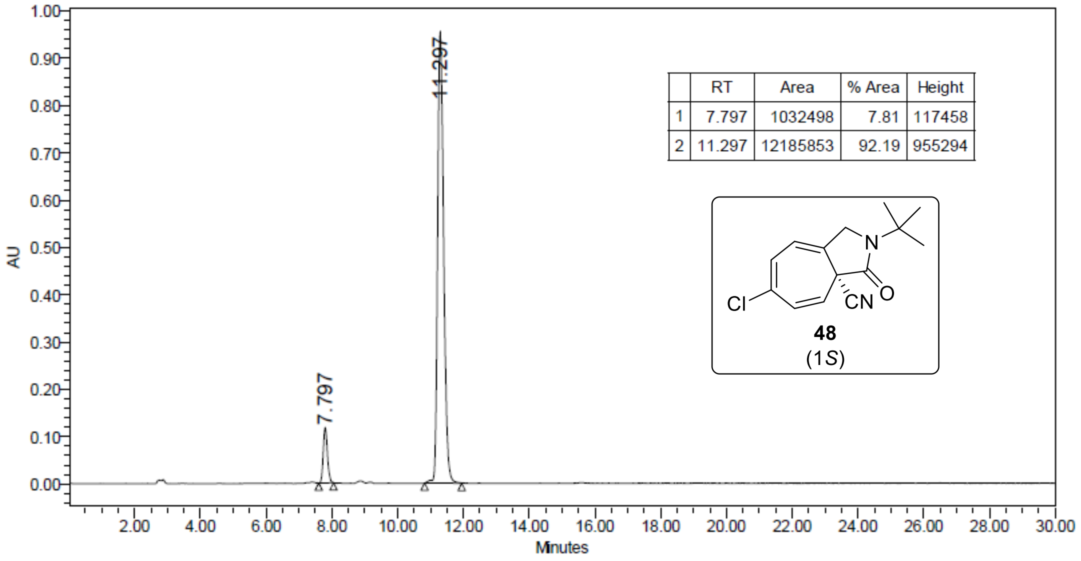

Isolated from reaction of **35 in the presence of Rh_2(2S-FOMePA)$_4$ **9e** (**Table 5**).*

**Compound**	**Column**	**λ max (nm)**	**Mobile Phase (Hex:IPA)**	**Temp (°C)**	**Flow (mL/min)**	**Retention Time**	
						**Enantiomer**	**min**
**48**	Phenomenex Lux® 3μm Amylose-1	209.8	85 : 25	25	1.0	(−) (1*R*)	8
						(+) (1*S*)	11

9-Aza-9-*tert*-butyl-1(*R**)-cyano-2,6-dichlorobicyclo[5.3.0]deca-2,4,6-trien-10-one (49)

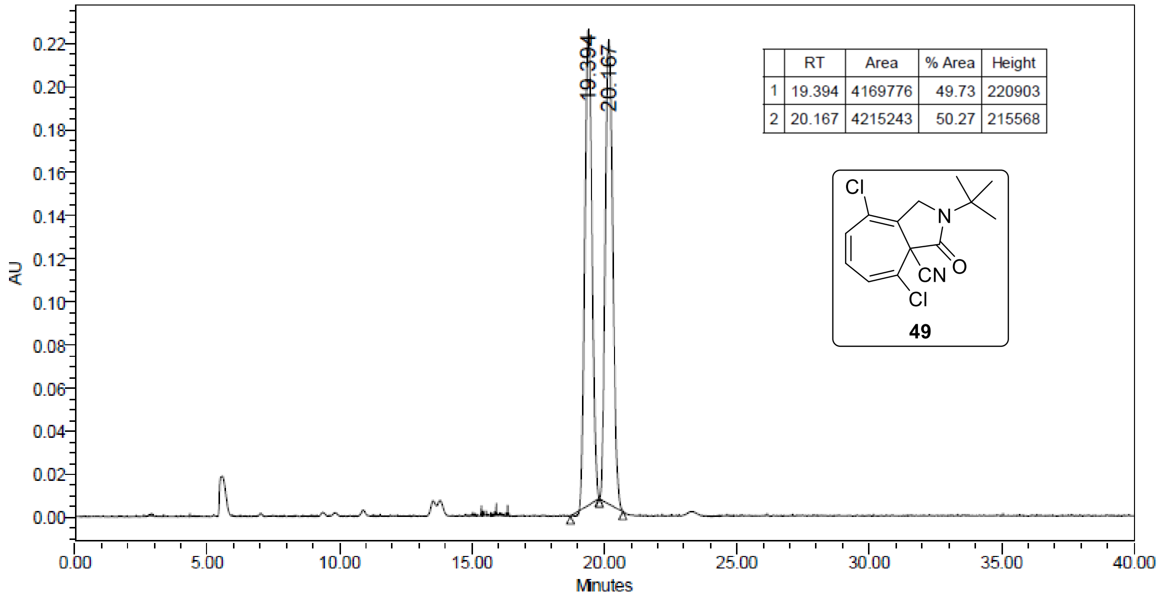

Isolated from reaction of **36 in the presence of $Rh_2(OAc)_4$ at room temperature.*

9-Aza-9-*tert*-butyl-1(*R*)-cyano-2,6-dichlorobicyclo[5.3.0]deca-2,4,6-trien-10-one (49)

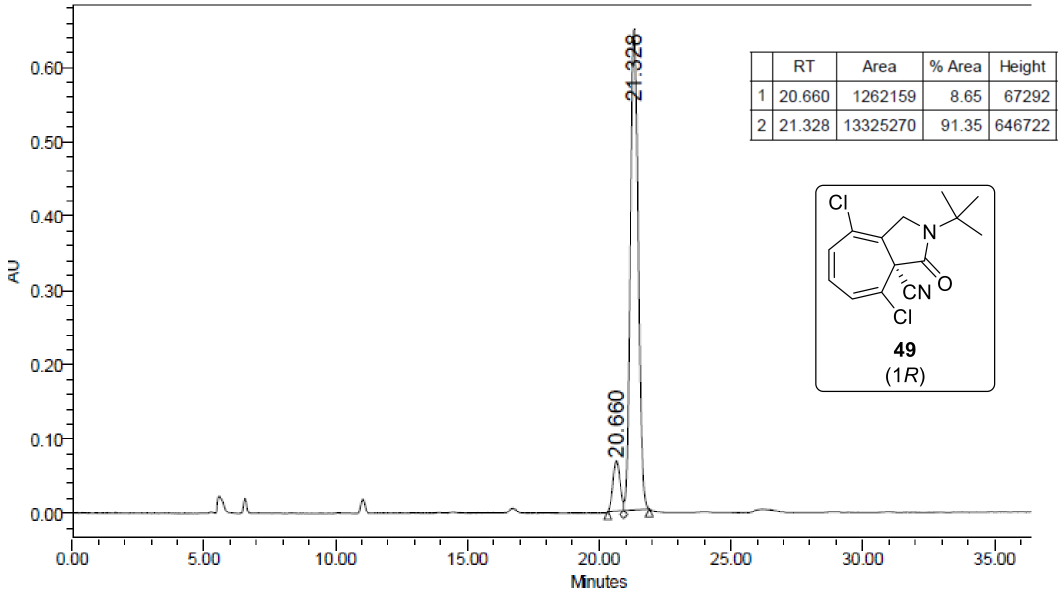

Isolated from reaction of **36 in the presence of Rh_2(2S-FOMePA)$_4$ **9e** (**Table 5**).*

Compound	Column	λ max (nm)	Mobile Phase (Hex:IPA)	Temp (°C)	Flow (mL/min)	Retention Time	
						Enantiomer	min
**49**	Phenomenex Lux® 3μm Amylose-1	214.5	95 : 05	25	0.5	(−) (1*S*)	19
						(+) (1*R*)	20

9-Aza-9-*tert*-butyl-1(*R**)-cyano-4-methylbicyclo[5.3.0]deca-2,4,6-trien-10-one (50)

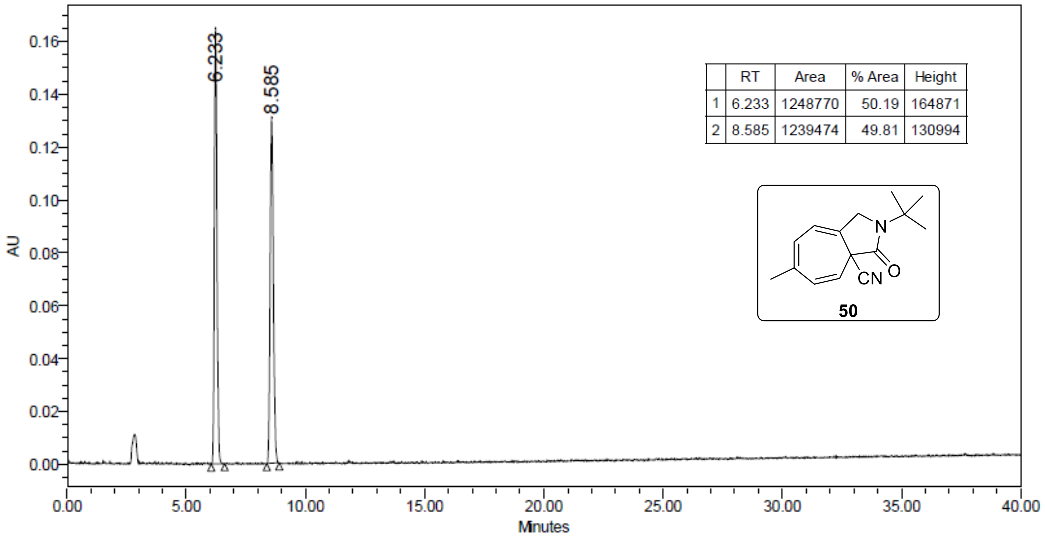

Isolated from reaction of* ***37* *in the presence of* $Rh_2(OAc)_4$ *at room temperature.*

9-Aza-9-*tert*-butyl-1(*S*)-cyano-4-methylbicyclo[5.3.0]deca-2,4,6-trien-10-one (50)

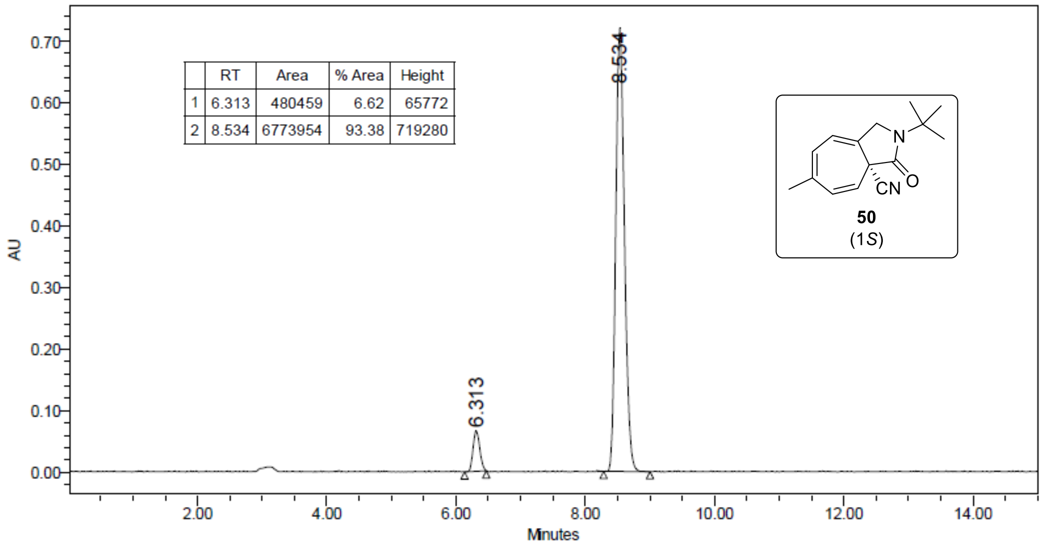

Isolated from reaction of* ***37* *in the presence of* $Rh_2(2S\text{-}FOMePA)_4$ ***9e*** *(****Table 5****).*

Compound	Column	λ max (nm)	Mobile Phase (Hex:IPA)	Temp (°C)	Flow (mL/min)	Retention Time	
						**Enantiomer**	**min**
**50**	Phenomenex Lux® 3μm Amylose-1	209.8	85 : 25	25	1.0	(−) (1*R*)	6
						(+) (1*S*)	9

9-Aza-9-*tert*-butyl-1(*R)-cyano-3,5-dimethylbicyclo[5.3.0]deca-2,4,6-trien-10-one (51)**

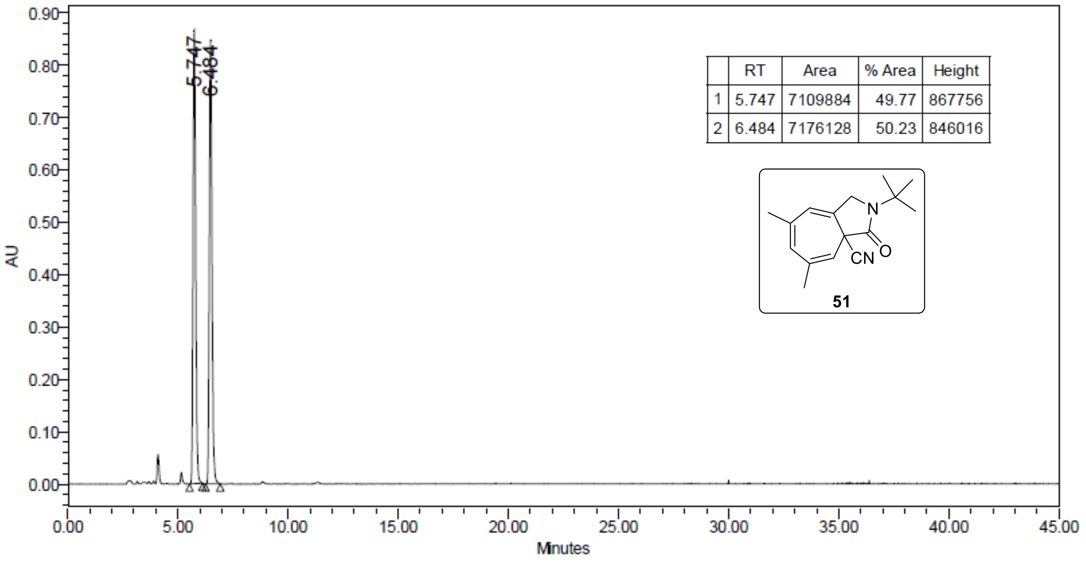

Isolated from reaction of **38 in the presence of $Rh_2(OAc)_4$ at room temperature.*

9-Aza-9-*tert*-butyl-1(*S*)-cyano-3,5-dimethylbicyclo[5.3.0]deca-2,4,6-trien-10-one (51)

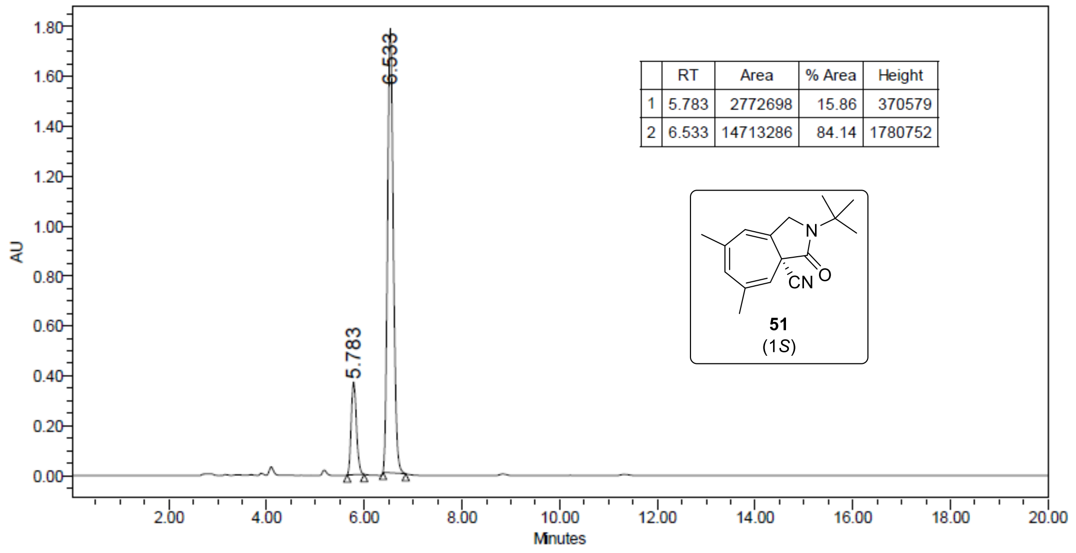

Isolated from reaction of **38 in the presence of Rh_2(2S-FOMePA)$_4$ **9e** (**Table 5**).*

Compound	Column	λ max (nm)	Mobile Phase (Hex:IPA)	Temp (°C)	Flow (mL/min)	Retention Time	
						Enantiomer	min
**51**	Phenomenex Lux® 3μm Amylose-1	209.8	85 : 25	25	1.0	(−) (1*R*)	6
						(+) (1*S*)	7

9-Aza-9-*tert*-butyl-1(*R)-cyano-2,4,6-trimethylbicyclo[5.3.0]deca-2,4,6-trien-10-one (52)**

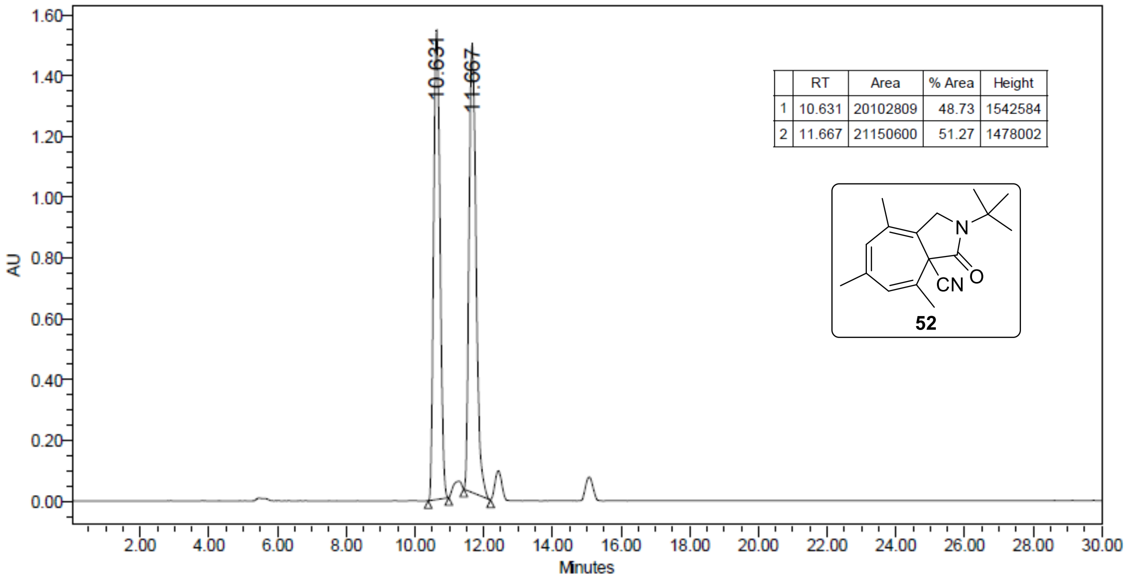

*Isolated from reaction of **39** in the presence of $Rh_2(OAc)_4$ at room temperature.*

9-Aza-9-*tert*-butyl-1(*S*)-cyano-2,4,6-trimethylbicyclo[5.3.0]deca-2,4,6-trien-10-one (52)

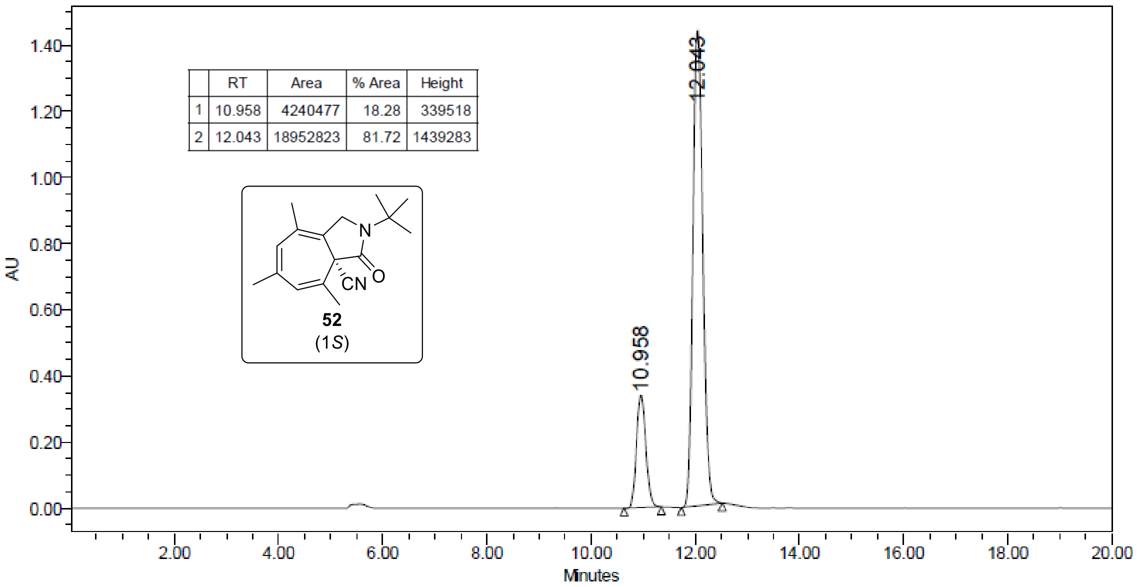

*Isolated from reaction of **39** in the presence of Rh_2(2S-FOMePA)$_4$ **9e** (**Table 5**).*

Compound	Column	λ max (nm)	Mobile Phase (Hex:IPA)	Temp (°C)	Flow (mL/min)	Retention Time	
						Enantiomer	min
**52**	Phenomenex Lux® 3μm Amylose-1	211.0	90 : 10	25	0.5	(−) (1*R*)	11
						(+) (1*S*)	12

9-Aza-9-*tert*-butyl-1(*R)-cyanobicyclo[5.3.0]deca-2,4,6-trien-10-one (53)**

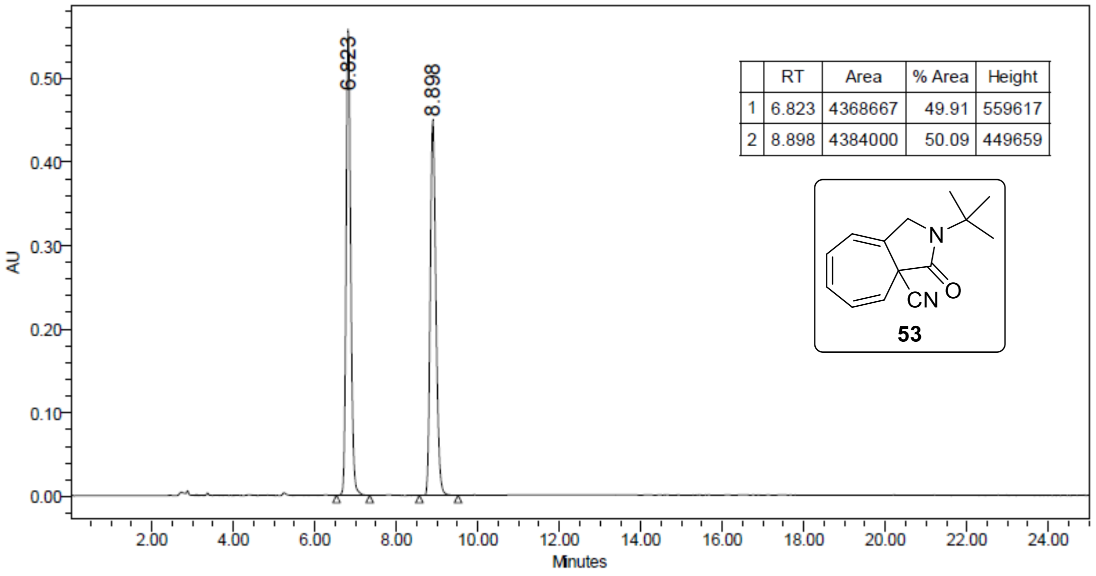

Isolated from reaction of **40 in the presence of $Rh_2(OAc)_4$ at room temperature.*

9-Aza-9-*tert*-butyl-1(*S*)-cyanobicyclo[5.3.0]deca-2,4,6-trien-10-one (53)

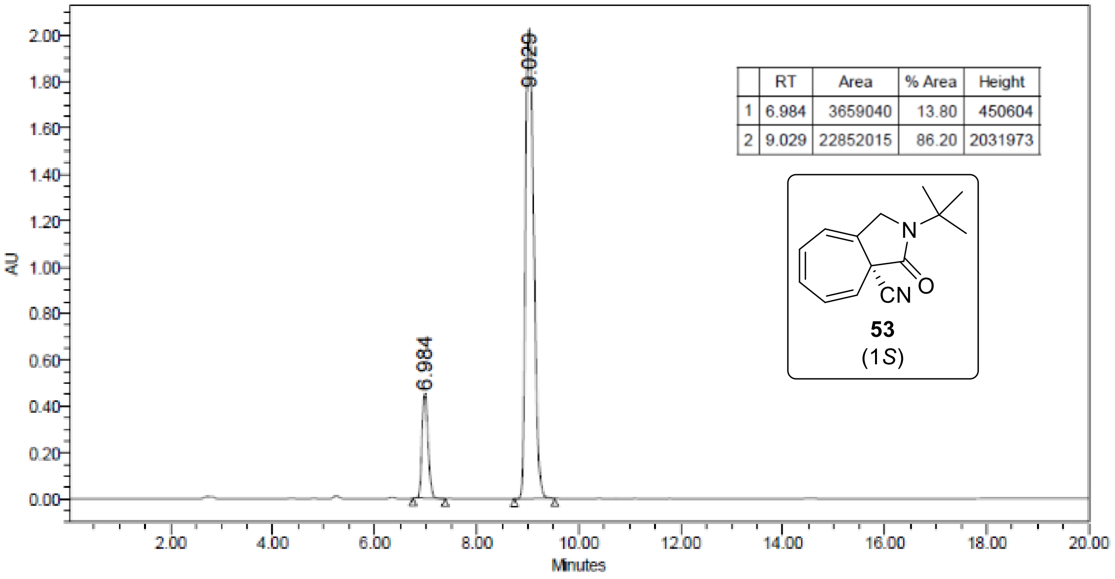

Isolated from reaction of **40 in the presence of $Rh_2(2S\text{-}FOMePA)_4$ **9e** (**Table 5**).*

Compound	Column	λ max (nm)	Mobile Phase (Hex:IPA)	Temp (°C)	Flow (mL/min)	Retention Time	
						Enantiomer	min
**53**	Phenomenex Lux® 3μm Amylose-1	209.8	85 : 25	25	1.0	(−) (1*R*)	7
						(+) (1*S*)	9

9-Aza-9-*tert*-butyl-1(*R)-cyano-4-nitrobicyclo[5.3.0]deca-2,4,6-trien-10-one (54)**

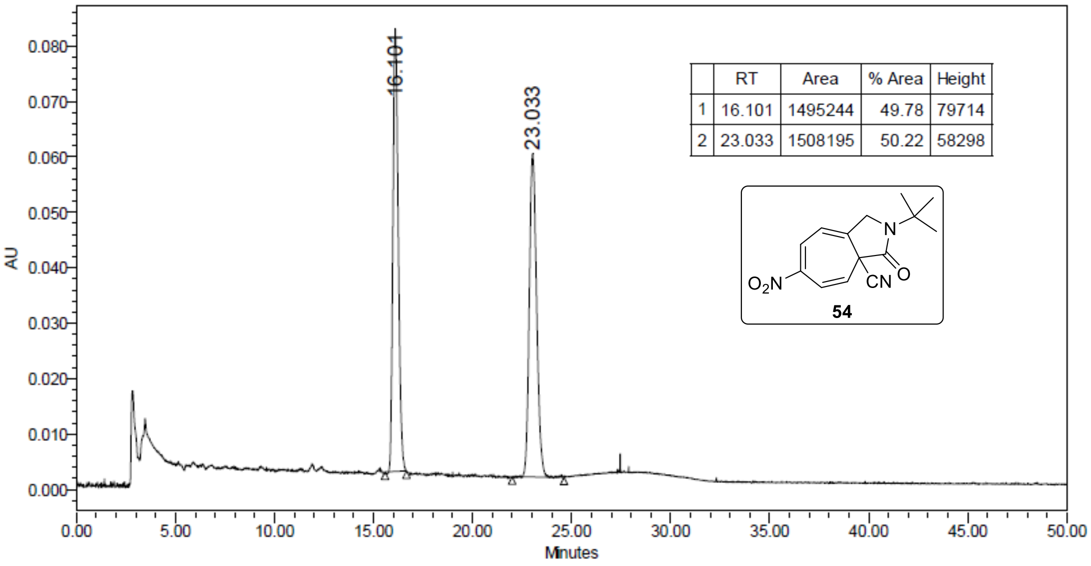

Isolated from reaction of **41 in the presence of $Rh_2(OAc)_4$ at room temperature.*

9-Aza-9-*tert*-butyl-1(*R*)-cyano-4-nitrobicyclo[5.3.0]deca-2,4,6-trien-10-one (54)

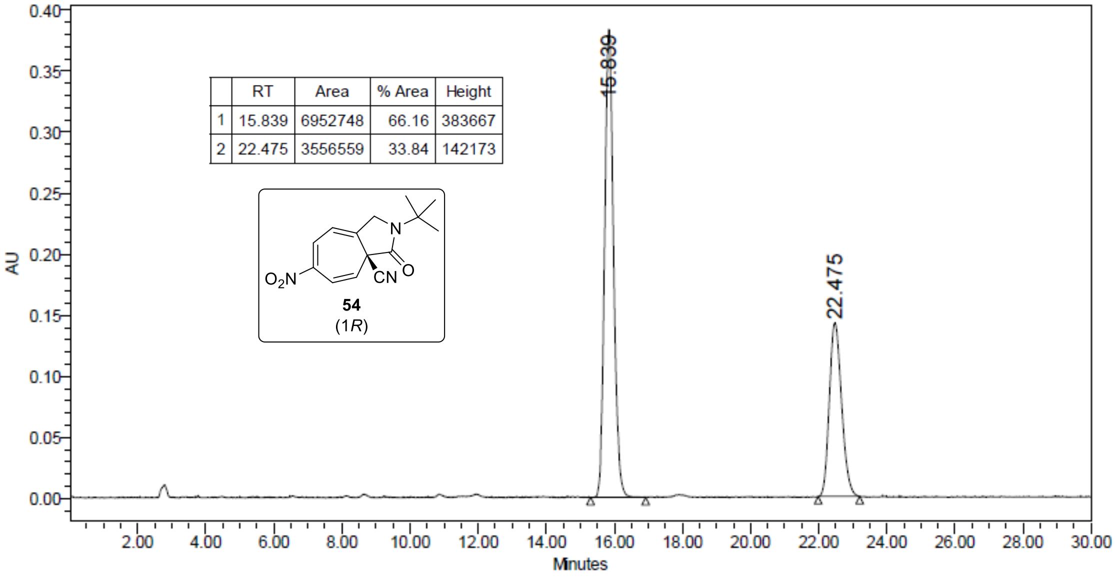

Isolated from reaction of **41 in the presence of $Rh_2(2S\text{-}FOMePA)_4$ **9e** (**Table 5**).*

Compound	Column	λ max (nm)	Mobile Phase (Hex:IPA)	Temp (°C)	Flow (mL/min)	Retention Time	
						Enantiomer	min
**54**	Phenomenex Lux® 3μm Amylose-1	209.8	85 : 25	25	1.0	(−) (1*R*)	16
						(+) (1*S*)	23

9-Aza-9-*tert*-butyl-1(*R)-cyano-4-methoxycarbonylbicyclo[5.3.0]deca-2,4,6-trien-10-one (55)**

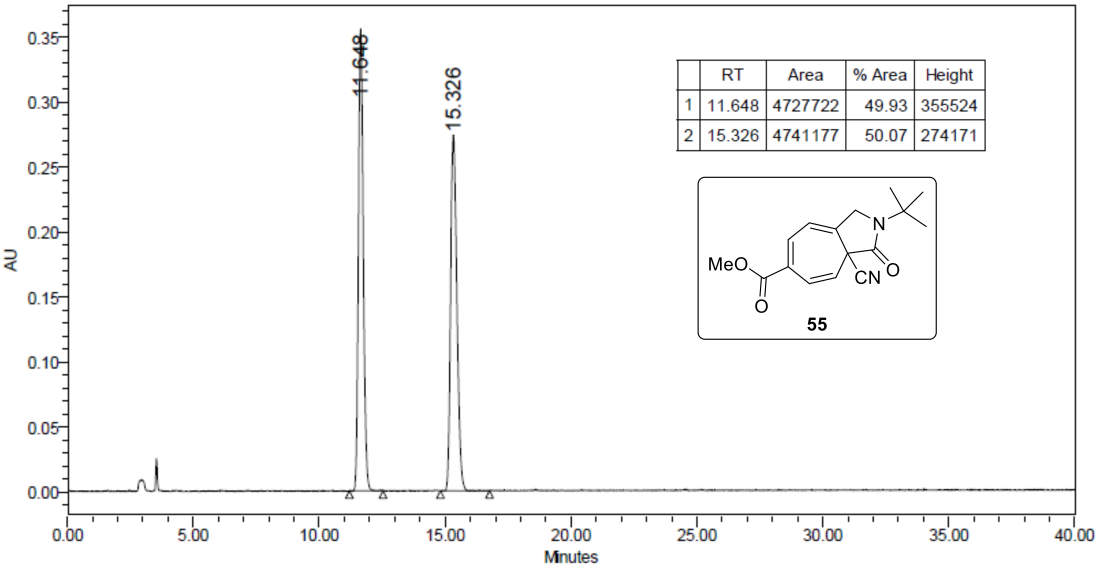

Isolated from reaction of **42 in the presence of $Rh_2(OAc)_4$ at room temperature.*

9-Aza-9-*tert*-butyl-1(*S*)-cyano-4-methoxycarbonylbicyclo[5.3.0]deca-2,4,6-trien-10-one (55)

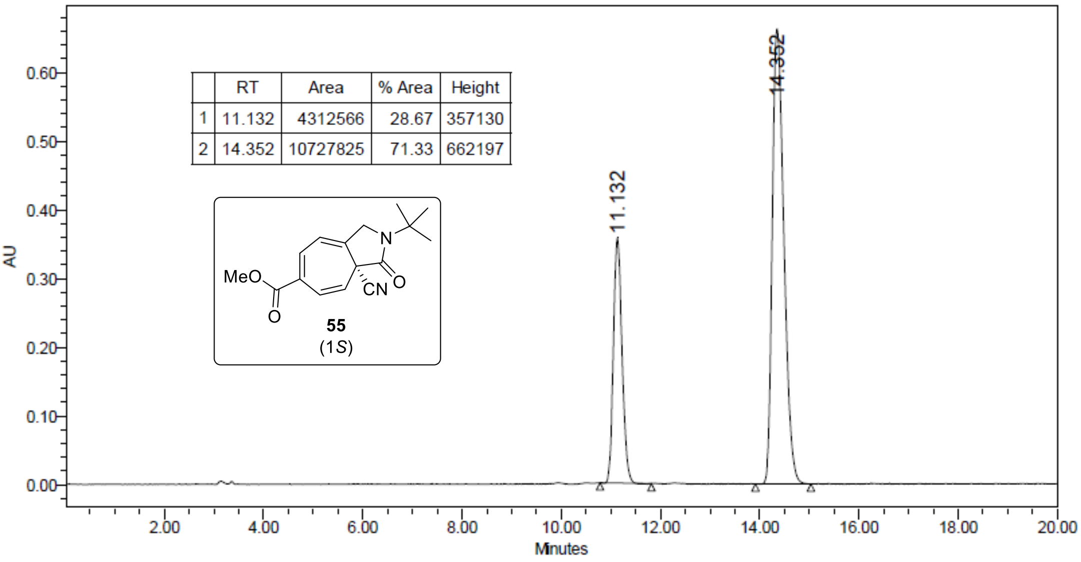

Isolated from reaction of **42 in the presence of Rh_2(2S-FOMePA)$_4$ **9e** (**Table 5**).*

Compound	Column	λ max (nm)	Mobile Phase (Hex:IPA)	Temp (°C)	Flow (mL/min)	Retention Time	
						Enantiomer	min
**55**	Phenomenex Lux® 3μm Amylose-1	209.8	85 : 25	25	1.0	(−) (1*R*)	12
						(+) (1*S*)	15

9-Aza-9-*tert*-butyl-1(*R)-cyano-4-trifluoromethylbicyclo[5.3.0]deca-2,4,6-trien-10-one (56)**

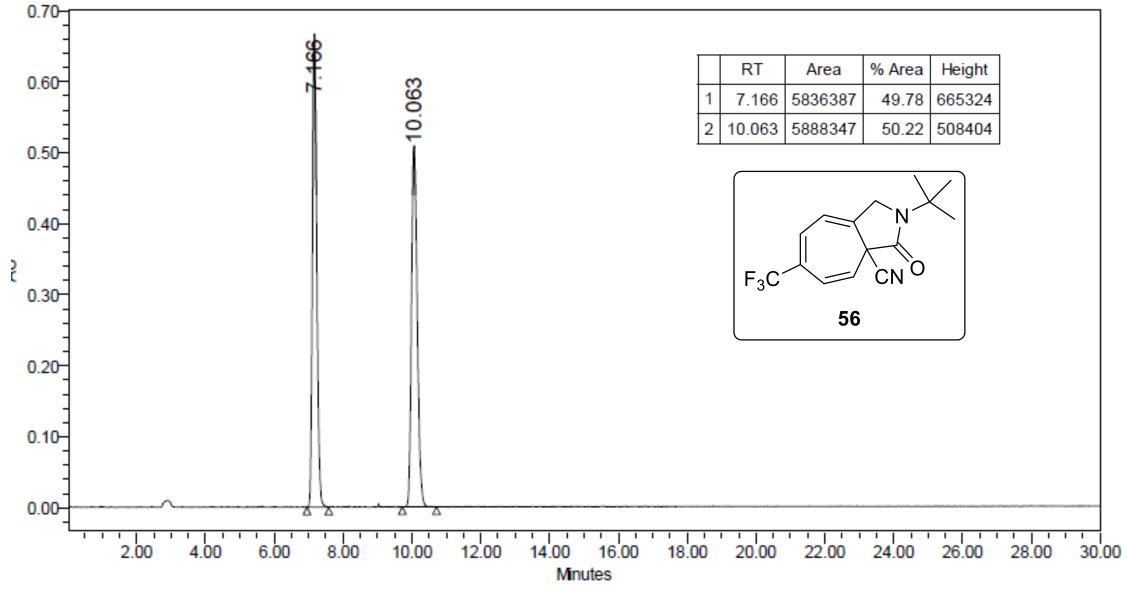

Isolated from reaction of* ***43* *in the presence of* $Rh_2(OAc)_4$ *at room temperature.*

9-Aza-9-*tert*-butyl-1(*R)-cyano-4-trifluoromethylbicyclo[5.3.0]deca-2,4,6-trien-10-one (56)**

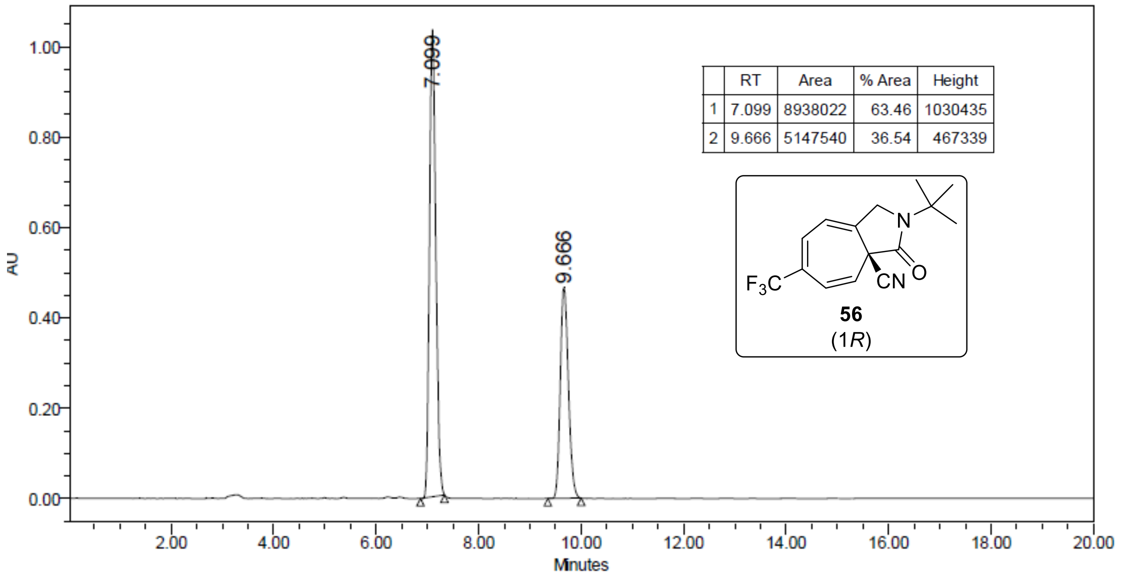

Isolated from reaction of* ***43* *in the presence of* Rh_2*(2S-FOMePA)*$_4$ ***9e*** ***(Table 5****).*

Compound	Column	λ max (nm)	Mobile Phase (Hex:IPA)	Temp (°C)	Flow (mL/min)	Retention Time	
						Enantiomer	min
**56**	Phenomenex Lux® 3μm Amylose-1	209.8	85 : 25	25	1.0	(−) (1*R*)	7
						(+) (1*S*)	10

9-Aza-9-*tert*-butyl-1(*R),4-dicyanobicyclo[5.3.0]deca-2,4,6-trien-10-one (57)**

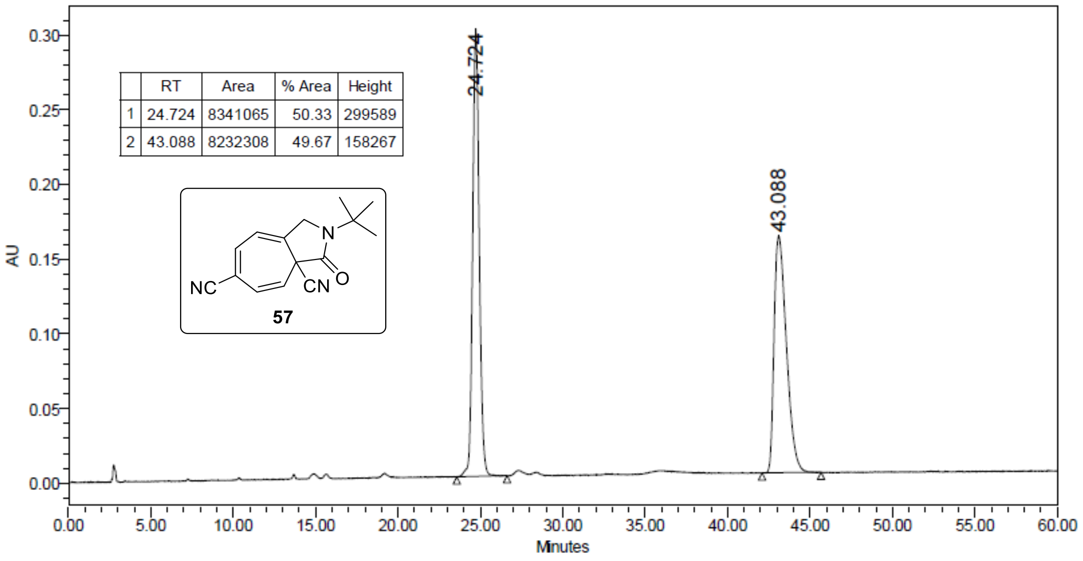

Isolated from reaction of **44 in the presence of $Rh_2(OAc)_4$ at room temperature.*

9-Aza-9-*tert*-butyl-1(*S*),4-dicyanobicyclo[5.3.0]deca-2,4,6-trien-10-one (57)

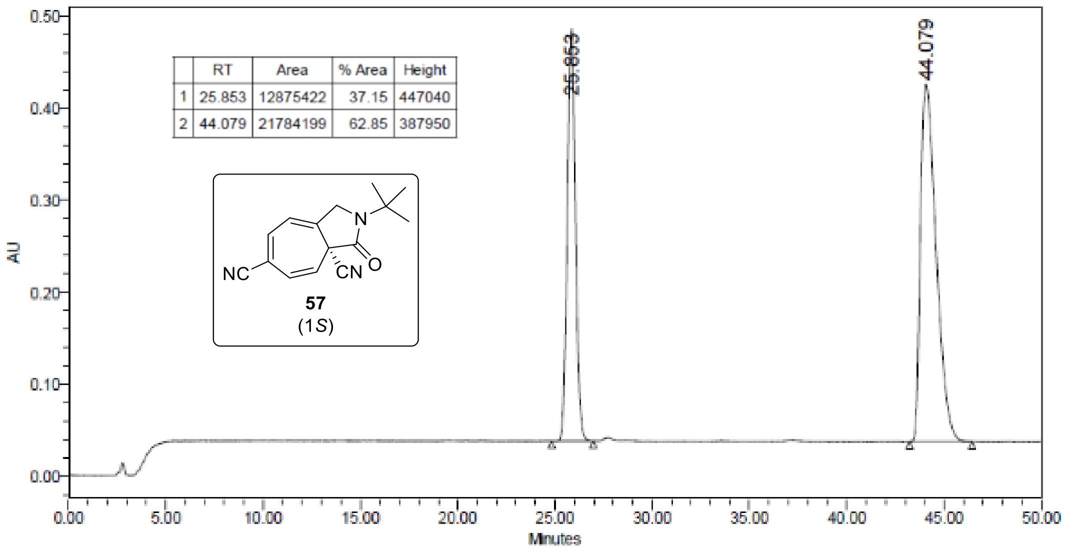

Isolated from reaction of **44 in the presence of $Rh_2(2S\text{-FOMePA})_4$ **9e** (**Table 5**).*

Compound	Column	λ max (nm)	Mobile Phase (Hex:IPA)	Temp (°C)	Flow (mL/min)	Retention Time	
						Enantiomer	min
**57**	Phenomenex Lux® 3μm Amylose-1	209.8	90 : 10	25	0.5	(−) (1*R*)	26
						(+) (1*S*)	44

9-Aza-9-*tert*-butyl-1(*R**)-cyano-4-methoxybicyclo[5.3.0]deca-2,4,6-trien-10-one (58)

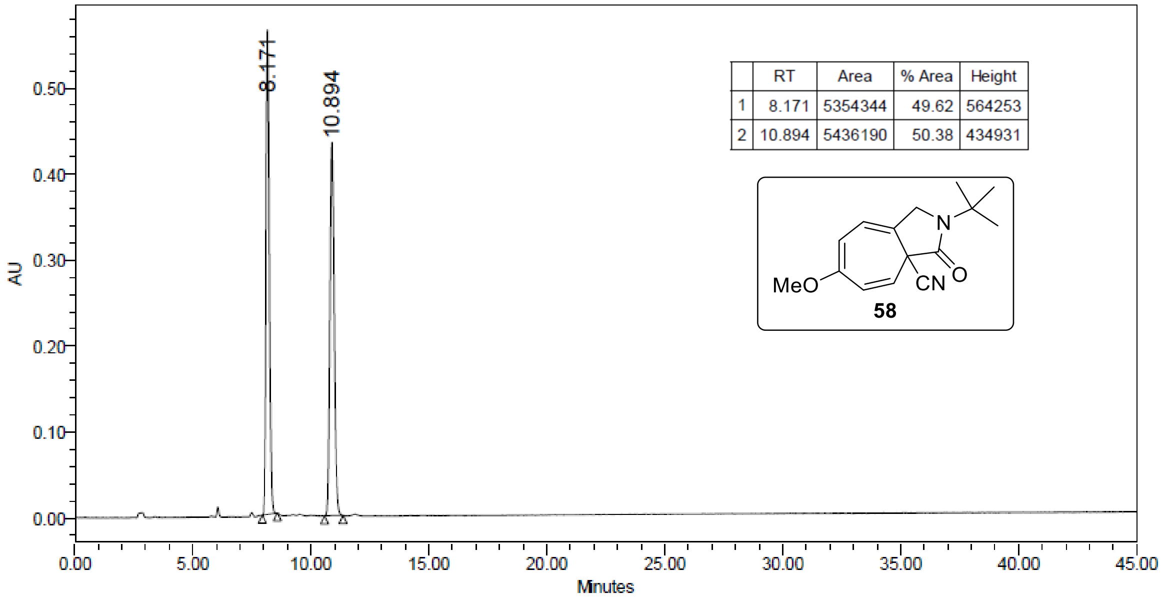

*Isolated from reaction of **45** in the presence of $Rh_2(OAc)_4$ at room temperature.*

9-Aza-9-*tert*-butyl-1(*S*)-cyano-4-methoxybicyclo[5.3.0]deca-2,4,6-trien-10-one (58)

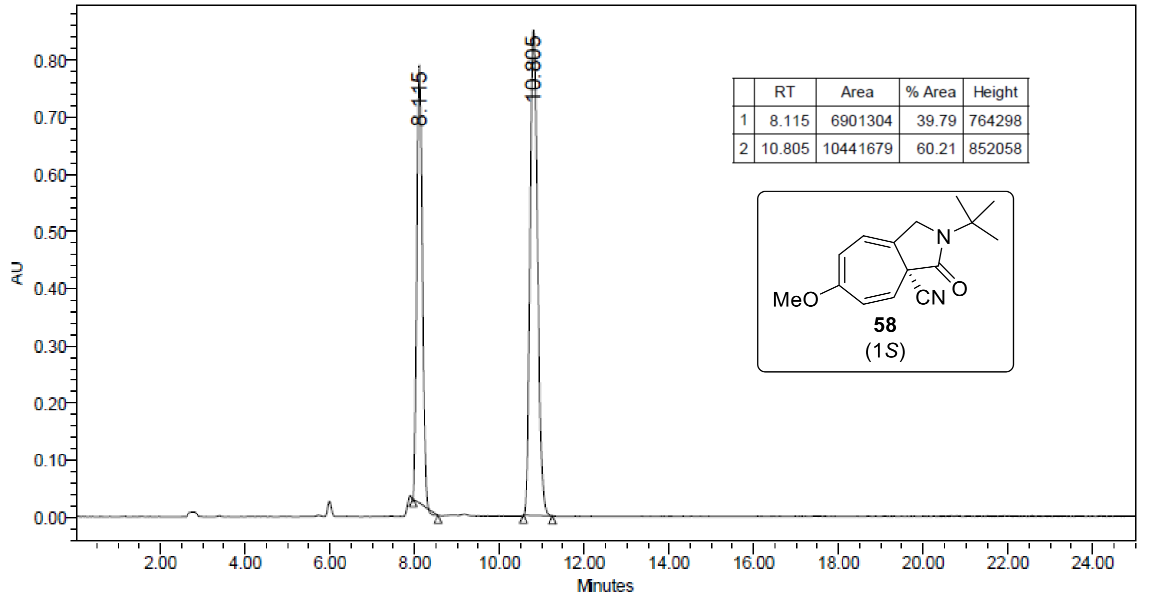

*Isolated from reaction of **45** in the presence of $Rh_2(2S\text{-}FOMePA)_4$ **9e** (**Table 5**).*

Compound	Column	λ max (nm)	Mobile Phase (Hex:IPA)	Temp (°C)	Flow (mL/min)	Retention Time	
						Enantiomer	min
**58**	Phenomenex Lux® 3μm Amylose-1	209.8	85 : 25	25	1.0	(−) (1*R*)	8
						(+) (1*S*)	11

Methyl 9-aza-9-*tert*-butyl-4-methylbicyclo[5.3.0]deca-2,4,6-trien-10-one-1(*R*)-carboxylate (61)

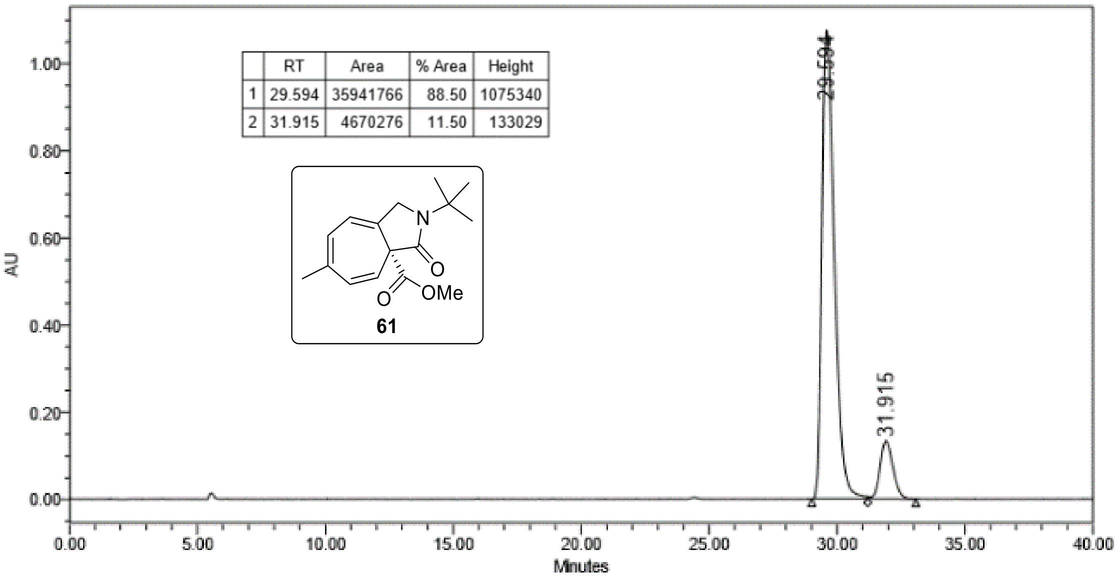

*Basic hydrolysis of enantioenriched **50** (**Scheme 4**).*

**Compound**	**Column**	**λ max (nm)**	**Mobile Phase (Hex:IPA)**	**Temp (°C)**	**Flow (mL/min)**	**Retention Time**	
						**Enantiomer**	**min**
**61**	Phenomenex Lux® 3*µ*m Amylose-1	209.8	85 : 25	25	1.0	(+) (1*R*)	30
						(−) (1*S*)	32

(*R)-2-Allyl-2-methoxycarbonyl-2,3-dihydrobenzofuran-3-one (63)**

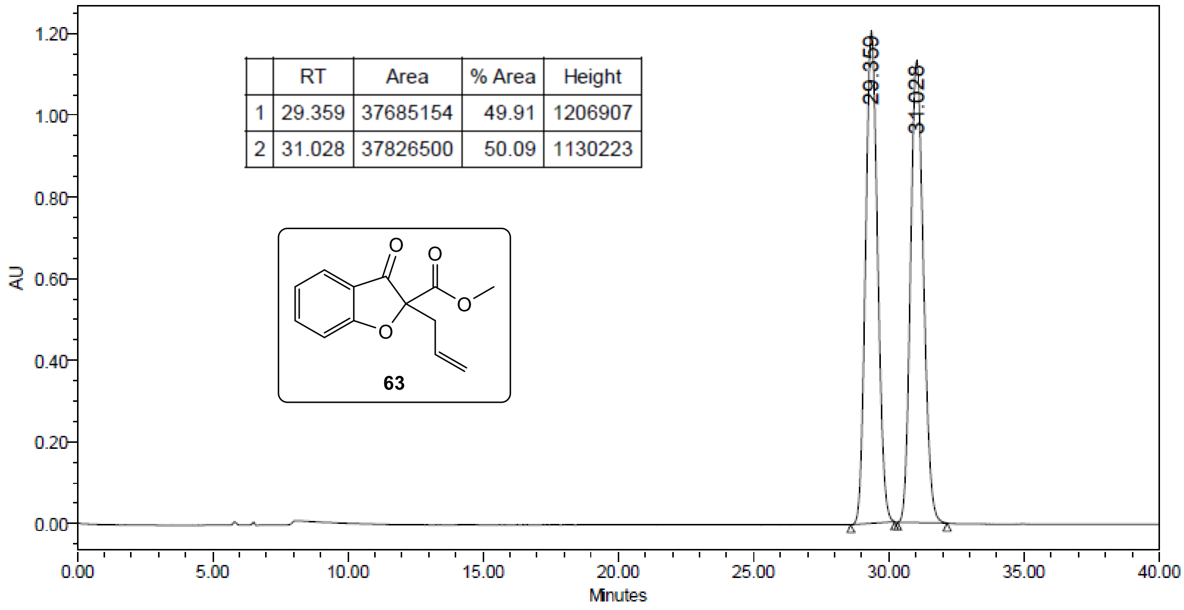

Isolated from the rection of **62 in the presence of $Rh_2(OAc)_4$.*

(*R*)-2-Allyl-2-methoxycarbonyl-2,3-dihydrobenzofuran-3-one (63)

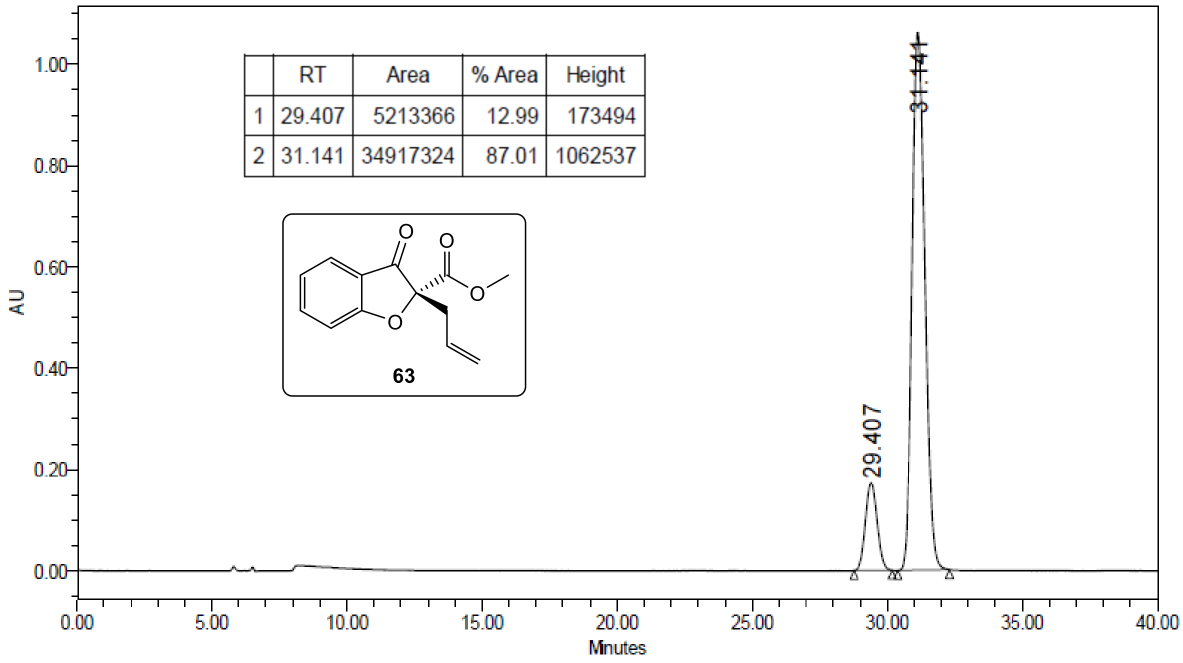

Isolated from the reaction of **62 in the presence of Rh_2(2S-F-2'-NA)$_4$ **9b** (**Scheme 5**).*

**Compound**	**Column**	**λ max (nm)**	**Mobile Phase (Hex:IPA)**	**Temp (°C)**	**Flow (mL/min)**	**Retention Time**	
						**Enantiomer**	**min**
**63**	Phenomenex Lux® 3μm Amylose-1	210	99 : 1	25	0.5	2*S*	29
						2*R*	31

10. Crystal Structures and Data

(+)-*tert*-Butyl (2*S*)-2-(1″*R*,2″*R*,4″*S*)-fenchyloxy-2-(naphthalen-2′-yl)acetate (7b)

The stereochemistry was determined by single crystal X-ray diffraction on a crystalline sample of **(2*S*)-7b** grown by slow evaporation over a number of days from a saturated solution of **(2*S*)-7b** in acetonitrile in a 20 mL vial.

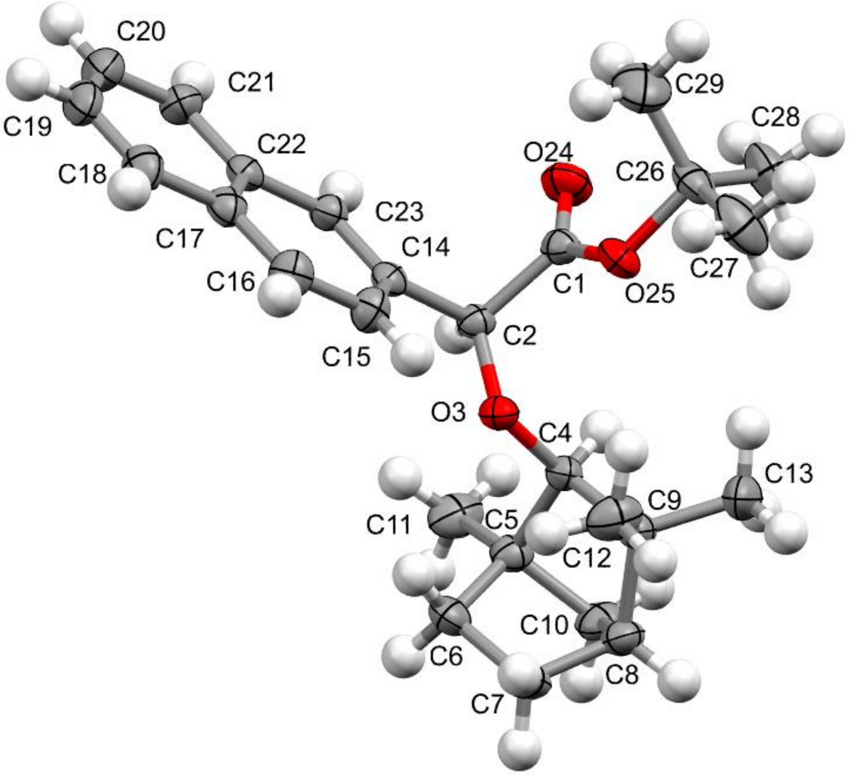

An ORTEP view of ***(2S)-7b*** *showing the structure and relative stereochemistry. Anisotropic parameters are drawn at the 30% probability level.*

The X-ray crystallography data of **(2*S*)-7b** can be found in **Table SI.7**.

(−)-*tert*-Butyl (2*S*)-2-(1″*R*,2″*R*,4″*S*)-fenchyloxy-2-(naphthalen-1′-yl)acetate (7c)

The stereochemistry was determined by single crystal X-ray diffraction on a crystalline sample of **(2*S*)-7c** grown by slow evaporation over a number of days from a saturated solution of **(2*S*)-7c** in acetonitrile in a 20 mL vial.

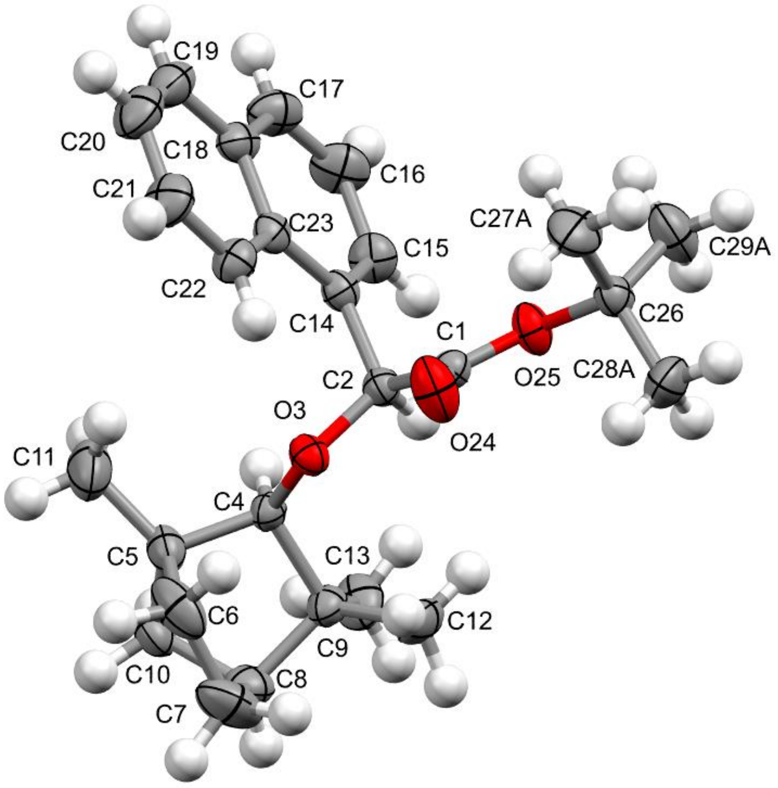

*An ORTEP view of **(2S)-7c** showing the structure and relative stereochemistry. Anisotropic parameters are drawn at the 30% probability level. Only one disordered component is shown for clarity.*

The X-ray crystallography data of **(2*S*)-7c** can be found in **Table SI.7**.

(+)-*tert*-Butyl (2*S*)-2-(4′-bromophenyl)-2-(1″*R*,2″*R*,4″*S*)-fenchyloxyacetate (7d)

The stereochemistry was determined by single crystal X-ray diffraction on a crystalline sample of **(2*S*)-7d** grown by slow evaporation over a number of days from a saturated solution of **(2*S*)-7d** in acetonitrile in a 20 mL vial.

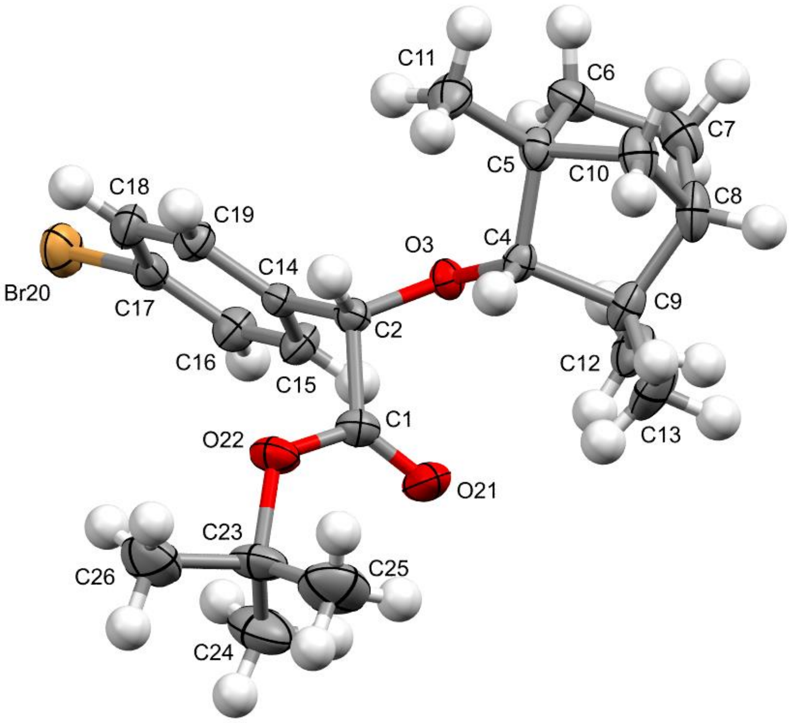

An ORTEP view of ***(2S)-7d*** *showing the structure and relative stereochemistry. Anisotropic parameters are drawn at the 30% probability level.*

The X-ray crystallography data of **(2*S*)-7d** can be found in **Table SI.7**.

(−)-*tert*-Butyl (2*S*)-2-(1″*R*,2″*S*,5″*R*)-menthyloxy-2-phenylacetate (7f)

The stereochemistry was determined by single crystal X-ray diffraction on a crystalline sample of **(2*S*)-7f** grown by slow evaporation over a number of days from a saturated solution of **(2*S*)-7f** in acetonitrile in a 20 mL vial.

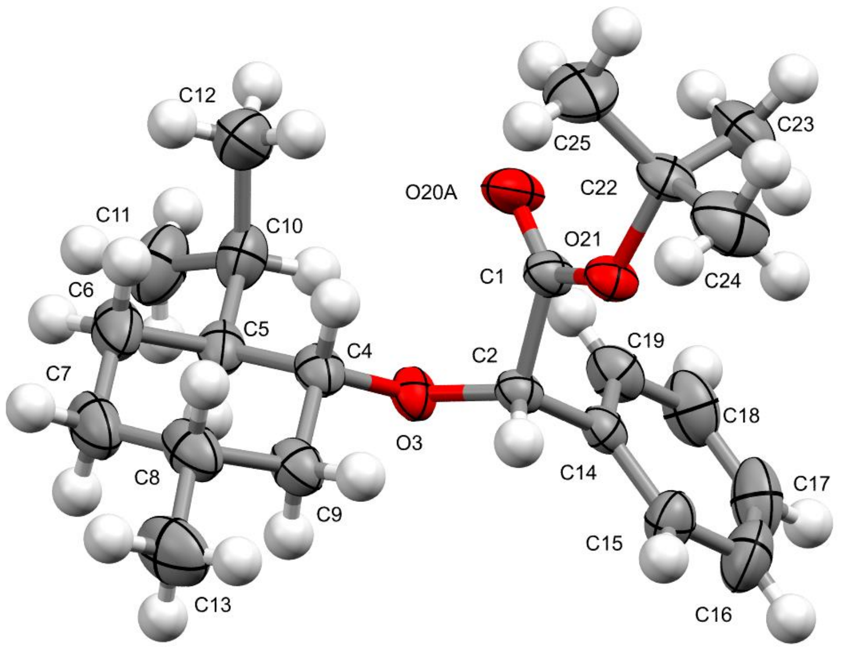

An ORTEP view of ***(2S)-7f*** *showing the structure and relative stereochemistry. Anisotropic parameters are drawn at the 30% probability level.*

The X-ray crystallography data of **(2*S*)-7f** can be found in **Table SI.7**.

(−)-*tert*-Butyl (2*S*)-2-(4′-bromophenyl)-2-(1″*R*,2″*S*,5″*R*)-menthyloxyacetate (7h)

The stereochemistry was determined by single crystal X-ray diffraction on a crystalline sample of **(2*S*)-7h** grown by slow evaporation over a number of days from a saturated solution of **(2*S*)-7h** in acetonitrile in a 20 mL vial.

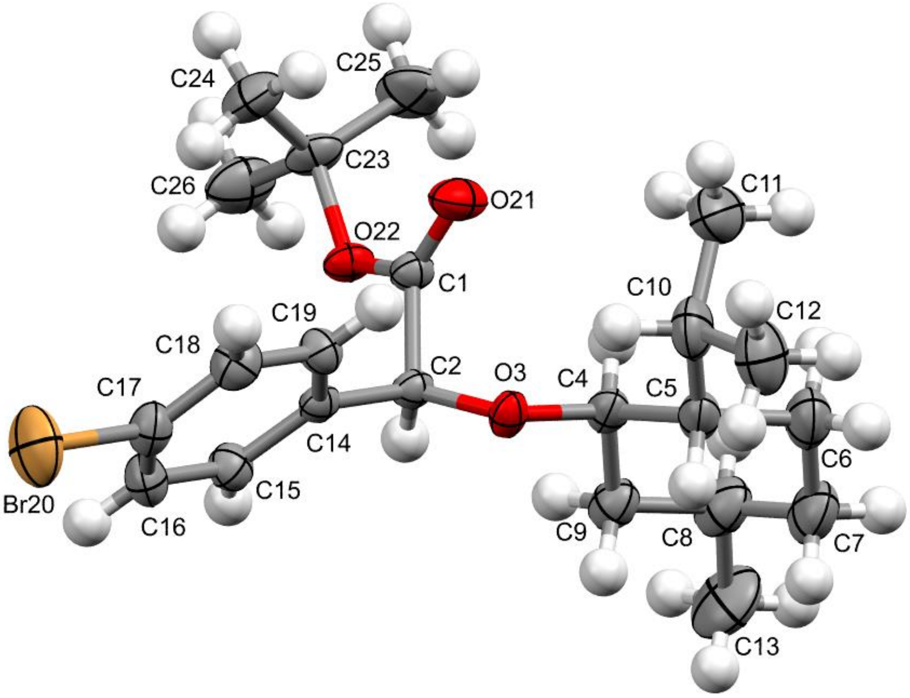

An ORTEP view of ***(2S)-7h*** *showing the structure and relative stereochemistry. Anisotropic parameters are drawn at the 30% probability level.*

The X-ray crystallography data of **(2*S*)-7h** can be found in **Table SI.7**.

(−)-Methyl (2*R*,3*S*)-3-(4'-fluorophenyl)tetrahydro-2*H*-thiopyran-2-carboxylate 1,1-dioxide (27a)

The stereochemistry was determined by single crystal X-ray diffraction on a crystalline sample of **27a** grown by slow evaporation over a number of days from a saturated solution of **27a** in ethyl acetate in a 25 mL roundbottom flask.

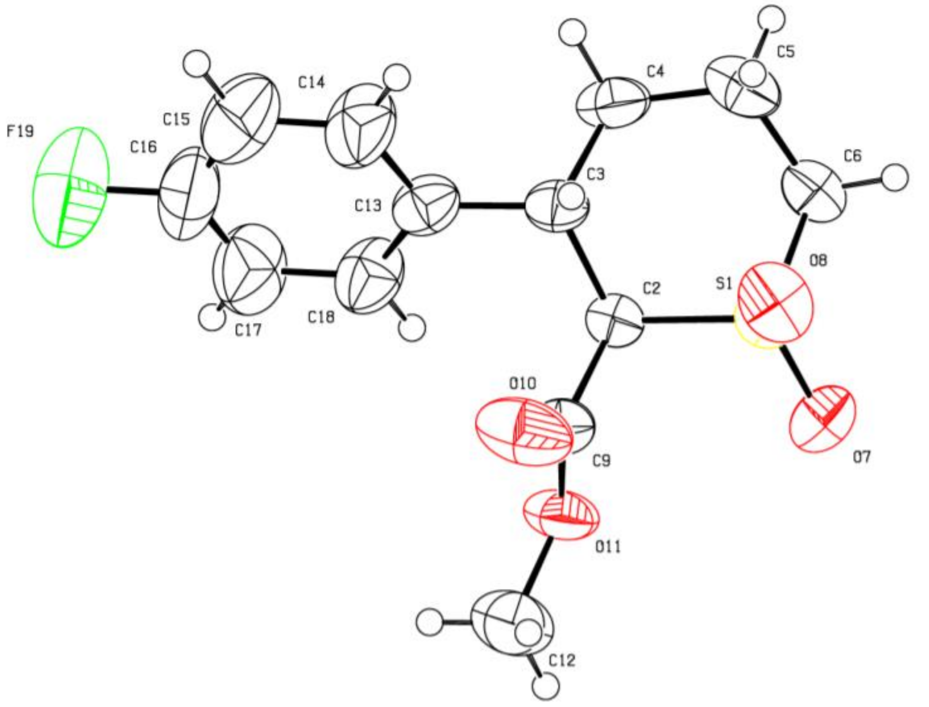

*An ORTEP view of (2R,3S)-**27a** showing the structure and relative stereochemistry. Anisotropic parameters are drawn at the 50% probability level.*

The X-ray crystallography data of **27a** can be found in **Table SI.7**.

(−)-Methyl (1*R*,4a*S*,8a*R*)-octahydro-1*H*-isothiochromene-1-carboxylate 2,2-dioxide (30a)

The stereochemistry was determined by single crystal X-ray diffraction on a crystalline sample of **30a** grown by slow evaporation over a number of days from a saturated solution of **30a** in ethyl acetate in a 25 mL roundbottom flask.

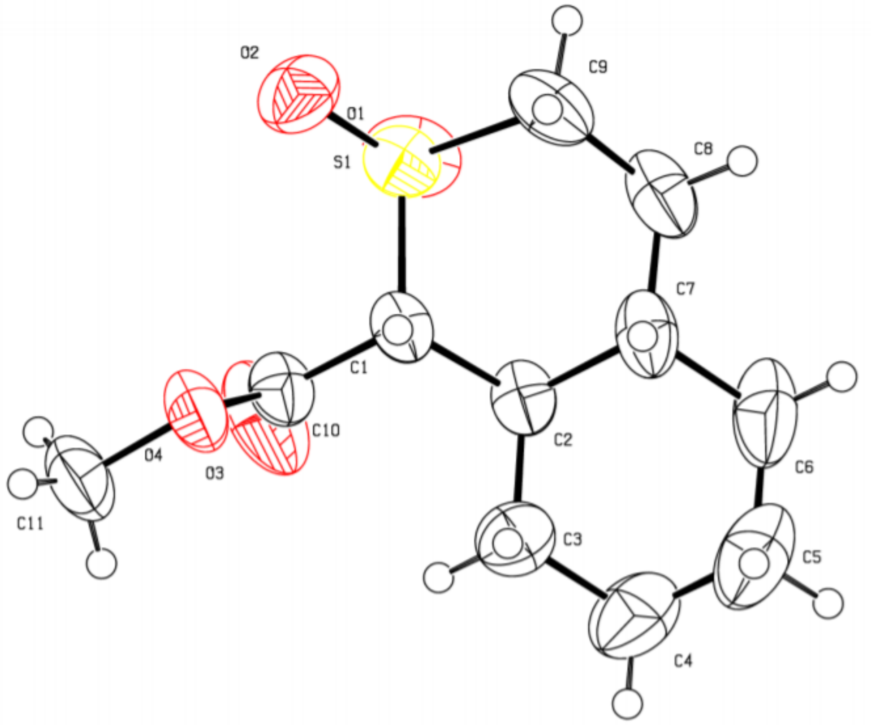

*An ORTEP view of (1R,4aS,8aR)-**30a** showing the structure and relative stereochemistry. Anisotropic parameters are drawn at the 50% probability level.*

The X-ray crystallography data of **30a** can be found in **Table SI.7**.

(+) 9-Aza-9-*tert*-butyl-1(*S*)-cyano-4-bromobicyclo[5.3.0]deca-2,4,6-trien-10-one (47)

The stereochemistry was determined by single crystal X-ray diffraction on crystalline sample of **47** crystallised from chloroform.

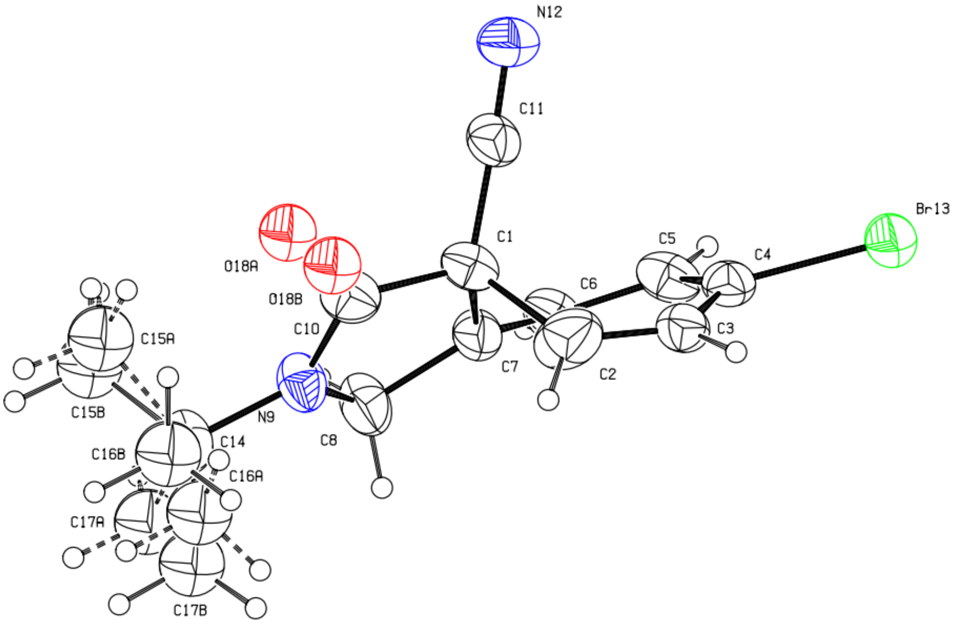

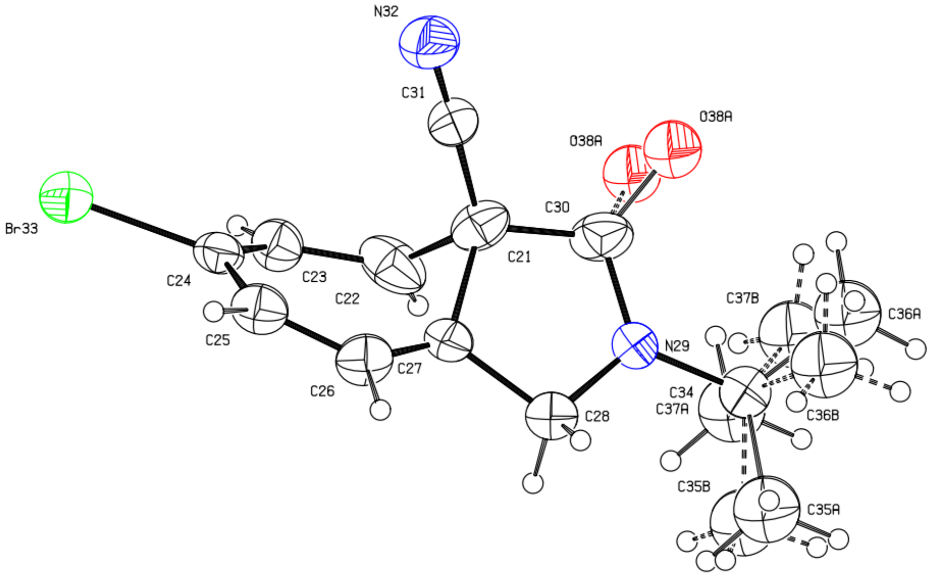

*An ORTEP view of **47** showing the structure and relative stereochemistry. Anisotropic displacement parameters are drawn at the 30% probability level. There is disorder in the* tert-*butyl group.*

The X-ray crystallography data of **47** can be found in **Table SI.7**.

Table SI.8: Crystallographic Data

Compound No	**7b**	**7c**	**7d**	**7f**	**7h**
CCDC No.	2081765	2081766	2081767	2081768	2081769
Crystal System	orthorhombic	orthorhombic	orthorhombic	orthorhombic	orthorhombic
Space Group, *Z*	$P2_12_12_1$, 4	$P2_12_12_1$, 4	$P2_12_12_1$, 4	$P2_12_12_1$, 4	$P2_12_12_1$, 4
Empirical Formula	$C_{26}H_{34}O_3$	$C_{26}H_{34}O_3$	$C_{22}H_{31}BrO_3$	$C_{22}H_{34}O_3$	$C_{22}H_{33}BrO_3$
Formula Weight	394.53	394.53	423.38	346.49	425.39
Crystal size (mm)	0.33 x 0.26 x 0.12	0.60 x 0.55 x 0.12	0.43 x 0.36 x 0.30	0.33 x 0.29 x 0.25	0.30 x 0.27 x 0.24
Wavelength, (Å)	1.54178	0.71073	1.54178	1.54178	1.54178
a (Å)	10.3931(2)	11.0136(15)	6.3385(2)	8.8806(2)	8.7929(2)
b (Å)	14.9104(3)	12.953(2)	11.9385(3)	10.9577(3)	10.8463(3)
c (Å)	15.0088(3)	16.420(3)	29.4096(7)	22.7011(6)	24.7189(6)
α (°)	90	90	90	90	90
β (°)	90	90	90	90	90
γ (°)	90	90	90	90	90
Volume, *V* ($Å^3$)	2325.84(8)	2342.5(6)	2225.49(10)	2209.07(10)	2357.45(10)
Temperature (K)	296(2)	300(2)	296(2)	296(2)	296(2)
Calculated Density, ($g{\cdot}cm^{-3}$)	1.127	1.119	1.264	1.042	1.199
μ (mm^{-1})	0.563	0.071	2.644	0.527	2.496
F(000)	856	856	888	760	896
θ range	4.18 – 67.31	3.10 – 26.82	4.00 – 67.28	5.61 – 67.36	6.18 – 66.93
No. unique reflections	3940	4961	3822	3789	3922
No. observed reflections	3896	3472	3791	3684	3809
R_1 [I>2σ(I)]	0.0356	0.0461	0.0313	0.0395	0.0371
wR_2	0.1020	0.1226	0.0830	0.1142	0.1021
S (Goof)	1.067	1.062	1.105	1.057	1.092
Flack	-0.01(3)	0.0(5)	0.050(6)	0.09(4)	0.024(6)

Compound No	**27a**	**30a**	**47**
CCDC No.	2081770	2081771	2081772
Crystal System	monoclinic	monoclinic	orthorhombic
Space Group, *Z*	$P2_1$, 2	$P2_1$, 2	$P2_12_12_1$
Empirical Formula	$C_{13}H_{15}FO_4S$	$C_{11}H_{18}O_4S$	$C_{14}H_{15}BrN_2O$
Formula Weight	286.31	246.31	307.19
Crystal size (mm)	0.39 x 0.31 x 0.16	0.30 x 0.10 x 0.02	0.35 x 0.29 x 0.25
Wavelength, (Å)	0.71073	1.54178	0.71073
a (Å)	5.0934(9)	5.0811(2)	9.8104(9)
b (Å)	10.1689(19)	10.4886(4)	35.977(3)
c (Å)	13.539(2)	11.6887(4)	8.0365(7)
α (°)	90	90	90
β (°)	96.887(3)	94.910(2)	90
γ (°)	90	90	90
Volume, *V* (Å^3)	696.2(2)	620.65(4)	2836.5(4)
Temperature (K)	296	293	293
Calculated Density, (g·cm^{-3})	1.366	1.318	1.439
μ (mm^{-1})	0.251	2.316	2.889
F(000)	300	264	1248
θ range	1.51 – 27.18	7.60 – 66.56	1.13 – 26.00
No. unique reflections	2730	1144	5474
No. observed reflections	1762	1082	3332
R_1 [I>2σ(I)]	0.0441	0.0544	0.0480
wR_2	0.1048	0.1566	0.1200
S (Goof)	1.031	1.116	1.019
Flack	0.03(6)	0.03(4)	0.009(9)

10. Author Contributions

The synthetic work described in the paper was undertaken by Aoife M. Buckley, Daniel C. Crowley, Thomas A. Brouder and Alan Ford (equal contribution); the crystallography was undertaken by U. B. Rao Khandavilli and Simon E. Lawrence, while Anita R. Maguire had overall responsibility for the project.

11. References

[1] APEX2 v2009.3-0. Bruker AXS Inc, Madison, WI, **2009**.

[2] a) G. M. Sheldrick, *Acta Crystallogr., Sect. C: Struct. Chem.* **2015**, *71*, 3‒8; b) C. F. Macrae, I. J. Bruno, J. A. Chisholm, P. R. Edgington, P. McCabe, E. Pidcock, L. Rodriguez-Monge, R. Taylor, J. van de Streek, P. A. Wood, *J. Appl. Crystallogr.* **2008**, *41*, 466‒470.

[3] T. León, A. Correa, R. Martin, *J. Am. Chem. Soc.* **2013**, *135*, 1221-1224.

[4] J. Ke, C. He, H. Liu, H. Xu, A. Lei, *Chem. Commun.* **2013**, *49*, 6767-6769.

[5] S. A. Lee, *Fluorine Chem.* **2001**, *109*, 55-57.

[6] W. A. Moradi, S. L. Buchwald, *J. Am. Chem. Soc.* **2001**, *123*, 7996-8002.

[7] R. J. Delley, S. Bandyopadhyay, M. A. Fox, C. Schliehe, D. R. W. Hodgson, F. Hollfelder, A. J. Kirby, A. C. O'Donoghue, *Org. Biomol. Chem.* **2012**, *10*, 590-596.

[8] D. Zhao, Y. Jiang, D. Ma, *Tetrahedron* **2014**, *70*, 3327-3332.

[9] X. Ma, S. B. Herzon, *J. Org. Chem* **2016**, *81*, 8673-8695.

[10] Z. Song, Y. Wu, T. Xin, C. Jin, X. Wen, H. Sun, Q.-L. Xu, *Chem. Commun.* **2016**, *52*, 6079-6082.

[11] T. Hashimoto, Y. Naganawa, T. Kano, K. Maruoka, *Chem. Commun.* **2007**, 5143-5145.

[12] P. G. Mattingly, M. J. Miller, *J. Org. Chem* **1981**, *46*, 1557-1564.

[13] U. B. R. Khandavilli, A. M. Buckley, A. R. Maguire, M. S. R. N. Kiran, U. Ramamurty, S. E. Lawrence, *CrystEngComm* **2021**.

[14] G. H. P. Roos, M. A. McKervey, *Synth. Commun.* **1992**, *22*, 1751-1756.

[15] J. Lee, J.-H. Lee, S. Y. Kim, N. A. Perry, N. E. Lewin, J. A. Ayres, P. M. Blumberg, *Biorg. Med. Chem.* **2006**, *14*, 2022-2031.

[16] E. Mannekens, D. Tourwé, W. D. Lubell, *Synthesis* **2000**, *2000*, 1214-1216.

[17] H. Saito, H. Oishi, S. Kitagaki, S. Nakamura, M. Anada, S. Hashimoto, *Org. Lett.* **2002**, *4*, 3887-3890.

[18] S. P. Pappas, B. C. Pappas, J. E. Backwell, *J. Org. Chem.* **1967**, *32*, 3066-3069.

[19] a) A. E. Shiely, L.-A. Clarke, C. J. Flynn, A. M. Buckley, A. Ford, S. E. Lawrence, A. R. Maguire, *Eur. J. Org. Chem.* **2018**, 2277–2289; b) C. J. Flynn, C. J. Elcoate, S. E. Lawrence, A. R. Maguire, *J. Am. Chem. Soc.* **2010**, *132*, 1184‒1185.

[20] T. A. Brouder, C. N. Slattery, A. Ford, U. B. R. Khandavilli, E. Skořepová, K. S. Eccles, M. Lusi, S. E. Lawrence, A. R. Maguire, *J. Org. Chem.* **2019**, *84*, 7543-7563.

[21] S. Mo, J. Xu, *ChemCatChem* **2014**, *6*, 1679-1683.

[22] A. Feceu, L. E. Sangster, D. B. C. Martin, *Organic Letters* **2018**, *20*, 3151-3155.

[23] T. Ye, C. F. Garcia, M. A. McKervey, *J. Chem. Soc., Perkin Trans. 1* **1995**, 1373-1379.

[24] J. Fu, H. Shang, Z. Wang, L. Chang, W. Shao, Z. Yang, Y. Tang, *Angew. Chem. Int. Ed.* **2013**, *52*, 4198-4202.
